# Additive-Controlled Regioswitching in Ni-Catalyzed Enantioselective Hydrophosphination of Unactivated Alkenes

**DOI:** 10.1021/jacs.5c19022

**Published:** 2026-01-13

**Authors:** Jian Zhou, Sichen Tao, Xinglong Zhang, Jun Wang

**Affiliations:** † Department of Chemistry, 26679 Hong Kong Baptist University, Kowloon, 999077 Hong Kong, China; ‡ Department of Chemistry, 26451 The Chinese University of Hong Kong, Shatin, New Territories, Kowloon, 999077 Hong Kong China

## Abstract

Transition metal-catalyzed asymmetric hydrophosphination of unsaturated bonds offers the most direct route to chiral organophosphorus compounds. However, unactivated double bonds remain a longstanding challenge in this field due to their inherent low reactivity and the difficulty in achieving precise enantio- and regiocontrol. Herein, we report an amide-assisted asymmetric and regiodivergent hydrophosphination of unactivated alkenes catalyzed by a synergistic Ni­(cod)_2_/BenzP and Brønsted acid system. Mechanistic studies and density functional theory calculations reveal that the weak noncovalent interactions between the amide substrate and the ligand are critical for selectivity. Diverging from conventional migratory insertion pathways, this strategy leverages distinct hydronickelation pathways mediated solely by pyridine-3-sulfonic acid or 3,5-difluorophenol additives, enabling precise control over enantioselectivity and regioselectivity. All of the branched and linear products are accessed with excellent regiodivergence, showcasing a versatile platform for the modular synthesis of chiral organophosphorus compounds.

## Graphical abstract

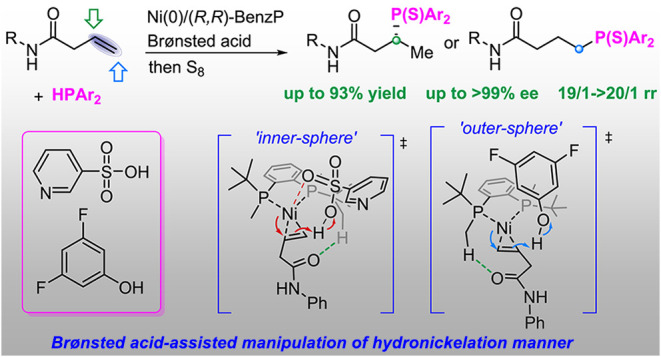

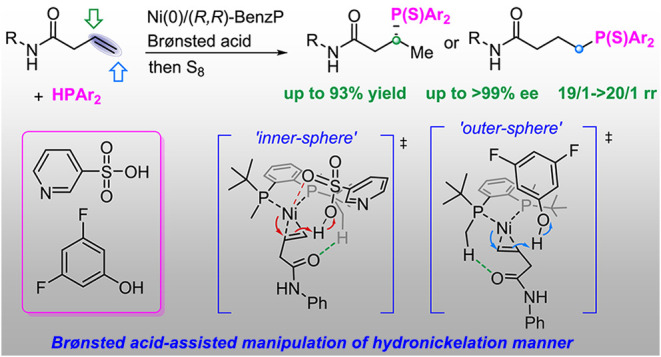

## Introduction

Unactivated olefins are simple, abundant structures derived from petrochemical feedstocks and traditional synthesis methods. Their scalability and versatility make them highly attractive building blocks in modern organic chemistry.^1,2^ Transition metal-catalyzed asymmetric hydrofunctionalization of C=C bonds represents a powerful strategy for the construction of enantiomerically enriched compounds with exceptional atom economy. However, achieving precise enantio- and regiocontrol in these reactions remains a formidable challenge, hindered by three inherent limitations: (i) the weak binding affinity of unactivated alkenes to the transition metal center, (ii) chain-walking isomerization during hydrometalation, and (iii) the difficulty in differentiating the prochiral faces and reaction sites due to the substrate’s nonpolar nature and subtle steric distinction.^3^ Current regiocontrol strategies predominantly rely on transition metal–ligand combinations that dictate reaction pathways via the Chalk–Harrod mechanism or its modified Chalk–Harrod mechanism, where the sequence of hydride and functional group transfer determines regioselectivity. Generally, it is formidable to tune elementary steps or the corresponding intermediates once the transition metal–ligand is selected.^4,5^ Consequently, achieving regiodivergence and enantiocontrol within the same catalytic framework in the hydrometalation step of double bonds through the Chalk–Harrod mechanism has remained elusive and presents a tremendous challenge. Therefore, the development of additive-mediated pathway switching represents a breakthrough strategy to realize distinctive regiocontrol. This approach not only provides valuable insights into the rational selection of additives for tunable catalysis but also significantly enhances synthetic utilities in alkene hydrofunctionalization (Scheme 1A).

**Scheme 1:**
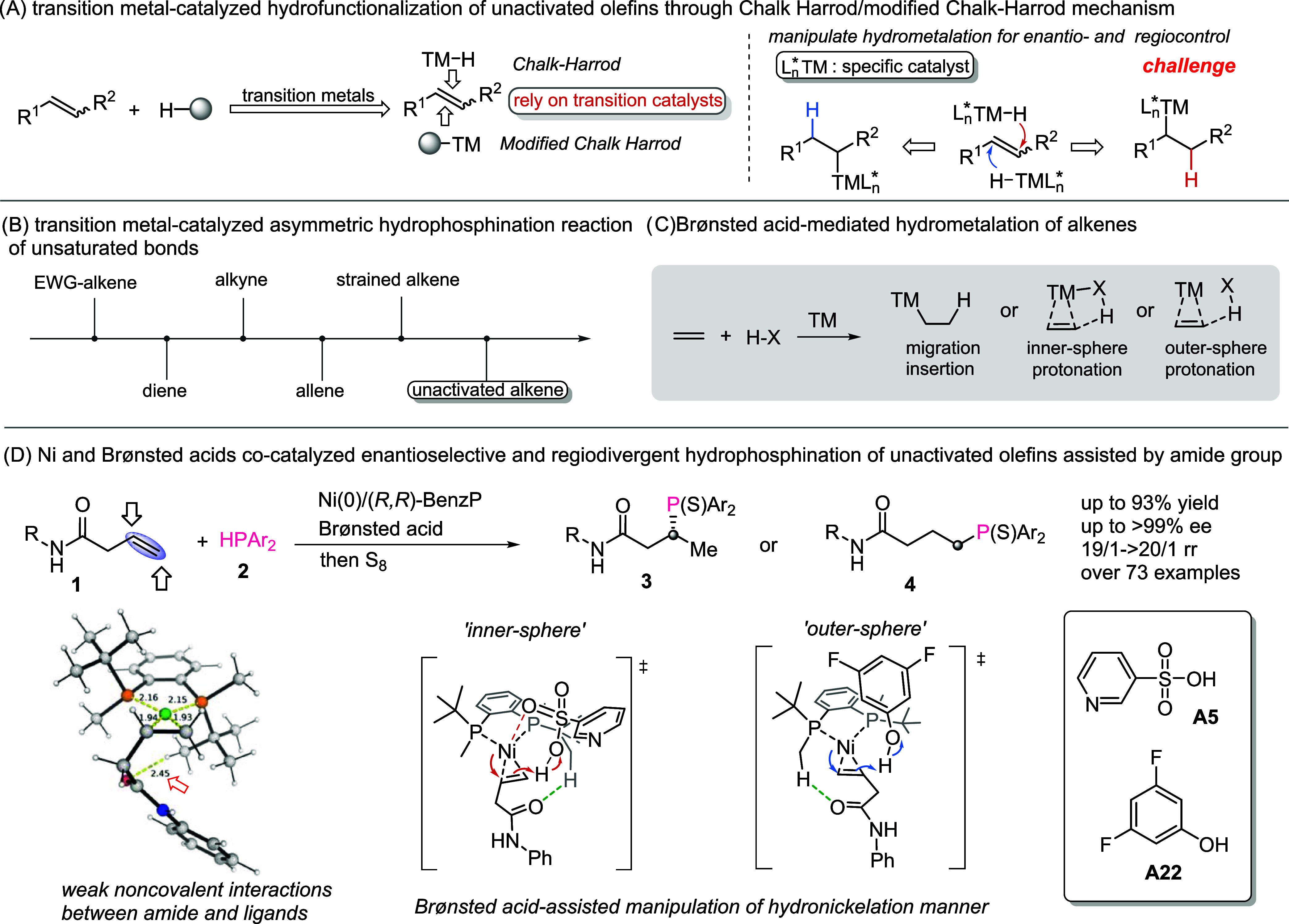
Proposed Enantioselective and Regiodivergent Hydrophosphination of Unactivated Alkenes

Transition metal-catalyzed hydrophosphination of unsaturated bonds has gained significant attention in recent years for the construction of chiral phosphorus compounds that demonstrate wide application in pharmaceuticals^6^ and functionalized materials,^7^ especially asymmetric catalysis.^8–11^ Despite the remarkable advances made in transition metal-catalyzed asymmetric nucleophilic addition of P–H reagents to various unsaturated compounds, such as EWG alkenes,^12–22^ dienes,^23,24^ allenes,^25–27^ alkynes,^28–35^ and strained alkenes,^36–41^ the corresponding transformation of unactivated alkenes still lags behind. Recently, Zhang and Yang reported Pd-catalyzed enantioselective hydrohosporylation of styrenes, where the utilization of different ligands allowed the regiodivergent transformation. However, a limitation in the scope of H-phosphonate reagents restricts its broader application.^42^ Thus, developing a general protocol for such enantio- and regioselective transformations of unactivated olefins remains both challenging and highly desirable (Scheme 1B). Moreover, regiodivergent access to such a transformation through precisely regulating the hydrometalation pathway of the C=C bond with the use of a specific catalyst is still a considerable synthetic challenge to date. Beyond the insertion of unsaturated bonds into TM–P species through the modified Chalk–Harrod mechanism, a competing pathway involving the hydrometalation of unsaturated bonds by TM–H species after the oxidative addition of a transition metal to the P–H bond is implicated. The reductive side product formed by H_2_ and the poor addition selectivity to unsaturated bonds are particularly problematic in this context.^43–48^ Being able to enhance the reactivity of substrates and assert the control of enantioselectivity, coordination assistance has been considered an effective strategy in varied hydrofunctionalization reactions of unactivated olefins, such as hydroamination, hydroboration, hydroarylation, hydrosilylation, and hydroacylation.^49^ We envisioned that the use of a directing group probably facilitates the reactivity and enantiotopic face-discriminating step in the hydrophosphination of unactivated olefin. On the other hand, Brønsted acids have been shown to facilitate the hydrometalation of unsaturated bonds through three distinct pathways: the insertion of unsaturated bonds into formed transition metal hydride species via migratory insertion,^23^ the process of ligand-to-ligand hydrogen transfer (LLHT)/inner-sphere protonation, or outer-sphere protonation in the formal hydronickelation step of the unsaturated bonds (Scheme 1C).^28,50–56^ By varying different Brønsted acids, the regiocontrol in the hydrometalation step might be potentially manipulated through the Chalk–Harrod mechanism within the same catalyst. Several key issues need to be considered in this proposed protocol: (a) an auxiliary group with native chemical functionality such as the amide group with a weak coordination ability is highly desirable;^57^ (b) given the unexpected formation of conjugated double bonds through the undesirable chain-walking process, the establishment of chirality might be affected; and (c) effective Brønsted acids that enable regiocontrol by precisely modulating the hydrometalation step of the catalyst to C=C bonds should be identified. To extend our research interests in asymmetric hydrofunctionalization reactions, particularly the hydrophosphination reaction of unsaturated bonds catalyzed by transition metals,^25,29,32,37–39^ herein, we identified a Ni-catalyzed enantioselective and regiodivergent hydrophosphination reaction of unactivated alkenes. DFT calculations demonstrate a special auxiliary effect through weak nonconvalent interactions between the carbonyl group of the substrates and the tertbutyl/methyl group on the ligand and the crucial role of Brønsted acids in achieving distinctive and precise regiocontrol via the hydronickelation of C=C bonds (Scheme 1D).

## Results and Discussion

The exploration of the amide-directed hydrophosphination reaction of unactivated alkenes began with *N*-phenylbut-3-enamide (**1a**) and diphenylphosphane (**2a**) as benchmark substrates. We first examined the Pd/L*_n_* * system that was developed in the hydrophosphination of heterobicyclic alkene, cyclopropene, and methylenecyclopropane (Table S1).^37–39^ The mixture of both anti-Markovnikov and Markovnikov products was obtained, with poor regio- and enantioselectivity. Then, we shifted our focus to a nickel catalyst, an inexpensive and abundant metal known for its unique reactivity profile. Various chiral ligands including BINAP, BenzP, Ph-BPE, and QunixoP were screened at the preliminary exploration; however, Ni­(cod)_2_/BenzP delivered **3aa** with a 22% yield and 20% ee at 120 °C and a reductive side product was observed. Besides, the whole process was impeded at a lower reaction temperature (Table S2). Therefore, we hypothesized that the introduction of Brønsted acids might promote the hydronickelation of the C=C bond and facilitate enantiocontrol through following ligand exchange with **2a**. When 20 mol % *p*TSOH **A1** was used, a significant improvement of the yield and ee value was observed; especially, (*R*, *R*)-BenzP gave a 33% yield and 96% ee. Halving the dosage of *p*TsOH **A1** resulted in a decrease in the enantioselectivity, demonstrating the predominant effect of the acid. After extensive screening of acids, pyridine-3-sulfonic acid **A5** increased the yield of **3a** with 95% ee. Since no sign of Ni-H species was detected, the regiocontrol through the subtle inner-sphere hydronickelation pathway assisted by Brønsted acids was expected to be operative. As anticipated, 3,5-difluorophenol **A22** and pivalic acid **A15** delivered anti-Markovnikov products **4aa** with excellent regiocontrol (Table S4). These results testify to the success of our strategy to establish enantioselectivity and manipulate regiocontrol relying on Brønsted acids.

With the optimized conditions established, we examined the scope of unactivated alkenes using a phosphorus nucleophile **2a** as a model substrate (Table 1). Various *N*-phenylbut-3-enamides containing electron-deficient or electron-donating groups at the arene backbone were tested with this protocol, giving Markovnikov products with good yields and extremely excellent enantioselectivities and regioselectivities. Notably, **3a**, **3e**, **3f**, **3g**, **3k**, **3n**, **3o**, **3q**, **3r**, **3s**, **3u**, and **3ac** were obtained with 99% ee values. Heterocycle-contained substrates also performed well in this process, with good yields and excellent enantioselectivies for thiophene, quinoline, and dibenzofuran. In addition, alkane amide-containing substrates were also compatible. Natural amino acid derivatives, l-tryptophan and l-alanine, could be converted into products **3aa** and **3ab**, achieving yields of 53 and 82%, respectively, with both exhibiting an outstanding diastereomeric ratio greater than 20:1. Particularly, when this methodology was extended to an unactivated internal alkene, product **3ad** was afforded with a 95% ee value and a >20/1 rr value. Then, the scope of the phosphorus reagents was investigated. Substituents on *para*- and 3,5-disubstituted positions were well tolerated, producing **3ae** and **3af** with >99 and 97% ee, respectively. To our delight, various secondary phosphine oxides with diverse electronic properties formed through the *in situ* reduction of secondary phosphine oxides with inexpensive silanes were compatible and delivered the corresponding products smoothly. Moreover, phosphines with large steric hindrances performed well, yielding **3ah**, **3al**, and **3am** with 80–94% ee values and >20/1 regioselectivities.

**Table 1:**
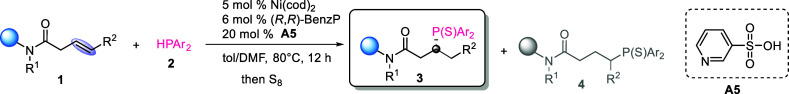

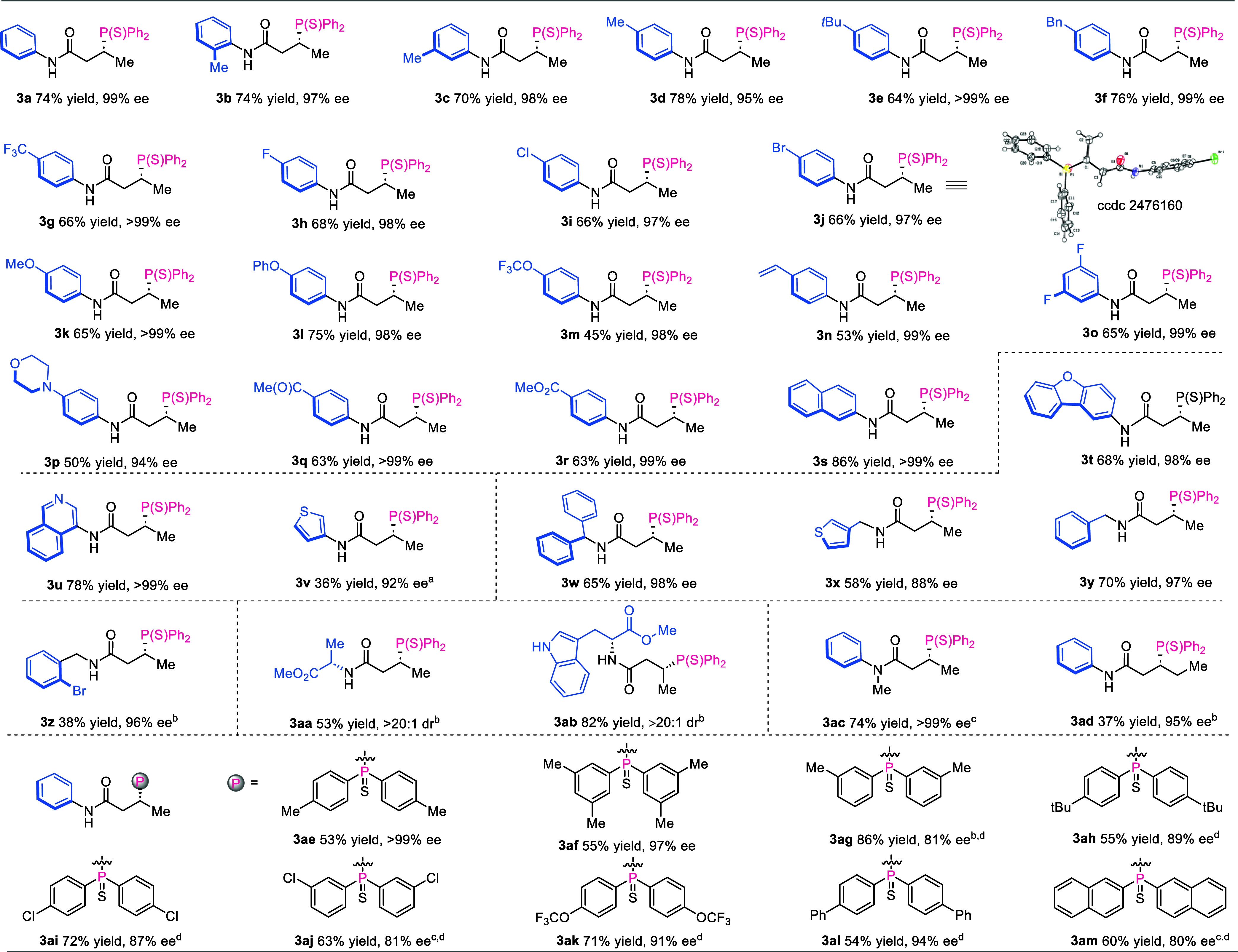
Substrate Scope of the Unactivated Olefins with Organophosphorus Compounds for Markovnikov-Type Products^a^

a Reaction conditions: Ni­(cod)_2_ (5 mol %) and (*R,R*)-Benzp (6 mol %) in toluene/DMF (0.48/0.02 mL) were stirred at rt for 20 min under argon. Then, **A5** was added and stirred for 10 min; **1** (0.22 mmol) and **2** (0.1 mmol) were added, and the reaction mixtures were stirred at 80 °C for 12 h. Then, they were oxidized by S_8_. Isolated yields were obtained. The rr values of product **3** were >20/1 determined by ^31^P NMR of the reaction mixture.

b At 100 °C.

c At 90 °C.

d HPAr_2_ was reduced from HP­(O)­Ar_2_ *in situ* and reacted for 36 h.

Next, we turn our attention to study the generality of anti-Markovnikov reactions mediated by 3,5-difluorophenol or pivalic acid (Table 2). In addition to an array of different electron-donating or electron-withdrawing substitutions on the arene of *N*-phenylbut-3-enamides, other functional groups including ester, acetyl, and morpholine groups were also compatible under this reaction, delivering the corresponding products with good yields and excellent regioselectivities. Substrates with heterocycles and natural amino acid derivatives, such as l-(+)-α-phenylglycine and l-alanine, also reacted to form desired Markovnikov derivatives in good yields and >20/1 rr values. This protocol was found to be applicable to electron-deficient or electron-rich phosphorus nucleophiles, resulting in target products in good yields and excellent regiocontrol.

**Table 2:**
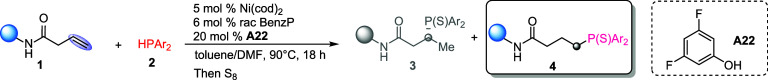

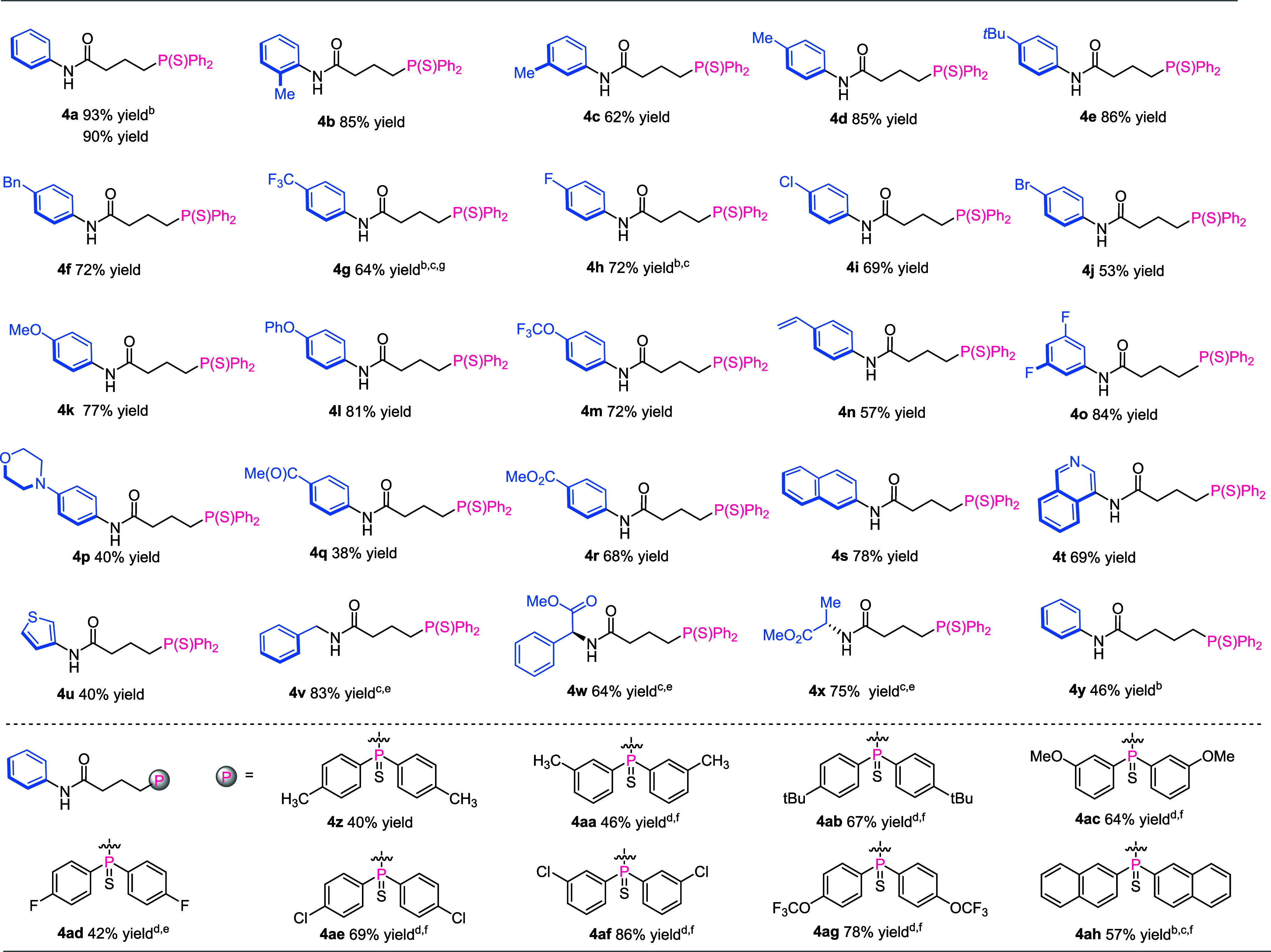
Substrate Scope of the Unactivated Olefins with Organophosphorus Compounds for Anti-Markovnikov-Type Products^a^

a Reaction conditions: Ni­(cod)_2_ (5 mol %) and rac Benzp (6 mol %) in toluene/DMF (0.48/0.02 mL) were stirred at rt for 20 min under argon. Then, **A22** was added and stirred for 10 min; **1** (0.22 mmol) and **2** (0.1 mmol) were added, and the reaction mixtures were stirred at 90 °C for 18 h. Then, they were oxidized by S_8_. Isolated yields were obtained. The rr values of product **4** were >20/1 determined by ^31^P NMR of the reaction mixture.

b DMF as the solvent.

c 20 mol % **A15** was used.

d 40 mol % **A15** was used.

e At 100 °C.

f HPAr_2_ was reduced from HP­(O)­Ar_2_ *in situ* and reacted at 100 °C for 36 h.

g The rr value was 19/1.

To further demonstrate the practical utility of this approach (Scheme 2), product **3s** was delivered with 98% ee in the gram-scale synthetic experiments. Linear product **4a** was obtained at a 1.0 mmol scale in a 62% yield. After facile reduction of the amide group of **3a′** and **4a**, compounds **5** and **8** can be further transformed to chiral γ-amino phosphine sulfide **6** with 99% ee and δ-amino phosphine sulfide **10**, as well as a phosphine-thiourea catalyst precursor **7** with 98% ee and **9**, respectively. In addition, compounds **3aa**, **3ab**, **4w**, and **4x** may be hydrolyzed into the corresponding chiral organophosphorus amino acids in high yields.

**Scheme 2:**
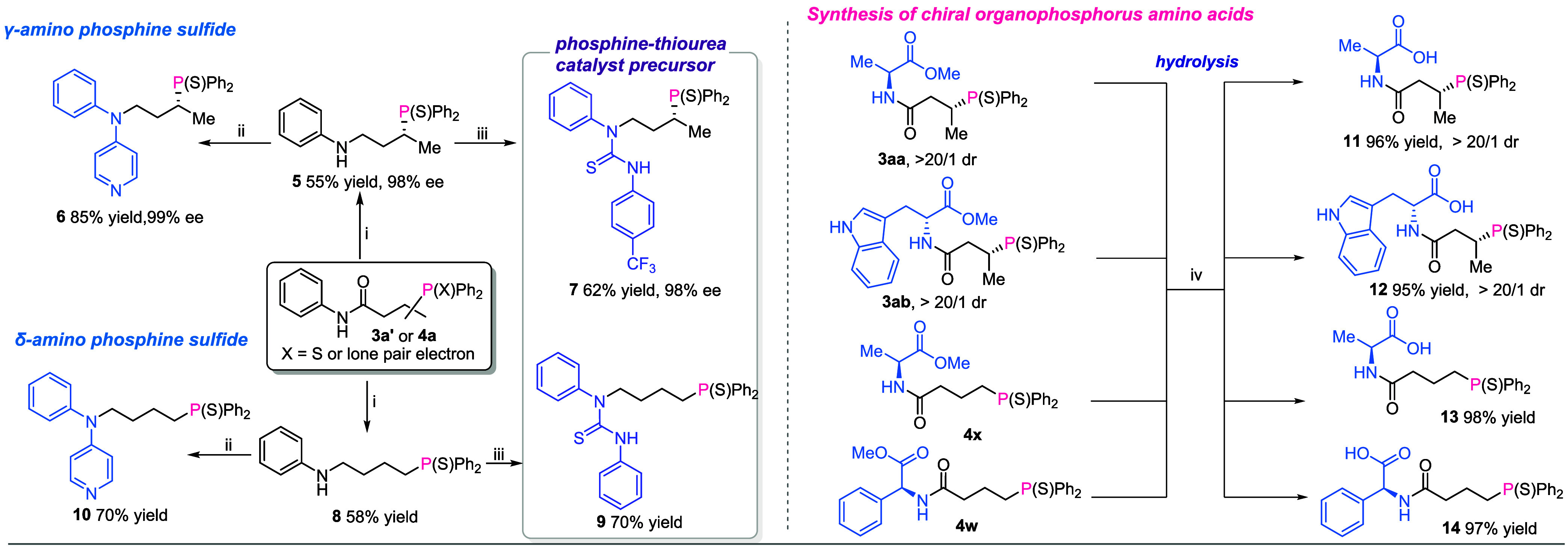
Synthetic Transformation of Products^a^

a Reaction conditions: (i) Co­(acac)_2_/DPEPhos, HSiPh_3_, THF, 70 °C. (ii) PC­(3DPAFIPN), DABCO, blue LEDs, MeCN, rt. (iii) 3,5-Bistrifluoromethylphenyl isothiocyanate, DCM, rt. (iv) LiOH, MeOH/H2O (v/v) = 10/1, rt.

To gain insights into the reaction mechanism, the observation of deuterium at α-, β-, and γ-positions of the carbon chain demonstrated a chain-walking process (Figure 1i and (ii)). Both (*E*)-*N*-phenylbut-2-enamide **17** and (*Z*)-*N*-phenylbut-2-enamide **17** as substrates in standard conditions gave ***R*** **-3a** with 99% ee, but in 70 and 50% yields, respectively (Figure 1iii). 51% deuterium found in the γ-position suggested that the unactivated alkene formed by isomerization is the predominant reaction intermediate in this protocol (Figure 1iv). Besides, similar chain walking was found with 3,5-difluorophenol used as additives (Figure 1vii); however, no Markovnikov product was delivered with (*E*)-*N*-phenylbut-2-enamide used as the substrate, indicating a distinct hydronickelation step compared to the reaction mode involving pyridine-3-sulfonic acid (Figure 1ix). Furthermore, phenyl but-3-enoate **15** as substrates in standard conditions for the regiodivergent transformation delivered no corresponding anti-Markovnikov/Markovnikov products **16** and **20** (Figure 1v,viii). In addition, allylbenzene **18** could not be transformed into Markovnikov product **19**, and anti-Markovnikov product **21** was obtained only in a 15% yield (Figure 1vi,x). These experiments demonstrated the pivotal role of the amide group in this transformation.

**Figure 1:**
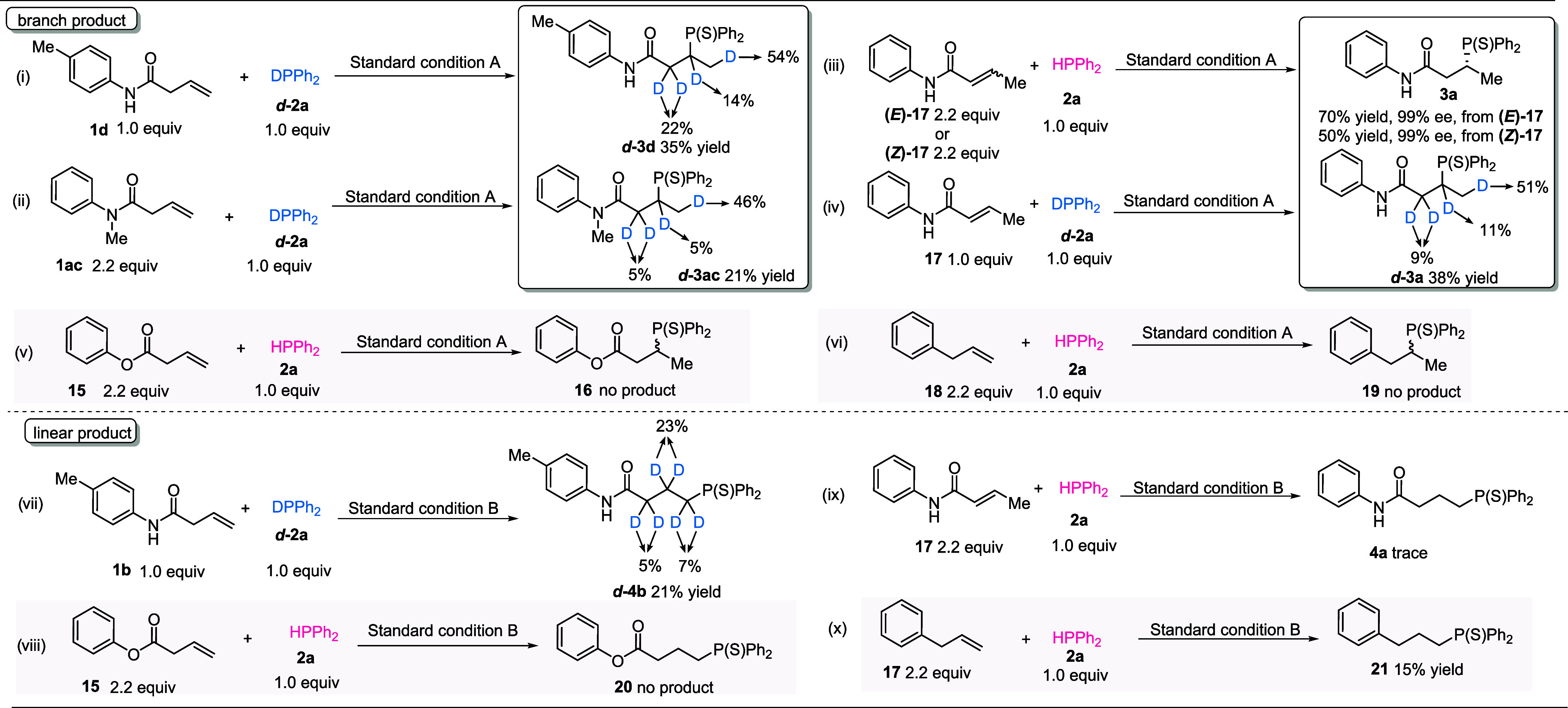
Control experiments. Deuterium-labeling and control experiments were performed. Standard condition A: Ni­(cod)_2_ (5 mol %) and (*R,R*)-Benzp (6 mol %) in toluene/DMF (0.48/0.02 mL) were stirred at rt for 20 min under argon. Then, **A5** was added and stirred for 10 min; **alkene** (x mmol) and **diphenylphosphane** (0.1 mmol) were added, and the reaction mixtures were stirred at 80 °C for 12 h. Then, they were oxidized by S_8_. Standard condition B: Ni­(cod)_2_ (5 mol %) and rac Benzp (6 mol %) in toluene/DMF (0.48/0.02 mL) were stirred at rt for 20 min under argon. Then, **A22** was added and stirred for 10 min; **alkene** (x mmol) and **diphenylphosphane** (0.1 mmol) were added, and the reaction mixtures were stirred at 90 °C for 18 h. Then, they were oxidized by S_8_.

We further performed density functional theory (DFT) studies (SI Section 8) to fully understand the catalytic cycle. The Gibbs energy profiles of the regiodivergent hydrophosphination are shown in Figure 2. Olefin substrate **1a** may coordinate to (*R*,*R*)-BenzP-ligated nickel to give two conformers, **INT1** and **INT1′**, with **INT1′** lying 4.5 [4.0] kcal/mol above **INT1** (Figure S1). In the presence of pyridine-3-sulfonic acid (Figure 2A), an inner-sphere protonation of the olefin C=C bond, assisted by the Ni–O­(sulfone) interaction, can occur at either the terminal or internal carbon in **INT1** and **INT1′** (this gives rise to four possibilities, Figure S2), with the protonation at the terminal carbon in **INT1** having the lowest barrier of 9.5 [11.0] kcal/mol (**TS1A_Cterm**, Figure S3) due to its lower distortion energy (Table S8). After protonation, the pyridine-3-sulfonate anion coordinates to the Ni center to give **INT4A**, at −7.7 [−6.6] kcal/mol. The pyridine-3-sulfonate anion can be exchanged by a diphenylphosphide anion to yield a more stable intermediate, **INT5A**, at −29.2 [−26.2] kcal/mol. This species can then undergo reductive elimination, via **TS2A** (Figure S4), with a barrier of 15.3 [15.2] kcal/mol, to give the hydrophoshpination product coordinated to the Ni complex, **INT6A**, at −43.1 [−40.0] kcal/mol. This process is thermodynamically downhill, with a Gibbs energy of reaction from **INT5A** to **INT6A** of −13.9 [−13.8] kcal/mol. Finally, the displacement of the phosphination product by olefin substrate **1a** regenerates **INT1A** and continues the catalytic cycle. This step is again thermodynamically downhill and favorable, with a Gibbs energy of reaction from **INT6A** to **INT1A** of −8.6 [−8.3] kcal/mol (Figure 2A). The more favorable barrier height of **TS1A_Cterm**, by 2.1 [2.0] kcal/mol (ΔΔ*G* ^‡^), than **TS1A′_Cin** leads to the formation of the Markovnikov product, by an estimated rr value of about 17–20:1.

**Figure 2:**
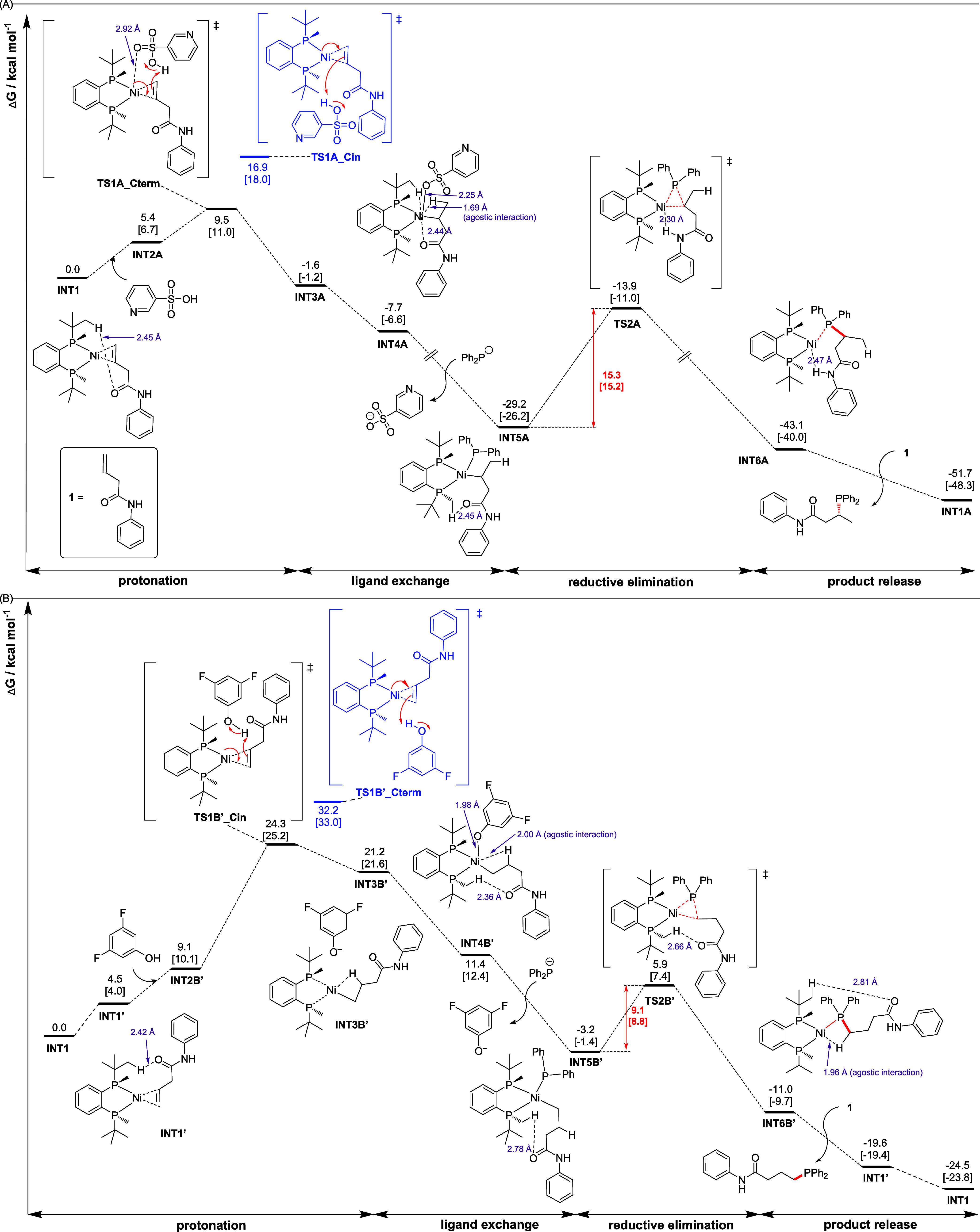
Computed Gibbs energy profiles. DFT calculations of the regiodivergent hydrophosphination effected by different Brønsted acids. Gibbs energies are given in kcal/mol at the C-PCM­(toluene-DMF)­[SMD­(toluene)]-MN15/def2-TZVP//MN15/def2-SVP levels of theory. Ni-catalyzed hydrophosphination reactions using (A) pyridine-3-sulfonic acid and (B) 3,5-difluorophenol.

In the presence of 3,5-difluorophenol, a similar catalytic cycle occurs following the process of protonation, ligand exchange, reductive elimination, and product release (Figure 2B). However, the most favorable protonation of internal olefin by 3,5-difluorophenol occurs on **INT1′**, via **TS1B′_Cin**, which has the lowest barrier, at 24.3 [25.2] kcal/mol (Figure S5), due to its better stabilization interactions (Figure S6 and Table S9). On the other hand, the protonation of terminal olefin has a barrier of 30.1 [30.6] kcal/mol, via **TS1B_Cterm**. This barrier difference of 5.8 [5.4] kcal/mol (ΔΔ*G* ^‡^) translates to an rr value of about 2200–3900:1, indicating that protonation by 3,5-difluorophenol predominantly occurs on the terminal carbon of the C=C bond of the substrate to yield the anti-Markovnikov product. We note that in contrast to the Markovnikov product formation enabled by using pyridine-3-sulfonic acid, the protonation step here occurs via an outer-sphere mechanism, as the additive 3,5-difluorophenol does not form a coordination interaction with the Ni center.

After protonation, the 3,5-difluorophenoxide anion coordinates to the Ni center to give **INT4B′**, at 11.4 [12.4] kcal/mol. The 3,5-difluorophenoxide anion can be exchanged by the diphenylphosphide anion to yield a more stable intermediate, **INT5B′**, at −3.2 [−1.4] kcal/mol. **INT5B′** can undergo reductive elimination, via **TS2B′** (Figure S7), with a barrier of 9.1 [8.8] kcal/mol, to give the hydrophoshpination product coordinated to the Ni complex, **INT6B′**, at −19.6 [−19.4] kcal/mol. This process is thermodynamically downhill, with a Gibbs energy of reaction, from **INT5B′** to **INT6B′** of −7.8 [−8.3] kcal/mol. Finally, the displacement of the phosphination product by olefin substrate **1** regenerates **INT1′** and continues the catalytic cycle. This step is again thermodynamically downhill and favorable, with the Gibbs energy of reaction from **INT6B′** to **INT1′** of −8.6 [−9.7] kcal/mol.

Comparing the additive-controlled reactivities (Figure 2A,B), we observe that the alternative Brønsted acids, through varied structural features that modulate the interactions with the catalyst–substrate complex and the different modes of action via inner- versus outer-sphere protonation, dictate the regiodivergent product selectivity outcomes.

With the assistance of the above control experiments and DFT calculations, a plausible mechanism for the regiodivergent and enantioselective hydrophosphination of unactivated alkenes was proposed (Figure 3). Initially, with the assistance of **A5** or **A22**, the inner/outer-sphere hydronickelation of alkene occurs and gives the corresponding intermediates **II** and **IV**. The intermediates **III** and **V** formed through the ligand exchange with HPAr_2_ undergo the following reductive elimination, delivering anti-Markovnikov/Markovnikov products, organophosphorus compounds **3** and **4**, respectively.

**Figure 3:**
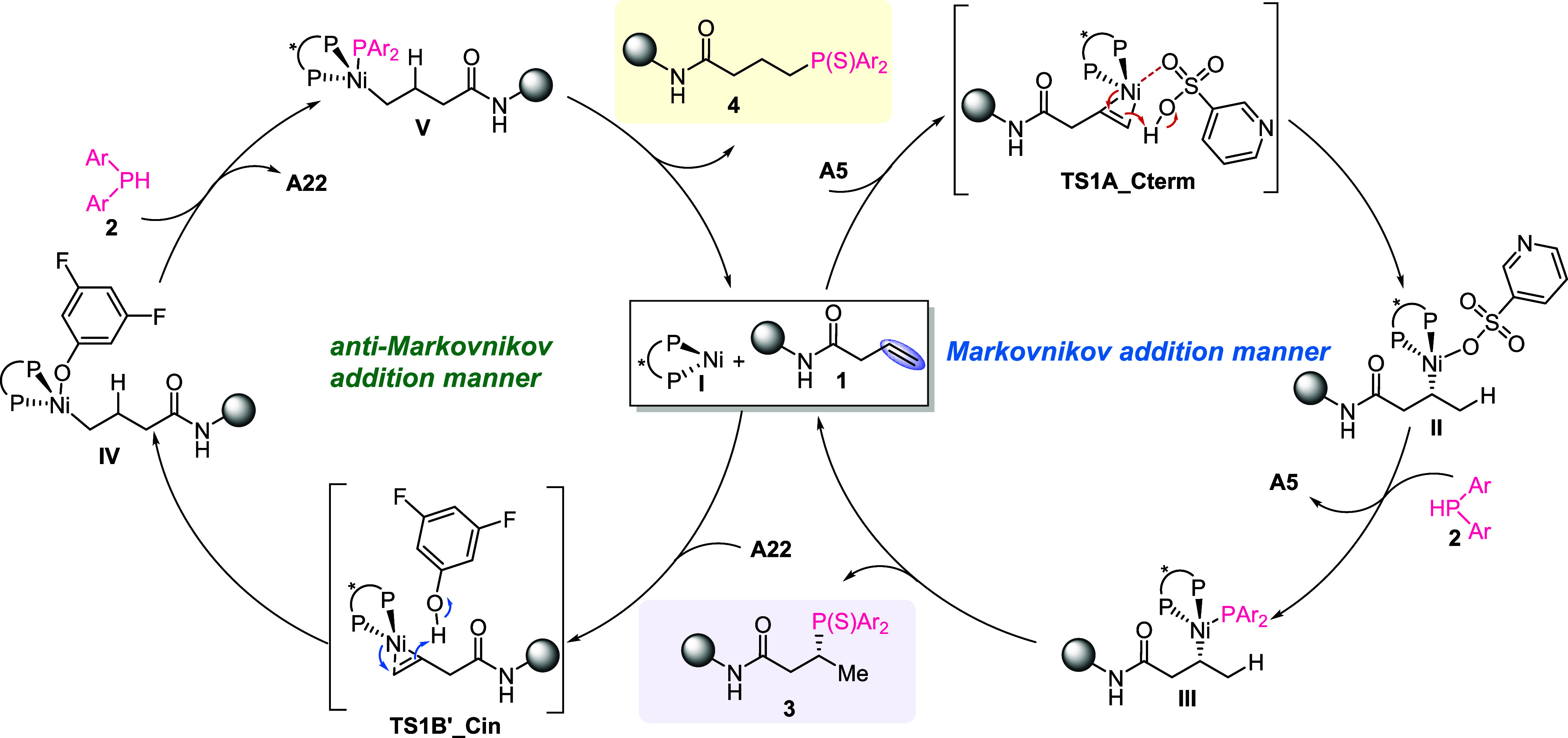
Reaction mechanism of Ni- and Brønsted acid-cocatalyzed regiodivergent hydrophosphination of unactivated alkenes.

## Conclusion

In this work, our reported approach enables the amide-directing, acid-additive-controlled regiodivergent Ni-catalyzed asymmetric hydrophosphination reaction of unactivated alkenes. Facilitated by cooperative weak noncovalent interactions between the amide group and the ligand, varying the Brønsted acid additives enables inner-sphere vs outer-sphere protonation in the formal hydronickelation of the C=C bond, thus affording regiodivergent Markovnikov and anti-Markovnikov products with good yields and excellent enantiomeric excess in previously elusive transformation. We believe that these findings provide a distinctive angle into and open up new avenues for the further design of the catalytic system in diverse hydrofunctionalization reactions and other related reactions of double bonds.

## Supplementary Material



## Data Availability

Geometries of all DFT-optimized structures (in the *.xyz* format with their associated gas-phase energies in Hartrees) have been uploaded to https://zenodo.org/records/16432375 (DOI: 10.5281/zenodo.16432375).

## Notes

Screening of Pd catalysts; screening of Ni catalysts; screening of solvents; distortion–interaction analysis for the protonation step using 3,5-difluorophenol; and distortion–interaction analysis for the protonation step using pyridine-3-sulfonic acid (PDF)
