# Supplementary material for: Additive-Controlled Regioswitching in Ni-Catalyzed Enantioselective Hydrophosphination of Unactivated Alkenes
Source: J Am Chem Soc. 2026 Jan 13;148(3):3481–90. doi: 10.1021/jacs.5c19022 (PMC12856893; doi:10.1021/jacs.5c19022)
Supplement: Supplementary file 1 [file ja5c19022_si_001.pdf]

Additive-controlled Regio-switching in Ni-catalyzed Enantioselective Hydrophosphination of Unactivated Alkenes

Jian Zhou[1], Sichen Tao[1], Xinglong Zhang[2]*, Jun (Joelle) Wang[1]*

[1]Department of Chemistry, Hong Kong Baptist University, Kowloon, Hong Kong, China

[2]Department of Chemistry, The Chinese University of Hong Kong, Shatin, New Territories, Hong Kong, China

Corresponding author. Email: xinglong.zhang@cuhk.edu.hk; junwang@hkbu.edu.hk

Content

1. Materials and Methods 2
2. Optimization of the reaction conditions 2
3. Typical procedure for nickel-catalyzed asymmetric Markovnikov hydrophosphination 7
4. Typical procedure for nickel-catalyzed *anti*-Markovnikov hydrophosphination ..8
5. Scale-up experiments 9
6. Diversification of target compounds 12
7. Mechanistic studies 15
8. Computational Studies 23
   8.1 Computational Methods 23
   8.2 Model reactions 24
   8.3 Conformational Considerations 24
   8.4 Reactivity with pyridine-3-sulfonic acid 26
   8.5 Reactivity with 3,5-difluorophenol 36
   8.6 Estimation of product ratio under kinetic control 46
   8.7 Optimized structures and absolute energies 47
9. Analytic data for the products 50
10. NMR Spectrum of target compounds 100
11. HPLC Spectrum of target compounds 231
12. X-ray crystal structure of **3j** 271
13. Rerference 272

1. Materials and Methods

General information

NMR Spectra were recorded on a Bruker DPX-500 (400) spectrometer at 600 MHz or 400 MHz for ^{1}H NMR, 376 MHz for ^{19}F NMR and 101 MHz or 125 MHz for ^{13}C NMR in $CDCl_3$ with tetramethylsilane (TMS) or the residual deuterated solvent peaks as internal standard. Chemical shifts (δ) are reported in ppm, and coupling constants (J) are in Hertz (Hz). Flash column chromatograph was carried out using 200-300 mesh silica gel at medium pressure. High resolution mass spectra (HRMS) were recorded on a LC-TOF spectrometer. ESI-HRMS data were acquired using a Thermo LTQ Orbitrap XL Instrument equipped with an ESI source. Optical rotation was obtained on a Rudolph Research Analytical (Atopol I). HPLC analysis was performed on Agilent 1260 series. Unless otherwise noted, all reagents were purchased from commercial suppliers and used without purification. All air- and moisture-sensitive manipulations were carried out with standard Schlenk techniques under nitrogen or in a glove box under argon. Anhydrous THF (Tetrahydrofuran), toluene was distilled from sodium benzophenone prior to use. Alkenes **1** and Secondary phosphine oxides are known compounds and were synthesized according to the literature procedure[1-8].

2. Optimization of the reaction conditions

Table S1. Screening of Pd catalysts[a]

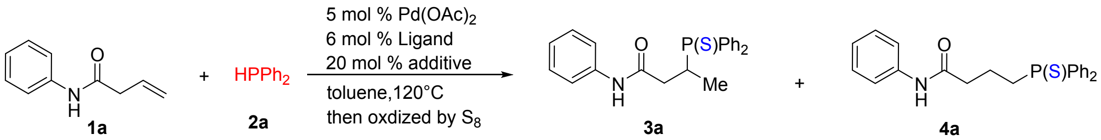

Entry	ligand	additives	yield for **3a**/**4a** (%)	ee for **3a** (%)
1	(*R*,*R*)-BenzP	/	30/12	7
2[b]	(*R*,*R*)-BenzP	$Ph_2P(O)OH$	25/6	3
3[b]	(*R*,*R*)-QunioxP	$Ph_2P(O)OH$	32/0	3
4	(*R*)-(*S*)-PPF-$P(tBu)_2$	/	40/12	0
5	(R)-(S)-cy_2PF-$PtBu_2$	/	38/6	0

[a]Reaction conditions: $Pd(OAc)_2$ (5 mol %) and Ligand (6 mol %) in toluene (0.5 mL) were stirred at r.t. for 20 min under argon, then additive (20 mol %) was added and stirred for 10 min. **1** (0.12 mmol) and **2** (0.1 mmol) were added, and the reaction mixtures were stirred at 120 °C for 12h. Then oxidized by S_8. Isolated yield, the ratio of **3a**/**4a** were determine by ^{31}P spectra of crude. [b]reacted at 100°C.

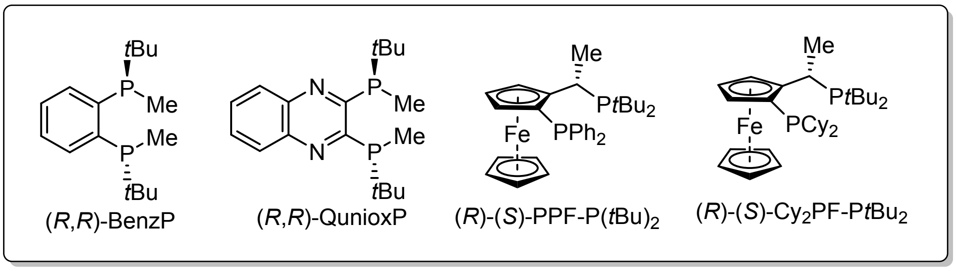

Table S2. Screening of Ni catalysts[a]

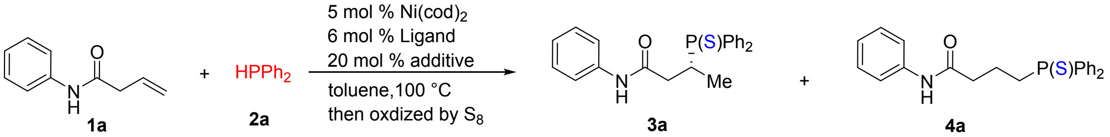

Entry	ligand	additives	yield for **3a**/**4a** (%)	ee for **3a** (%)
1	BINAP	/	10/0	/
2	(*R*,*R*)-QunioxP	/	10/0	9
3	(*R*,*R*)-Ph-BPE	/	trace	/
4[b]	(*R*,*R*)-Ph-BPE	/	27/0	0
5	(*R*,*R*)-Ph-BPE	*p*-TsOH	12/0	50
6	(*R*,*R*)-BenzP	/	trace	/
7[b]	(*R*,*R*)-BenzP	/	22/0	20
8	(*R*,*R*)-BenzP	*p*-TsOH	33/0	96
9	(*Rc*,*Sp*)-DuanPhos	*p*-TsOH	40/0	0
10	(*R*,*R*)-*i*Pr-DuPhos	*p*-TsOH	36/0	80
11	(*R*,*R*)-BDPP	*p*-TsOH	10/0	3
12	(*R*,*R*)-QunioxP	*p*-TsOH	50/0	65
13[c]	(*R*,*R*)-BenzP	*p*-TsOH	30/0	77

[a]Reaction conditions: $Ni(cod)_2$ (5 mol %) and Ligand (6 mol %) in toluene (0.5 mL) were stirred at r.t. for 20 min under argon, then additive (20 mol %) was added and stirred for 10 min. **1** (0.12 mmol) and **2** (0.1 mmol) were added, and the reaction mixtures were stirred at 100 °C for 12h. Then oxidized by S_8. Isolated yield, the ratio of **3a**/**4a** were determine by ^{31}P spectra of crude. [b]reacted at 120 °C. [c]$Pd(OAC)_2$ was used.

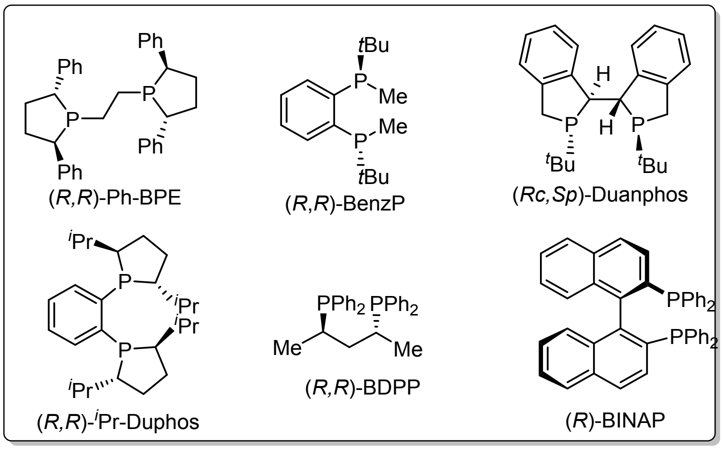

Table S3. Screening of reaction temperature[a]

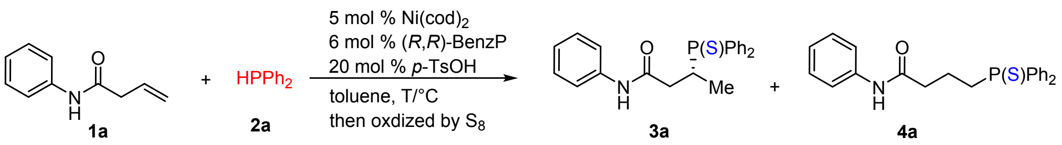

Entry	T/°C	yield for **3a**/**4a** (%)	ee for **3a** (%)
1	90	35/0	97
2 [b]	90	35/2	89
3	70	24/2	93
4	60	trace	/
5	40	trace	/
6	rt	trace	/

[a]Reaction conditions: $Ni(cod)_2$ (5 mol %) and Ligand (6 mol %) in toluene (0.5 mL) were stirred at r.t. for 20 min under argon, then *p*-TsOH (20 mol %) was added and stirred for 10 min. **1** (0.12 mmol) and **2** (0.1 mmol) were added, and the reaction mixtures were stirred at indicated temperature for 12h. Then oxidized by S_8. Isolated yield, the ratio of **3a**/**4a** were determine by ^{31}P spectra of crude. [b]10 mol % *p*-TsOH was used.

Table S4. Screening of Brønsted acids[a]

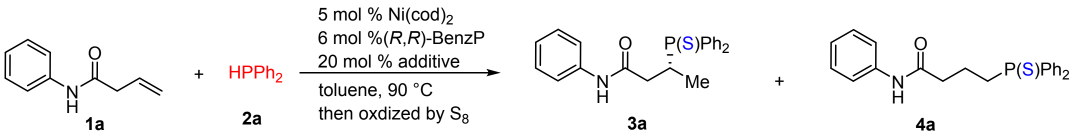

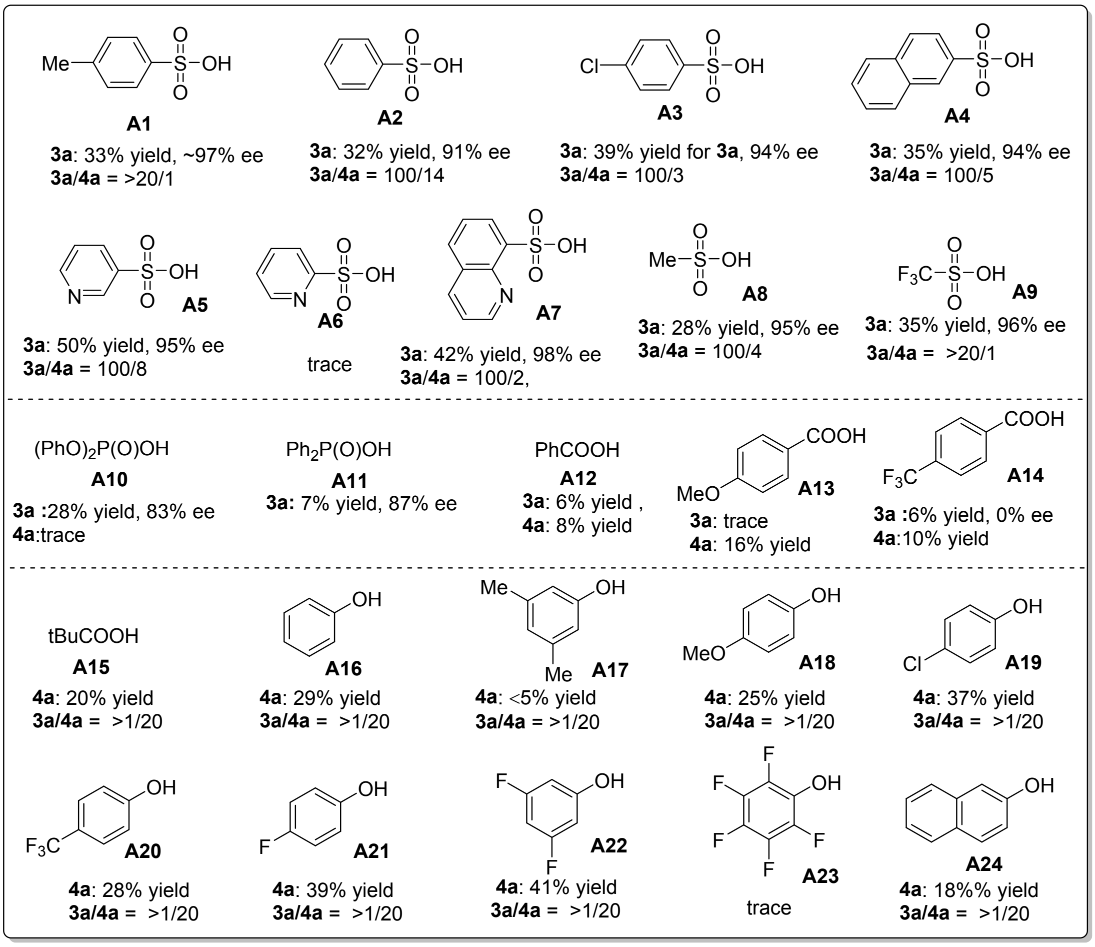

[a]Reaction conditions: $Ni(cod)_2$ (5 mol %) and Ligand (6 mol %) in toluene (0.5 mL) were stirred at r.t. for 20 min under argon, then additives (20 mol %) was added and stirred for 10 min. **1** (0.12 mmol) and **2** (0.1 mmol) were added, and the reaction mixtures were stirred at 90 °C for 12h. Then oxidized by S_8. Isolated yield, the ratio of **3a**/**4a** were determine by ^{31}P spectra of crude.

Table S5. Screening of Ni precursors[a]

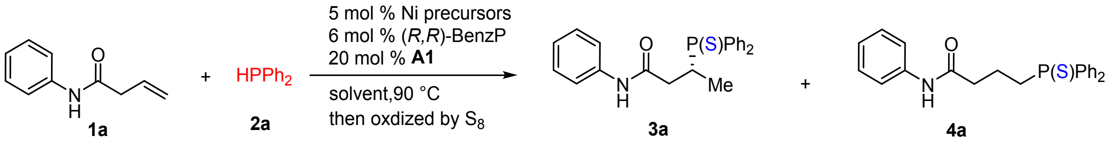

Entry	solvent	yield for **3a**/**4a** (%)	ee (%)
1	$NiCl_2$•dme	trace	/
2	$NiCl_2$	8	47
3	$NiCl_2$(dppe)	trace	/
4	$Ni(OTf)_2$	trace	/
5	$Ni(acac)_2$	7	3
6	NiI_2	9	15
7	$NiBr_2$•dme	trace	/
8	$Ni(OAc)_2$•$4H_2O$	17/0	92

9	$Ni(ClO_4)_2•6H_2O$	trace	/
10	$Ni(BF_4)_2•6H_2O$	11/0	90
11	$Ni(PPh_3)_4$	9/0	83
12	$Ni(tBuSTB)_4$	27/0	98
13	$Ni[P(OC_6H_5)_3]_4$	15/0	92
14	$Ni(4\text{-}CF_3STB)_4$	7/0	85
15	Ni_2B	trace	/
16	$Ni(cod)_2$	35	97

[a]Reaction conditions: Ni precursor (5 mol %) and Ligand (6 mol %) in toluene (0.5 mL) were stirred at r.t. for 20 min under argon, then *p*-TsOH (20 mol %) was added and stirred for 10 min. **1** (0.12 mmol) and **2** (0.1 mmol) were added, and the reaction mixtures were stirred at 90 °C for 12h. Then oxidized by S_8. Isolated yield, the ratio of **3a**/**4a** were determine by ^{31}P spectra of crude.

Table S6. Screening of solvents[a]

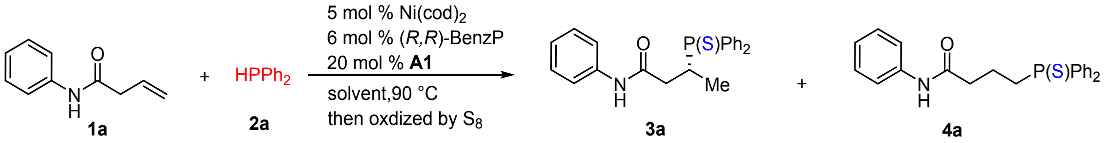

Entry	solvent	yield for **3a/4a** (%)	ee (%)
1	PhCl	8/0	4
2	Anisole	trace	/
3	$PhCF_3$	trace	/
4	DCE	15/0	0
5	THF	31/0	87
6	1,4-dioxane	32/0	90
7	MeCN	28/0	55
8	CH_3Cl	trace	/
9	EA	29/1	90
10	DMF	70/1	89
11	DMSO	trace	/

[a]Reaction conditions: $Ni(cod)_2$ (5 mol %) and Ligand (6 mol %) in solvent (0.5 mL) were stirred at r.t. for 20 min under argon, then *p*-TsOH (20 mol %) was added and stirred for 10 min. **1** (0.12 mmol) and **2** (0.1 mmol) were added, and the reaction mixtures were stirred at 90 °C for 12h. Then oxidized by S_8. Isolated yield, the ratio of **3a**/**4a** were determine by ^{31}P spectra of crude.

Table S7. Screening of mixed solvents[a]

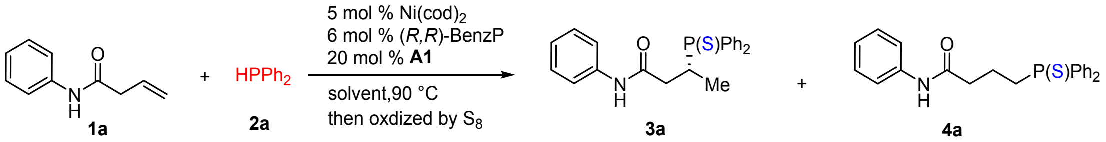

Entry	solvent (tol/DMF)	yield for **3a/4a** (%)	ee (%)
1	0.5 mL/0 mL	42/0	98
2	0.25 mL/0.25 mL	56/0	85

3	0.4 mL/0.1 mL	58/0	93
4	0.1 mL/0.4 mL	60/0	83
5	0.48 mL/0.02 mL	57/0	97
6[b]	0.48 mL/0.02 mL	70/0	98
7 [b,c]	0.48 mL/0.02 mL	73/0	99
8[d,e]	0.48 mL/0.02 mL	0/90	/

[a]Reaction conditions: Ni(cod)$_2$ (5 mol %) and Ligand (6 mol %) in solvent (0.5 mL) were stirred at r.t. for 20 min under argon, then *p*-TsOH (20 mol %) was added and stirred for 10 min. **1** (0.22 mmol) and **2** (0.1 mmol) were added, and the reaction mixtures were stirred at 90 °C for 12h. Then oxidized by S_8. [b]**A5** was used. [c]reacted at 80 °C. [d]**A22** was used. [e]reacted for 18h. Isolated yield, the ratio of **3a**/**4a** were determine by ^{31}P spectra of crude.

3. Typical procedure for nickel-catalyzed asymmetric Markovnikov hydrophosphination

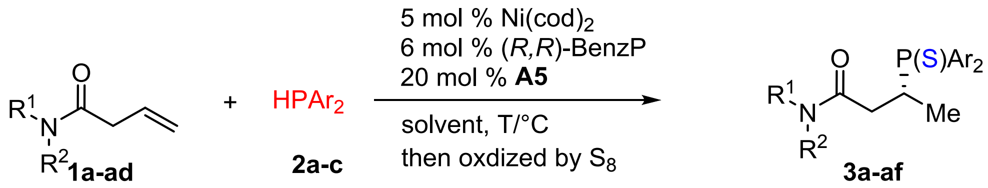

General procedure A: An oven-dried Schlenk tube with a stirred bar was charged with 5 mol % Ni(cod)$_2$ (1.5 mg, 0.0050 mmol), 6 mol % (*R*,*R*)-BenzP (1.8 mg, 0.0060 mmol) and 0.48 mL toluene and 0.02 mL DMF in argon atmosphere. The resulting solution was stirred at indicated reaction temperature for 20 min, then **A5** (3.2 mg, 0.0200 mmol) was allowed to add and stirred for additional 10 min, then 0.22 mmol alkene **1** and 0.1 mmol diarylphosphane **2** were added, the mixture was stirred at indicated reaction temperature under argon atmosphere for 12 h. Then 0.2 mmol S_8 was added and stirred at room temperature for 3 h. The reaction mixture purified by column chromatography on silica gel to get the corresponding product **3.** The ee values was determined by HPLC.

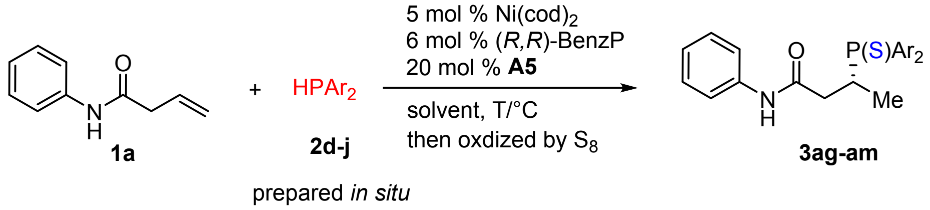

General procedure B: To oven-dried Schlenk tube equipped with a stirrer bar, 0.1 mmol diarylphosphine oxdies **2d-j** and 0.1 mmol $PhSiH_3$ or DEMS were dissolved in 200 μL tol and stirred at 70℃ for 48 h. Next, prepared the catalysts by dissolve 5 mol % $Ni(cod)_2$ (1.5 mg, 0.0050 mmol), 6 mol % (*R*,*R*)-BenzP (1.8 mg, 0.0060 mmol) with 0.2 mL toluene and 0.02 mL DMF in argon atmosphere and stirred at room temperature for 20 min, then followed by the addition of **A5** (3.2 mg, 0.0200 mmol) and stirred for additional 10 min. Subsequently, the catalyst mixture and 0.22 mmol alkene **1** were added into diarylphosphane prepared *in situ*, the mixture was stirred at indicated reaction temperature under argon atmosphere for 36 h. Then 0.2 mmol S_8 was added and stirred at room temperature for 3 h. The reaction mixture purified by column chromatography on silica gel to get the corresponding product **3.** The ee values was determined by HPLC.

4. Typical procedure for nickel-catalyzed *anti*-Markovnikov hydrophosphination

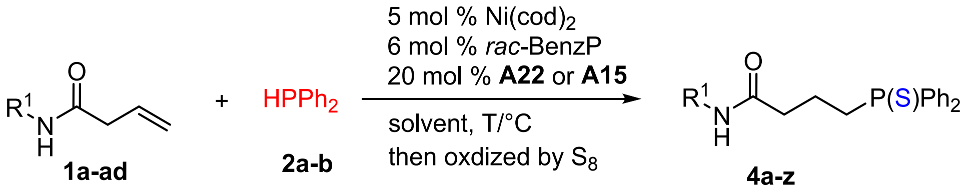

General procedure C: An oven-dried Schlenk tube with a stirred bar was charged with 5 mol % $Ni(cod)_2$ (1.5 mg, 0.0050 mmol), 6 mol % rac-BenzP (1.8 mg, 0.0060 mmol) and 0.48 mL toluene and 0.02 mL DMF in argon atmosphere. The resulting solution was stirred at room temperature for 20 min, then **A22** (2.6 mg, 0.0200 mmol) or **A15** (2.1 mg, 0.0200 mmol) was allowed to add and stirred for additional 10 min, then 0.22 mmol alkene **1** and 0.1 mmol diarylphosphane **2** were added, the mixture was stirred at indicated reaction temperature under argon atmosphere for 12 h. Then 0.2 mmol S_8 was added and stirred at room temperature for 3 h. The reaction mixture purified by column chromatography on silica gel to get the corresponding product **4**.

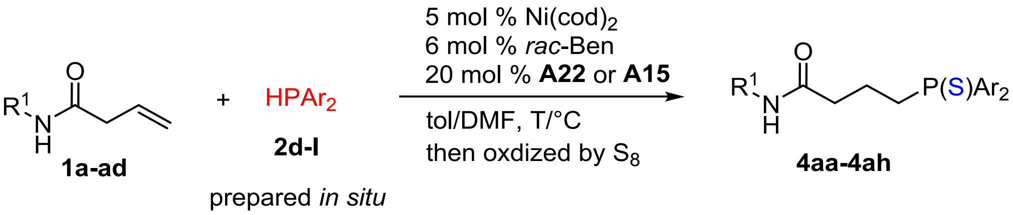

General procedure D: To oven-dried Schlenk tube equipped with a stirrer bar, 0.1 mmol diarylphosphine oxdies **2d-j** and 0.1 mmol $PhSiH_3$ or DEMS were dissolved in 200 μL tol and stirred at 70°C for 48 h. Next, prepared the catalysts by dissolve 5 mol % $Ni(cod)_2$ (1.5 mg, 0.0050 mmol), 6 mol % (*R*,*R*)-BenzP (1.8 mg, 0.0060 mmol) with 0.2 mL toluene and 0.02 mL DMF in argon atmosphere and stirred at room temperature for 20 min, then followed by the addition of **A22** (2.6 mg, 0.0200 mmol) or **A15** (2.1 mg, 0.0200 mmol) and stirred for additional 10 min. Subsequently, the catalyst mixture and 0.22 mmol alkene **1** were added into diarylphosphane, the mixture was stirred at indicated reaction temperature under argon atmosphere for 36 h. Then 0.2 mmol S_8 was added and stirred at room temperature for 3 h. The reaction mixture purified by column chromatography on silica gel to get the corresponding product **4**.

5. Scale-up experiments

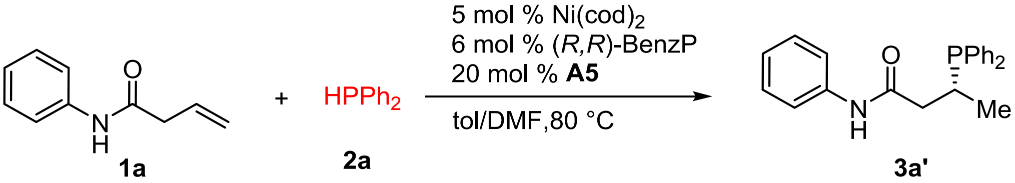

An oven-dried Schlenk tube with a stirred bar was charged with 5 mol % $Ni(cod)_2$ (45.0 mg, 0.1500 mmol), 6 mol % (*R*,*R*)-BenzP (48 mg, 0. 1800 mmol) and 14.4 mL toluene and 6.0 mL DMF in argon atmosphere. The resulting solution was stirred at room temperature for 20 min, then **A5** (96 mg, 0.6000 mmol) was allowed to add and stirred for additional 10 min, then 6.6 mmol alkene **1** and 3.0 mmol $HPPh_2$ **2a** were added, the mixture was stirred at 80 °C under argon atmosphere for 18 h. The reaction mixture purified by column chromatography on silica gel to get the corresponding product **3a'** (521.0 mg, 50% yield, 98% ee). The ee values was determined by HPLC. ^1H NMR (400 MHz, Chloroform-*d*) δ 7.60-7.50 (m, 4H), 7.50-7.45 (m, 2H), 7.39-7.27

(m, 8H), 7.19 (s, 1H), 7.13-7.05 (m, 1H), 3.11-3.00 (m, 1H), 2.63-2.51 (m, 1H), 2.17-2.06 (m, 1H), 1.14 (dd, *J* = 14.9, 6.8 Hz, 3H). ^{31}P NMR (162 MHz, Chloroform-*d*) δ -1.47. ^{13}C NMR (101 MHz, Chloroform-*d*) δ 170.07 (d, *J* = 14.2 Hz), 137.86, 136.51-135.63 (m), 133.72 (dd, *J* = 19.2, 5.1 Hz), 129.24 (d, *J* = 9.7 Hz), 129.13, 128.69 (dd, *J* = 11.6, 7.1 Hz), 124.46, 119.86, 41.65 (d, *J* = 17.9 Hz), 27.34 (d, *J* = 9.2 Hz), 16.95 (d, *J* = 16.7 Hz).

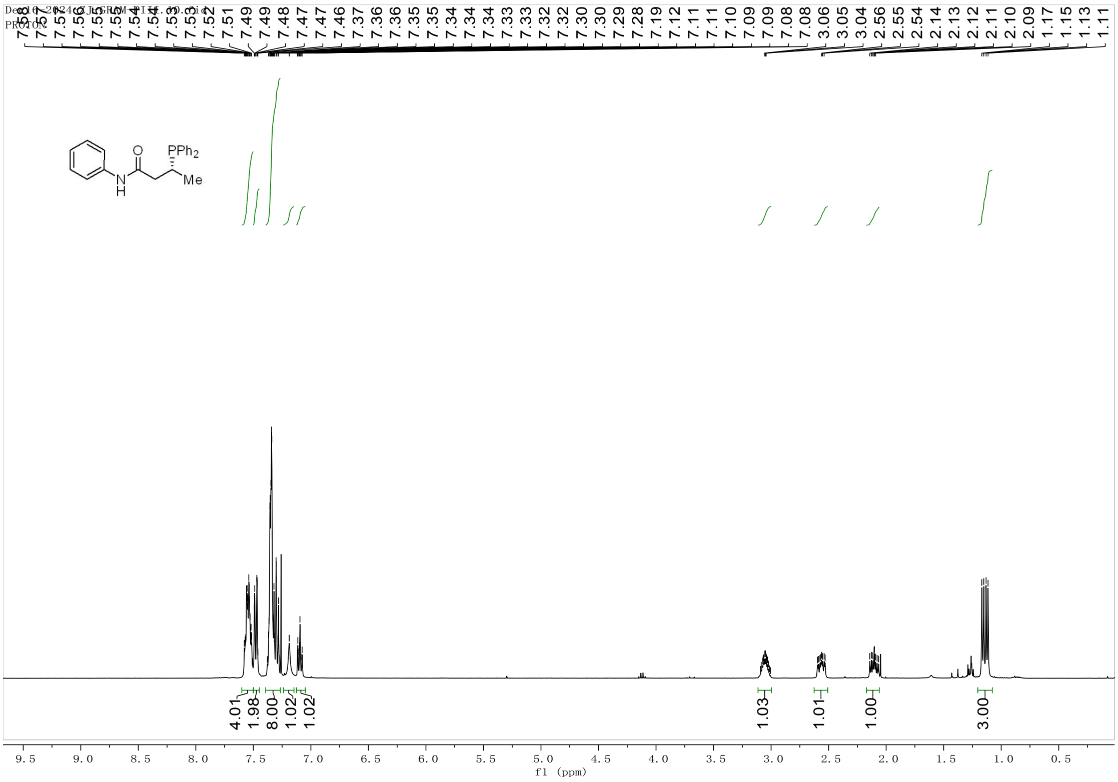

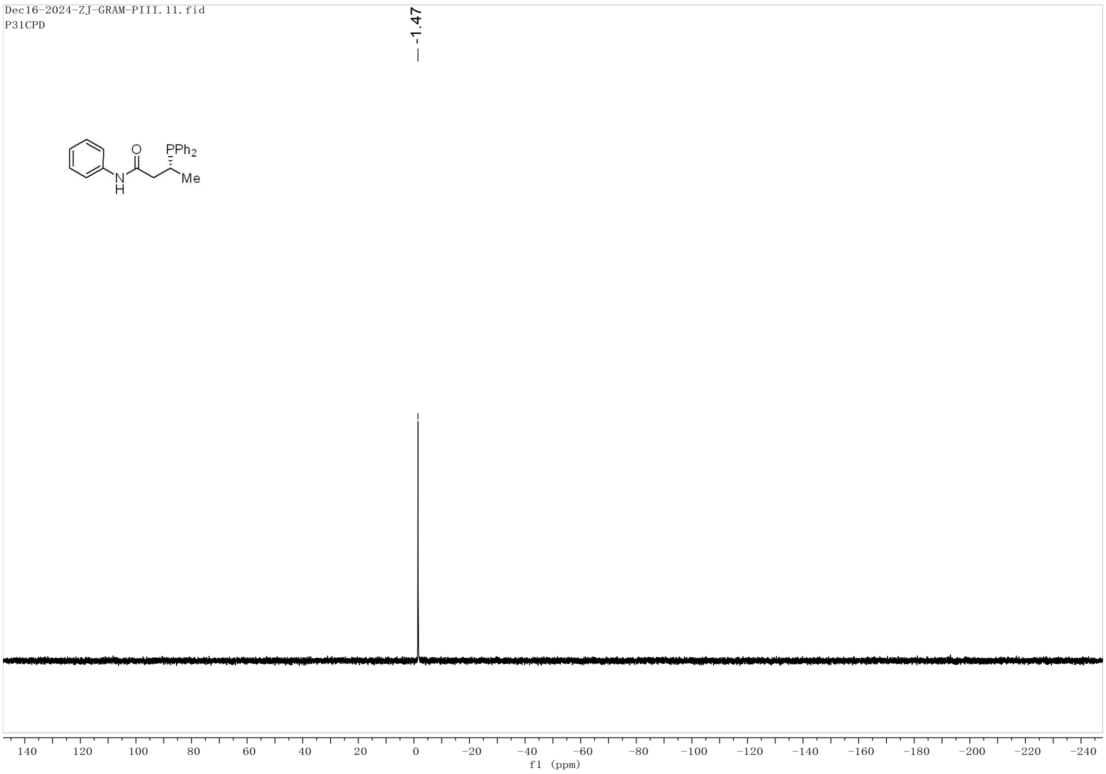

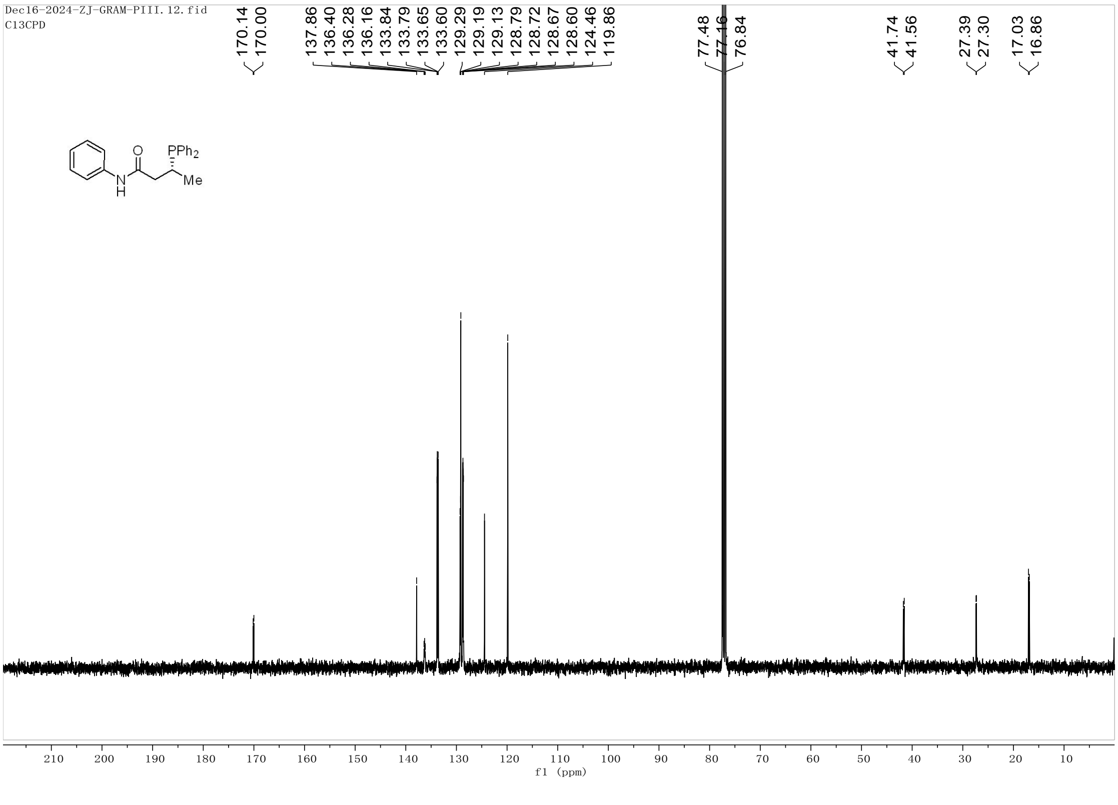

Scale-up synthesis of 4a

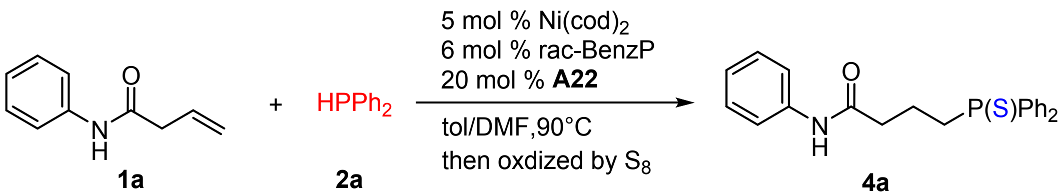

An oven-dried Schlenk tube with a stirred bar was charged with 5 mol % $Ni(cod)_2$ (15 mg, 0.0500 mmol), 6 mol % rac-BenzP (18 mg, 0.0600 mmol) and 4.8 mL toluene and 0.2 mL DMF in argon atmosphere. The resulting solution was stirred at room temperature for 20 min, then **A22** (32 mg, 0.2000 mmol) was allowed to add and stirred for additional 10 min, then 2.2 mmol alkene **1** and 1.0 mmol diarylphosphane **2** were added, the mixture was stirred at 90 ℃ under argon atmosphere for 12 h. Then 2 mmol S_8 was added and stirred at room temperature for 3 h. The reaction mixture purified by column chromatography on silica gel to get the corresponding product **4a** (215.0 mg, 62% yield).

6. Diversification of target compounds

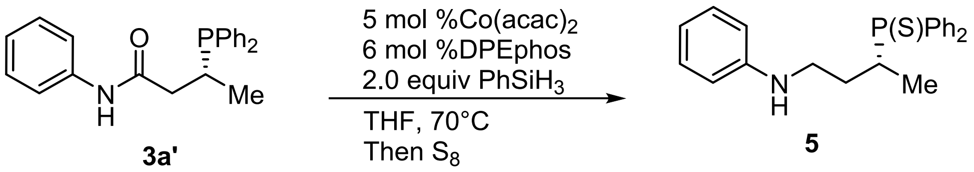

According to literature[9], An oven-dried Schlenk tube with a stirred bar was charged with 5 mol % $Co(acac)_2$ (1.2 mg, 0.0050 mmol), 6 mol % DPEphos (2.6 mg, 0.0060 mmol) and 0.5 mL THF in argon atmosphere. The resulting solution was stirred at room temperature for 20 min, then phenylsilane (21.6 mg, 0.2000 mmol) and 0.1 mmol **3a'** were allowed to add and stirred at 70 ℃ under argon atmosphere for 12 h. Then 0.2 mmol S_8 was added and stirred at room temperature for 3 h. The reaction mixture purified by column chromatography on silica gel to get the corresponding product **5** (20.0 mg, 55% yield, 98% ee)

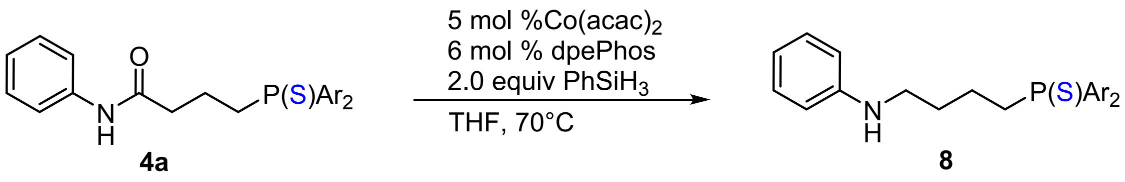

According to literature[9], an oven-dried Schlenk tube with a stirred bar was charged with 5 mol % $Co(acac)_2$ (1.2 mg, 0.0050 mmol), 6 mol % DPEphos (2.6 mg, 0.0060 mmol) and 0.5 mL THF in argon atmosphere. The resulting solution was stirred at room temperature for 20 min, then phenylsilane (21.6 mg, 0.2000 mmol) and 0.1 mmol **4a** were allowed to add and stirred at 70 °C under argon atmosphere for 12 h. The reaction mixture purified by column chromatography on silica gel to get the corresponding product **8** (21.2 mg, 58% yield).

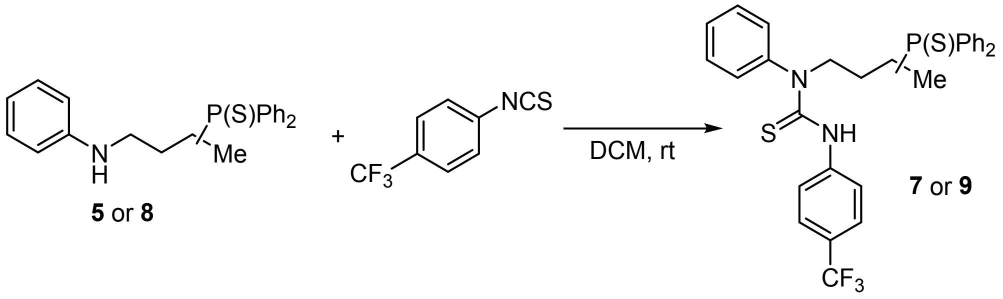

To a 10 mL flask equipped with stir bar and **5** or **8** (0.1 mmol, 1.0 equiv.) 1-isothiocyanato-4-(trifluoromethyl)benzeneanhydrous (20.3 mg, 0.11 mmol 1.0 equiv.) was added. Then 3 mL DCM was added. The mixture was stirred at room temperature for 18 h. The reaction mixture purified by column chromatography on silica gel to get the corresponding product **7** (34.8 mg, 62% yield, 98% ee) and **9** (40.1 mg, 70% yield).

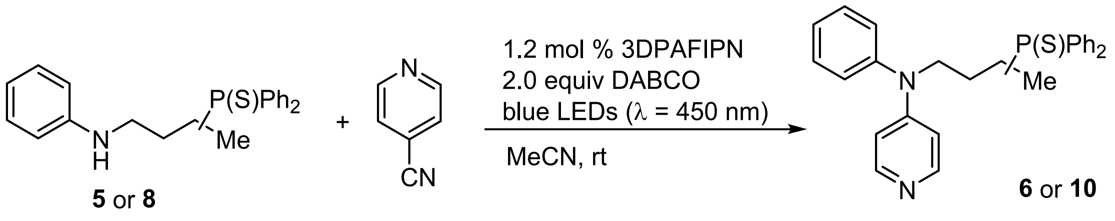

According to literature[10], to a 10 mL Schlenk tube equipped with stir bar was charged with isonicotinonitrile (10.4 mg, 0.1 mmol), DABCO (11.2 mg, 0.1 mmol), PC (3DPAFIPN) (0.8 mg, 0.0012 mmol) and **5** or **8** (0.1 mmol, 1.0 equiv.) in CH_3CN (3 mL). The mixture was irradiated by blue LEDs (λ = 450 nm) for 16 hours at room

temperature. The reaction mixture purified by column chromatography on silica gel to get the corresponding product **6** (37.6 mg, 85% yield, 99% ee) and **10** (30.9 mg, 70% yield).

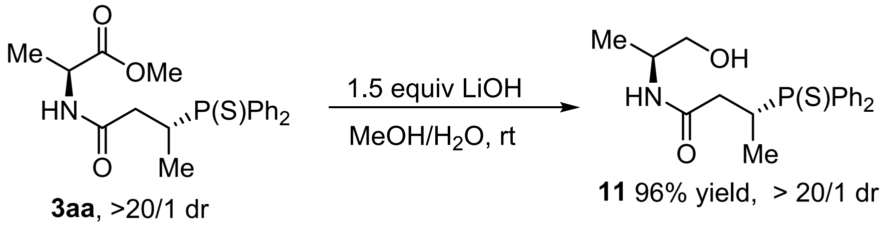

To a 10 mL flask equipped with stir bar and **3aa** (38.9 mg, 0.1 mmol, 1.0 equiv.) lithium hydroxide anhydrous (3 mg, 0.15 mmol 1.6 equiv.) was added. Then MeOH/H_2O (3 mL/0.3 mL) was added. The mixture was stirred at room temperature. After monitored by TLC to see the full conversion of phosphorus-containing amino esters, 0.3 mL 1N HCl aq. was added and extract with EA. The combined organic extract was dried over anhydrous magnesium sulfate, filtered and concentrated under vacuum to give **11** (34.5 mg, 96% yield).

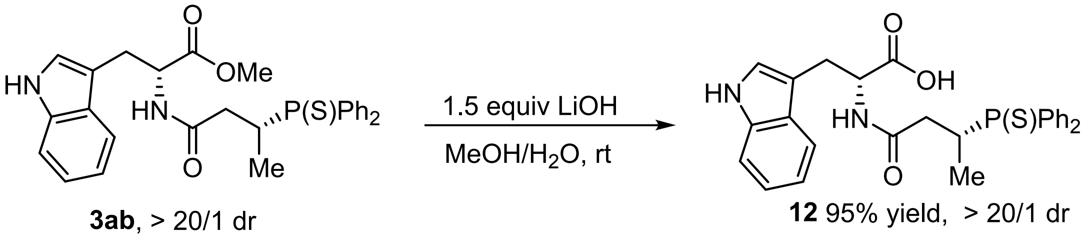

To a 10 mL flask equipped with stir bar and **3ab** (50.4 mg, 0.1 mmol, 1.0 equiv.) lithium hydroxide anhydrous (3 mg, 0.15 mmol 1.6 equiv.) was added. Then MeOH/H_2O (3 mL/0.3 mL) was added. The mixture was stirred at room temperature. After monitored by TLC to see the full conversion of phosphorus-containing amino esters, 0.3 mL 1N HCl aq. was added and extract with EA. The combined organic extract was dried over anhydrous magnesium sulfate, filtered and concentrated under vacuum to give **12** (46.5 mg, 95% yield).

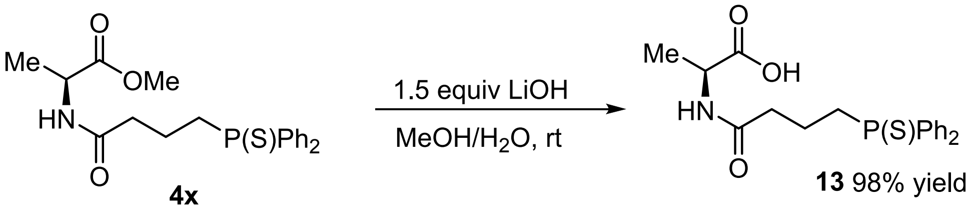

To a 10 mL flask equipped with stir bar and **4x** (38.9 mg, 0.1 mmol, 1.0 equiv.) lithium hydroxide anhydrous (3 mg, 0.15 mmol 1.6 equiv.) was added. Then MeOH/H_2O (3 mL/0.3 mL) was added. The mixture was stirred at room temperature. After monitored by TLC to see the full conversion of phosphorus-containing amino esters, 0.3 mL 1N HCl aq. was added and extract with EA. The combined organic extract was dried over anhydrous magnesium sulfate, filtered and concentrated under vacuum to give **13** (35.2 mg, 95% yield).

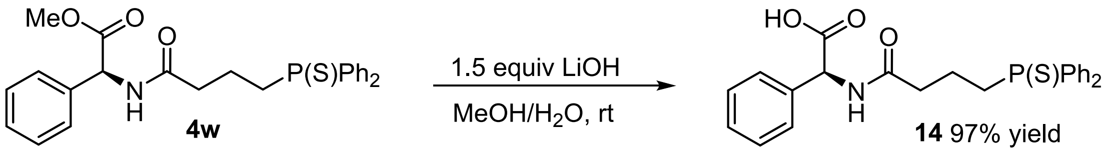

To a 10 mL flask equipped with stir bar and **4w** (45.1 mg, 0.1 mmol, 1.0 equiv.) lithium hydroxide anhydrous (3 mg, 0.15 mmol 1.6 equiv.) was added. Then MeOH/H_2O (3 mL/0.3 mL) was added. The mixture was stirred at room temperature. After monitored by TLC to see the full conversion of phosphorus-containing amino esters, 0.3 mL 1N HCl aq. was added and extract with EA. The combined organic extract was dried over anhydrous magnesium sulfate, filtered and concentrated under vacuum to give **14** (42.4 mg, 97% yield).

7. Mechanistic studies

$DPPh_2$ was prepared according to the previous general procedure[5].

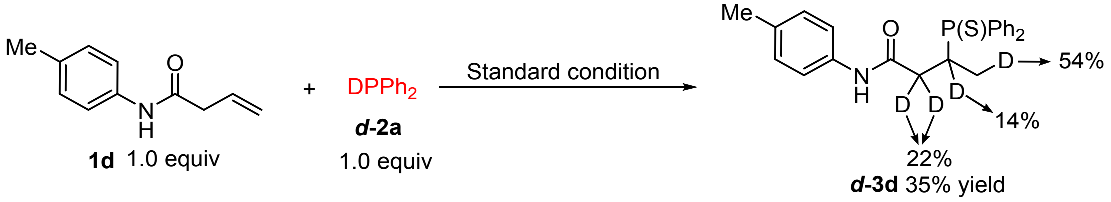

An oven-dried Schlenk tube with a stirred bar was charged with 5 mol % $Ni(cod)_2$ (1.5 mg, 0.0050 mmol), 6 mol % (*R*,*R*)-BenzP (1.8 mg, 0.0060 mmol) and 0.48 mL toluene and 0.02 mL DMF in argon atmosphere. The resulting solution was stirred at room temperature for 20 min, then **A5** (3.2 mg, 0.0200 mmol) was allowed to add and stirred for additional 10 min, then 0.22 mmol alkene **1d** and 0.1 mmol $DPPh_2$ ***d*-2a** were added, the mixture was stirred at 80 °C under argon atmosphere for 12 h. Then 0.2 mmol S_8 was added and stirred at room temperature for 3 h. The reaction mixture purified by

column chromatography on silica gel to get the corresponding product ***d*-3d** (13.1 mg, 35% yield).

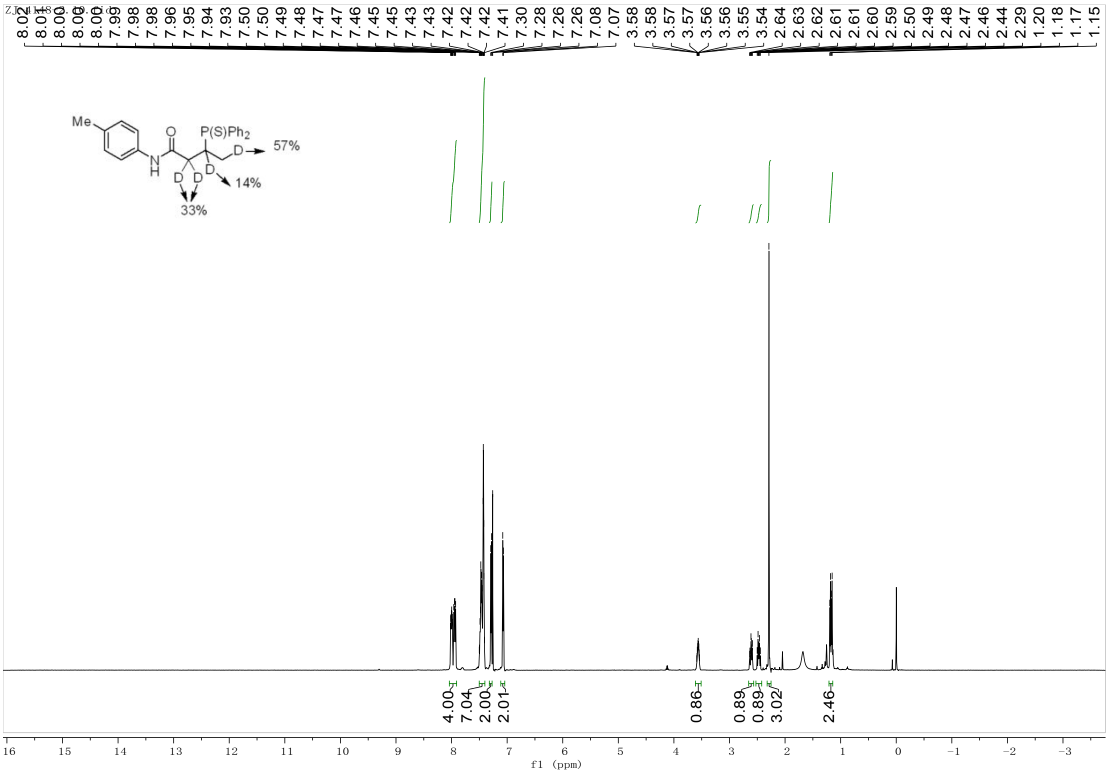

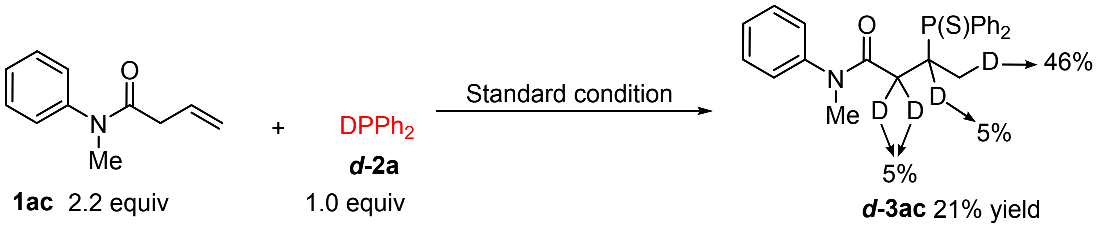

An oven-dried Schlenk tube with a stirred bar was charged with 5 mol % Ni(cod)$_2$ (1.5 mg, 0.0050 mmol), 6 mol % (*R*,*R*)-BenzP (1.8 mg, 0.0060 mmol) and 0.48 mL toluene and 0.02 mL DMF in argon atmosphere. The resulting solution was stirred at room temperature for 20 min, then **A5** (3.2 mg, 0.0200 mmol) was allowed to add and stirred for additional 10 min, then 0.22 mmol alkene **1ac** and 0.1 mmol DPPh$_2$ ***d*-2a** were added, the mixture was stirred at 80 °C under argon atmosphere for 12 h. Then 0.2 mmol S_8 was added and stirred at room temperature for 3 h. The reaction mixture purified by column chromatography on silica gel to get the corresponding product ***d*-3ac** (8.3 mg, 21% yield).

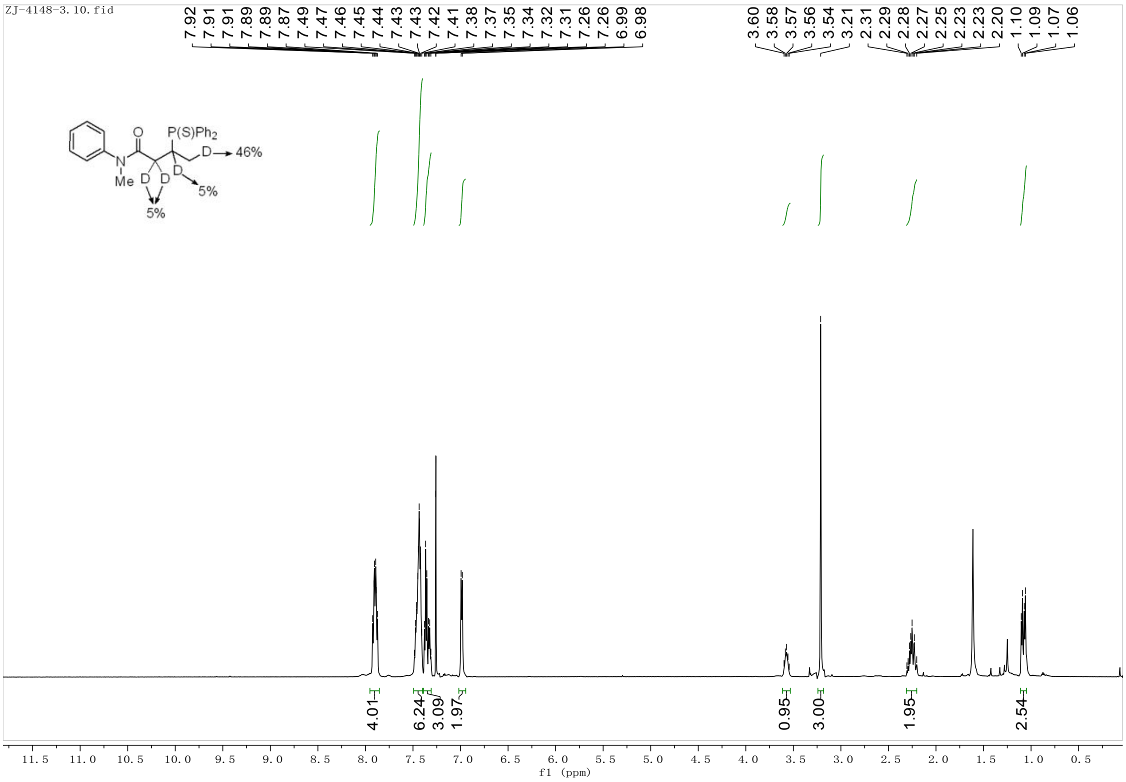

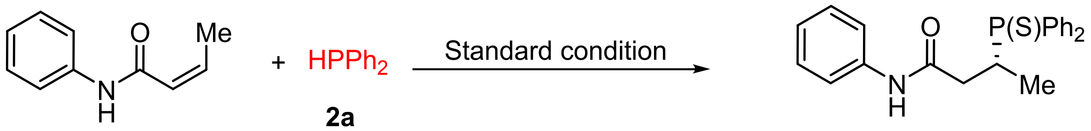

An oven-dried Schlenk tube with a stirred bar was charged with 5 mol % Ni(cod)$_2$ (1.5 mg, 0.0050 mmol), 6 mol % (*R*,*R*)-BenzP (1.8 mg, 0.0060 mmol) and 0.48 mL toluene and 0.02 mL DMF in argon atmosphere. The resulting solution was stirred at room temperature for 20 min, then **A5** (3.2 mg, 0.0200 mmol) was allowed to add and stirred for additional 10 min, then 0.22 mmol **(*Z*)-17** and 0.1 mmol HPPh$_2$ **2a** were added, the mixture was stirred at 80 °C under argon atmosphere for 12 h. Then 0.2 mmol S$_8$ was added and stirred at room temperature for 3 h. The reaction mixture purified by column chromatography on silica gel to get the corresponding product **3a** (18.9 mg, 50% yield, 99% ee).

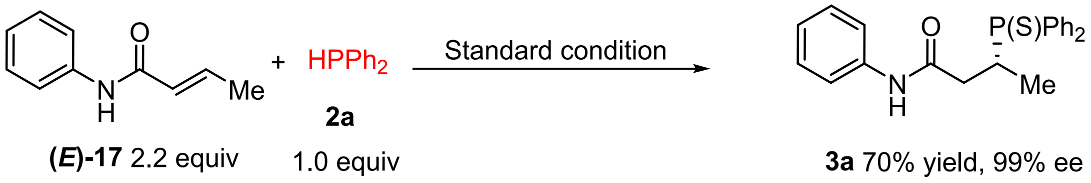

An oven-dried Schlenk tube with a stirred bar was charged with 5 mol % Ni(cod)$_2$ (1.5 mg, 0.0050 mmol), 6 mol % (*R*,*R*)-BenzP (1.8 mg, 0.0060 mmol) and 0.48 mL toluene and 0.02 mL DMF in argon atmosphere. The resulting solution was stirred at room temperature for 20 min, then **A5** (3.2 mg, 0.0200 mmol) was allowed to add and stirred for additional 10 min, then 0.22 mmol **(*E*)-17** and 0.1 mmol HPPh$_2$ **2a** were added, the mixture was stirred at 80 °C under argon atmosphere for 12 h. Then 0.2 mmol S$_8$ was added and stirred at room temperature for 3 h. The reaction mixture purified by column chromatography on silica gel to get the corresponding product **3a** (26.5 mg, 70% yield, 99% ee).

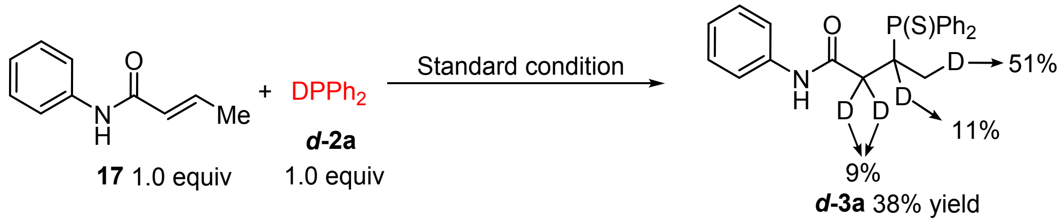

An oven-dried Schlenk tube with a stirred bar was charged with 5 mol % Ni(cod)$_2$ (1.5 mg, 0.0050 mmol), 6 mol % (*R*,*R*)-BenzP (1.8 mg, 0.0060 mmol) and 0.48 mL toluene and 0.02 mL DMF in argon atmosphere. The resulting solution was stirred at room temperature for 20 min, then **A5** (3.2 mg, 0.0200 mmol) was allowed to add and stirred for additional 10 min, then 0.22 mmol **17** and 0.1 mmol DPPh$_2$ ***d*-2a** were added, the mixture was stirred at 80 °C under argon atmosphere for 12 h. Then 0.2 mmol S$_8$ was added and stirred at room temperature for 3 h. The reaction mixture purified by column chromatography on silica gel to get the corresponding product ***d*-3a** (14.5 mg, 38% yield).

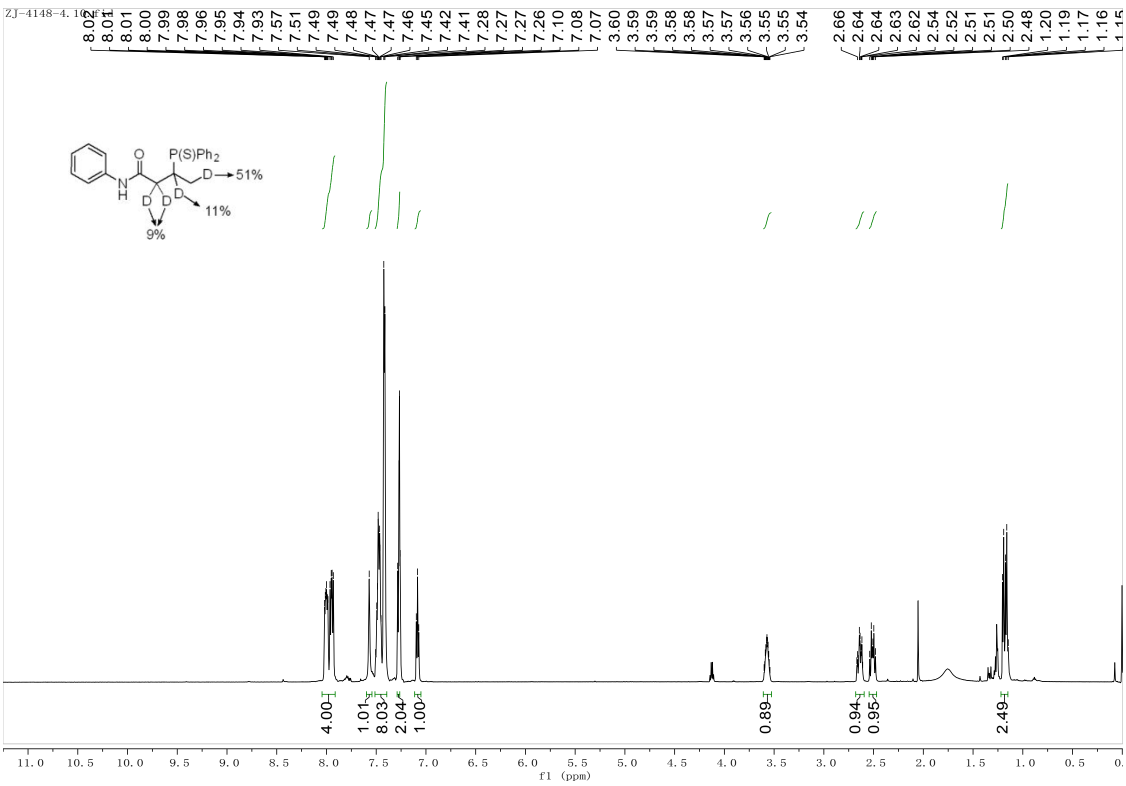

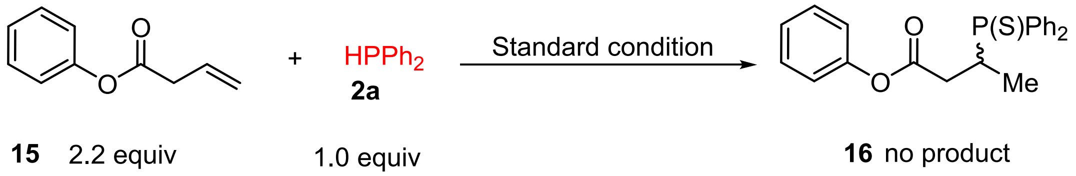

An oven-dried Schlenk tube with a stirred bar was charged with 5 mol % Ni(cod)$_2$ (1.5 mg, 0.0050 mmol), 6 mol % (*R*,*R*)-BenzP (1.8 mg, 0.0060 mmol) and 0.48 mL toluene and 0.02 mL DMF in argon atmosphere. The resulting solution was stirred at room temperature for 20 min, then **A5** (3.2 mg, 0.0200 mmol) was allowed to add and stirred for additional 10 min, then 0.22 mmol **15** and 0.1 mmol HPPh$_2$ **2a** were added, the mixture was stirred at 80 °C under argon atmosphere for 12 h. No desired product **16** was observed.

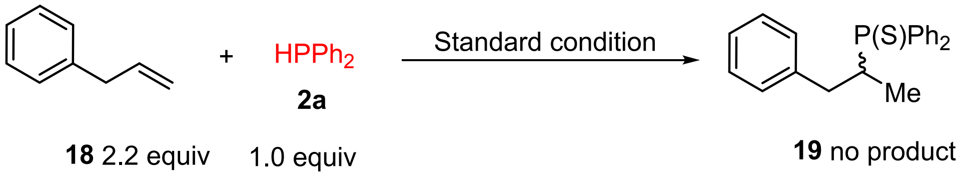

An oven-dried Schlenk tube with a stirred bar was charged with 5 mol % Ni(cod)$_2$ (1.5 mg, 0.0050 mmol), 6 mol % (*R*,*R*)-BenzP (1.8 mg, 0.0060 mmol) and 0.48 mL

toluene and 0.02 mL DMF in argon atmosphere. The resulting solution was stirred at room temperature for 20 min, then **A5** (3.2 mg, 0.0200 mmol) was allowed to add and stirred for additional 10 min, then 0.22 mmol **18** and 0.1 mmol $HPPh_2$ **2a** were added, the mixture was stirred at 80 °C under argon atmosphere for 12 h. No desired product **19** was observed.

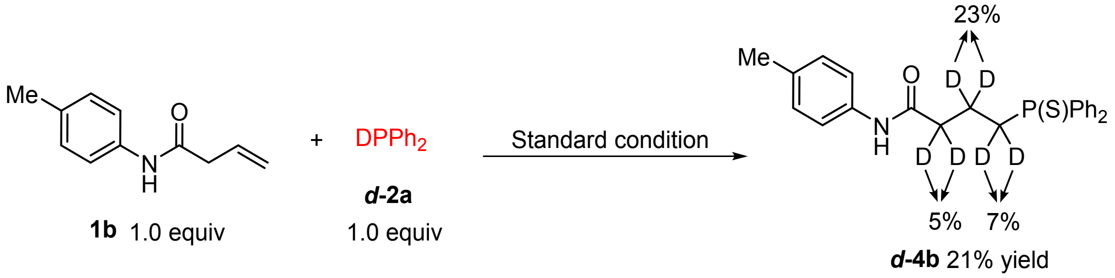

An oven-dried Schlenk tube with a stirred bar was charged with 5 mol % $Ni(cod)_2$ (1.5 mg, 0.0050 mmol), 6 mol % rac-BenzP (1.8 mg, 0.0060 mmol) and 0.48 mL toluene and 0.02 mL DMF in argon atmosphere. The resulting solution was stirred at room temperature for 20 min, then **A22** (2.6 mg, 0.0200 mmol) was allowed to add and stirred for additional 10 min, then 0.22 mmol alkene **1b** and 0.1 mmol $DPPh_2$ ***d*-2a** were added, the mixture was stirred at indicated reaction temperature under argon atmosphere for 12 h. Then 0.2 mmol S_8 was added and stirred at room temperature for 3 h. The reaction mixture purified by column chromatography on silica gel to get the corresponding product ***d*-4b** (8.4 mg, 21% yield).

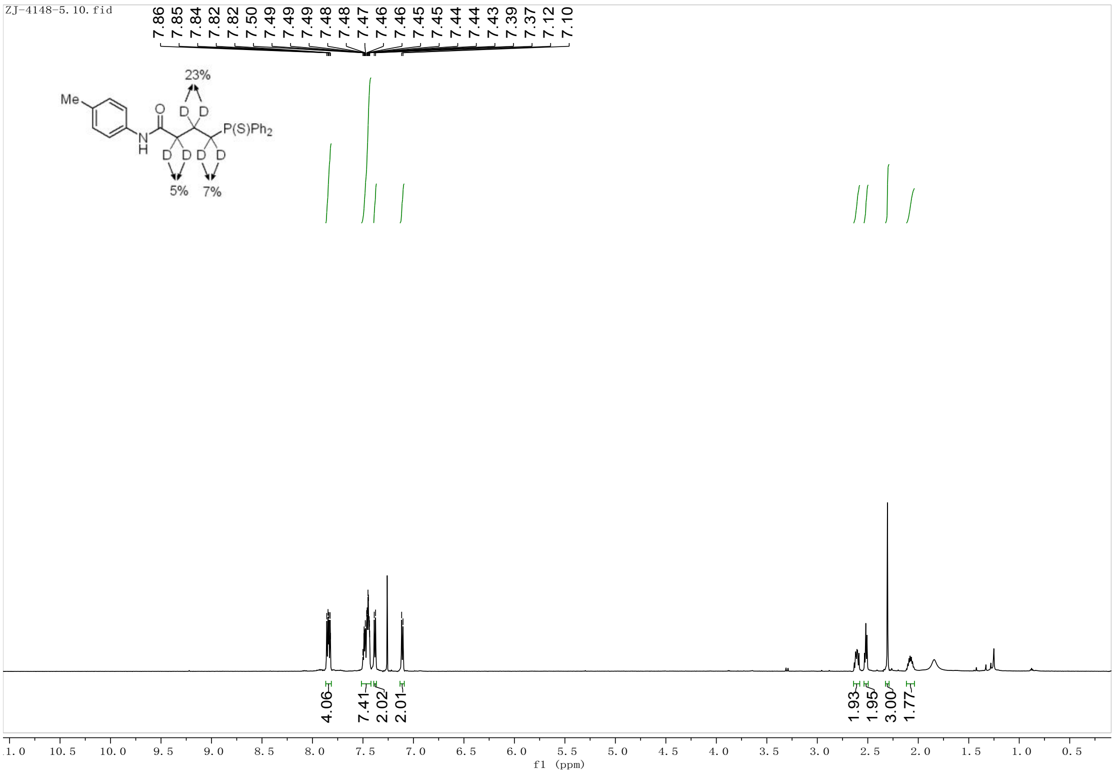

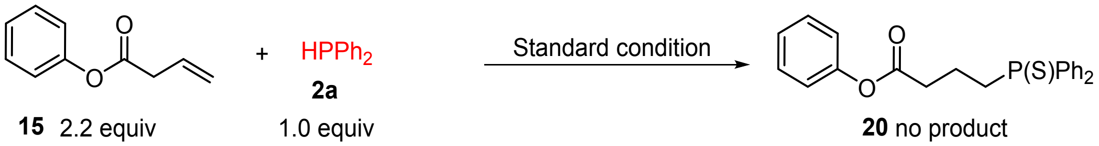

An oven-dried Schlenk tube with a stirred bar was charged with 5 mol % Ni(cod)$_2$ (1.5 mg, 0.0050 mmol), 6 mol % rac-BenzP (1.8 mg, 0.0060 mmol) and 0.48 mL toluene and 0.02 mL DMF in argon atmosphere. The resulting solution was stirred at room temperature for 20 min, then **A22** (2.6 mg, 0.0200 mmol) was allowed to add and stirred for additional 10 min, then 0.22 mmol alkene **15** and 0.1 mmol HPPh$_2$ **2a** were added, the mixture was stirred at indicated reaction temperature under argon atmosphere for 12 h. Then 0.2 mmol S$_8$ was added and stirred at room temperature for 3 h. No desired product **20** was observed.

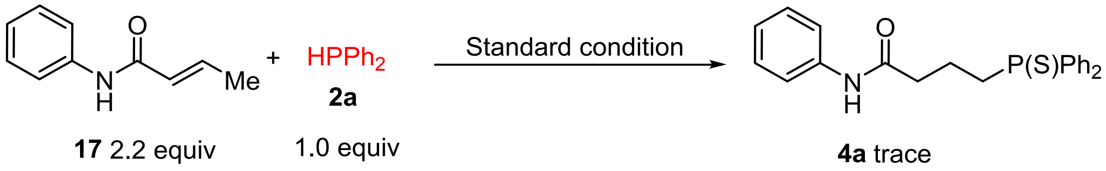

An oven-dried Schlenk tube with a stirred bar was charged with 5 mol % Ni(cod)$_2$ (1.5 mg, 0.0050 mmol), 6 mol % rac-BenzP (1.8 mg, 0.0060 mmol) and 0.48 mL toluene and 0.02 mL DMF in argon atmosphere. The resulting solution was stirred at room temperature for 20 min, then **A22** (2.6 mg, 0.0200 mmol) was allowed to add and stirred for additional 10 min, then 0.22 mmol alkene **17** and 0.1 mmol HPPh$_2$ **2a** were added, the mixture was stirred at indicated reaction temperature under argon atmosphere for 12 h. Then 0.2 mmol S$_8$ was added and stirred at room temperature for 3 h. No desired product **4a** was observed.

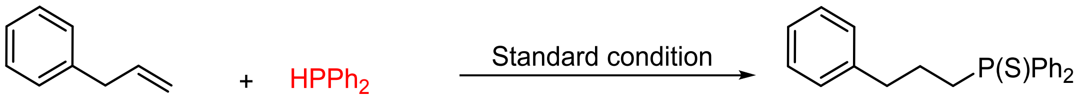

17 2.2 equiv   **2a** 1.0 equiv   **21** 15% yield

An oven-dried Schlenk tube with a stirred bar was charged with 5 mol % Ni(cod)$_2$ (1.5 mg, 0.0050 mmol), 6 mol % rac-BenzP (1.8 mg, 0.0060 mmol) and 0.48 mL toluene and 0.02 mL DMF in argon atmosphere. The resulting solution was stirred at room temperature for 20 min, then **A22** (2.6 mg, 0.0200 mmol) was allowed to add and stirred for additional 10 min, then 0.22 mmol alkene **17** and 0.1 mmol HPPh$_2$ **2a** were added, the mixture was stirred at indicated reaction temperature under argon atmosphere for 12 h. Then 0.2 mmol S$_8$ was added and stirred at room temperature for 3 h. The reaction mixture purified by column chromatography on silica gel to get the corresponding product **21** (5.0 mg, 15% yield). ^{1}H NMR (400 MHz, Chloroform-d) δ 7.85-7.77 (m, 4H), 7.50-7.42 (m, 6H), 7.26-7.22 (m, 2H), 7.19-7.14 (m, 1H), 7.13-7.08 (m, 2H), 2.59 (t, J = 7.2 Hz, 2H), 2.51-2.42 (m, 2H), 1.73-1.70 (m, 2H). ^{31}P NMR (162 MHz, Chloroform-*d*) δ 42.61. ^{13}C NMR (101 MHz, Chloroform-*d*) δ 142.06, 133.40, 132.61, 131.56 (d, *J* = 3.0 Hz), 131.20 (d, *J* = 10.0 Hz), 128.77 (d, *J* = 12.0 Hz), 128.46 (d, *J* = 3.2 Hz), 125.95, 35.57, 32.40 (d, *J* = 16.7 Hz), 22.13 (d, *J* = 2.7 Hz).

8. Computational Studies

8.1 Computational Methods

8.1.1 Conformational sampling

Conformational sampling was performed using Grimme's *CREST* program[11,12], which used metadynamics (MTD) with genetic z-matrix crossing (GC) performed at theGFN2-xTB[13-15] extended semiempirical tight-binding level of theory with *opt=vtight* option. The isolated conformers were used for further density functional theory (DFT) calculations.

8.1.2 Density functional theory (DFT) calculations

Geometry optimizations were carried out using *Gaussian 16* rev. B.01 software[16] in the gas phase using the global-hybrid meta-NGA (nonseparable gradient approximation) MN15 functional[17] and the def2-SVP[18,19] basis set for all atoms. Truhlar's MN15 functional was chosen to study the present system, as this functional has been employed in the studies of a range of organometallic systems with good accuracy[20-27] Minima and transition structures on the potential energy surface (PES) were confirmed as such by harmonic frequency analysis, showing respectively zero and one imaginary frequency. To improve on the accuracy of the corrected Gibbs energy profile, single point (SP) calculations on the gas phase optimized geometries were performed at MN15 with def2-TZVP[18,19] basis set for all atoms in the implicit C-PCM continuum solvation model[28,29] to model the effect of toluene : DMF (0.48 : 0.02) mixed solvent that was used experimentally, on the potential energy surface. Following our previous work[30], a linearly interpolated dielectric constant (ε) value of 1.8764 for the solvent mixture, toluene : DMF = 0.48 : 0.02, was used ($2.38 \times 0.48 + 36.7 \times 0.02$). We use simple linear interpolation for generality to other solvent mixtures for future work. To verify the results, and since pure toluene solvent can be used to achieve the same transformation (albeit with reduced yield and selectivity (Table S7), we separately run single point (SP) calculations on the gas phase optimized geometries at MN15/def2-TZVP[8,9] in the implicit SMD solvation model for toluene. We note that the values produced from both levels of theory are consistent with each other and give the same conclusion.

Gibbs energies were evaluated at the reaction temperature of 80 ºC, using Grimme's scheme of quasi-RRHO treatment of vibrational entropies[31], using the GoodVibes code[32]. Vibrational entropies of frequencies below 100 cm^{-1} were obtained according to a free rotor description, using a smooth damping function to interpolate between the two limiting descriptions[31]. The free energies reported in *Gaussian* from gas-phase optimization were further corrected using standard concentration of 1 mol/L[33], which were used in solvation calculations, instead of the gas-phase 1atm used by default in the *Gaussian* program.

Unless otherwise stated, the final corrected Gibbs energy C-PCM(toluene-DMF)-MN15/def2-TZVP//MN15/def2-SVP is used for discussion. The Gibbs energies in SMD(toluene)-MN15/def2-TZVP//MN15/def2-SVP are included in square brackets. All Gibbs energy values in the text and figures are quoted in kcal mol^{-1}.

Optimized structures and molecular orbitals are visualized using *PyMOL* software[34].

8.2 Model reactions

Scheme S1 shows the model reaction that we used for computational studies of the reaction mechanism for the present reaction. Using pyridine-3-sulfonic acid (reaction on the left), no anti-Markovnikov product was observed and the Markovnikov product was formed in 73% yield with 99% ee. On the other hand, using 3,5-difluorophenol (reaction on the right), only anti-Markovnikov product was formed and no Markovnikov product was observed.

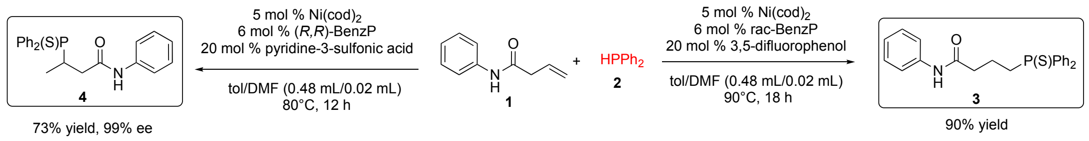

Scheme S1. Model reaction used in computational modelling.

8.3 Conformational Considerations

The bisphosphine ligated Ni-complex where the olefin coordinates via C=C double bond was conformationally sampled to locate the most stable species. We note that, due to the chiral nature of the bisphosphine ligand, the C=C bond can coordinate in two different orientations. The DFT optimized structures of these two coordination modes

are shown in Figure S1. Using each of these structures (**INT1** and **INT1'**) as an initial guess and running the CREST conformational sampling, the results both converge to the same most stable structure, **INT1** after DFT optimization; in other words, CREST only locates one form of the conformer as the most stable structure on the GFN2-xTB potential energy surface, despite having two different initial guess structures. We note that in an initial guess structure where the O atom of the substrate is coordinated to Ni center in **INT1**, the optimized structure has O that is uncoordinated (Figure S1). The amide O atom in **INT1** has a non-covalent interaction with the C–H bond of the *tert*-butyl group on the ligand. On the other hand, in **INT1'**, the amide O atom interacts with Ni center to form an elongated Ni–O bond of 2.44 Å. Interestingly, despite this, **INT1'** is higher in Gibbs energy than **INT1** by 4.5 [4.0] kcal/mol.

**INT1**	**INT1'**
ΔG = 0.0 [0.0]	ΔG = 4.5 [4.0]
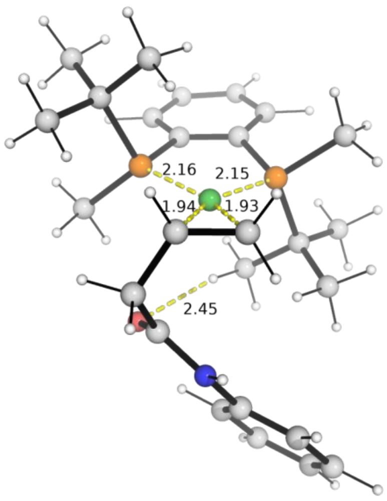	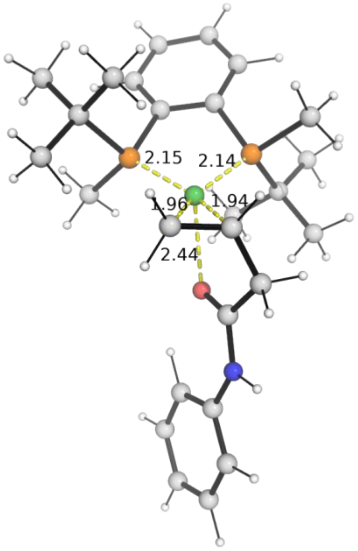
**INT1o**	
ΔG = 26.7 [26.4]	

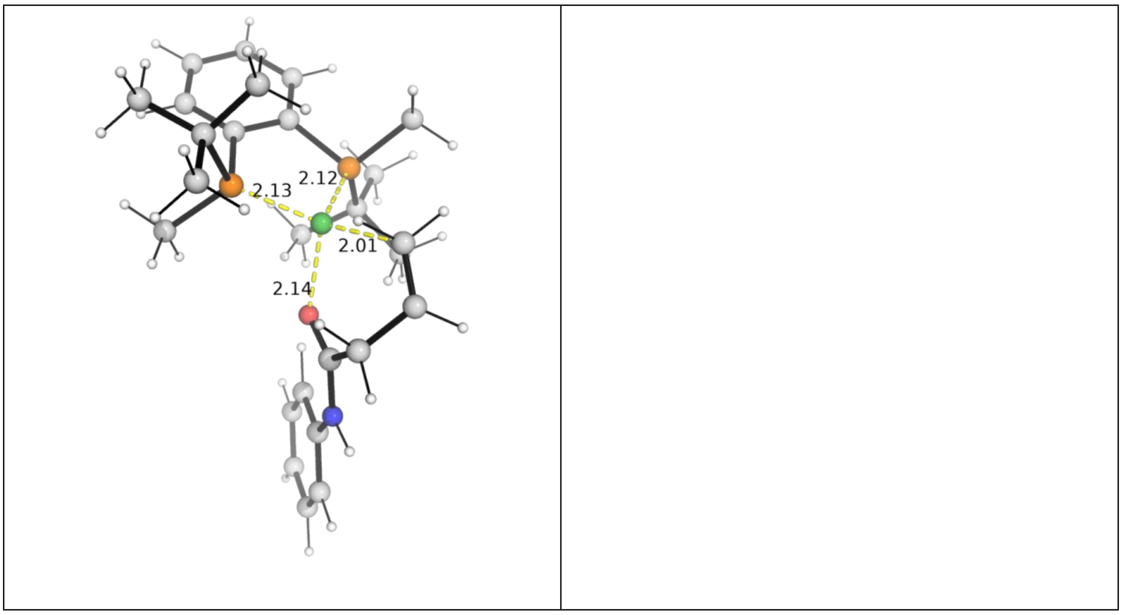

Figure S1. DFT-optimized structures of different conformers of substrate bound to the Ni catalyst. Gibbs energies are given in C-PCM(toluene-DMF)[SMD(toluene)]-MN15/def2-TZVP//MN15/def2-SVP level of theories.

We also try to start from a square planar guess structure where the amide O atom coordinates to the Ni center directly. The optimized structure, **IN1o**, is much higher in energy, by 26.7 [26.4] kcal/mol, than **INT1**, thus, it is thermodynamically less favorable to form O-coordinated **INT1o**.

8.4 Reactivity with pyridine-3-sulfonic acid

As a note, for structures involved in the reaction with pyridine-3-sulfonic acid, the suffix “A” is added after the number. For example, **TS1** leading from **INT1** in this reaction will be denoted **TS1A** and **TS1’** leading from **INT1’** will be denoted as **TS1A’**.

8.4.1 Protonation step

After conformational sampling, we see that for substrate **INT1**, pyridine-3-sulfonic acid can protonate either the terminal carbon of the olefin, via **TS1A_Cterm**, or the internal carbon of the olefin, via **TS1A_Cin** (Figure S3). Alternatively, it can also protonate either carbon of the olefin coordinated in **INT1’**, via **TS1A’_Cterm** or **TS1A’_Cterm**. We located all these TSs and found that protonation of the terminal carbon via **TS1A_Cterm** has the lowest barrier, at 9.5 [11.0] kcal/mol, whereas the protonation of internal carbon via **TS1A’_Cin** has a higher barrier, at 11.6 [13.0] kcal/mol (Figure

S4). This barrier difference of 2.1 [2.0] kcal/mol ($\Delta\Delta G^{\ddagger}$) translates to a d.r. of about 20 : 1 at the reaction temperature of 80ºC, using simple transition state theory.

It is worth noting that the protonation from the approach direction of least steric hinderance (methyl group instead of tert-butyl group) is more favourable than from the other direction (tert-butyl group instead of methyl group). For example, comparing **TS1A_Cterm** and **TS1A_Cin**, the former has a lower barrier than the latter; similarly, comparing **TS1A'_Cin** and **TS1A'_Cterm**, the former has a lower barrier than the latter. In both **TS1A_Cterm** and **TS1A'_Cin**, protonation occurs from the right hand side, whereas in **TS1A_Cin** and **TS1A'_Cterm**, protonation occurs from the left hand side. Structurally, protonation from the right hand side (**TS1A_Cterm** and **TS1A'_Cin**) preserves the planar structure of Ni coordination whereas protonation from the left hand side (**TS1A_Cin** and **TS1A'_Cterm**) distorts the square planar geometry, thus introducing more strains, resulting in elevated activation barriers.

Looking at the conformers of the transition states, we observe favorable interaction between C–H bond of on the *tert*-butyl group and the amide oxygen atom in many TS structures. It is also possible that the amide N–H bond coordinates to Ni-center to stabilize the transition state. The lowest energy TS, **TS1A_Cterm**, may undergo an inner-sphere protonation where an oxygen atom on the sulfone group coordinates to Ni-center at a distance of 2.92Å, while another oxygen atom carries out deprotonation.

**TS1A_Cterm**	**TS1A_Cterm_c2**
$\Delta G^{\ddagger}$ = 9.5 [11.0]	$\Delta G^{\ddagger}$ = 9.8 [11.3]

<table>
<tr><td>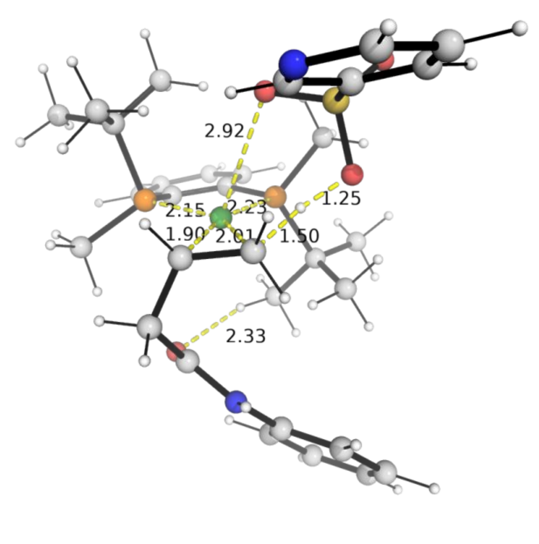
</td><td>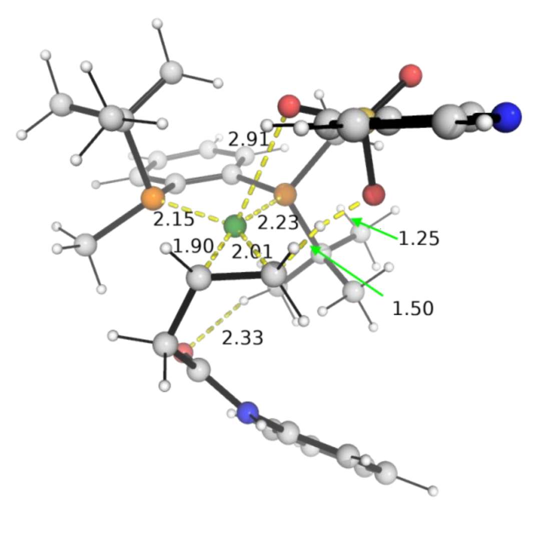
</td></tr>
<tr><td>TS1A_Cterm_c3</td><td>TS1A_Cterm_c4</td></tr>
<tr><td>$\Delta G^{\ddagger}$ = 10.1 [11.0]</td><td>$\Delta G^{\ddagger}$ = 12.5 [13.1]</td></tr>
<tr><td>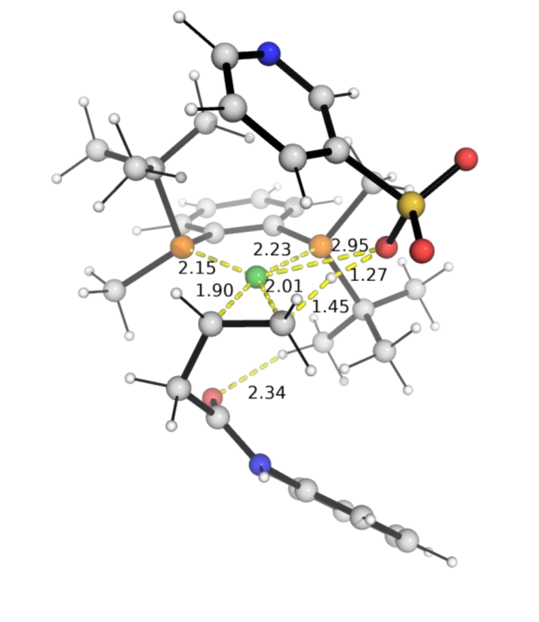
</td><td>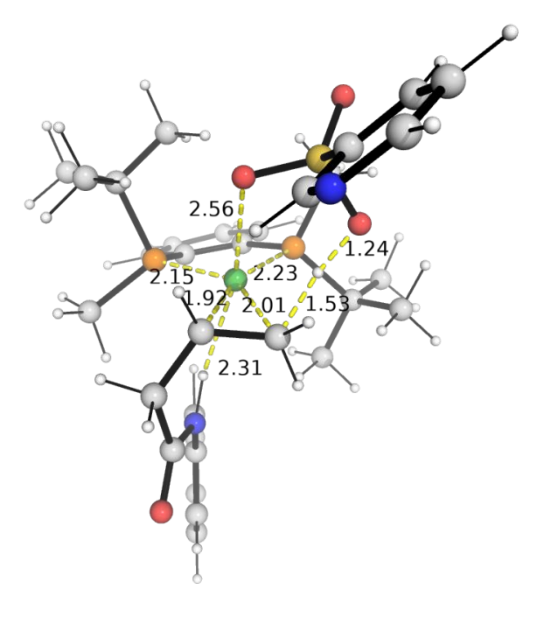
</td></tr>
<tr><td>TS1A_Cin</td><td>TS1A’_Cterm</td></tr>
<tr><td>$\Delta G^{\ddagger}$ = 16.9 [18.0]</td><td>$\Delta G^{\ddagger}$ = 19.2 [20.1]</td></tr>
</table>

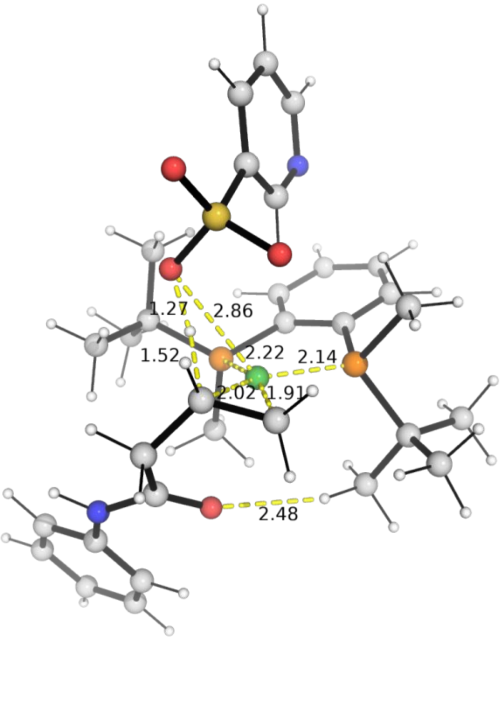	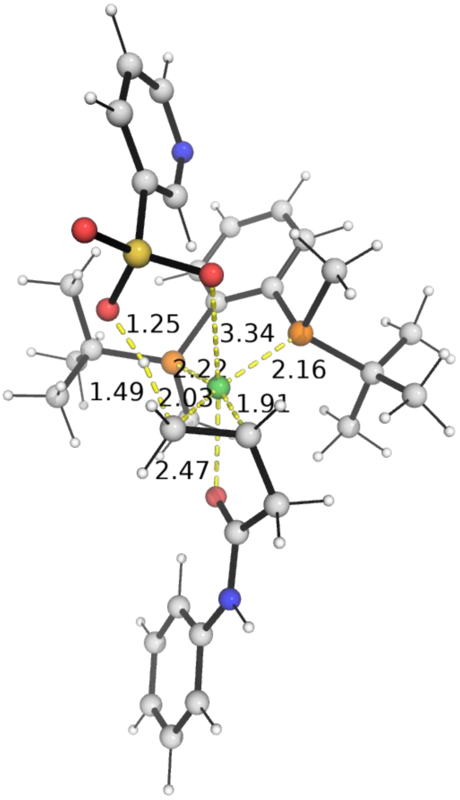
**TS1A'_Cin**	**TS1A'_Cin_c2**
ΔG‡ = 11.6 [13.0]	ΔG‡ = 13.0 [13.7]
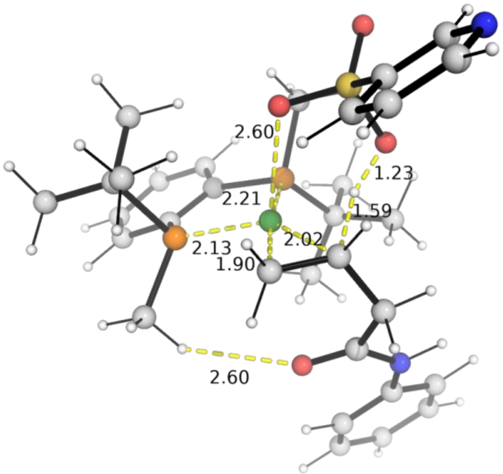	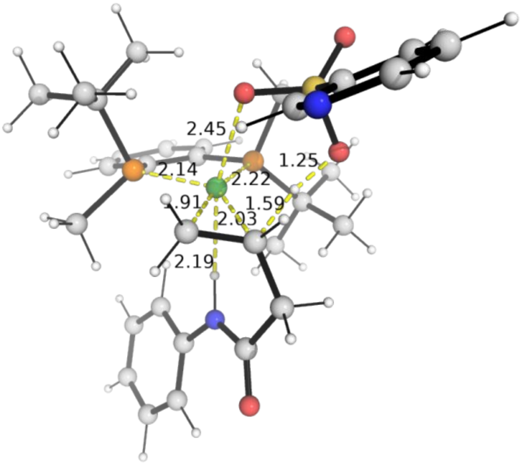
**TS1A'_Cin_c3**	
ΔG‡ = 14.0 [15.4]	

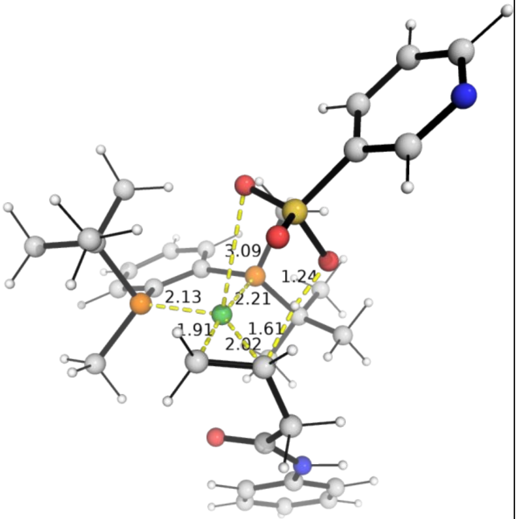

Figure S2. DFT-optimized structures of different conformers of first C–C bond formation transition states (TSs) using pyridine-3-sulfonic acid as the proton source. Gibbs energies relative are given relative to the ground state of **INT1**. Gibbs energies are given in C-PCM(toluene-DMF)[SMD(toluene)]-MN15/def2-TZVP//MN15/def2-SVP level of theories.

8.4.2 Competing transition states

We further analyze the factors influencing the selectivities by comparing the frontier molecular orbitals (FMOs), non-covalent interactions and distortion-interaction analysis in the lowest energy competing TSs, **TS1A_Cterm**, **TS1A_Cin**, **TS1A'_Cterm** and **TS1A'_Cin**. The results are shown in Figure S3 and Table S8.

	**TS1A_Cterm**	**TS1A_Cin**
**barrier**	$\Delta G^{\ddagger} = 9.5$ [11.0]	$\Delta G^{\ddagger} = 16.9$ [18.0]

**DFT Structure**	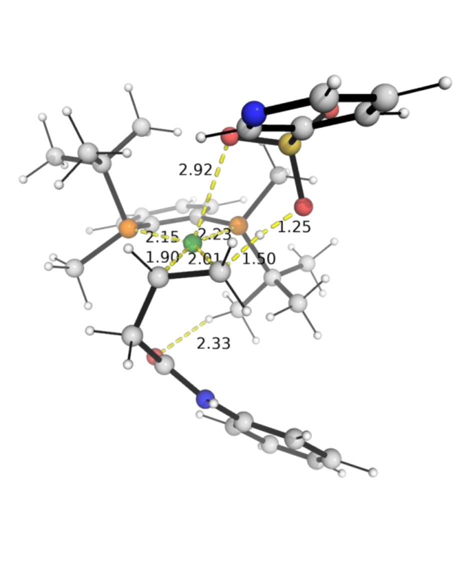	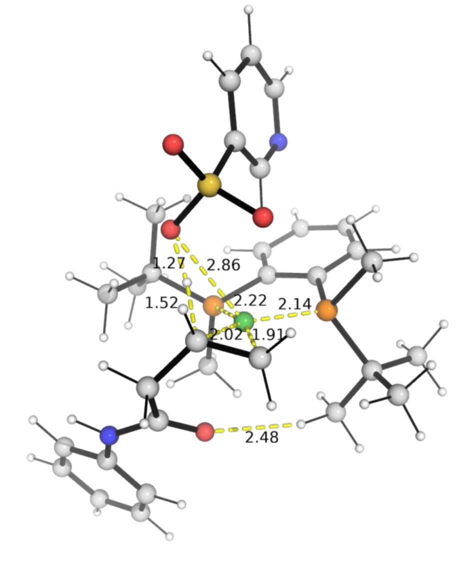
**HOMO**	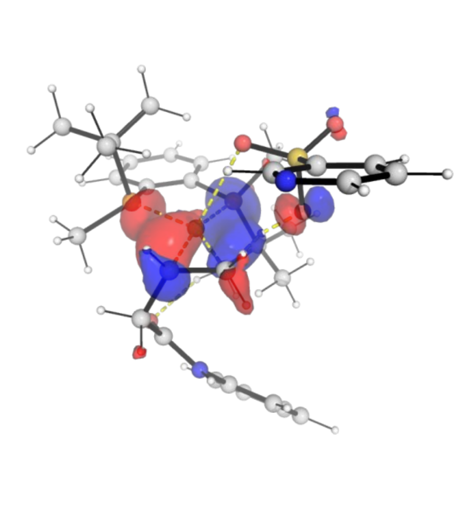	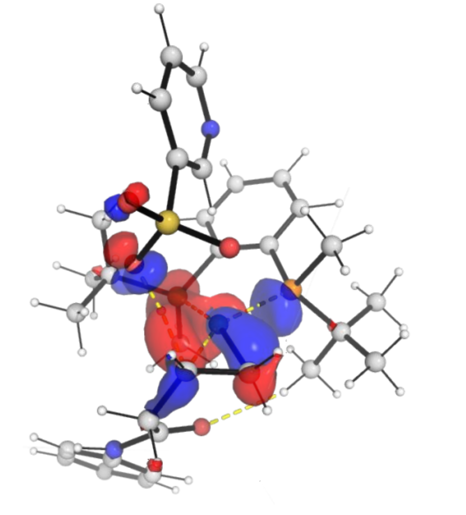
**LUMO**	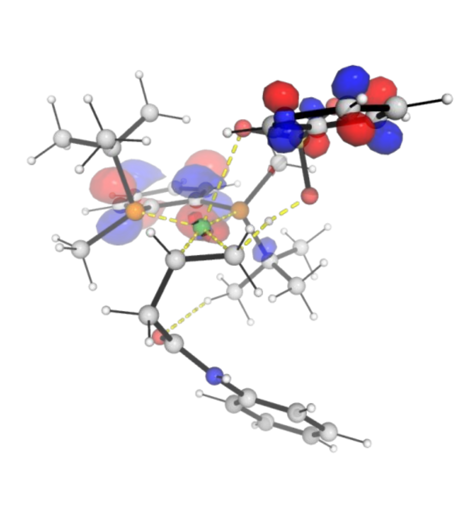	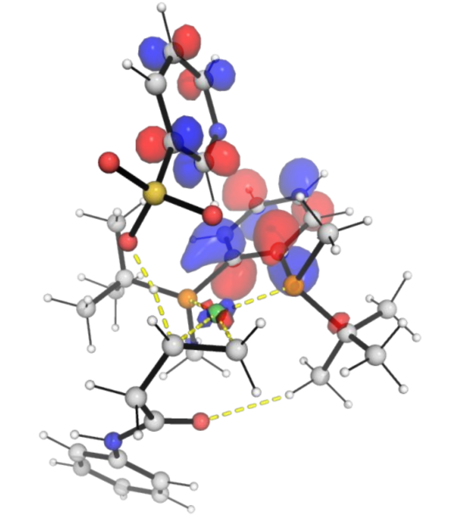

**NCI**	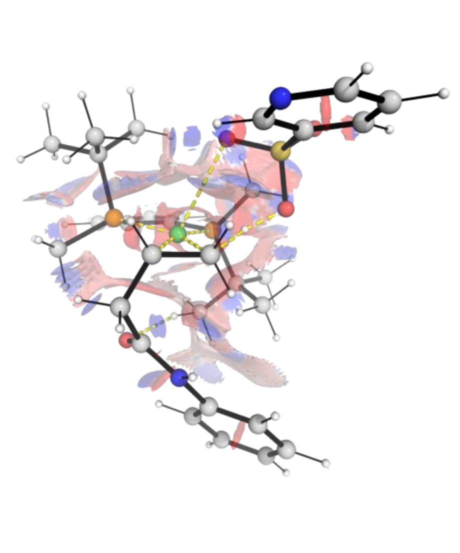	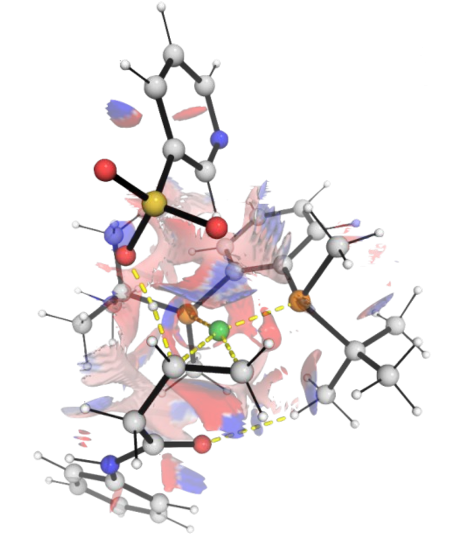
	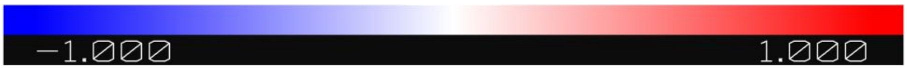	
	**TS1A'_Cterm**	**TS1A'_Cin**
**barrier**	ΔG‡ = 19.2 [20.1]	ΔG‡ = 11.6 [13.0]
**DFT Structure**	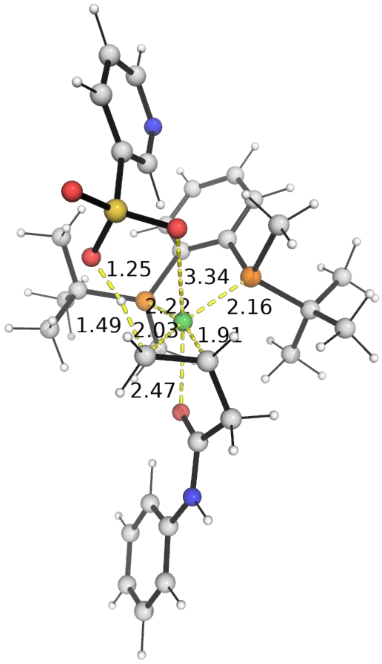	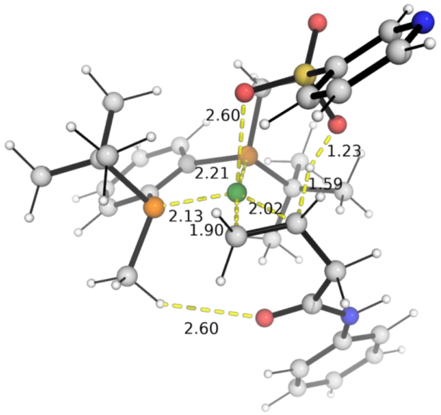

**HOMO**	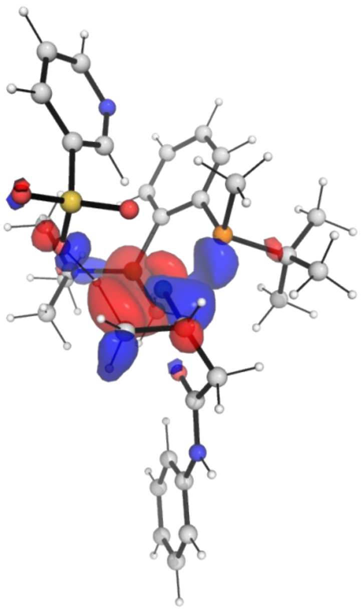	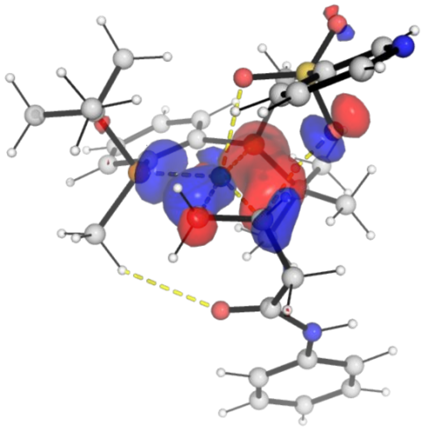
**LUMO**	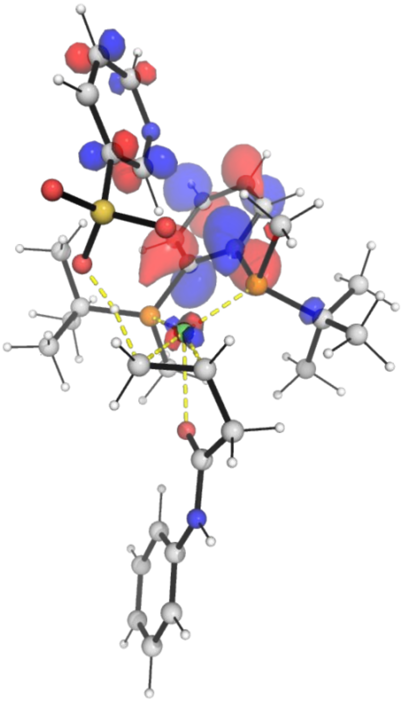	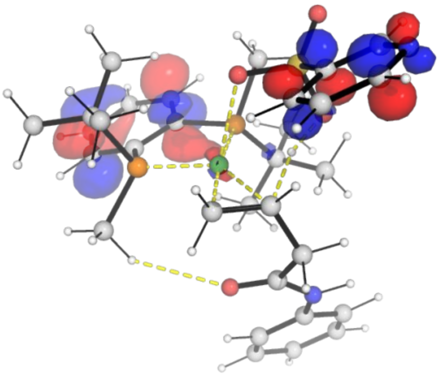

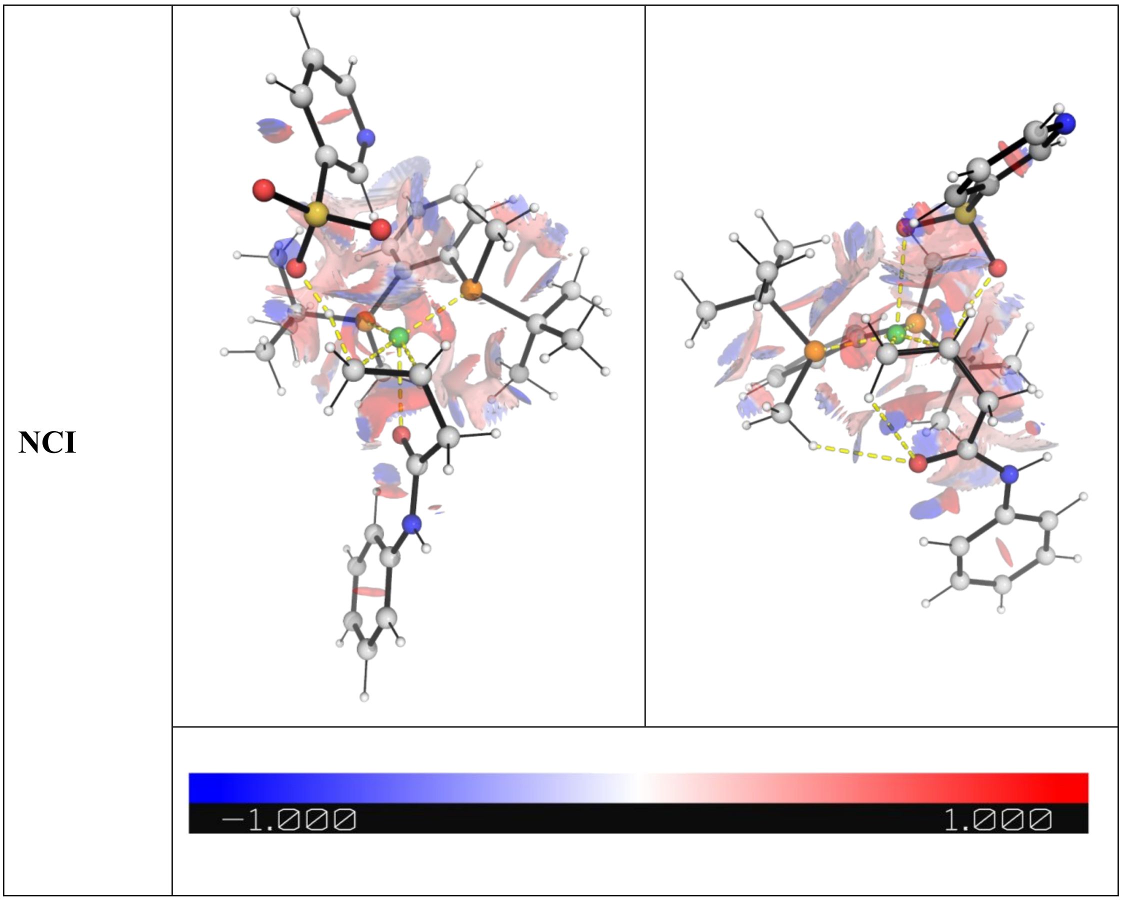

Figure S3. DFT-optimized structures, frontier molecular orbitals (HOMO and LUMO) and non-covalent interaction (NCI) plots for the lowest barrier transition state for the first C–C bond formation transition states (TSs) using pyridine-3-sulfonic acid as the proton source.

Distortion-interaction[35,36], analysis is applied to key TSs to discern the factors affecting regioselectivity. The transition state structures are decomposed by dividing the acid and the olefin-bound Ni-species as components. Single point calculations at C-PCM(toluene-DMF) solvent correction were applied performed at M06-2x/def2-TZVP level of theory to obtain distortion and interaction energies. The distortion energy is given by:

$$E_{dist} = E_{TS,frag1} + E_{TS,frag2} - (E_{eq,frag1} + E_{eq,frag2})$$

where *TS,frag1,2* represent individual fragments in their distorted transition state geometries; and *eq,frag1,2* represent individual fragments in their optimized, equilibrium ground-state geometries; the interaction energy is given by:

$$E_{int} = E_{TS} - (E_{TS,frag1} + E_{TS,frag2})$$

which accounts for the stabilizing interactions (e.g., electrostatic, orbital, dispersion) between the distorted fragments in the TS.

Thus, the total activation energy is given by:

$$\Delta E^{\ddagger} = E_{dist} + E_{int.}$$

Note that this single point activation energy and the activation energy differences $\Delta\Delta E^{\ddagger}$ between the major and minor TSs may be different from the Gibbs energy differences $\Delta\Delta G^{\ddagger}$ that is computed fully (including vibrational frequencies analysis) at C-PCM(toluene-DMF)-MN15/def2-TZVP//MN15/def2-SVP level of theory.

Table S8. Distortion-interaction analysis for the protonation step using pyridine-3-sulfonic acid.

Transition State	$\Delta E^{\ddagger}$	E_{dist}	E_{int}
**TS1A_Cterm**	-5.4	37.1	-42.5
**TS1A_Cin**	3.0	48.5	-45.5
**TS1A'_Cterm**	5.7	49.5	-43.8
**TS1A'_Cin**	-2.7	39.6	-42.3

This analysis shows that **TS1A_Cterm** has the lowest barrier and is more stable than **TS1A_Cin** by 8.4 kcal/mol, which results from much lower distortion energy, by 11.4 kcal/mol, when the two components approach each other despite the interaction energy that is 3.0 kcal/mol less stabilised. For the protonation of **INT1'**, **TS1A'_Cin** has a lower barrier by 8.4 kcal/mol than **TS1A'_Cterm**, due to the former having much lower distortion energy, by 9.9 kcal/mol, although the interaction energy is slightly less stable, by 1.5 kcal/mol. Thus, the protonation from the approach direction of least steric hinderance (methyl group instead of tert-butyl group) is more favourable (**TS1A_Cterm** and **TS1A'_Cin**) than from the other direction (tert-butyl group instead of methyl group, **TS1A_Cin** and **TS1A'_Cterm**).

In terms of regioselectivity outcome, **TS1A_Cterm** is favoured over **TS1A'_Cin**, by $\Delta\Delta E^{\ddagger}$ = 2.7 kcal/mol, primarily due to the smaller distortion in the former than the latter, by 2.5 kcal/mol, whereas the interaction E_{int} is similar in both TSs.

8.4.3 Oxidative addition of O–H of pyridine-3-sulfonic acid to Nickel

The possibility of oxidative addition of O–H of pyridine-3-sulfonic acid to nickel was explored, however, in the process of TS search and the optimization of a potential Ni–H species, it was shown that such Ni–H species could not be formed and that the H atom on Ni-center will add to olefin C=C bond upon geometry optimization, even if we started with a guess Ni–H structure.

8.4.4 Reductive elimination step

**TS2A**	
$\Delta G^{\ddagger}$ = -13.9 [-11.0]	
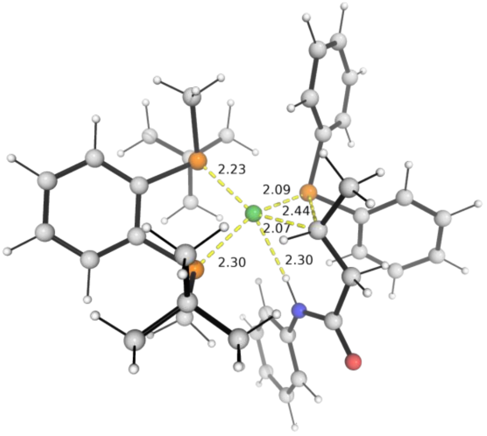	

Figure S4. DFT-optimized structures of the reductive elimination step, **TS2A**.

8.5 Reactivity with 3,5-difluorophenol

For the reaction with 3,5-difluorophenol additive, the suffix "B" is added after the number. For example, **TS1** leading from **INT1** in this reaction will be denoted **TS1B** and **TS1'** leading from **INT1'** will be denoted as **TS1B'**.

8.5.1 Protonation step

Similar to the protonation by pyridine-3-sulfonic acid discussed in the previous section, for **INT1**, the protonation of olefin can occur at either carbon of the C=C bond, via **TS1B_Cterm** and **TS1B_Cin**, Figure S5; for **INT1'**, via **TS1B'_Cterm** and

TS1B'_Cin, Figure S5. As for pyridine-3-sulfonic acid, the protonation from the right hand side has lower barriers than from the left hand side (approach of least steric hinderance). Using 3,5-difluorophenol, the protonation of internal olefin on **INT1'**, via **TS1B'_Cin**, has the lowest barrier, at 24.3 [25.2] kcal/mol. On the other hand, the protonation of terminal olefin has a barrier of 30.1 [30.6] kcal/mol, via **TS1B_Cterm**. This barrier difference of 5.8 [5.4] kcal/mol ($\Delta\Delta G^{\ddagger}$) translates to a d.r. of about 2200–3900 : 1 at the reaction temperature of 80ºC, using simple transition state theory, indicating that protonation by 3,5-difluorophenol predominantly occurs on terminal carbon of the C=C bond of the substrate.

**TS1B_Cterm**	**TS1B_Cin**
$\Delta G^{\ddagger}$ = 30.1 [30.6]	$\Delta G^{\ddagger}$ = 31.7 [31.1]
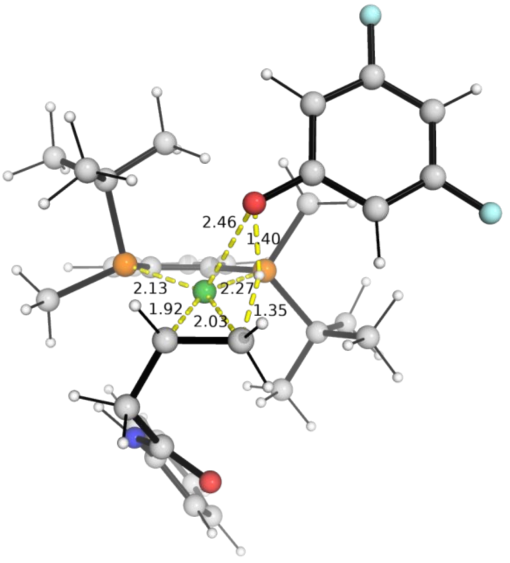	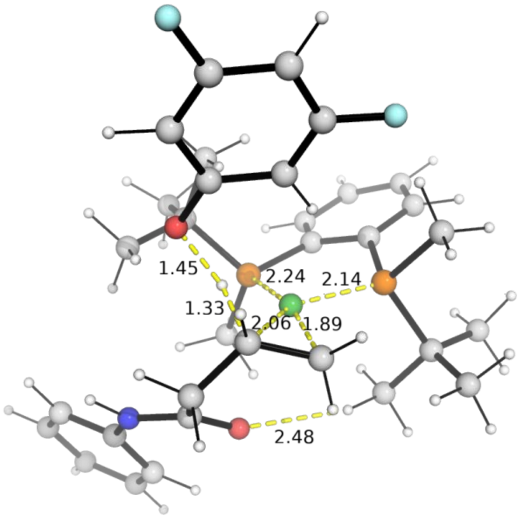
**TS1B_Cin_c2**	**TS1B_Cin_c3**
$\Delta G^{\ddagger}$ = 31.7 [31.9]	$\Delta G^{\ddagger}$ = 31.7 [33.1]

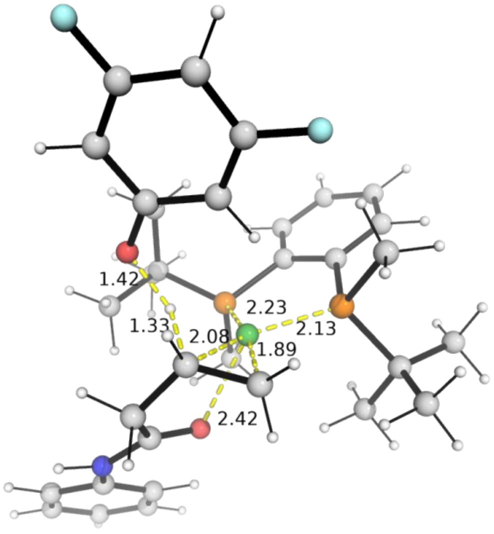	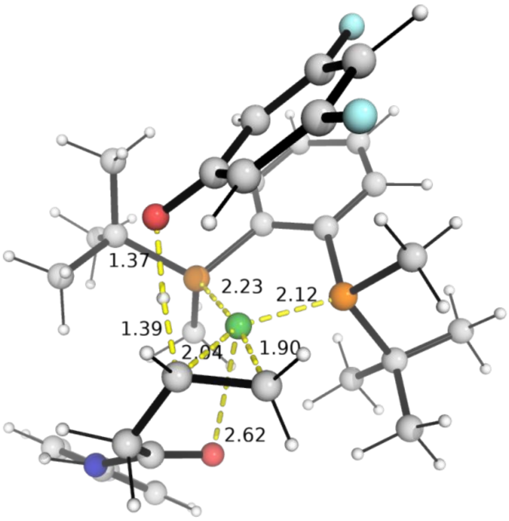
**TS1B_Cin_c4**	**TS1B’_Cterm**
ΔG‡ = 34.2 [35.6]	ΔG‡ = 32.2 [33.0]
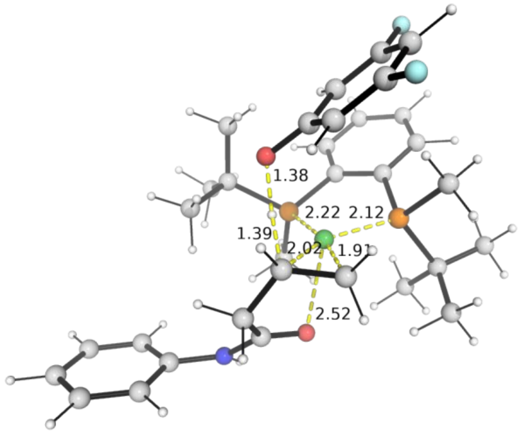	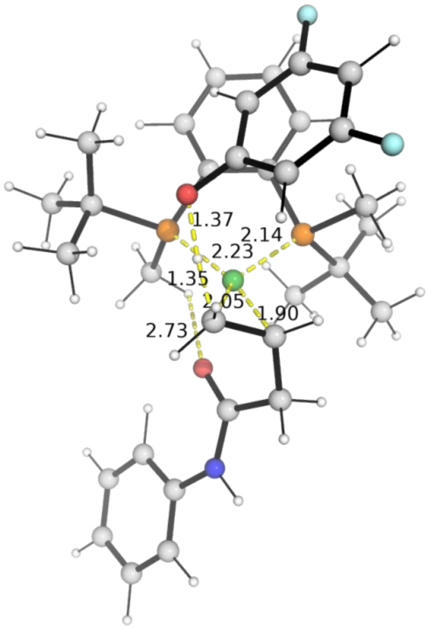
**TS1B'_Cterm_c2**	**TS1B'_Cterm_c3**
ΔG‡ = 34.0 [35.6]	ΔG‡ = 32.2 [33.0]

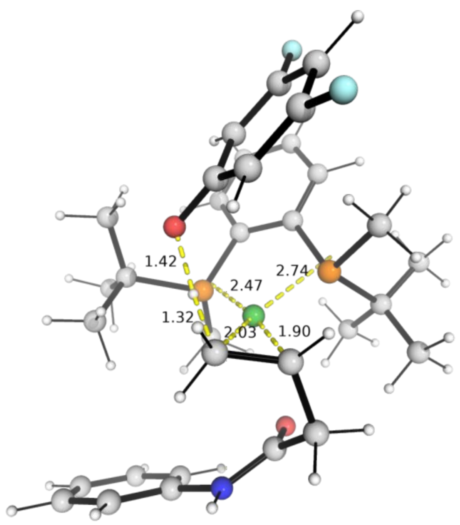

TS1B'_Cin

ΔG‡ = 24.3 [25.2]

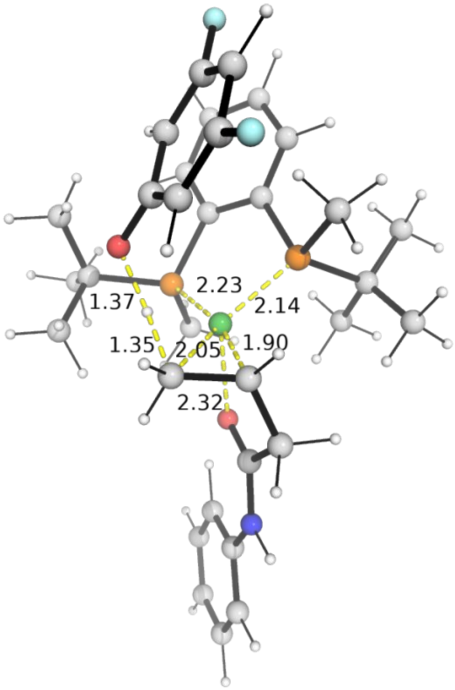

TS1B'_Cin_c2

ΔG‡ = 26.4 [27.7]

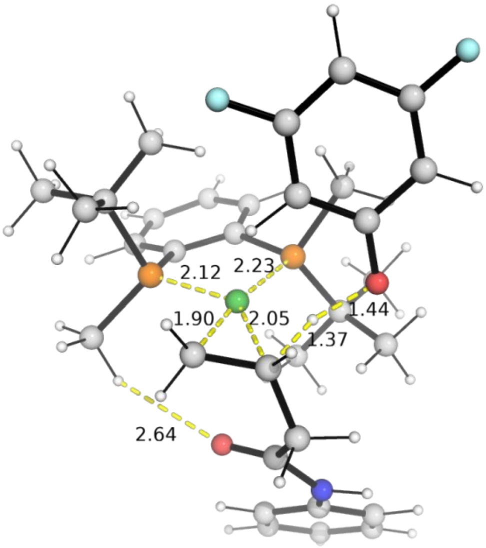

TS1B'_Cin_c3

ΔG‡ = 31.8 [32.3]

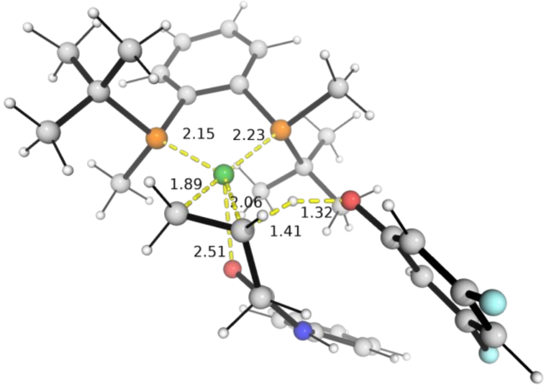

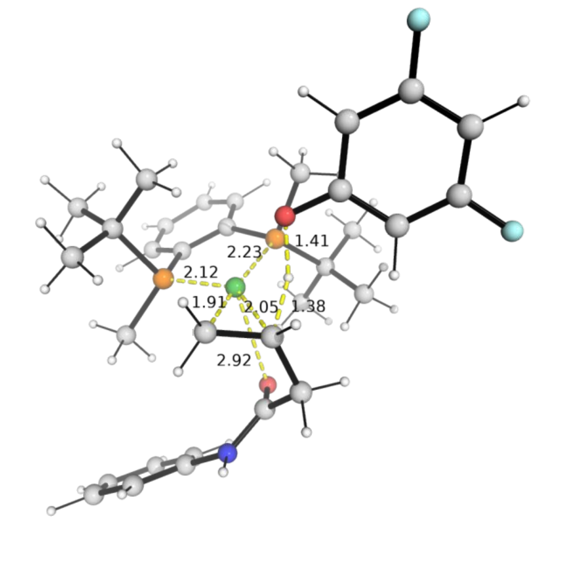

Figure S5. DFT-optimized structures of different conformers of first C–C bond formation transition states (TSs) using 3,5-difluorophenol as the proton source. Gibbs energies relative are given relative to the ground state of **INT1**. Gibbs energies are given in C-PCM(toluene-DMF)[SMD(toluene)]-MN15/def2-TZVP//MN15/def2-SVP level of theories.

8.5.2 Competing transition states

We further analyze the factors influencing the selectivities by comparing the frontier molecular orbitals (FMOs), non-covalent interactions and distortion-interaction analysis in the lowest energy competing TSs, **TS1B_Cterm**, **TS1B_Cin**, **TS1B'_Cterm** and **TS1B'_Cin**. The results are shown in Figure S6 and Table S9.

	**TS1B_Cterm**	**TS1B_Cin**
barrier	$\Delta G^{\ddagger}$ = 30.1 [30.6]	$\Delta G^{\ddagger}$ = 31.7 [31.1]

DFT Structure	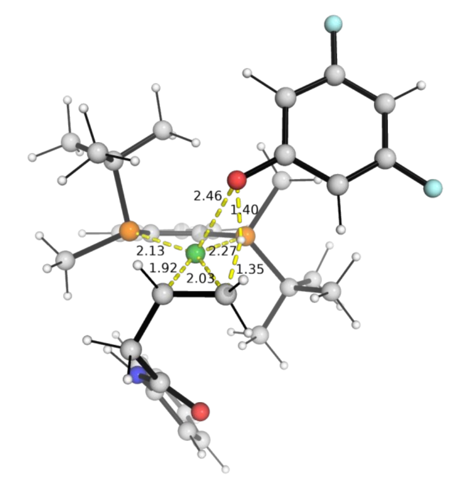	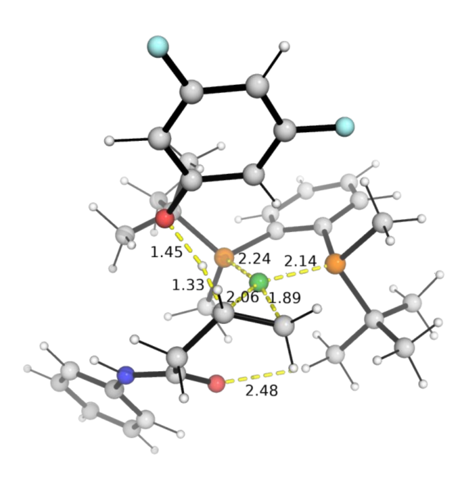
HOMO	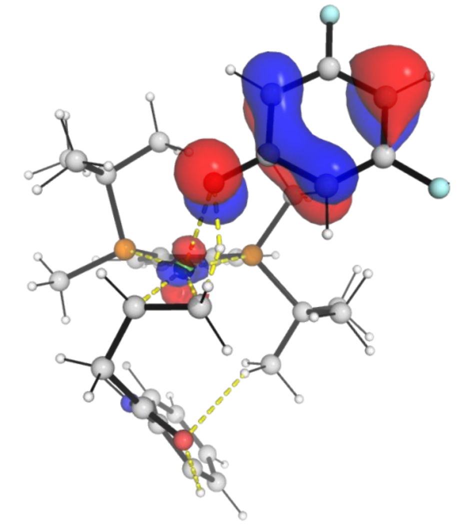	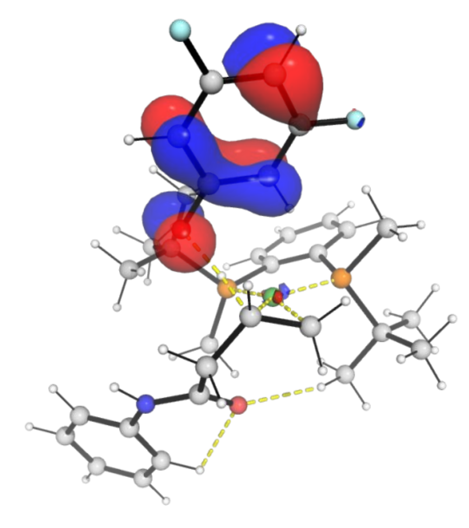
LUMO	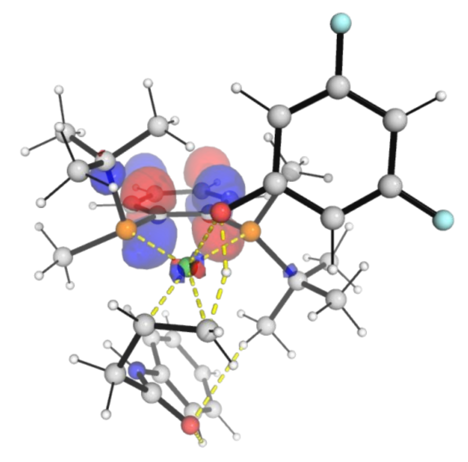	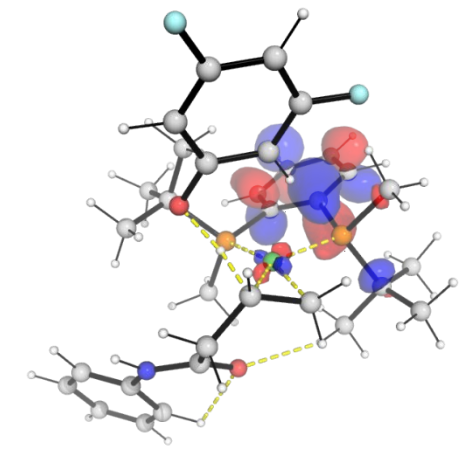

NCI	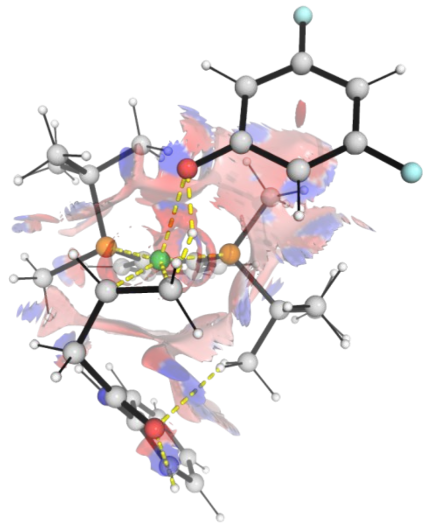	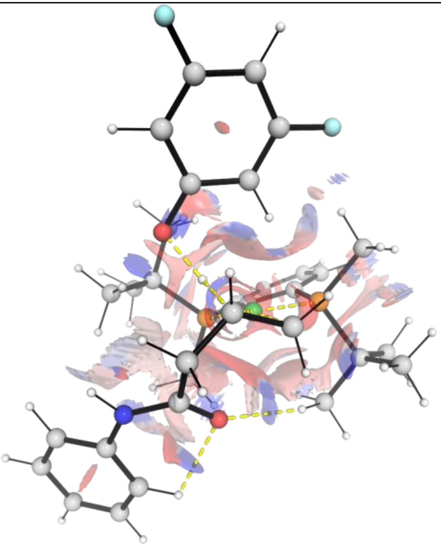
	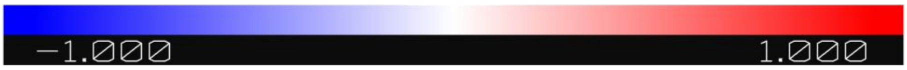	
	**TS1B'_Cterm**	**TS1B'_Cin**
barrier	ΔG‡ = 32.2 [33.0]	ΔG‡ = 24.3 [25.2]
DFT Structure	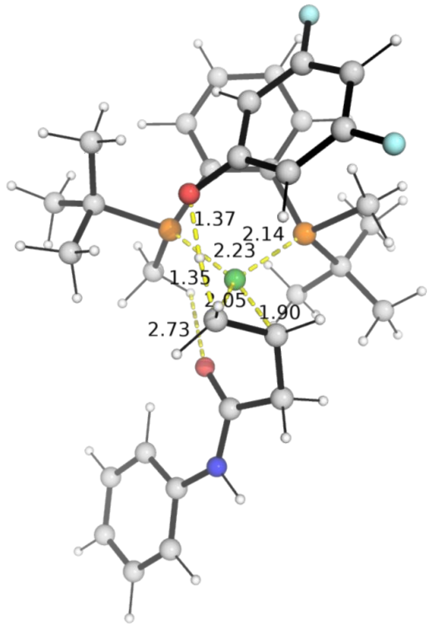	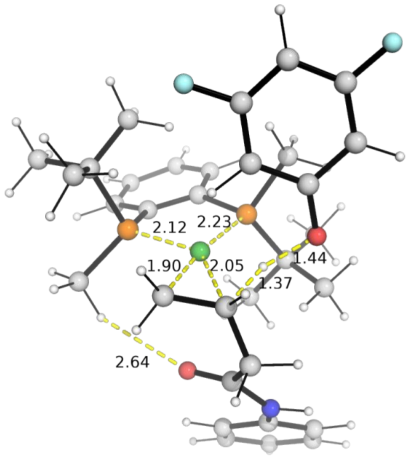

HOMO	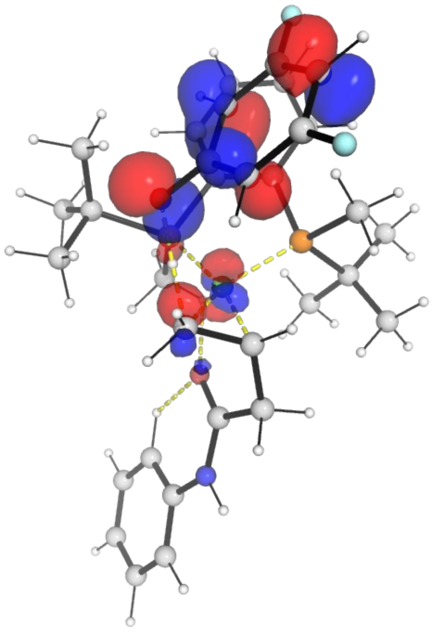	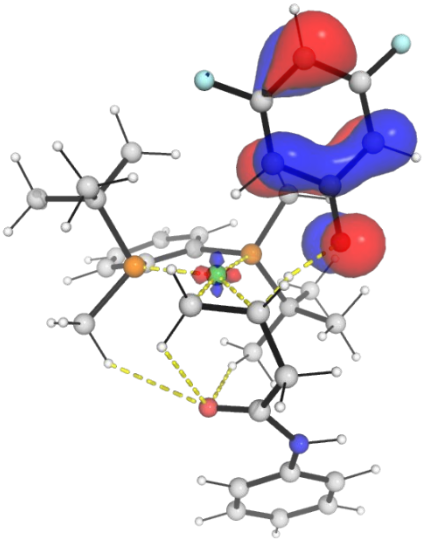
LUMO	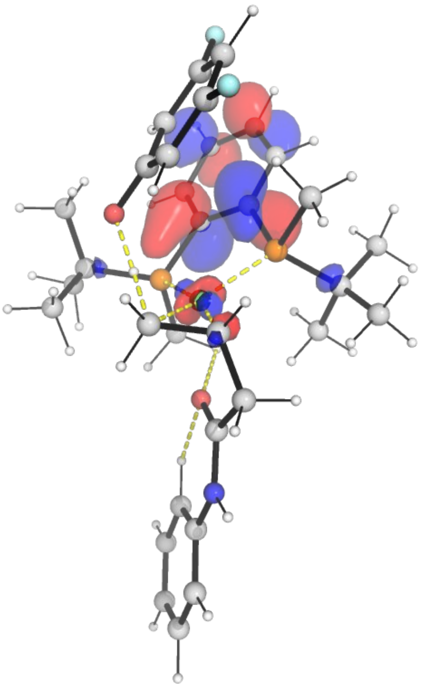	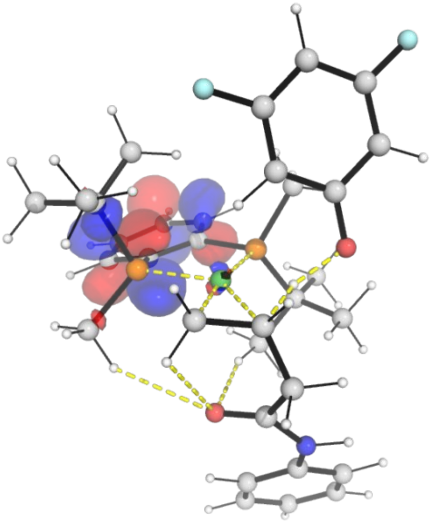

NCI

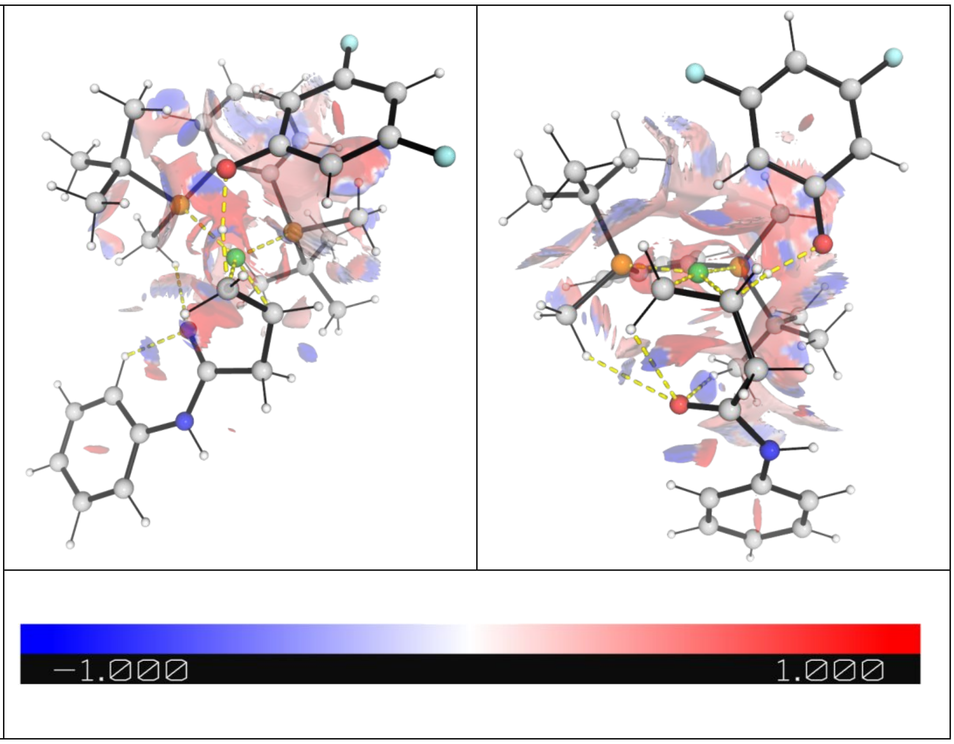

Figure S6. DFT-optimized structures, frontier molecular orbitals (HOMO and LUMO) and non-covalent interaction (NCI) plots for the lowest barrier transition state for the first C–C bond formation transition states (TSs) using 3,5-difluorophenol as the proton source.

As before, distortion-interaction[35,36] analysis is applied to key TSs to discern the factors affecting regioselectivity. The transition state structures are decomposed by dividing 3,5-difluorophenol and the olefin-bound Ni-species as components. Single point calculations at C-PCM(toluene-DMF) solvent correction were applied performed at M06-2x/def2-TZVP level of theory to obtain distortion and interaction energies. The results are given in Table S9.

Table S9. Distortion-interaction analysis for the protonation step using 3,5-difluorophenol.

Transition State	$\Delta E^{\ddagger}$	E_{dist}	E_{int}
**TS1B_Cterm**	15.2	66.1	-50.9
**TS1B_Cin**	18.1	75.7	-57.6
**TS1B'_Cterm**	19.4	72.0	-52.6
**TS1B'_Cin**	10.0	70.2	-60.2

This analysis shows the protonation of **INT1’** via **TS1B’_Cin** has the lowest barrier, and is more stable than **TS1B’_Cterm** by 9.4 kcal/mol, which results from both lower distortion energy, by 1.8 kcal/mol, and the much more stablized interaction energy, by 7.6 kcal/mol. For the protonation of **INT1**, **TS1B_Cterm** has a lower barrier by 2.9 kcal/mol than **TS1B_Cin**, due to the former having much lower distortion energy, by 9.6 kcal/mol, although the interaction energy is slightly less stable, by 6.7 kcal/mol. As before, the protonation from the approach direction of least steric hinderance (methyl group instead of tert-butyl group) is more favourable (**TS1B_Cterm** and **TS1B’_Cin**) than from the other direction (tert-butyl group instead of methyl group, **TS1B_Cin** and **TS1B’_Cterm**).

In terms of regioselectivity outcome, **TS1B’_Cin** is favoured over **TS1B_Cterm**, by $\Delta\Delta E^{\ddagger}$ = 5.2 kcal/mol, primarily due to the much better stabilization interactions, by 9.3 kcal/mol in **TS1B’_Cin** than in **TS1B_Cterm**, despite the larger distortion in **TS1B’_Cin** than in **TS1B_Cterm**, by 4.1 kcal/mol.

8.5.3 Oxidative addition of O–H of 3,5-difluorophenol to Nickel

Similarly, the possibility of oxidative addition of O–H of 3,5-difluorophenol to nickel was explored. Again, in the process of TS search and the optimization of a potential Ni–H species, it was shown that such Ni–H species could not be formed and that the H atom on Ni-center will add to olefin C=C bond upon geometry optimization, even if we started with a guess Ni–H structure.

8.5.4 Reductive elimination step

**TS2B’**	
$\Delta G^{\ddagger}$ = 5.9 [7.4]	

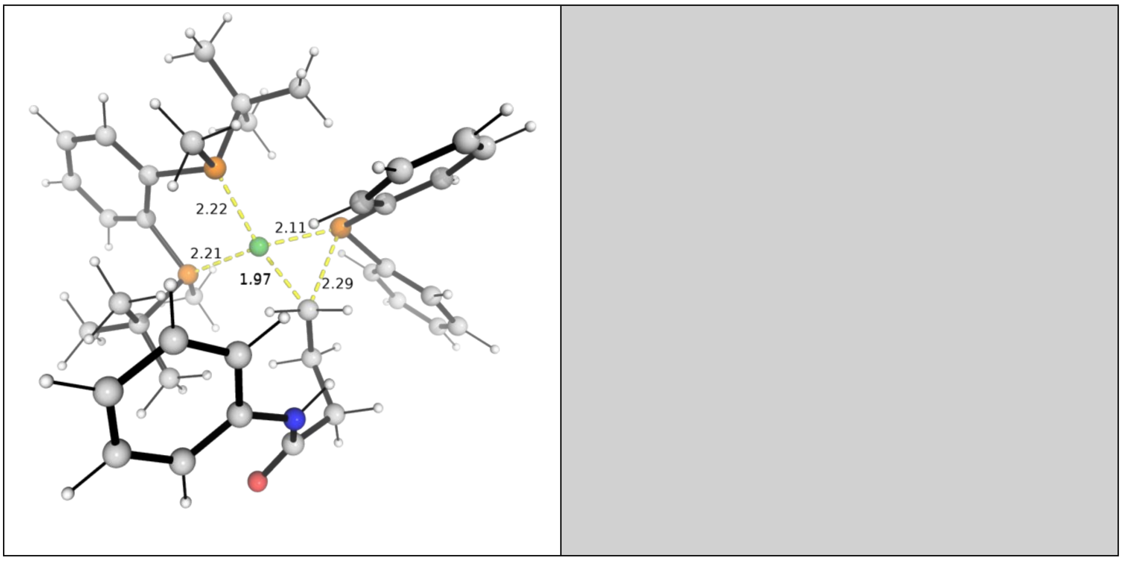

Figure S7. DFT-optimized structures of the reductive elimination step, **TS2B'**.

8.6 Estimation of product ratio under kinetic control

Under kinetic control, the product ratio of two pathways can be estimated using the ratio between the reaction rates of each pathway. The barrier difference $\Delta\Delta G^{\ddagger}$ between two transition states gives a kinetic preference for the major product over the minor product. This can be estimated using simple transition state theory, without Boltzmann weighting of all the conformers via the following:

The Eyring equation

$$k = \frac{k_{\mathrm{B}}T}{h}\mathrm{e}^{-\Delta G^{\ddagger}/RT}$$

gives the rate constant under simple transition state theory (TST) assumptions.

Under kinetic control, as we compare the barrier heights difference between competing transition states, the ratio of the rates between two pathways is given by:

$$\frac{k_A}{k_B} = \frac{e^{-\Delta G_A^{\ddagger}/RT}}{e^{-\Delta G_B^{\ddagger}/RT}} = e^{-\Delta\Delta G^{\ddagger}/RT}$$

where k_X is the rate constant of pathway X (X=A or B); $\Delta G_X^{\ddagger}$ is the activation barrier for pathway X; and $\Delta\Delta G_X^{\ddagger}$ is the difference in the barrier heights; and R is the gas constant, T the temperature. Note that the Eyring Equation pre-exponential factor

cancels when comparing the ratio of the rate constants. Thus, using the calculated $\Delta\Delta G_X^{\ddagger}$ value (difference of barrier heights between competing TSs) at the reaction temperature (e.g., 80ºC = 353.15K), we are able to obtain the ratio of competing rates.

8.7 Optimized structures and absolute energies

Geometries of all optimized structures (in *.xyz* format with their associated gas-phase energy in Hartrees) are included in a separate folder named *DFT_optimized_structures*. All these data have been uploaded to https://zenodo.org/records/15683959 (DOI: 10.5281/zenodo.15683959).

Absolute values (in Hartrees) for SCF energy, zero-point vibrational energy (ZPE), enthalpy and quasi-harmonic Gibbs free energy (at 60ºC/333.15 K) for optimized structures are given below. Single point corrections in SMD THF using B3LYP-D3BJ/def2-TZVP level of theory are also included.

Structure	E/au	ZPE/au	H/au	T.S/au	qh-G/au	SP CPCM(toluene-DMF)	SP SMD(toluene)
**substrate1**	-516.592634	0.189856	-516.38655	0.060904	-516.44473	-517.2216598	-517.2319173
**HPPh2**	-804.018129	0.19271	-803.80916	0.062175	-803.867969	-804.7180776	-804.7287202
**NiCOD2**	-2131.111086	0.364727	-2130.7223	0.072916	-2130.79436	-2132.101668	-2132.114489
**difluorophenol**	-505.014972	0.089438	-504.91475	0.046048	-504.960808	-505.6431965	-505.6481572
**sulfonic_acid**	-870.896284	0.104466	-870.77902	0.051952	-870.829978	-871.7189721	-871.7256604
**PPh2_anion_opt**	-803.445716	0.181289	-803.24856	0.05917	-803.305954	-804.182809	-804.1999164

**difluorophenol_anion_opt**	-504.453006	0.075622	-504.3669	0.045653	-504.412552	-505.12693	-505.1378214
**sulfonate_opt**	-870.373325	0.0927	-870.26865	0.051076	-870.318368	-871.248698	-871.2592342
**INT1**	-3332.448935	0.59562	-3331.8039	0.129325	-3331.925897	-3334.386218	-3334.403416
**INT1'**	-3332.43917	0.595187	-3331.7941	0.132624	-3331.918195	-3334.377001	-3334.394991
**INT1o**	-3332.401125	0.593297	-3331.7577	0.134029	-3331.882434	-3334.339269	-3334.357009
**INT2A**	-4203.375226	0.702856	-4202.6102	0.158396	-4202.757197	-4206.125307	-4206.147158
**TS1A_Cterm**	-4203.371447	0.698973	-4202.6112	0.155936	-4202.756166	-4206.115919	-4206.137342
**TS1A_Cterm_c2**	-4203.370986	0.698922	-4202.6108	0.156075	-4202.755809	-4206.115423	-4206.136908
**TS1A_Cterm_c3**	-4203.363995	0.698512	-4202.6041	0.155799	-4202.749153	-4206.114602	-4206.136972
**TS1A_Cterm_c4**	-4203.367748	0.698772	-4202.6076	0.157093	-4202.753252	-4206.113239	-4206.135016
**TS1A_Cterm_c5**	-4203.364468	0.698062	-4202.6046	0.158028	-4202.751255	-4206.109129	-4206.132025
**INT3A**	-4203.377621	0.703734	-4202.6121	0.157735	-4202.758436	-4206.137644	-4206.160905
**TS1A_Cin**	-4203.354998	0.698316	-4202.5949	0.156848	-4202.741207	-4206.102639	-4206.124812
**TS1A'_Cterm**	-4203.350197	0.698221	-4202.5903	0.157513	-4202.73687	-4206.098504	-4206.121023
**TS1A'_Cin**	-4203.367163	0.698385	-4202.6072	0.156694	-4202.752956	-4206.11161	-4206.133187

**TS1A'_Cin_c2**	-4203.365757	0.698431	-4202.6057	0.156484	-4202.751453	-4206.109454	-4206.132154
**TS1A'_Cin_c3**	-4203.362332	0.698082	-4202.6025	0.158433	-4202.749226	-4206.106621	-4206.128325
**INT4A**	-4203.39806	0.70375	-4202.6324	0.159134	-4202.779735	-4206.146428	-4206.168536
**INT5A**	-4136.489952	0.794025	-4135.6301	0.164318	-4135.783115	-4139.118573	-4139.14416
**TS2A**	-4136.459567	0.792445	-4135.6014	0.1658	-4135.755049	-4139.091769	-4139.117576
**INT6A**	-4136.497856	0.793291	-4135.6383	0.168104	-4135.793237	-4139.138406	-4139.163894
**TS1B_Cin**	-3837.442905	0.681554	-3836.7006	0.156631	-3836.84548	-3840.000637	-3840.022279
**TS1B_Cin_c2**	-3837.44235	0.681913	-3836.6999	0.15739	-3836.844993	-3839.999114	-3840.02094
**TS1B_Cin_c3**	-3837.446679	0.682033	-3836.7044	0.152954	-3836.847112	-3840.001244	-3840.02122
**TS1B_Cin_c4**	-3837.441423	0.681571	-3836.6996	0.152833	-3836.842398	-3839.996732	-3840.016707
**TS1B_Cterm**	-3837.448687	0.683028	-3836.7057	0.151342	-3836.84772	-3840.005238	-3840.026502
**TS1B'_Cterm**	-3837.443629	0.681223	-3836.702	0.155481	-3836.846011	-3839.998488	-3840.019344
**TS1B'_Cterm_c2**	-3837.444553	0.6822	-3836.7021	0.152253	-3836.84458	-3839.998042	-3840.017653
**TS1B'_Cterm_c3**	-3837.443629	0.681224	-3836.702	0.155481	-3836.84601	-3839.998488	-3840.019345
**INT2B’**	-3837.484887	0.687263	-3836.7368	0.155675	-3836.881432	-3840.041245	-3840.061804

**TS1B'_Cin**	-3837.457574	0.68236	-3836.7152	0.152167	-3836.857526	-3840.013494	-3840.034225
**TS1B'_Cin_c2**	-3837.455155	0.681874	-3836.7131	0.152971	-3836.856155	-3840.00917	-3840.029164
**TS1B'_Cin_c3**	-3837.441656	0.681612	-3836.6993	0.157921	-3836.844441	-3839.998821	-3840.02013
**INT3B'**	-3837.462239	0.686306	-3836.7154	0.154204	-3836.858999	-3840.021633	-3840.043201
**INT4B'**	-3837.482346	0.688084	-3836.734	0.153141	-3836.876798	-3840.039563	-3840.06012
**INT5B'**	-4136.490165	0.795508	-4135.6295	0.164509	-4135.781562	-4139.122521	-4139.147995
**TS2B'**	-4136.46651	0.79201	-4135.6089	0.16606	-4135.76258	-4139.103329	-4139.129338
**INT6B'**	-4136.48813	0.793364	-4135.6284	0.168984	-4135.783697	-4139.130793	-4139.15707

9. Analytic data for the products

(*R*)-3-(diphenylphosphorothioyl)-N-phenylbutanamide (3a)

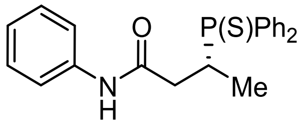

General procedure A was used with N-phenylbut-3-enamide **1a** (35.4 mg, 0.22 mmol, 2.2 equiv.) and diphenylphosphane **2a** (18.6 mg, 0.1 mmol, 1.0 equiv.) at 80 ℃ for 12 h to afford **3a** as foam (29.0 mg, 74% yield, 99% ee). ^{1}H NMR (400 MHz, Chloroform-*d*) δ 8.08-7.87 (m, 4H), 7.65 (s, 1H), 7.54-7.37 (m, 8H), 7.28-7.24 (m, 2H), 7.08 (t, *J* = 7.4 Hz, 1H), 3.62-3.49 (m, 1H), 2.70-2.44 (m, 2H), 1.17 (dd, *J* = 18.5, 6.8 Hz, 3H). ^{31}P NMR (162 MHz, Chloroform-*d*) δ 52.48. ^{13}C NMR (101 MHz, Chloroform-*d*) δ 168.95 (d, *J* = 16.0 Hz), 137.66, 131.74 (dd, *J* = 4.7, 2.9 Hz), 131.43 (dd, *J* = 9.6, 6.0 Hz), 130.65, 128.92 (d, *J* = 17.4 Hz), 128.84 (d, *J* = 23.1 Hz), 124.54, 119.85, 38.58 (d, *J* =

2.0 Hz), 30.05 (d, *J* = 58.1 Hz), 13.58. $[\alpha]^{20}_{D}$= -57.2 (c 0.86, $CHCl_3$). The enantiomeric excess was determined by Daicel Chiralcel IF (0.46 cm x 25 cm), Hexanes /IPA = 85 / 15, 1.0 mL/min, λ = 254 nm, t (major) = 8.8 min, t (minor) = 10.0 min. HRMS (ESI-ion trap) m/z: [M+H]+ calcd for $C_{22}H_{23}NOPS$ 380.1238; found 380.1233.

(*R*)-3-(diphenylphosphorothioyl)-N-(o-tolyl)butanamide (3b)

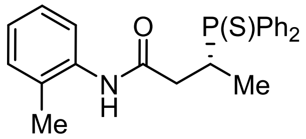

General procedure A was used with N-(o-tolyl)but-3-enamide **1b** (38.5mg, 0.22 mmol, 2.2 equiv.) and diphenylphosphane **2a** (18.6 mg, 0.1 mmol, 1.0 equiv.) at 80 ℃ for 12 h to afford **3b** as colorless oil (29.0 mg, 74% yield, 97% ee). ^{1}H NMR (600 MHz, Chloroform-*d*) δ 8.06-7.89 (m, 4H), 7.67 (d, *J* = 8.1 Hz, 1H), 7.50-7.42 (m, 6H), 7.19 (s, 1H), 7.17-7.12 (m, 2H), 7.05 (t, *J* = 7.5 Hz, 1H), 3.64-3.55 (m, 1H), 2.71-2.63 (m, 1H), 2.58-2.48 (m, 1H), 2.20 (s, 3H), 1.20 (dd, *J* = 18.5, 6.8 Hz, 3H). ^{31}P NMR (243 MHz, Chloroform-*d*) δ 52.50. ^{13}C NMR (151 MHz, Chloroform-*d*) δ 168.92 (d, *J* = 16.5 Hz), 135.47, 131.74 (dd, *J* = 14.0, 2.9 Hz), 131.45 (d, *J* = 9.7 Hz), 130.92 (d, *J* = 7.3 Hz), 130.58, 129.10, 128.84 (dd, *J* = 24.0, 11.8 Hz), 126.74, 125.44, 123.03, 38.37, 30.00 (d, *J* = 58.3 Hz), 17.98, 13.50. $[\alpha]^{20}_{D}$= -43.7 (c 0.9, $CHCl_3$). The enantiomeric excess was determined by Daicel Chiralcel IF (0.46 cm x 25 cm), Hexanes /IPA = 85 / 15, 1.0 mL/min, λ = 254 nm, t (minor) = 12.4 min, t (major) = 14.5 min. HRMS (ESI-ion trap) m/z: [M+H]+ calcd for $C_{23}H_{25}NOPS$ 394.1394; found 394.1391.

(*R*)-3-(diphenylphosphorothioyl)-N-(m-tolyl)butanamide (3c)

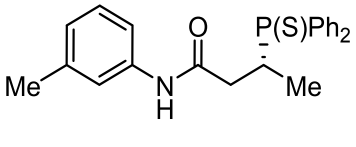

General procedure A was used with N-(m-tolyl)but-3-enamide **1c** (38.5mg, 0.22 mmol, 2.2 equiv.) and diphenylphosphane **2a** (18.6 mg, 0.1 mmol, 1.0 equiv.) at 80 ℃ for 12 h to afford **3c** as colorless oil (27.4 mg, 70% yield, 98% ee). ^{1}H NMR (600 MHz,

Chloroform-*d*) δ 8.03-7.87 (m, 4H), 7.59 (s, 1H), 7.49-7.37 (m, 6H), 7.25 (d, *J* = 8.8 Hz, 1H), 7.18 (d, *J* = 8.2 Hz, 1H), 7.13 (t, *J* = 7.7 Hz, 1H), 6.88 (d, *J* = 7.4 Hz, 1H), 3.61-3.51 (m, 1H), 2.64-2.56 (m, 1H), 2.53-2.45 (m, 1H), 2.28 (s, 3H), 1.16 (dd, *J* = 18.5, 6.7 Hz, 3H). ^{31}P NMR (243 MHz, Chloroform-*d*) δ 52.47. ^{13}C NMR (151 MHz, Chloroform-*d*) δ 168.95 (d, *J* = 16.3 Hz), 138.91, 137.57, 131.72 (dd, *J* = 7.3, 3.0 Hz), 131.43 (dd, *J* = 9.7, 6.3 Hz), 130.86, 129.90-127.38 (m), 125.35, 120.54, 116.99, 38.57 (d, *J* = 2.0 Hz), 30.06 (d, *J* = 58.2 Hz), 21.57, 13.54. $[\alpha]^{20}_{D}$= -54.4 (c 0.85, $CHCl_3$). The enantiomeric excess was determined by Daicel Chiralcel IF (0.46 cm x 25 cm), Hexanes /IPA = 85 / 15, 1.0 mL/min, λ = 254 nm, t (minor) = 7.4 min, t (major) = 8.9 min. HRMS (ESI-ion trap) m/z: [M+H]+ calcd for $C_{23}H_{25}NOPS$ 394.1394; found 394.1391.

(*R*)-3-(diphenylphosphorothioyl)-N-(p-tolyl)butanamide (3d)

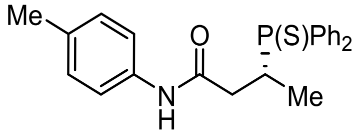

General procedure A was used with N-(p-tolyl)but-3-enamide **1d** (38.5mg, 0.22 mmol, 2.2 equiv.) and diphenylphosphane **2a** (18.6 mg, 0.1 mmol, 1.0 equiv.) at 80 ℃ for 12 h to afford **3d** as foam (30.5 mg, 78% yield, 95% ee). ^{1}H NMR (400 MHz, Chloroform-*d*) δ 8.04-7.89 (m, 4H), 7.59-7.37 (m, 7H), 7.34-7.27 (m, 2H), 7.11-7.03 (m, 2H), 3.63-3.51 (m, 1H), 2.66-2.55 (m, 1H), 2.54-2.42 (m, 1H), 2.29 (s, 3H), 1.17 (dd, *J* = 18.5, 6.8 Hz, 3H). ^{31}P NMR (162 MHz, Chloroform-*d*) δ 52.50. ^{13}C NMR (101 MHz, Chloroform-*d*) δ 168.80 (d, *J* = 16.3 Hz), 135.09, 134.20, 131.73 (dd, *J* = 6.6, 2.9 Hz), 131.44 (dd, *J* = 9.6, 3.5 Hz), 130.72, 129.50, 128.83 (t, *J* = 12.3 Hz), 119.96, 38.46 (d, *J* = 2.0 Hz), 30.04 (d, *J* = 58.0 Hz), 20.99, 13.51. $[\alpha]^{20}_{D}$= -43.6 (c 0.95, $CHCl_3$). The enantiomeric excess was determined by Daicel Chiralcel IF (0.46 cm x 25 cm), Hexanes /IPA = 85 / 15, 1.0 mL/min, λ = 254 nm, t (minor) = 7.7 min, t (major) = 8.4 min. HRMS (ESI-ion trap) m/z: [M+H]+ calcd for $C_{23}H_{25}NOPS$ 394.1394; found 394.1390.

(*R*)-N-(4-(tert-butyl)phenyl)-3-(diphenylphosphorothioyl)butanamide (3e)

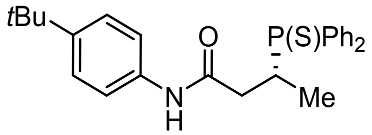

General procedure A was used with N-(4-(tert-butyl)phenyl)but-3-enamide **1e** (47.7mg, 0.22 mmol, 2.2 equiv.) and diphenylphosphane **2a** (18.6 mg, 0.1 mmol, 1.0 equiv.) at 80 °C for 12 h to afford **3e** as foam (27.7 mg, 64% yield, >99% ee). ^{1}H NMR (600 MHz, Chloroform-*d*) δ 8.00-7.89 (m, 4H), 7.58 (s, 1H), 7.48-7.35 (m, 6H), 7.34-7.30 (m, 2H), 7.28-7.24 (m, 2H), 3.57-3.51 (m, 1H), 2.63-2.54 (m, 1H), 2.51-2.44 (m, 1H), 1.25 (d, *J* = 1.7 Hz, 9H), 1.14 (dd, *J* = 18.6, 6.8 Hz, 3H). ^{31}P NMR (243 MHz, Chloroform-*d*) δ 52.52. ^{13}C NMR (151 MHz, Chloroform-*d*) δ 168.85 (d, *J* = 16.3 Hz), 147.56, 135.07, 38.50, 34.47, 31.46, 30.11 (d, *J* = 57.8 Hz), 13.49. $[\alpha]^{20}_{D}$= -51.5 (c 0.86, $CHCl_3$). The enantiomeric excess was determined by Daicel Chiralcel IF (0.46 cm x 25 cm), Hexanes /IPA = 85 / 15, 1.0 mL/min, λ = 254 nm, t (minor) = 8.7 min, t (major) = 9.8 min. HRMS (ESI-ion trap) m/z: [M+H]+ calcd for $C_{26}H_{31}NOPS$ 436.1864; found 436.1861.

(*R*)-N-(4-benzylphenyl)-3-(diphenylphosphorothioyl)butanamide (3f)

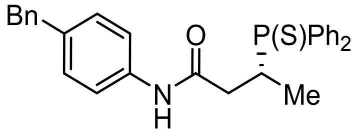

General procedure A was used with N-(4-benzylphenyl)but-3-enamide **1f** (55.2mg, 0.22 mmol, 2.2 equiv.) and diphenylphosphane **2a** (18.6 mg, 0.1 mmol, 1.0 equiv.) at 80 °C for 12 h to afford **3f** as foam (35.8 mg, 76% yield, 99% ee). ^{1}H NMR (600 MHz, Chloroform-*d*) δ 8.05-7.89 (m, 4H), 7.66 (s, 1H), 7.50-7.42 (m, 3H), 7.42-7.36 (m, 3H), 7.34 (d, *J* = 8.0 Hz, 2H), 7.29-7.25 (m, 2H), 7.19 (t, *J* = 7.4 Hz, 1H), 7.15 (d, *J* = 7.6 Hz, 2H), 7.08 (d, *J* = 8.1 Hz, 2H), 3.92 (s, 2H), 3.61-3.51 (m, 1H), 2.70-2.56 (m, 1H), 2.54-2.46 (m, 1H), 1.16 (dd, *J* = 18.5, 6.8 Hz, 3H). ^{31}P NMR (243 MHz, Chloroform-*d*) δ 52.50. ^{13}C NMR (151 MHz, Chloroform-*d*) δ 168.87 (d, *J* = 16.2 Hz), 141.19, 137.41, 135.76, 131.71 (dd, *J* = 7.9, 2.9 Hz), 131.42 (t, *J* = 9.7 Hz), 131.32, 130.82 (d, *J* = 3.3 Hz), 129.44, 128.94, 128.81 (dd, *J* = 17.4, 11.9 Hz), 128.57, 126.20, 120.07, 41.43, 38.52, 30.07 (d, *J* = 58.1 Hz), 13.55. $[\alpha]^{20}_{D}$= -43.7 (c 1.12, $CHCl_3$). The

enantiomeric excess was determined by Daicel Chiralcel IF (0.46 cm x 25 cm), Hexanes /IPA = 85 / 15, 1.0 mL/min, λ = 254 nm, t (minor) = 9.8 min, t (major) = 12.0 min. HRMS (ESI-ion trap) m/z: [M+H]+ calcd for $C_{29}H_{29}NOPS$ 470.1707; found 470.1707.

(*R*)-3-(diphenylphosphorothioyl)-N-(4-(trifluoromethyl)phenyl)butanamide (3g)

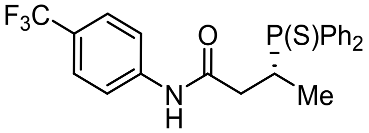

General procedure A was used with N-(4-(trifluoromethyl)phenyl)but-3-enamide **1g** (50.4mg, 0.22 mmol, 2.2 equiv.) and diphenylphosphane **2a** (18.6 mg, 0.1 mmol, 1.0 equiv.) at 80 °C for 12 h to afford **3g** as foam (29.6 mg, 66% yield, >99% ee). ^{1}H NMR (600 MHz, Chloroform-*d*) δ 8.01 (s, 1H), 8.00-7.89 (m, 4H), 7.56-7.43 (m, 7H), 7.43-7.33 (m, 3H), 3.60-3.52 (m, 1H), 2.73-2.67 (m, 1H), 2.62-2.55 (m, 1H), 1.17 (dd, *J* = 18.4, 6.8 Hz, 3H). ^{31}P NMR (243 MHz, Chloroform-*d*) δ 52.43. ^{19}F NMR (565 MHz, Chloroform-*d*) δ -62.12. ^{13}C NMR (151 MHz, Chloroform-*d*) δ 169.31 (d, *J* = 14.4 Hz), 140.69, 131.84 (t, *J* = 3.3 Hz), 131.47 (d, *J* = 9.8 Hz), 131.32 (d, *J* = 9.6 Hz), 130.88 (dd, *J* = 78.3, 7.5 Hz), 128.87 (dd, *J* = 11.8, 7.2 Hz), 126.19 (q, *J* = 3.8 Hz), 124.16 (q, *J* = 271.5 Hz), 119.35, 38.99, 30.11 (d, *J* = 57.7 Hz), 13.93. $[\alpha]^{20}_{D}$= -44.3 (c 0.92, $CHCl_3$). The enantiomeric excess was determined by Daicel Chiralcel IF (0.46 cm x 25 cm), Hexanes /IPA = 85 / 15, 1.0 mL/min, λ = 254 nm, t (minor) = 6.4 min, t (major) = 7.2 min. HRMS (ESI-ion trap) m/z: [M+H]+ calcd for $C_{23}H_{22}F_3NOPS$ 448.1112; found 448.1111.

(*R*)-3-(diphenylphosphorothioyl)-N-(4-fluorophenyl)butanamide (3h)

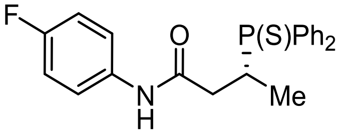

General procedure A was used with N-(4-fluorophenyl)but-3-enamide **1h** (39.4mg, 0.22 mmol, 2.2 equiv.) and diphenylphosphane **2a** (18.6 mg, 0.1 mmol, 1.0 equiv.) at 80 °C for 12 h to afford **3h** as foam (26.8 mg, 68% yield, 98% ee). ^{1}H NMR (600 MHz,

Chloroform-*d*) δ 8.04-7.89 (m, 4H), 7.60-7.53 (m, 1H), 7.52-7.44 (m, 3H), 7.44-7.37 (m, 3H), 7.37-7.31 (m, 2H), 6.95 (t, *J* = 8.4 Hz, 2H), 3.59-3.52 (m, 1H), 2.70-2.62 (m, 1H), 2.54-2.46 (m, 1H), 1.17 (dd, *J* = 18.4, 6.8 Hz, 3H). ^{31}P NMR (243 MHz, Chloroform-*d*) δ 52.45. ^{19}F NMR (565 MHz, Chloroform-*d*) δ -117.80. ^{13}C NMR (151 MHz, Chloroform-*d*) δ 168.82 (d, *J* = 14.9 Hz), 159.51 (d, *J* = 243.7 Hz), 133.65 (d, *J* = 2.7 Hz), 131.78 (t, *J* = 2.6 Hz), 131.43 (dd, *J* = 12.3, 9.6 Hz), 130.80 (d, *J* = 2.6 Hz), 128.86 (t, *J* = 11.6 Hz), 121.58 (d, *J* = 7.8 Hz), 115.62 (d, *J* = 22.5 Hz), 38.72, 30.09 (d, *J* = 57.7 Hz), 13.81. $[\alpha]^{20}_D$= -29.3 (c 0.82, $CHCl_3$). The enantiomeric excess was determined by Daicel Chiralcel IF (0.46 cm x 25 cm), Hexanes /IPA = 85 / 15, 1.0 mL/min, λ = 254 nm, t (minor) = 7.8 min, t (major) = 8.8 min. HRMS (ESI-ion trap) m/z: [M+H]+ calcd for $C_{22}H_{22}FNOPS$ 398.1144; found 398.1144.

(*R*)-N-(4-chlorophenyl)-3-(diphenylphosphorothioyl)butanamide (3i)

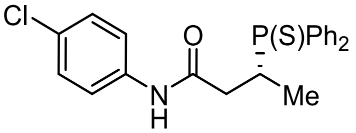

General procedure A was used with N-(4-chlorophenyl)but-3-enamide **1i** (42.9 mg, 0.22 mmol, 2.2 equiv.) and diphenylphosphane **2a** (18.6 mg, 0.1 mmol, 1.0 equiv.) at 80 °C for 12 h to afford **3i** as foam (27.3 mg, 66% yield, 97% ee). ^{1}H NMR (600 MHz, Chloroform-*d*) δ 8.02-7.88 (m, 4H), 7.83 (s, 1H), 7.52-7.43 (m, 3H), 7.43-7.32 (m, 5H), 7.23-7.17 (m, 2H), 3.59-3.51 (m, 1H), 2.69-2.61 (m, 1H), 2.57-2.49 (m, 1H), 1.16 (dd, *J* = 18.5, 6.8 Hz, 3H). ^{31}P NMR (243 MHz, Chloroform-*d*) δ 52.47. ^{13}C NMR (151 MHz, Chloroform-*d*) δ 169.01 (d, *J* = 14.8 Hz), 136.25, 131.80 (t, *J* = 3.3 Hz), 131.41 (dd, *J* = 14.5, 9.6 Hz), 130.97 (dd, *J* = 78.1, 4.5 Hz), 129.43, 128.95, 128.85 (t, *J* = 11.4 Hz), 121.06, 38.74, 30.08 (d, *J* = 57.8 Hz), 13.80. $[\alpha]^{20}_D$= -62.6 (c 0.84, $CHCl_3$). The enantiomeric excess was determined by Daicel Chiralcel IF (0.46 cm x 25 cm), Hexanes /IPA = 85 / 15, 1.0 mL/min, λ = 254 nm, t (minor) = 8.5 min, t (major) = 9.9 min. HRMS (ESI-ion trap) m/z: [M+H]+ calcd for $C_{22}H_{22}ClNOPS$ 414.0848; found 414.0848.

(*R*)-N-(4-bromophenyl)-3-(diphenylphosphorothioyl)butanamide (3j)

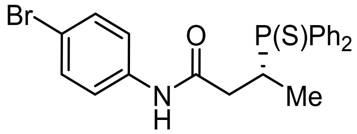

General procedure A was used with N-(4-bromophenyl)but-3-enamide **1j** (52.8 mg, 0.22 mmol, 2.2 equiv.) and diphenylphosphane **2a** (18.6 mg, 0.1 mmol, 1.0 equiv.) at 80 °C for 12 h to afford **3j** as foam (30.0 mg, 66% yield, 97% ee). ^{1}H NMR (600 MHz, Chloroform-*d*) δ 8.02-7.87 (m, 4H), 7.65 (s, 1H), 7.51-7.44 (m, 3H), 7.43-7.33 (m, 5H), 7.32-7.27 (m, 2H), 3.59-3.51 (m, 1H), 2.69-2.62 (m, 1H), 2.55-2.47 (m, 1H), 1.16 (dd, *J* = 18.4, 6.8 Hz, 3H). ^{31}P NMR (243 MHz, Chloroform-*d*) δ 52.42. ^{13}C NMR (151 MHz, Chloroform-*d*) δ 168.93 (d, *J* = 14.5 Hz), 136.72, 131.93, 131.81 (t, *J* = 3.5 Hz), 131.42 (dd, *J* = 15.9, 9.7 Hz), 131.00 (d, *J* = 79.2 Hz), 128.86 (t, *J* = 11.4 Hz), 121.32, 117.07, 38.88, 30.10 (d, *J* = 57.7 Hz), 13.86. $[\alpha]^{20}_{D}$= -28.3 (c 0.93, $CHCl_3$). The enantiomeric excess was determined by Daicel Chiralcel IF (0.46 cm x 25 cm), Hexanes /IPA = 85 / 15, 1.0 mL/min, λ = 254 nm, t (minor) = 9.6 min, t (major) = 11.6 min. HRMS (ESI-ion trap) m/z: [M+H]+ calcd for $C_{22}H_{22}BrNOPS$ 458.0343; found 458.0343.

(*R*)-3-(diphenylphosphorothioyl)-N-(4-methoxyphenyl)butanamide (3k)

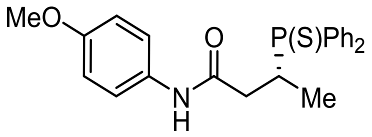

General procedure A was used with N-(4-methoxyphenyl)but-3-enamide **1k** (42.0 mg, 0.22 mmol, 2.2 equiv.) and diphenylphosphane **2a** (18.6 mg, 0.1 mmol, 1.0 equiv.) at 80 °C for 12 h to afford **3k** as foam (26.5 mg, 65% yield, >99% ee). ^{1}H NMR (600 MHz, Chloroform-*d*) δ 8.03-7.90 (m, 4H), 7.51-7.39 (m, 7H), 7.33-7.27 (m, 2H), 6.83-6.77 (m, 2H), 3.77 (s, 3H), 3.59-3.53 (m, 1H), 2.65-2.58 (m, 1H), 2.51-2.44 (m, 1H), 1.17 (dd, *J* = 18.5, 6.8 Hz, 3H). ^{31}P NMR (243 MHz, Chloroform-*d*) δ 52.50. ^{13}C NMR (151 MHz, Chloroform-*d*) δ 168.68 (dd, *J* = 16.0, 3.8 Hz), 156.60, 131.72 (dd, *J* = 8.1, 2.9 Hz), 131.46 (dd, *J* = 9.5, 5.2 Hz), 131.43, 130.85 (d, *J* = 15.2 Hz), 128.83 (dd, *J* = 17.6,

11.8 Hz), 121.72, 114.15, 55.60, 38.44, 30.08 (d, *J* = 58.1 Hz), 13.59. $[\alpha]^{20}_D$= -42.6 (c 0.82, $CHCl_3$). The enantiomeric excess was determined by Daicel Chiralcel IF (0.46 cm x 25 cm), Hexanes /IPA = 85 / 15, 1.0 mL/min, λ = 254 nm, t (minor) = 15.2 min, t (major) = 18.2 min. HRMS (ESI-ion trap) m/z: [M+H]+ calcd for $C_{23}H_{25}NO_2PS$ - 410.1344; found 410.1341.

(*R*)-3-(diphenylphosphorothioyl)-N-(4-phenoxyphenyl)butanamide (3l)

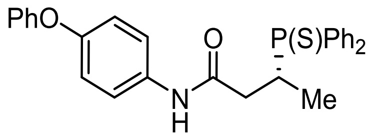

General procedure A was used with N-(4-phenoxyphenyl)but-3-enamide **1l** (55.7 mg, 0.22 mmol, 2.2 equiv.) and diphenylphosphane **2a** (18.6 mg, 0.1 mmol, 1.0 equiv.) at 80 ℃ for 12 h to afford **3l** as foam (35.4 mg, 75% yield, 98% ee). ^{1}H NMR (400 MHz, Chloroform-*d*) δ 8.05-7.89 (m, 4H), 7.77 (s, 1H), 7.51-7.34 (m, 8H), 7.31 (t, *J* = 7.9 Hz, 2H), 7.07 (t, *J* = 7.4 Hz, 1H), 6.99-6.87 (m, 4H), 3.64-3.50 (m, 1H), 2.71-2.59 (m, 1H), 2.59-2.47 (m, 1H), 1.17 (dd, *J* = 18.5, 6.8 Hz, 3H). ^{31}P NMR (162 MHz, Chloroform-*d*) δ 52.50. ^{13}C NMR (101 MHz, Chloroform-*d*) δ 168.89 (d, *J* = 15.6 Hz), 157.62, 153.58, 133.21, 131.75 (t, *J* = 3.3 Hz), 131.42 (dd, *J* = 9.7, 5.5 Hz), 130.66 (d, *J* = 3.8 Hz), 129.83, 129.27-128.28 (m), 123.19, 121.59, 119.61, 118.48, 38.51 (d, *J* = 1.9 Hz), 30.07 (d, *J* = 57.8 Hz), 13.65. $[\alpha]^{20}_D$= -46.7 (c 1.11, $CHCl_3$). The enantiomeric excess was determined by Daicel Chiralcel IF (0.46 cm x 25 cm), Hexanes /IPA = 85 / 15, 1.0 mL/min, λ = 254 nm, t (minor) = 8.7 min, t (major) = 11.0 min. HRMS (ESI-ion trap) m/z: [M+H]+ calcd for $C_{28}H_{27}NO_2PS$ 472.1500; found 472.1500.

(*R*)-3-(diphenylphosphorothioyl)-N-(4-(trifluoromethoxy)phenyl)butanamide (3m)

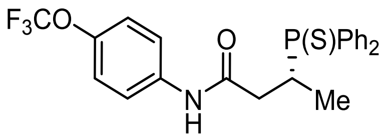

General procedure A was used with N-(4-(trifluoromethoxy)phenyl)but-3-enamide **1m** (53.9 mg, 0.22 mmol, 2.2 equiv.) and diphenylphosphane **2a** (18.6 mg, 0.1 mmol, 1.0 equiv.) at 80 ℃ for 12 h to afford **3m** as foam (20.9 mg, 45% yield, 98% ee). ^{1}H NMR (600 MHz, Chloroform-*d*) δ 8.01-7.87 (m, 5H), 7.50-7.41 (m, 5H), 7.40-7.34 (m, 3H), 7.09 (d, *J* = 8.5 Hz, 2H), 3.59-3.52 (m, 1H), 2.72-2.65 (m, 1H), 2.59-2.51 (m, 1H), 1.16 (dd, *J* = 18.4, 6.8 Hz, 3H). ^{19}F NMR (565 MHz, Chloroform-*d*) δ -58.16. ^{31}P NMR (243 MHz, Chloroform-*d*) δ 52.46. ^{13}C NMR (151 MHz, Chloroform-*d*) δ 169.07 (d, *J* = 14.3 Hz), 145.38, 136.37, 131.80 (t, *J* = 2.6 Hz), 131.40 (dd, *J* = 19.4, 9.6 Hz), 131.20 (d, *J* = 2.3 Hz), 130.68, 128.85 (dd, *J* = 11.8, 7.8 Hz), 121.68, 120.89, 38.85, 30.13 (d, *J* = 57.5 Hz), 13.89. $[\alpha]^{20}_D$= 45.7 (c 0.63, $CHCl_3$). The enantiomeric excess was determined by Daicel Chiralcel IF (0.46 cm x 25 cm), Hexanes /IPA = 85 / 15, 1.0 mL/min, λ = 254 nm, t (major) = 6.2 min, t (minor) = 7.0 min. HRMS (ESI-ion trap) m/z: [M+H]+ calcd for $C_{23}H_{22}F_3NO_2PS$ 464.1061; found 464.1060.

(*R*)-3-(diphenylphosphorothioyl)-N-(4-vinylphenyl)butanamide (3n)

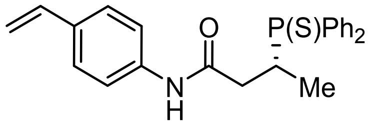

General procedure A was used with N-(4-vinylphenyl)but-3-enamide **1n** (41.1 mg, 0.22 mmol, 2.2 equiv.) and diphenylphosphane **2a** (18.6 mg, 0.1 mmol, 1.0 equiv.) at 80 ℃ for 12 h to afford **3n** as foam (21.5 mg, 53% yield, 99% ee). ^{1}H NMR (600 MHz, Chloroform-*d*) δ 8.01-7.90 (m, 4H), 7.65 (s, 1H), 7.52-7.44 (m, 3H), 7.44-7.35 (m, 5H), 7.31 (d, *J* = 8.2 Hz, 2H), 6.64 (dd, *J* = 17.6, 10.8 Hz, 1H), 5.66 (d, *J* = 17.6 Hz, 1H), 5.18 (d, *J* = 10.9 Hz, 1H), 3.60-3.53 (m, 1H), 2.67-2.60 (m, 1H), 2.55-2.47 (m, 1H), 1.17 (dd, *J* = 18.5, 6.8 Hz, 3H). ^{31}P NMR (243 MHz, Chloroform-*d*) δ 52.47. ^{13}C NMR (151 MHz, Chloroform-*d*) δ 168.86 (d, *J* = 15.8 Hz), 137.22, 136.20, 133.95, 131.76 (dd, *J* = 8.6, 3.0 Hz), 131.44 (t, *J* = 9.8 Hz), 131.06 (d, *J* = 78.1 Hz), 128.85 (dd, *J* = 15.8, 11.9 Hz), 126.83, 119.79, 113.21, 38.68, 30.09 (d, *J* = 58.2 Hz), 13.66. $[\alpha]^{20}_D$= -78.2 (c 0.65, $CHCl_3$). The enantiomeric excess was determined by Daicel Chiralcel IF (0.46 cm x 25 cm), Hexanes /IPA = 85 / 15, 1.0 mL/min, λ = 254 nm, t (minor) = 10.6

min, t (major) = 12.5 min. HRMS (ESI-ion trap) m/z: [M+H]+ calcd for $C_{24}H_{25}NOPS$ 406.1394; found 406.1390.

(*R*)-N-(3,5-difluorophenyl)-3-(diphenylphosphorothioyl)butanamide (3o)

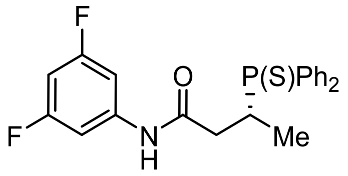

General procedure A was used with N-(3,5-difluorophenyl)but-3-enamide **1o** (43.3 mg, 0.22 mmol, 2.2 equiv.) and diphenylphosphane **2a** (18.6 mg, 0.1 mmol, 1.0 equiv.) at 80 °C for 12 h to afford **3o** as foam (27.1 mg, 65% yield, 99% ee). ^{1}H NMR (600 MHz, Chloroform-*d*) δ 8.10 (s, 1H), 8.01-7.87 (m, 4H), 7.51-7.43 (m, 3H), 7.42-7.35 (m, 3H), 7.02 (d, *J* = 8.3 Hz, 2H), 6.54-6.46 (m, 1H), 3.58-3.51 (m, 1H), 2.71-2.65 (m, 1H), 2.60-2.53 (m, 1H), 1.15 (dd, *J* = 18.4, 6.8 Hz, 3H). ^{31}P NMR (243 MHz, Chloroform-*d*) δ 52.43. ^{19}F NMR (565 MHz, Chloroform-*d*) δ -109.01. ^{13}C NMR (151 MHz, Chloroform-*d*) δ 169.27 (d, *J* = 14.1 Hz), 163.08 (dd, *J* = 246.3, 14.4 Hz), 139.77 (t, *J* = 13.3 Hz), 131.86 (t, *J* = 3.5 Hz), 131.38 (dd, *J* = 19.3, 9.6 Hz), 130.82 (dd, *J* = 78.3, 12.3 Hz), 128.89 (dd, *J* = 11.8, 4.9 Hz), 102.73 (dd, *J* = 23.4 Hz, 6.7 Hz), 99.59 (t, *J* = 25.6 Hz), 38.97, 30.09 (d, *J* = 57.6 Hz), 13.96. $[\alpha]^{20}_{D}$= -57.8 (c 0.84, $CHCl_3$). The enantiomeric excess was determined by Daicel Chiralcel IF (0.46 cm x 25 cm), Hexanes /IPA = 85 / 15, 1.0 mL/min, λ = 254 nm, t (minor) = 4.5 min, t (major) = 5.0 min. HRMS (ESI-ion trap) m/z: [M+H]+ calcd for $C_{22}H_{21}F_2NOPS$ 416.1050; found 416.1046.

(*R*)-3-(diphenylphosphorothioyl)-N-(4-morpholinophenyl)butanamide (3p)

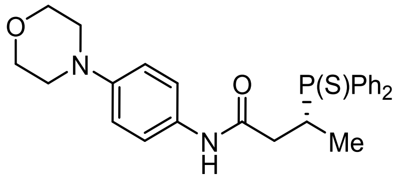

General procedure A was used with **1p** (54.1 mg, 0.22 mmol, 2.2 equiv.) and diphenylphosphane **2a** (18.6 mg, 0.1 mmol, 1.0 equiv.) at 80 °C for 12 h to afford **3p** as

foam (23.1 mg, 50% yield, 94% ee). ^{1}H NMR (600 MHz, Chloroform-*d*) δ 8.04-7.90 (m, 4H), 7.53-7.39 (m, 7H), 7.33-7.28 (m, 2H), 6.86-6.79 (m, 2H), 3.89-3.79 (m, 4H), 3.60-3.53 (m, 1H), 3.14-3.02 (m, 4H), 2.64-2.54 (m, 1H), 2.52-2.40 (m, 1H), 1.17 (dd, *J* = 18.5, 6.8 Hz, 3H). ^{31}P NMR (243 MHz, Chloroform-*d*) δ 52.51. ^{13}C NMR (151 MHz, Chloroform-*d*) δ 168.64 (d, *J* = 16.2 Hz), 148.45, 131.71 (dd, *J* = 7.5, 3.0 Hz), 131.45 (dd, *J* = 9.7, 3.9 Hz), 130.68 (d, *J* = 67.8 Hz), 128.83 (dd, *J* = 18.8, 11.8 Hz), 121.34, 116.28, 66.98, 49.84, 38.38, 30.07 (d, *J* = 58.1 Hz), 13.52. $[\alpha]^{20}_D$= -29.4 (c 0.70, $CHCl_3$). The enantiomeric excess was determined by Daicel Chiralcel OD-H (0.46 cm x 25 cm), Hexanes /IPA = 85 / 15, 1.0 mL/min, λ = 254 nm, t (major) = min, t (minor) = min. HRMS (ESI-ion trap) m/z: [M+H]+ calcd for $C_{26}H_{30}N_2O_2PS$ 465.1766; found 465.1764.

(*R*)-N-(4-acetylphenyl)-3-(diphenylphosphorothioyl)butanamide (3q)

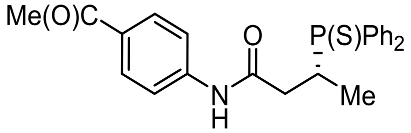

General procedure A was used with N-(4-acetylphenyl)but-3-enamide **1q** (44.7 mg, 0.22 mmol, 2.2 equiv.) and diphenylphosphane **2a** (18.6 mg, 0.1 mmol, 1.0 equiv.) at 80 °C for 12 h to afford **3q** as foam (26.4 mg, 62% yield, >99% ee). ^{1}H NMR (400 MHz, Chloroform-*d*) δ 8.20 (s, 1H), 8.02-7.89 (m, 4H), 7.89-7.83 (m, 2H), 7.54 (d, *J* = 8.5 Hz, 2H), 7.51-7.42 (m, 3H), 7.42-7.33 (m, 3H), 3.61-3.50 (m, 1H), 2.76-2.62 (m, 1H), 2.61-2.52 (m, 1H), 2.56 (s, 3H), 1.17 (dd, *J* = 18.5, 6.8 Hz, 3H). ^{31}P NMR (162 MHz, Chloroform-*d*) δ 52.41. ^{13}C NMR (101 MHz, Chloroform-*d*) δ 197.24, 169.40 (d, *J* = 15.2 Hz), 142.11, 132.97, 131.82 (d, *J* = 2.9 Hz), 131.39 (dd, *J* = 11.4, 9.6 Hz), 131.22, 130.49 (d, *J* = 8.9 Hz), 129.71, 128.85 (dd, *J* = 11.9, 6.2 Hz), 118.96, 38.84, 30.07 (d, *J* = 57.7 Hz), 26.61, 13.82. $[\alpha]^{20}_D$= -66.6 (c 0.81, $CHCl_3$). The enantiomeric excess was determined by Daicel Chiralcel IF (0.46 cm x 25 cm), Hexanes /IPA = 85 / 15, 1.0 mL/min, λ = 254 nm, t (minor) = 16.4 min, t (major) = 19.2 min. HRMS (ESI-ion trap) m/z: [M+H]+ calcd for C24H24NO2PS 422.1344; found 422.1341.

methyl (*R*)-4-(3-(diphenylphosphorothioyl)butanamido)benzoate (3r)

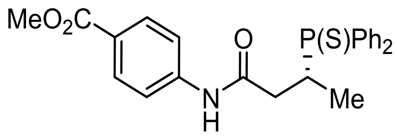

General procedure A was used with methyl 4-(but-3-enamido)benzoate **1r** (48.1 mg, 0.22 mmol, 2.2 equiv.) and diphenylphosphane **2a** (18.6 mg, 0.1 mmol, 1.0 equiv.) at 80 °C for 12 h to afford **3r** as foam (27.3 mg, 63% yield, 99% ee). ^{1}H NMR (400 MHz, Chloroform-*d*) δ 8.07-7.84 (m, 7H), 7.49 (td, *J* = 7.5, 6.2, 3.0 Hz, 5H), 7.43-7.34 (m, 3H), 3.89 (d, *J* = 1.2 Hz, 3H), 3.57 (dtd, *J* = 9.6, 7.5, 7.0, 4.8 Hz, 1H), 2.70 (ddd, *J* = 15.7, 11.2, 4.7 Hz, 1H), 2.64-2.50 (m, 1H), 1.18 (dd, *J* = 18.4, 6.8 Hz, 2H). ^{31}P NMR (162 MHz, Chloroform-*d*) δ 52.39. ^{13}C NMR (101 MHz, Chloroform-*d*) δ 169.26 (d, *J* = 14.7 Hz), 166.72, 141.82, 131.82 (d, *J* = 2.9 Hz), 131.39 (dd, *J* = 12.3, 9.7 Hz), 130.78, 130.50 (d, *J* = 3.5 Hz), 128.86 (dd, *J* = 11.9, 5.6 Hz), 125.75, 118.84, 52.19, 38.97, 30.09 (d, *J* = 57.7 Hz), 13.89. $[\alpha]^{20}_{D}$= -69.4 (c 0.84, $CHCl_3$). The enantiomeric excess was determined by Daicel Chiralcel IF (0.46 cm x 25 cm), Hexanes /IPA = 85 / 15, 1.0 mL/min, λ = 254 nm, t (minor) = 12.1 min, t (major) = 14.1 min. HRMS (ESI-ion trap) m/z: [M+H]+ calcd for $C_{24}H_{25}$NO3PS 438.1293; found 438.1293.

(*R*)-3-(diphenylphosphorothioyl)-N-(naphthalen-2-yl)butanamide (3s)

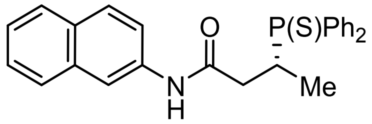

General procedure A was used with N-(naphthalen-2-yl)but-3-enamide **1s** (46.4 mg, 0.22 mmol, 2.2 equiv.) and diphenylphosphane **2a** (18.6 mg, 0.1 mmol, 1.0 equiv.) at 80 °C for 12 h to afford **3s** as foam (36.7 mg, 86% yield, >99% ee). ^{1}H NMR (400 MHz, Chloroform-*d*) δ 8.10 (s, 1H), 8.05-7.89 (m, 5H), 7.78-7.69 (m, 3H), 7.52-7.33 (m, 9H), 3.69-3.56 (m, 1H), 2.78-2.66 (m, 1H), 2.66-2.55 (m, 1H), 1.21 (dd, *J* = 18.5, 6.8 Hz, 3H). ^{31}P NMR (162 MHz, Chloroform-*d*) δ 52.50. ^{13}C NMR (101 MHz, Chloroform-*d*) δ 169.22 (d, *J* = 15.6 Hz), 135.10, 133.78, 131.74 (t, *J* = 3.1 Hz), 131.42 (dd, *J* = 9.5,

5.8 Hz), 130.70 (d, J = 11.7 Hz), 128.94, 128.84 (d, J = 2.1 Hz), 128.73, 127.76, 127.64, 126.59, 125.17, 119.86, 116.73, 38.73 (d, J = 1.9 Hz), 30.13 (d, J = 58.0 Hz), 13.71. $[\alpha]^{20}_{D}$= -55.9 (c 1.15, $CHCl_3$). The enantiomeric excess was determined by Daicel Chiralcel IF (0.46 cm x 25 cm), Hexanes /IPA = 85 / 15, 1.0 mL/min, λ = 254 nm, t (minor) = 9.0 min, t (major) = 12.0 min. HRMS (ESI-ion trap) m/z: [M+H]+ calcd for $C_{26}H_{25}NOPS$ 430.1394; found 430.1397.

(*R*)-N-(dibenzo[b,d]furan-2-yl)-3-(diphenylphosphorothioyl)butanamide (3t)

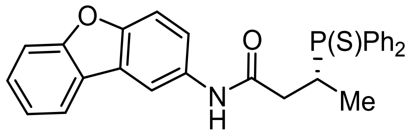

General procedure A was used with N-(dibenzo[b,d]furan-2-yl)but-3-enamide **1t** (55.2 mg, 0.22 mmol, 2.2 equiv.) and diphenylphosphane **2a** (18.6 mg, 0.1 mmol, 1.0 equiv.) at 80 ℃ for 12 h to afford **3t** as foam (31.6 mg, 68% yield, 98% ee). ^1H NMR (600 MHz, Chloroform-*d*) δ 8.07-7.89 (m, 6H), 7.86 (d, J = 7.6 Hz, 1H), 7.77 (d, J = 8.3 Hz, 1H), 7.53 (d, J = 8.2 Hz, 1H), 7.51-7.44 (m, 3H), 7.43-7.34 (m, 4H), 7.31 (t, J = 7.5 Hz, 1H), 7.17 (d, J = 8.3 Hz, 1H), 3.66-3.57 (m, 1H), 2.78-2.65 (m, 1H), 2.65-2.54 (m, 1H), 1.21 (dd, J = 18.5, 6.8 Hz, 3H). ^{31}P NMR (243 MHz, Chloroform-*d*) δ 52.50. ^{13}C NMR (151 MHz, Chloroform-*d*) δ 169.00 (d, J = 14.9 Hz), 156.65 (d, J = 15.0 Hz), 137.03, 131.78 (dd, J = 4.9, 2.9 Hz), 131.45 (dd, J = 12.2, 9.6 Hz), 131.03 (dd, J = 78.2, 4.8 Hz), 128.85 (t, J = 11.5 Hz), 126.72, 124.11, 122.91, 120.65, 120.62, 120.32, 114.90, 111.71, 103.51, 38.92, 30.17 (d, J = 57.7 Hz), 13.86. $[\alpha]^{20}_{D}$= -85.2 (c 0.83, $CHCl_3$). The enantiomeric excess was determined by Daicel Chiralcel IF (0.46 cm x 25 cm), Hexanes /IPA = 85 / 15, 1.0 mL/min, λ = 254 nm, t (major) = min, t (minor) = min. HRMS (ESI-ion trap) m/z: [M+H]+ calcd for $C_{28}H_{25}NO_2PS$ 470.1344; found 470.1343.

(*R*)-3-(diphenylphosphorothioyl)-N-(isoquinolin-4-yl)butanamide (3u)

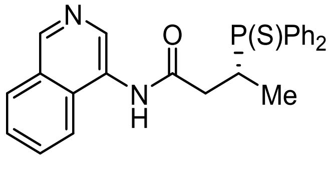

General procedure A was used with **1u** (mg, 0.22 mmol, 2.2 equiv.) and diphenylphosphane **2a** (18.6 mg, 0.1 mmol, 1.0 equiv.) at 80 °C for 12 h to afford **3u** as foam (33.3 mg, 78% yield, >99% ee). ^{1}H NMR (600 MHz, Chloroform-*d*) δ 9.04 (s, 1H), 8.69 (s, 1H), 8.20 (s, 1H), 8.06-7.90 (m, 5H), 7.80 (d, *J* = 8.5 Hz, 1H), 7.68 (t, *J* = 7.7 Hz, 1H), 7.59 (t, *J* = 7.6 Hz, 1H), 7.51-7.33 (m, 6H), 3.68-3.58 (m, 1H), 2.91-2.81 (m, 1H), 2.81-2.71 (m, 1H), 1.25 (dd, *J* = 18.2, 7.1 Hz, 3H). ^{31}P NMR (243 MHz, Chloroform-*d*) δ 52.64. ^{13}C NMR (151 MHz, Chloroform-*d*) δ 169.91 (d, *J* = 14.8 Hz), 149.93, 137.93, 132.64-131.65 (m), 131.47 (dd, *J* = 9.6, 4.2 Hz), 130.82, 130.09, 128.85 (dd, *J* = 14.8, 11.9 Hz), 128.71, 128.21, 127.78, 127.55, 120.70, 38.48, 30.15 (d, *J* = 57.2 Hz), 14.00. $[\alpha]^{20}_D$= -25.1 (c 0.93, $CHCl_3$). The enantiomeric excess was determined by Daicel Chiralcel IF (0.46 cm x 25 cm), Hexanes /IPA = 85 / 15, 1.0 mL/min, λ = 254 nm, t (minor) = 21.6 min, t (major) = 27.5 min. HRMS (ESI-ion trap) m/z: [M+H]+ calcd for $C_{25}H_{23}$N2OPS 431.1347; found 431.1343.

(*R*)-3-(diphenylphosphorothioyl)-N-(thiophen-3-yl)butanamide (3v)

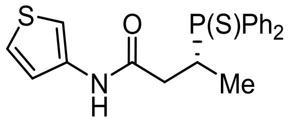

General procedure A was used with N-(thiophen-3-yl)but-3-enamide **1v** (36.7 mg, 0.22 mmol, 2.2 equiv.) and diphenylphosphane **2a** (18.6 mg, 0.1 mmol, 1.0 equiv.) at 80 °C for 12 h to afford **3v** as foam (13.6 mg, 36% yield, 92% ee). ^{1}H NMR (600 MHz, Chloroform-*d*) δ 8.02-7.91 (m, 4H), 7.86 (s, 1H), 7.54-7.36 (m, 7H), 7.17 (dd, *J* = 5.2, 3.3 Hz, 1H), 6.90 (dd, *J* = 5.1, 1.6 Hz, 1H), 3.59-3.52 (m, 1H), 2.65-2.59 (m, 1H), 2.51-2.45 (m, 1H), 1.16 (dd, *J* = 18.5, 6.7 Hz, 3H). ^{31}P NMR (243 MHz, Chloroform-*d*) δ 52.40. ^{13}C NMR (151 MHz, Chloroform-*d*) δ 168.11 (d, *J* = 15.5 Hz), 135.24, 131.75 (d, *J* = 3.5 Hz), 131.42 (dd, *J* = 14.5, 9.6 Hz), 131.04 (dd, *J* = 78.0, 6.9 Hz), 128.85 (dd, *J* = 13.0 Hz, 12.2 Hz), 124.53, 121.01, 110.44, 38.13 (d, *J* = 1.9 Hz), 30.18 (d, *J* = 57.9 Hz), 13.68. $[\alpha]^{20}_D$= -64.4 (c 0.39, $CHCl_3$). The enantiomeric excess was determined by Daicel Chiralcel IF (0.46 cm x 25 cm), Hexanes /IPA = 85 / 15, 1.0 mL/min, λ = 254

nm, t (minor) = 8.4 min, t (major) = 9.2 min. HRMS (ESI-ion trap) m/z: [M+H]+ calcd for $C_{20}H_{21}NOPS_2$ 386.0802; found 386.0796.

(*R*)-N-benzhydryl-3-(diphenylphosphorothioyl)butanamide (3w)

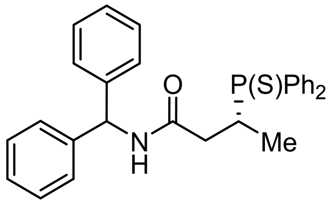

General procedure A was used with N-benzhydrylbut-3-enamide **1w** (55.2 mg, 0.22 mmol, 2.2 equiv.) and diphenylphosphane **2a** (18.6 mg, 0.1 mmol, 1.0 equiv.) at 80 ℃ for 12 h to afford **3w** as foam (30.3 mg, 65% yield, 98% ee). ^{1}H NMR (400 MHz, Chloroform-*d*) δ 8.04-7.84 (m, 4H), 7.50-7.38 (m, 6H), 7.33-7.23 (m, 6H), 7.21-7.09 (m, 4H), 6.29 (d, *J* = 7.9 Hz, 1H), 6.18 (d, *J* = 7.9 Hz, 1H), 3.56-3.44 (m, 1H), 2.54-2.30 (m, 2H), 1.11 (dd, *J* = 18.6, 6.7 Hz, 3H). ^{31}P NMR (162 MHz, Chloroform-*d*) δ 52.50. ^{13}C NMR (101 MHz, Chloroform-*d*) δ 169.84 (d, *J* = 17.8 Hz), 141.26 (d, *J* = 24.4 Hz), 131.69 (dd, *J* = 10.6, 3.0 Hz), 131.44 (dd, *J* = 9.6, 3.4 Hz), 131.17 (dd, *J* = 77.9, 4.6 Hz), 128.81 (dd, *J* = 18.0, 11.7 Hz), 128.81 (d, *J* = 2.5 Hz), 127.69, 127.62, 127.46, 127.32, 57.45, 37.29 (d, *J* = 2.0 Hz), 29.99 (d, *J* = 58.4 Hz), 13.15. $[\alpha]^{20}_{D}$= -3.71 (c 0.94, $CHCl_3$). The enantiomeric excess was determined by Daicel Chiralcel IF (0.46 cm x 25 cm), Hexanes /IPA = 85 / 15, 1.0 mL/min, λ = 254 nm, t (minor) = 9.0 min, t (major) = 11.0 min. HRMS (ESI-ion trap) m/z: [M+H]+ calcd for $C_{29}H_{29}NOPS$ 470.1707; found 470.1709.

(*R*)-3-(diphenylphosphorothioyl)-N-(thiophen-3-ylmethyl)butanamide (3x)

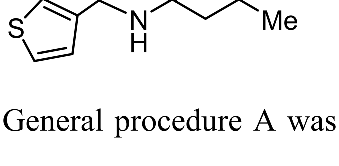

General procedure A was used with N-(thiophen-3-ylmethyl)but-3-enamide **1x** (39.8 mg, 0.22 mmol, 2.2 equiv.) and diphenylphosphane **2a** (18.6 mg, 0.1 mmol, 1.0 equiv.) at 80 ℃ for 12 h to afford **3x** as foam (23.0 mg, 58% yield, 88% ee). ^{1}H NMR (600

MHz, Chloroform-*d*) δ 8.02-7.91 (m, 4H), 7.49-7.43 (m, 6H), 7.26 (t, *J* = 2.7 Hz, 1H), 7.07 (d, *J* = 2.8 Hz, 1H), 6.94 (d, *J* = 5.0 Hz, 1H), 5.93 (t, *J* = 5.7 Hz, 1H), 4.39 (dd, *J* = 14.9, 5.9 Hz, 1H), 4.21 (dd, *J* = 14.9, 5.3 Hz, 1H), 3.55-3.49 (m, 1H), 2.49-2.42 (m, 1H), 2.34-2.27 (m, 1H), 1.11 (dd, *J* = 18.5, 6.8 Hz, 3H). ^{31}P NMR (243 MHz, Chloroform-*d*) δ 52.48. ^{13}C NMR (151 MHz, Chloroform-*d*) δ 170.40 (d, *J* = 16.3 Hz), 138.63, 131.70 (dd, *J* = 14.9, 3.0 Hz), 131.47 (dd, *J* = 9.4, 8.3 Hz), 131.27 (dd, *J* = 78.0, 12.3 Hz), 128.81 (dd, *J* = 19.0, 11.9 Hz), 127.30, 126.62, 122.49, 38.97, 37.38 (d, *J* = 1.7 Hz), 29.96 (d, *J* = 58.1 Hz), 13.39. $[\alpha]^{20}_{D}$= -11.5 (c 0.60, $CHCl_3$). The enantiomeric excess was determined by Daicel Chiralcel IF (0.46 cm x 25 cm), Hexanes /IPA = 85 / 15, 1.0 mL/min, λ = 254 nm, t (major) = 7.7 min, t (minor) = 13.9 min. HRMS (ESI-ion trap) m/z: [M+H]+ calcd for $C_{21}H_{22}NOPS_2$ 400.0959; found 400.0956.

(*R*)-N-benzyl-3-(diphenylphosphorothioyl)butanamide (3y)

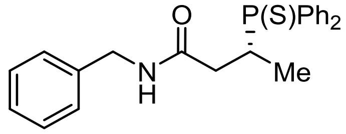

General procedure A was used with N-benzylbut-3-enamide **1y** (38.5 mg, 0.22 mmol, 2.2 equiv.) and diphenylphosphane **2a** (18.6 mg, 0.1 mmol, 1.0 equiv.) at 80 °C for 12 h to afford **3y** as foam (27.3 mg, 70% yield, 97% ee). ^{1}H NMR (600 MHz, Chloroform-*d*) δ 8.04-7.90 (m, 4H), 7.46 (qt, *J* = 8.3, 3.8 Hz, 6H), 7.31-7.23 (m, 3H), 7.19 (d, *J* = 7.4 Hz, 2H), 5.97 (d, *J* = 5.9 Hz, 1H), 4.39 (dd, *J* = 14.7, 5.9 Hz, 1H), 4.20 (dd, *J* = 14.7, 5.4 Hz, 1H), 3.57-3.50 (m, 1H), 2.51-2.41 (m, 1H), 2.37-2.27 (m, 1H), 1.12 (dd, *J* = 18.5, 6.8 Hz, 3H). ^{31}P NMR (243 MHz, Chloroform-*d*) δ 52.49. ^{13}C NMR (151 MHz, Chloroform-*d*) δ 170.50 (d, *J* = 16.5 Hz), 137.91, 131.69 (dd, *J* = 15.5, 3.0 Hz), 131.47 (t, *J* = 9.5 Hz), 131.28 (dd, *J* = 77.8, 10.0 Hz), 128.92, 128.84, 128.74 (d, *J* = 11.8 Hz), 127.84, 127.70, 43.88, 37.39 (d, *J* = 1.9 Hz), 29.96 (d, *J* = 58.2 Hz), 13.38. $[\alpha]^{20}_{D}$= -22.7 (c 0.83, $CHCl_3$). The enantiomeric excess was determined by Daicel Chiralcel IF (0.46 cm x 25 cm), Hexanes /IPA = 85 / 15, 1.0 mL/min, λ = 254 nm, t (minor) = 16.1 min, t (major) = 18.7 min. HRMS (ESI-ion trap) m/z: [M+H]+ calcd for $C_{23}H_{25}NOPS$ 394.1394; found 394.1389.

(*R*)-N-(2-bromobenzyl)-3-(diphenylphosphorothioyl)butanamide (3z)

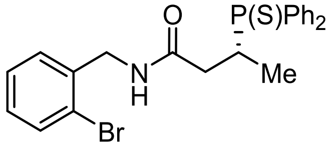

General procedure A was used with N-(2-bromobenzyl)but-3-enamide **1z** (55.9 mg, 0.22 mmol, 2.2 equiv.) and diphenylphosphane **2a** (18.6 mg, 0.1 mmol, 1.0 equiv.) at 80 °C for 12 h to afford **3z** as foam (17.6 mg, 38% yield, 96% ee). ^{1}H NMR (600 MHz, Chloroform-*d*) δ 8.03-7.86 (m, 4H), 7.51 (d, *J* = 7.9 Hz, 1H), 7.49-7.39 (m, 6H), 7.30-7.26 (m, 1H), 7.23 (t, *J* = 7.5 Hz, 1H), 7.14-7.07 (m, 1H), 6.17-6.14 (m, 1H), 4.43 (dd, *J* = 14.9, 6.2 Hz, 1H), 4.31 (dd, *J* = 15.0, 5.8 Hz, 1H), 3.54-3.45 (m, 1H), 2.48-2.40 (m, 1H), 2.36-2.29 (m, 1H), 1.10 (dd, *J* = 18.5, 6.8 Hz, 3H). ^{31}P NMR (243 MHz, Chloroform-*d*) δ 52.45. ^{13}C NMR (151 MHz, Chloroform-*d*) δ 170.56 (d, *J* = 16.8 Hz), 137.02, 132.93, 131.67 (dd, *J* = 16.1, 3.0 Hz), 131.44 (dd, *J* = 9.6, 7.2 Hz), 131.24 (dd, *J* = 77.9, 7.8 Hz), 130.36, 129.35, 128.78 (dd, *J* = 22.2, 11.8 Hz), 127.79, 123.79, 44.10, 37.30 (d, *J* = 1.9 Hz), 29.98 (d, *J* = 58.3 Hz), 13.32. $[\alpha]^{20}_{D}$= -48.5 (c 0.93, $CHCl_3$). The enantiomeric excess was determined by Daicel Chiralcel IF (0.46 cm x 25 cm), Hexanes /IPA = 85 / 15, 1.0 mL/min, λ = 254 nm, t (minor) = 12.4 min, t (major) = 16.2 min. HRMS (ESI-ion trap) m/z: [M+H]+ calcd for $C_{23}H_{24}BrNOPS$ 472.0500; found 472.0497.

methyl ((*R*)-3-(diphenylphosphorothioyl)butanoyl)-L-alaninate (3aa)

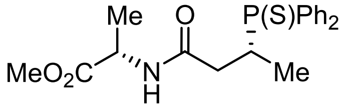

General procedure A was used with methyl but-3-enoyl-*L*-alaninate **1aa** (37.6 mg, 0.22 mmol, 2.2 equiv.) and diphenylphosphane **2a** (18.6 mg, 0.1 mmol, 1.0 equiv.) at 100 °C for 12 h to afford **3aa** as foam (20.5 mg, 53% yield,). ^{1}H NMR (600 MHz, Chloroform-*d*) δ 8.03-7.89 (m, 4H), 7.51-7.41 (m, 6H), 6.12 (d, *J* = 7.3 Hz, 1H), 4.51-4.40 (m, 1H), 3.72 (s, 3H), 3.55-3.42 (m, 1H), 2.47-2.40 (m, 1H), 2.38-2.30 (m, 1H), 1.30 (d, *J* = 7.2 Hz, 3H), 1.13 (dd, *J* = 18.6, 6.8 Hz, 3H). ^{31}P NMR (243 MHz, Chloroform-*d*) δ 52.44.

^{13}C NMR (151 MHz, Chloroform-*d*) δ 173.25, 170.37 (d, *J* = 17.1 Hz), 131.68 (dd, *J* = 13.5, 3.0 Hz), 131.49 (t, *J* = 9.9 Hz), 131.05 (d, *J* = 18.3 Hz), 128.79 (dd, *J* = 20.9, 11.8 Hz), 52.59, 48.32, 37.14 (d, *J* = 2.1 Hz), 30.02 (d, *J* = 58.3 Hz), 18.14, 13.22. $[\alpha]^{20}_{D}$= -74.2 (c 0.60, $CHCl_3$). HRMS (ESI-ion trap) m/z: [M+H]+ calcd for $C_{20}H_{24}NO_3PS$ 390.1293; found 390.1287.

methyl ((*R*)-3-(diphenylphosphorothioyl)butanoyl)-D-tryptophanate (3ab)

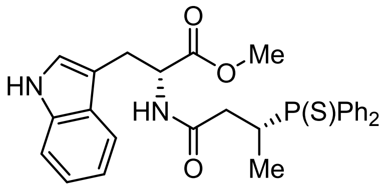

General procedure A was used withmethyl but-3-enoyl-D-tryptophanate **1ab** (62.9 mg, 0.22 mmol, 2.2 equiv.) and diphenylphosphane **2a** (18.6 mg, 0.1 mmol, 1.0 equiv.) at 80 ℃ for 12 h to afford **3ab** as foam (41.2 mg, 82% yield, >20/1 dr). ^{1}H NMR (600 MHz, Chloroform-*d*) δ 8.27 (s, 1H), 8.00-7.94 (m, 2H), 7.92-7.86 (m, 2H), 7.49-7.41 (m, 7H), 7.33 (d, *J* = 8.1 Hz, 1H), 7.18-7.13 (m, 1H), 7.00 (t, *J* = 7.5 Hz, 1H), 6.90 (d, *J* = 2.4 Hz, 1H), 6.07 (d, *J* = 7.6 Hz, 1H), 4.86-4.81 (m, 1H), 3.66 (d, *J* = 1.5 Hz, 3H), 3.51-3.42 (m, 1H), 3.22 (dd, *J* = 5.6, 2.1 Hz, 2H), 2.42-2.32 (m, 1H), 2.29-2.21 (m, 1H), 1.09 (dd, *J* = 18.6, 6.8 Hz, 3H). ^{31}P NMR (243 MHz, Chloroform-*d*) δ 52.43. ^{13}C NMR (151 MHz, Chloroform-*d*) δ 172.20, 170.52 (d, *J* = 17.5 Hz), 136.23, 131.67 (dd, *J* = 18.5, 3.0 Hz), 131.45 (t, *J* = 9.2 Hz), 130.99 (d, *J* = 20.1 Hz), 128.79 (dd, *J* = 28.4, 11.9 Hz), 127.54, 122.88, 122.43, 119.86, 118.48, 111.46, 109.76, 52.82 (d, *J* = 98.6 Hz), 37.10 (d, *J* = 2.1 Hz), 30.00 (d, *J* = 58.4 Hz), 27.46, 13.10. $[\alpha]^{20}_{D}$= -0.79 (c 1.27, $CHCl_3$). HRMS (ESI-ion trap) m/z: [M+H]+ calcd for $C_{28}H_{30}N_2O_3PS$ 505.1715; found 505.1712.

(*R*)-3-(diphenylphosphorothioyl)-N-methyl-N-phenylbutanamide (3ac)

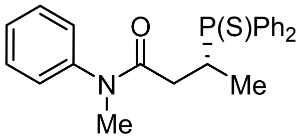

General procedure A was used with N-methyl-N-phenylbut-3-enamide **1ac** (38.5 mg, 0.22 mmol, 2.2 equiv.) and diphenylphosphane **2a** (18.6 mg, 0.1 mmol, 1.0 equiv.) at 90 °C for 12 h to afford **3ac** as foam (29.0 mg, 74% yield, >99% ee). ^{1}H NMR (600 MHz, Chloroform-*d*) δ 7.93-7.85 (m, 4H), 7.48-7.40 (m, 6H), 7.38-7.30 (m, 3H), 6.98 (d, *J* = 7.5 Hz, 2H), 3.61-3.54 (m, 1H), 3.21 (s, 3H), 2.31-2.20 (m, 2H), 1.08 (dd, *J* = 18.8, 6.8 Hz, 3H). ^{31}P NMR (243 MHz, Chloroform-*d*) δ 52.46. ^{13}C NMR (151 MHz, Chloroform-*d*) δ 170.83 (d, *J* = 16.6 Hz), 143.28, 131.52 (dd, *J* = 77.5, 7.1 Hz), 131.50 (d, *J* = 10.9 Hz), 131.46 (dd, *J* = 18.9, 9.7 Hz), 130.07, 128.68 (dd, *J* = 16.8, 11.7 Hz), 128.22, 127.22, 37.74, 34.72 (d, *J* = 3.0 Hz), 30.17 (d, *J* = 58.8 Hz), 13.65. $[\alpha]^{20}_D$= 7.67 (c 0.90, $CHCl_3$). The enantiomeric excess was determined by Daicel Chiralcel IF (0.46 cm x 25 cm), Hexanes /IPA = 85 / 15, 1.0 mL/min, λ = 254 nm, t (minor) = 16.9 min, t (major) = 18.8 min. HRMS (ESI-ion trap) m/z: [M+H]+ calcd for $C_{23}H_{25}NOPS$ 394.1394; found 394.1392.

(*R*)-3-(diphenylphosphorothioyl)-N-phenylpentanamide (3ad)

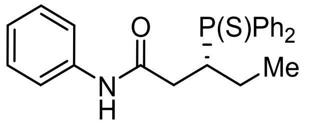

General procedure A was used with (*E*)-N-phenylpent-3-enamide **1ad** (38.5 mg, 0.22 mmol, 2.2 equiv.) and diphenylphosphane **2a** (14.3 mg, 0.1 mmol, 1.0 equiv.) at 80 °C for 12 h to afford **3ad** as foam (mg, 37% yield, 95% ee). ^{1}H NMR (600 MHz, Chloroform-*d*) δ 8.04-7.92 (m, 4H), 7.57 (s, 1H), 7.51-7.42 (m, 3H), 7.40-7.30 (m, 5H), 7.24 (t, *J* = 7.8 Hz, 2H), 7.06 (t, *J* = 7.4 Hz, 1H), 3.50-3.43 (m, 1H), 2.80-2.73 (m, 1H), 2.58-2.51 (m, 1H), 1.67-1.60 (m, 2H), 0.93 (t, *J* = 7.5 Hz, 3H). ^{31}P NMR (243 MHz, Chloroform-*d*) δ 52.25. ^{13}C NMR (151 MHz, Chloroform-*d*) δ 169.27 (d, *J* = 11.4 Hz), 137.72, 131.83, 131.65, 131.49 (dd, *J* = 13.6, 9.9 Hz), 131.25 (d, *J* = 19.0 Hz), 128.91, 128.76 (dd, *J* = 12.1, 3.1 Hz), 124.42, 119.83, 37.21, 36.00 (d, *J* = 56.6 Hz), 22.92,

12.44 (d, J = 12.9 Hz). $[\alpha]^{20}_{D}$= -108.0 (c 0.40, $CHCl_3$). The enantiomeric excess was determined by Daicel Chiralcel IF (0.46 cm x 25 cm), Hexanes /IPA = 85 / 15, 1.0 mL/min, λ = 254 nm, t (minor) = 5.9 min, t (major) = 6.5 min. HRMS (ESI-ion trap) m/z: [M+H]+ calcd for $C_{23}H_{25}NOPS$ 394.1394; found 394.1391.

(*R*)-3-(di-*p*-tolylphosphorothioyl)-N-phenylbutanamide (3ae)

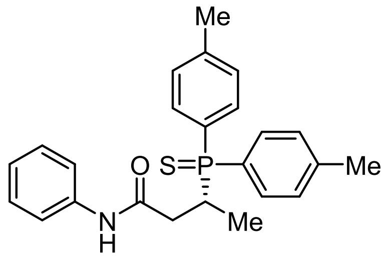

General procedure A was used with N-phenylbut-3-enamide **1a** (35.4 mg, 0.22 mmol, 2.2 equiv.) and di-*p*-tolylphosphane **2b** (21.4 mg, 0.1 mmol, 1.0 equiv.) at 80 ℃ for 12 h to afford **3ae** as foam (21.3 mg, 53% yield, >99% ee). ^{1}H NMR (400 MHz, Chloroform-*d*) δ 7.90-7.77 (m, 4H), 7.59 (s, 1H), 7.45-7.36 (m, 2H), 7.28-7.24 (m, 4H), 7.19 (dd, J = 8.2, 2.8 Hz, 2H), 7.11-7.02 (m, 1H), 3.55-3.45 (m, 1H), 2.70-2.59 (m, 1H), 2.54-2.44 (m, 1H), 2.37 (s, 3H), 2.28 (s, 3H), 1.17 (dd, J = 18.4, 6.8 Hz, 3H). ^{31}P NMR (162 MHz, Chloroform-*d*) δ 51.91. ^{13}C NMR (101 MHz, Chloroform-*d*) δ 169.07 (d, J = 15.2 Hz), 142.23 (dd, J = 10.9, 2.9 Hz), 137.72, 131.41 (dd, J = 10.0, 6.0 Hz), 129.55 (dd, J = 12.2, 7.1 Hz), 128.96, 128.93-126.90 (m), 124.44, 119.77, 38.74 (d, J = 1.9 Hz), 30.21 (d, J = 58.1 Hz), 21.51 (dd, J = 6.9, 1.4 Hz), 13.74. $[\alpha]^{20}_{D}$= -32.4 (c 0.63, $CHCl_3$). The enantiomeric excess was determined by Daicel Chiralcel IF (0.46 cm x 25 cm), Hexanes /IPA = 85 / 15, 1.0 mL/min, λ = 254 nm, t (minor) = 9.0 min, t (major) = 10.5 min. HRMS (ESI-ion trap) m/z: [M+H]+ calcd for $C_{24}H_{27}NOPS$ 408.1551; found 408.1547.

(*R*)-3-(bis(3,5-dimethylphenyl)phosphorothioyl)-N-phenylbutanamide (3af)

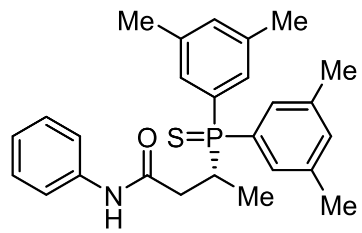

General procedure A was used with N-phenylbut-3-enamide **1a** (35.4 mg, 0.22 mmol, 2.2 equiv.) and bis(3,5-dimethylphenyl)phosphane **2c** (24.2mg, 0.1 mmol, 1.0 equiv.) at 90 °C for 12 h to afford **3af** as foam (24.1 mg, 55% yield, 97% ee). ^{1}H NMR (600 MHz, Chloroform-*d*) δ 7.61-7.52 (m, 5H), 7.44-7.40 (m, 2H), 7.27-7.25 (m, 2H), 7.11-7.05 (m, 2H), 6.99 (s, 1H), 3.57-3.50 (m, 1H), 2.68-2.60 (m, 1H), 2.54-2.46 (m, 1H), 2.34 (s, 6H), 2.28 (s, 6H), 1.18 (dd, *J* = 18.3, 6.8 Hz, 3H). ^{31}P NMR (243 MHz, Chloroform-*d*) δ 52.47. ^{13}C NMR (151 MHz, Chloroform-*d*) δ 169.12 (d, *J* = 15.2 Hz), 138.53 (dd, *J* = 12.4, 9.5 Hz), 137.77, 134.88-131.86 (m), 130.87 (dd, *J* = 77.2, 16.2 Hz), 128.95 (d, *J* = 20.5 Hz), 128.94, 124.44, 119.65, 38.81, 29.96 (d, *J* = 57.6 Hz), 21.48 (d, *J* = 5.4 Hz), 13.82. $[\alpha]^{20}_{D}$= -45.8 (c 0.67, $CHCl_3$). The enantiomeric excess was determined by Daicel Chiralcel IF (0.46 cm x 25 cm), Hexanes /IPA = 85 / 15, 1.0 mL/min, λ = 254 nm, t (major) = 6.8 min, t (minor) = 7.7 min. HRMS (ESI-ion trap) m/z: [M+H]+ calcd for $C_{26}H_{31}NOPS$ 436.1864; found 436.1861.

(*R*)-3-(di-m-tolylphosphorothioyl)-N-phenylbutanamide (3ag)

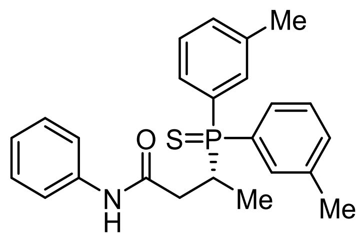

General procedure B was used with N-phenylbut-3-enamide **1a** (35.4 mg, 0.22 mmol, 2.2 equiv.) and di-*m*-tolylphosphane prepared through reduction of di-*m*-tolylphosphine oxide **2d** (23.0 mg, 0.1 mmol, 1.0 equiv.) *in situ* by $PhSiH_3$ (10.8 mg, 0.1 mmol, 1.0 equiv.) at 90 °C for 36 h to afford **3ag** as foam (34.8 mg, 84% yield, 81% ee). ^{1}H NMR (600 MHz, Chloroform-*d*) δ 7.87-7.69 (m, 5H), 7.46 (d, *J* = 8.0 Hz, 2H), 7.38-7.34 (m, 1H), 7.33-7.26 (m, 4H), 7.22 (d, *J* = 7.6 Hz, 1H), 7.09 (t, *J* = 7.4 Hz, 1H), 3.63-3.55 (m,

1H), 2.74-2.62 (m, 1H), 2.61-2.52 (m, 1H), 2.40 (s, 3H), 2.32 (s, 3H), 1.20 (dd, *J* = 18.5, 6.7 Hz, 3H). ^{31}P NMR (243 MHz, Chloroform-*d*) δ 52.51. ^{13}C NMR (151 MHz, Chloroform-*d*) δ 169.08 (d, *J* = 15.7 Hz), 138.78 (dd, *J* = 22.1, 11.8 Hz), 137.75, 132.52 (dd, *J* = 5.3, 3.0 Hz), 132.11 (dd, *J* = 13.9, 9.8 Hz), 130.92 (dd, *J* = 77.6, 3.1 Hz), 128.93, 128.61 (dd, *J* = 12.4, 10.1 Hz), 128.18 (dd, *J* = 35.3, 9.5 Hz), 124.44, 119.79, 38.65, 30.02 (d, *J* = 57.8 Hz), 21.57 (d, *J* = 9.2 Hz), 13.68. $[\alpha]^{20}_{D}$= -23.3 (c 1.06, $CHCl_3$). The enantiomeric excess was determined by Daicel Chiralcel IF (0.46 cm x 25 cm), Hexanes /IPA = 85 / 15, 1.0 mL/min, λ = 254 nm, t (minor) = 6.7 min, t (major) = 7.3 min. HRMS (ESI-ion trap) m/z: [M+H]+ calcd for $C_{24}H_{27}NOPS$ 408.1551; found 408.1547.

(*R*)-3-(bis(4-(tert-butyl)phenyl)phosphorothioyl)-N-phenylbutanamide (3ah)

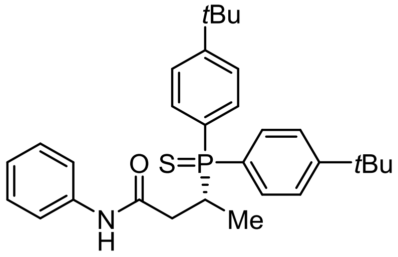

General procedure B was used with N-phenylbut-3-enamide **1a** (35.4 mg, 0.22 mmol, 2.2 equiv.) and bis(4-(tert-butyl)phenyl)phosphane prepared through reduction of bis(4-(tert-butyl)phenyl)phosphine oxide **2e** (31.4 mg, 0.1 mmol, 1.0 equiv.) *in situ* by DEMS (17.2 mg, 0.1 mmol, 1.0 equiv.) at 80℃ for 36 h to afford **3ah** as foam (27.0 mg, 55% yield, 89% ee). ^{1}H NMR (600 MHz, Chloroform-*d*) δ 7.94-7.83 (m, 4H), 7.74 (s, 1H), 7.48-7.44 (m, 2H), 7.44-7.38 (m, 4H), 7.26-7.22 (m, 2H), 7.06 (t, *J* = 7.4 Hz, 1H), 3.58-3.49 (m, 1H), 2.71-2.63 (m, 1H), 2.57-2.48 (m, 1H), 1.30 (d, *J* = 1.6 Hz, 9H), 1.24 (d, *J* = 1.7 Hz, 9H), 1.17 (dd, *J* = 18.3, 6.8 Hz, 3H). ^{31}P NMR (243 MHz, Chloroform-d) δ 51.45. ^{13}C NMR (151 MHz, Chloroform-*d*) δ 169.14 (d, *J* = 14.9 Hz), 155.09 (dd, *J* = 5.5, 2.8 Hz), 137.77, 131.33 (t, *J* = 10.4 Hz), 128.97, 127.80 (dd, *J* = 80.4, 12.9 Hz), 126.52-125.45 (m), 124.39, 119.78, 38.79, 34.98 (d, *J* = 11.1 Hz), 31.18 (d, *J* = 10.4 Hz), 30.29 (d, *J* = 58.1 Hz), 13.79. $[\alpha]^{20}_{D}$= -25.1 (c 0.83, $CHCl_3$). The enantiomeric excess was determined by Daicel Chiralcel IF (0.46 cm x 25 cm), Hexanes /IPA = 85 /

15, 1.0 mL/min, λ = 254 nm, t (minor) = 4.7 min, t (major) = 5.5 min. HRMS (ESI-ion trap) m/z: [M+H]+ calcd for $C_{30}H_{39}NOPS$ 492.2490; found 492.2487.

(*R*)-3-(bis(4-chlorophenyl)phosphorothioyl)-N-phenylbutanamide (3ai)

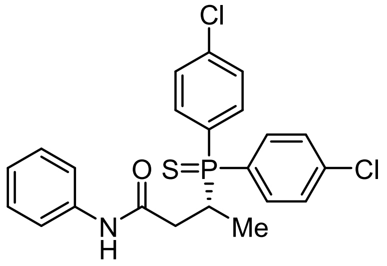

General procedure B was used with N-phenylbut-3-enamide **1a** (35.4 mg, 0.22 mmol, 2.2 equiv.) and bis(4-chlorophenyl)phosphane prepared through reduction of bis(4-chlorophenyl)phosphine oxide **2f** (27.1 mg, 0.1 mmol, 1.0 equiv.) *in situ* by $PhSiH_3$ (10.8 mg, 0.1 mmol, 1.0 equiv.) at 90℃ for 36 h to afford **3ai** as foam (32.0 mg, 72% yield, 87% ee). ^1H NMR (600 MHz, Chloroform-*d*) δ 7.97-7.80 (m, 4H), 7.78 (s, 1H), 7.50-7.33 (m, 6H), 7.29 (dd, *J* = 9.2, 6.3 Hz, 2H), 7.11 (t, *J* = 7.4 Hz, 1H), 3.60-3.50 (m, 1H), 2.67-2.58 (m, 1H), 2.58-2.47 (m, 1H), 1.17 (dd, *J* = 18.8, 6.8 Hz, 3H). ^{31}P NMR (243 MHz, Chloroform-*d*) δ 51.58. ^{13}C NMR (151 MHz, Chloroform-*d*) δ 168.64 (d, *J* = 15.2 Hz), 138.71 (dd, *J* = 13.9, 3.5 Hz), 137.47, 132.73 (dd, *J* = 10.6, 4.7 Hz), 129.34 (dd, *J* = 79.4, 16.8 Hz), 129.21 (dd, *J* = 12.5, 6.0 Hz), 129.06, 124.72, 119.87, 38.57, 30.09 (d, *J* = 58.4 Hz), 13.70. $[\alpha]^{20}_D$= -50.4 (c 1.0, $CHCl_3$). The enantiomeric excess was determined by Daicel Chiralcel IF (0.46 cm x 25 cm), Hexanes /IPA = 85 / 15, 1.0 mL/min, λ = 254 nm, t (minor) = 7.5 min, t (major) = 8.6 min. HRMS (ESI-ion trap) m/z: [M+H]+ calcd for $C_{22}H_{21}Cl_2NOPS$ 448.0459; found 448.0458.

(*R*)-3-(bis(3-chlorophenyl)phosphorothioyl)-N-phenylbutanamide (3aj)

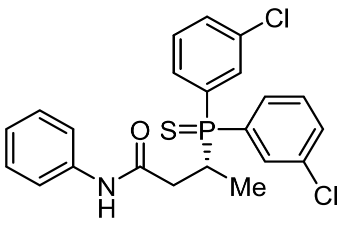

General procedure B was used with N-phenylbut-3-enamide **1a** (35.4 mg, 0.22 mmol, 2.2 equiv.) and bis(3-chlorophenyl)phosphane prepared through reduction of bis(3-chlorophenyl)phosphine oxide **2g** (27.1 mg, 0.1 mmol, 1.0 equiv.) *in situ* by $PhSiH_3$ (10.8 mg, 0.1 mmol, 1.0 equiv.) at 90 °C for 12 h to afford **3aj** as foam (27.9 mg, 63% yield, 81% ee). ^{1}H NMR (600 MHz, Chloroform-*d*) δ 7.98 (dd, *J* = 38.1, 13.1 Hz, 2H), 7.83-7.71 (m, 2H), 7.64 (s, 1H), 7.47 (d, *J* = 8.1 Hz, 1H), 7.43-7.32 (m, 5H), 7.27-7.25 (m, 2H), 7.08 (t, *J* = 7.4 Hz, 1H), 3.60-3.53 (m, 1H), 2.64-2.57 (m, 1H), 2.54-2.47 (m, 1H), 1.17 (dd, *J* = 19.0, 6.7 Hz, 3H). ^{31}P NMR (243 MHz, Chloroform-*d*) δ 51.95. ^{13}C NMR (151 MHz, Chloroform-*d*) δ 168.48 (d, *J* = 15.1 Hz), 137.47, 135.57 (t, *J* = 15.8 Hz), 133.13 (dd, *J* = 75.9, 5.8 Hz), 132.17 (d, *J* = 2.6 Hz), 131.67 (dd, *J* = 36.1, 11.1 Hz), 130.24 (d, *J* = 12.6 Hz), 129.03, 128.96 (dd, *J* = 48.8, 9.0 Hz), 124.69, 119.81, 38.53, 30.06 (d, *J* = 58.3 Hz), 13.75. $[\alpha]^{20}_D$= -25.9 (c 0.53, $CHCl_3$). The enantiomeric excess was determined by Daicel Chiralcel IF (0.46 cm x 25 cm), Hexanes /IPA = 85 / 15, 1.0 mL/min, λ = 254 nm, t (minor) = 5.7 min, t (major) = 6.3 min. HRMS (ESI-ion trap) m/z: [M+H]+ calcd for $C_{22}H_{21}Cl_2NOPS$ 448.0459; found 448.0454.

(*R*)-3-(bis(4-(trifluoromethoxy)phenyl)phosphorothioyl)-N-phenylbutanamide (3ak)

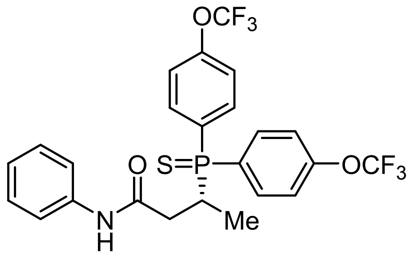

General procedure B was used with N-phenylbut-3-enamide **1a** (35.4 mg, 0.22 mmol, 2.2 equiv.) and bis(4-(trifluoromethoxy)phenyl)phosphane prepared through reduction of bis(4-(trifluoromethoxy)phenyl)phosphine oxide **2h** (37.0 mg, 0.1 mmol, 1.0 equiv.) *in situ* by $PhSiH_3$ (10.8 mg, 0.1 mmol, 1.0 equiv.) at 80°C for 36 h to afford **3ak** as foam (38.6 mg, 71% yield, 91% ee). ^{1}H NMR (600 MHz, Chloroform-*d*) δ 8.07-7.90 (m, 4H), 7.71 (s, 1H), 7.39 (d, *J* = 8.0 Hz, 2H), 7.31-7.21 (m, 6H), 7.09 (t, *J* = 7.4 Hz, 1H), 3.62-3.54 (m, 1H), 2.66-2.58 (m, 1H), 2.56-2.46 (m, 1H), 1.16 (dd, *J* = 18.8, 6.6

Hz, 3H). ^{31}P NMR (243 MHz, Chloroform-*d*) δ 51.12. ^{19}F NMR (565 MHz, Chloroform-*d*) δ -57.64, -57.65. ^{13}C NMR (151 MHz, Chloroform-*d*) δ 168.56 (d, *J* = 15.1 Hz), 151.99, 137.45, 133.41 (d, *J* = 11.0 Hz), 129.53 (d, *J* = 26.7 Hz), 129.08, 128.92, 124.78, 120.76 (dd, *J* = 12.8, 7.2 Hz), 120.34 (qd, *J* = 259.2, 9.8 Hz), 119.84, 38.62, 30.18 (d, *J* = 58.5 Hz), 13.73. $[\alpha]^{20}_{D}$= -17.7 (c 1.20, $CHCl_3$). The enantiomeric excess was determined by Daicel Chiralcel IF (0.46 cm x 25 cm), Hexanes /IPA = 85 / 15, 1.0 mL/min, λ = 254 nm, t (minor) = 4.1 min, t (major) = 4.4 min. HRMS (ESI-ion trap) m/z: [M+H]+ calcd for $C_{24}H_{21}F_6NO_3PS$ 548.0884; found 548.0881.

(*R*)-3-(di([1,1'-biphenyl]-4-yl)phosphorothioyl)-N-phenylbutanamide (3al)

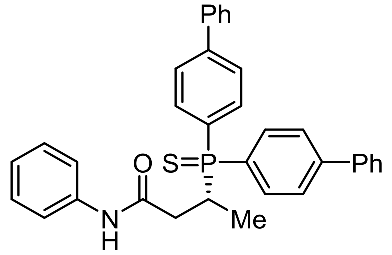

General procedure B was used with N-phenylbut-3-enamide **1a** (35.4 mg, 0.22 mmol, 2.2 equiv.) and di([1,1'-biphenyl]-4-yl)phosphane prepared through reduction of di([1,1'-biphenyl]-4-yl)phosphine oxide **2i** (35.4 mg, 0.1 mmol, 1.0 equiv.) *in situ* by $PhSiH_3$ (10.8 mg, 0.1 mmol, 1.0 equiv.) at 90 °C for 36 h to afford **3al** as foam (27.2 mg, 54% yield, 94% ee). ^{1}H NMR (600 MHz, Chloroform-*d*) δ 8.14-8.01 (m, 4H), 7.70 (d, *J* = 8.0 Hz, 2H), 7.65-7.56 (m, 5H), 7.53-7.35 (m, 10H), 7.25 (dd, *J* = 12.1, 5.5 Hz, 2H), 7.06 (t, *J* = 7.5 Hz, 1H), 3.72-3.62 (m, 1H), 2.79-2.70 (m, 1H), 2.63-2.52 (m, 1H), 1.26 (dd, *J* = 18.6, 6.8 Hz, 3H). ^{31}P NMR (243 MHz, Chloroform-*d*) δ 51.98. ^{13}C NMR (151 MHz, Chloroform-*d*) δ 168.91 (d, *J* = 14.9 Hz), 144.64 (dd, *J* = 14.2, 2.8 Hz), 139.80 (d, *J* = 6.5 Hz), 137.64, 133.25-131.35 (m), 129.69 (dd, *J* = 79.4, 11.7 Hz), 129.09, 129.02 (d, *J* = 3.7 Hz), 128.30 (d, *J* = 9.2 Hz), 127.54 (t, *J* = 11.3 Hz), 127.40, 124.54, 119.80, 38.87, 30.30 (d, *J* = 57.9 Hz), 13.90. $[\alpha]^{20}_{D}$= -21.1 (c 0.83, $CHCl_3$). The enantiomeric excess was determined by Daicel Chiralcel IF (0.46 cm x 25 cm), Hexanes /IPA = 85 / 15, 1.0 mL/min, λ = 254 nm, t (minor) = 14.3 min, t (major) = 18.1 min. HRMS (ESI-ion trap) m/z: [M+H]+ calcd for $C_{34}H_{31}NOPS$ 532.1864; found 532.1864.

(*R*)-3-(di(naphthalen-2-yl)phosphorothioyl)-N-phenylbutanamide (3am)

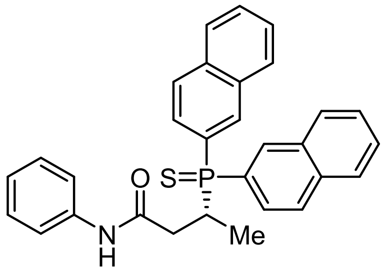

General procedure B was used with N-phenylbut-3-enamide **1a** (35.4 mg, 0.22 mmol, 2.2 equiv.) and di(naphthalen-2-yl)phosphane prepared through reduction of di(naphthalen-2-yl)phosphine oxide **2j** (30.2 mg, 0.1 mmol, 1.0 equiv.) *in situ* by $PhSiH_3$ (10.8 mg, 0.1 mmol, 1.0 equiv.) at 90 ℃ for 12 h to afford **3am** as foam (28.5 mg, 60% yield, 80% ee). ^{1}H NMR (600 MHz, Chloroform-*d*) δ 8.70-8.61 (m, 2H), 8.00-7.88 (m, 5H), 7.87-7.82 (m, 2H), 7.78 (d, *J* = 8.0 Hz, 1H), 7.60-7.49 (m, 5H), 7.35 (d, *J* = 8.0 Hz, 2H), 7.18 (t, *J* = 7.9 Hz, 2H), 7.01 (t, *J* = 7.4 Hz, 1H), 3.87-3.80 (m, 1H), 2.78-2.72 (m, 1H), 2.63-2.56 (m, 1H), 1.27 (dd, *J* = 18.3, 6.7 Hz, 4H). ^{31}P NMR (243 MHz, Chloroform-*d*) δ 52.90. ^{13}C NMR (151 MHz, Chloroform-*d*) δ 168.92 (d, *J* = 15.5 Hz), 137.54, 134.57 (d, *J* = 2.5 Hz), 133.80 (dd, *J* = 17.9, 9.8 Hz), 132.66 (dd, *J* = 13.3, 2.5 Hz), 129.14 (d, *J* = 4.4 Hz), 128.92, 128.76 (dd, *J* = 21.8, 11.5 Hz), 128.41 (d, *J* = 8.6 Hz), 128.24 (dd, *J* = 78.3, 7.8 Hz), 127.81 (d, *J* = 7.5 Hz), 127.16 (d, *J* = 9.0 Hz), 125.88 (dd, *J* = 10.1, 5.3 Hz), 124.50, 119.75, 38.80, 29.93 (d, *J* = 58.2 Hz), 13.87. $[\alpha]^{20}_D$= -48.2 (c 0.88, $CHCl_3$). The enantiomeric excess was determined by Daicel Chiralcel IF (0.46 cm x 25 cm), Hexanes /IPA = 85 / 15, 1.0 mL/min, λ = 254 nm, t (minor) = 14.8 min, t (major) = 17.3 min. HRMS (ESI-ion trap) m/z: [M+H]+ calcd for $C_{30}H_{27}NOPS$ 480.1551; found 480.1551.

4-(diphenylphosphorothioyl)-N-phenylbutanamide (4a)

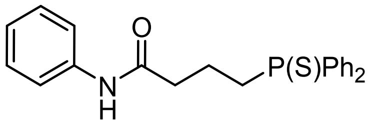

General procedure C was used with N-phenylbut-3-enamide **1a** (35.4 mg, 0.22 mmol, 2.2 equiv.) and diphenylphosphane **2a** (18.6 mg, 0.1 mmol, 1.0 equiv.) at 90 ℃ for 18

h to afford **4a** as colorless oil (34.2 mg, 90% yield). ^{1}H NMR (600 MHz, Chloroform-*d*) δ 7.89-7.78 (m, 4H), 7.56-7.42 (m, 8H), 7.34-7.27 (m, 2H), 7.14-7.06 (m, 1H), 2.64-2.56 (m, 2H), 2.56-2.49 (m, 2H), 2.12-2.02 (m, 2H). ^{31}P NMR (162 MHz, Chloroform-*d*) δ 42.37. ^{13}C NMR (101 MHz, Chloroform-*d*) δ 170.49, 137.91, 132.51 (d, *J* = 80.3 Hz), 131.72 (d, *J* = 2.9 Hz), 131.18 (d, *J* = 10.2 Hz), 129.08, 128.85 (d, *J* = 12.0 Hz), 124.40, 119.96 (d, *J* = 2.1 Hz), 37.11 (d, *J* = 13.1 Hz), 31.28 (d, *J* = 56.6 Hz), 18.93 (d, *J* = 2.2 Hz). HRMS (ESI-ion trap) m/z: [M+H]+ calcd for $C_{22}H_{23}NOPS$ 380.1238; found 380.1234.

4-(diphenylphosphorothioyl)-N-(o-tolyl)butanamide (4b)

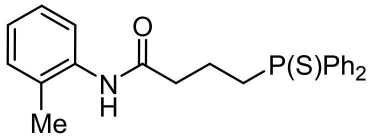

General procedure C was used with N-(o-tolyl)but-3-enamide **1b** (38.5mg, 0.22 mmol, 2.2 equiv.) and diphenylphosphane **2a** (18.6 mg, 0.1 mmol, 1.0 equiv.) at 90 ℃ for 18 h to afford **4b** as colorless oil (33.3 mg, 85% yield). ^{1}H NMR (600 MHz, Chloroform-*d*) δ 7.88-7.81 (m, 4H), 7.75 (d, *J* = 8.1 Hz, 1H), 7.50-7.42 (m, 6H), 7.23-7.13 (m, 3H), 7.07 (t, *J* = 7.4 Hz, 1H), 2.68-2.52 (m, 4H), 2.23 (s, 3H), 2.16-2.04 (m, 2H). ^{31}P NMR (243 MHz, Chloroform-*d*) δ 42.36. ^{13}C NMR (151 MHz, Chloroform-*d*) δ 170.47, 135.63, 132.71 (d, *J* = 80.2 Hz), 131.69 (d, *J* = 3.0 Hz), 131.21 (d, *J* = 10.0 Hz), 130.66, 129.38, 128.85 (d, *J* = 12.1 Hz), 126.85, 125.44, 123.37, 37.03 (d, *J* = 13.1 Hz), 31.40 (d, *J* = 57.0 Hz), 19.12, 18.11. HRMS (ESI-ion trap) m/z: [M+H]+ calcd for $C_{23}H_{25}NOPS$ 394.1394; found 394.1392.

4-(diphenylphosphorothioyl)-N-(m-tolyl)butanamide (4c)

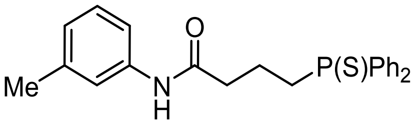

General procedure C was used with N-(m-tolyl)but-3-enamide 1c (38.5mg, 0.22 mmol, 2.2 equiv.) and diphenylphosphane **2a** (18.6 mg, 0.1 mmol, 1.0 equiv.) at 90 ℃ for 18

h to afford **4c** as colorless oil (24.4 mg, 62% yield). ^{1}H NMR (600 MHz, Chloroform-*d*) δ 7.88-7.79 (m, 4H), 7.52-7.41 (m, 7H), 7.39-7.34 (m, 1H), 7.28 (d, *J* = 8.0 Hz, 1H), 7.19 (t, *J* = 7.8 Hz, 1H), 2.64-2.58 (m, 2H), 2.52 (t, *J* = 6.8 Hz, 2H), 2.33 (s, 3H), 2.11-2.04 (m, 2H). ^{31}P NMR (243 MHz, Chloroform-*d*) δ 42.33. ^{13}C NMR (151 MHz, Chloroform-*d*) δ 170.38, 139.04, 137.82, 132.91, 132.38, 131.72 (d, *J* = 3.0 Hz), 131.22 (d, *J* = 10.2 Hz), 128.87 (d, *J* = 12.1 Hz), 125.26, 120.62, 117.06, 37.18 (d, *J* = 12.6 Hz), 31.31 (d, *J* = 56.7 Hz), 21.62, 19.00 (d, *J* = 2.4 Hz). HRMS (ESI-ion trap) m/z: [M+H]+ calcd for $C_{23}H_{25}NOPS$ 394.1394; found 394.1391.

4-(diphenylphosphorothioyl)-N-(p-tolyl)butanamide (4d)

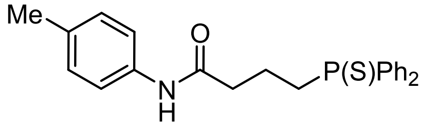

General procedure C was used with N-(p-tolyl)but-3-enamide **1d** (38.5mg, 0.22 mmol, 2.2 equiv.) and diphenylphosphane **2a** (18.6 mg, 0.1 mmol, 1.0 equiv.) at 90 °C for 18 h to afford **4d** as colorless oil (33.4mg, 85% yield). ^{1}H NMR (400 MHz, Chloroform-*d*) δ 7.88-7.79 (m, 4H), 7.54 (s, 1H), 7.51-7.41 (m, 6H), 7.41-7.35 (m, 2H), 7.10 (d, *J* = 8.1 Hz, 2H), 2.67-2.55 (m, 2H), 2.50 (t, *J* = 6.8 Hz, 2H), 2.30 (s, 3H), 2.14-2.00 (m, 2H). ^{31}P NMR (162 MHz, Chloroform-*d*) δ 42.38. ^{13}C NMR (101 MHz, Chloroform-*d*) δ 170.32, 135.32, 134.05, 132.56 (d, *J* = 80.3 Hz), 131.71 (d, *J* = 2.9 Hz), 131.19 (d, *J* = 10.2 Hz), 129.57, 128.85 (d, *J* = 12.1 Hz), 120.08, 37.09 (d, *J* = 13.0 Hz), 31.32 (d, *J* = 56.8 Hz), 20.99, 18.95 (d, *J* = 2.2 Hz). ^{31}P NMR (243 MHz, Chloroform-*d*) δ 42.36. HRMS (ESI-ion trap) m/z: [M+H]+ calcd for $C_{23}H_{25}NOPS$ 394.1394; found 394.1390.

N-(4-(tert-butyl)phenyl)-4-(diphenylphosphorothioyl)butanamide (4e)

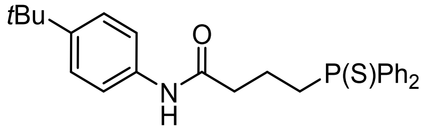

General procedure C was used with N-(4-(tert-butyl)phenyl)but-3-enamide **1e** (47.7mg, 0.22 mmol, 2.2 equiv.) and diphenylphosphane **2a** (18.6 mg, 0.1 mmol, 1.0 equiv.) at

90 °C for 18 h to afford **4e** as colorless oil (37.5 mg, 86% yield). ^{1}H NMR (600 MHz, Chloroform-*d*) δ 7.88-7.79 (m, 4H), 7.63 (s, 1H), 7.50-7.40 (m, 8H), 7.31 (d, *J* = 8.3 Hz, 2H), 2.63-2.56 (m, 2H), 2.50 (t, *J* = 6.8 Hz, 2H), 2.10-2.03 (m, 2H), 1.29 (s, 9H). ^{31}P NMR (243 MHz, Chloroform-*d*) δ 42.40. ^{13}C NMR (151 MHz, Chloroform-*d*) δ 170.37, 147.39, 135.25, 132.57 (d, *J* = 80.2 Hz), 131.69 (d, *J* = 2.9 Hz), 131.19 (d, *J* = 10.2 Hz), 128.84 (d, *J* = 12.0 Hz), 125.88, 119.88, 37.08 (d, *J* = 13.1 Hz), 34.47, 31.47, 31.34 (d, *J* = 56.7 Hz), 18.96 (d, *J* = 2.1 Hz). HRMS (ESI-ion trap) m/z: [M+H]+ calcd for $C_{26}H_{31}NOPS$ 436.1864; found 436.1862.

N-(4-benzylphenyl)-4-(diphenylphosphorothioyl)butanamide (4f)

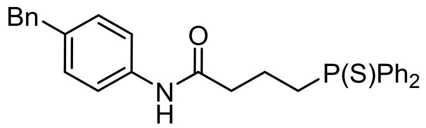

General procedure C was used with N-(4-benzylphenyl)but-3-enamide **1f** (55.2mg, 0.22 mmol, 2.2 equiv.) and diphenylphosphane **2a** (18.6 mg, 0.1 mmol, 1.0 equiv.) at 90 °C for 18 h to afford **4f** as colorless oil (33.6 mg, 72% yield). ^{1}H NMR (600 MHz, Chloroform-*d*) δ 7.87-7.80 (m, 4H), 7.64 (s, 1H), 7.50-7.39 (m, 8H), 7.30-7.26 (m, 2H), 7.22-7.14 (m, 3H), 7.11 (d, *J* = 8.2 Hz, 2H), 3.93 (s, 2H), 2.63-2.55 (m, 2H), 2.49 (t, *J* = 6.8 Hz, 2H), 2.10-2.02 (m, 2H). ^{31}P NMR (243 MHz, Chloroform-*d*) δ 42.38. ^{13}C NMR (151 MHz, Chloroform-*d*) δ 170.40, 141.21, 137.27, 136.02, 132.60 (d, *J* = 80.3 Hz), 131.68 (d, *J* = 3.1 Hz), 131.19 (d, *J* = 10.1 Hz), 129.50, 128.96, 128.83 (d, *J* = 12.1 Hz), 128.56, 126.19, 120.26, 41.46, 37.09 (d, *J* = 12.9 Hz), 31.35 (d, *J* = 56.7 Hz), 18.96. HRMS (ESI-ion trap) m/z: [M+H]+ calcd for $C_{29}H_{29}NOPS$ 470.1707; found 470.1708.

4-(diphenylphosphorothioyl)-N-(4-(trifluoromethyl)phenyl)butanamide (4g)

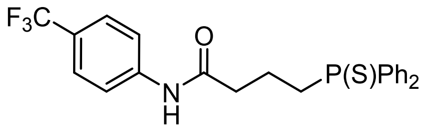

General procedure C was used with N-(4-(trifluoromethyl)phenyl)but-3-enamide **1g** (50.4mg, 0.22 mmol, 2.2 equiv.) and diphenylphosphane **2a** (18.6 mg, 0.1 mmol, 1.0 equiv.) at 90 °C for 18 h to afford **4g** as colorless oil (29.0 mg, 68% yield). ^{1}H NMR (600 MHz, Chloroform-*d*) δ 8.12 (s, 1H), 7.86-7.79 (m, 4H), 7.65 (d, *J* = 8.3 Hz, 2H), 7.54-7.41 (m, 8H), 2.65-2.51 (m, 4H), 2.11-2.03 (m, 2H). ^{31}P NMR (243 MHz, Chloroform-*d*) δ 42.29. ^{13}C NMR (151 MHz, Chloroform-*d*) δ 170.90, 141.08, 132.65, 132.11, 131.84 (d, *J* = 3.0 Hz), 131.16 (d, *J* = 10.0 Hz), 128.91 (d, *J* = 12.1 Hz), 126.28 (q, *J* = 3.8 Hz), 124.20 (q, *J* = 271.6 Hz), 119.49, 37.11 (d, *J* = 12.2 Hz), 31.16 (d, *J* = 56.6 Hz), 18.95 (d, *J* = 2.2 Hz). ^{19}F NMR (565 MHz, Chloroform-*d*) δ -62.08. HRMS (ESI-ion trap) m/z: [M+H]+ calcd for $C_{23}H_{22}F_3NOPS$ 448.1112; found 448.1110.

4-(diphenylphosphorothioyl)-N-(4-fluorophenyl)butanamide (4h)

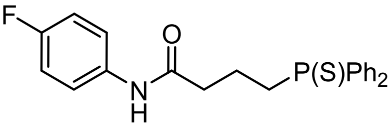

General procedure C was used with N-(4-fluorophenyl)but-3-enamide **1h** (39.4mg, 0.22 mmol, 2.2 equiv.) and diphenylphosphane **2a** (18.6 mg, 0.1 mmol, 1.0 equiv.) at 90 °C for 18 h to afford **4h** as colorless oil (28.5 mg, 72% yield). ^{1}H NMR (600 MHz, Chloroform-*d*) δ 7.87-7.80 (m, 4H), 7.74 (s, 1H), 7.51-7.43 (m, 8H), 6.98 (t, *J* = 8.6 Hz, 2H), 2.63-2.57 (m, 2H), 2.53 (t, *J* = 6.9 Hz, 2H), 2.10-2.03 (m, 2H). ^{31}P NMR (243 MHz, Chloroform-*d*) δ 42.31. ^{13}C NMR (151 MHz, Chloroform-*d*) δ 170.46, 159.45 (d, *J* = 243.5 Hz), 133.95 (d, *J* = 2.9 Hz), 132.49 (d, *J* = 80.4 Hz), 131.78 (d, *J* = 3.0 Hz), 131.19 (d, *J* = 10.0 Hz), 128.89 (d, *J* = 12.2 Hz), 121.79 (d, *J* = 7.8 Hz), 115.70 (d, *J* = 22.4 Hz), 36.96 (d, *J* = 12.5 Hz), 31.22 (d, *J* = 56.8 Hz), 19.01 (d, *J* = 2.3 Hz). ^{19}F NMR (376 MHz, Chloroform-*d*) δ -118.02. HRMS (ESI-ion trap) m/z: [M+H]+ calcd for $C_{22}H_{22}FNOPS$ 398.1144; found 398.1144.

N-(4-chlorophenyl)-4-(diphenylphosphorothioyl)butanamide (4i)

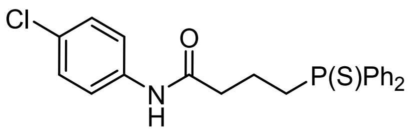

General procedure C was used with N-(4-chlorophenyl)but-3-enamide **1i** (42.9 mg, 0.22 mmol, 2.2 equiv.) and diphenylphosphane **2a** (18.6 mg, 0.1 mmol, 1.0 equiv.) at 90 °C for 18 h to afford **4i** as colorless oil (28.2 mg, 69% yield). ^{1}H NMR (600 MHz, Chloroform-*d*) δ 7.89-7.80 (m, 5H), 7.53-7.44 (m, 8H), 7.29-7.26 (m, 2H), 2.64-2.58 (m, 2H), 2.55 (t, *J* = 6.8 Hz, 2H), 2.12-2.04 (m, 2H). ^{31}P NMR (243 MHz, Chloroform-*d*) δ 42.28. ^{13}C NMR (151 MHz, Chloroform-*d*) δ 170.55, 136.56, 132.42 (d, *J* = 80.5 Hz), 131.80 (d, *J* = 2.9 Hz), 131.18 (d, *J* = 10.3 Hz), 129.28, 129.07, 128.90 (d, *J* = 12.0 Hz), 121.17, 37.04 (d, *J* = 12.2 Hz), 31.16 (d, *J* = 56.7 Hz), 18.98 (d, *J* = 2.2 Hz). HRMS (ESI-ion trap) m/z: [M+H]+ calcd for $C_{22}H_{22}ClNOPS$ 414.0848; found 414.0847.

N-(4-bromophenyl)-4-(diphenylphosphorothioyl)butanamide (4j)

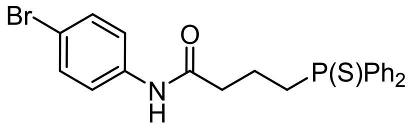

General procedure C was used with N-(4-bromophenyl)but-3-enamide **1j** (52.8 mg, 0.22 mmol, 2.2 equiv.) and diphenylphosphane **2a** (18.6 mg, 0.1 mmol, 1.0 equiv.) at 90 °C for 18 h to afford **4j** as colorless oil (24.0 mg, 53% yield). ^{1}H NMR (600 MHz, Chloroform-*d*) δ 7.87-7.78 (m, 5H), 7.51-7.47 (m, 2H), 7.47-7.38 (m, 8H), 2.62-2.57 (m, 2H), 2.53 (t, *J* = 6.8 Hz, 2H), 2.09-2.02 (m, 2H). ^{31}P NMR (243 MHz, Chloroform-*d*) δ 42.25. ^{13}C NMR (151 MHz, Chloroform-*d*) δ 170.55, 137.07, 132.71, 132.03, 131.81 (d, *J* = 3.0 Hz), 131.18 (d, *J* = 10.3 Hz), 128.91 (d, *J* = 12.0 Hz), 121.48, 116.90, 37.05 (d, *J* = 12.1 Hz), 31.13 (d, *J* = 56.6 Hz), 18.99 (d, *J* = 2.3 Hz). HRMS (ESI-ion trap) m/z: [M+H]+ calcd for $C_{22}H_{22}BrNOPS$ 458.0343; found 458.0344.

4-(diphenylphosphorothioyl)-N-(4-methoxyphenyl)butanamide (4k)

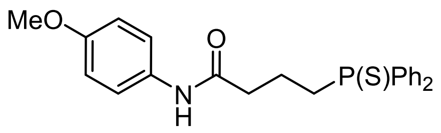

General procedure C was used with N-(4-methoxyphenyl)but-3-enamide **1k** (42.0 mg, 0.22 mmol, 2.2 equiv.) and diphenylphosphane **2a** (18.6 mg, 0.1 mmol, 1.0 equiv.) at 90 °C for 18 h to afford **4k** as colorless oil (31.6 mg, 77% yield). ^{1}H NMR (600 MHz, Chloroform-*d*) δ 7.86-7.80 (m, 4H), 7.64 (s, 1H), 7.49-7.42 (m, 6H), 7.41-7.38 (m, 2H), 6.82 (d, *J* = 9.0 Hz, 2H), 3.77 (s, 3H), 2.64-2.54 (m, 2H), 2.48 (t, *J* = 6.8 Hz, 2H), 2.12-1.97 (m, 2H). ^{31}P NMR (243 MHz, Chloroform-*d*) δ 42.42. ^{13}C NMR (151 MHz, Chloroform-*d*) δ 170.31, 156.48, 132.56 (d, *J* = 80.2 Hz), 131.70 (d, *J* = 3.0 Hz), 131.18 (d, *J* = 10.2 Hz), 131.03, 128.84 (d, *J* = 12.0 Hz), 121.92, 114.20, 55.59, 36.95 (d, *J* = 13.2 Hz), 31.36 (d, *J* = 56.5 Hz), 18.96 (d, *J* = 2.1 Hz). HRMS (ESI-ion trap) m/z: [M+H]+ calcd for $C_{23}H_{25}NO_2PS$ 410.1344; found 410.1341.

4-(diphenylphosphorothioyl)-N-(4-phenoxyphenyl)butanamide (4l)

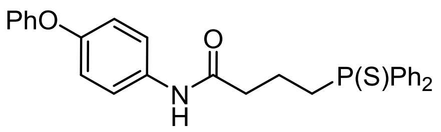

General procedure C was used with N-(4-phenoxyphenyl)but-3-enamide 1l (55.7 mg, 0.22 mmol, 2.2 equiv.) and diphenylphosphane **2a** (18.6 mg, 0.1 mmol, 1.0 equiv.) at 90 °C for 18 h to afford **4l** as colorless oil (38.0 mg, 81% yield). ^{1}H NMR (600 MHz, Chloroform-*d*) δ 7.88-7.80 (m, 4H), 7.69 (s, 1H), 7.51-7.42 (m, 8H), 7.31 (t, *J* = 7.8 Hz, 2H), 7.08 (t, *J* = 7.4 Hz, 1H), 6.99-6.93 (m, 4H), 2.64-2.58 (m, 2H), 2.53 (t, *J* = 6.8 Hz, 2H), 2.11-2.04 (m, 2H). ^{31}P NMR (243 MHz, Chloroform-*d*) δ 42.35. ^{13}C NMR (151 MHz, Chloroform-*d*) δ 170.40, 157.67, 153.53, 133.45, 132.52 (d, *J* = 80.2 Hz), 131.76 (d, *J* = 2.9 Hz), 131.20 (d, *J* = 10.3 Hz), 129.84, 128.88 (d, *J* = 12.1 Hz), 123.18, 121.75, 119.73, 118.51, 37.00 (d, *J* = 12.6 Hz), 31.27 (d, *J* = 56.7 Hz), 19.01 (d, *J* = 2.1 Hz). HRMS (ESI-ion trap) m/z: [M+H]+ calcd for $C_{28}H_{27}NO_2PS$ 472.1500; found 472.1499.

4-(diphenylphosphorothioyl)-N-(4-(trifluoromethoxy)phenyl)butanamide (4m)

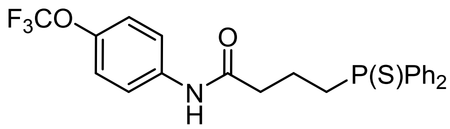

General procedure C was used with N-(4-(trifluoromethoxy)phenyl)but-3-enamide **1m** (53.9 mg, 0.22 mmol, 2.2 equiv.) and diphenylphosphane **2a** (18.6 mg, 0.1 mmol, 1.0 equiv.) at 90 °C for 18 h to afford **4m** as colorless oil (33.2 mg, 72% yield). ^{1}H NMR (600 MHz, Chloroform-*d*) δ 8.02 (s, 1H), 7.82 (dd, *J* = 12.9, 7.6 Hz, 4H), 7.54 (d, *J* = 8.5 Hz, 2H), 7.50-7.41 (m, 6H), 7.13 (d, *J* = 8.5 Hz, 2H), 2.62-2.56 (m, 2H), 2.53 (t, *J* = 6.9 Hz, 2H), 2.09-2.01 (m, 2H). ^{31}P NMR (243 MHz, Chloroform-*d*) δ 42.35. ^{13}C NMR (151 MHz, Chloroform-*d*) δ 170.67, 145.28, 136.66, 132.35 (d, *J* = 80.5 Hz), 131.82 (d, *J* = 2.9 Hz), 131.16 (d, *J* = 10.1 Hz), 128.89 (d, *J* = 12.1 Hz), 121.77, 121.09, 120.57 (q, *J* = 256.7 Hz), 37.00 (d, *J* = 12.5 Hz), 31.18 (d, *J* = 56.7 Hz), 18.96 (d, *J* = 2.2 Hz). ^{19}F NMR (565 MHz, Chloroform-*d*) δ -58.10. HRMS (ESI-ion trap) m/z: [M+H]+ calcd for $C_{23}H_{22}F_3NO_2PS$ 464.1061; found 464.1060.

4-(diphenylphosphorothioyl)-N-(4-vinylphenyl)butanamide (4n)

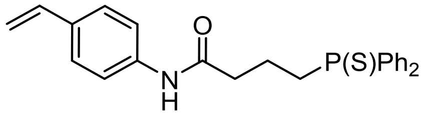

General procedure C was used with N-(4-vinylphenyl)but-3-enamide **1n** (41.1 mg, 0.22 mmol, 2.2 equiv.) and diphenylphosphane **2a** (18.6 mg, 0.1 mmol, 1.0 equiv.) at 90 °C for 18 h to afford **4n** as colorless oil (22.9 mg, 57% yield). ^{1}H NMR (600 MHz, Chloroform-*d*) δ 7.86-7.80 (m, 4H), 7.75 (s, 1H), 7.52-7.41 (m, 8H), 7.34 (d, *J* = 8.1 Hz, 2H), 6.66 (dd, *J* = 17.6, 10.9 Hz, 1H), 5.67 (d, *J* = 17.6 Hz, 1H), 5.19 (d, *J* = 10.8 Hz, 1H), 2.62-2.56 (m, 2H), 2.52 (t, *J* = 6.9 Hz, 2H), 2.10-2.02 (m, 2H).^{31}P NMR (243 MHz, Chloroform-*d*) δ 42.35. ^{13}C NMR (151 MHz, Chloroform-*d*) δ 170.45, 137.51, 136.23, 133.78, 132.50 (d, *J* = 80.2 Hz), 131.74 (d, *J* = 2.9 Hz), 131.18 (d, *J* = 10.2 Hz), 128.86 (d, *J* = 12.0 Hz), 126.90, 119.90, 113.13, 37.13 (d, *J* = 12.8 Hz), 31.25 (d, *J* = 56.6 Hz), 18.95 (d, *J* = 2.6 Hz). HRMS (ESI-ion trap) m/z: [M+H]+ calcd for $C_{24}H_{25}NOPS$ 406.1394; found 406.1390.

N-(3,5-difluorophenyl)-4-(diphenylphosphorothioyl)butanamide (4o)

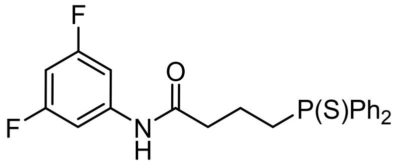

General procedure C was used with N-(3,5-difluorophenyl)but-3-enamide **1o** (43.3 mg, 0.22 mmol, 2.2 equiv.) and diphenylphosphane **2a** (18.6 mg, 0.1 mmol, 1.0 equiv.) at 90 °C for 18 h to afford **4o** as colorless oil (34.6 mg, 84% yield). ^{1}H NMR (600 MHz, Chloroform-*d*) δ 8.12 (s, 1H), 7.86-7.78 (m, 4H), 7.51-7.47 (m, 2H), 7.47-7.42 (m, 4H), 7.20-7.11 (m, 2H), 6.55-6.48 (m, 1H), 2.62-2.52 (m, 4H), 2.07-2.02 (m, 2H). ^{31}P NMR (243 MHz, Chloroform-*d*) δ 42.24. ^{19}F NMR (565 MHz, Chloroform-*d*) δ -108.90. ^{13}C NMR (151 MHz, Chloroform-*d*) δ 170.85, 163.21 (dd, *J* = 246.1, 14.5 Hz), 140.18, 132.30 (d, *J* = 80.8 Hz), 131.86 (d, *J* = 3.1 Hz), 131.16 (d, *J* = 10.0 Hz), 128.92 (d, *J* = 12.0 Hz), 110.86-101.95 (m), 99.45 (t, *J* = 25.6 Hz), 37.02 (d, *J* = 11.9 Hz), 31.06 (d, *J* = 56.4 Hz), 18.92 (d, *J* = 2.3 Hz). HRMS (ESI-ion trap) m/z: [M+H]+ calcd for $C_{22}H_{21}F_2NOPS$ 416.1050; found 416.1047.

4-(diphenylphosphorothioyl)-N-(4-morpholinophenyl)butanamide (4p)

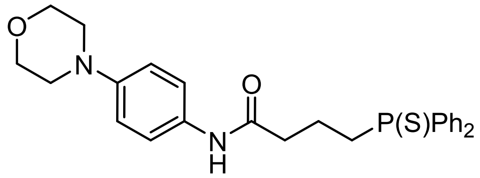

General procedure C was used with **1p** (54.1 mg, 0.22 mmol, 2.2 equiv.) and diphenylphosphane **2a** (18.6 mg, 0.1 mmol, 1.0 equiv.) at 90 °C for 18 h to afford **4p** as colorless oil (18.4 mg, 40% yield). ^{1}H NMR (600 MHz, Chloroform-*d*) δ 7.88-7.77 (m, 4H), 7.66 (s, 1H), 7.48-7.37 (m, 8H), 6.84 (d, *J* = 8.9 Hz, 2H), 3.85-3.82 (m, 4H), 3.11-3.06 (m, 4H), 2.62-2.53 (m, 2H), 2.46 (t, *J* = 6.8 Hz, 2H), 2.07-2.00 (m, 2H). ^{31}P NMR (243 MHz, Chloroform-*d*) δ 42.42. ^{13}C NMR (151 MHz, Chloroform-*d*) δ 170.26, 148.31, 132.58 (d, *J* = 80.2 Hz), 131.66 (d, *J* = 3.0 Hz), 131.16 (d, *J* = 10.0 Hz), 130.70, 128.81 (d, *J* = 12.0 Hz), 121.53, 116.33, 66.96, 49.84, 36.96 (d, *J* = 13.5 Hz), 31.38 (d,

J = 56.7 Hz), 18.95 (d, J = 2.1 Hz). HRMS (ESI-ion trap) m/z: [M+H]+ calcd for $C_{26}H_{30}N_2O_2PS$ 465.1766; found 465.1764.

N-(4-acetylphenyl)-4-(diphenylphosphorothioyl)butanamide (4q)

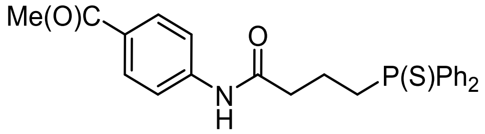

General procedure C was used with N-(4-acetylphenyl)but-3-enamide **1q** (44.7 mg, 0.22 mmol, 2.2 equiv.) and diphenylphosphane **2a** (18.6 mg, 0.1 mmol, 1.0 equiv.) at 90 °C for 18 h to afford **4q** as colorless oil (15.1 mg, 36% yield). ^{1}H NMR (400 MHz, Chloroform-*d*) δ 8.18 (s, 1H), 7.90 (d, J = 8.5 Hz, 2H), 7.87-7.76 (m, 4H), 7.64 (d, J = 8.4 Hz, 2H), 7.53-7.40 (m, 6H), 2.66-2.53 (m, 7H), 2.15-2.03 (m, 2H). ^{31}P NMR (162 MHz, Chloroform-*d*) δ 42.24. ^{13}C NMR (101 MHz, Chloroform-*d*) δ 197.23, 170.91, 142.45, 132.85, 132.35 (d, J = 80.5 Hz), 131.82 (d, J = 3.0 Hz), 131.15 (d, J = 10.2 Hz), 129.82, 128.90 (d, J = 12.1 Hz), 119.00, 37.15 (d, J = 12.4 Hz), 31.12 (d, J = 56.6 Hz), 26.60, 18.91 (d, J = 2.2 Hz). HRMS (ESI-ion trap) m/z: [M+H]+ calcd for C24H24NO2PS 422.1344; found 422.1342.

methyl 4-(4-(diphenylphosphorothioyl)butanamido)benzoate (4r)

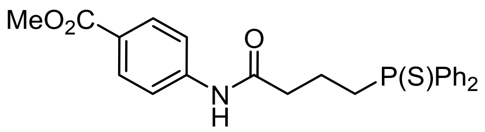

General procedure C was used with methyl 4-(but-3-enamido)benzoate **1r** (48.1 mg, 0.22 mmol, 2.2 equiv.) and diphenylphosphane **2a** (18.6 mg, 0.1 mmol, 1.0 equiv.) at 90 °C for 18 h to afford **4r** as colorless oil (29.5 mg, 68% yield). ^{1}H NMR (600 MHz, Chloroform-*d*) δ 8.01 (s, 1H), 7.98 (d, J = 8.4 Hz, 2H), 7.86-7.79 (m, 4H), 7.61 (d, J = 8.3 Hz, 2H), 7.50-7.42 (m, 6H), 3.89 (s, 3H), 2.63-2.55 (m, 4H), 2.11-2.04 (m, 2H). ^{31}P NMR (243 MHz, Chloroform-*d*) δ 42.22. ^{13}C NMR (151 MHz, Chloroform-*d*) δ 170.81, 166.76, 142.20, 132.42 (d, J = 80.5 Hz), 131.81 (d, J = 3.0 Hz), 131.18 (d, J = 10.2 Hz), 130.91, 128.90 (d, J = 12.2 Hz), 125.63, 118.92, 52.16, 37.16 (d, J = 12.0

Hz), 31.11 (d, J = 56.6 Hz), 18.95 (d, J = 2.3 Hz). HRMS (ESI-ion trap) m/z: [M+H]+ calcd for $C_{24}H_{25}NO3PS$ 438.1293; found 438.1293.

4-(diphenylphosphorothioyl)-N-(naphthalen-2-yl)butanamide (4s)

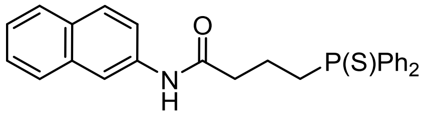

General procedure C was used with N-(naphthalen-2-yl)but-3-enamide 1s (46.4 mg, 0.22 mmol, 2.2 equiv.) and diphenylphosphane **2a** (18.6 mg, 0.1 mmol, 1.0 equiv.) at 90 °C for 18 h to afford **4s** as colorless oil (33.3 mg, 78% yield). ^{1}H NMR (600 MHz, Chloroform-*d*) δ 8.21 (d, J = 2.1 Hz, 1H), 7.90 (s, 1H), 7.84 (dd, J = 13.0, 7.5 Hz, 4H), 7.78-7.73 (m, 3H), 7.50-7.41 (m, 8H), 7.39 (t, J = 7.5 Hz, 1H), 2.65-2.59 (m, 2H), 2.57 (t, J = 6.8 Hz, 2H), 2.14-2.07 (m, 2H). ^{31}P NMR (243 MHz, Chloroform-*d*) δ 42.37. ^{13}C NMR (151 MHz, Chloroform-*d*) δ 170.72, 135.41, 133.92, 132.54 (d, J = 80.3 Hz), 131.74 (d, J = 2.9 Hz), 131.19 (d, J = 10.2 Hz), 130.72, 128.91, 128.83, 127.77, 127.66, 126.59, 125.10, 120.01, 116.70, 37.19 (d, J = 12.6 Hz), 31.27 (d, J = 56.6 Hz), 19.01 (d, J = 2.2 Hz). HRMS (ESI-ion trap) m/z: [M+H]+ calcd for $C_{26}H_{25}NOPS$ 430.1394; found 430.1397.

4-(diphenylphosphorothioyl)-N-(isoquinolin-4-yl)butanamide (4t)

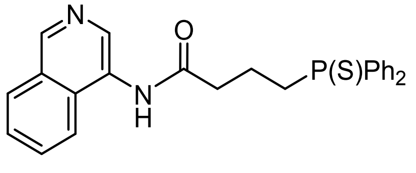

General procedure C was used with **1u** (mg, 0.22 mmol, 2.2 equiv.) and diphenylphosphane **2a** (18.6 mg, 0.1 mmol, 1.0 equiv.) at 90 °C for 18 h to afford **4t** as colorless oil (29.5 mg, 69% yield). ^{1}H NMR (600 MHz, Chloroform-*d*) δ 9.03 (s, 1H), 8.76 (s, 1H), 8.37 (s, 1H), 7.92 (d, J = 8.2 Hz, 1H), 7.88-7.79 (m, 5H), 7.63 (t, J = 7.7 Hz, 1H), 7.57 (t, J = 7.6 Hz, 1H), 7.48-7.40 (m, 6H), 2.71 (t, J = 7.0 Hz, 2H), 2.64 (d, J = 9.3 Hz, 2H), 2.16-2.08 (m, 2H). ^{31}P NMR (243 MHz, Chloroform-*d*) δ 42.31. ^{13}C NMR (151 MHz, Chloroform-*d*) δ 171.66, 150.06, 138.58, 132.49 (d, J = 80.4 Hz), 131.74, 131.15 (d, J = 10.3 Hz), 130.79, 130.62, 128.90, 128.82, 128.12, 128.06,

127.57, 121.14, 36.59 (d, *J* = 12.6 Hz), 31.27 (d, *J* = 56.7 Hz), 19.14. HRMS (ESI-ion trap) m/z: [M+H]+ calcd for $C_{25}H_{23}N2OPS$ 431.1347; found 431.1345.

4-(diphenylphosphorothioyl)-N-(thiophen-3-yl)butanamide (4u)

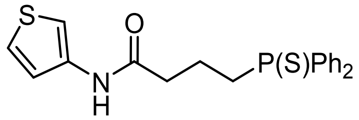

General procedure C was used with N-(thiophen-3-yl)but-3-enamide **1v** (36.7 mg, 0.22 mmol, 2.2 equiv.) and diphenylphosphane **2a** (18.6 mg, 0.1 mmol, 1.0 equiv.) at 90 ℃ for 18 h to afford **4u** as colorless oil (15.2 mg, 40% yield). ^{1}H NMR (600 MHz, Chloroform-*d*) δ 7.98 (s, 1H), 7.85-7.80 (m, 4H), 7.55 (d, *J* = 3.1 Hz, 1H), 7.51-7.43 (m, 6H), 7.21-7.19 (m, 1H), 7.00 (d, *J* = 5.2 Hz, 1H), 2.62-2.56 (m, 2H), 2.51 (t, *J* = 6.9 Hz, 2H), 2.10-2.03 (m, 2H). ^{31}P NMR (243 MHz, Chloroform-*d*) δ 42.36. ^{13}C NMR (151 MHz, Chloroform-*d*) δ 169.66, 135.56, 132.50 (d, *J* = 80.5 Hz), 131.77 (d, *J* = 2.9 Hz), 131.20 (d, *J* = 10.1 Hz), 128.89 (d, *J* = 12.1 Hz), 124.60, 121.18, 110.35, 36.59 (d, *J* = 12.5 Hz), 31.24 (d, *J* = 56.6 Hz), 18.99 (d, *J* = 2.2 Hz). HRMS (ESI-ion trap) m/z: [M+H]+ calcd for $C_{20}H_{21}NOPS_2$ 386.0802; found 386.0798.

N-benzyl-4-(diphenylphosphorothioyl)butanamide (4v)

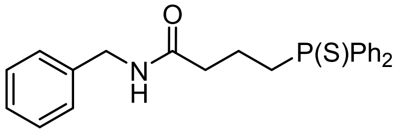

General procedure C was used with N-benzylbut-3-enamide 1y (38.5 mg, 0.22 mmol, 2.2 equiv.) and diphenylphosphane **2a** (18.6 mg, 0.1 mmol, 1.0 equiv.) at 90 ℃ for 18 h to afford **4v** as colorless oil (32.8 mg, 83% yield). ^{1}H NMR (600 MHz, Chloroform-*d*) δ 7.87-7.80 (m, 4H), 7.52-7.44 (m, 6H), 7.34-7.29 (m, 2H), 7.29-7.25 (m, 3H), 6.10 (t, *J* = 5.9 Hz, 1H), 4.41 (d, *J* = 5.8 Hz, 2H), 2.58-2.51 (m, 2H), 2.36 (t, *J* = 6.9 Hz, 2H), 2.04-1.98 (m, 2H). ^{31}P NMR (243 MHz, Chloroform-*d*) δ 42.41. ^{13}C NMR (151 MHz, Chloroform-*d*) δ 171.88, 138.32, 132.65 (d, *J* = 80.2 Hz), 131.62 (d, *J* = 2.9 Hz), 131.15 (d, *J* = 10.0 Hz), 128.80 (d, *J* = 5.0 Hz), 128.74, 127.92, 127.59, 43.63, 36.27 (d, *J* =

14.3 Hz), 31.55 (d, *J* = 56.7 Hz), 18.89 (d, *J* = 2.1 Hz). HRMS (ESI-ion trap) m/z: [M+H]+ calcd for $C_{23}H_{25}NOPS$ 394.1394; found 394.1390.

methyl (S)-2-(4-(diphenylphosphorothioyl)butanamido)-2-phenylacetate (4w)

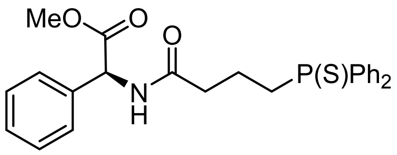

General procedure C was used with **1ae** (37.6 mg, 0.22 mmol, 2.2 equiv.) and diphenylphosphane **2a** (18.6 mg, 0.1 mmol, 1.0 equiv.) at 90 ℃ for 18 h to afford **4w** as colorless oil (mg, 64% yield). ^{1}H NMR (600 MHz, Chloroform-*d*) δ 7.85-7.75 (m, 4H), 7.50-7.40 (m, 6H), 7.37-7.31 (m, 5H), 6.61 (d, *J* = 7.1 Hz, 1H), 5.54 (d, *J* = 7.1 Hz, 1H), 3.71 (d, *J* = 1.2 Hz, 3H), 2.58-2.48 (m, 2H), 2.43-2.33 (m, 2H), 2.02-1.92 (m, 2H). ^{31}P NMR (243 MHz, Chloroform-*d*) δ 42.33. ^{13}C NMR (151 MHz, Chloroform-*d*) δ 171.48 (d, *J* = 15.2 Hz), 136.39, 132.71 (dd, *J* = 80.1, 21.2 Hz), 131.57 (d, *J* = 2.7 Hz), 131.19 (d, *J* = 10.2 Hz), 129.14, 128.76 (dd, *J* = 11.2, 3.6 Hz), 128.72, 127.46, 56.61, 52.92, 35.92 (d, *J* = 14.3 Hz), 31.34 (d, *J* = 56.9 Hz), 18.77 (d, *J* = 2.0 Hz). HRMS (ESI-ion trap) m/z: [M+H]+ calcd for $C_{25}H_{27}O_3PS$ 452.1449; found 452.1447.

methyl (4-(diphenylphosphorothioyl)butanoyl)-L-alaninate (4x)

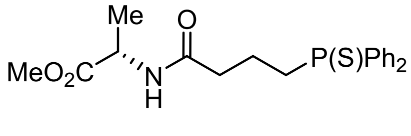

General procedure C was used with methyl but-3-enoyl-*L*-alaninate 1aa (37.6 mg, 0.22 mmol, 2.2 equiv.) and diphenylphosphane **2a** (18.6 mg, 0.1 mmol, 1.0 equiv.) at 90 ℃ for 18 h to afford **4x** as colorless oil (28.9 mg, 75% yield). ^{1}H NMR (600 MHz, Chloroform-*d*) δ 7.83 (dd, *J* = 12.9, 7.4 Hz, 4H), 7.51-7.38 (m, 6H), 6.20 (d, *J* = 7.4 Hz, 1H), 4.57-4.50 (m, 1H), 3.71 (s, 3H), 2.60-2.53 (m, 2H), 2.38-2.30 (m, 2H), 2.02-1.92 (m, 2H), 1.37 (d, *J* = 7.6 Hz, 3H). ^{31}P NMR (243 MHz, Chloroform-*d*) δ 42.44. ^{13}C NMR (151 MHz, Chloroform-*d*) δ 173.52, 171.66, 132.69 (dd, *J* = 80.2, 55.5 Hz), 131.58 (dd, *J* = 4.3 Hz, 3.1 Hz), 131.17 (dd, *J* = 11.4 Hz, 10.6 Hz), 128.75 (d, *J* = 12.0 Hz), 52.52, 48.09, 36.02 (d, *J* = 14.6 Hz), 31.44 (d, *J* = 56.8 Hz), 18.72 (d, *J* = 2.0 Hz),

18.32. HRMS (ESI-ion trap) m/z: [M+H]+ calcd for $C_{20}H_{24}NO_3PS$ 390.1293; found 390.1287.

5-(diphenylphosphorothioyl)-N-phenylpentanamide (4y)

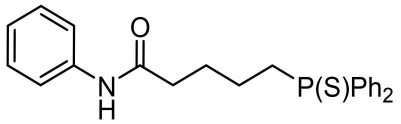

General procedure C was used with N-phenylpent-4-enamide **1af** (38.5 mg, 0.22 mmol, 2.2 equiv.) and diphenylphosphane **2a** (18.6 mg, 0.1 mmol, 1.0 equiv.) at 90 °C for 18 h to afford **4y** as colorless oil (18.3 mg, 46% yield). ^{1}H NMR (400 MHz, Chloroform-*d*) δ 7.85 (s, 1H), 7.83-7.73 (m, 4H), 7.54-7.36 (m, 8H), 7.28-7.20 (m, 2H), 7.05 (t, *J* = 7.4 Hz, 1H), 2.50-2.37 (m, 2H), 2.27 (t, *J* = 7.4 Hz, 2H), 1.83-1.61 (m, 4H). ^{31}P NMR (162 MHz, Chloroform-*d*) δ 42.72. ^{13}C NMR (101 MHz, Chloroform-*d*) δ 171.12, 138.00, 132.55 (d, *J* = 80.0 Hz), 131.61 (d, *J* = 3.0 Hz), 131.04 (d, *J* = 10.1 Hz), 128.92, 128.75 (d, *J* = 12.0 Hz), 124.21, 120.07, 36.98, 32.12 (d, *J* = 56.6 Hz), 26.37 (d, *J* = 16.7 Hz), 21.98 (d, *J* = 2.5 Hz). HRMS (ESI-ion trap) m/z: [M+H]+ calcd for $C_{23}H_{25}NOPS$ 394.1394; found 394.1390.

4-(di-p-tolylphosphorothioyl)-N-phenylbutanamide (4z)

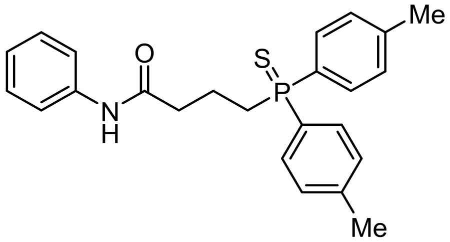

General procedure C was used with N-phenylbut-3-enamide **1a** (35.4 mg, 0.22 mmol, 2.2 equiv.) and di-*p*-tolylphosphane **2b** (21.4 mg, 0.1 mmol, 1.0 equiv.) at 90 °C for 18 h to afford **4z** as colorless oil (16.4 mg, 40% yield). ^{1}H NMR (600 MHz, Chloroform-*d*) δ 7.74-7.67 (m, 4H), 7.58 (s, 1H), 7.51 (d, *J* = 8.0 Hz, 2H), 7.31 (t, *J* = 7.7 Hz, 2H), 7.26-7.22 (m, 4H), 7.10 (t, *J* = 7.5 Hz, 1H), 2.61-2.51 (m, 4H), 2.37 (s, 6H), 2.11-2.04 (m, 2H). ^{31}P NMR (243 MHz, Chloroform-*d*) δ 41.57. ^{13}C NMR (151 MHz, Chloroform-*d*) δ 170.49, 142.19 (d, *J* = 2.9 Hz), 137.98, 131.20 (d, *J* = 10.4 Hz), 129.59

(d, J = 12.5 Hz), 129.47 (d, J = 82.8 Hz), 129.10, 124.37, 119.91, 37.15 (d, J = 11.7 Hz), 31.34 (d, J = 57.0 Hz), 21.58, 19.12 (d, J = 2.3 Hz). HRMS (ESI-ion trap) m/z: [M+H]+ calcd for $C_{24}H_{27}NOPS$ 408.1551; found 408.1547.

4-(di-m-tolylphosphorothioyl)-N-phenylbutanamide (4aa)

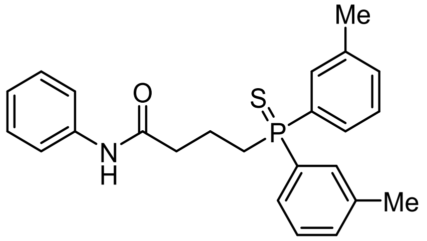

General procedure D was used with N-phenylbut-3-enamide **1a** (35.4 mg, 0.22 mmol, 2.2 equiv.) and di-*m*-tolylphosphane prepared through reduction of di-*m*-tolylphosphine oxide **2d** (23.0 mg, 0.1 mmol, 1.0 equiv.) *in situ* by $PhSiH_3$ (10.8 mg, 0.1 mmol, 1.0 equiv.) at 100 ℃ for 18 h to afford **4aa** as colorless oil (18.5 mg, 46% yield). ^{1}H NMR (400 MHz, Chloroform-*d*) δ 7.76-7.45 (m, 7H), 7.38-7.26 (m, 6H), 7.09 (t, J = 7.4 Hz, 1H), 2.66-2.49 (m, 4H), 2.36 (s, 6H), 2.14-2.00 (m, 2H). ^{31}P NMR (162 MHz, Chloroform-*d*) δ 42.25. ^{13}C NMR (101 MHz, Chloroform-*d*) δ 170.51, 138.84 (d, J = 12.1 Hz), 137.95, 132.55 (d, J = 3.0 Hz), 132.42 (d, J = 79.9 Hz), 131.81 (d, J = 10.3 Hz), 129.09, 128.65 (d, J = 12.7 Hz), 128.07 (d, J = 10.1 Hz), 124.38, 119.91, 37.17 (d, J = 12.4 Hz), 31.14 (d, J = 56.6 Hz), 21.61, 19.04 (d, J = 2.2 Hz).HRMS (ESI-ion trap) m/z: [M+H]+ calcd for $C_{24}H_{27}NOPS$ 408.1551; found 408.1546.

4-(bis(4-(tert-butyl)phenyl)phosphorothioyl)-N-phenylbutanamide (4ab)

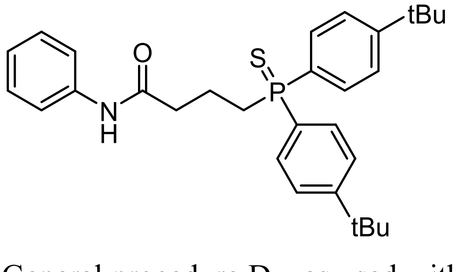

General procedure D was used with N-phenylbut-3-enamide **1a** (35.4 mg, 0.22 mmol, 2.2 equiv.) and bis(4-(tert-butyl)phenyl)phosphane prepared through reduction of bis(4-(tert-butyl)phenyl)phosphine oxide **2e** (31.4 mg, 0.1 mmol, 1.0 equiv.) *in situ* by DEMS

(13.4 mg, 0.1 mmol, 1.0 equiv.) at 100 ℃ for 18 h to afford **4ab** as colorless oil (32.6 mg, 67% yield). ^{1}H NMR (600 MHz, Chloroform-*d*) δ 7.79-7.73 (m, 4H), 7.67 (s, 1H), 7.53 (d, *J* = 8.0 Hz, 2H), 7.46 (dd, *J* = 8.6, 2.6 Hz, 4H), 7.31 (t, *J* = 7.7 Hz, 2H), 7.09 (t, *J* = 7.4 Hz, 1H), 2.61-2.54 (m, 4H), 2.13-2.05 (m, 2H), 1.32-1.28 (m, 18H). ^{31}P NMR (243 MHz, Chloroform-*d*) δ 41.14. ^{13}C NMR (151 MHz, Chloroform-*d*) δ 170.58, 155.12 (d, *J* = 2.8 Hz), 137.99, 131.08 (d, *J* = 10.6 Hz), 129.27 (d, *J* = 82.6 Hz), 129.10, 125.89 (d, *J* = 12.4 Hz), 124.38, 119.94, 37.31 (d, *J* = 12.2 Hz), 35.06, 31.47 (d, *J* = 57.0 Hz), 31.22, 19.19 (d, *J* = 2.1 Hz). HRMS (ESI-ion trap) m/z: [M+H]+ calcd for $C_{30}H_{39}NOPS$ 492.2490; found 492.2487.

4-(bis(3-methoxyphenyl)phosphorothioyl)-N-phenylbutanamide (4ac)

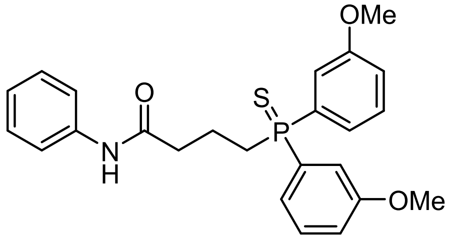

General procedure D was used with N-phenylbut-3-enamide **1a** (35.4 mg, 0.22 mmol, 2.2 equiv.) and bis(3-methoxyphenyl)phosphane prepared through reduction of bis(3-methoxyphenyl)phosphine oxide **2l** (26.2 mg, 0.1 mmol, 1.0 equiv.) *in situ* by $PhSiH_3$ (10.8 mg, 0.1 mmol, 1.0 equiv.) at 100 ℃ for 18 h to afford **4ac** as colorless oil (28.1 mg, 64% yield). ^{1}H NMR (600 MHz, Chloroform-*d*) δ 7.56-7.47 (m, 3H), 7.46-7.41 (m, 2H), 7.37-7.33 (m, 4H), 7.30 (t, *J* = 7.9 Hz, 2H), 7.09 (t, *J* = 7.4 Hz, 1H), 7.03-6.97 (m, 2H), 3.81 (s, 6H), 2.61-2.56 (m, 2H), 2.53 (t, *J* = 6.8 Hz, 2H), 2.12-2.06 (m, 2H). ^{31}P NMR (243 MHz, Chloroform-*d*) δ 43.01. ^{13}C NMR (151 MHz, Chloroform-*d*) δ 170.38, 159.83 (d, *J* = 15.2 Hz), 137.93, 133.97 (d, *J* = 79.8 Hz), 130.00 (d, *J* = 14.1 Hz), 129.11, 124.41, 123.07 (d, *J* = 9.8 Hz), 119.91, 117.68 (d, *J* = 2.7 Hz), 116.62 (d, *J* = 12.0 Hz), 55.59, 37.11 (d, *J* = 12.4 Hz), 31.29 (d, *J* = 56.7 Hz), 19.05. HRMS (ESI-ion trap) m/z: [M+H]+ calcd for $C_{24}H_{27}O_3PS$ 440.1449; found 440.1447.

4-(bis(4-fluorophenyl)phosphorothioyl)-N-phenylbutanamide (4ad)

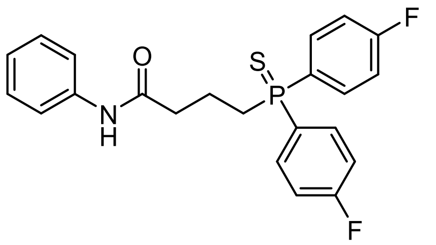

General procedure C was used with N-phenylbut-3-enamide **1a** (35.4 mg, 0.22 mmol, 2.2 equiv.) and bis(4-fluorophenyl)phosphane **2k** (21.4 mg, 0.1 mmol, 1.0 equiv.) at 100 °C for 18 h to afford **4ad** as colorless oil (17.5 mg, 42% yield). ^{1}H NMR (600 MHz, Chloroform-*d*) δ 7.86-7.80 (m, 4H), 7.56 (s, 1H), 7.52-7.46 (m, 2H), 7.31 (t, *J* = 7.9 Hz, 2H), 7.16-7.08 (m, 5H), 2.60-2.55 (m, 2H), 2.53 (t, *J* = 6.7 Hz, 2H), 2.08-2.01 (m, 2H). ^{31}P NMR (243 MHz, Chloroform-*d*) δ 41.14. ^{19}F NMR (565 MHz, Chloroform-*d*) δ -107.13. ^{13}C NMR (151 MHz, Chloroform-*d*) δ 170.32, 164.98 (dd, *J* = 253.9, 3.2 Hz), 137.80, 133.64 (dd, *J* = 11.9, 8.8 Hz), 129.16, 128.39 (dd, *J* = 83.4, 3.4 Hz), 124.56, 120.00, 116.25 (dd, *J* = 21.5, 13.3 Hz), 36.91 (d, *J* = 13.8 Hz), 31.69 (d, *J* = 57.5 Hz), 18.76. HRMS (ESI-ion trap) m/z: [M+H]+ calcd for C22H20F2NOPS 416.1050; found 416.1050

4-(bis(4-chlorophenyl)phosphorothioyl)-N-phenylbutanamide (4ae)

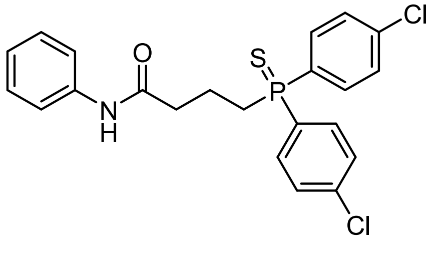

General procedure D was used with N-phenylbut-3-enamide **1a** (35.4 mg, 0.22 mmol, 2.2 equiv.) and bis(4-chlorophenyl)phosphane prepared through reduction of bis(4-chlorophenyl)phosphine oxide **2f** (27.1 mg, 0.1 mmol, 1.0 equiv.) *in situ* by $PhSiH_3$ (10.8 mg, 0.1 mmol, 1.0 equiv.) at 100 °C for 18 h to afford **4ae** as colorless oil (30.7 mg, 69% yield). ^{1}H NMR (600 MHz, Chloroform-*d*) δ 7.79-7.73 (m, 4H), 7.48 (d, *J* = 8.0 Hz, 2H), 7.46-7.38 (m, 5H), 7.32 (t, *J* = 7.7 Hz, 2H), 7.11 (t, *J* = 7.4 Hz, 1H), 2.62-2.56 (m, 2H), 2.53 (t, *J* = 6.7 Hz, 2H), 2.08-2.03 (m, 2H). ^{31}P NMR (243 MHz, Chloroform-*d*) δ 41.48. ^{13}C NMR (151 MHz, Chloroform-*d*) δ 170.19, 138.64 (d, *J* =

3.5 Hz), 137.76, 132.56 (d, J = 11.2 Hz), 130.97 (d, J = 81.5 Hz), 129.27 (d, J = 12.7 Hz), 129.20, 124.61, 120.00, 36.85 (d, J = 13.7 Hz), 31.32 (d, J = 57.3 Hz), 18.71. HRMS (ESI-ion trap) m/z: [M+H]+ calcd for $C_{22}H_{21}Cl_2NOPS$ 448.0459; found 448.0456.

4-(bis(3-chlorophenyl)phosphorothioyl)-N-phenylbutanamide (4af)

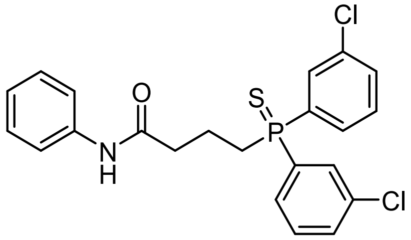

General procedure D was used with N-phenylbut-3-enamide **1a** (35.4 mg, 0.22 mmol, 2.2 equiv.) and bis(3-chlorophenyl)phosphane prepared through reduction of bis(3-chlorophenyl)phosphine oxide **2g** (27.1 mg, 0.1 mmol, 1.0 equiv.) *in situ* by DEMS (13.4 mg, 0.1 mmol, 1.0 equiv.) at 100 ℃ for 18 h to afford **4af** as colorless oil (38.3 mg, 86% yield). ^{1}H NMR (600 MHz, Chloroform-*d*) δ 7.88-7.81 (m, 2H), 7.69-7.65 (m, 2H), 7.55 (s, 1H), 7.51-7.44 (m, 4H), 7.41-7.36 (m, 2H), 7.32-7.27 (m, 2H), 7.09 (t, J = 7.5 Hz, 1H), 2.63-2.56 (m, 2H), 2.52 (t, J = 6.7 Hz, 2H), 2.08-2.01 (m, 2H). ^{31}P NMR (243 MHz, Chloroform-*d*) δ 41.81. ^{13}C NMR (151 MHz, Chloroform-*d*) δ 170.22, 137.78, 135.48 (d, J = 15.7 Hz), 134.63 (d, J = 78.4 Hz), 132.13 (d, J = 2.7 Hz), 131.21 (d, J = 11.5 Hz), 130.30 (d, J = 12.9 Hz), 129.13, 128.97 (d, J = 9.5 Hz), 124.54, 120.03, 36.88 (d, J = 13.8 Hz), 31.09 (d, J = 57.2 Hz), 18.64. HRMS (ESI-ion trap) m/z: [M+H]+ calcd for $C_{22}H_{21}Cl_2NOPS$ 448.0459; found 448.0456.

4-(bis(4-(trifluoromethoxy)phenyl)phosphorothioyl)-N-phenylbutanamide (4ag)

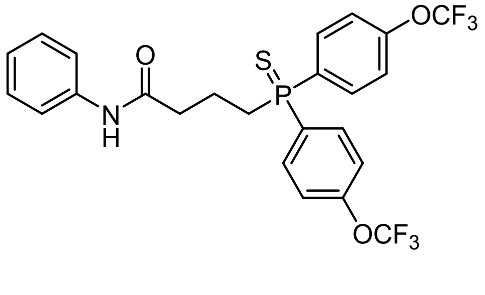

General procedure D was used with N-phenylbut-3-enamide **1a** (35.4 mg, 0.22 mmol, 2.2 equiv.) and bis(4-(trifluoromethoxy)phenyl)phosphane prepared through reduction of bis(4-(trifluoromethoxy)phenyl)phosphine oxide **2h** (37.0 mg, 0.1 mmol, 1.0 equiv.) *in situ* by $PhSiH_3$ (10.8 mg, 0.1 mmol, 1.0 equiv.) at 100 °C for 18 h to afford **4ag** as colorless oil (42.5 mg, 78% yield). ^{1}H NMR (600 MHz, Chloroform-*d*) δ 7.93-7.86 (m, 4H), 7.52-7.45 (m, 3H), 7.34-7.27 (m, 6H), 7.11 (t, J = 7.4 Hz, 1H), 2.65-2.58 (m, 2H), 2.54 (t, J = 6.6 Hz, 2H), 2.11-2.03 (m, 2H). ^{31}P NMR (243 MHz, Chloroform-*d*) δ 41.12. ^{13}C NMR (151 MHz, Chloroform-*d*) δ 170.23, 151.95, 137.75, 133.20 (d, J = 11.5 Hz), 130.89 (d, J = 81.6 Hz), 129.19, 124.64, 120.89 (d, J = 13.0 Hz), 120.41 (q, J = 259.1 Hz), 120.04, 36.85 (d, J = 14.5 Hz), 31.53 (d, J = 57.4 Hz), 18.66. ^{19}F NMR (565 MHz, Chloroform-*d*) δ -57.63, -57.64. HRMS (ESI-ion trap) m/z: [M+H]+ calcd for $C_{24}H_{21}F_6NO_3PS$ 548.0884; found 548.0880.

4-(di(naphthalen-2-yl)phosphorothioyl)-N-phenylbutanamide (4ah)

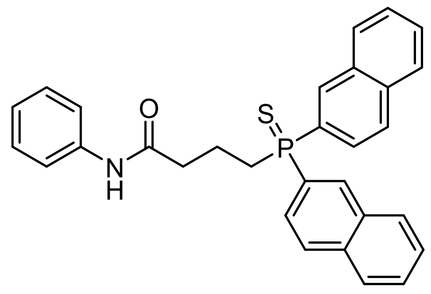

General procedure D was used with N-phenylbut-3-enamide **1a** (35.4 mg, 0.22 mmol, 2.2 equiv.) and di(naphthalen-2-yl)phosphane prepared through reduction of di(naphthalen-2-yl)phosphine oxide **2j** (30.2 mg, 0.1 mmol, 1.0 equiv.) *in situ* by $PhSiH_3$ (10.8 mg, 0.1 mmol, 1.0 equiv.) at 100 °C for 18 h to afford **4ah** as colorless oil (27.1 mg, 57% yield). ^{1}H NMR (600 MHz, Chloroform-*d*) δ 8.52 (dd, J = 15.1, 1.6 Hz, 2H), 7.92 (d, J = 8.0 Hz, 2H), 7.89-7.83 (m, 4H), 7.79-7.74 (m, 2H), 7.61-7.52 (m, 5H), 7.51-7.45 (m, 2H), 7.28 (t, J = 7.9 Hz, 2H), 7.08 (t, J = 7.5 Hz, 1H), 2.84-2.78 (m, 2H), 2.56 (t, J = 6.8 Hz, 2H), 2.19-2.11 (m, 2H). ^{31}P NMR (243 MHz, Chloroform-*d*) δ 42.61. ^{13}C NMR (151 MHz, Chloroform-*d*) δ 170.46, 137.90, 134.58 (d, J = 2.5 Hz), 133.22 (d, J = 10.3 Hz), 132.65 (d, J = 13.5 Hz), 129.66 (d, J = 80.7 Hz), 129.12, 129.09, 128.83 (d, J = 11.8 Hz), 128.42, 127.89, 127.22, 125.98 (d, J = 10.8 Hz), 124.39,

119.94, 37.07 (d, J = 12.4 Hz), 30.94 (d, J = 56.9 Hz), 19.08 (d, J = 2.2 Hz). HRMS (ESI-ion trap) m/z: [M+H]+ calcd for $C_{30}H_{27}NOPS$ 480.1551; found 480.1550.

(*R*)-diphenyl(4-(phenylamino)butan-2-yl)phosphine sulfide (5)

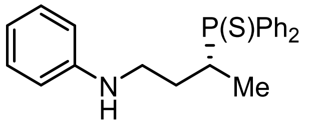

^{1}H NMR (600 MHz, Chloroform-*d*) δ 8.05-7.84 (m, 4H), 7.53-7.34 (m, 6H), 7.14 (t, J = 7.7 Hz, 2H), 6.71 (t, J = 7.4 Hz, 1H), 6.52 (d, J = 7.9 Hz, 2H), 3.53 (s, 1H), 3.29-3.21 (m, 1H), 3.18-3.11 (m, 1H), 3.00-2.92 (m, 1H), 1.97-1.87 (m, 1H), 1.78-1.68 (m, 1H), 1.20 (dd, J = 19.2, 6.8 Hz, 3H). ^{31}P NMR (243 MHz, Chloroform-*d*) δ 52.86. ^{13}C NMR (151 MHz, Chloroform-*d*) δ 147.98, 131.93 (d, J = 39.1 Hz), 131.52 (d, J = 10.4 Hz), 131.49 (dd, J = 9.4, 2.8 Hz), 131.29, 129.43, 128.72 (t, J = 11.0 Hz), 117.72, 112.93, 41.38 (d, J = 14.4 Hz), 30.62 (d, J = 56.9 Hz), 29.68, 12.82. $[\alpha]^{20}_{D}$= -2.9 (c 0.77, $CHCl_3$). The enantiomeric excess was determined by Daicel Chiralcel IF (0.46 cm x 25 cm), Hexanes /IPA = 85 / 15, 1.0 mL/min, λ = 254 nm, t (minor) = 14.3 min, t (major) = 18.1 min.HRMS (ESI-ion trap) m/z: [M+H]+ calcd for $C_{22}H_{25}NPS$ 366.1445; found 366.1441.

(*R*)-diphenyl(4-(phenyl(pyridin-4-yl)amino)butan-2-yl)phosphine sulfide (6)

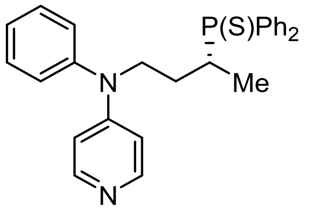

^{1}H NMR (600 MHz, Chloroform-*d*) δ 8.09-8.04 (m, 2H), 7.93-7.86 (m, 4H), 7.50-7.37 (m, 8H), 7.28 (t, J = 7.4 Hz, 1H), 7.09-7.05 (m, 2H), 6.31-6.25 (m, 2H), 3.84-3.78 (m, 1H), 3.66-3.61 (m, 1H), 2.82-2.75 (m, 1H), 2.03-1.98 (m, 1H), 1.83-1.76 (m, 1H), 1.17 (dd, J = 19.0, 6.8 Hz, 3H). ^{31}P NMR (243 MHz, Chloroform-*d*) δ 52.21. ^{13}C NMR (151 MHz, Chloroform-*d*) δ 153.26, 149.67, 144.76, 131.69 (dd, J = 20.4, 3.1 Hz), 131.43 (dd, J = 9.5, 2.6 Hz), 131.25 (d, J = 8.4 Hz), 130.34, 128.80 (dd, J = 11.8, 8.0 Hz),

127.56, 127.02, 108.41, 50.03 (d, J = 14.3 Hz), 31.26 (d, J = 56.4 Hz), 27.63, 13.09. $[\alpha]^{20}_{D}$= 6.0 (c 1.1, $CHCl_3$). The enantiomeric excess was determined by Daicel Chiralcel OD-H *2 (0.46 cm x 25 cm), Hexanes /IPA = 80 / 20, 0.5 mL/min, λ = 254 nm, t (minor) = 38.2 min, t (major) = 41.2 min. HRMS (ESI-ion trap) m/z: [M+H]+ calcd for $C_{27}H_{28}N_2PS$ 443.1711; found 443.1707.

(R)-1-(3-(diphenylphosphorothioyl)butyl)-1-phenyl-3-(4-(trifluoromethyl)phenyl) thiourea (7)

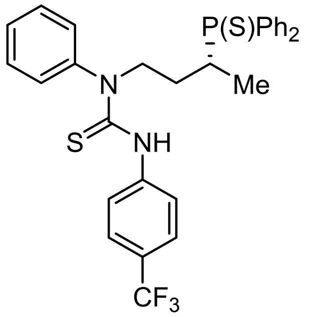

^{1}H NMR (600 MHz, Chloroform-*d*) δ 8.05-7.95 (m, 2H), 7.94-7.87 (m, 2H), 7.53 (d, J = 8.4 Hz, 2H), 7.51-7.36 (m, 11H), 7.05 (s, 1H), 6.90 (s, 1H), 4.82-4.74 (m, 1H), 3.94-3.87 (m, 1H), 3.12-3.03 (m, 1H), 2.14-2.05 (m, 1H), 1.87-1.77 (m, 1H), 1.19 (dd, J = 19.2, 6.8 Hz, 3H). ^{31}P NMR (243 MHz, Chloroform-*d*) δ 52.82. ^{13}C NMR (151 MHz, Chloroform-*d*) δ 180.60, 142.24, 140.86, 131.98 (d, J = 37.3 Hz), 131.70 (d, J = 9.4 Hz), 131.56 (d, J = 9.6 Hz), 131.34, 131.08, 129.44, 128.76 (dd, J = 18.0, 11.8 Hz), 127.80, 127.48 (d, J = 32.7 Hz), 125.79 (q, J = 3.4 Hz), 124.09 (q, J = 271.8 Hz), 124.82, 53.38 (d, J = 16.4 Hz), 31.18 (d, J = 56.3 Hz), 28.22, 13.10. ^{19}F NMR (565 MHz, Chloroform-*d*) δ -62.28. $[\alpha]^{20}_{D}$= -24.0 (c 0.6, $CHCl_3$). The enantiomeric excess was determined by Daicel Chiralcel IF (0.46 cm x 25 cm), Hexanes /IPA = 85 / 15, 1.0 mL/min, λ = 254 nm, t (major) = 13.5 min, t (minor) = 15.5 min. HRMS (ESI-ion trap) m/z: [M+H]+ calcd for $C_{30}H_{29}F_3N_2PS_2$ 569.1462; found 569.1461.

diphenyl(4-(phenylamino)butyl)phosphine sulfide (8)

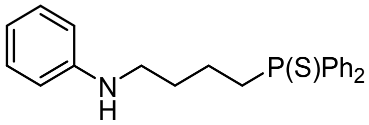

^1H NMR (400 MHz, Chloroform-*d*) δ 7.89-7.78 (m, 4H), 7.55-7.41 (m, 6H), 7.20-7.12 (m, 2H), 6.69 (t, *J* = 7.3 Hz, 1H), 6.60-6.52 (m, 2H), 3.11 (t, *J* = 6.4 Hz, 2H), 2.54-2.44 (m, 2H), 1.79-1.67 (m, 4H). ^{31}P NMR (162 MHz, Chloroform-*d*) δ 42.62. ^{13}C NMR (101 MHz, Chloroform-*d*) δ 148.22, 132.81 (d, *J* = 79.9 Hz), 131.62 (d, *J* = 2.9 Hz), 131.17 (d, *J* = 9.9 Hz), 129.34, 128.79 (d, *J* = 12.0 Hz), 117.39, 112.84, 43.35, 32.30 (d, *J* = 56.6 Hz), 30.29 (d, *J* = 15.8 Hz), 20.03 (d, *J* = 2.5 Hz). HRMS (ESI-ion trap) m/z: [M+H]+ calcd for $C_{22}H_{25}NPS$ 366.1445; found 366.1440.

1-(4-(diphenylphosphorothioyl)butyl)-1-phenyl-3-(4-(trifluoromethyl) phenyl) thiourea (9)

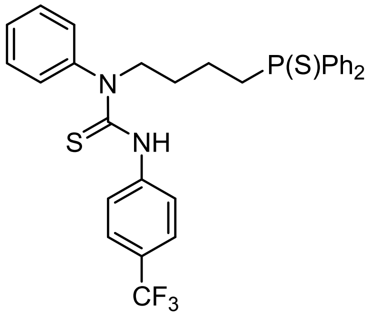

^1H NMR (400 MHz, Chloroform-*d*) δ 7.88-7.78 (m, 4H), 7.55-7.41 (m, 13H), 7.25-7.19 (m, 2H), 6.93 (s, 1H), 4.29-4.17 (m, 2H), 2.63-2.51 (m, 2H), 1.82-1.73 (m, 2H), 1.70-1.62 (m, 2H). ^{19}F NMR (376 MHz, Chloroform-*d*) δ -62.20. ^{31}P NMR (162 MHz, Chloroform-*d*) δ 42.79. ^{13}C NMR (101 MHz, Chloroform-*d*) δ 180.40, 142.25 (d, *J* = 1.5 Hz), 140.62, 132.72 (d, *J* = 79.9 Hz), 131.55 (d, *J* = 2.9 Hz), 131.19 (d, *J* = 10.1 Hz), 131.04, 129.46, 128.72 (d, *J* = 12.0 Hz), 127.84, 127.39 (d, *J* = 32.7 Hz), 125.71 (q, *J* = 3.7 Hz), 124.90, 124.06 (q, *J* = 271.8 Hz), 54.00, 32.17 (d, *J* = 56.5 Hz), 28.05 (d, *J* = 16.2 Hz), 19.26 (d, *J* = 2.4 Hz). HRMS (ESI-ion trap) m/z: [M+H]+ calcd for $C_{30}H_{29}F_3N_2PS_2$ 569.1462; found 569.1461.

diphenyl(4-(phenyl(pyridin-4-yl)amino)butyl)phosphine sulfide (10)

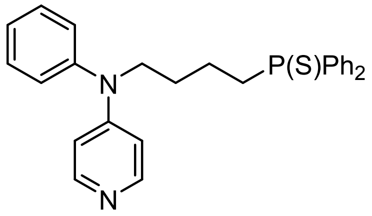

^{1}H NMR (600 MHz, Chloroform-*d*) δ 8.17-8.11 (m, 2H), 7.82-7.74 (m, 4H), 7.52-7.46 (m, 2H), 7.46-7.37 (m, 6H), 7.32-7.27 (m, 1H), 7.12-7.07 (m, 2H), 6.42-6.37 (m, 2H), 3.64-3.59 (m, 2H), 2.45-2.40 (m, 2H), 1.76-1.63 (m, 4H). ^{31}P NMR (243 MHz, Chloroform-*d*) δ 42.35. ^{13}C NMR (151 MHz, Chloroform-*d*) δ 153.46, 149.64, 144.59, 132.68 (d, *J* = 80.1 Hz), 131.67 (d, *J* = 3.1 Hz), 131.14 (d, *J* = 10.3 Hz), 130.30, 128.80 (d, *J* = 12.0 Hz), 127.90, 127.05, 108.27, 51.35, 32.49 (d, *J* = 56.3 Hz), 28.14 (d, *J* = 15.5 Hz), 19.88 (d, *J* = 2.2 Hz). HRMS (ESI-ion trap) m/z: [M+H]+ calcd for $C_{27}H_{28}N_2PS$ 443.1711; found 443.1707.

((*R*)-3-(diphenylphosphorothioyl)butanoyl)-L-alanine (11)

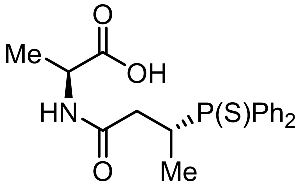

Colorless oil (36.1 mg, 96% yield).^{1}H NMR (600 MHz, Chloroform-*d*) δ 8.23 (s, 1H), 8.01 (dd, *J* = 12.4, 7.0 Hz, 2H), 7.93 (dd, *J* = 12.6, 7.5 Hz, 2H), 7.49-7.42 (m, 6H), 6.59 (d, *J* = 6.6 Hz, 1H), 4.38-4.25 (m, 1H), 3.60-3.51 (m, 1H), 2.54-2.44 (m, 2H), 1.32 (d, *J* = 7.3 Hz, 3H), 1.10 (dd, *J* = 18.6, 6.7 Hz, 3H). ^{31}P NMR (243 MHz, Chloroform-*d*) δ 52.69. ^{13}C NMR (151 MHz, Chloroform-*d*) δ 176.01, 171.84 (d, *J* = 15.8 Hz), 131.84 (dd, *J* = 15.7, 3.0 Hz), 131.53 (dd, *J* = 13.5, 9.6 Hz), 130.91 (dd, *J* = 78.1, 24.2 Hz), 128.88 (dd, *J* = 19.1, 11.9 Hz), 48.56, 37.05, 29.98 (d, *J* = 57.4 Hz), 17.60, 13.34. HRMS (ESI-ion trap) m/z: [M+H]+ calcd for $C_{19}H_{23}NO_3PS$ 376.1136; found 376.1133.

((*R*)-3-(diphenylphosphorothioyl)butanoyl)-D-tryptophan (12)

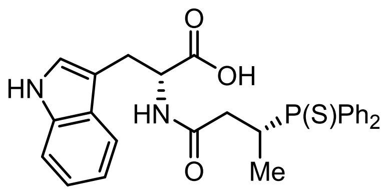

Colorless oil (46.5 mg, 95% yield). ^{1}H NMR (600 MHz, Chloroform-*d*) δ 8.57 (s, 1H), 8.35 (d, *J* = 2.5 Hz, 1H), 7.99-7.81 (m, 4H), 7.47-7.35 (m, 7H), 7.29 (d, *J* = 8.2 Hz, 1H), 7.12 (t, *J* = 7.6 Hz, 1H), 6.96 (t, *J* = 7.5 Hz, 1H), 6.89 (d, *J* = 2.4 Hz, 1H), 6.36 (d, *J* = 7.3 Hz, 1H), 4.77-4.70 (m, 1H), 3.47-3.40 (m, 1H), 3.29-3.12 (m, 2H), 2.39-2.30 (m, 1H), 2.30-2.21 (m, 1H), 1.02 (dd, *J* = 18.7, 6.7 Hz, 3H). ^{31}P NMR (243 MHz, Chloroform-*d*) δ 52.60. ^{13}C NMR (151 MHz, Chloroform-*d*) δ 175.28, 171.78 (d, *J* = 17.2 Hz), 136.17, 131.81 (d, *J* = 18.8 Hz), 131.47 (t, *J* = 8.2 Hz), 130.79 (dd, *J* = 78.1, 17.0 Hz), 128.85 (dd, *J* = 26.9, 11.8 Hz), 127.55, 123.36 (d, *J* = 2.6 Hz), 122.35, 119.87, 118.44, 111.58, 109.26, 53.41, 36.91, 30.03 (d, *J* = 57.6 Hz), 27.07, 13.09. HRMS (ESI-ion trap) m/z: [M+H]+ calcd for $C_{27}H_{28}N_2O_3PS$ 491.1558; found 491.1556.

(4-(diphenylphosphorothioyl)butanoyl)-L-alanine (13)

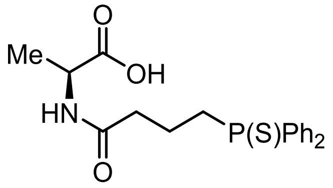

Colorless oil (36.5 mg, 98% yield).^{1}H NMR (600 MHz, Chloroform-*d*) δ 8.68 (s, 1H), 7.88-7.73 (m, 4H), 7.52-7.35 (m, 6H), 6.72 (d, *J* = 7.0 Hz, 1H), 4.61-4.37 (m, 1H), 2.64-2.51 (m, 2H), 2.47-2.35 (m, 2H), 2.00-1.91 (m, 2H), 1.40 (d, *J* = 7.2 Hz, 3H). ^{31}P NMR (243 MHz, Chloroform-*d*) δ 42.52. ^{13}C NMR (151 MHz, Chloroform-*d*) δ 176.03, 173.33, 132.35 (dd, *J* = 80.5, 63.3 Hz), 131.76 (dd, *J* = 6.2, 2.9 Hz), 131.21 (dd, *J* = 18.0, 10.3 Hz), 128.87 (d, *J* = 12.0 Hz), 48.53, 35.85 (d, *J* = 14.2 Hz), 31.02 (d, *J* = 56.6 Hz), 18.88, 17.74. HRMS (ESI-ion trap) m/z: [M+H]+ calcd for $C_{19}H_{23}NO_3PS$ 376.1136; found 376.1132.

.

(*S*)-2-(4-(diphenylphosphorothioyl)butanamido)-2-phenylacetic acid (14)

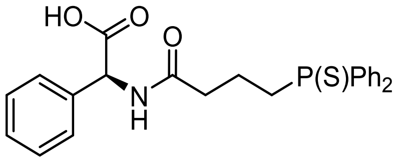

Colorless oil (42.4 mg, 97% yield). ^{1}H NMR (600 MHz, Chloroform-*d*) δ 8.35 (s, 1H), 7.79-7.72 (m, 4H), 7.46-7.41 (m, 2H), 7.41-7.34 (m, 6H), 7.32-7.27 (m, 3H), 6.97 (d, *J* = 7.0 Hz, 1H), 5.52 (d, *J* = 7.0 Hz, 1H), 2.56-2.34 (m, 4H), 1.98-1.87 (m, 2H). ^{31}P NMR (243 MHz, Chloroform-*d*) δ 42.39. ^{13}C NMR (151 MHz, Chloroform-*d*) δ 173.95, 172.70, 135.74, 132.36 (dd, *J* = 80.8, 42.5 Hz), 131.70, 131.19 (t, *J* = 11.0 Hz), 129.20, 128.92, 128.81 (dd, *J* = 12.0, 3.2 Hz), 127.64, 56.80, 35.77 (d, *J* = 13.8 Hz), 30.86 (d, *J* = 56.4 Hz), 18.89. HRMS (ESI-ion trap) m/z: [M+H]+ calcd for $C_{24}H_{25}NO_3PS$ 438.1293; found 438.1293.

10. NMR Spectrum of target compounds

(R)-3-(diphenylphosphorothioyl)-N-phenylbutanamide (3a)

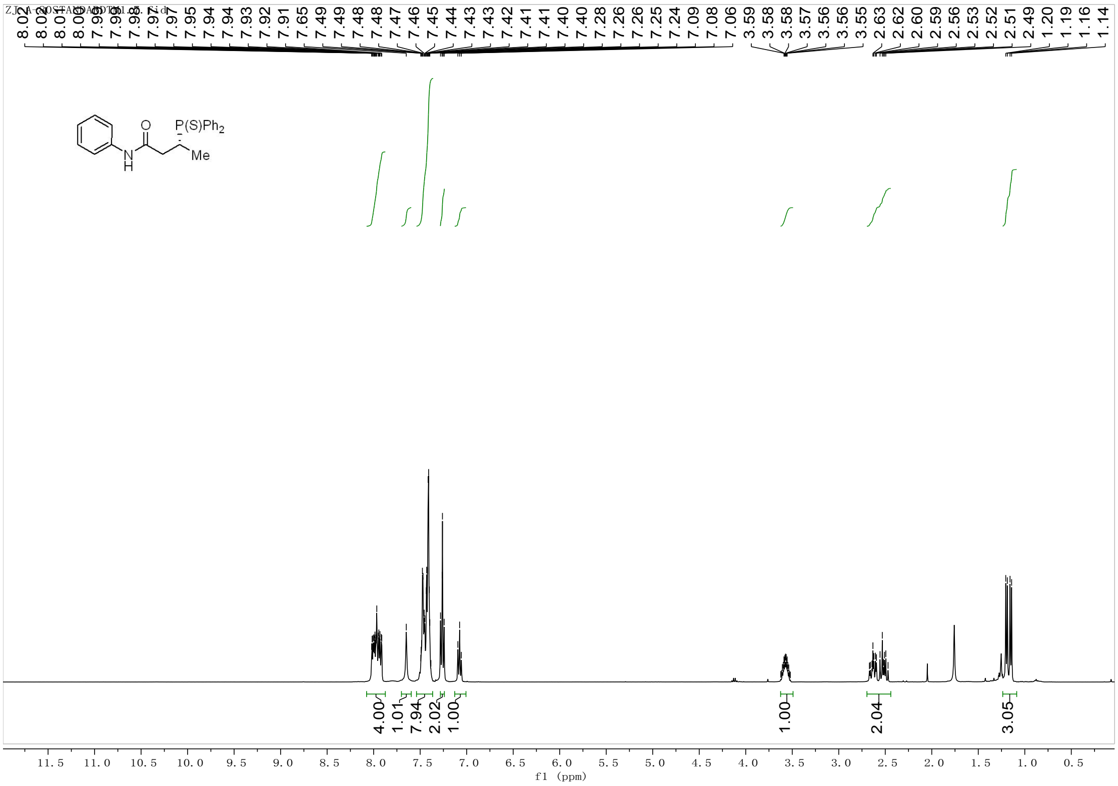

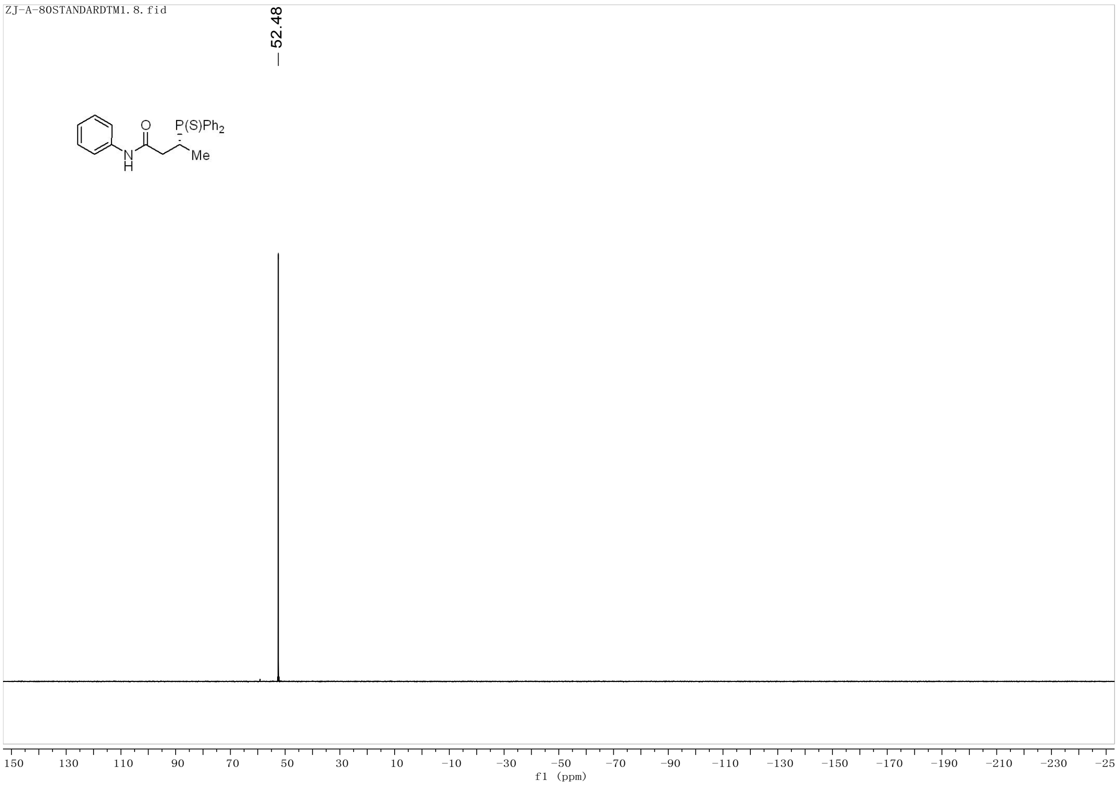

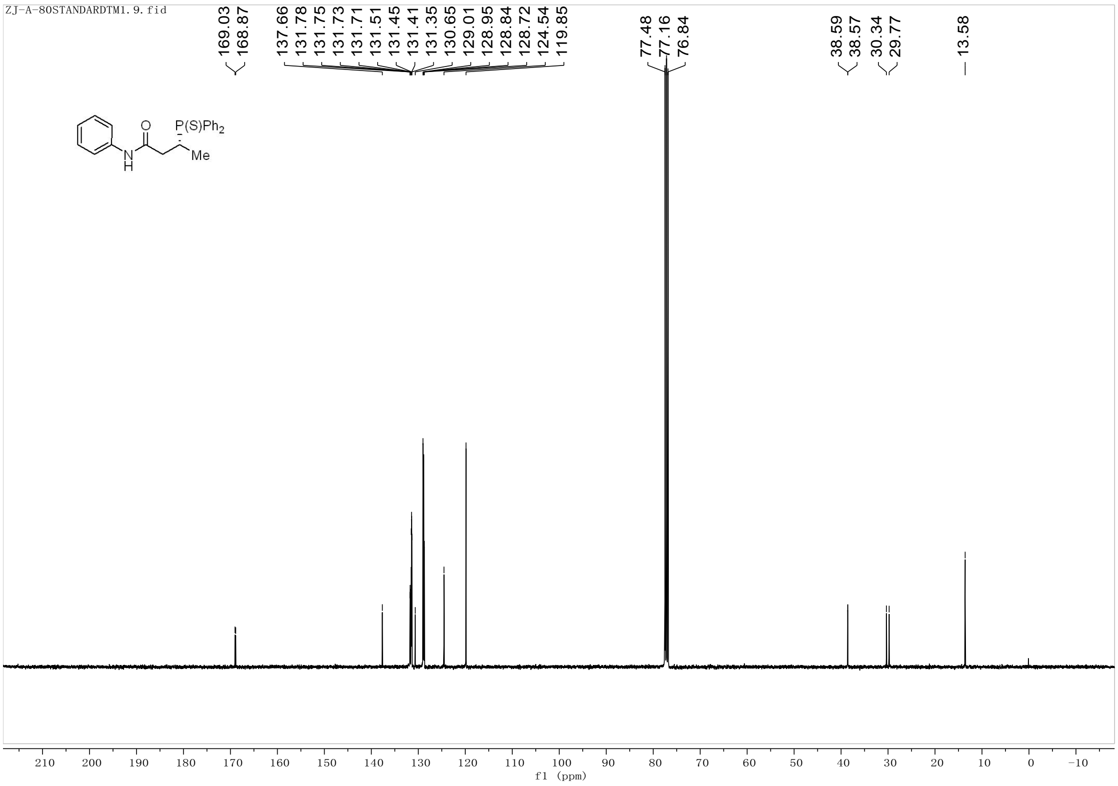

(*R*)-3-(diphenylphosphorothioyl)-N-(o-tolyl)butanamide (3b)

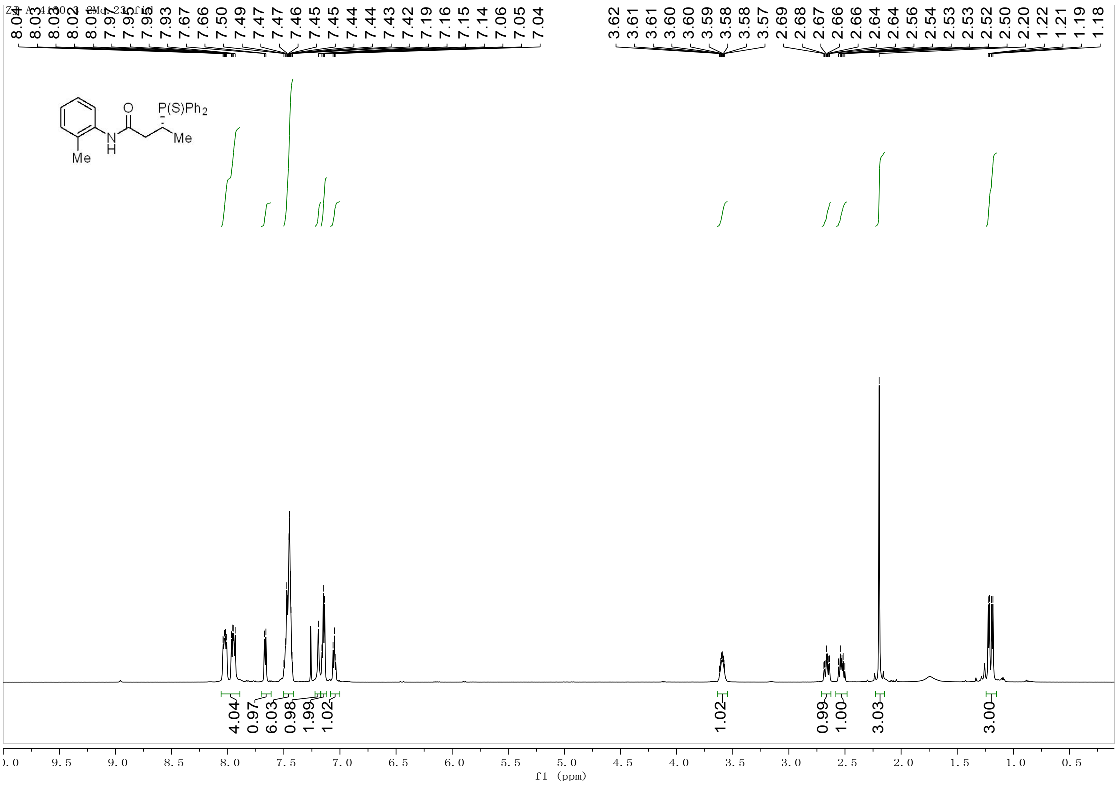

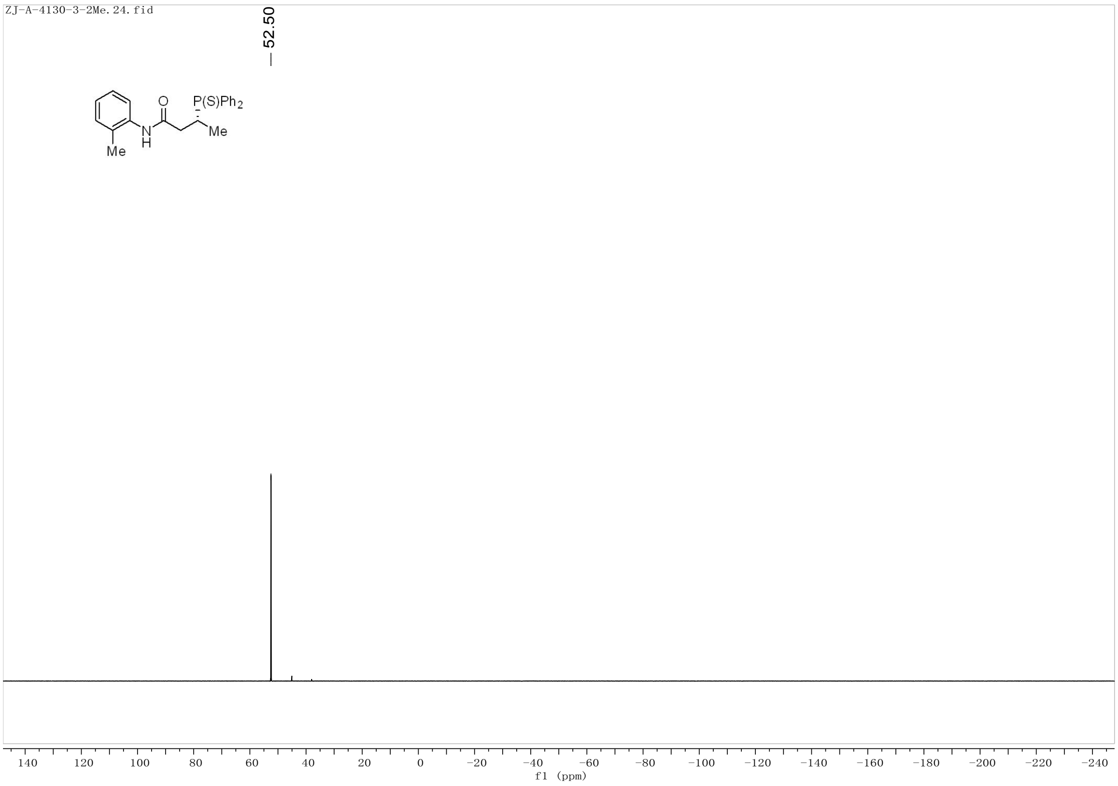
ZJ-A-4130-3-2Me.24.fid
52.50
P(S)Ph2
O
N
H
Me
Me
f1 (ppm)

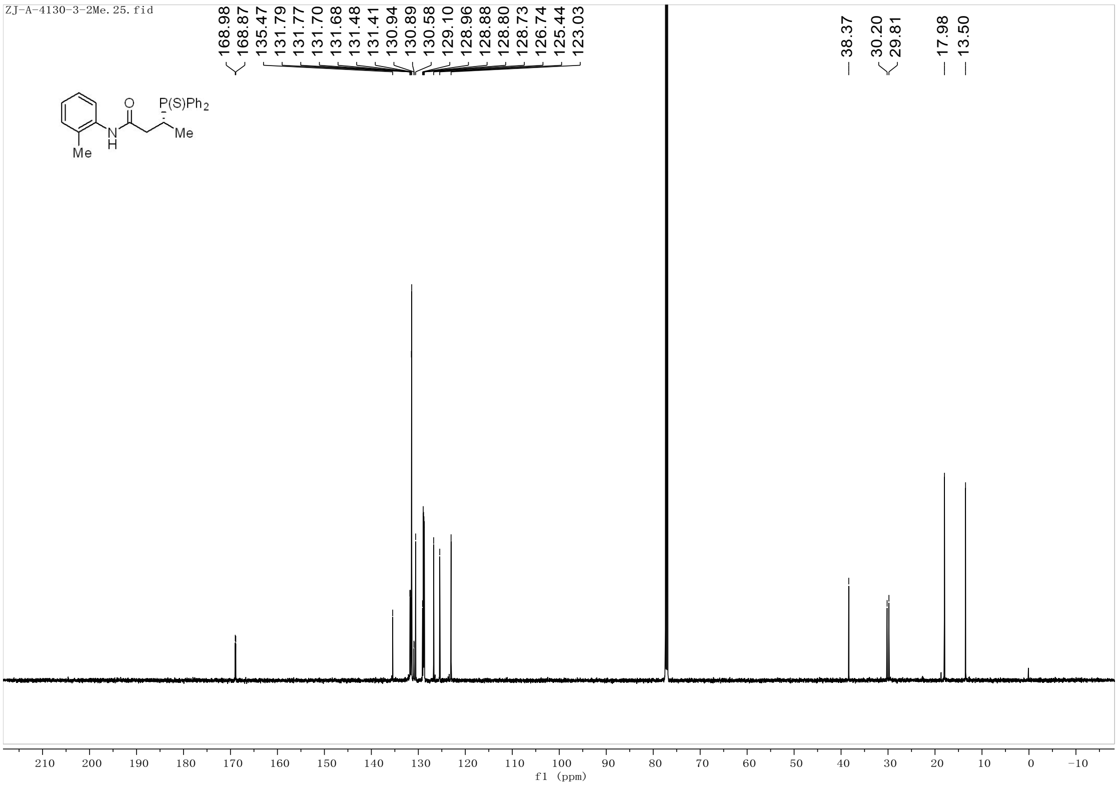
ZJ-A-4130-3-2Me.25.fid
168.98
168.87
135.47
131.79
131.77
131.70
131.68
131.48
131.41
130.94
130.89
130.58
129.10
128.96
128.88
128.80
128.73
126.74
125.44
123.03
38.37
30.20
29.81
17.98
13.50
P(S)Ph2
O
N
H
Me
Me
f1 (ppm)

(*R*)-3-(diphenylphosphorothioyl)-N-(m-tolyl)butanamide (3c)

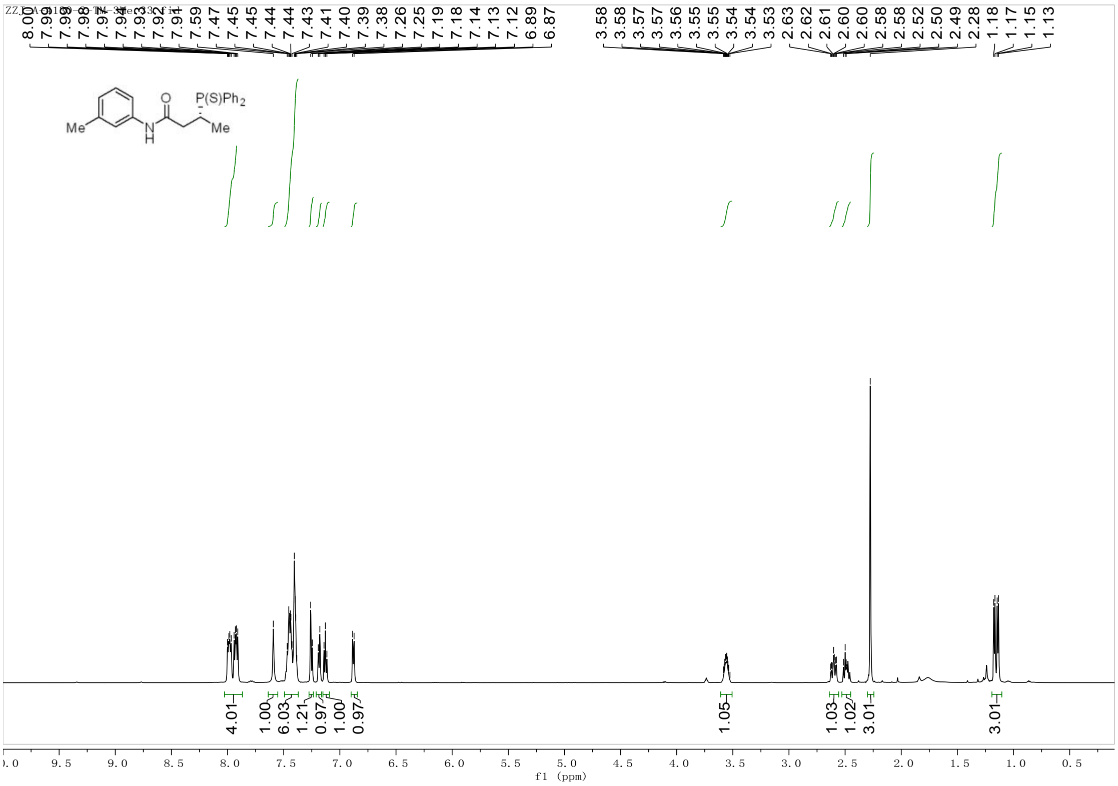

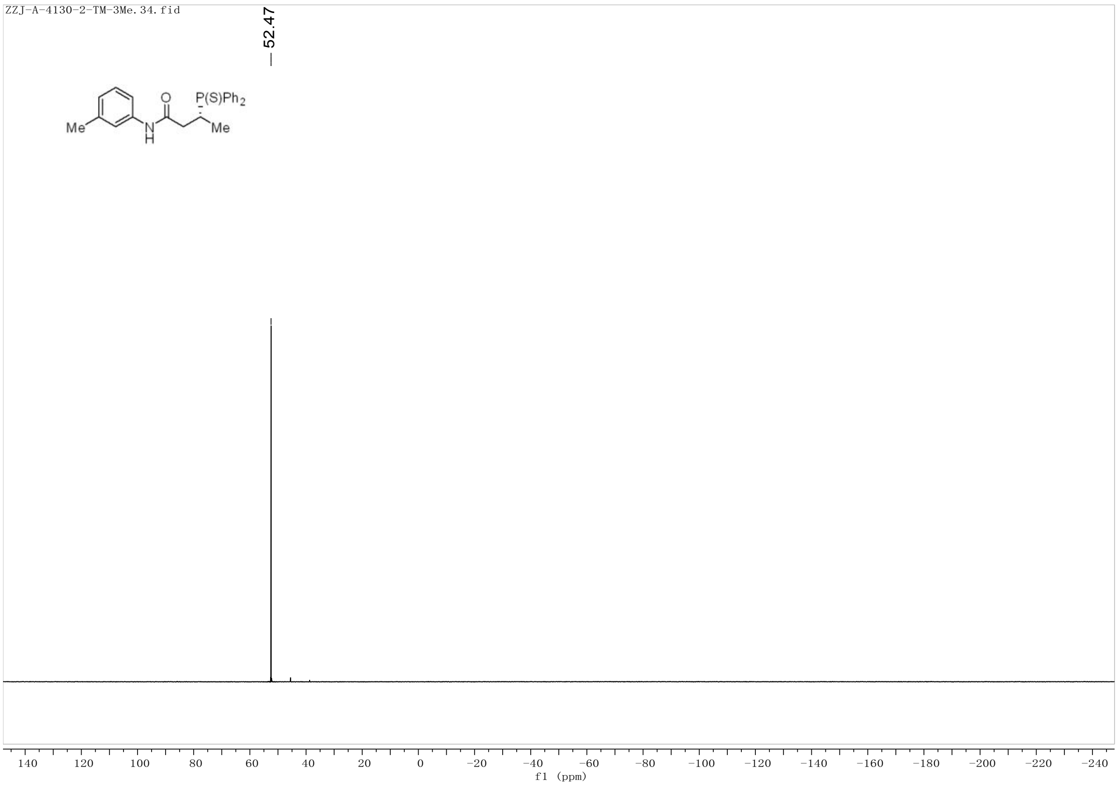

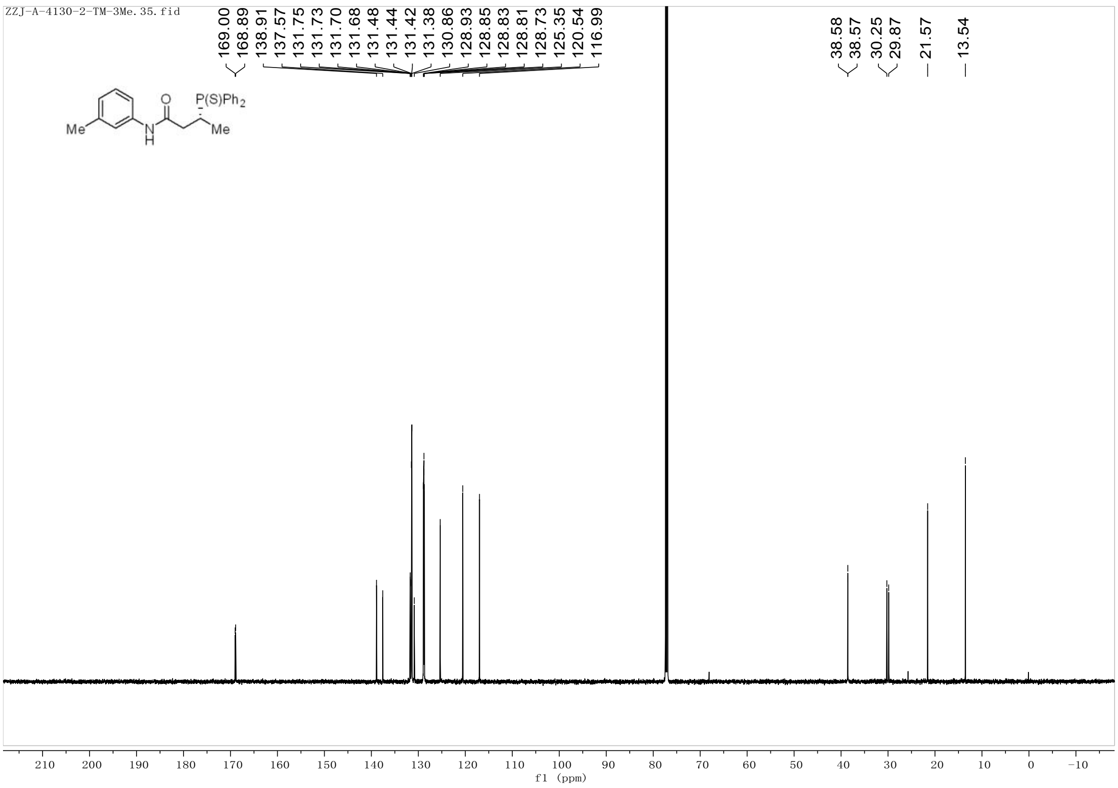

(*R*)-3-(diphenylphosphorothioyl)-N-(p-tolyl)butanamide (3d)

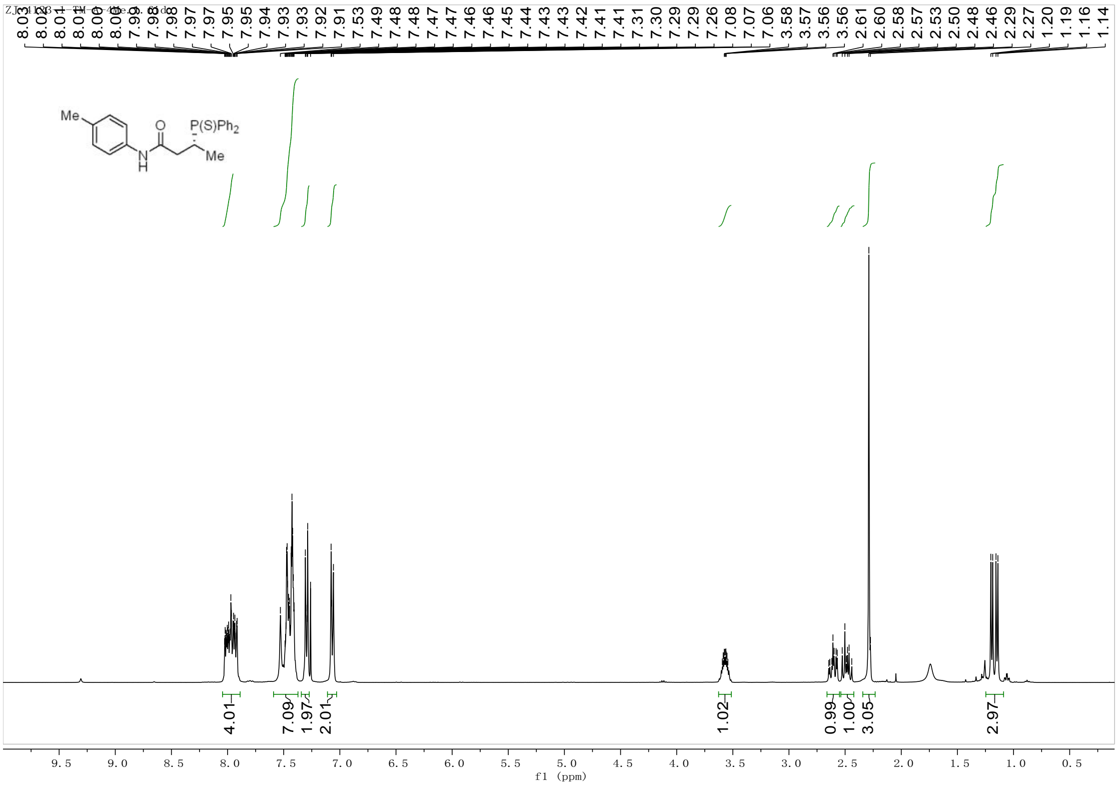

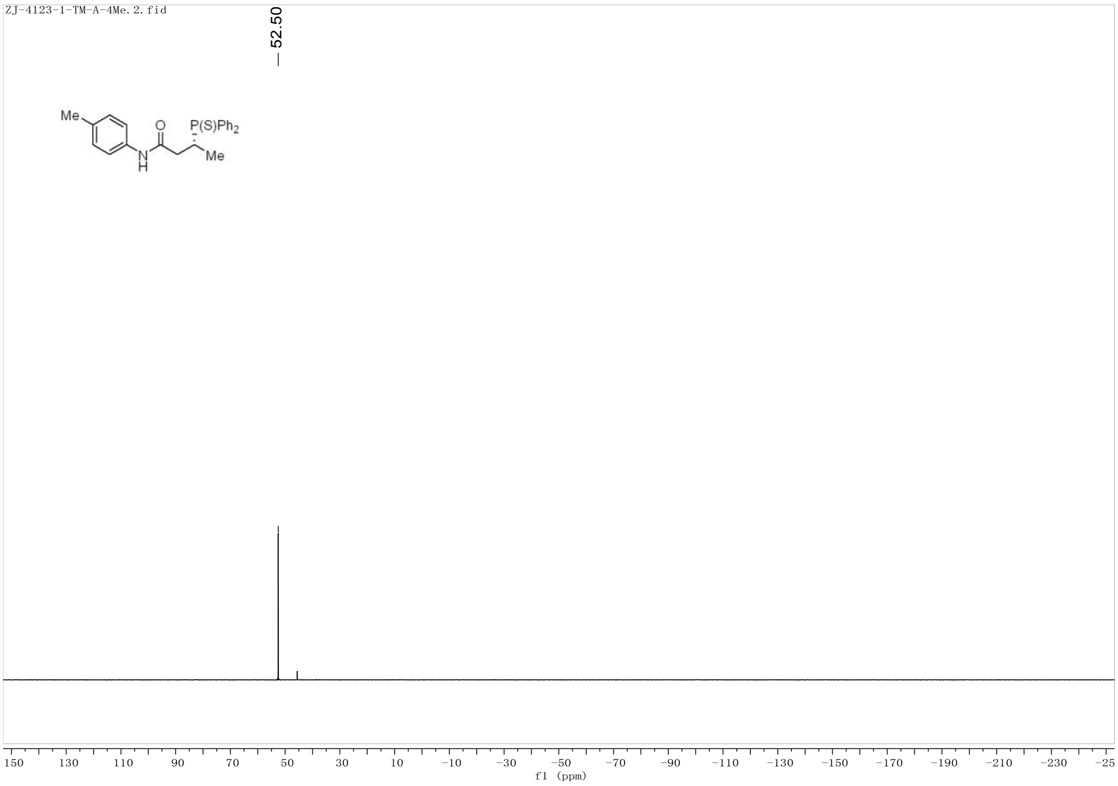
ZJ-4123-1-TM-A-4Me.2.fid
52.50
Me
O
P(S)Ph2
N
H
Me
150 130 110 90 70 50 30 10 −10 −30 −50 −70 −90 −110 −130 −150 −170 −190 −210 −230 −25
f1 (ppm)

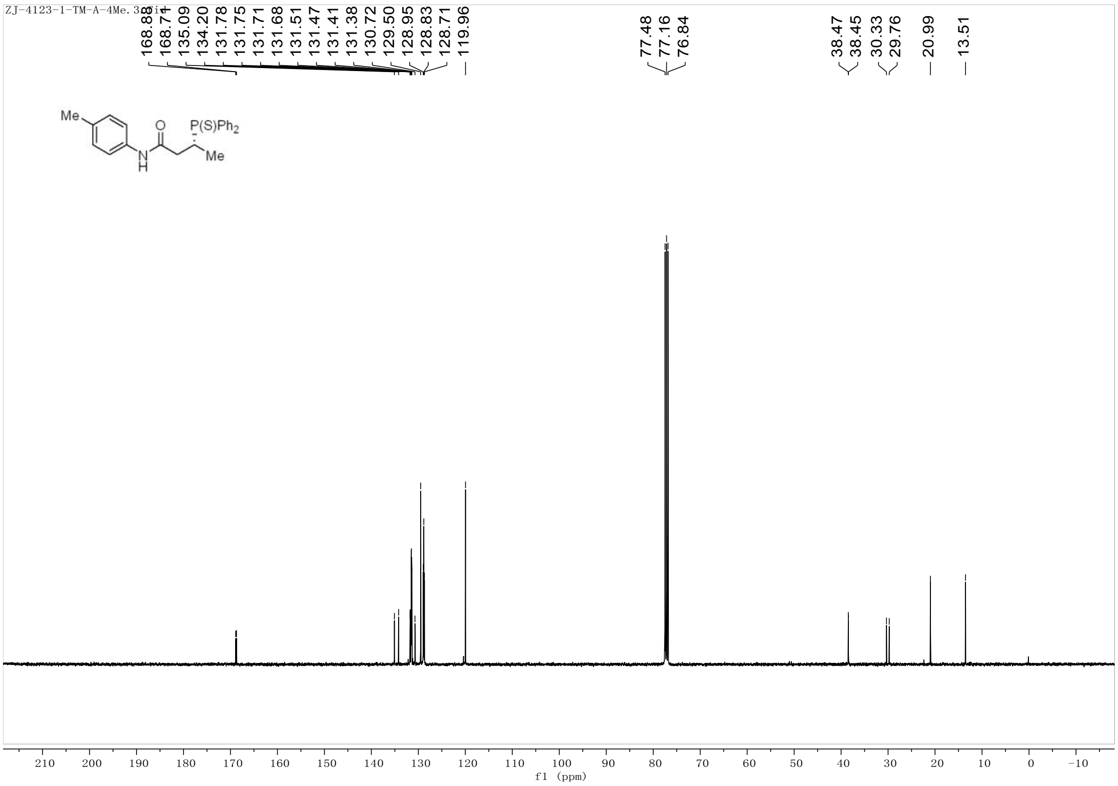
ZJ-4123-1-TM-A-4Me.3
168.83
168.71
135.09
134.20
131.78
131.75
131.71
131.68
131.51
131.47
131.41
131.38
130.72
129.50
128.95
128.83
128.71
119.96
77.48
77.16
76.84
38.47
38.45
30.33
29.76
20.99
13.51
Me
O
P(S)Ph2
N
H
Me
210 200 190 180 170 160 150 140 130 120 110 100 90 80 70 60 50 40 30 20 10 0 −10
f1 (ppm)

(*R*)-N-(4-(tert-butyl)phenyl)-3-(diphenylphosphorothioyl)butanamide (3e)

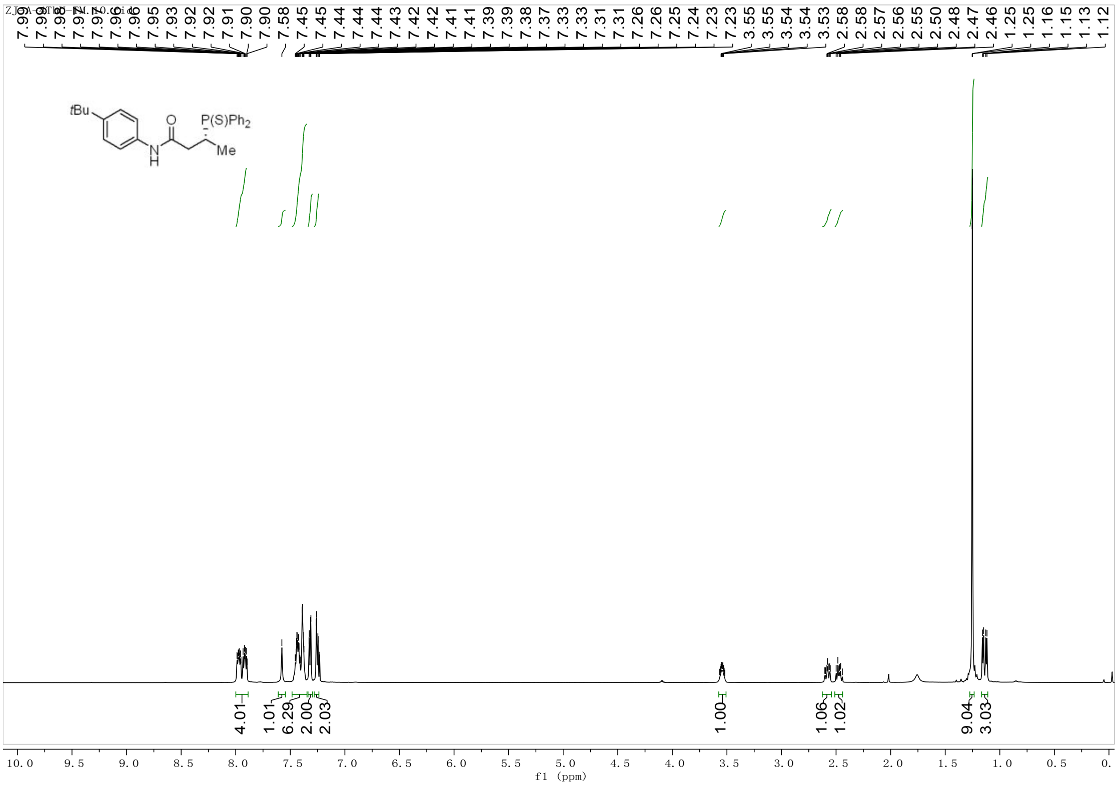

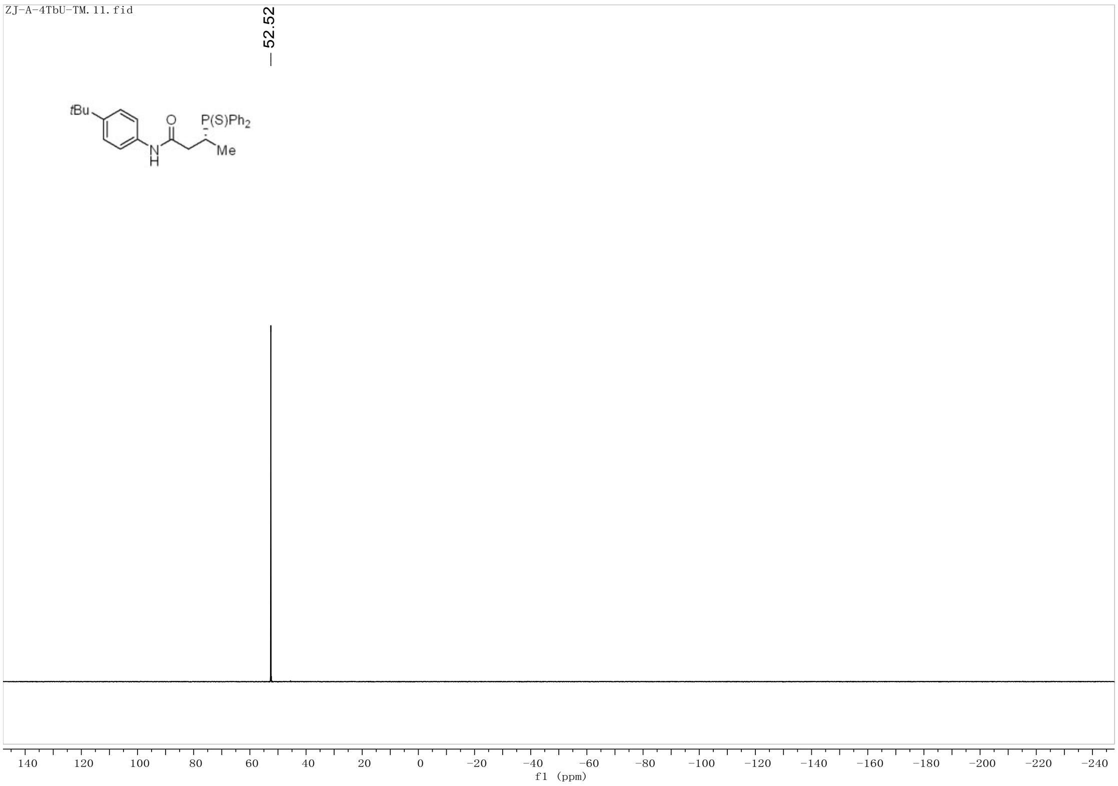

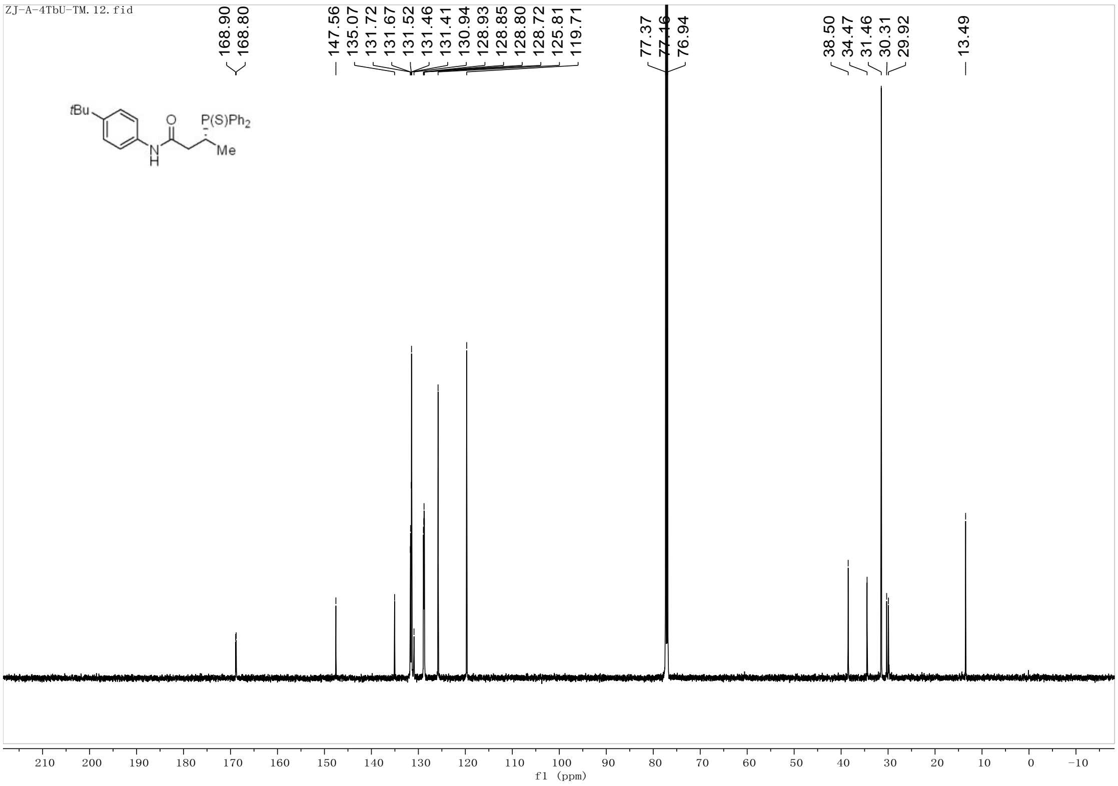

(*R*)-N-(4-benzylphenyl)-3-(diphenylphosphorothioyl)butanamide (3f)

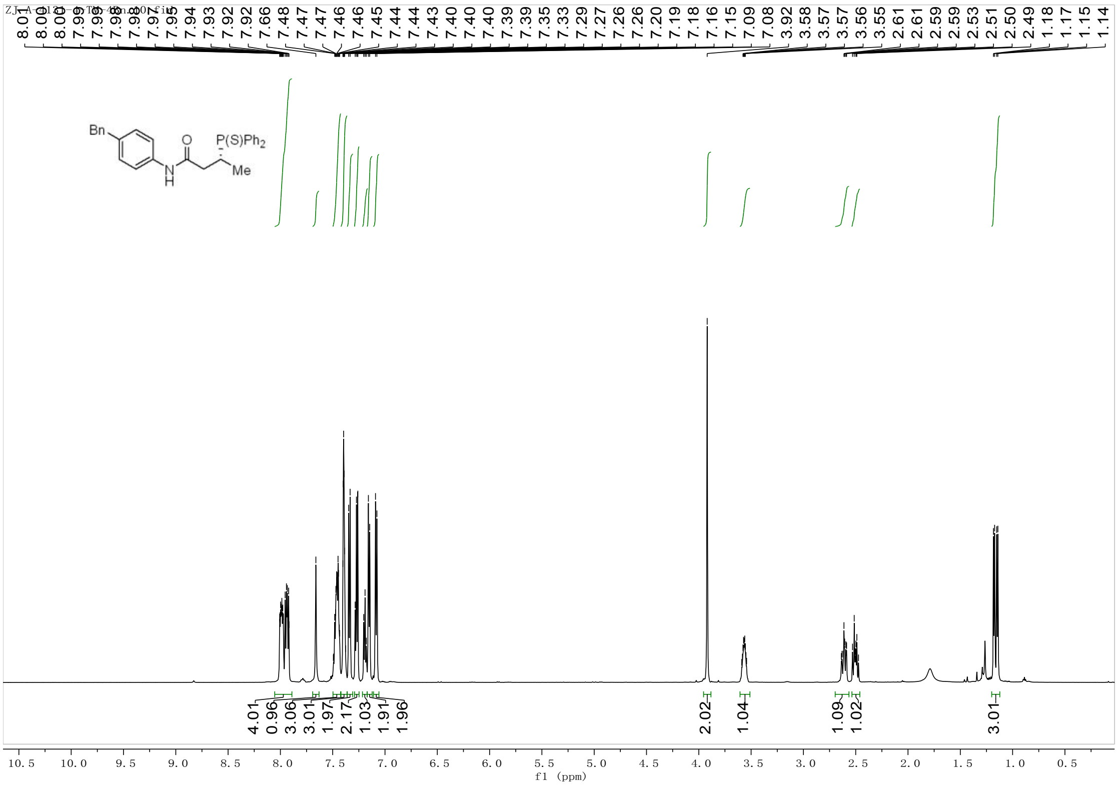

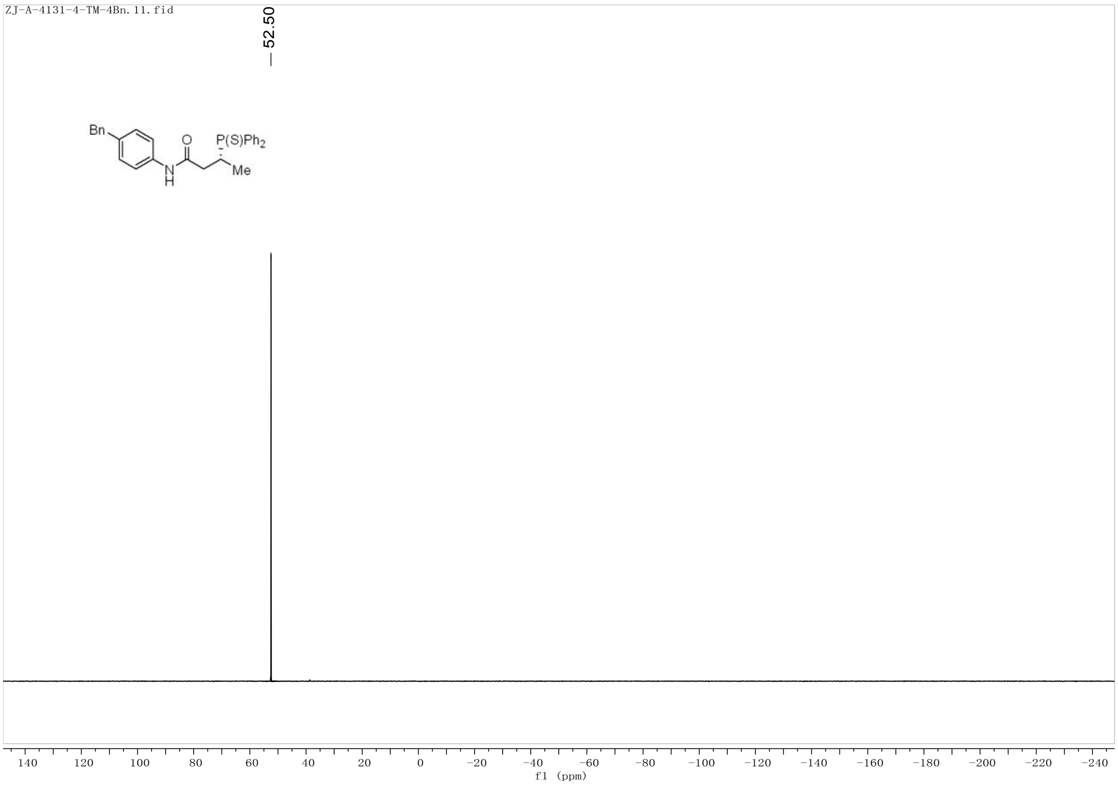
ZJ-A-4131-4-TM-4Bn.11.fid
52.50
Bn
O
P(S)Ph2
N
H
Me
140 120 100 80 60 40 20 0 −20 −40 −60 −80 −100 −120 −140 −160 −180 −200 −220 −240
f1 (ppm)

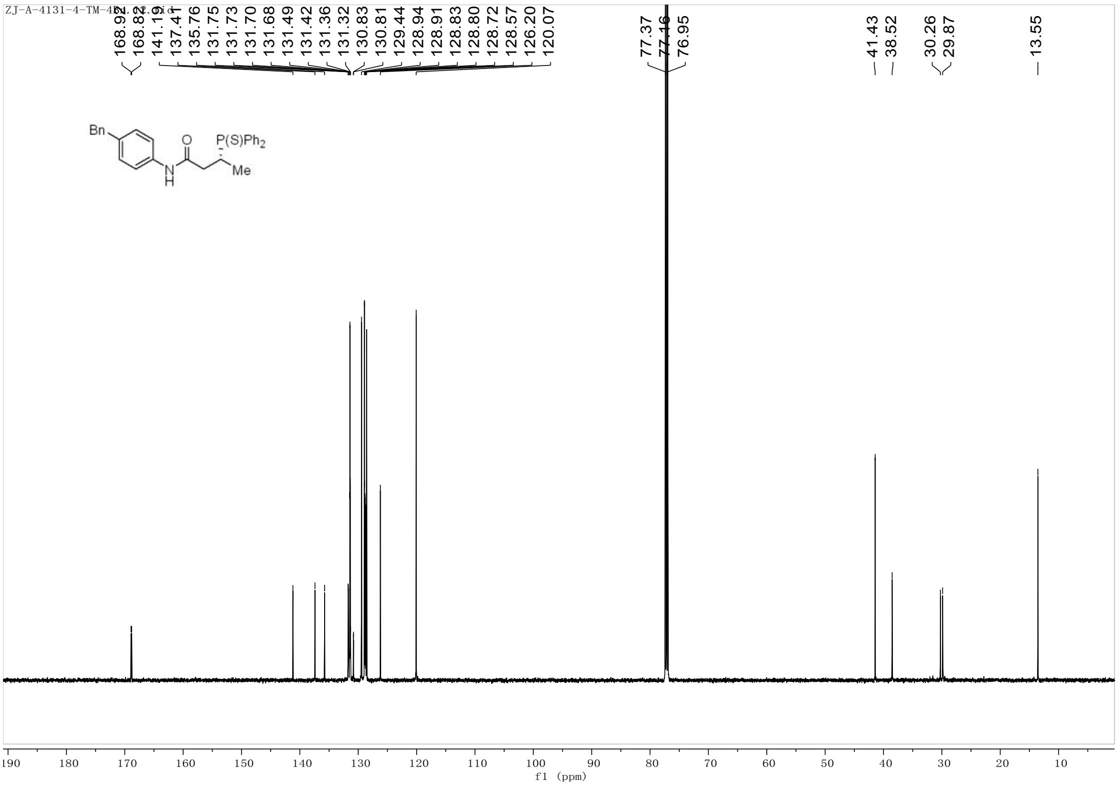
168.92
168.82
141.19
137.41
135.76
131.75
131.73
131.70
131.68
131.49
131.42
131.36
131.32
130.83
130.81
129.44
128.94
128.91
128.83
128.80
128.72
128.57
126.20
120.07
77.37
77.16
76.95
41.43
38.52
30.26
29.87
13.55
Bn
O
P(S)Ph2
N
H
Me
190 180 170 160 150 140 130 120 110 100 90 80 70 60 50 40 30 20 10
f1 (ppm)

(*R*)-3-(diphenylphosphorothioyl)-N-(4-(trifluoromethyl)phenyl)butanamide (3g)

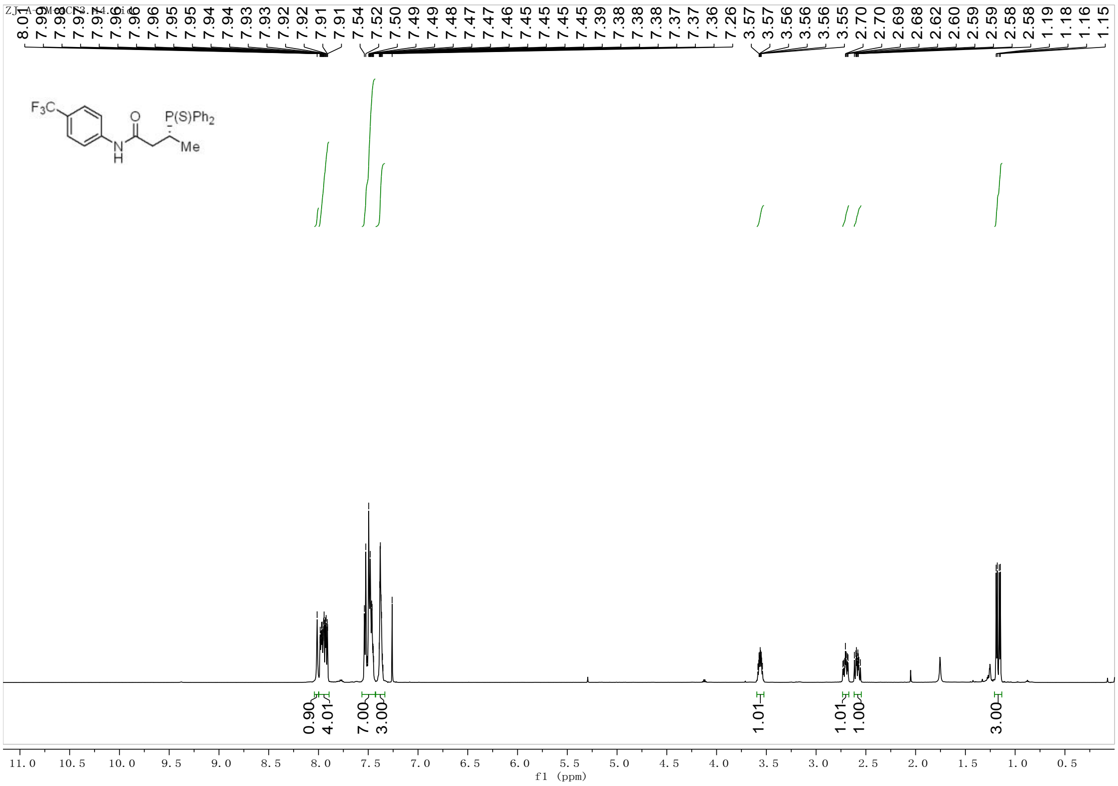

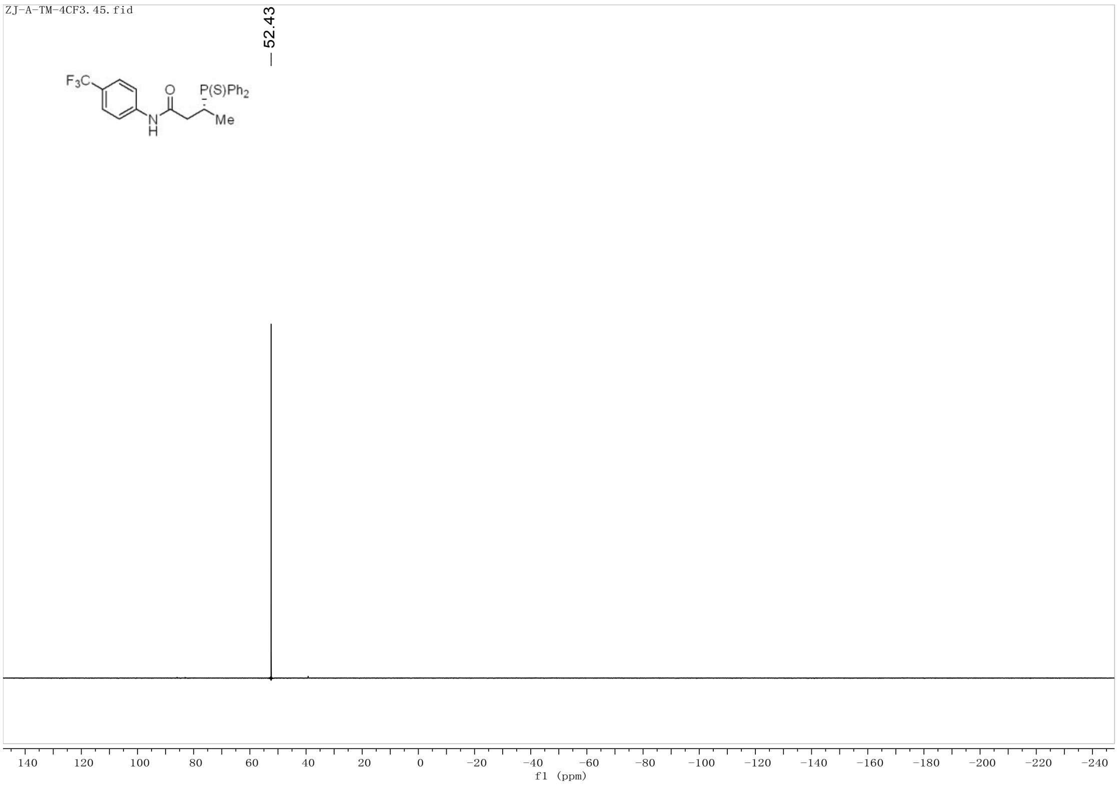

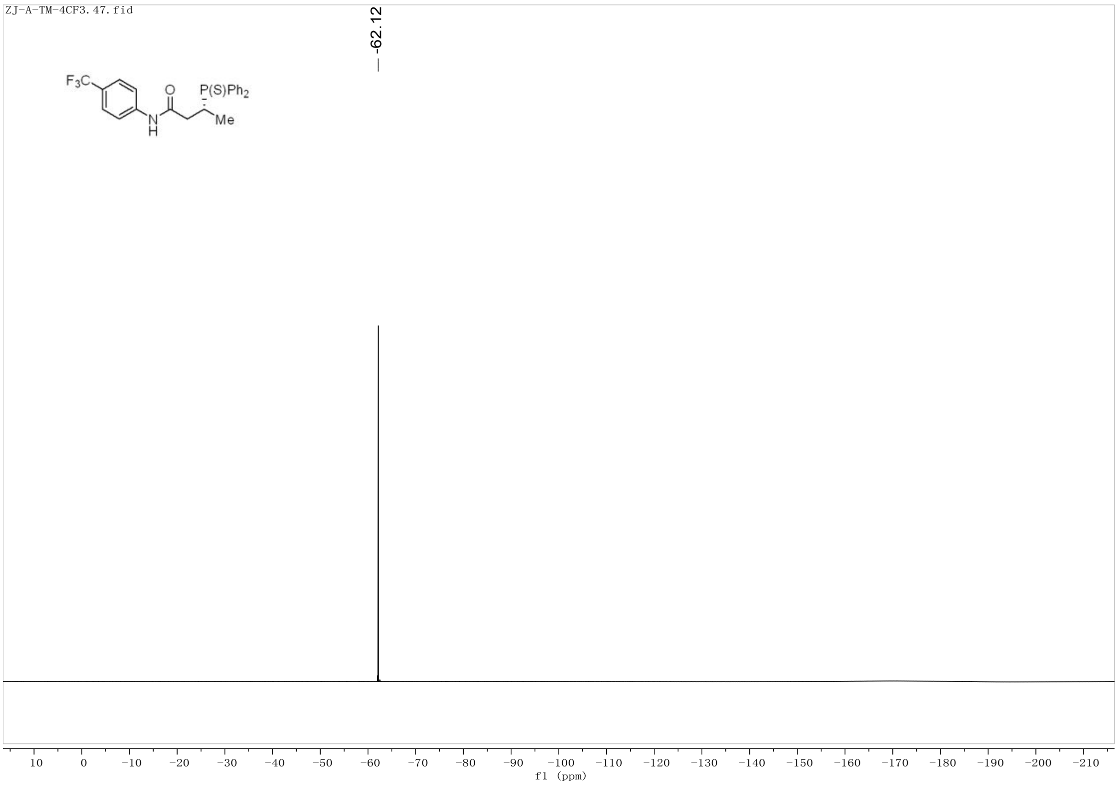
ZJ-A-TM-4CF3.47.fid
-62.12
F3C
O
P(S)Ph2
N
H
Me
10 0 −10 −20 −30 −40 −50 −60 −70 −80 −90 −100 −110 −120 −130 −140 −150 −160 −170 −180 −190 −200 −210
f1 (ppm)

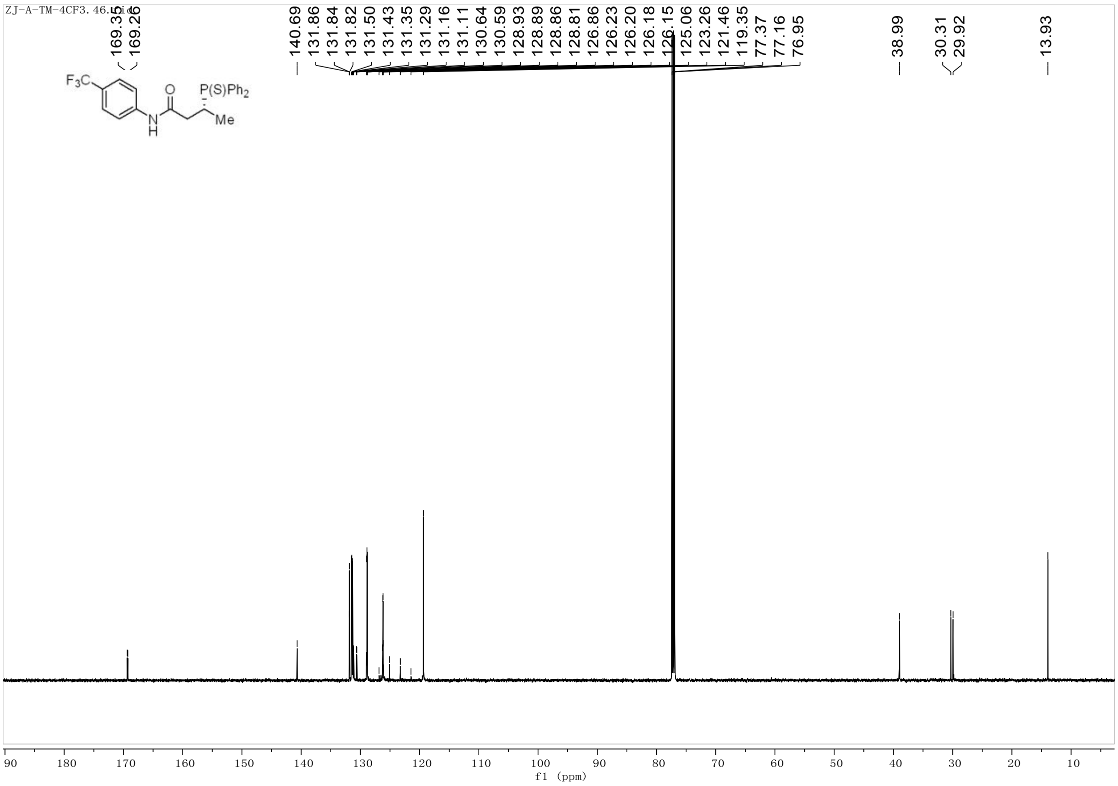
ZJ-A-TM-4CF3.46.fid
169.35
169.26
140.69
131.86
131.84
131.82
131.50
131.43
131.35
131.29
131.16
131.11
130.64
130.59
128.93
128.89
128.86
128.81
126.86
126.23
126.20
126.18
126.15
125.06
123.26
121.46
119.35
77.37
77.16
76.95
38.99
30.31
29.92
13.93
F3C
O
P(S)Ph2
N
H
Me
90 180 170 160 150 140 130 120 110 100 90 80 70 60 50 40 30 20 10
f1 (ppm)

(*R*)-3-(diphenylphosphorothioyl)-N-(4-fluorophenyl)butanamide (3h)

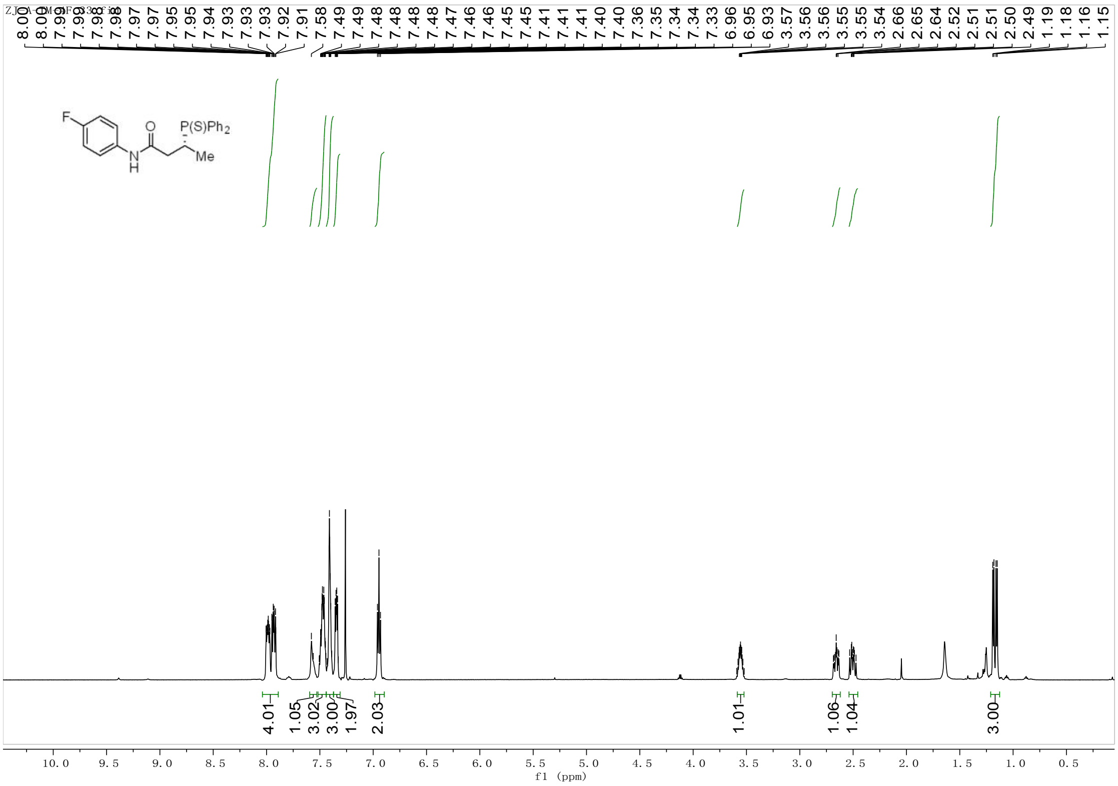

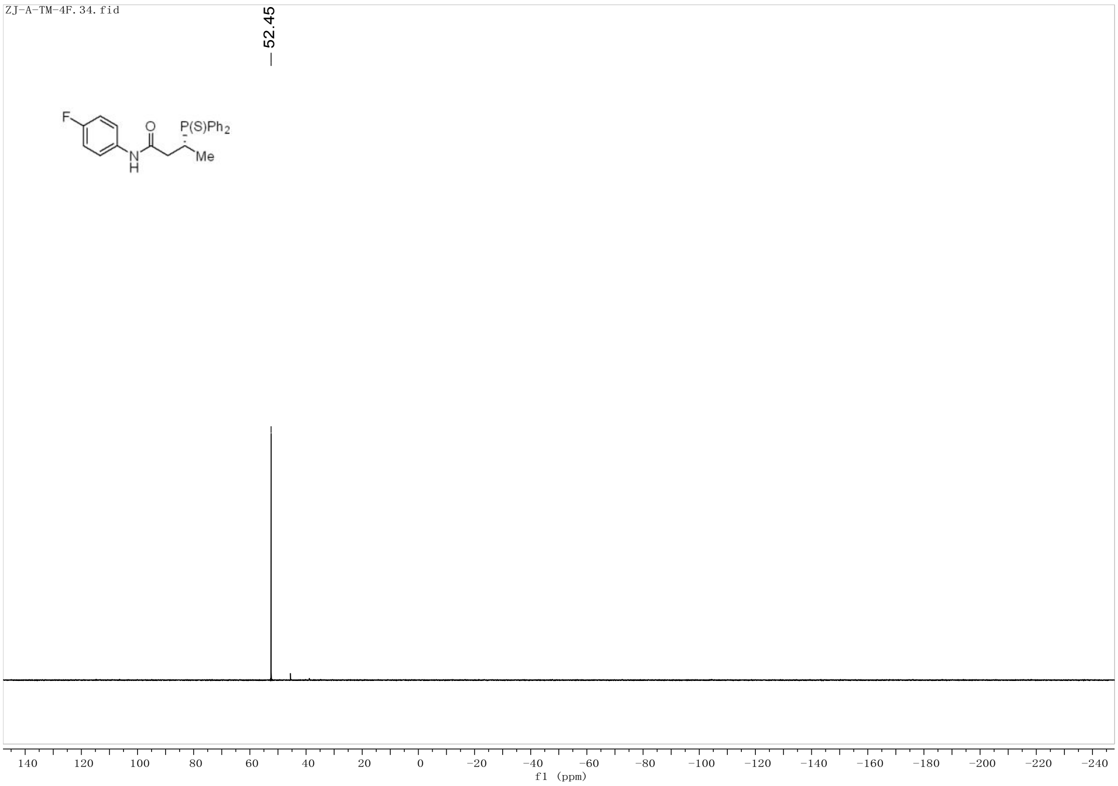

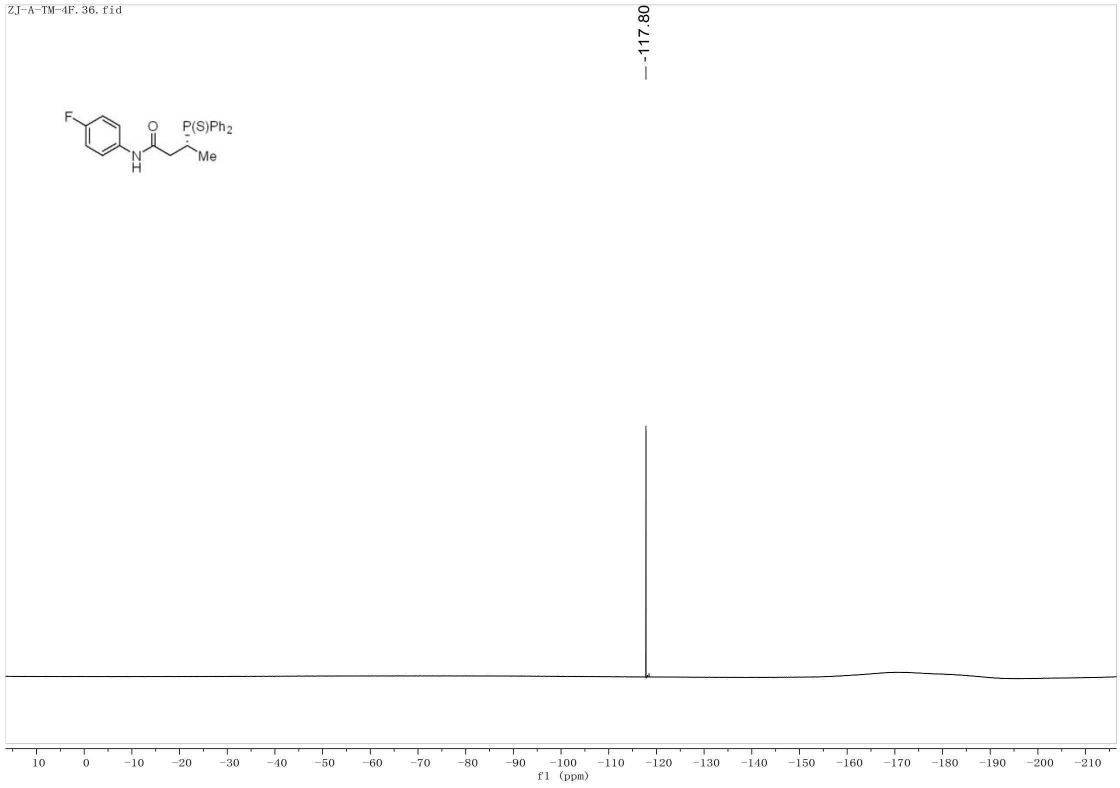
ZJ-A-TM-4F.36.fid
-117.80
F
O
P(S)Ph2
N
H
Me
f1 (ppm)

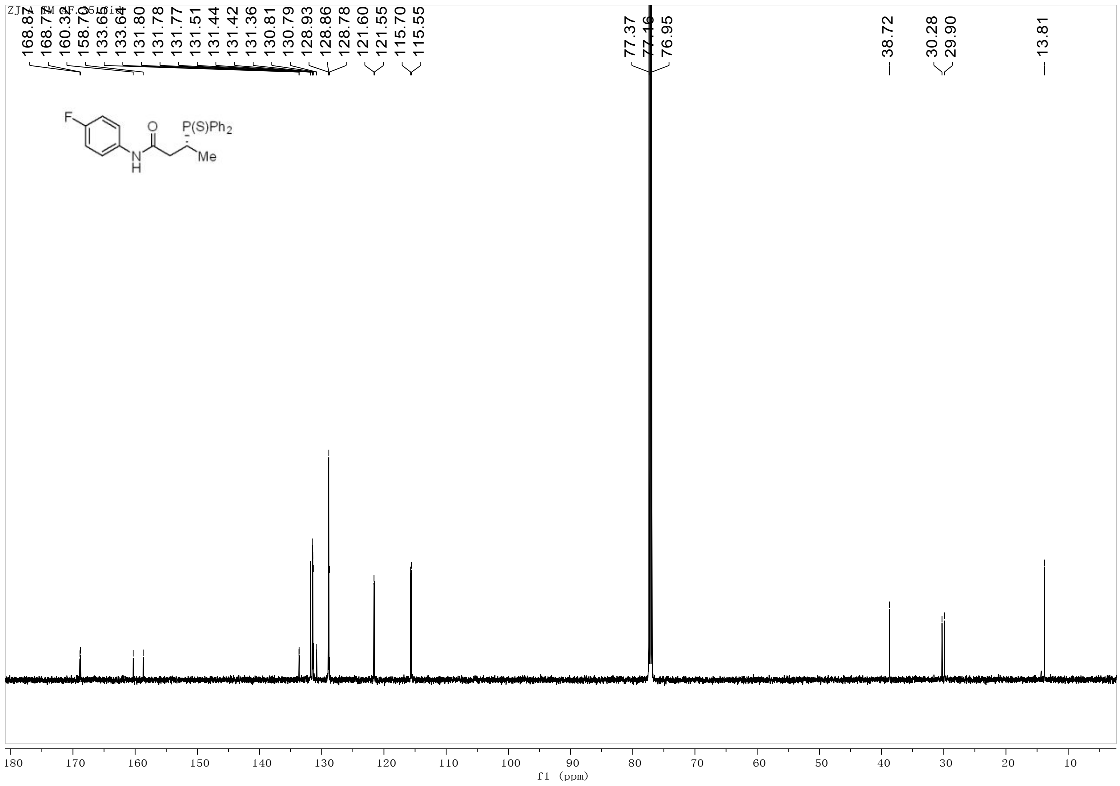
168.87
168.77
160.32
158.70
133.65
133.64
131.80
131.78
131.77
131.51
131.44
131.42
131.36
130.81
130.79
128.93
128.86
128.78
121.60
121.55
115.70
115.55
77.37
77.16
76.95
38.72
30.28
29.90
13.81
F
O
P(S)Ph2
N
H
Me
f1 (ppm)

(*R*)-N-(4-chlorophenyl)-3-(diphenylphosphorothioyl)butanamide (3i)

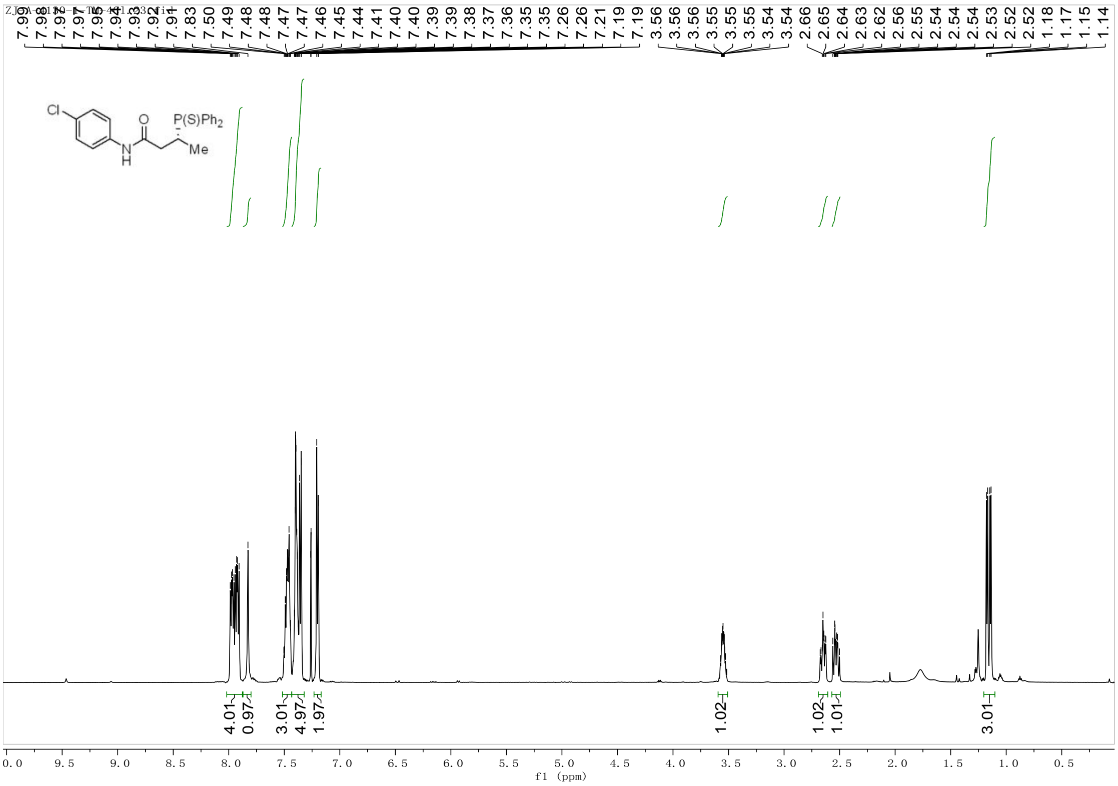

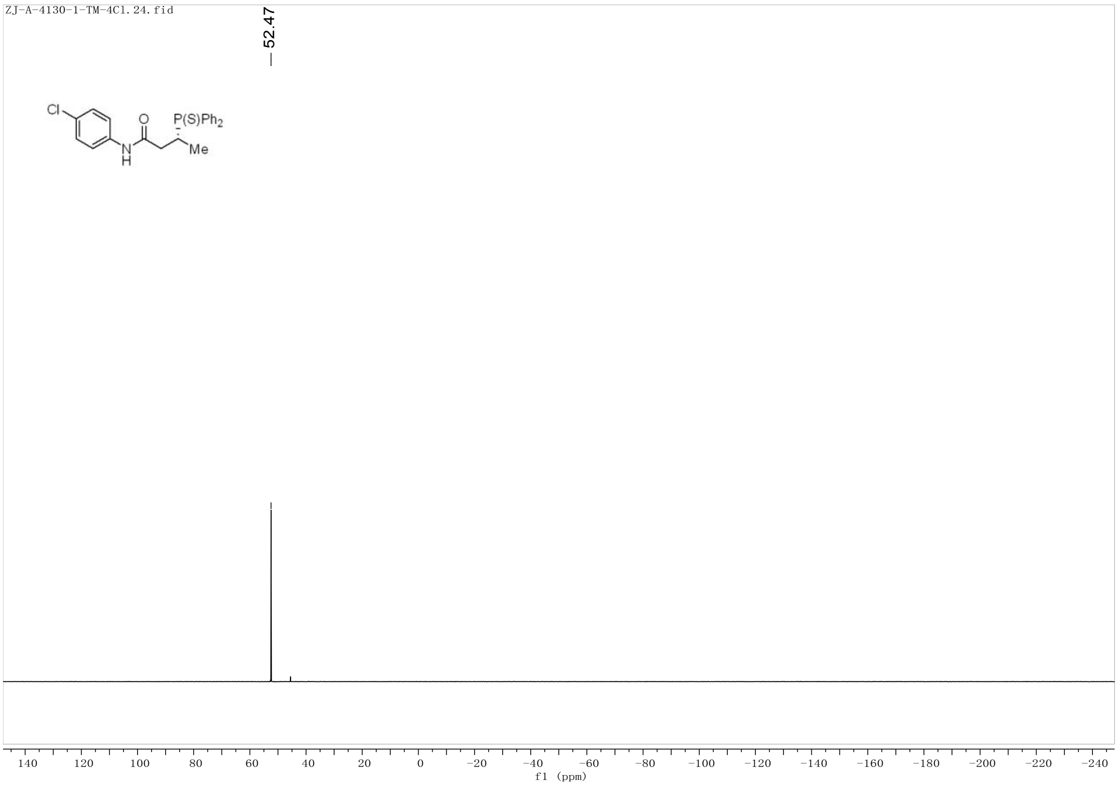

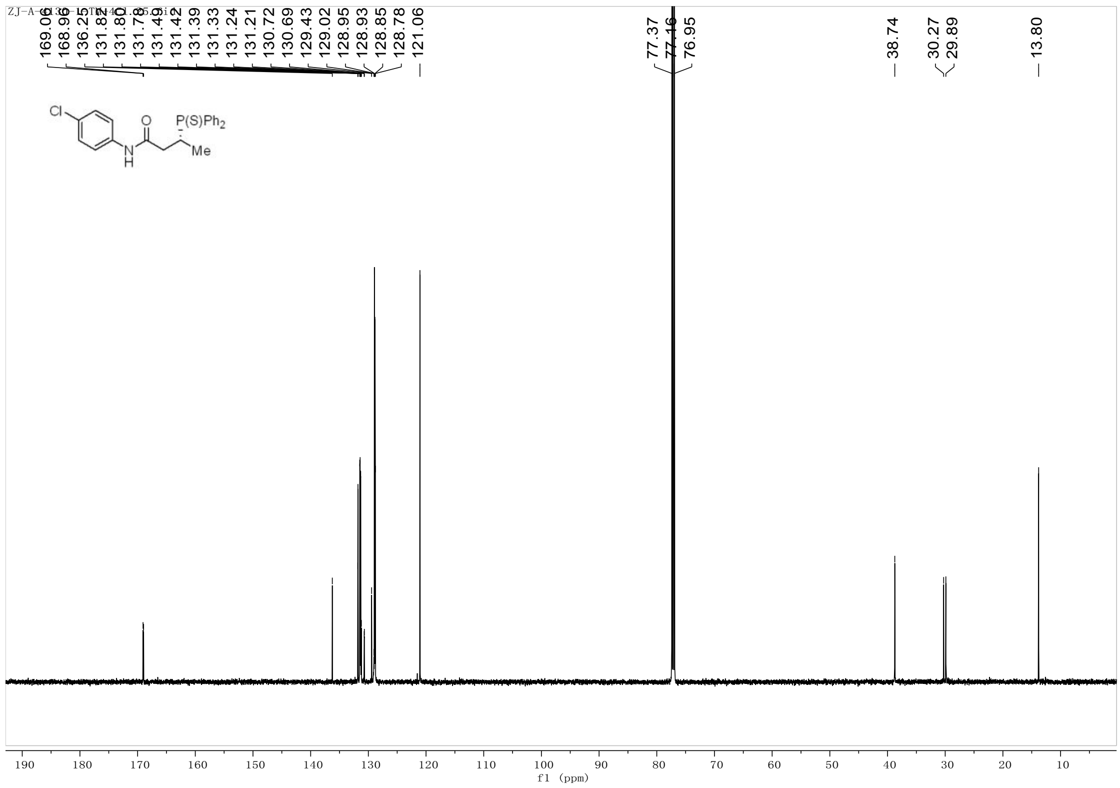

(*R*)-N-(4-bromophenyl)-3-(diphenylphosphorothioyl)butanamide (3j)

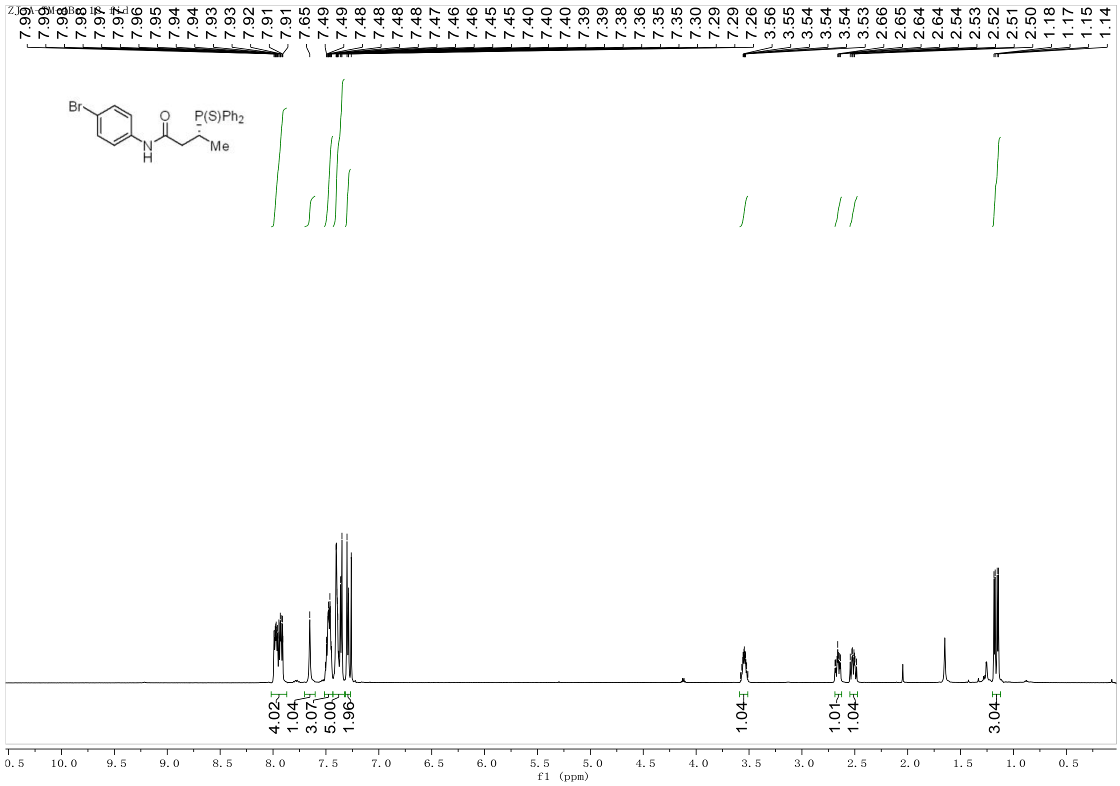

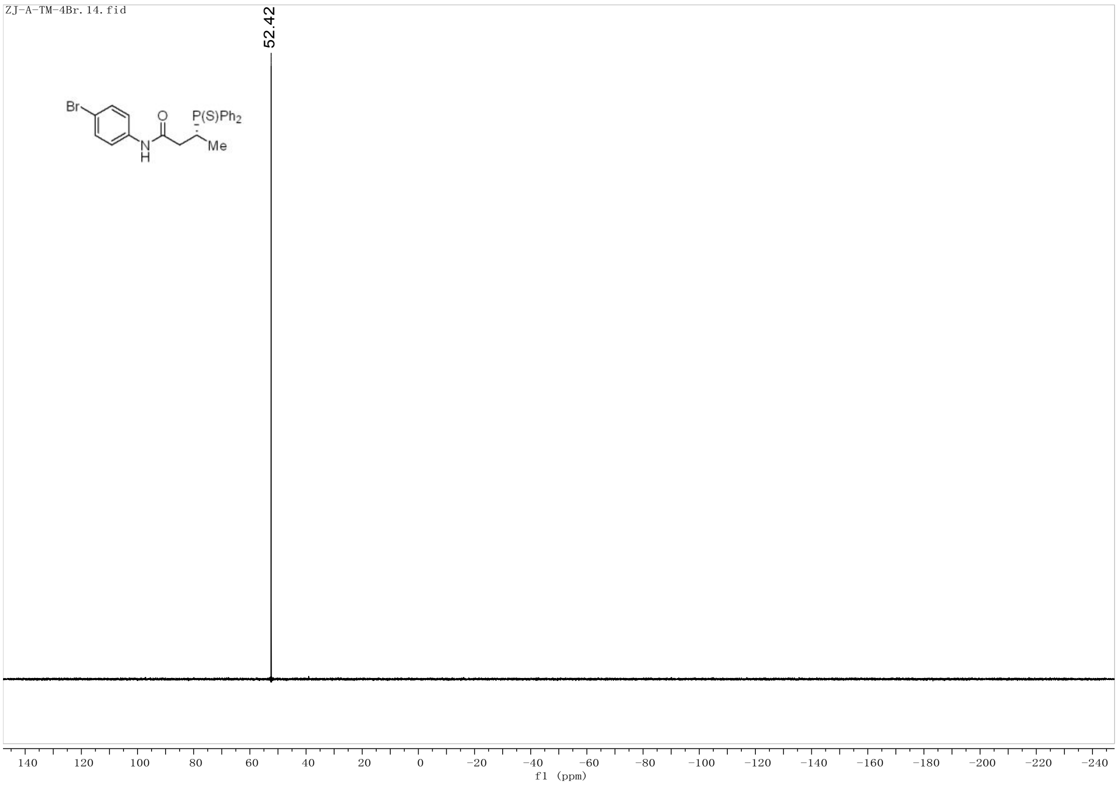
ZJ-A-TM-4Br.14.fid
52.42
f1 (ppm)

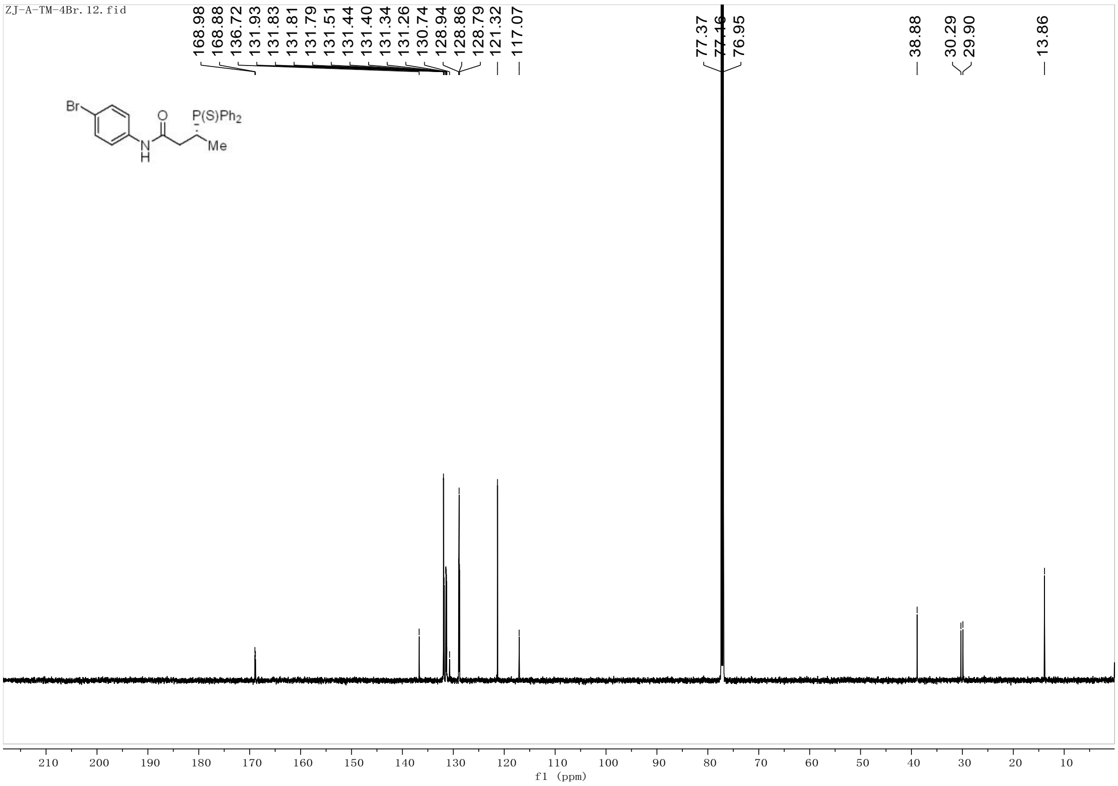
ZJ-A-TM-4Br.12.fid
168.98
168.88
136.72
131.93
131.83
131.81
131.79
131.51
131.44
131.40
131.34
131.26
130.74
128.94
128.86
128.79
121.32
117.07
77.37
77.16
76.95
38.88
30.29
29.90
13.86
f1 (ppm)

(*R*)-3-(diphenylphosphorothioyl)-N-(4-methoxyphenyl)butanamide (3k)

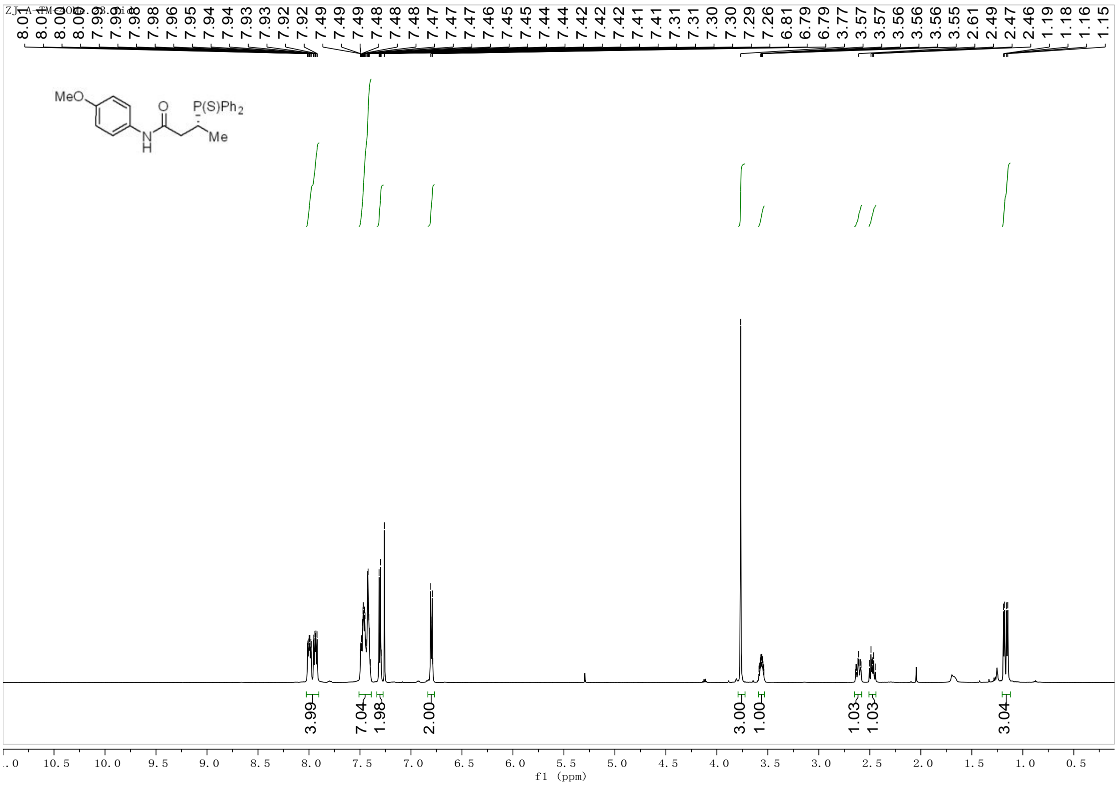

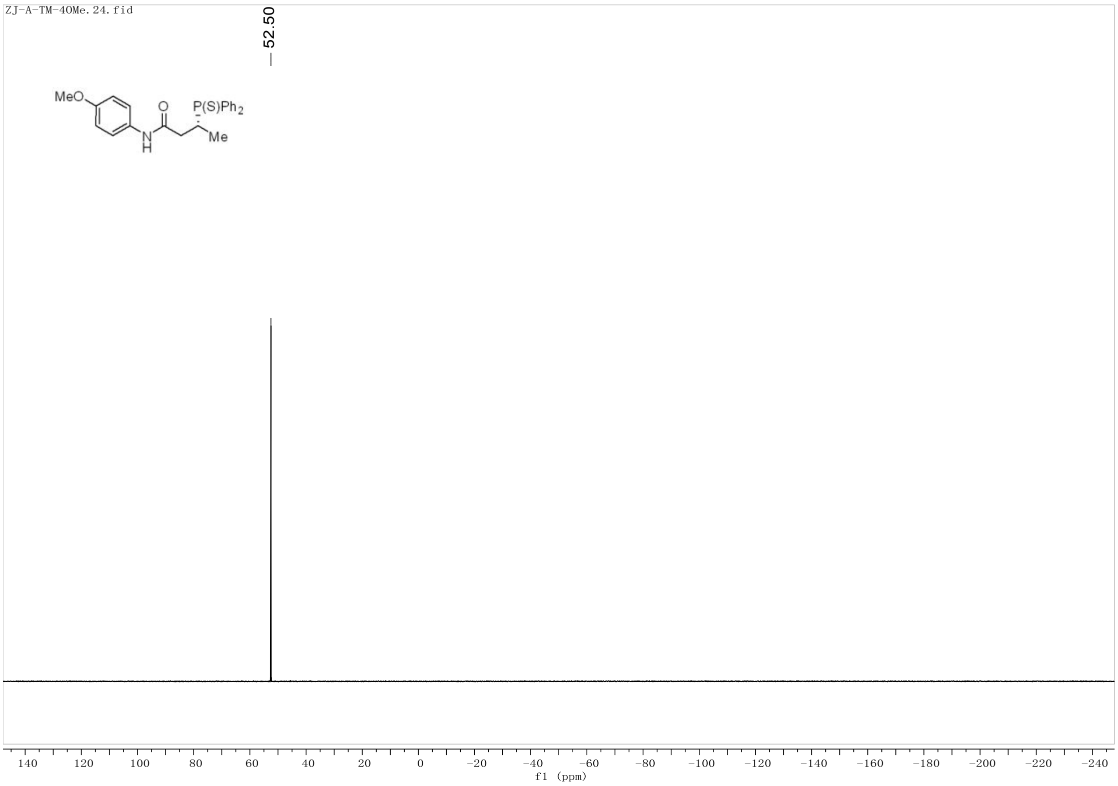

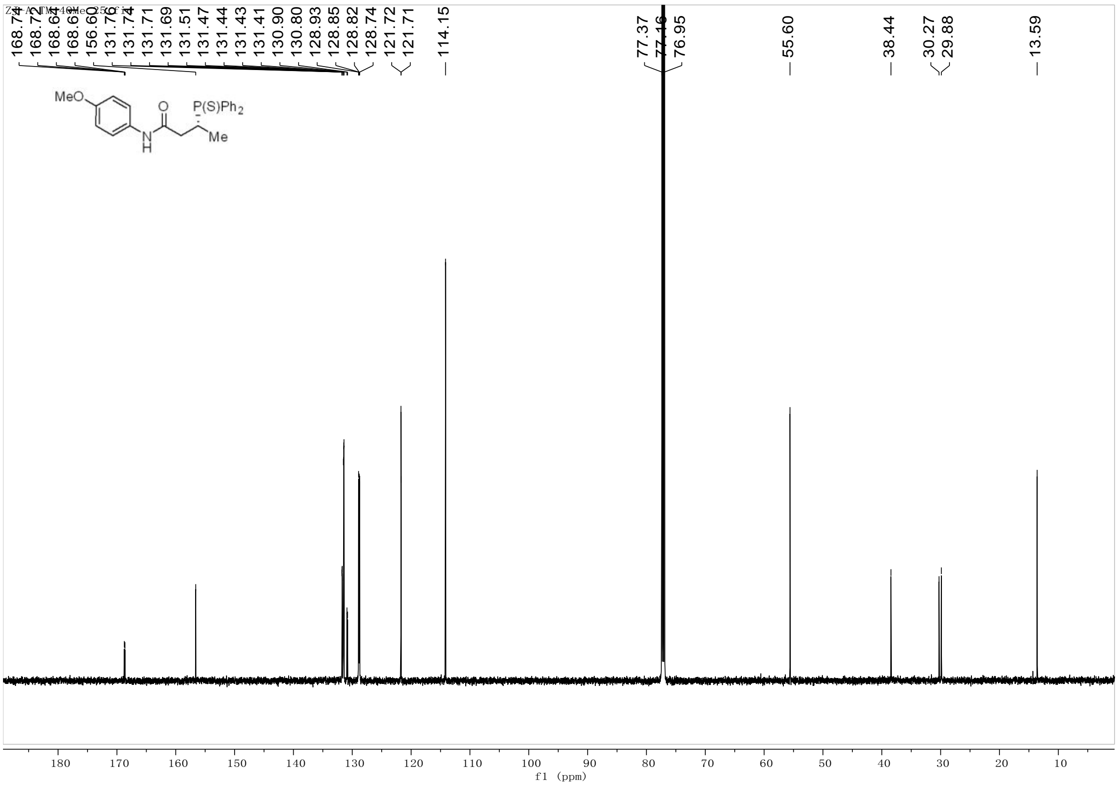

(*R*)-3-(diphenylphosphorothioyl)-N-(4-phenoxyphenyl)butanamide (3l)

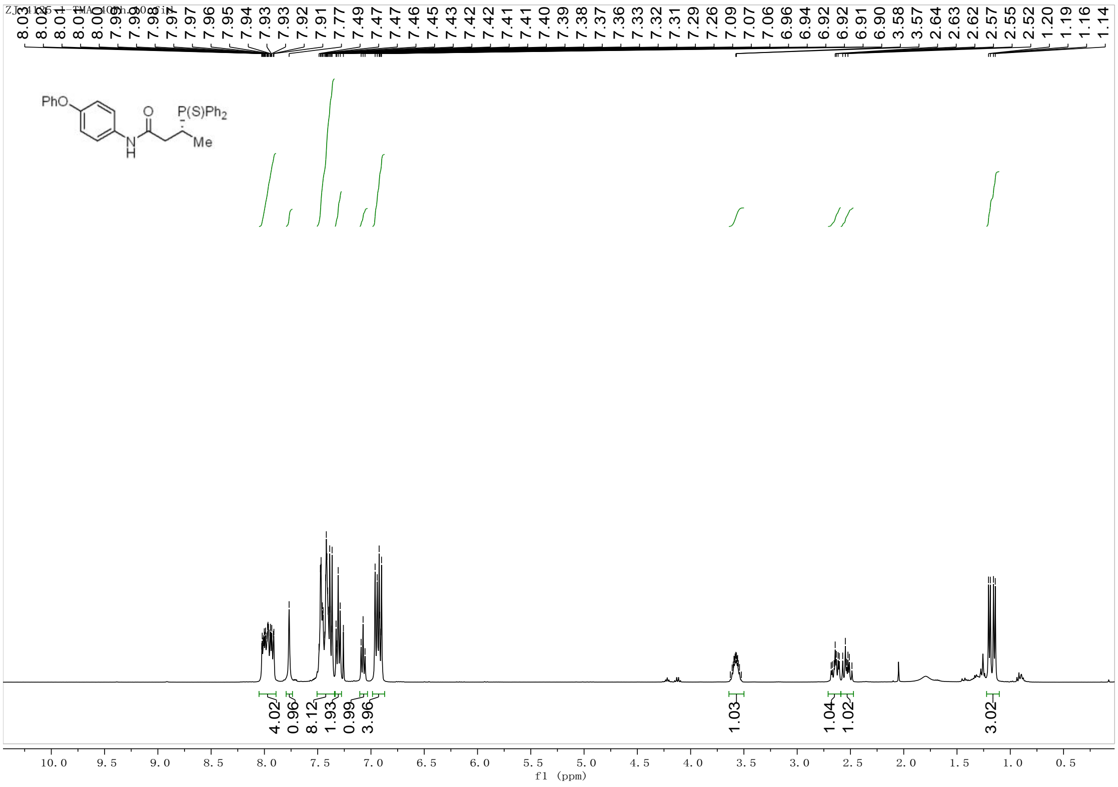

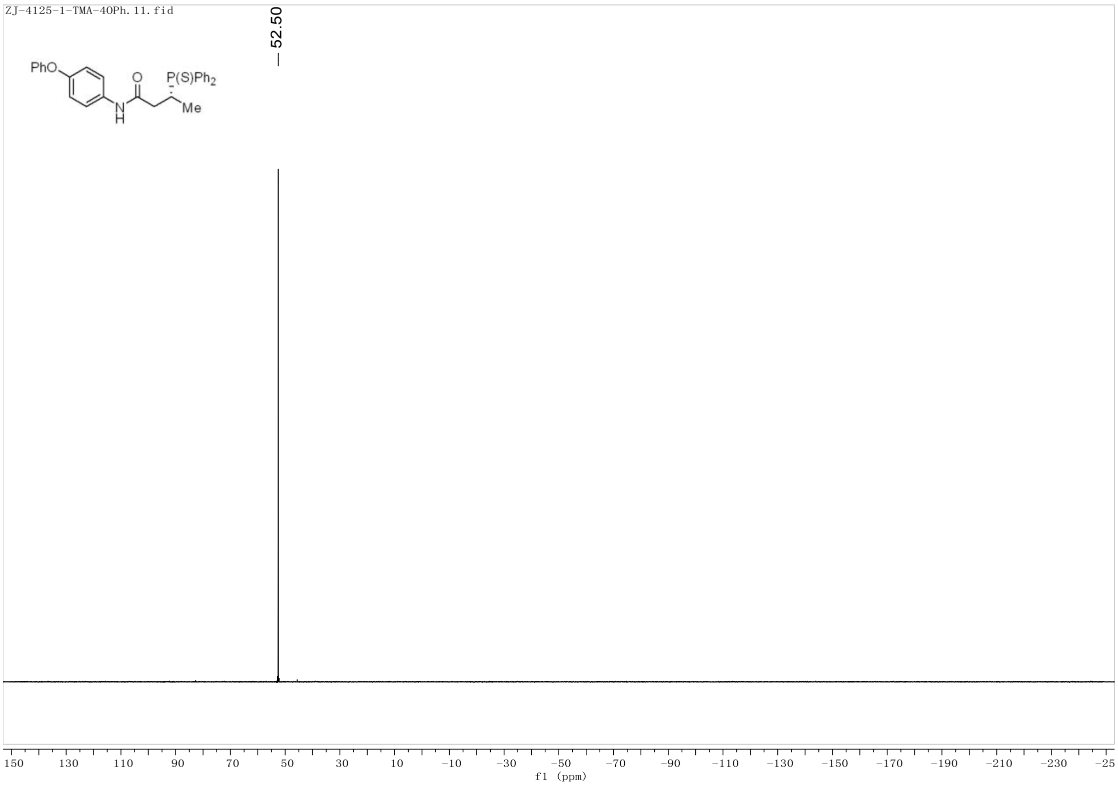
ZJ-4125-1-TMA-4OPh.11.fid
PhO
O
P(S)Ph2
N
H
Me
52.50
150
130
110
90
70
50
30
10
−10
−30
−50
−70
−90
−110
−130
−150
−170
−190
−210
−230
f1 (ppm)

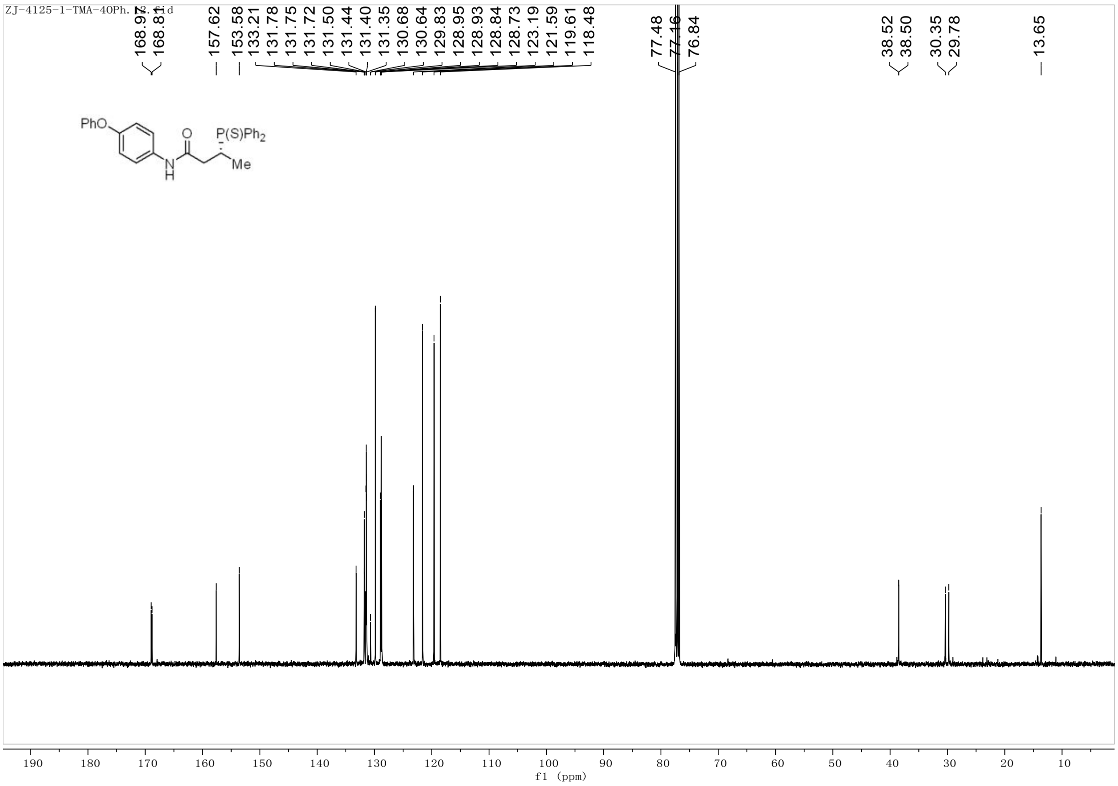
PhO
O
P(S)Ph2
N
H
Me
157.62
153.58
133.21
131.78
131.75
131.72
131.50
131.44
131.40
131.35
130.68
130.64
129.83
128.95
128.93
128.84
128.73
123.19
121.59
119.61
118.48
77.48
76.84
38.52
38.50
30.35
29.78
13.65
190
180
170
160
150
140
130
120
110
100
90
80
70
60
50
40
30
20
10
f1 (ppm)

(*R*)-3-(diphenylphosphorothioyl)-N-(4-(trifluoromethoxy)phenyl)butanamide (3m)

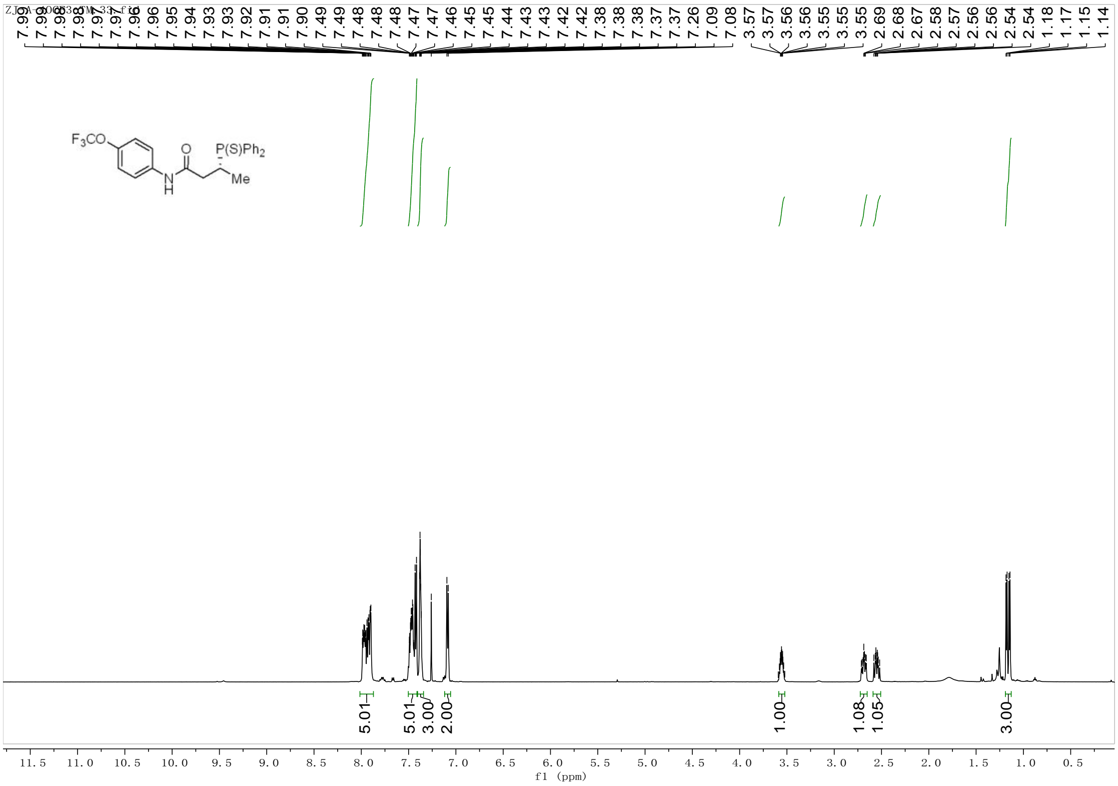

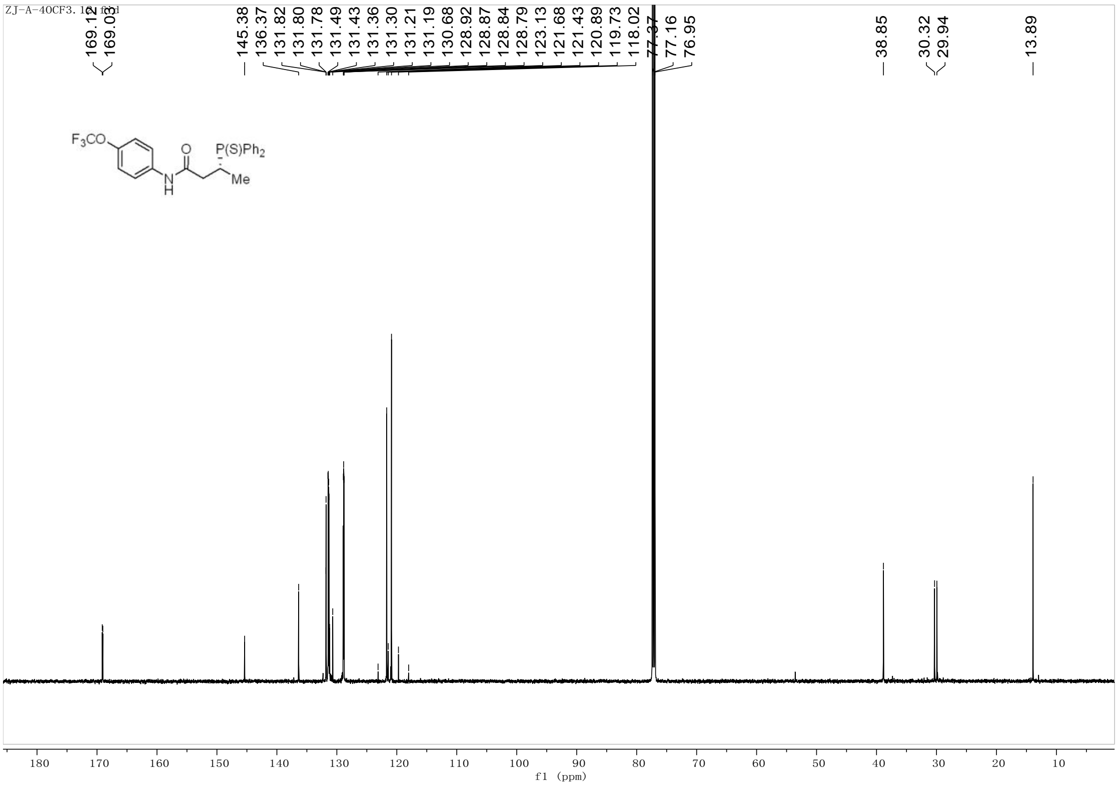

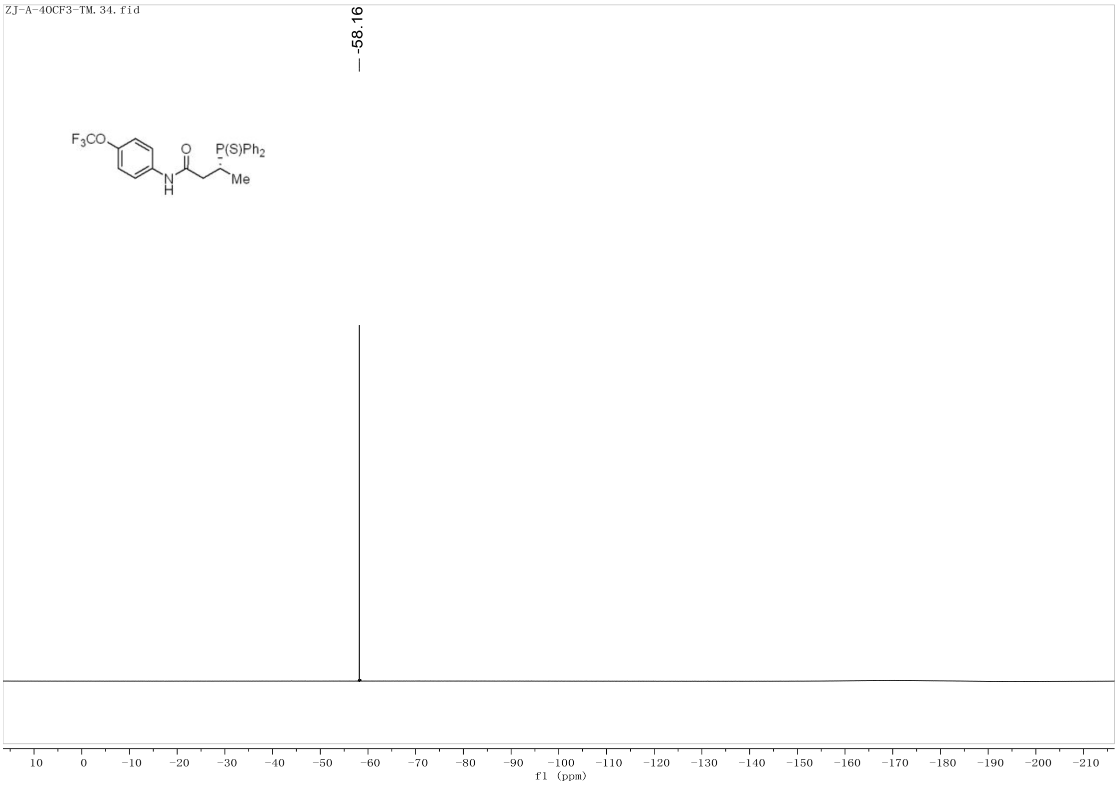
ZJ-A-4OCF3-TM.34.fid
-58.16
F3CO
O
N
H
P(S)Ph2
Me
10 0 −10 −20 −30 −40 −50 −60 −70 −80 −90 −100 −110 −120 −130 −140 −150 −160 −170 −180 −190 −200 −210
f1 (ppm)

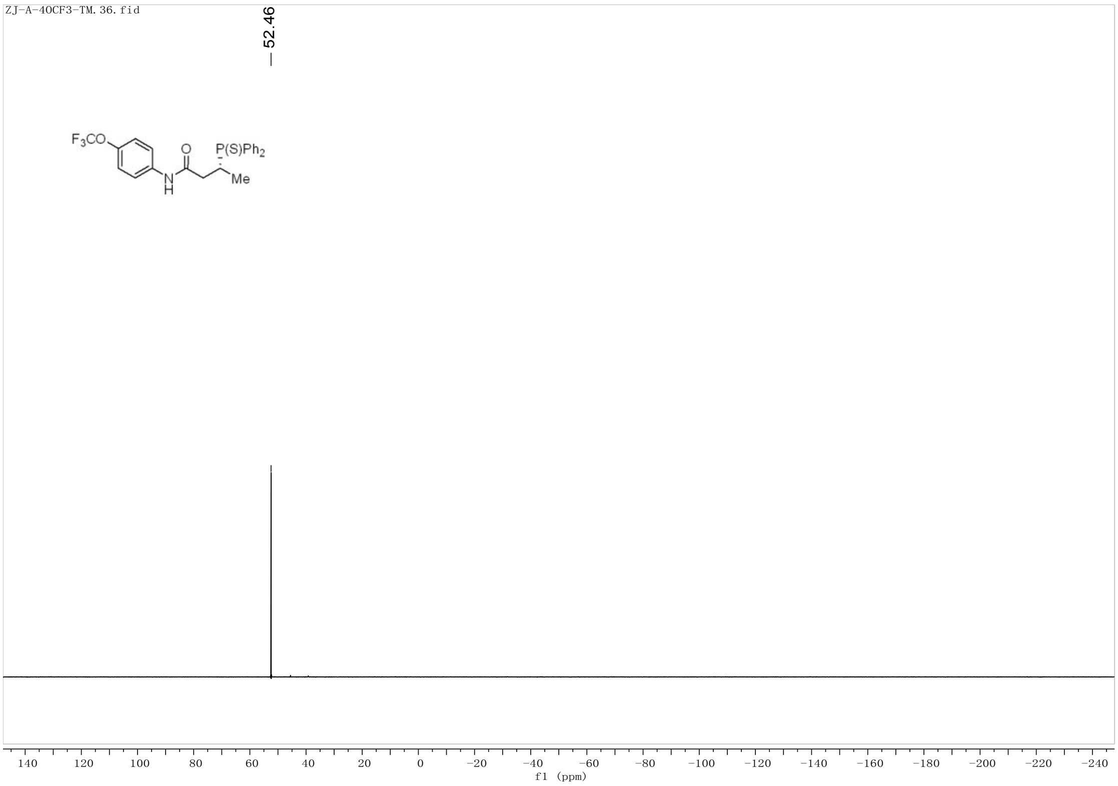
ZJ-A-4OCF3-TM.36.fid
52.46
F3CO
O
N
H
P(S)Ph2
Me
140 120 100 80 60 40 20 0 −20 −40 −60 −80 −100 −120 −140 −160 −180 −200 −220 −240
f1 (ppm)

(*R*)-3-(diphenylphosphorothioyl)-N-(4-vinylphenyl)butanamide (3n)

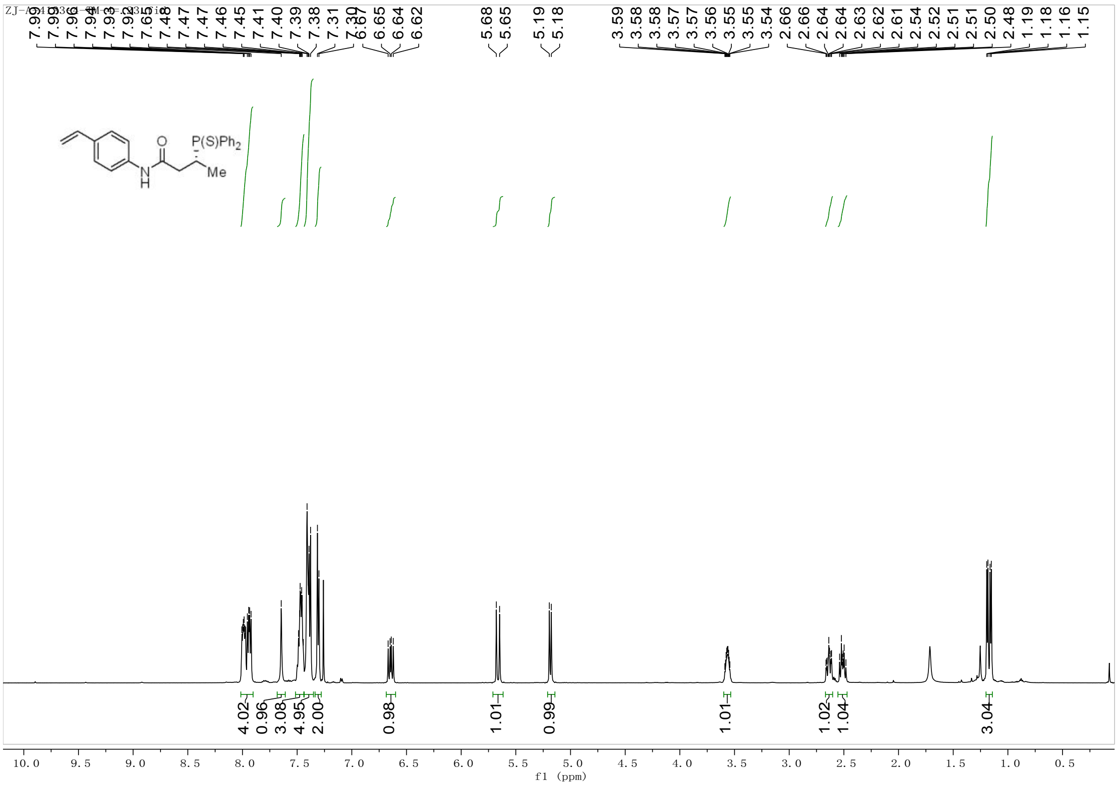

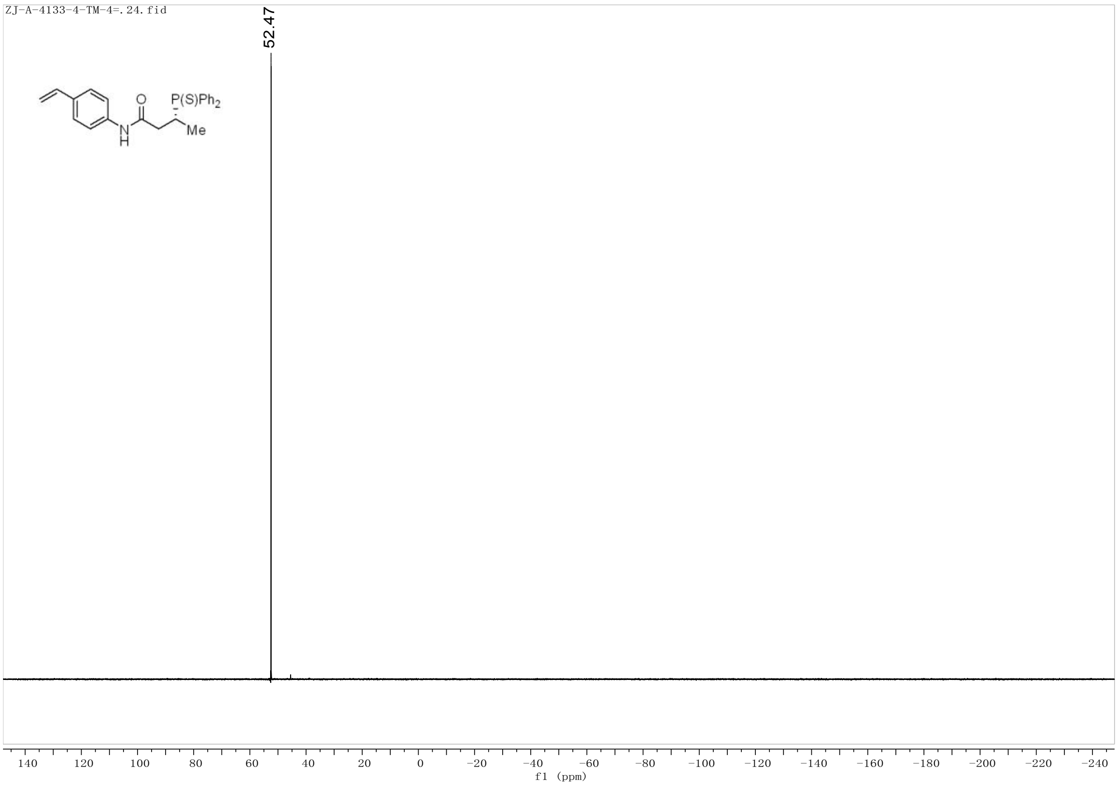

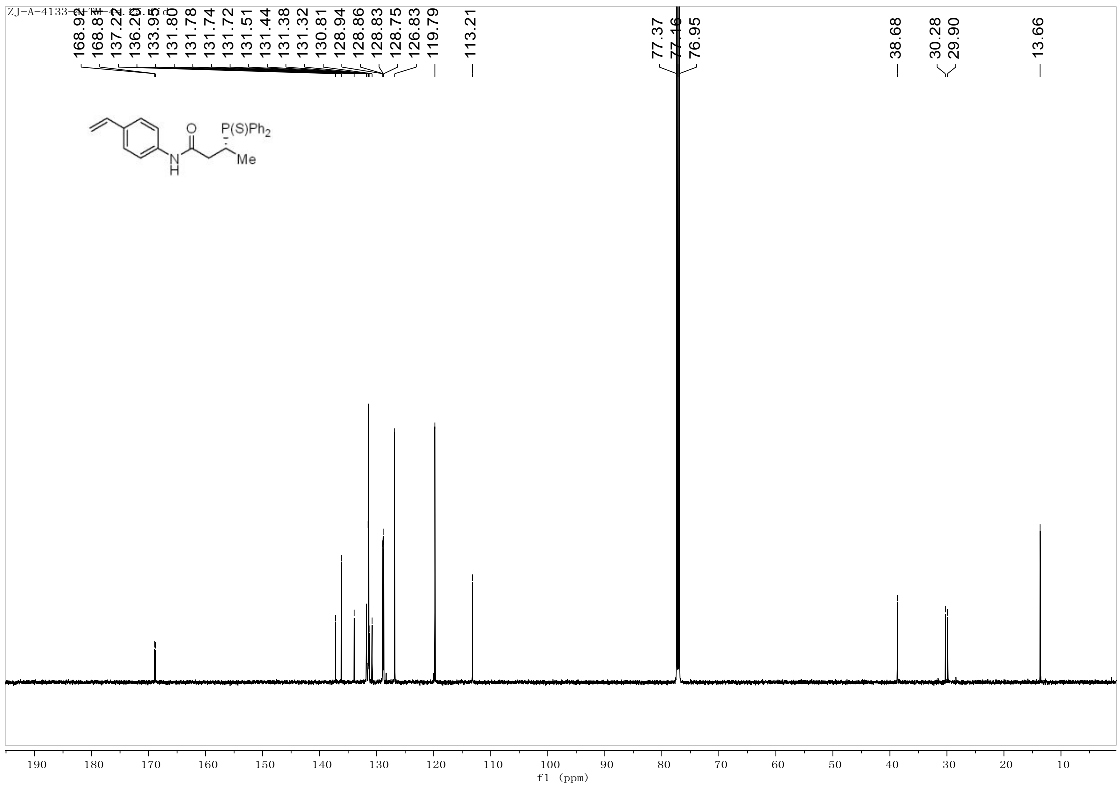

(*R*)-N-(3,5-difluorophenyl)-3-(diphenylphosphorothioyl)butanamide (3o)

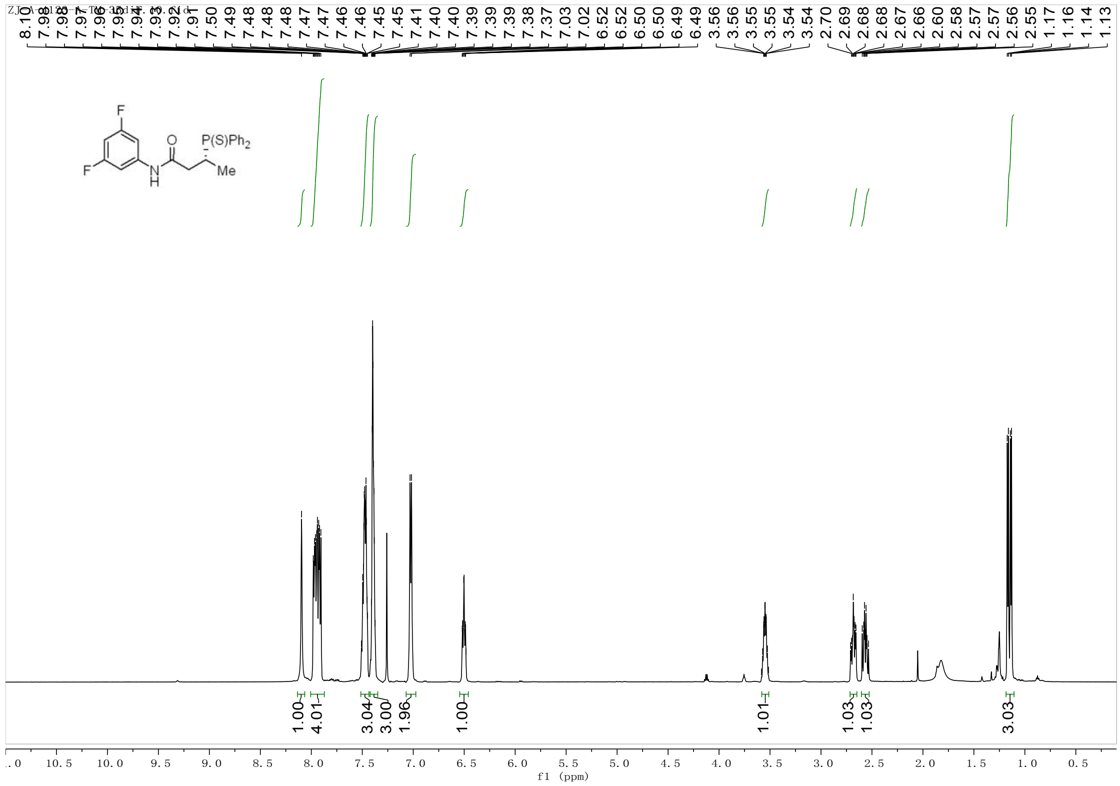

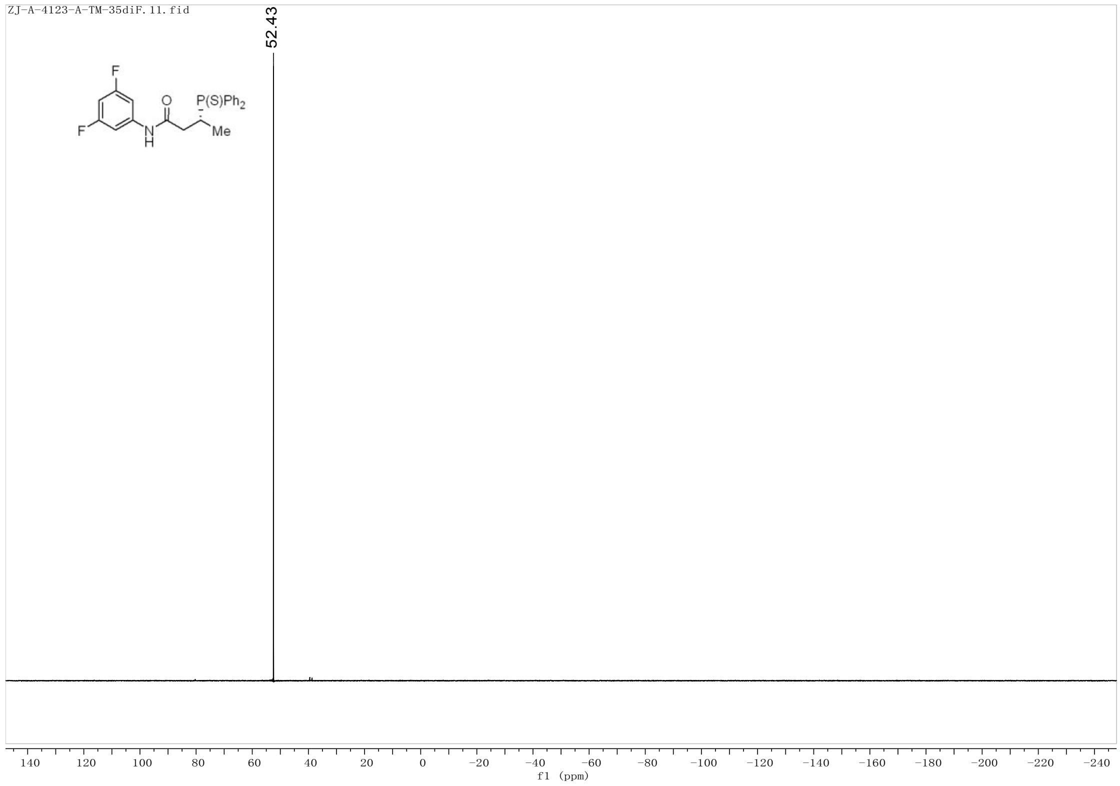
ZJ-A-4123-A-TM-35diF.11.fid
52.43
F
F
N
H
O
P(S)Ph2
Me
140
120
100
80
60
40
20
0
-20
-40
-60
-80
-100
-120
-140
-160
-180
-200
-220
-240
f1 (ppm)

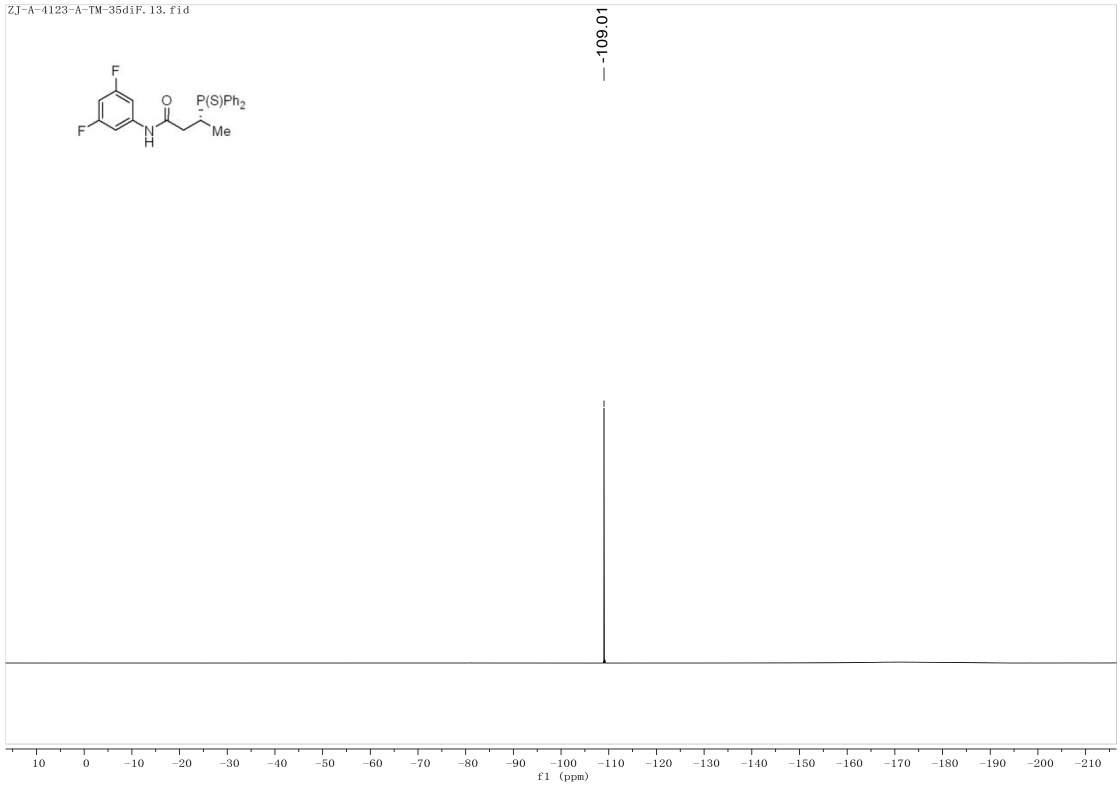
ZJ-A-4123-A-TM-35diF.13.fid
-109.01
F
F
N
H
O
P(S)Ph2
Me
10
0
-10
-20
-30
-40
-50
-60
-70
-80
-90
-100
-110
-120
-130
-140
-150
-160
-170
-180
-190
-200
-210
f1 (ppm)

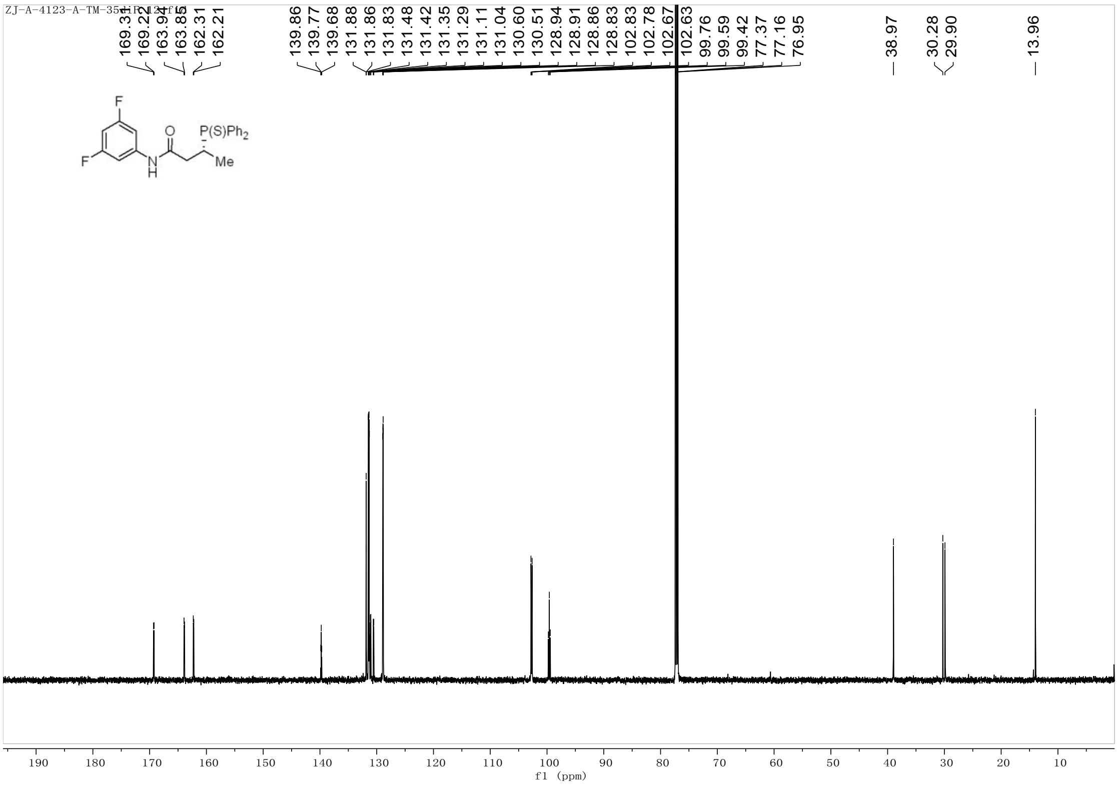

(*R*)-3-(diphenylphosphorothioyl)-N-(4-morpholinophenyl)butanamide (3p)

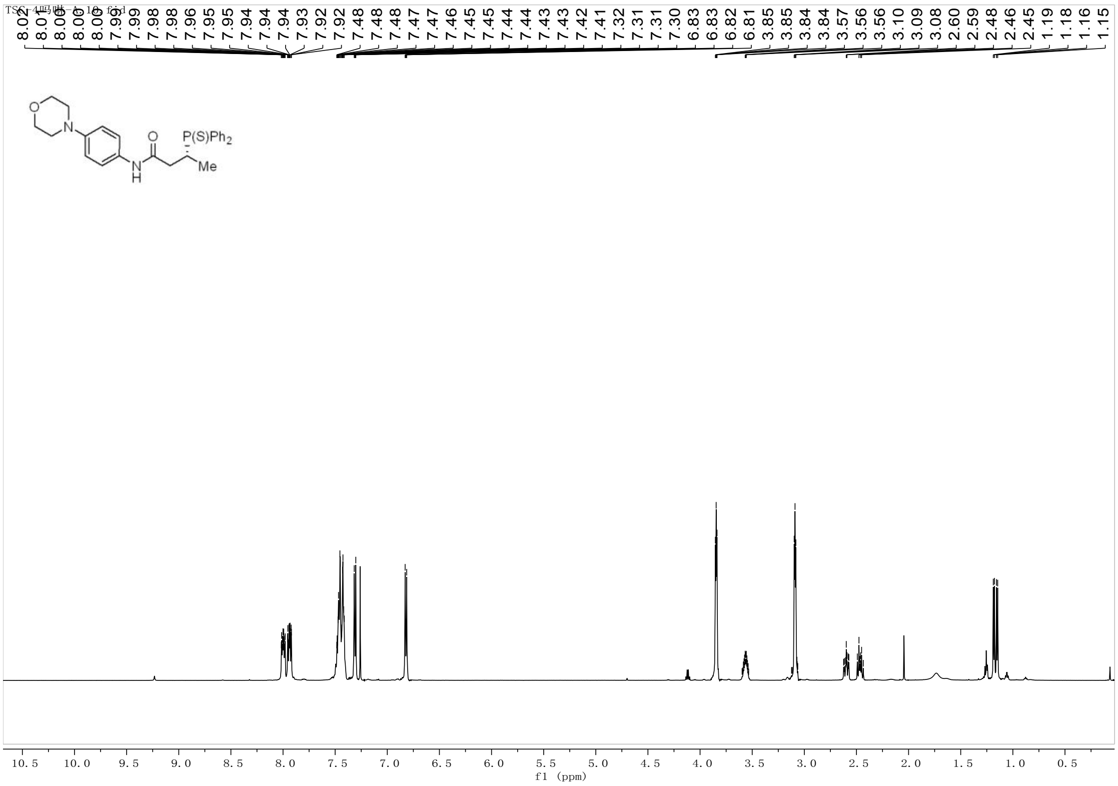

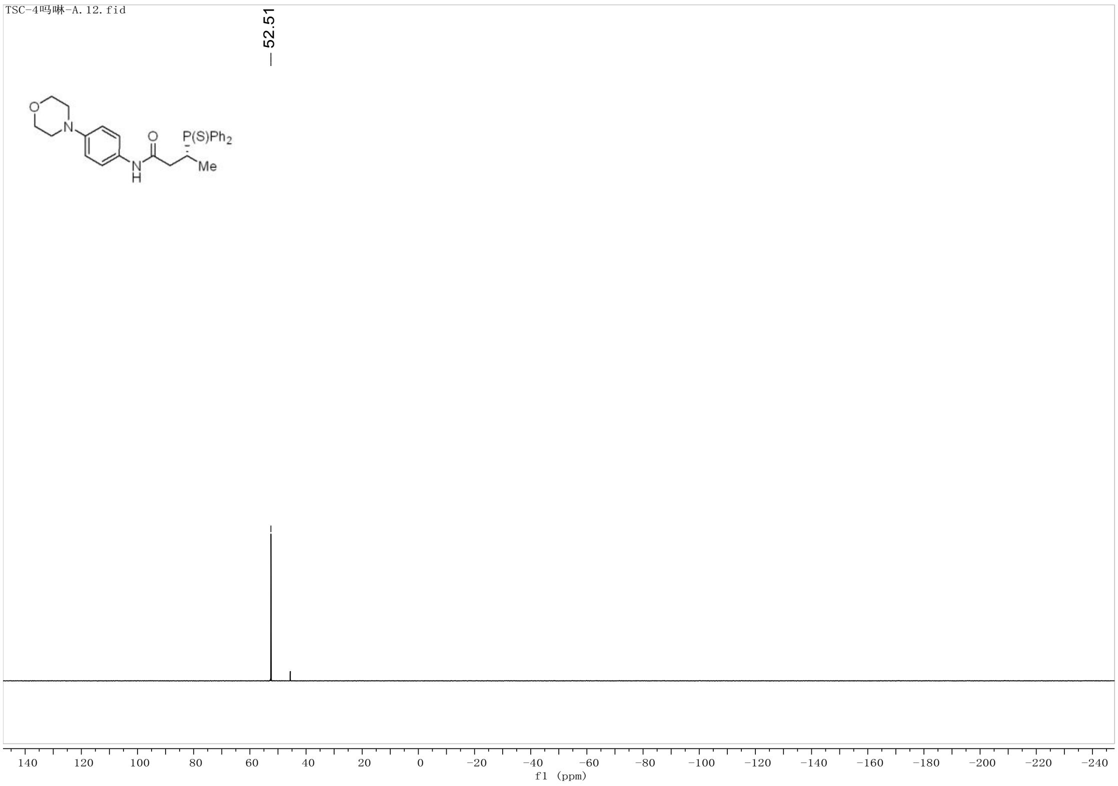
TSC-4吗啉-A.12.fid
52.51
P(S)Ph2
Me
O
N
H
f1 (ppm)

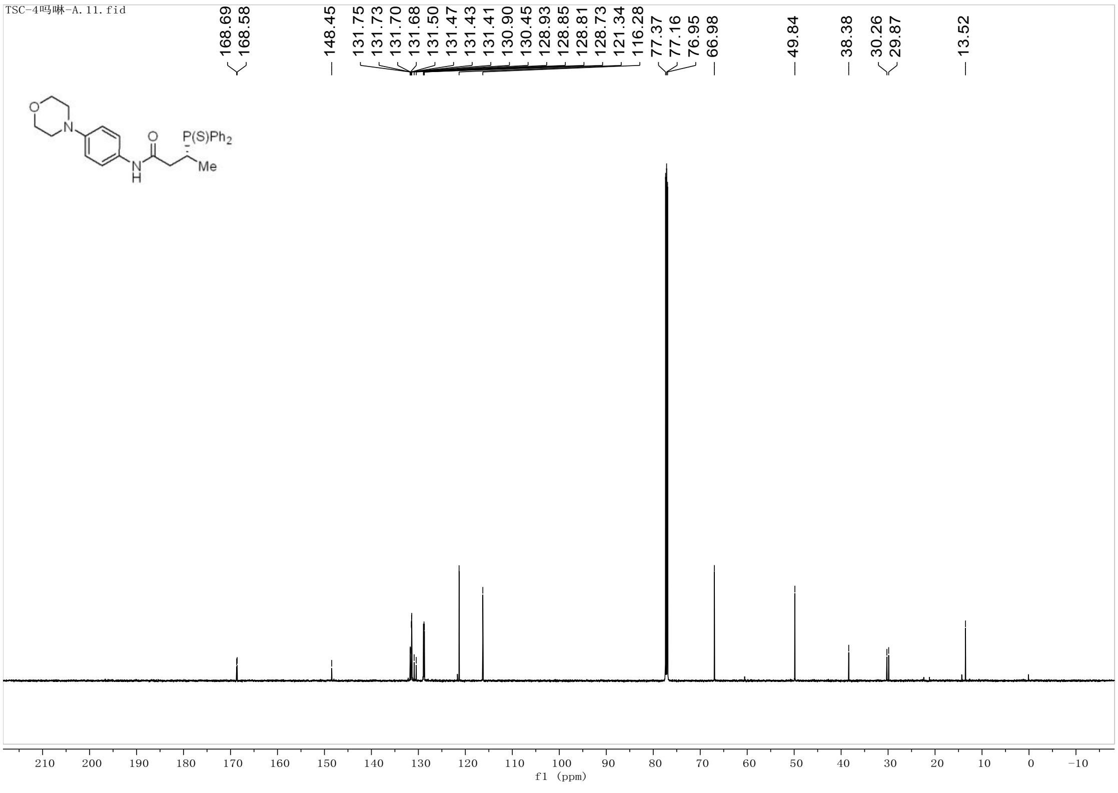
TSC-4吗啉-A.11.fid
168.69
168.58
148.45
131.75
131.73
131.70
131.68
131.50
131.47
131.43
131.41
130.90
130.45
128.93
128.85
128.81
128.73
121.34
116.28
77.37
77.16
76.95
66.98
49.84
38.38
30.26
29.87
13.52
P(S)Ph2
Me
f1 (ppm)

(*R*)-N-(4-acetylphenyl)-3-(diphenylphosphorothioyl)butanamide (3q)

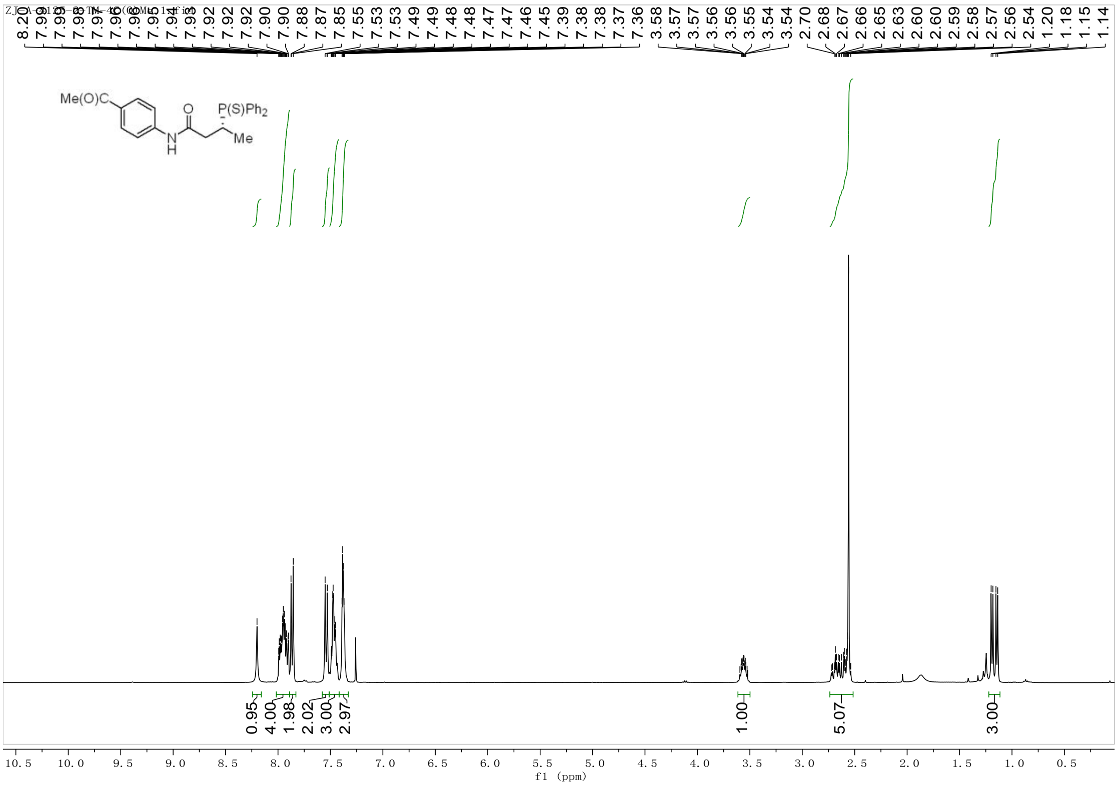

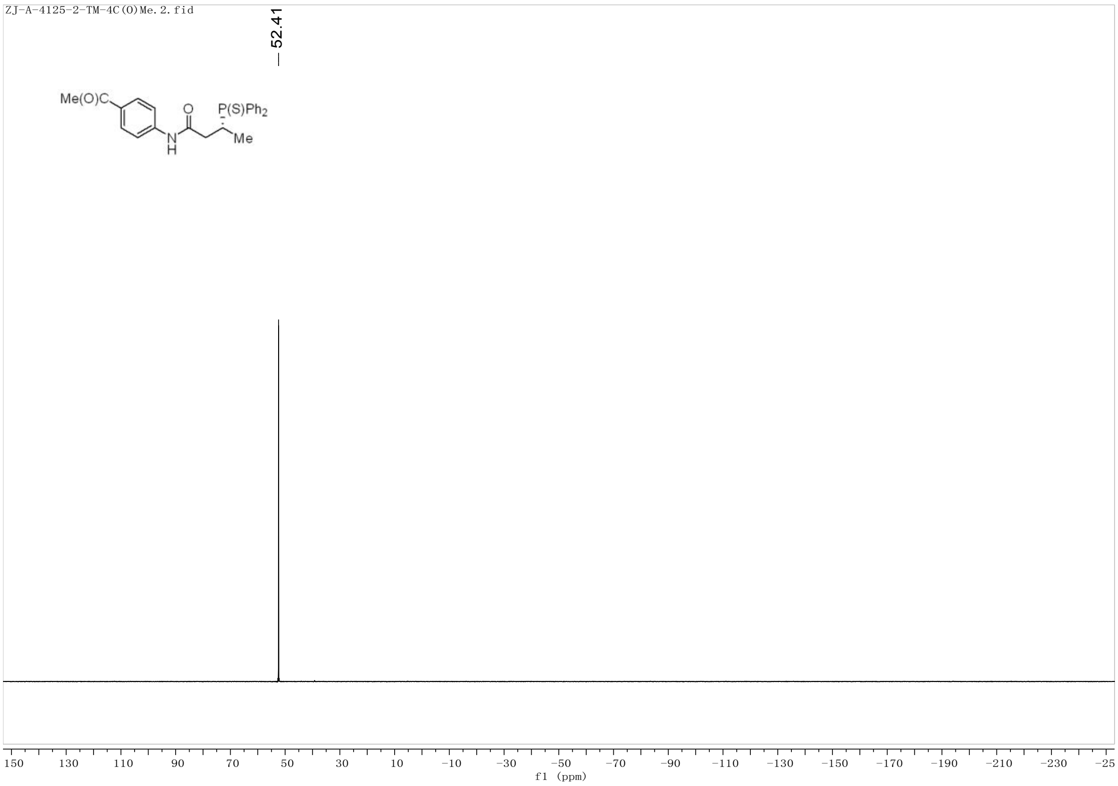

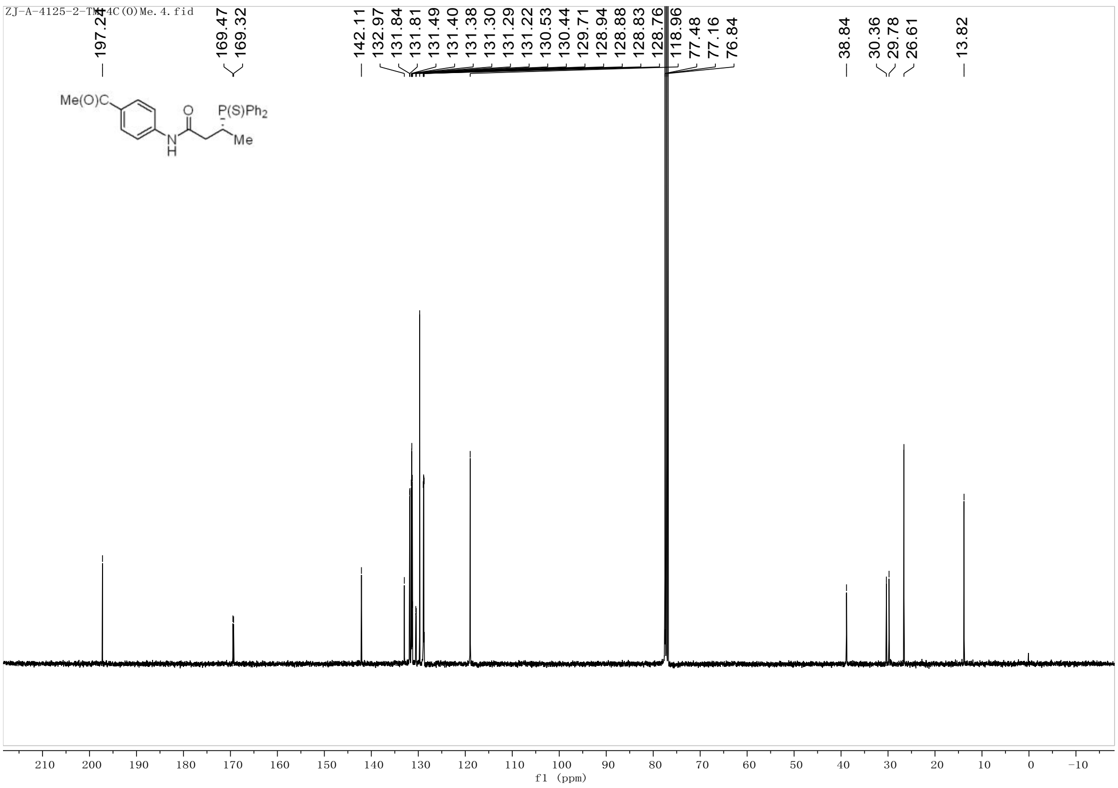

methyl (*R*)-4-(3-(diphenylphosphorothioyl)butanamido)benzoate (3r)

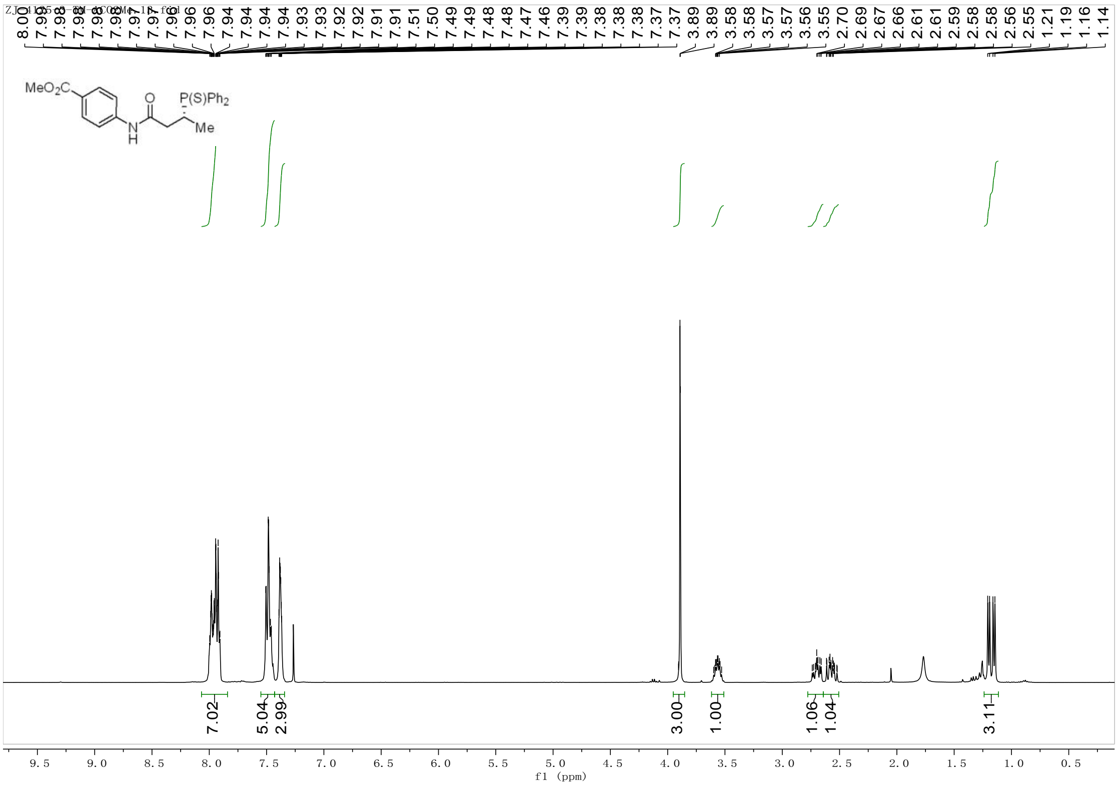

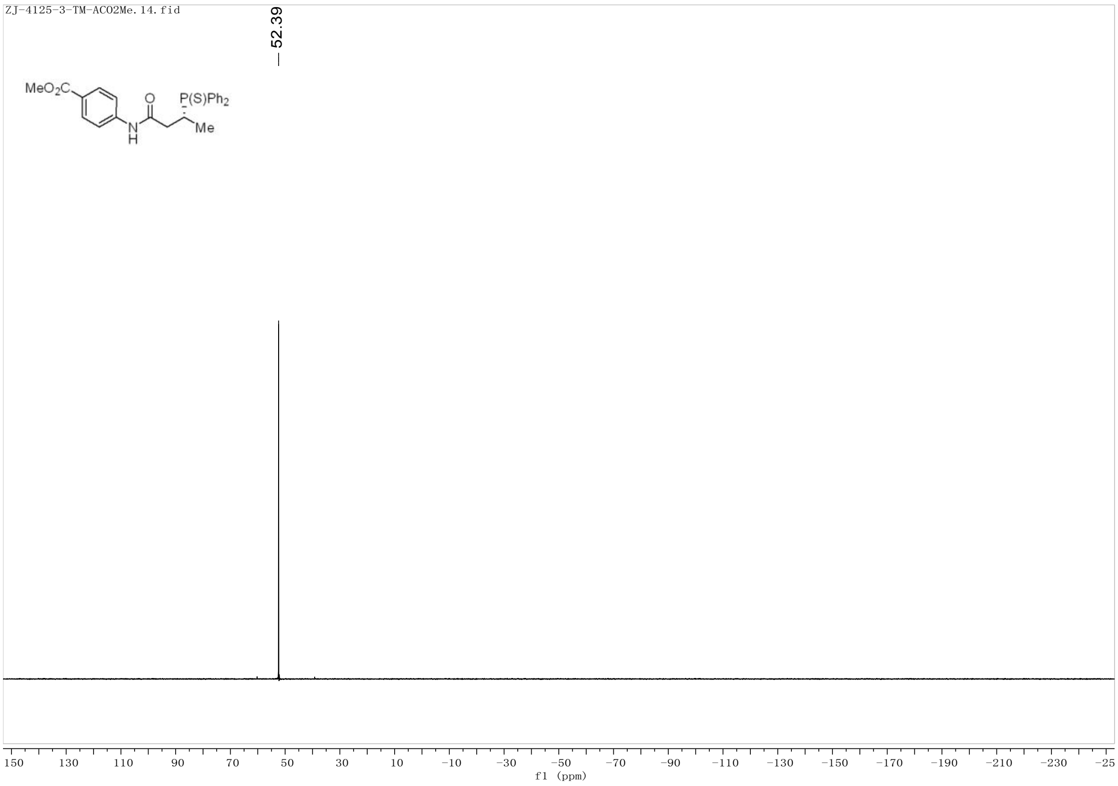
ZJ-4125-3-TM-ACO2Me.14.fid
52.39
MeO2C
O
N
H
P(S)Ph2
Me
f1 (ppm)

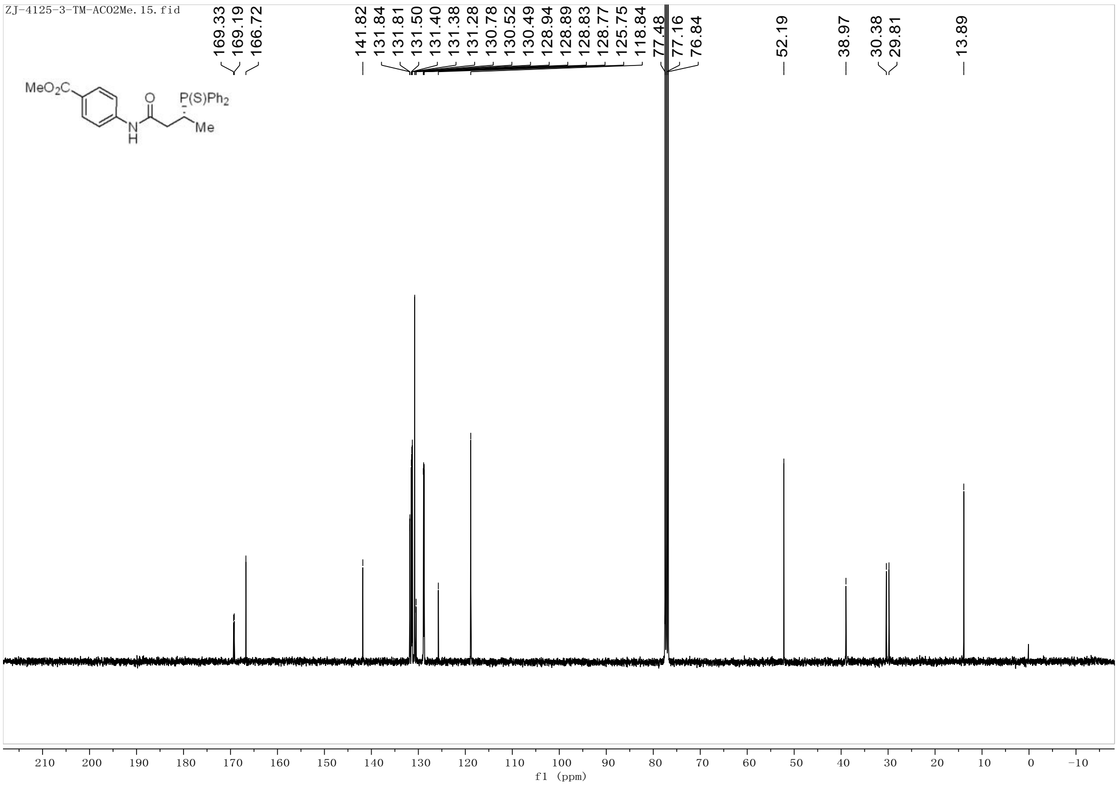
ZJ-4125-3-TM-ACO2Me.15.fid
169.33
169.19
166.72
141.82
131.84
131.81
131.50
131.40
131.38
131.28
130.78
130.52
130.49
128.94
128.89
128.83
128.77
125.75
118.84
77.48
77.16
76.84
52.19
38.97
30.38
29.81
13.89
MeO2C
O
N
H
P(S)Ph2
Me
f1 (ppm)

(*R*)-3-(diphenylphosphorothioyl)-N-(naphthalen-2-yl)butanamide (3s)

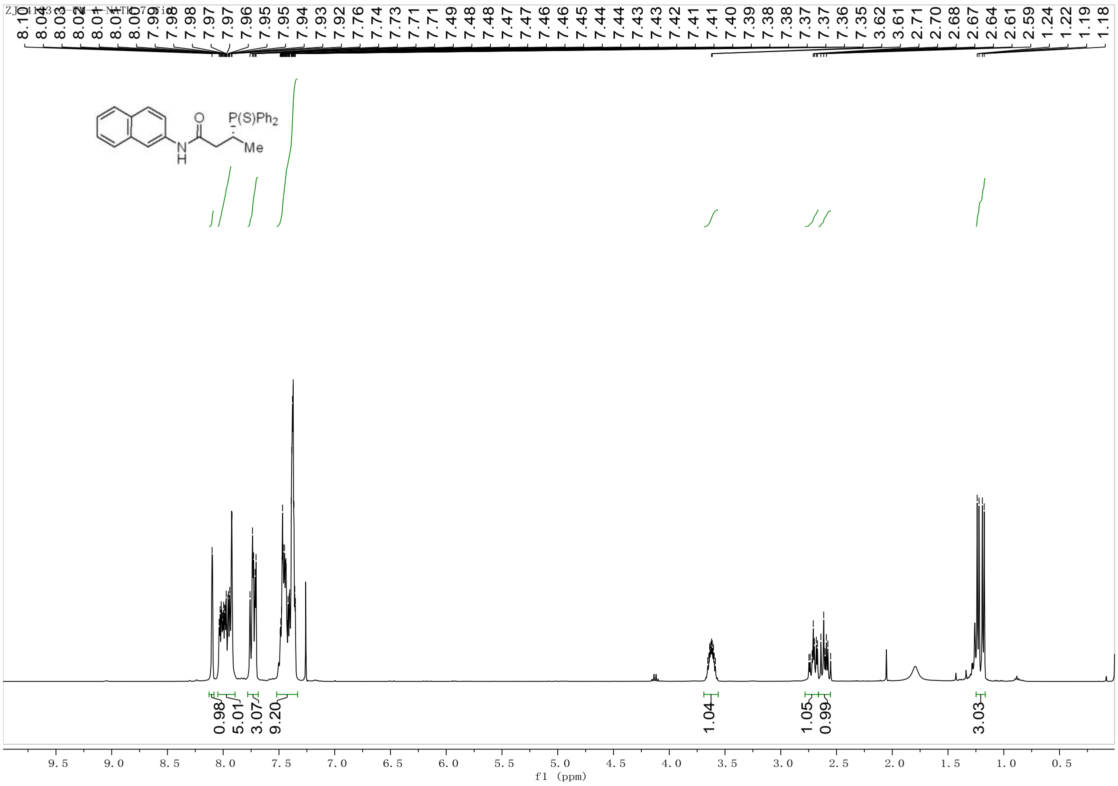

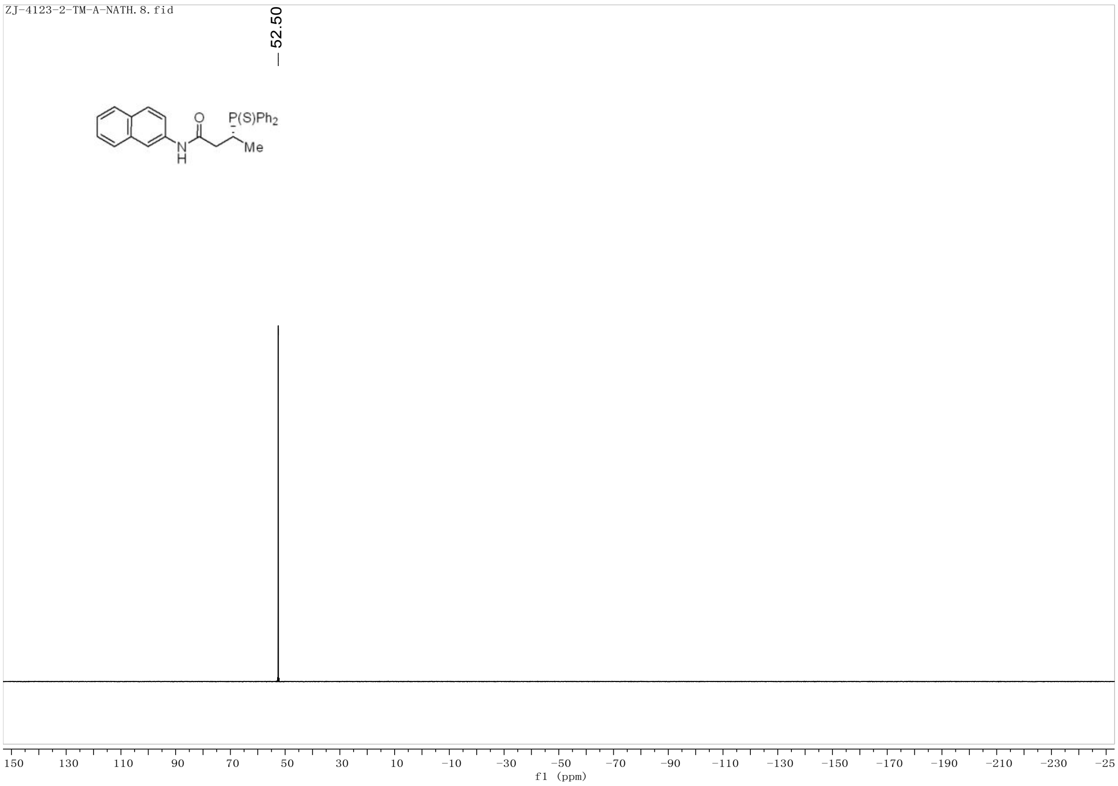

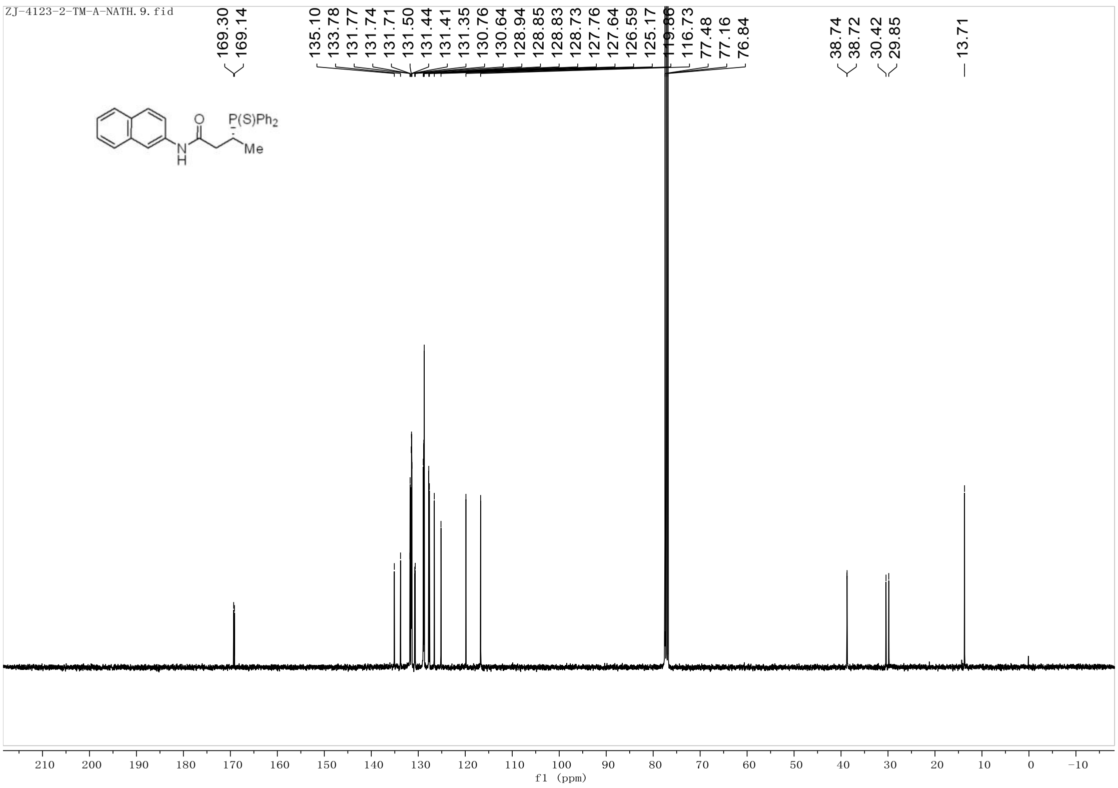

(*R*)-N-(dibenzo[b,d]furan-2-yl)-3-(diphenylphosphorothioyl)butanamide (3t)

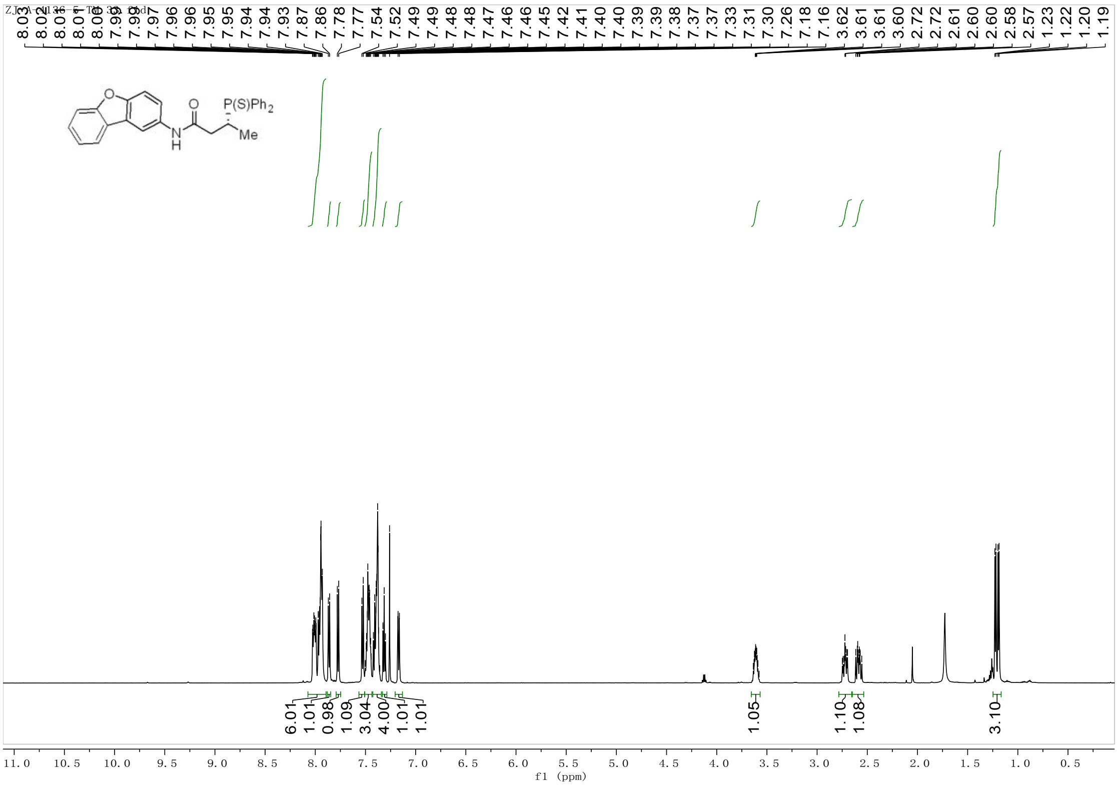

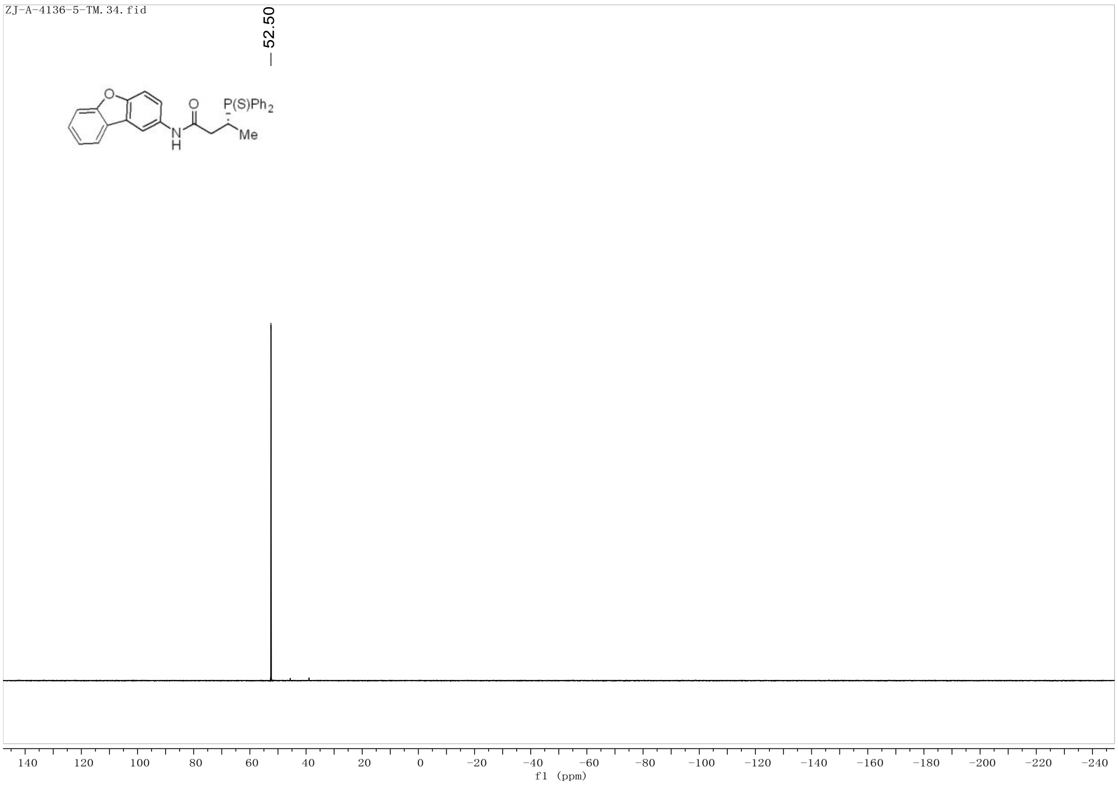
ZJ-A-4136-5-TM.34.fid
52.50
P(S)Ph2
Me
O
N
H
f1 (ppm)

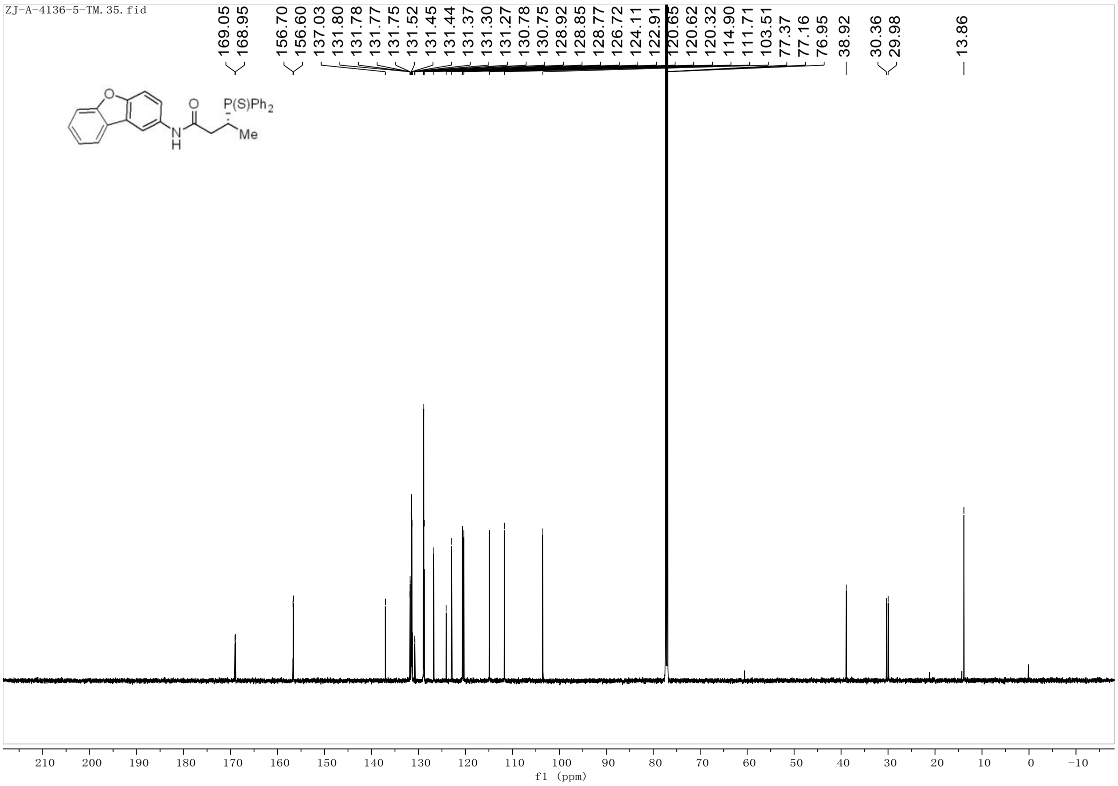
ZJ-A-4136-5-TM.35.fid
169.05
168.95
156.70
156.60
137.03
131.80
131.78
131.77
131.75
131.52
131.45
131.44
131.37
131.30
131.27
130.78
130.75
128.92
128.85
128.77
126.72
124.11
122.91
120.65
120.62
120.32
114.90
111.71
103.51
77.37
77.16
76.95
38.92
30.36
29.98
13.86
P(S)Ph2
Me
f1 (ppm)

(*R*)-3-(diphenylphosphorothioyl)-N-(isoquinolin-4-yl)butanamide (3u)

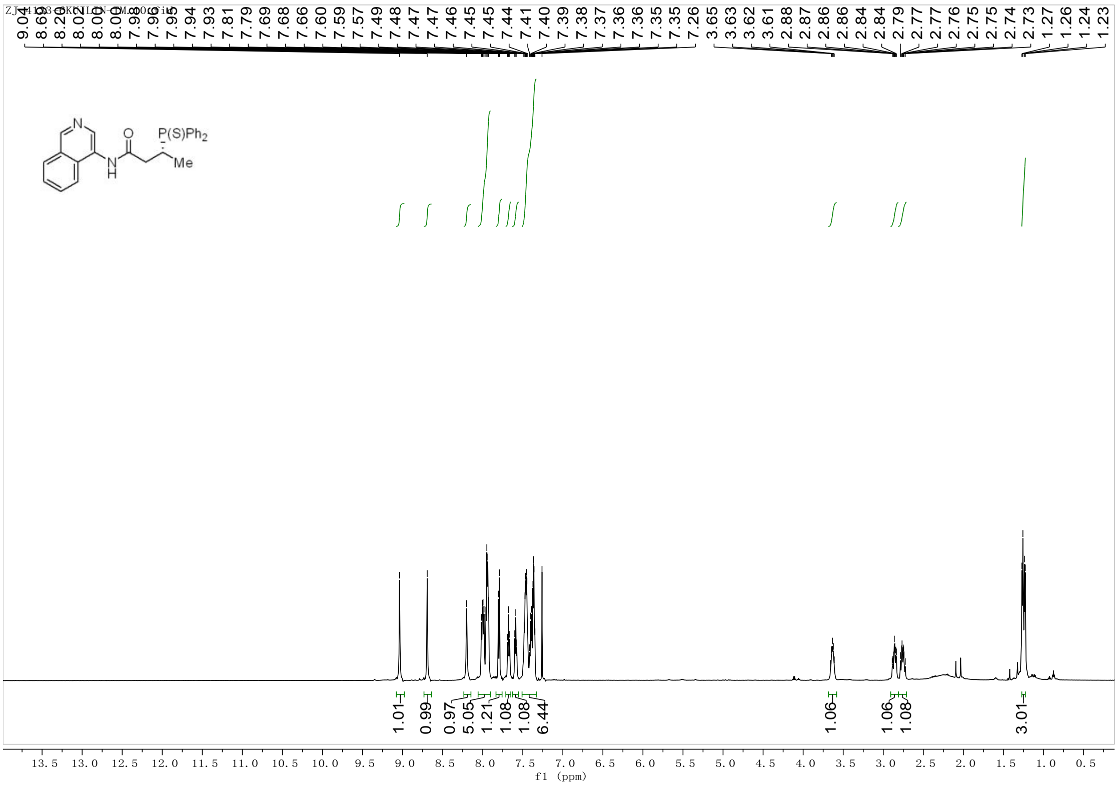

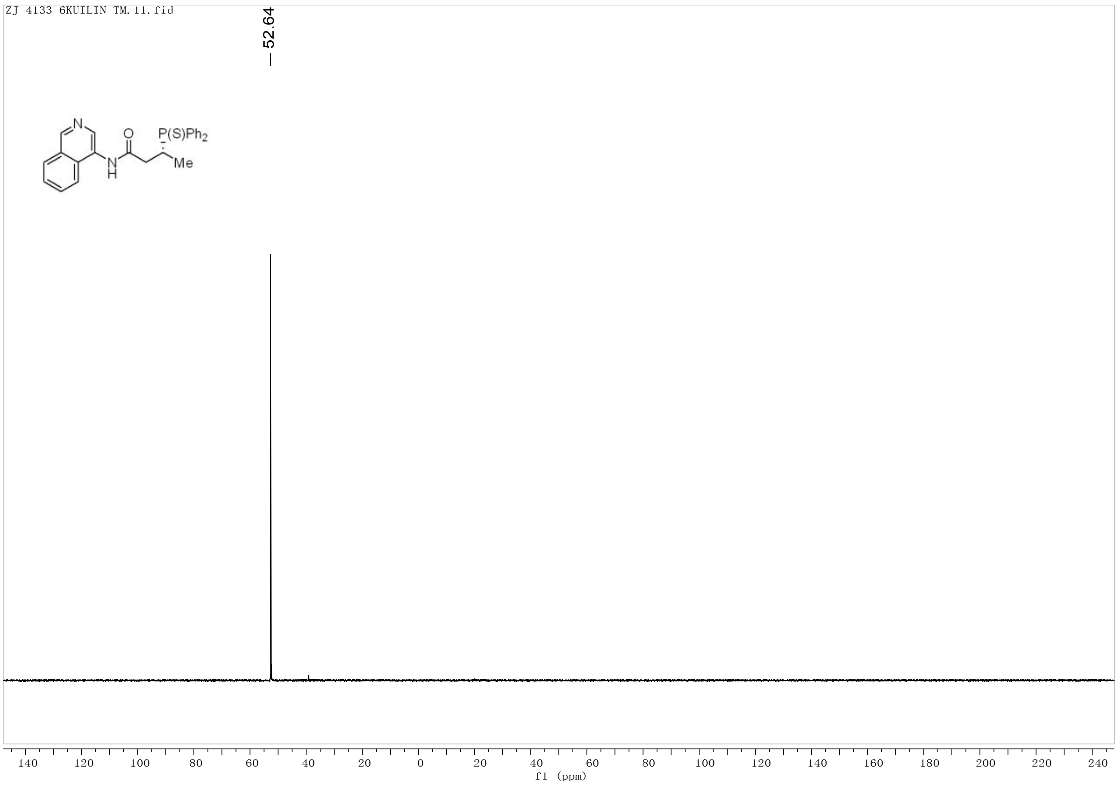

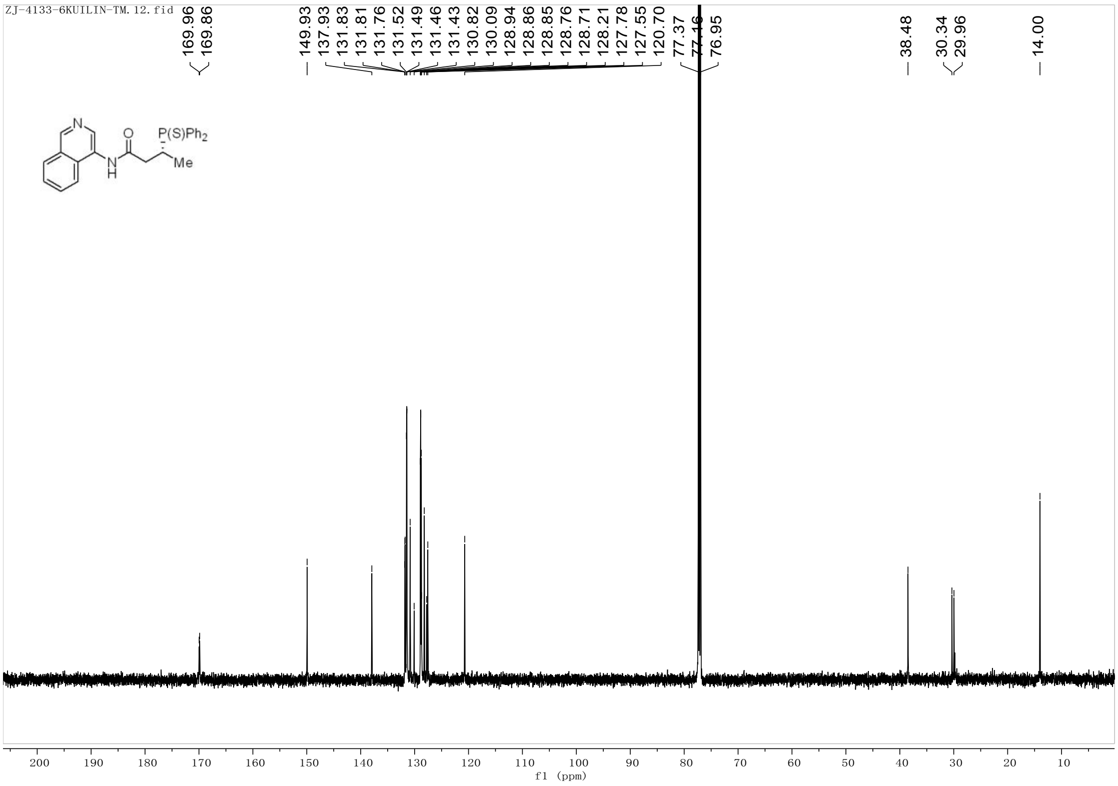

(*R*)-3-(diphenylphosphorothioyl)-N-(thiophen-3-yl)butanamide (3v)

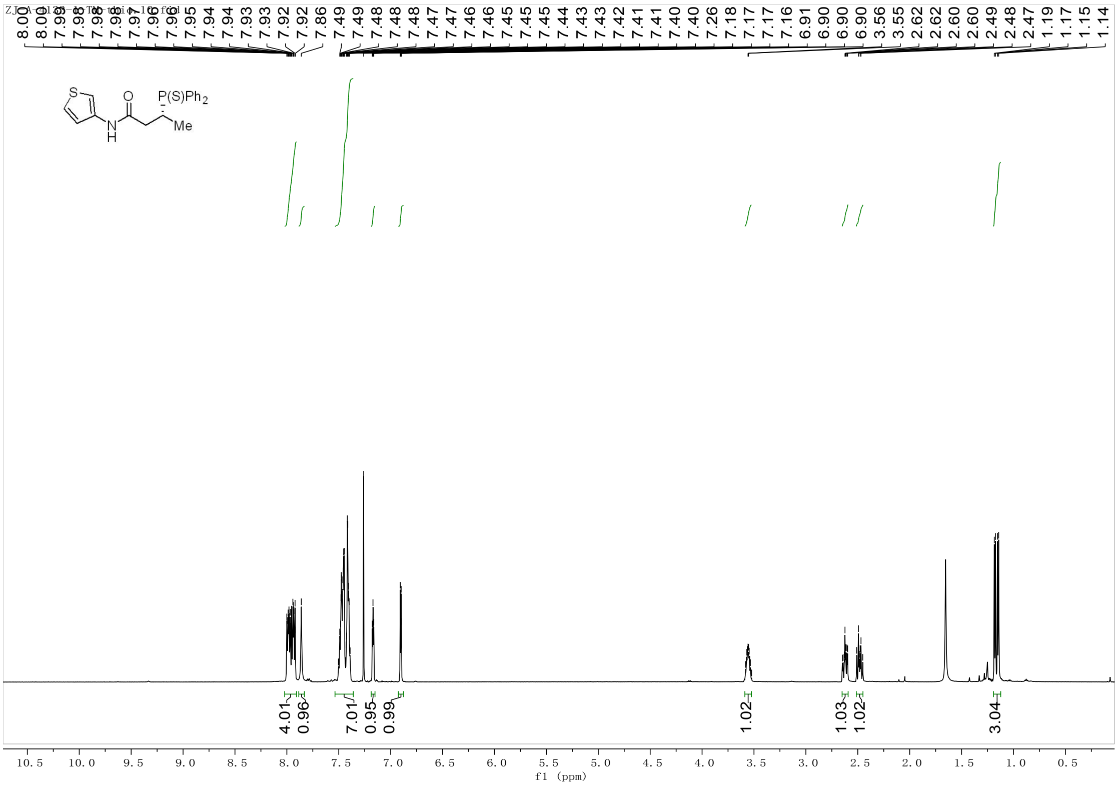

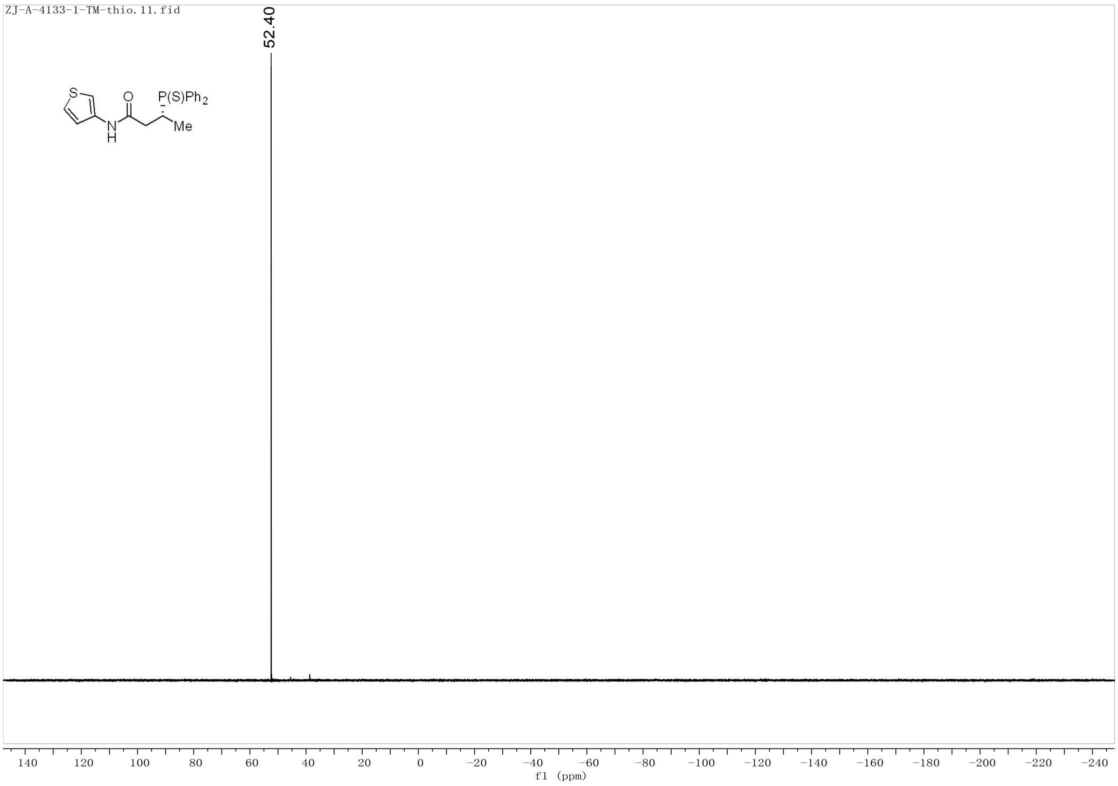
ZJ-A-4133-1-TM-thio.11.fid
52.40
P(S)Ph2
Me
S
O
N
H
f1 (ppm)

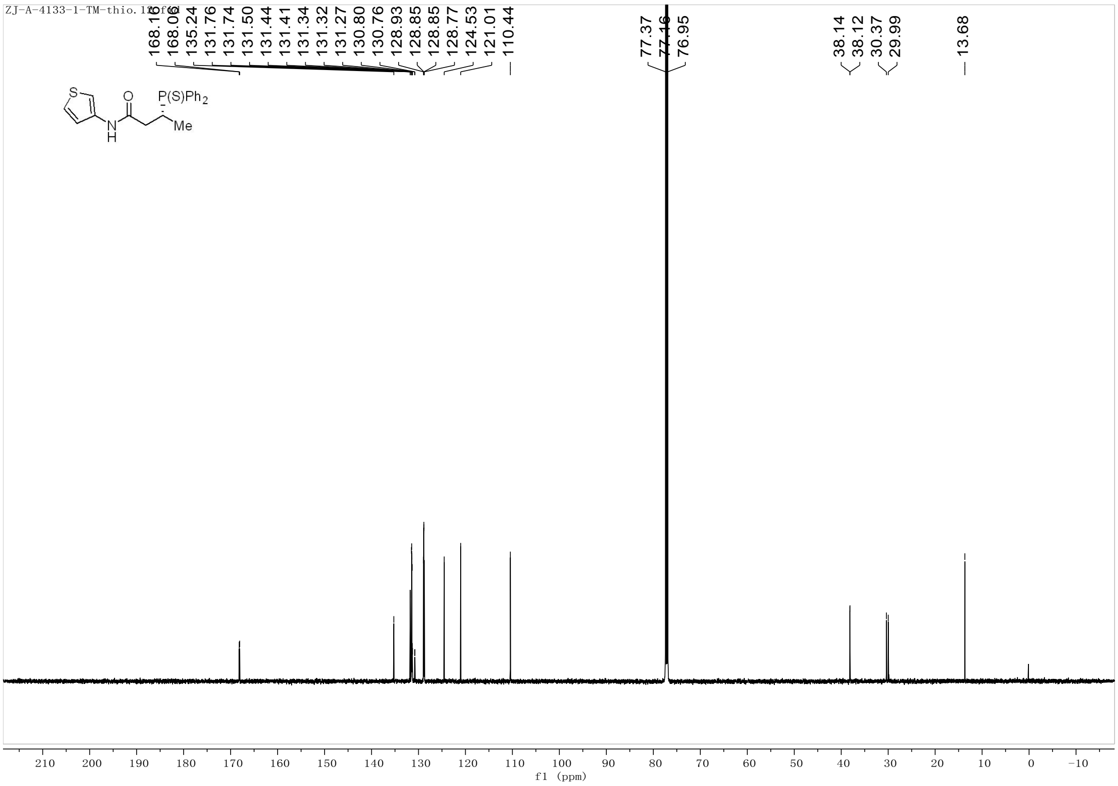
168.16
168.06
135.24
131.76
131.74
131.50
131.44
131.41
131.34
131.32
131.27
130.80
130.76
128.93
128.85
128.85
128.77
124.53
121.01
110.44
77.37
77.16
76.95
38.14
38.12
30.37
29.99
13.68
P(S)Ph2
Me
f1 (ppm)

(*R*)-N-benzhydryl-3-(diphenylphosphorothioyl)butanamide (3w)

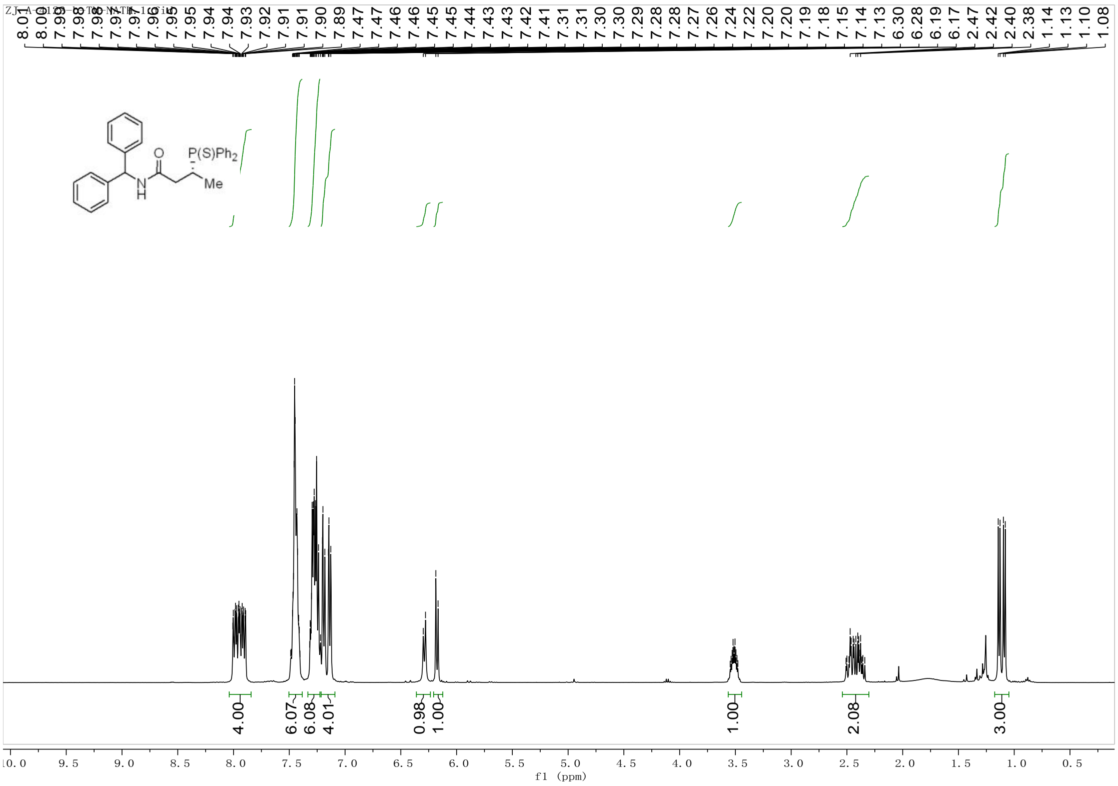

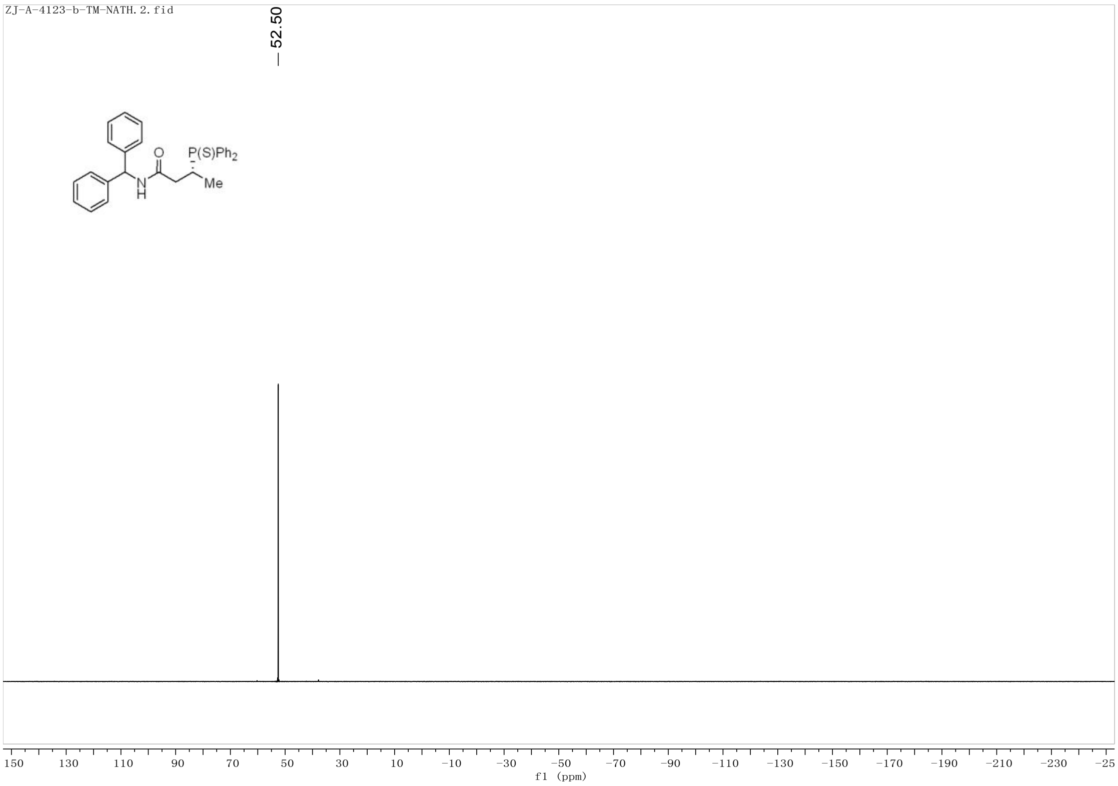

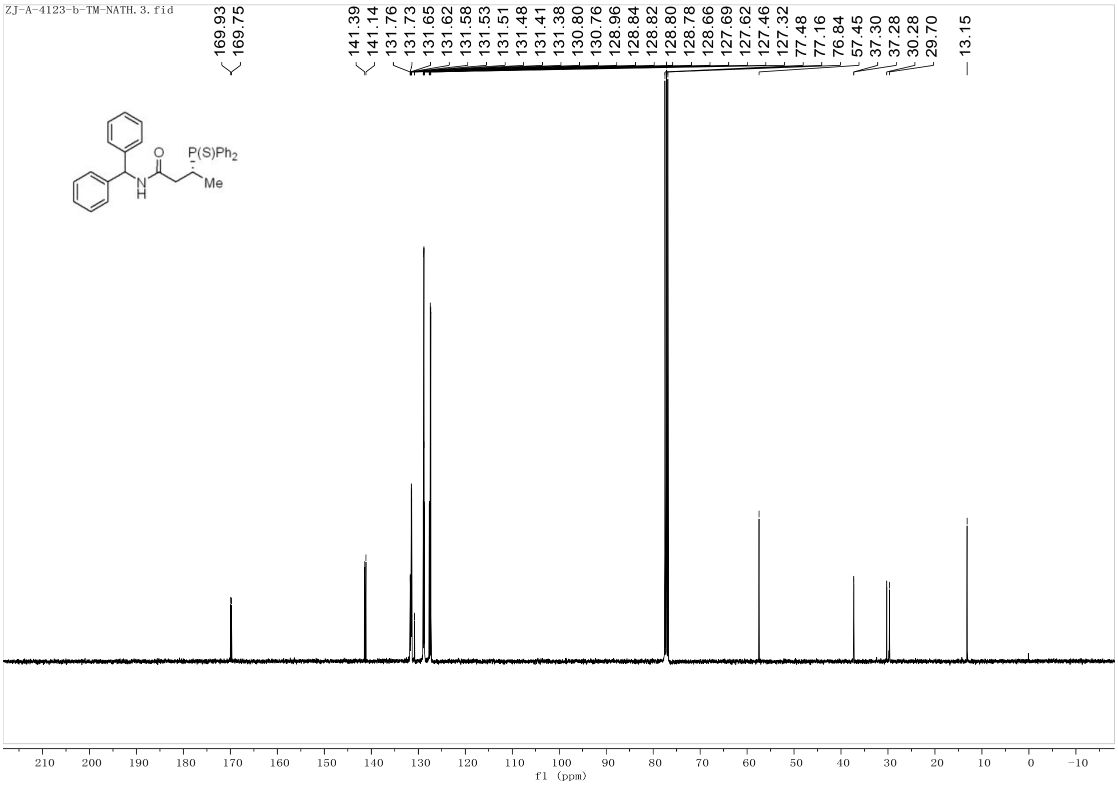

(*R*)-3-(diphenylphosphorothioyl)-N-(thiophen-3-ylmethyl)butanamide (3x)

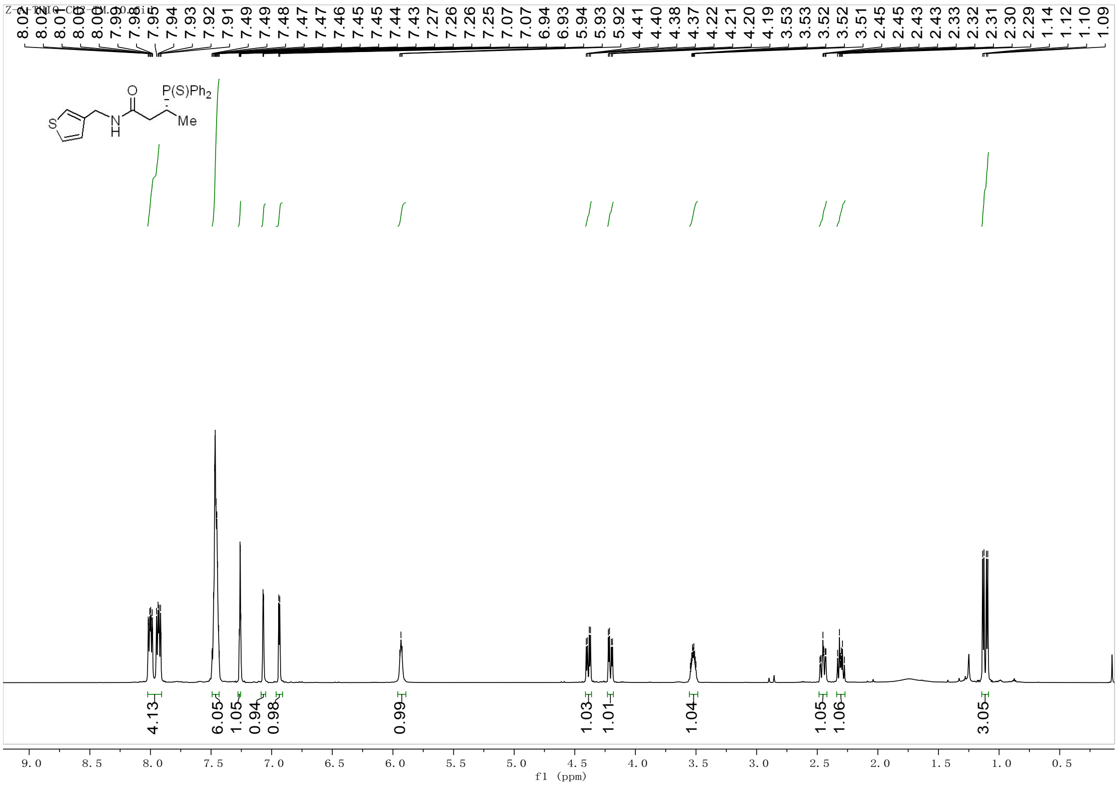

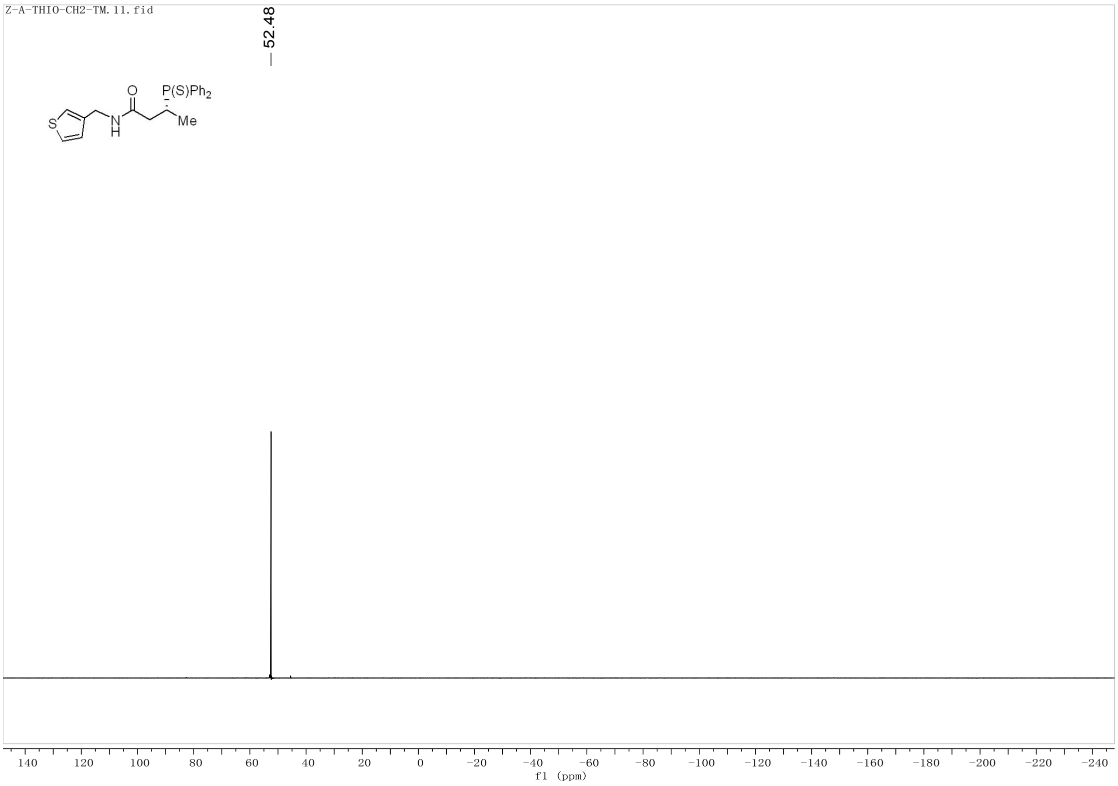
Z-A-THIO-CH2-TM.11.fid
52.48
P(S)Ph2
Me
S
N
H
O
f1 (ppm)

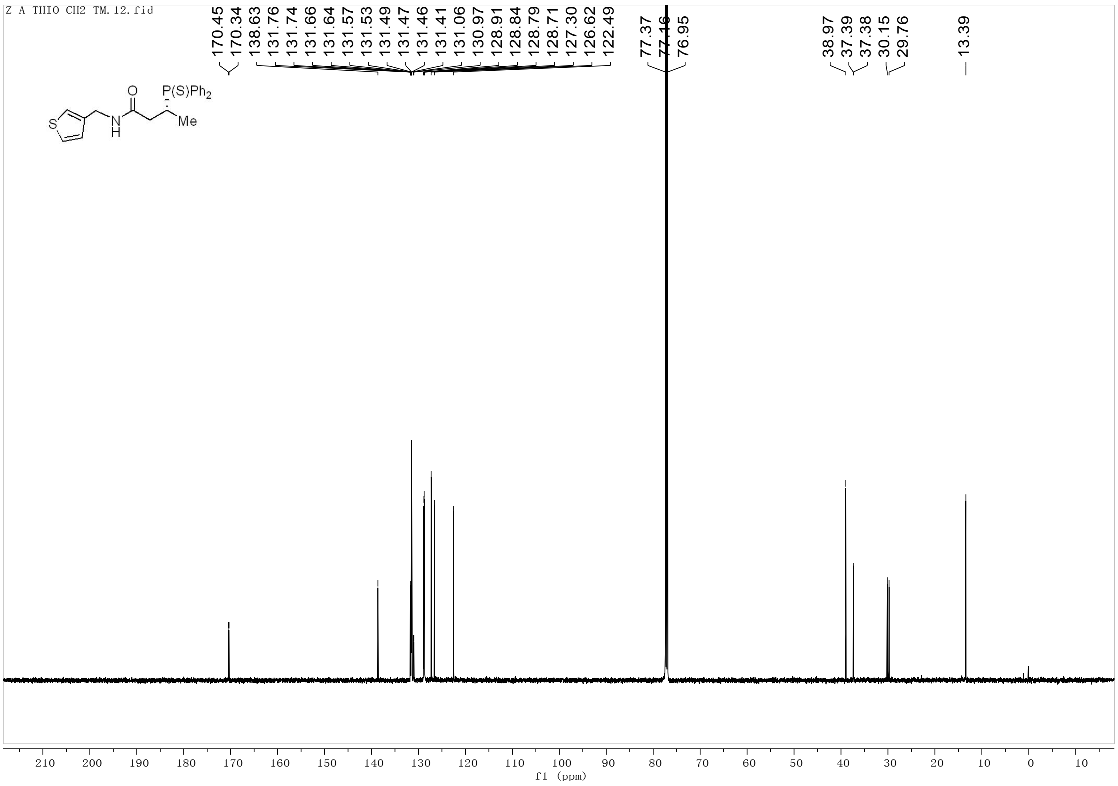
Z-A-THIO-CH2-TM.12.fid
170.45
170.34
138.63
131.76
131.74
131.66
131.64
131.57
131.53
131.49
131.47
131.46
131.41
131.06
130.97
128.91
128.84
128.79
128.71
127.30
126.62
122.49
77.37
77.16
76.95
38.97
37.39
37.38
30.15
29.76
13.39
P(S)Ph2
Me
S
N
H
O
f1 (ppm)

(*R*)-N-benzyl-3-(diphenylphosphorothioyl)butanamide (3y)

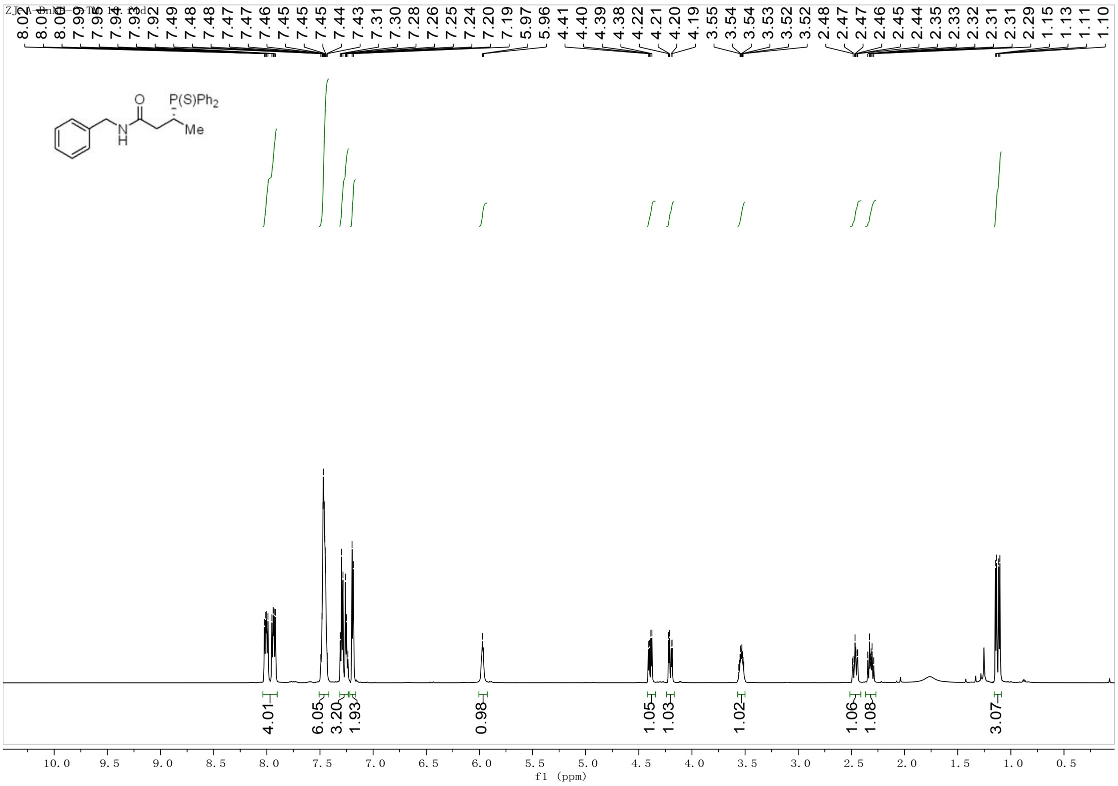

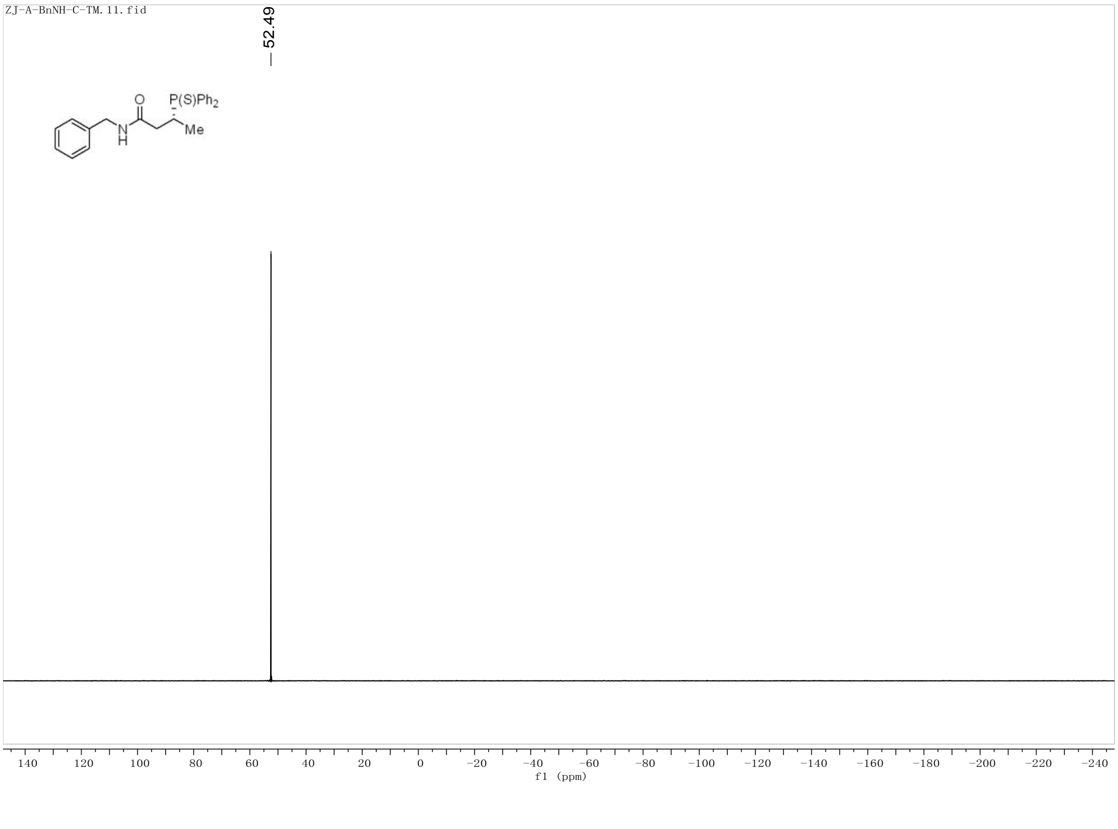

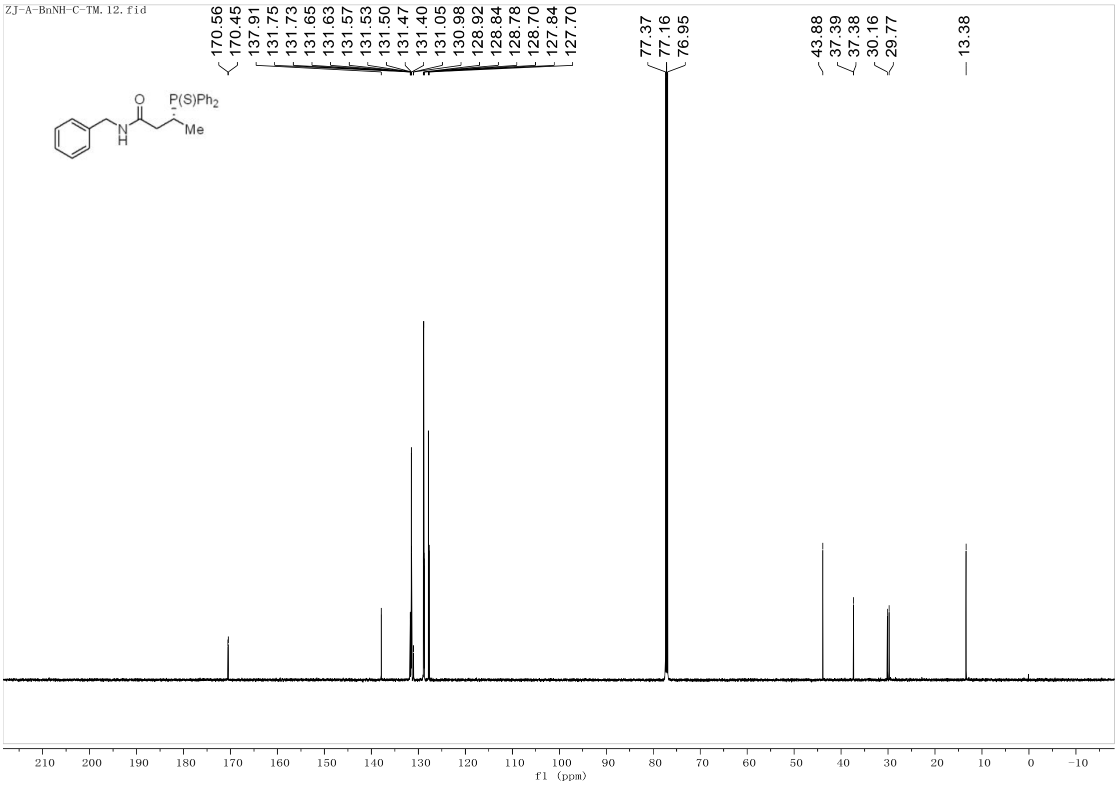

(*R*)-N-(2-bromobenzyl)-3-(diphenylphosphorothioyl)butanamide (3z)

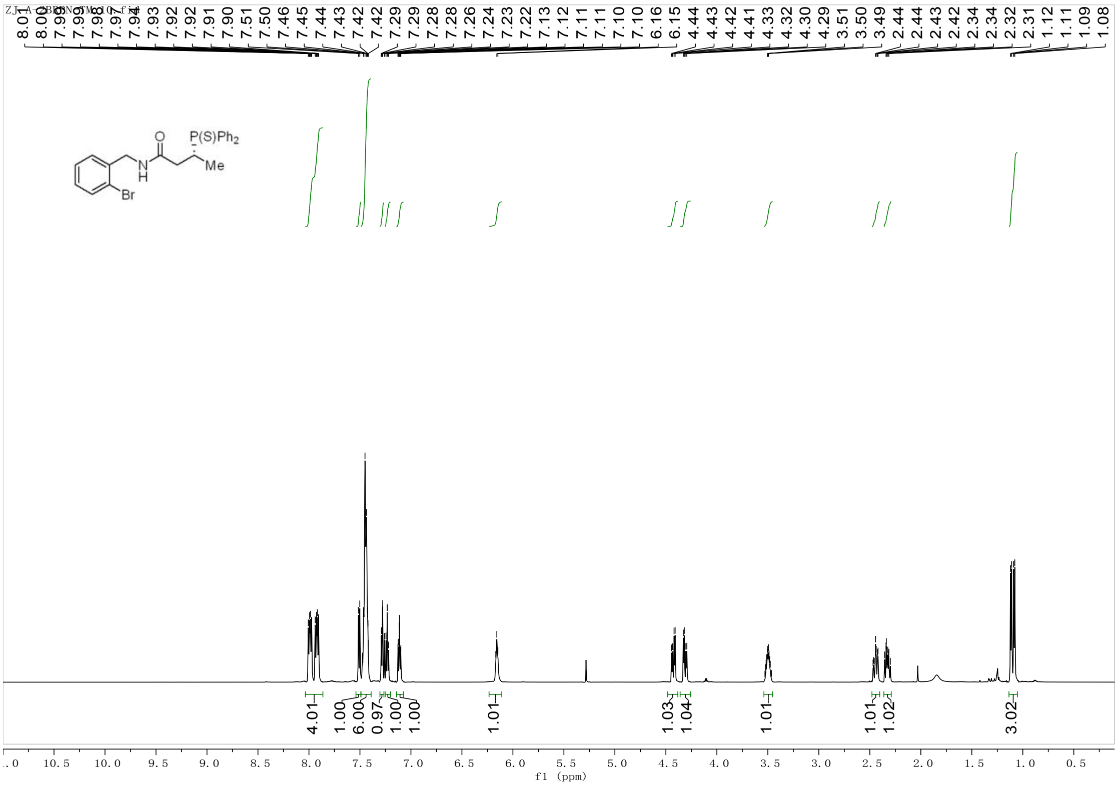

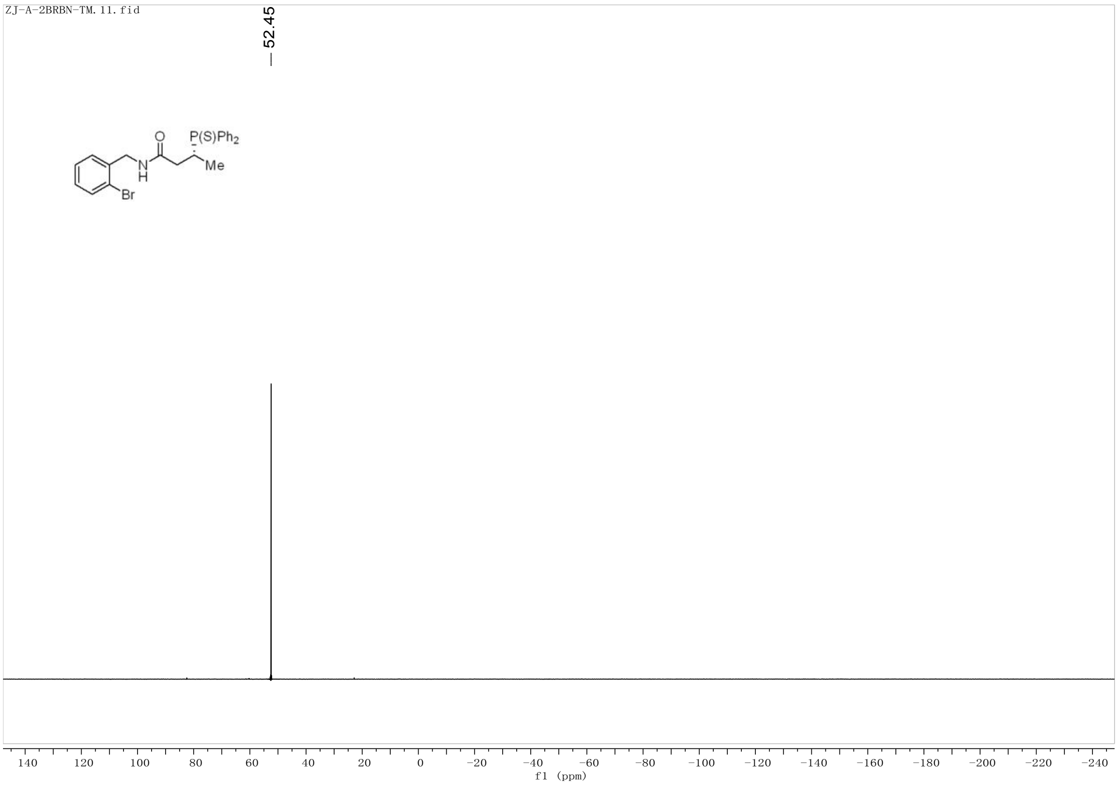
ZJ-A-2BRBN-TM.11.fid
52.45
O
P(S)Ph2
N
H
Me
Br
f1 (ppm)

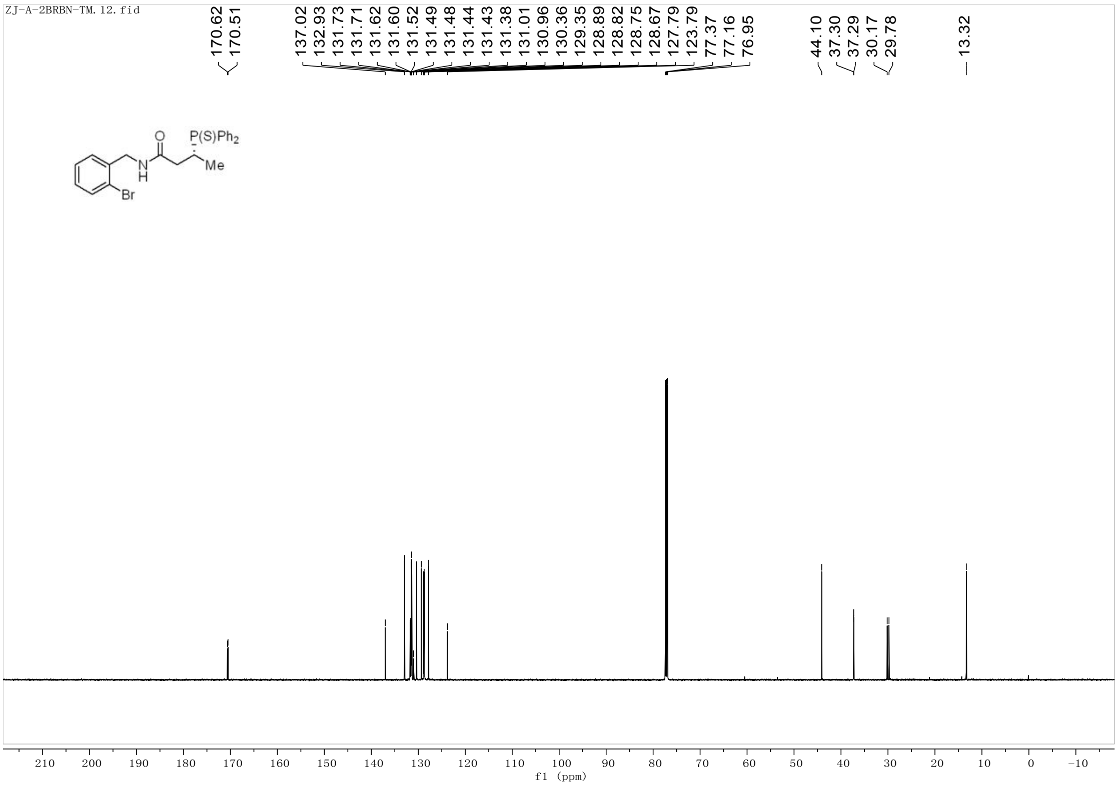
ZJ-A-2BRBN-TM.12.fid
170.62
170.51
137.02
132.93
131.73
131.71
131.62
131.60
131.52
131.49
131.48
131.44
131.43
131.38
131.01
130.96
130.36
129.35
128.89
128.82
128.75
128.67
127.79
123.79
77.37
77.16
76.95
44.10
37.30
37.29
30.17
29.78
13.32
O
P(S)Ph2
N
H
Me
Br
f1 (ppm)

methyl ((*R*)-3-(diphenylphosphorothioyl)butanoyl)-L-alaninate (3aa)

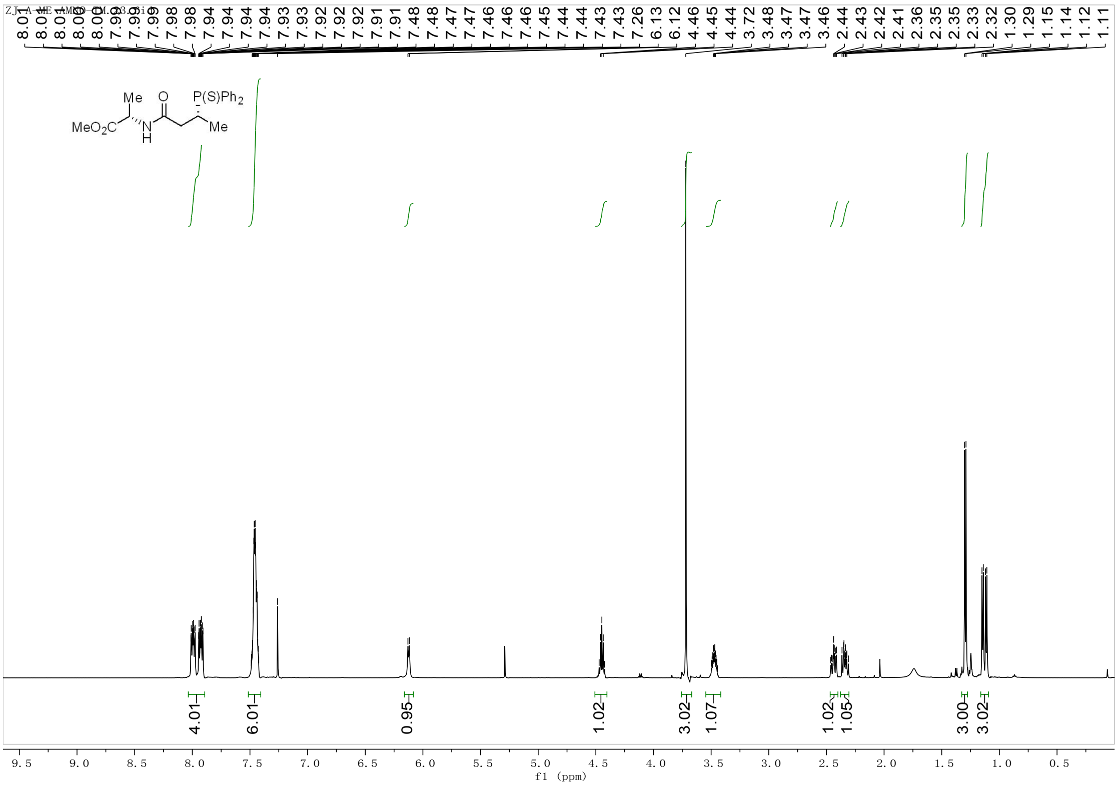

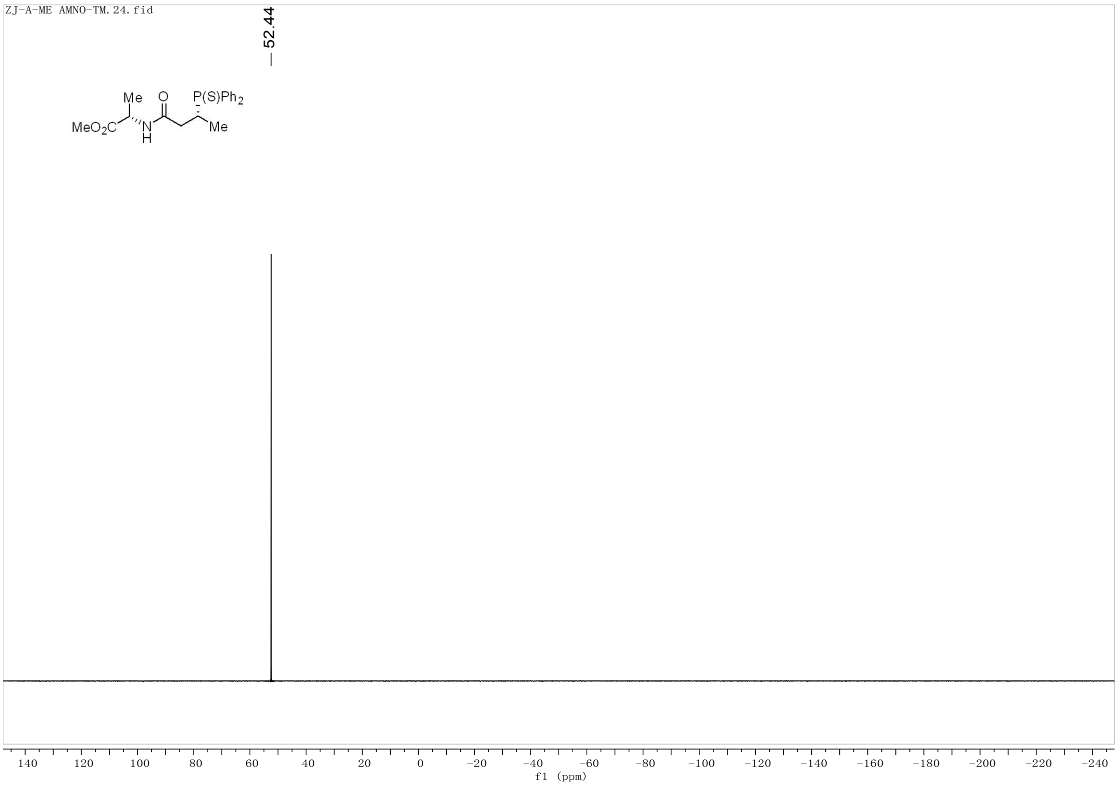

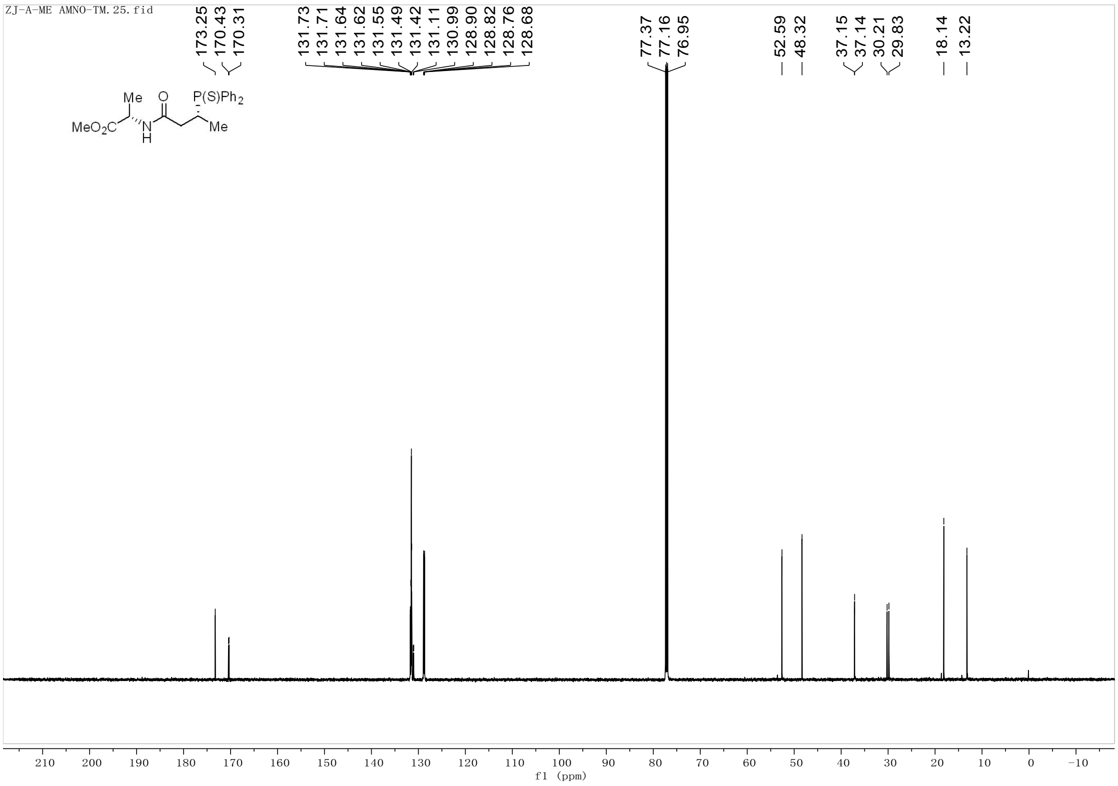

methyl ((*R*)-3-(diphenylphosphorothioyl)butanoyl)-D-tryptophanate (3ab)

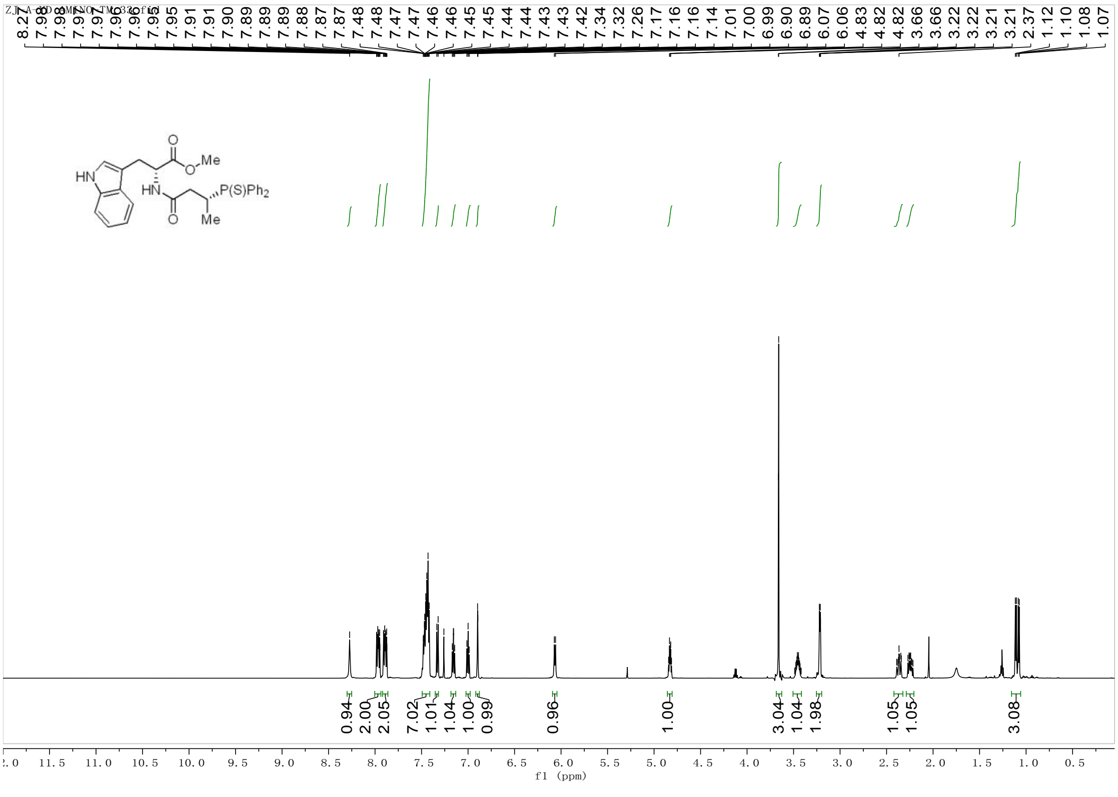

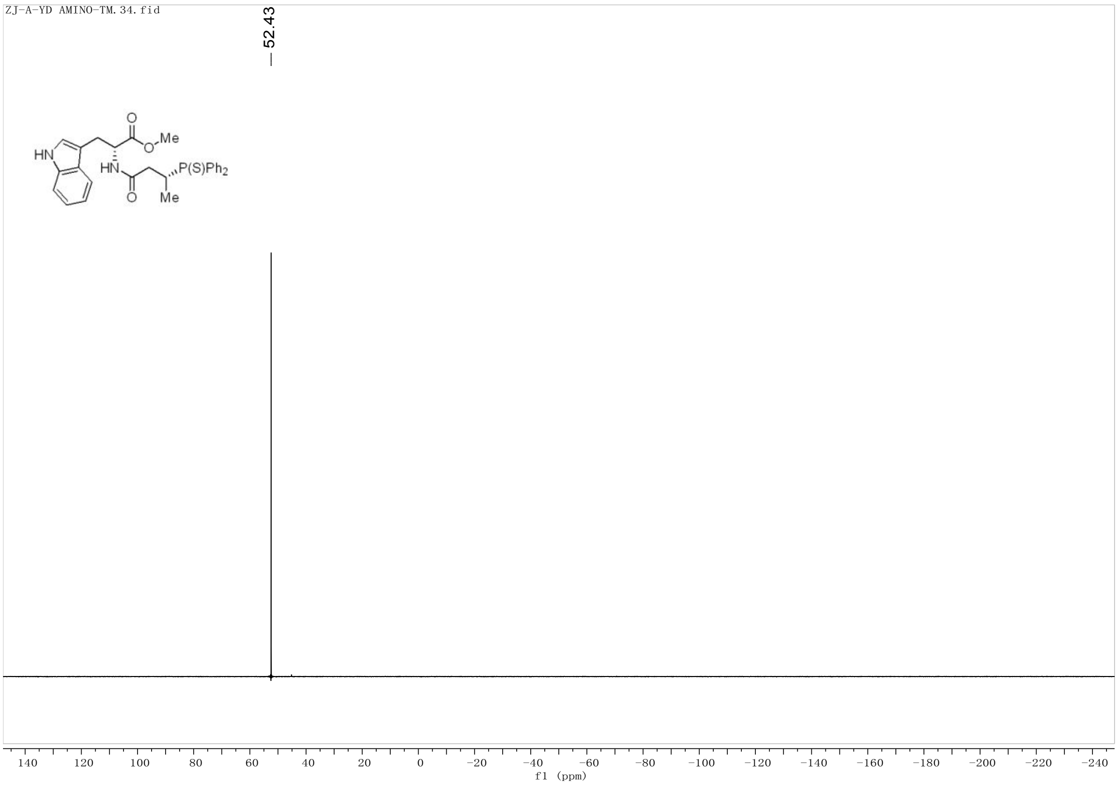
ZJ-A-YD AMINO-TM.34.fid
52.43
HN
O
Me
HN
P(S)Ph2
O
Me
140 120 100 80 60 40 20 0 −20 −40 −60 −80 −100 −120 −140 −160 −180 −200 −220 −240
f1 (ppm)

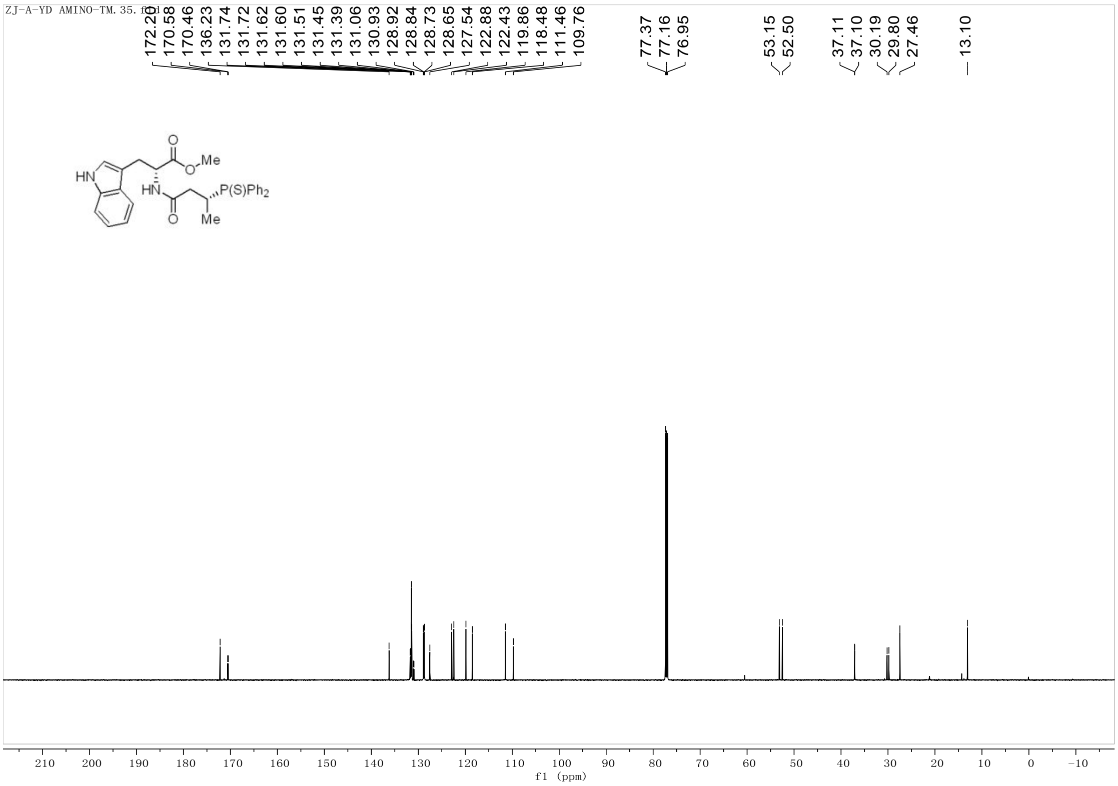
ZJ-A-YD AMINO-TM.35.fid
172.20
170.58
170.46
136.23
131.74
131.72
131.62
131.60
131.51
131.45
131.39
131.06
130.93
128.92
128.84
128.73
128.65
127.54
122.88
122.43
119.86
118.48
111.46
109.76
77.37
77.16
76.95
53.15
52.50
37.11
37.10
30.19
29.80
27.46
13.10
HN
O
Me
HN
P(S)Ph2
O
Me
210 200 190 180 170 160 150 140 130 120 110 100 90 80 70 60 50 40 30 20 10 0 −10
f1 (ppm)

(*R*)-3-(diphenylphosphorothioyl)-N-methyl-N-phenylbutanamide (3ac)

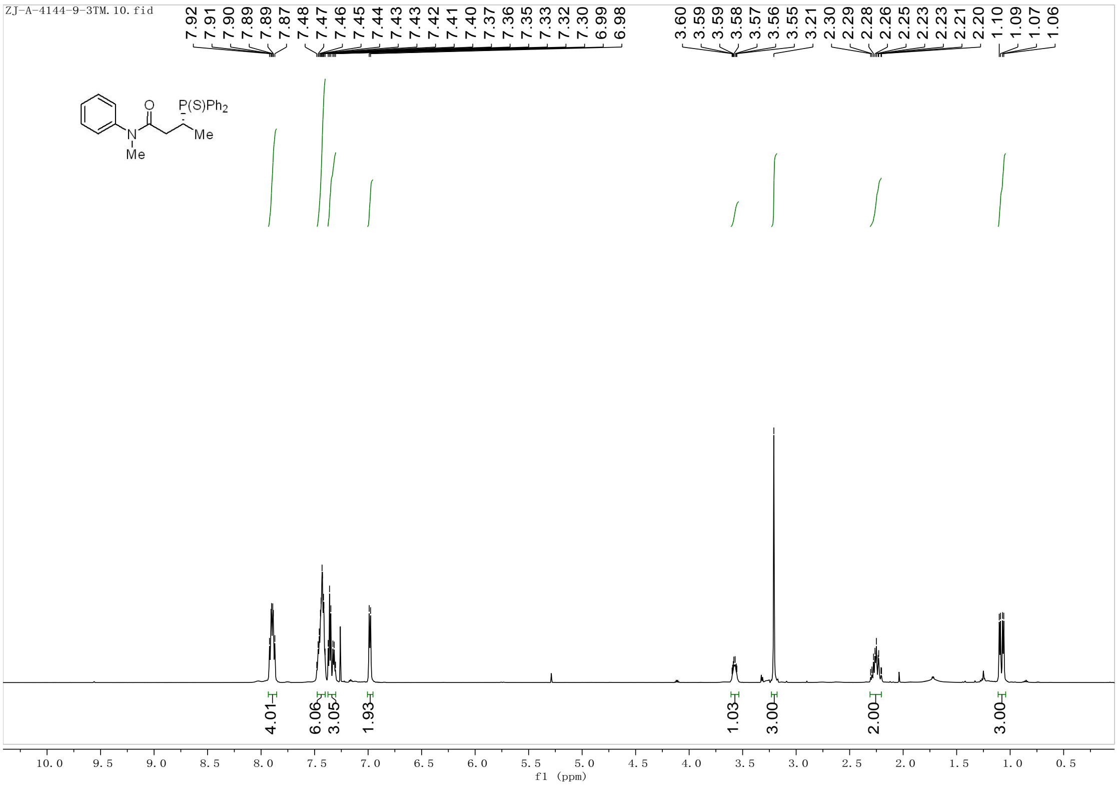

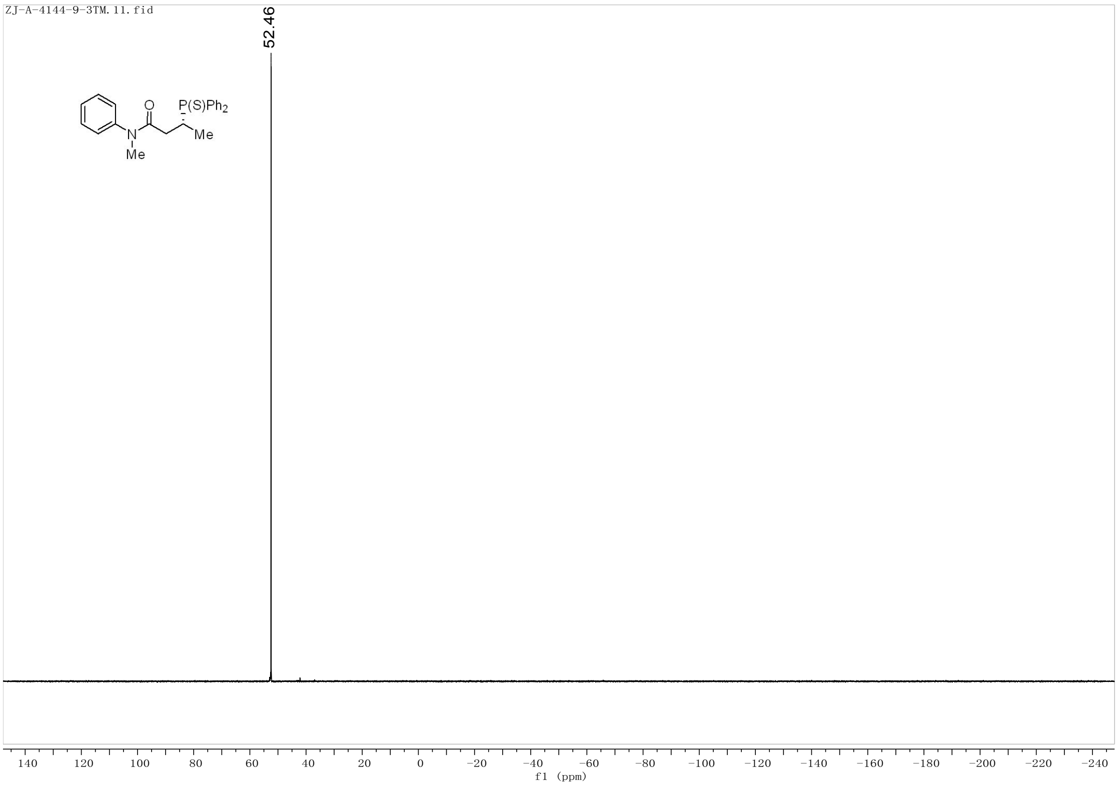

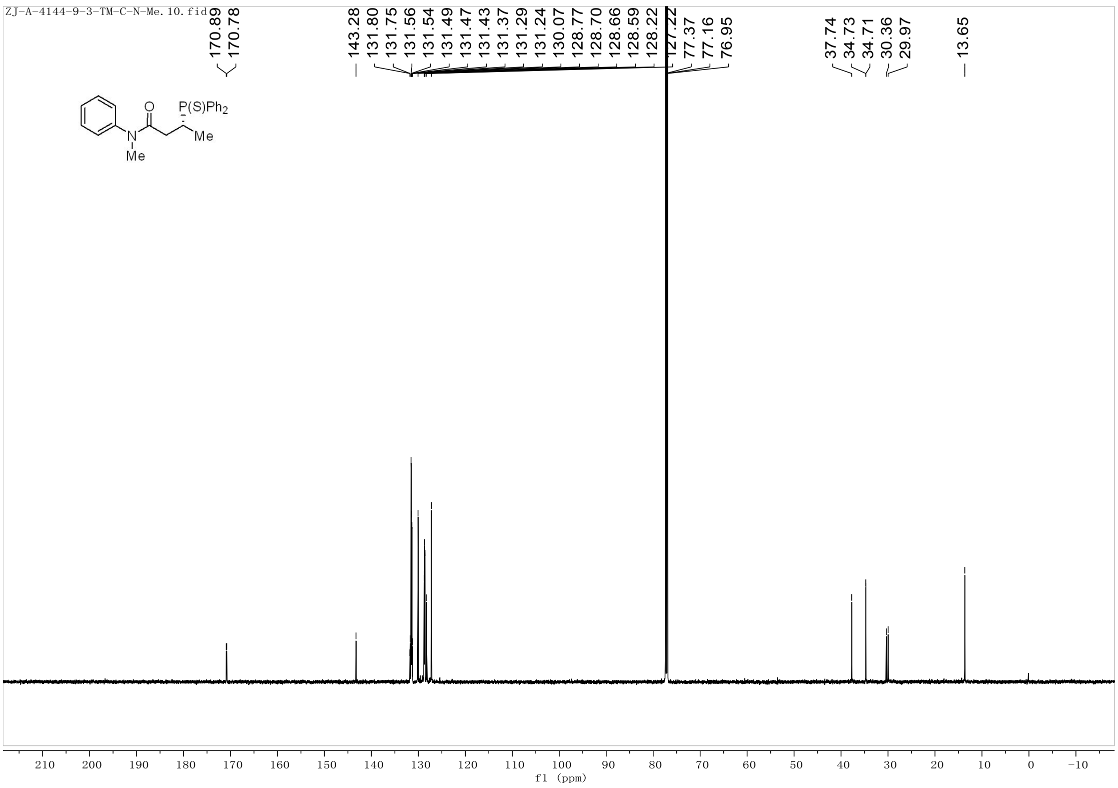

(*R*)-3-(diphenylphosphorothioyl)-N-phenylpentanamide (3ad)

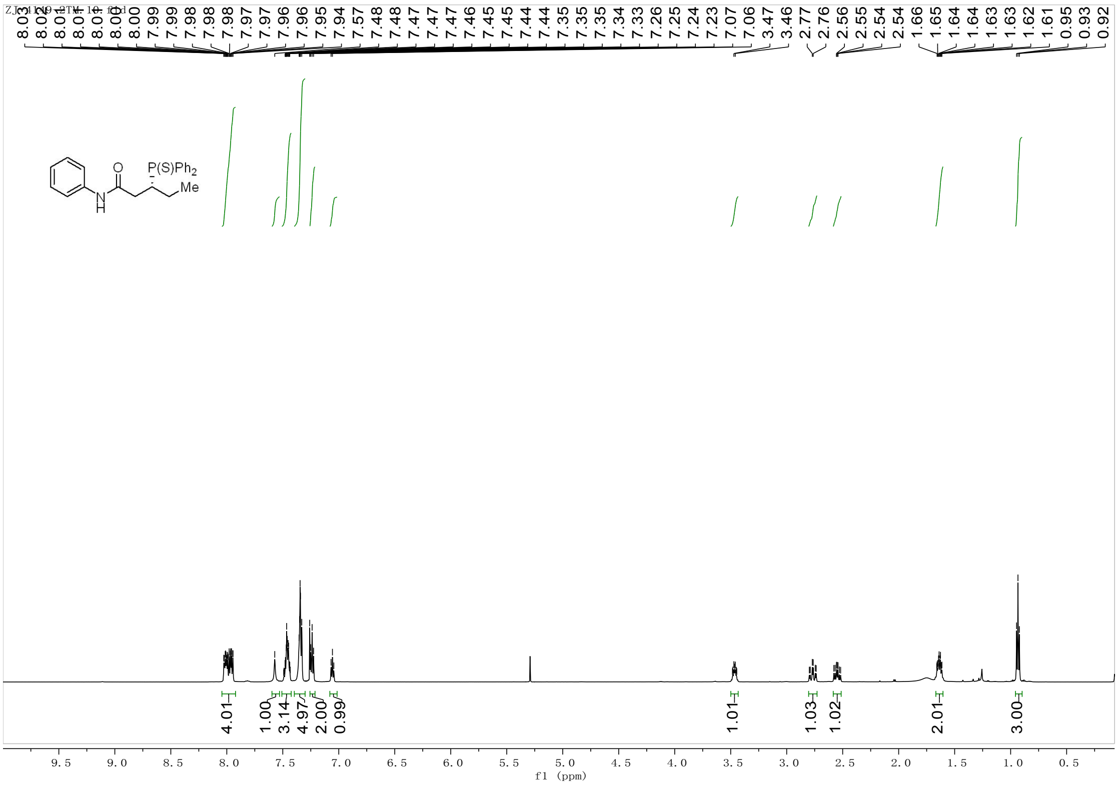

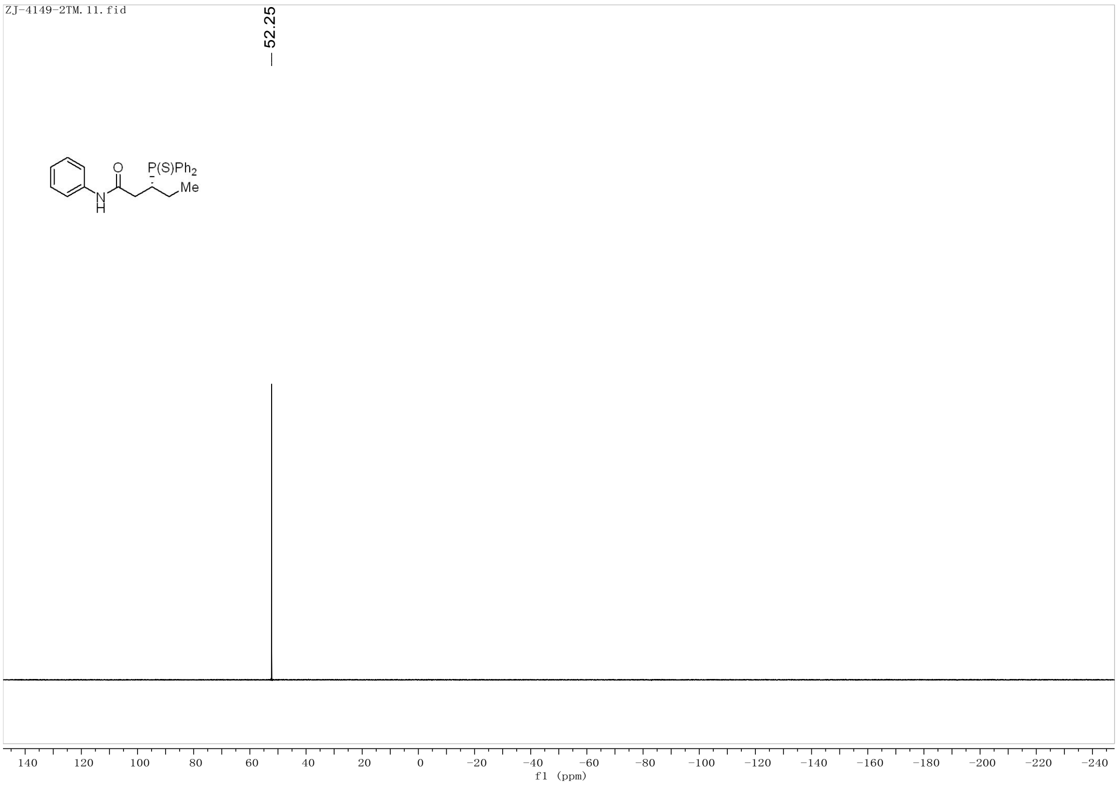
ZJ-4149-2TM.11.fid
52.25
P(S)Ph2
O
N
H
Me
f1 (ppm)

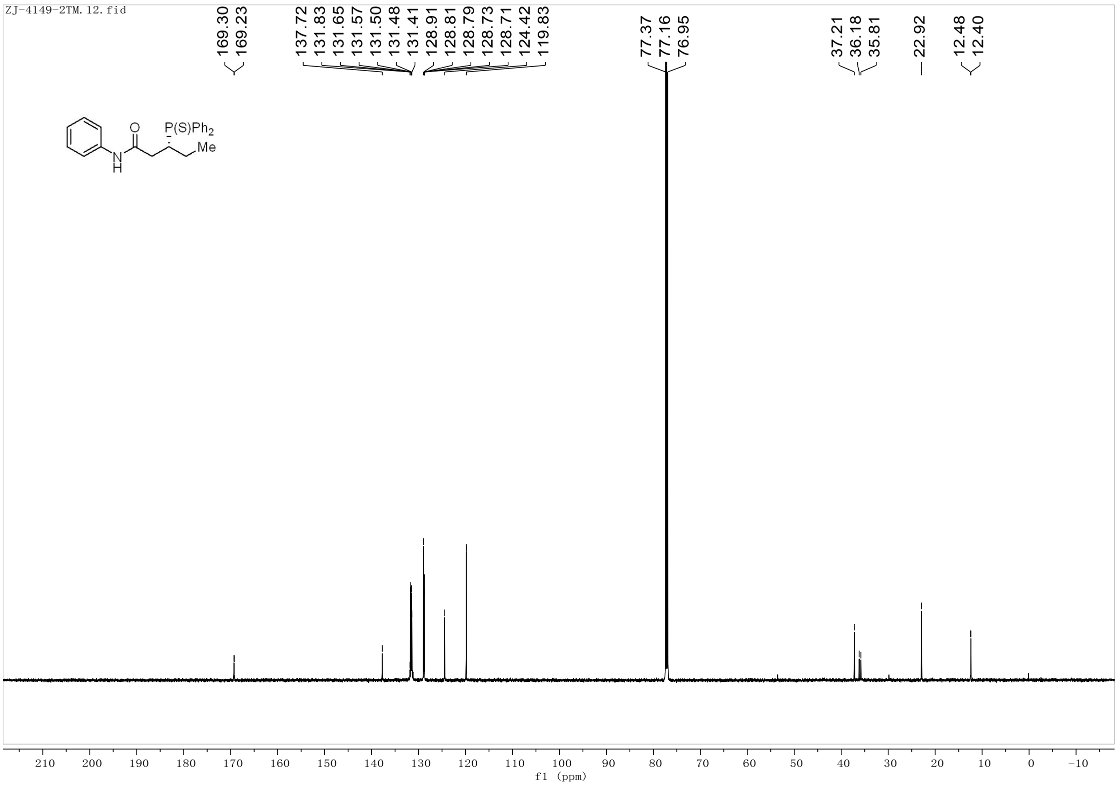
ZJ-4149-2TM.12.fid
169.30
169.23
137.72
131.83
131.65
131.57
131.50
131.48
131.41
128.91
128.81
128.79
128.73
128.71
124.42
119.83
77.37
77.16
76.95
37.21
36.18
35.81
22.92
12.48
12.40
P(S)Ph2
O
N
H
Me
f1 (ppm)

(*R*)-3-(di-*p*-tolylphosphorothioyl)-N-phenylbutanamide (3ae)

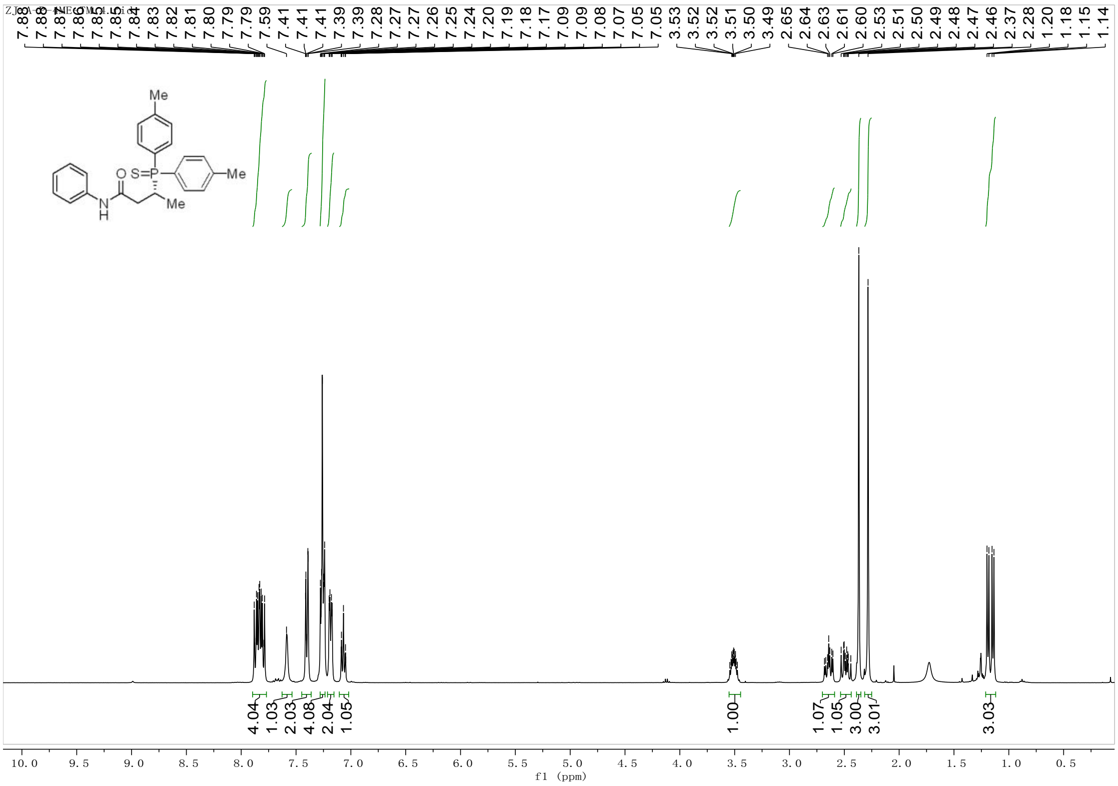

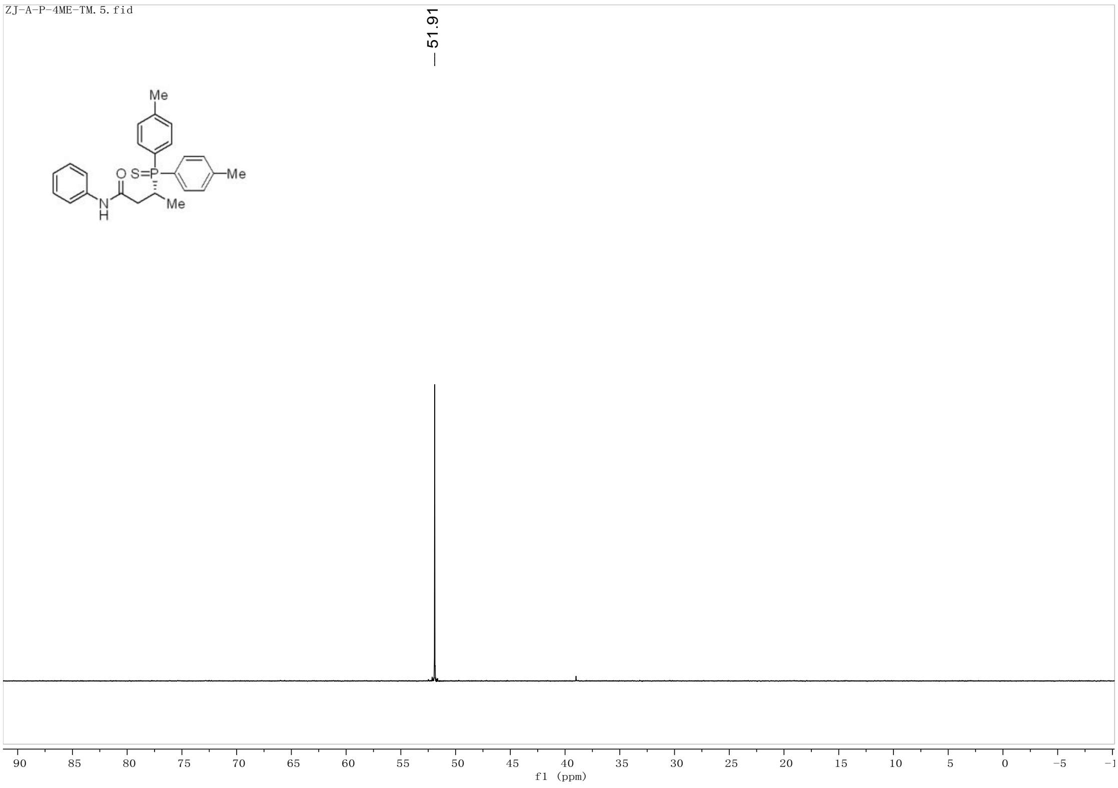

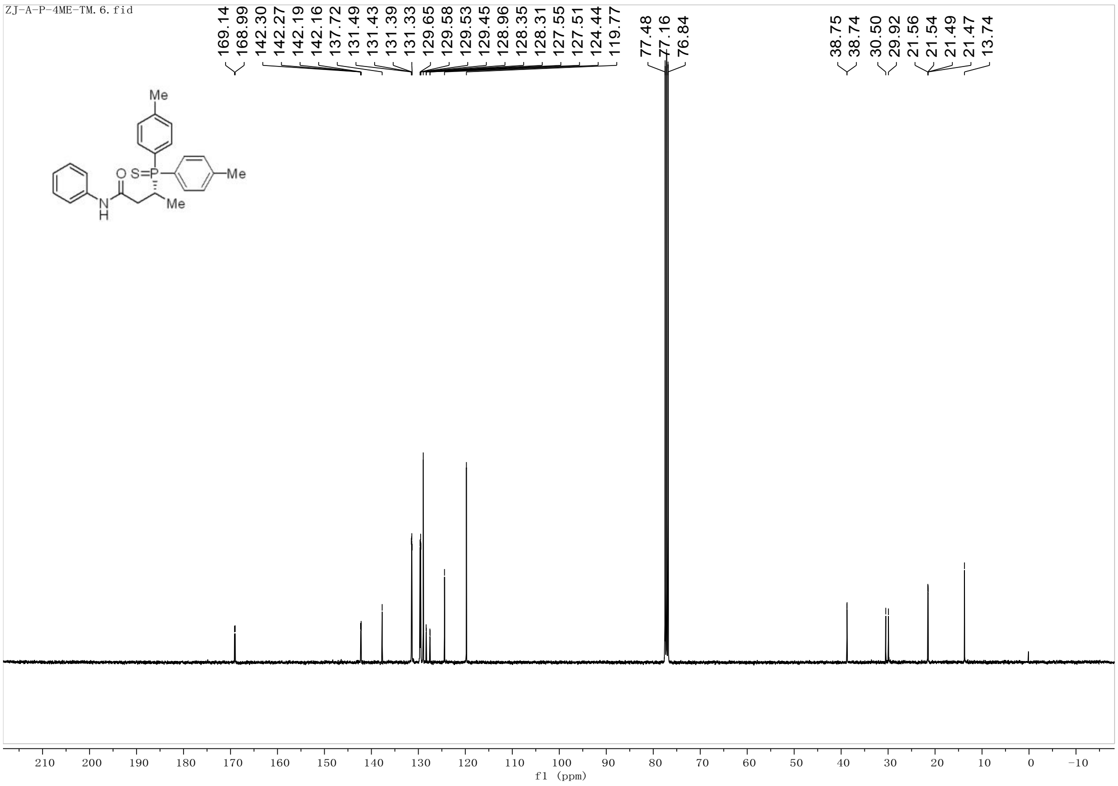

(*R*)-3-(bis(3,5-dimethylphenyl)phosphorothioyl)-N-phenylbutanamide (3af)

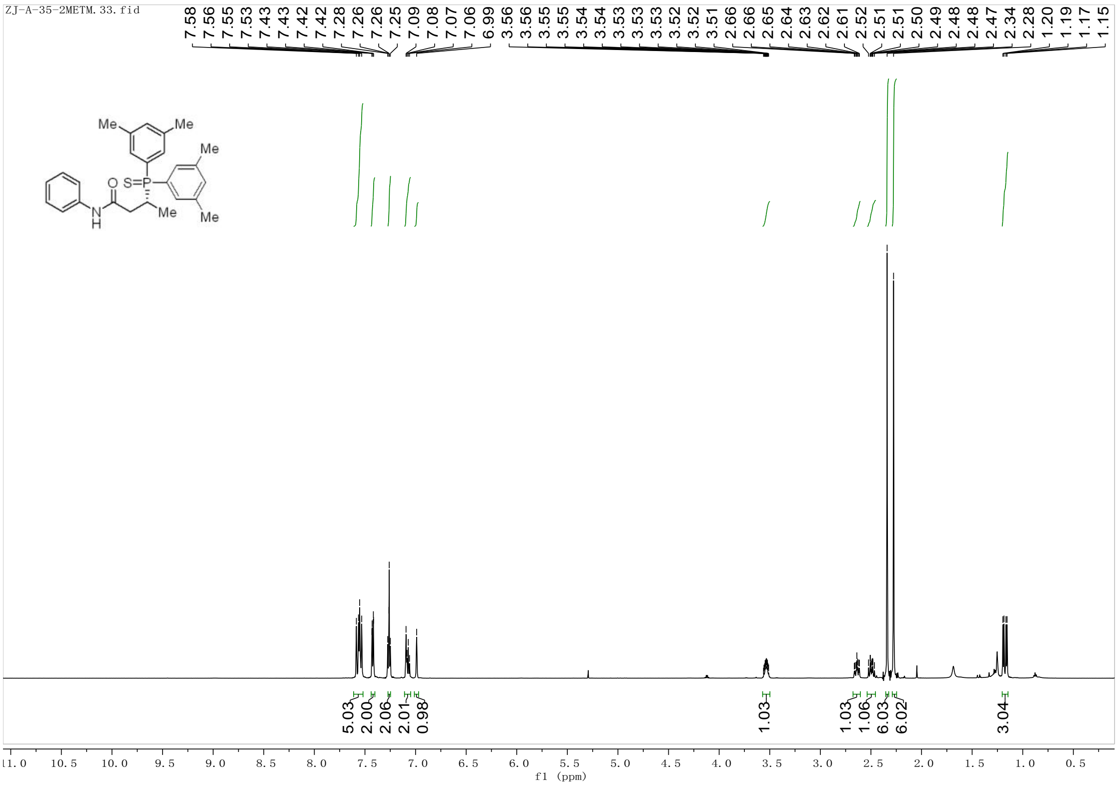

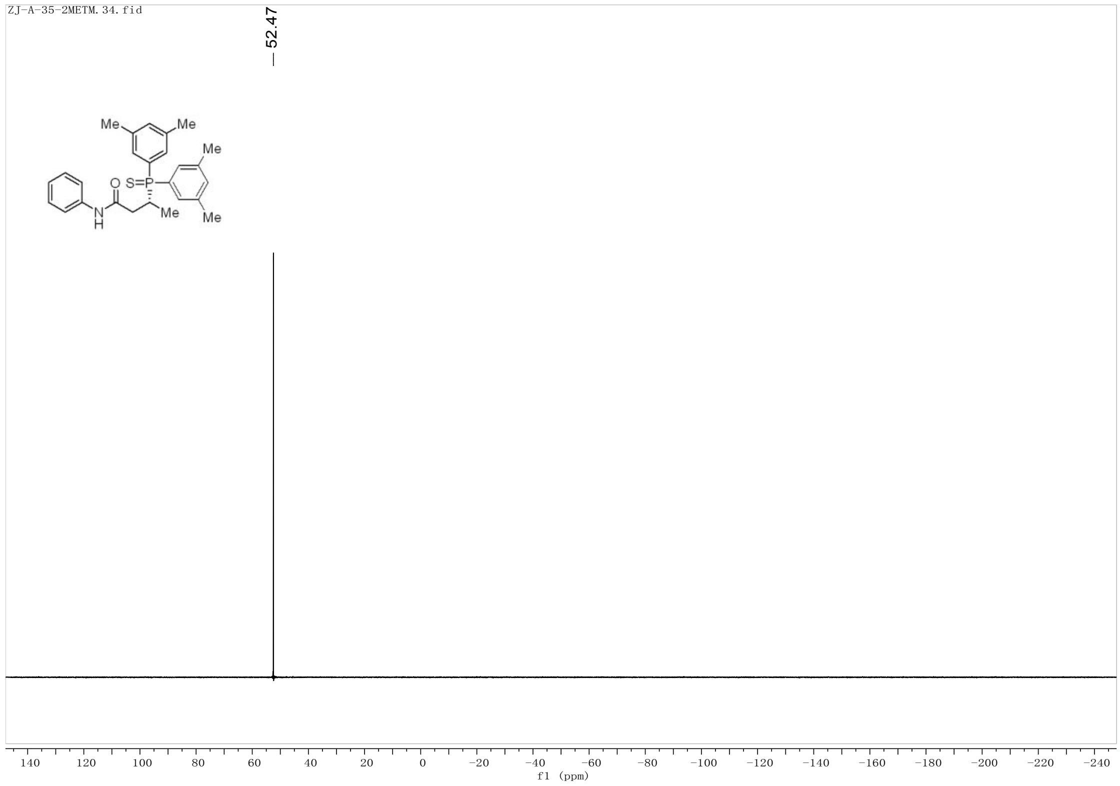
ZJ-A-35-2METM.34.fid
52.47
Me
Me
Me
S=P
Me
O
N
H
Me
f1 (ppm)

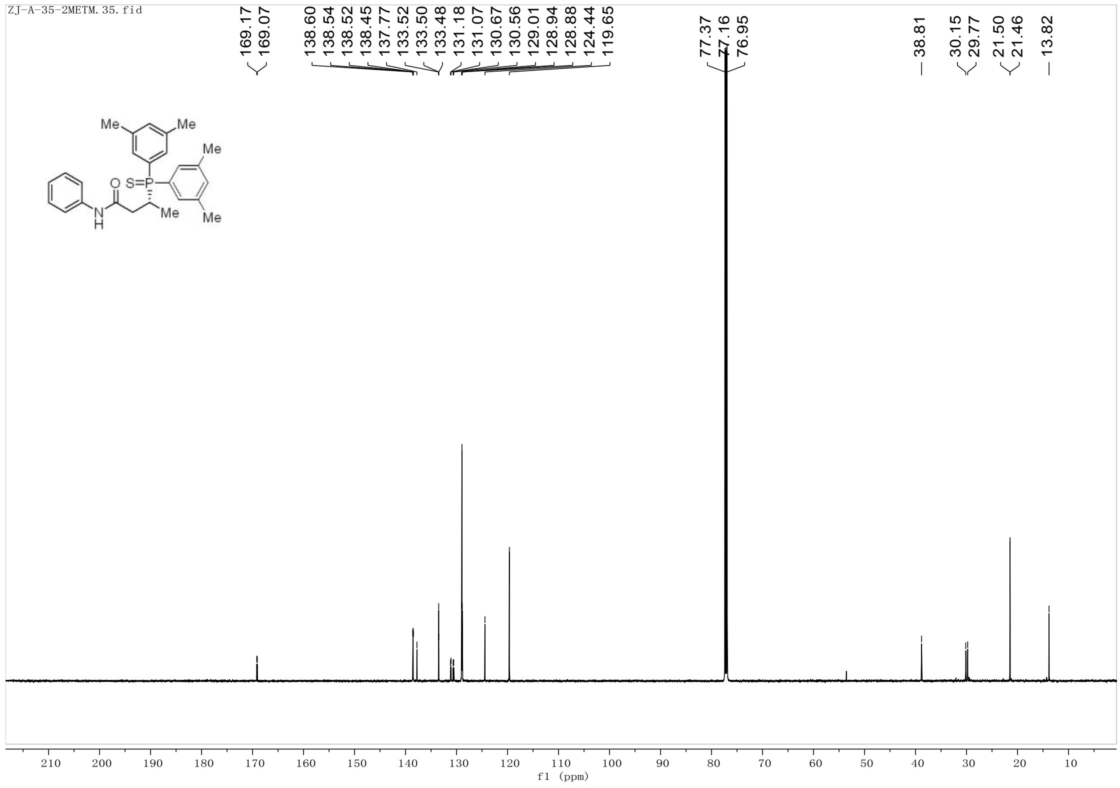
ZJ-A-35-2METM.35.fid
169.17
169.07
138.60
138.54
138.52
138.45
137.77
133.52
133.50
133.48
131.18
131.07
130.67
130.56
129.01
128.94
128.88
124.44
119.65
77.37
77.16
76.95
38.81
30.15
29.77
21.50
21.46
13.82
f1 (ppm)

(*R*)-3-(di-m-tolylphosphorothioyl)-N-phenylbutanamide (3ag)

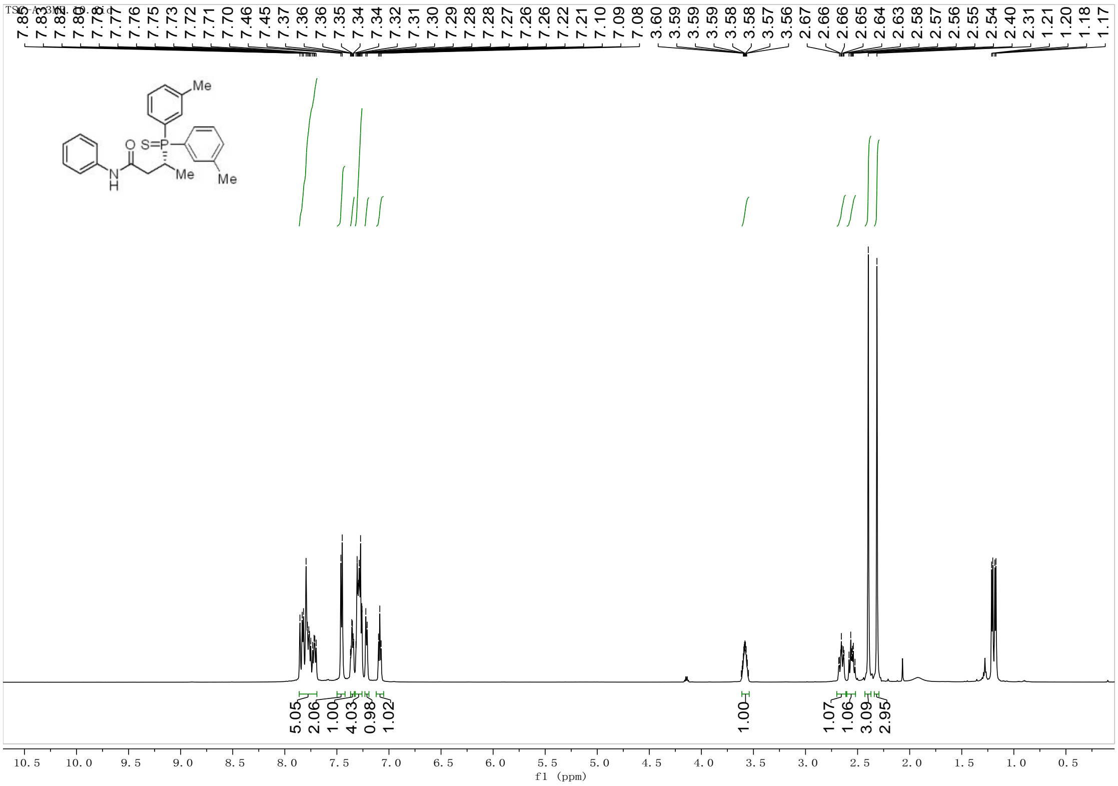

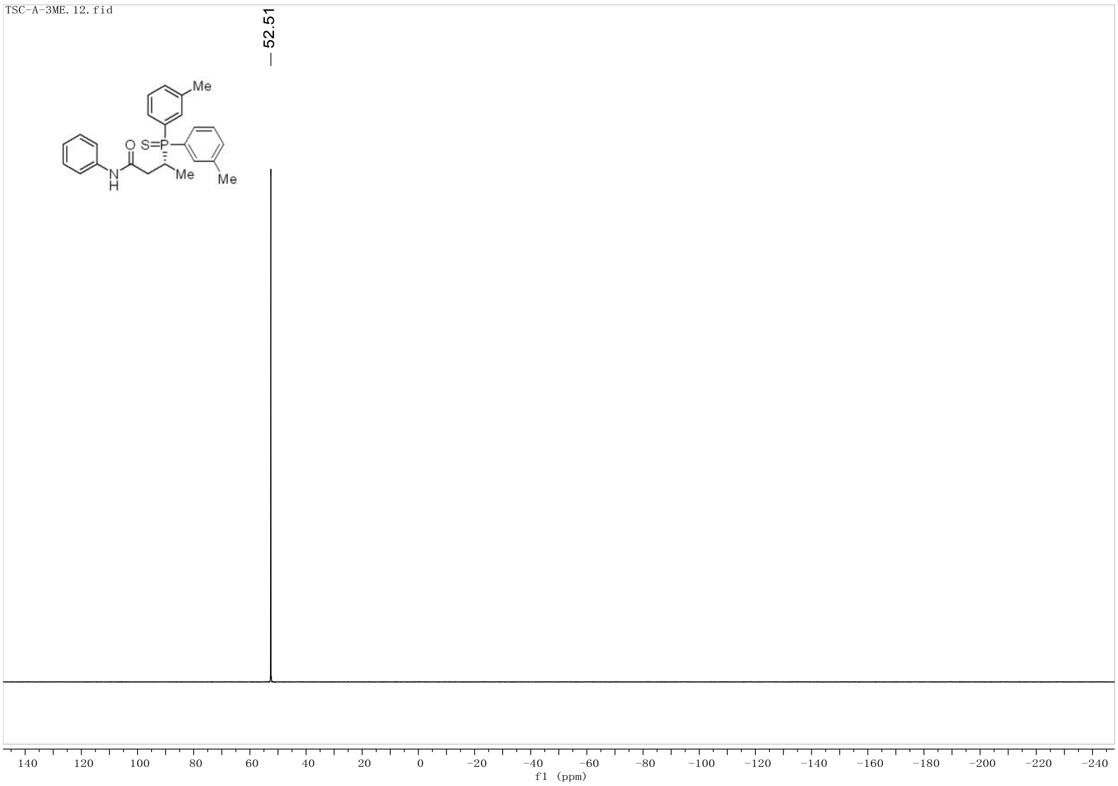

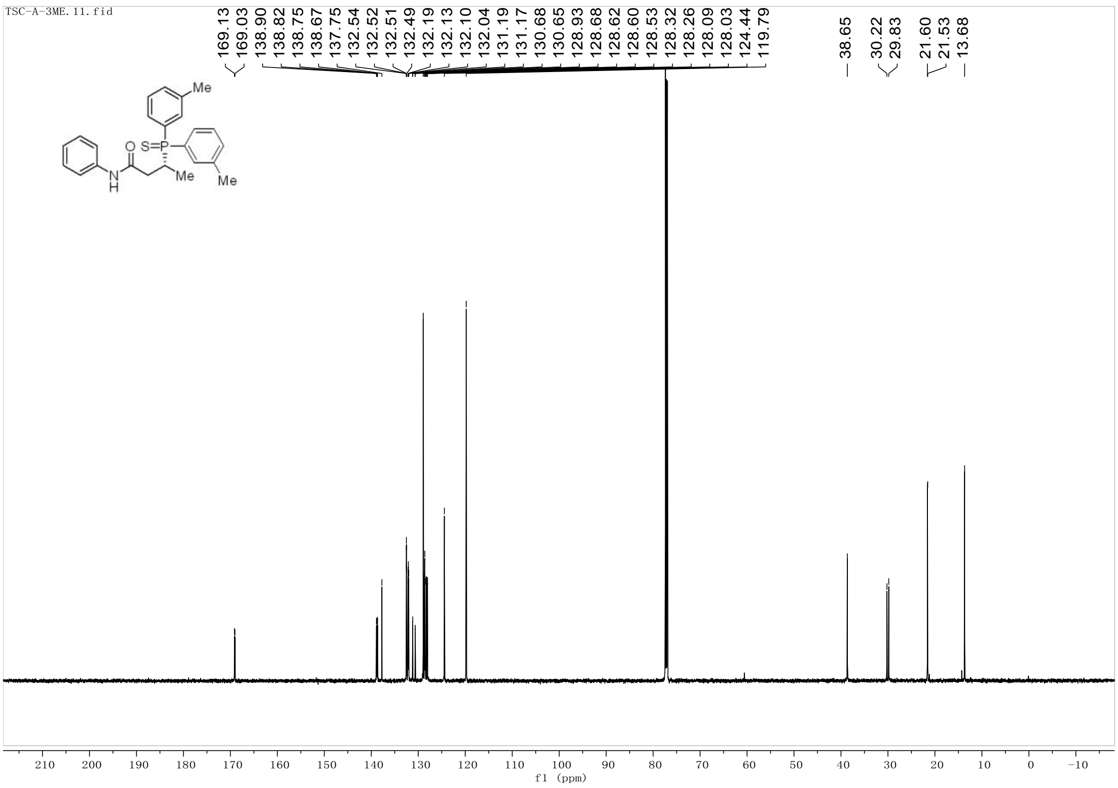

(*R*)-3-(bis(4-(tert-butyl)phenyl)phosphorothioyl)-N-phenylbutanamide (3ah)

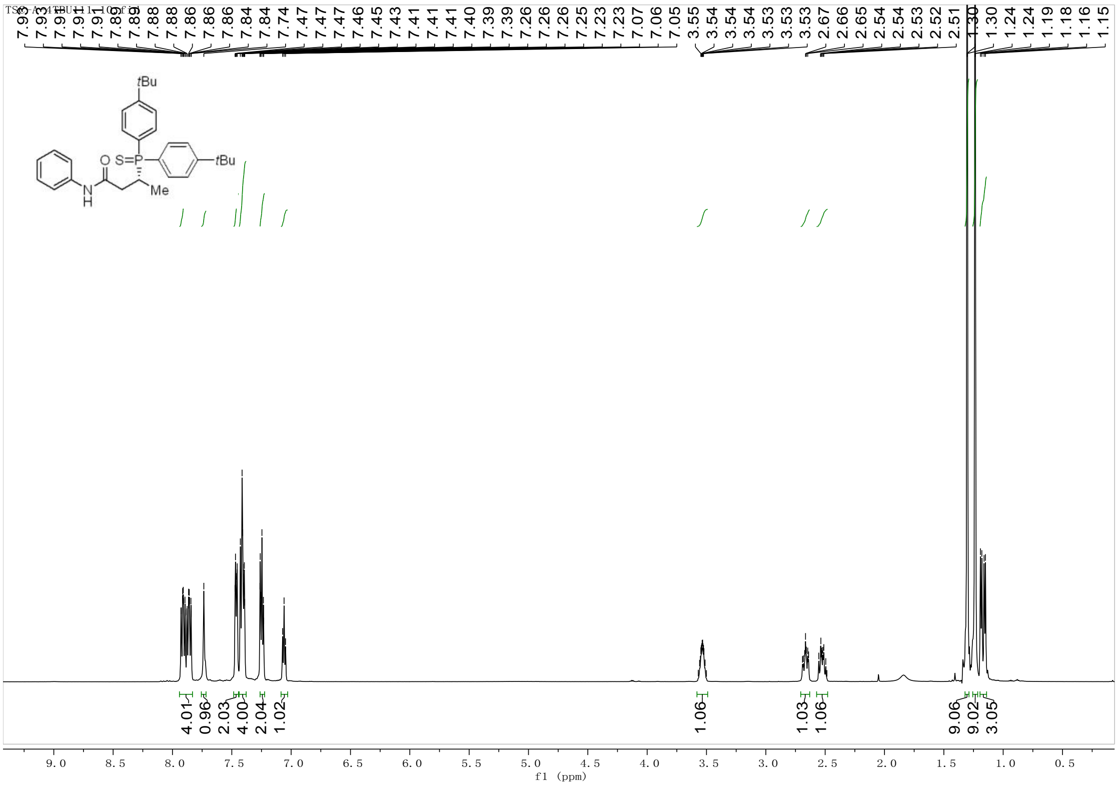

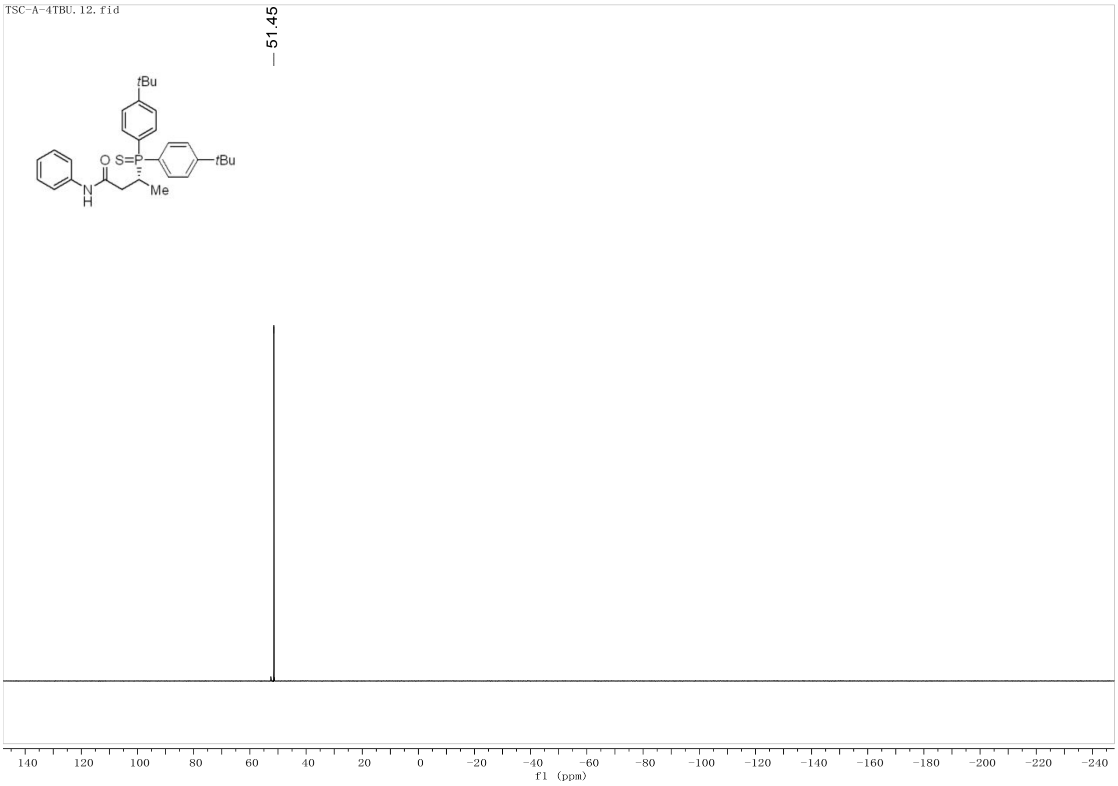
TSC-A-4TBU.12.fid
51.45
tBu
S=P
tBu
O
N
H
Me
f1 (ppm)

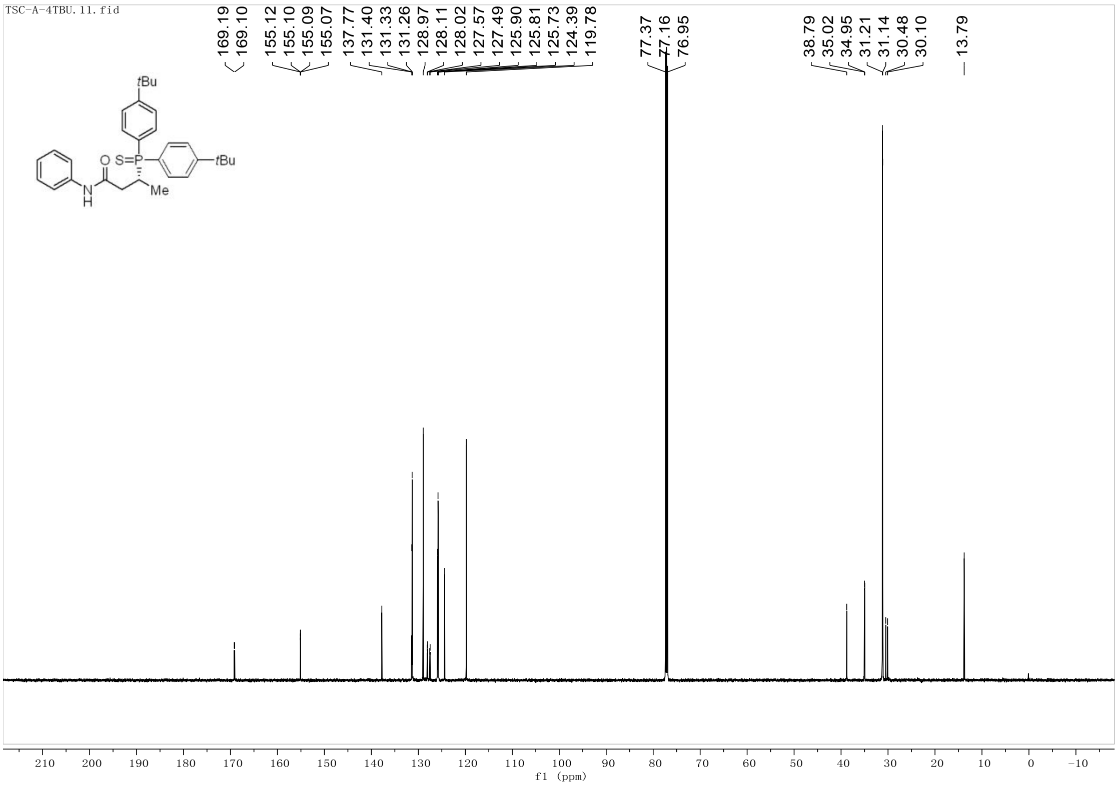
TSC-A-4TBU.11.fid
169.19
169.10
155.12
155.10
155.09
155.07
137.77
131.40
131.33
131.26
128.97
128.11
128.02
127.57
127.49
125.90
125.81
125.73
124.39
119.78
77.37
77.16
76.95
38.79
35.02
34.95
31.21
31.14
30.48
30.10
13.79
tBu
S=P
tBu
O
N
H
Me
f1 (ppm)

(*R*)-3-(bis(4-chlorophenyl)phosphorothioyl)-N-phenylbutanamide (3ai)

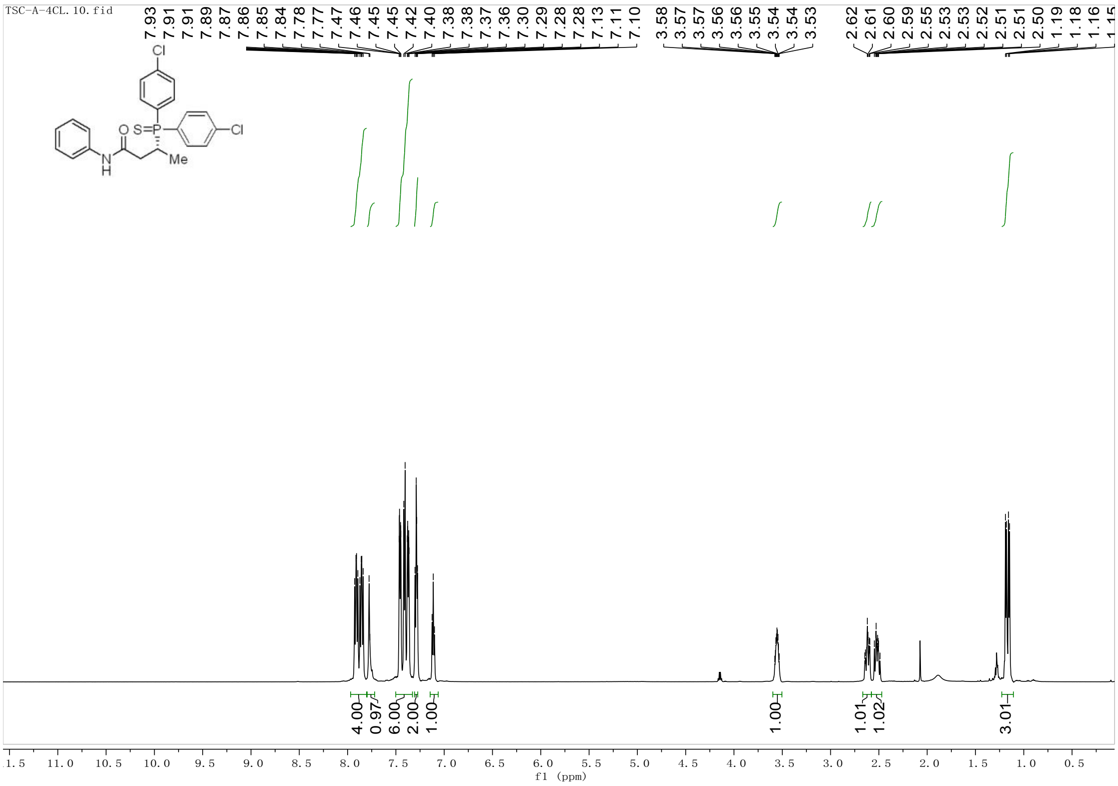

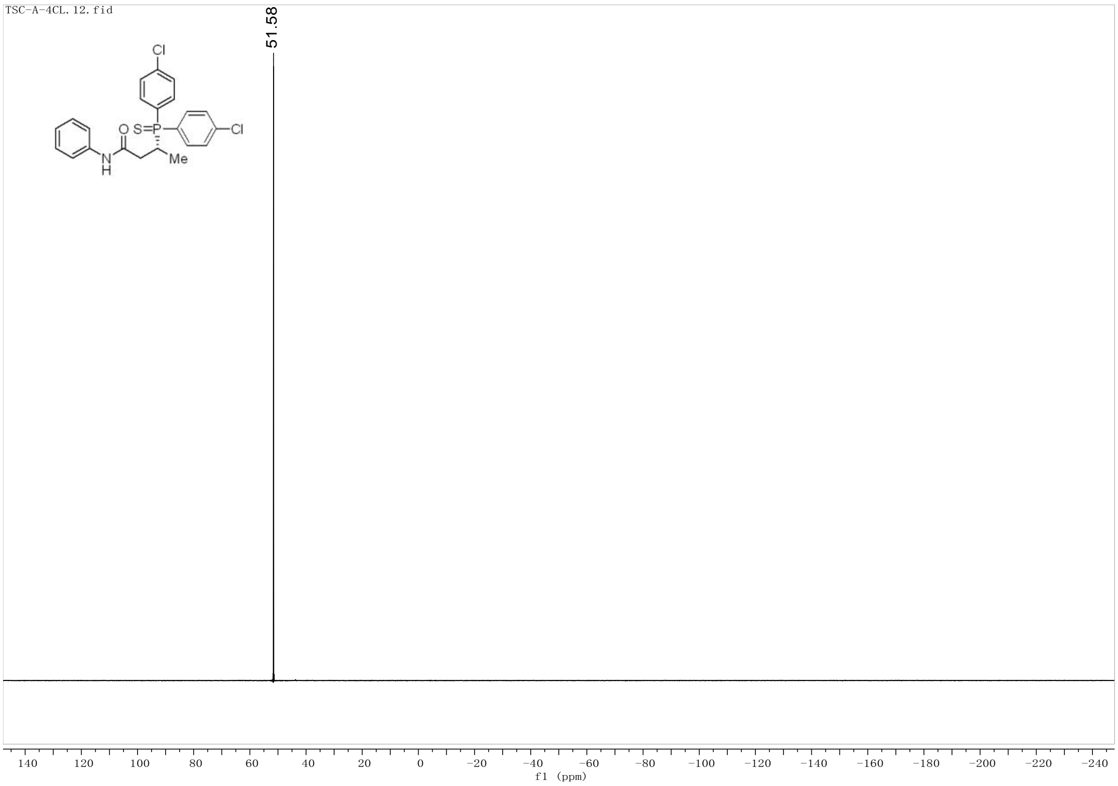

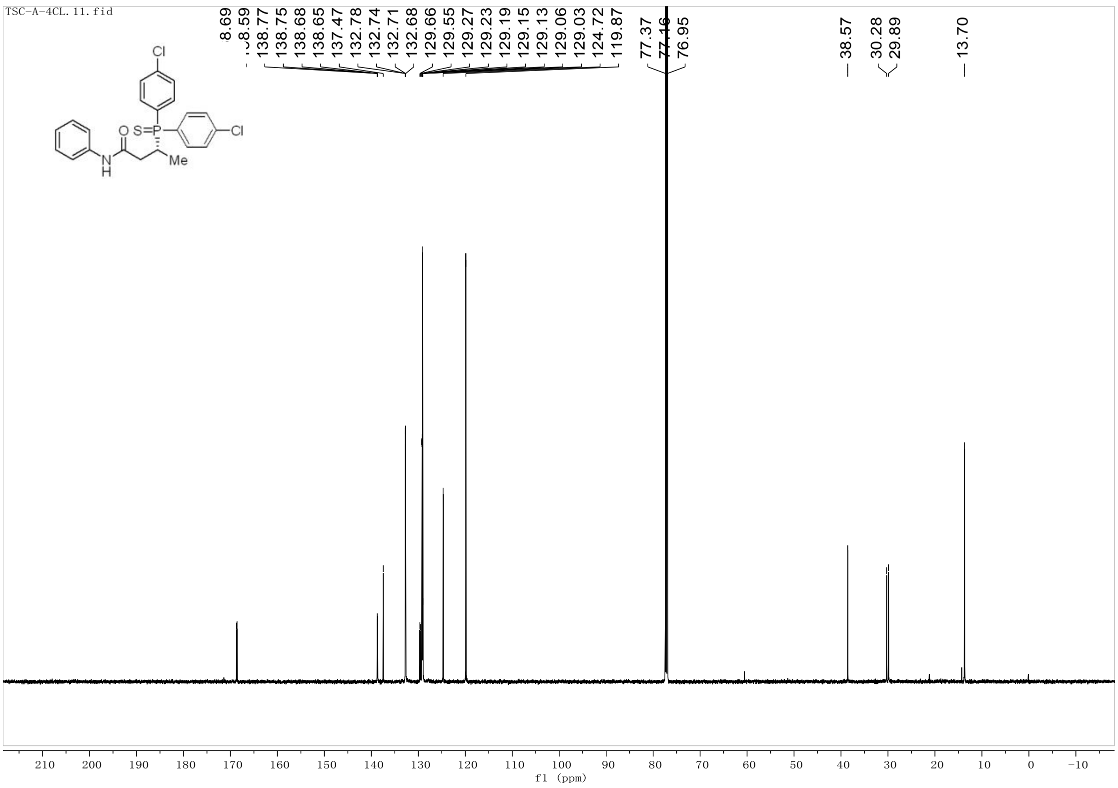

(*R*)-3-(bis(3-chlorophenyl)phosphorothioyl)-N-phenylbutanamide (3aj)

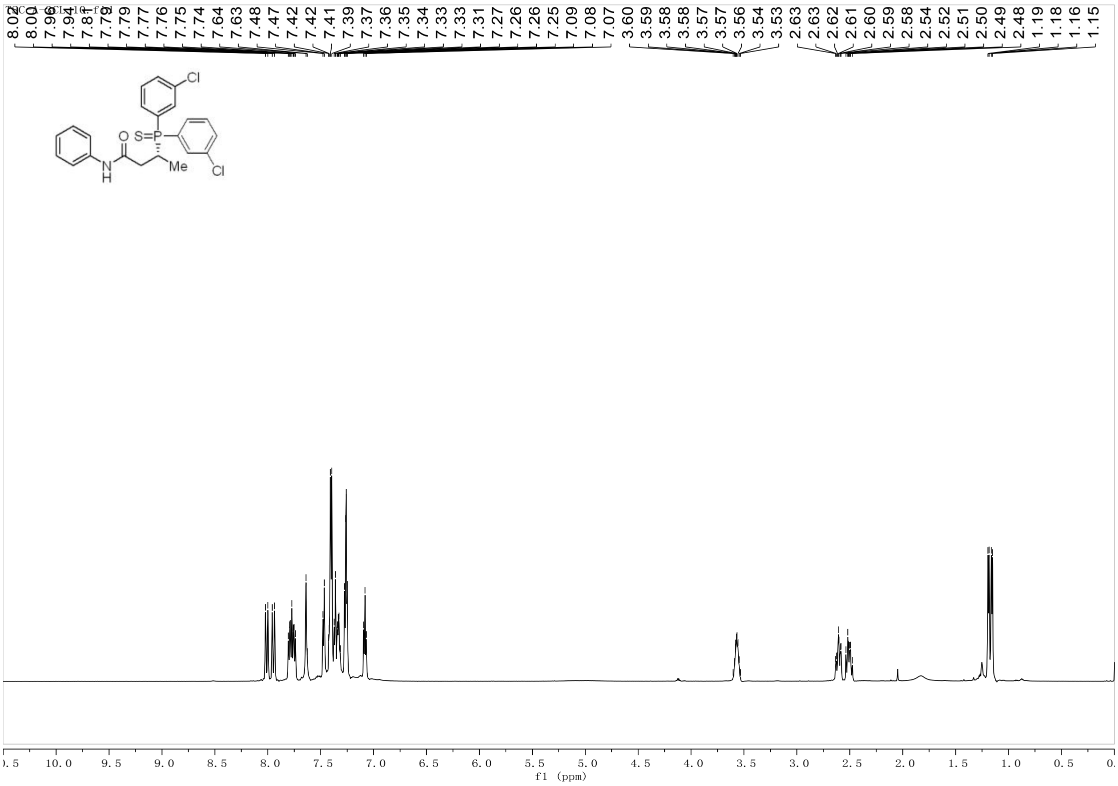

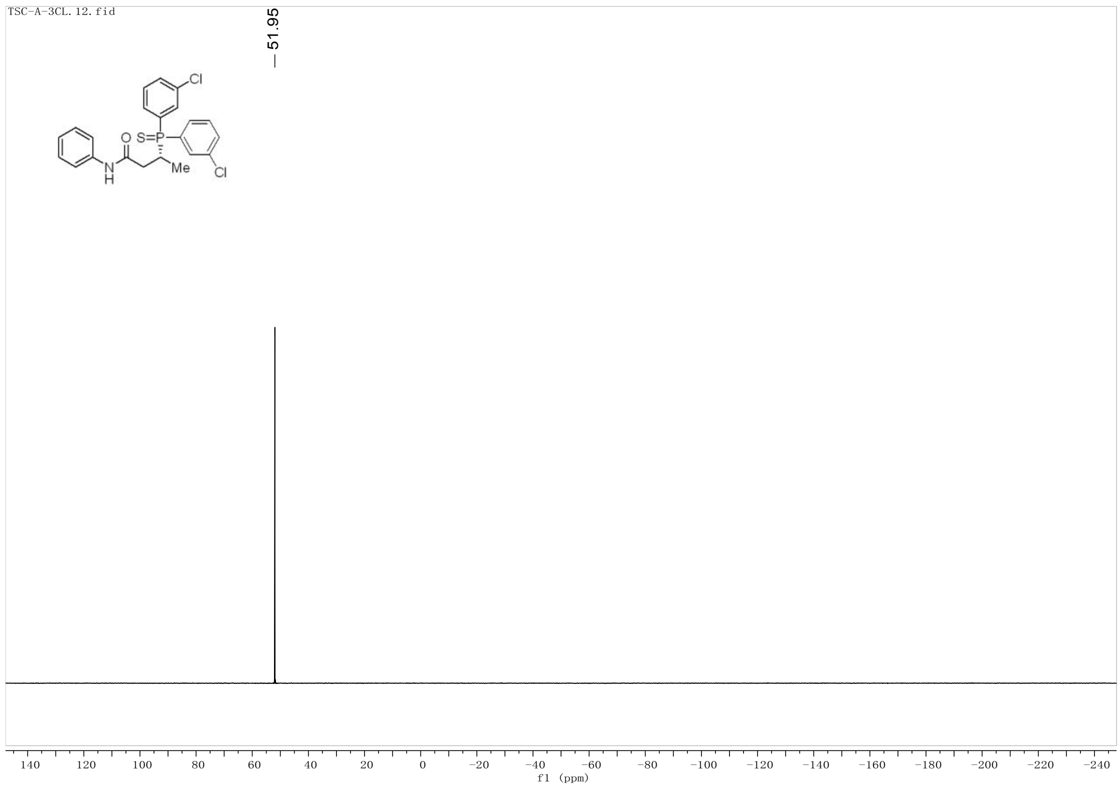
TSC-A-3CL.12.fid
51.95
Cl
S=P
O
N
H
Me
Cl
f1 (ppm)

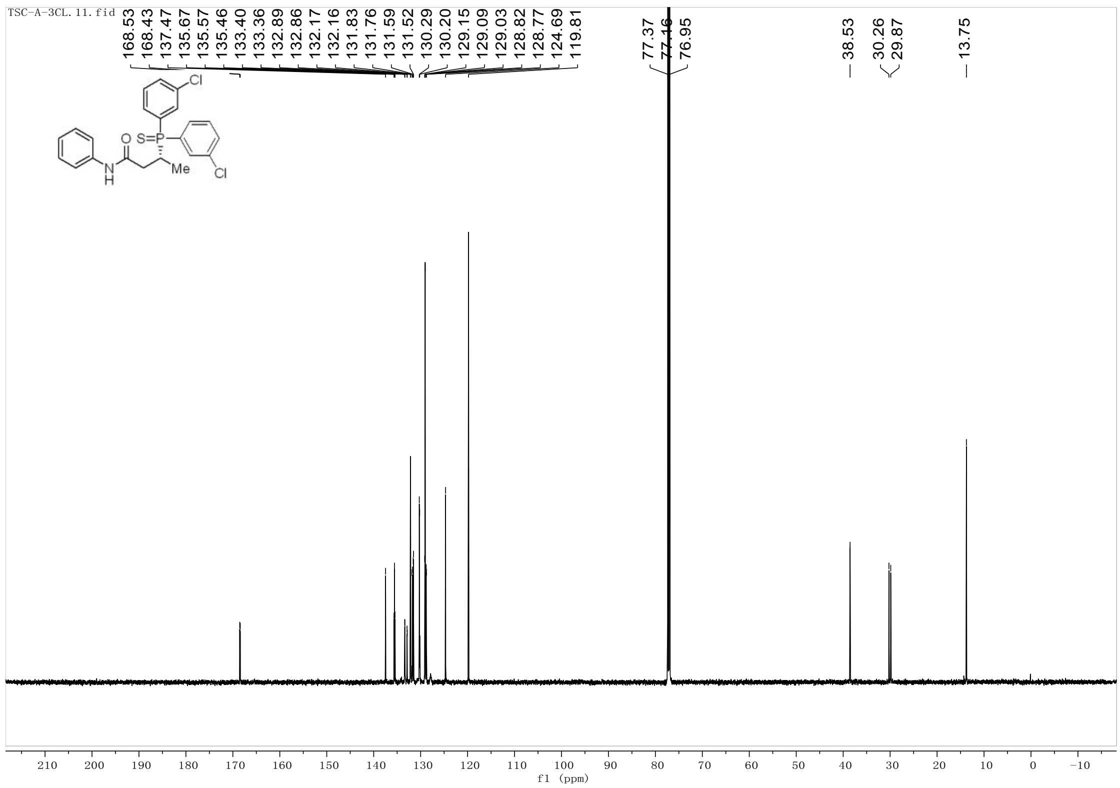
TSC-A-3CL.11.fid
168.53
168.43
137.47
135.67
135.57
135.46
133.40
133.36
132.89
132.86
132.17
132.16
131.83
131.76
131.59
131.52
130.29
130.20
129.15
129.09
129.03
128.82
128.77
124.69
119.81
77.37
77.16
76.95
38.53
30.26
29.87
13.75
Cl
S=P
O
N
H
Me
Cl
f1 (ppm)

(*R*)-3-(bis(4-(trifluoromethoxy)phenyl)phosphorothioyl)-N-phenylbutanamide (3ak)

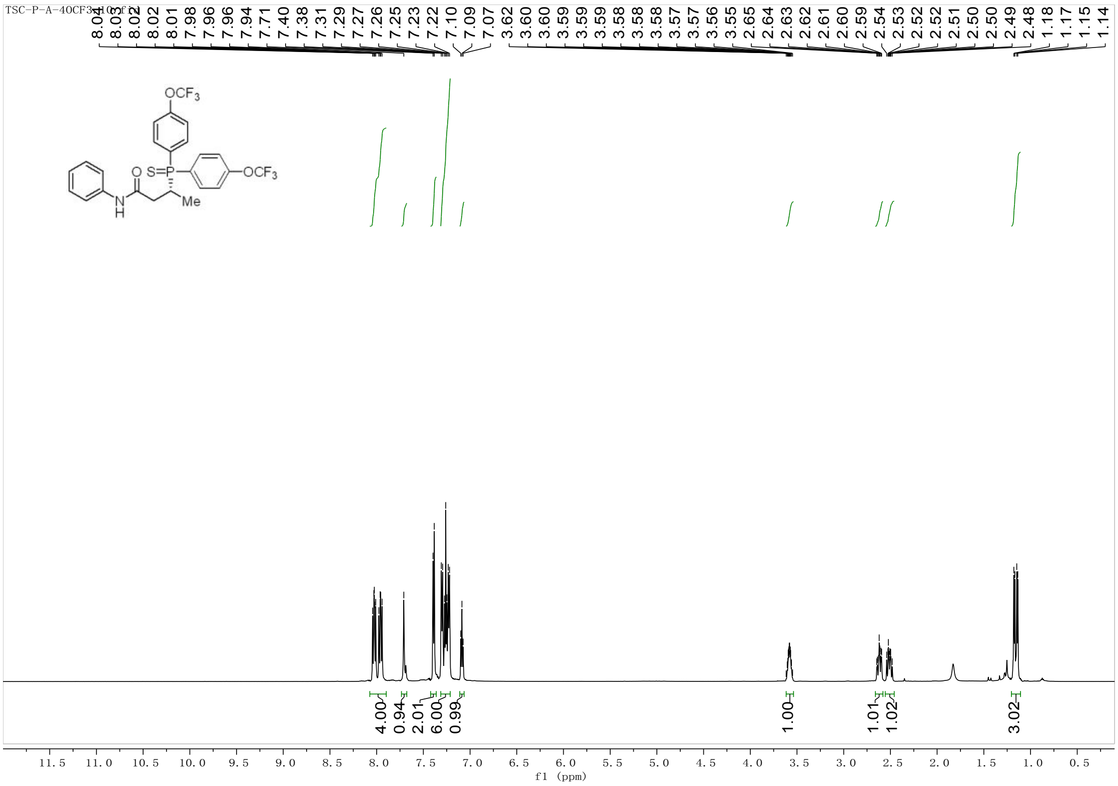

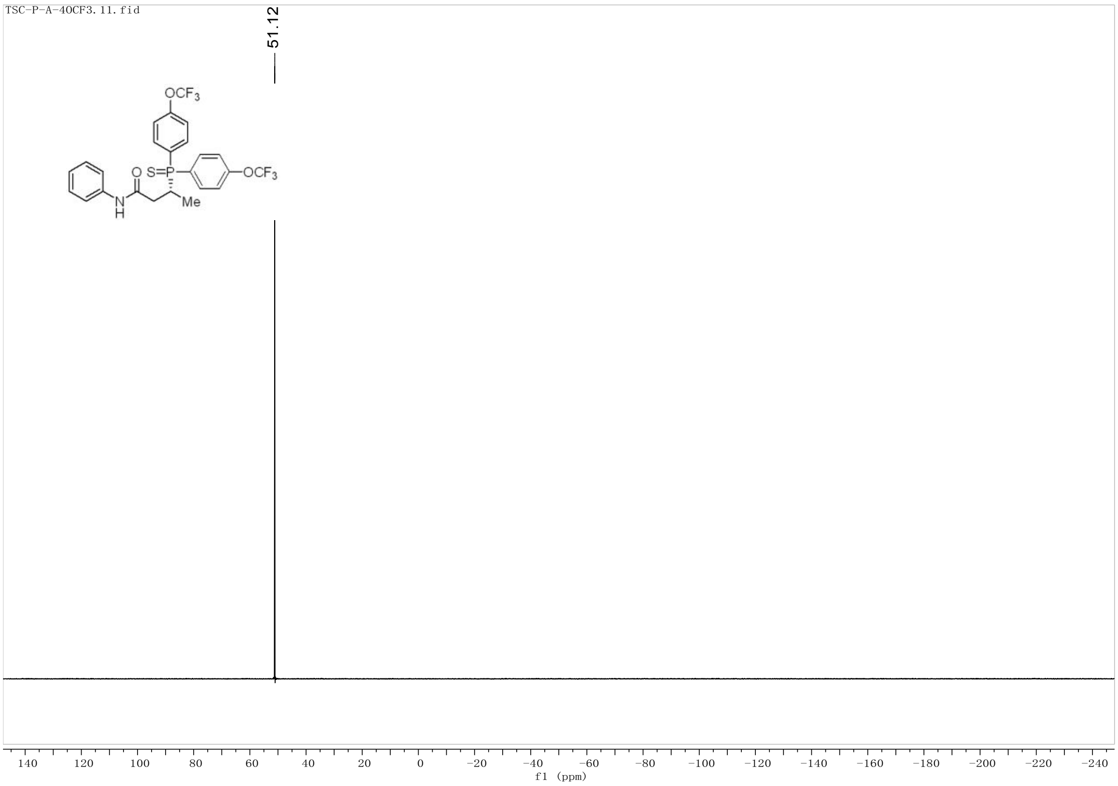

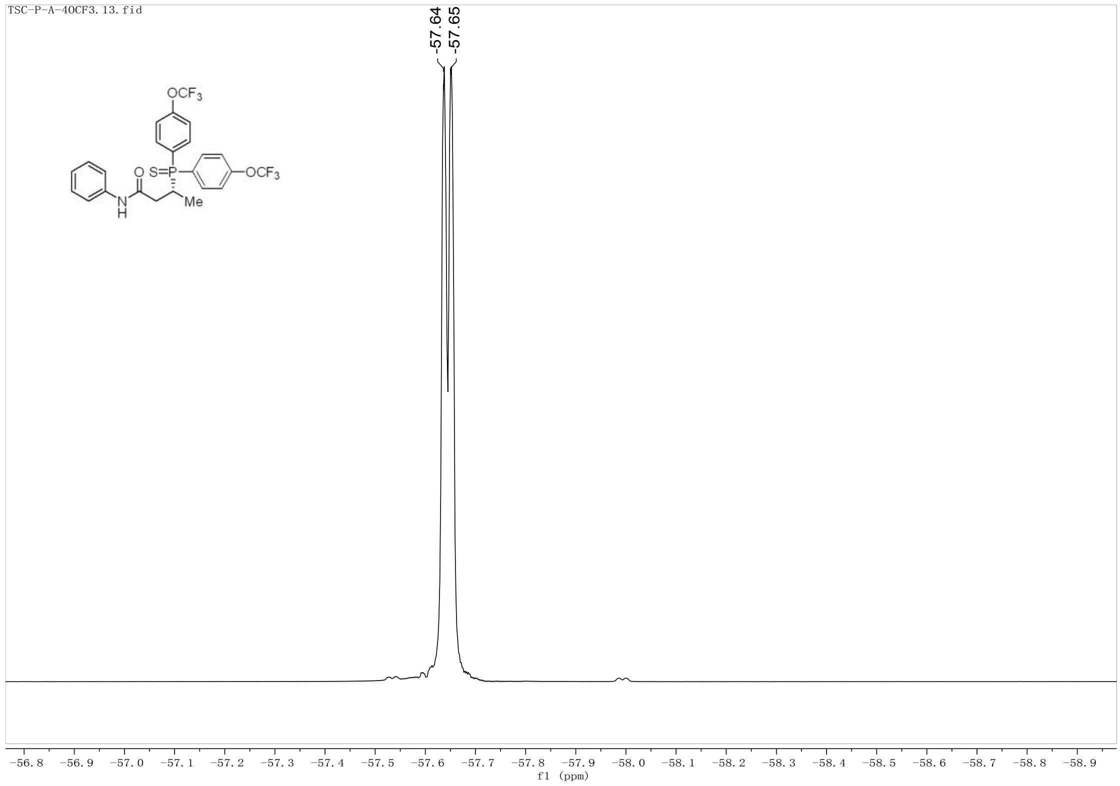
TSC-P-A-4OCF3.13.fid
-57.64
-57.65
OCF3
S=P
OCF3
O
N
H
Me
-56.8 -56.9 -57.0 -57.1 -57.2 -57.3 -57.4 -57.5 -57.6 -57.7 -57.8 -57.9 -58.0 -58.1 -58.2 -58.3 -58.4 -58.5 -58.6 -58.7 -58.8 -58.9
f1 (ppm)

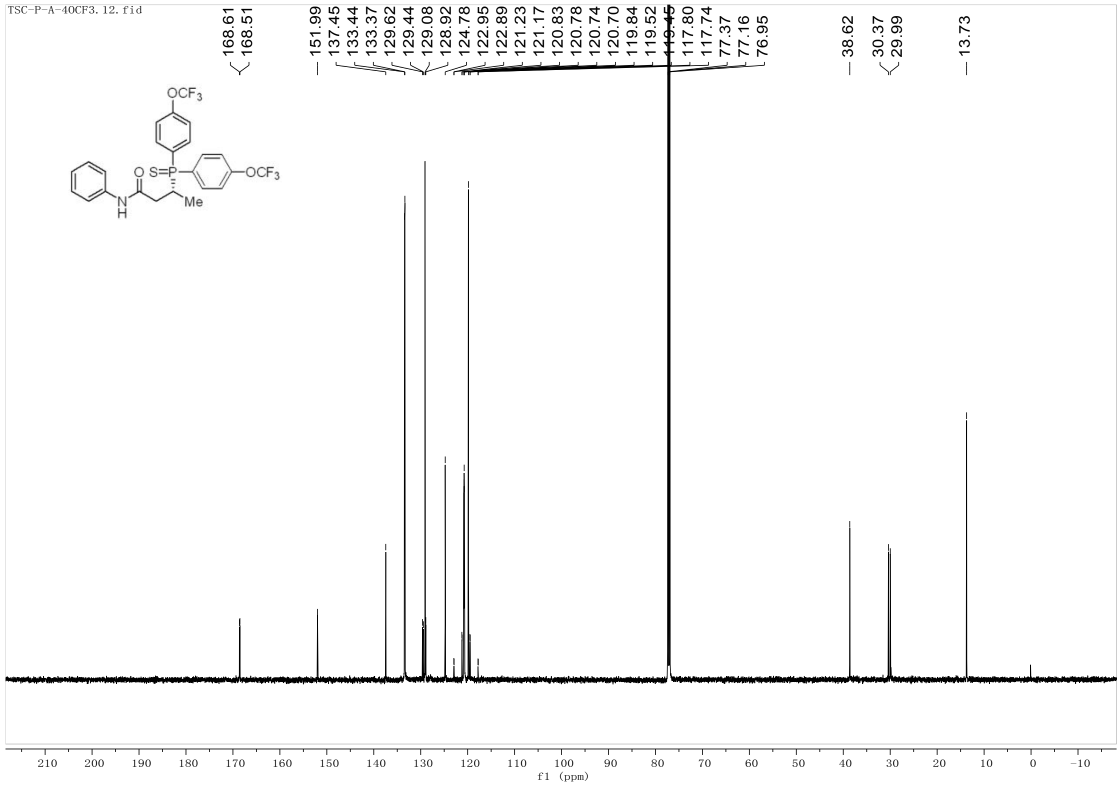
TSC-P-A-4OCF3.12.fid
168.61
168.51
151.99
137.45
133.44
133.37
129.62
129.44
129.08
128.92
124.78
122.95
122.89
121.23
121.17
120.83
120.78
120.74
120.70
119.84
119.52
117.80
117.74
77.37
77.16
76.95
38.62
30.37
29.99
13.73
OCF3
S=P
OCF3
O
N
H
Me
210 200 190 180 170 160 150 140 130 120 110 100 90 80 70 60 50 40 30 20 10 0 -10
f1 (ppm)

(*R*)-3-(di([1,1'-biphenyl]-4-yl)phosphorothioyl)-N-phenylbutanamide (3al)

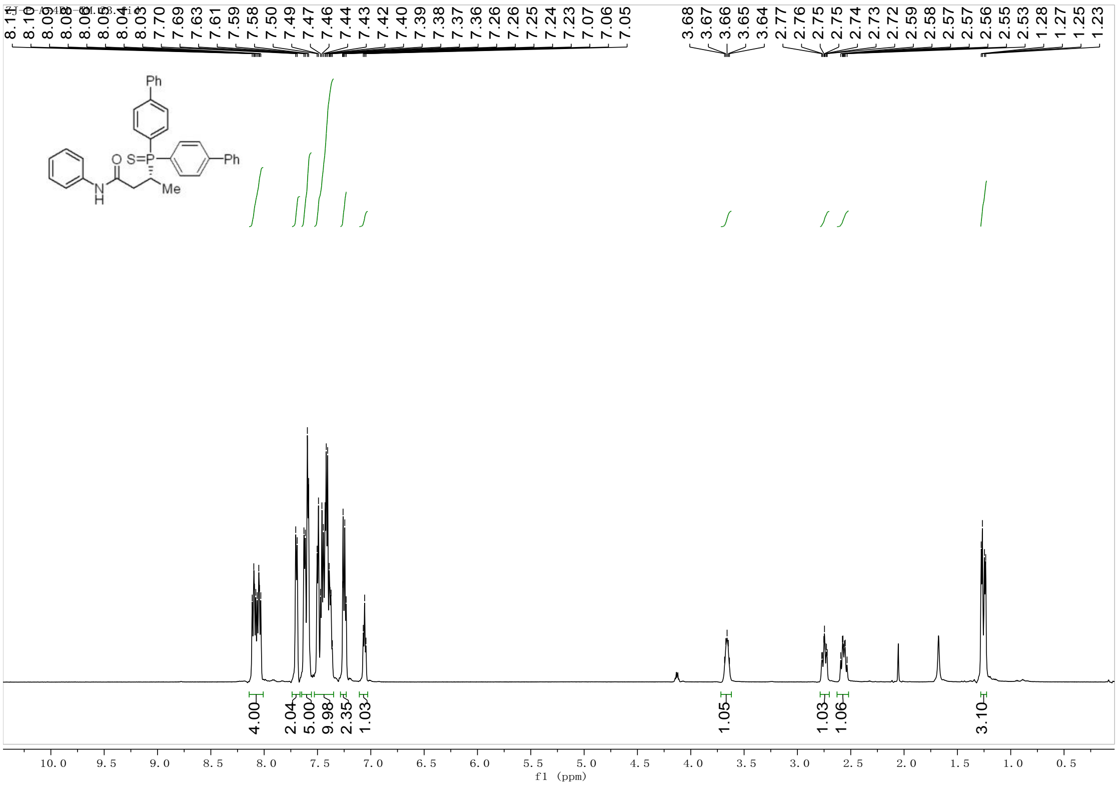

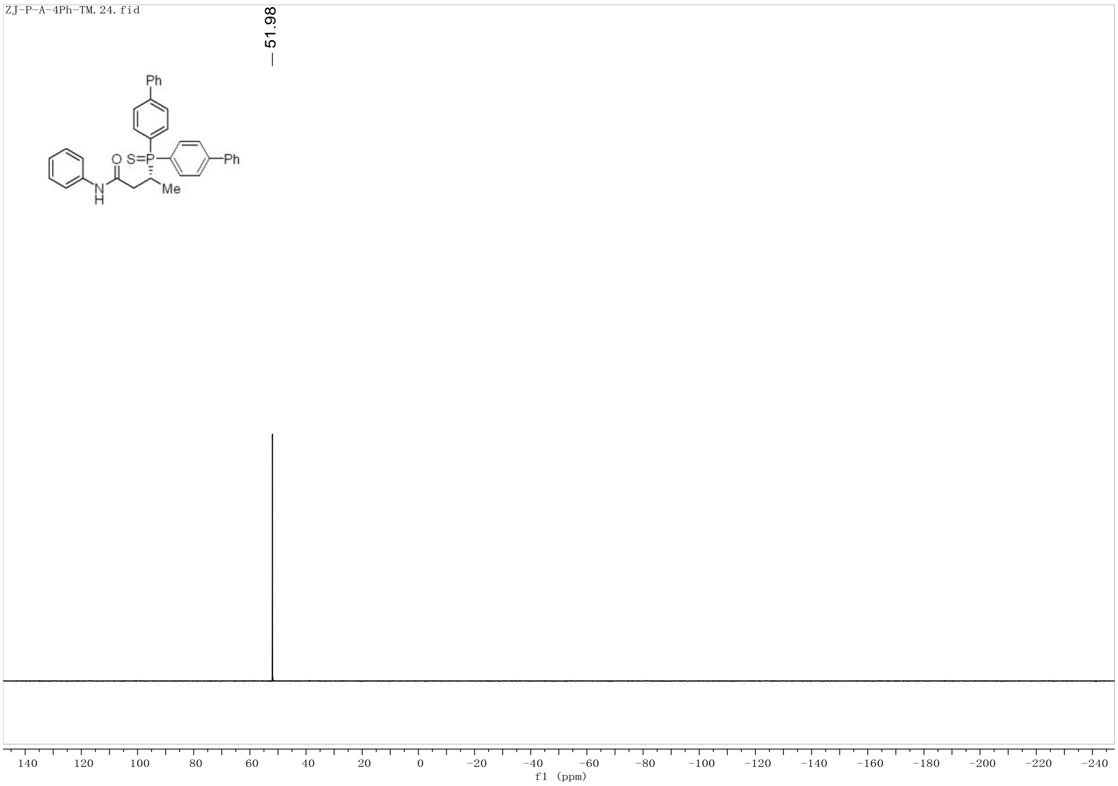

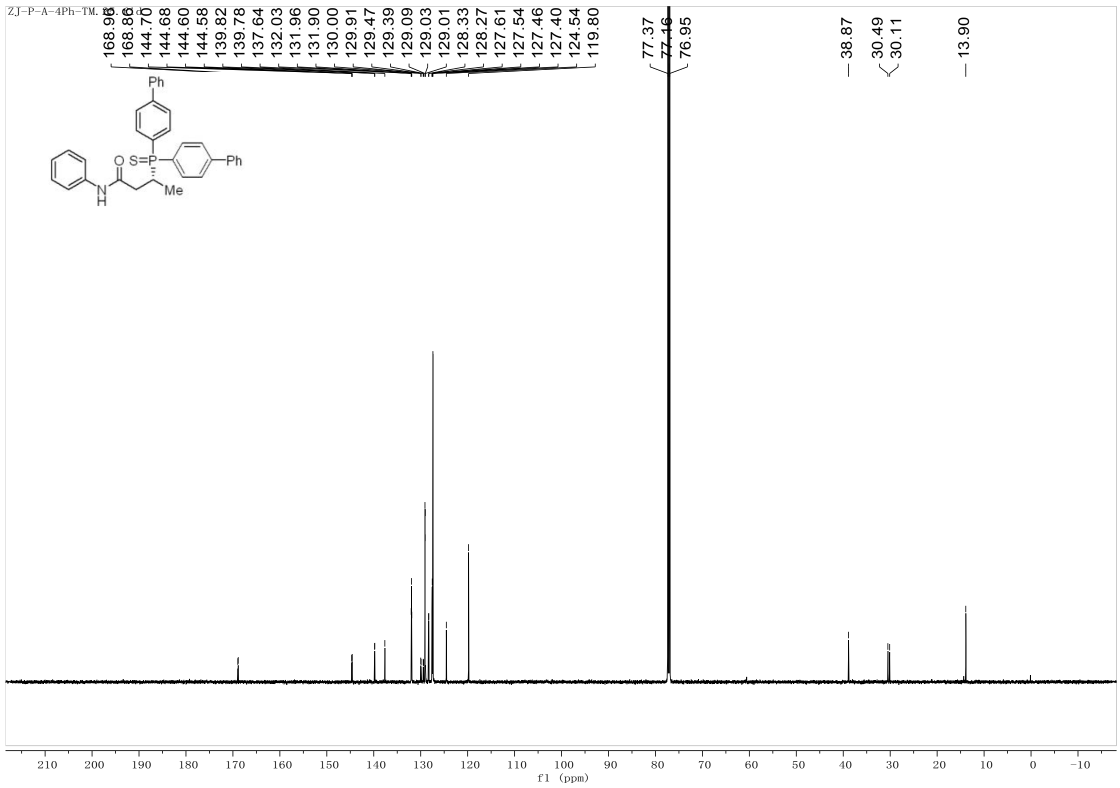

(*R*)-3-(di(naphthalen-2-yl)phosphorothioyl)-N-phenylbutanamide (3am)

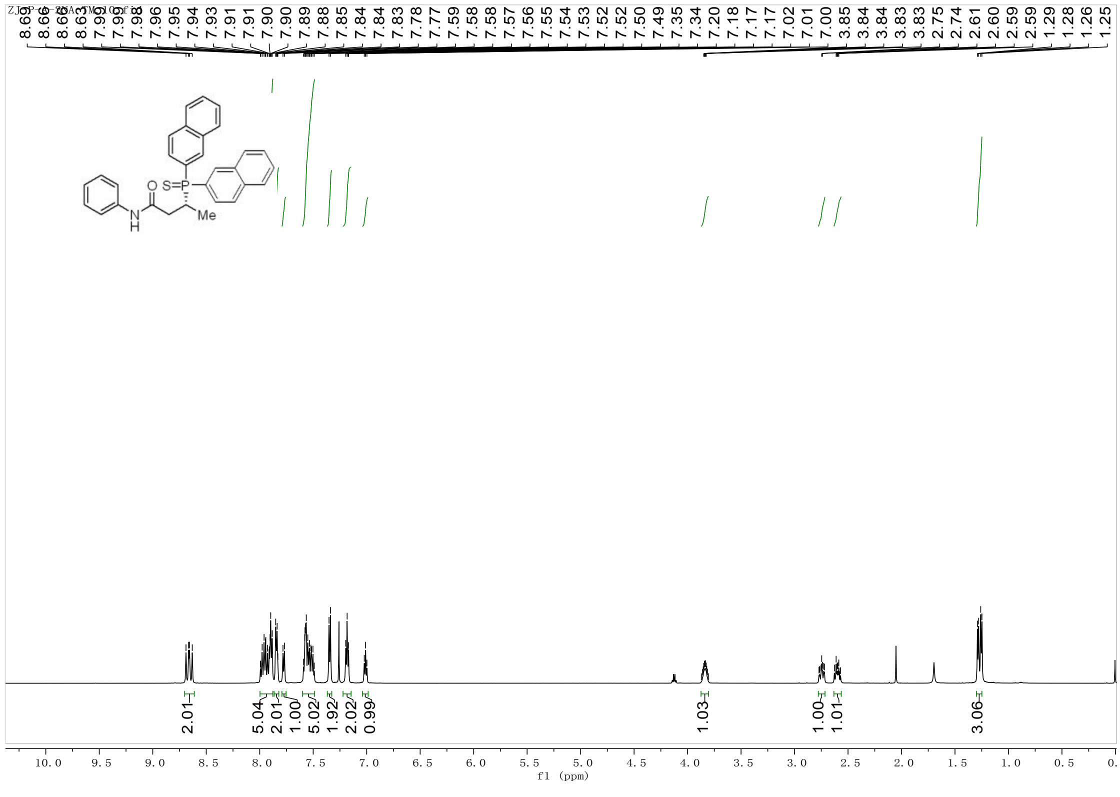

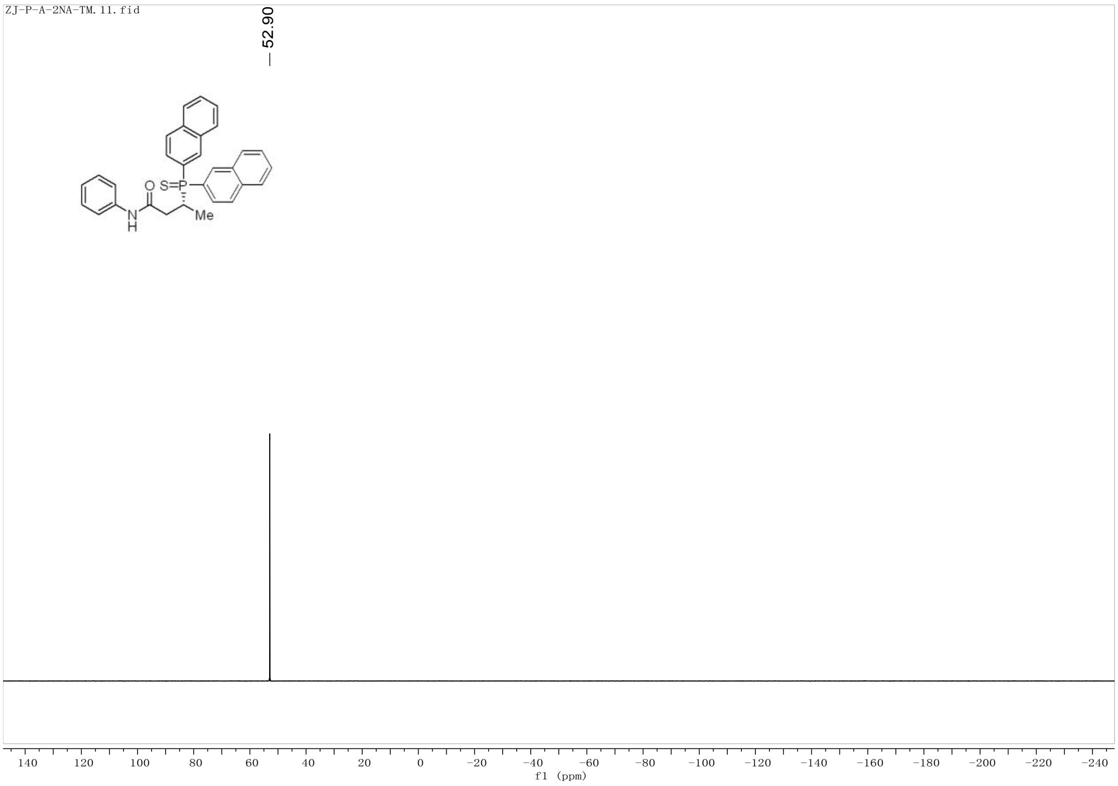
ZJ-P-A-2NA-TM.11.fid
52.90
S=P
O
N
H
Me
f1 (ppm)

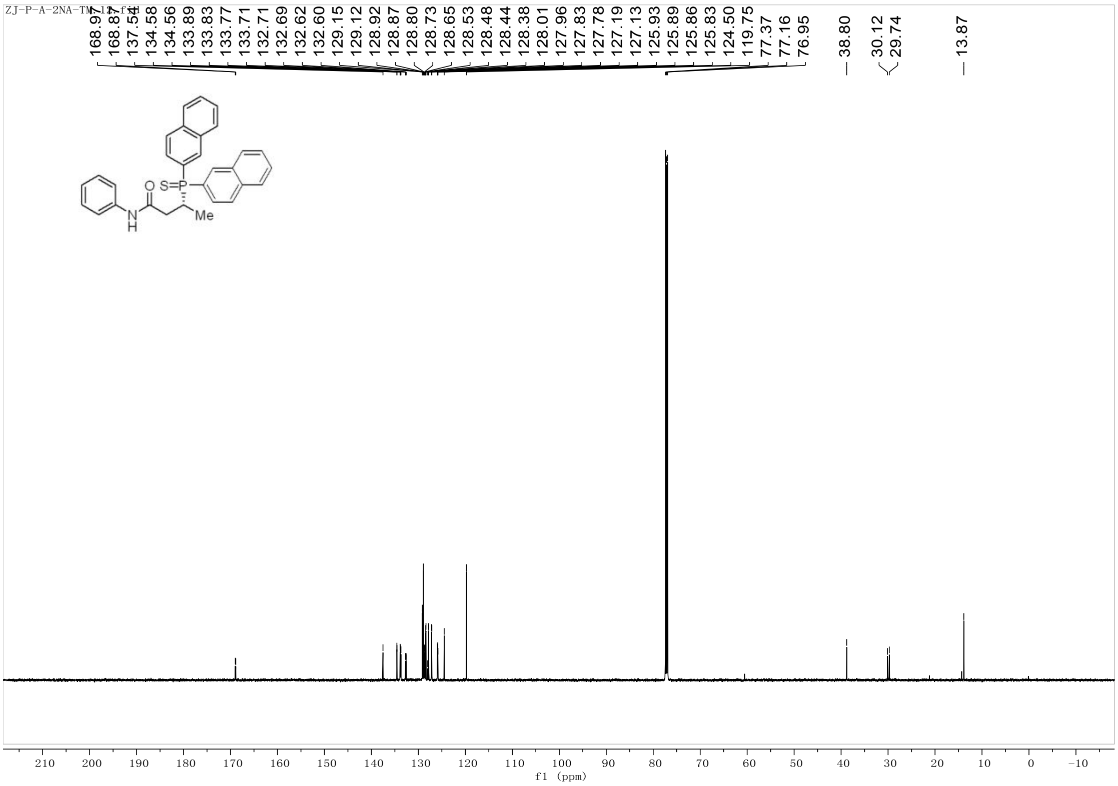
ZJ-P-A-2NA-TM
168.91
168.87
137.54
134.58
134.56
133.89
133.83
133.77
133.71
132.71
132.69
132.62
132.60
129.15
129.12
128.92
128.87
128.80
128.73
128.65
128.53
128.48
128.44
128.38
128.01
127.96
127.83
127.78
127.19
127.13
125.93
125.89
125.86
125.83
124.50
119.75
77.37
77.16
76.95
38.80
30.12
29.74
13.87
S=P
O
N
H
Me
f1 (ppm)

4-(diphenylphosphorothioyl)-N-phenylbutanamide (4a)

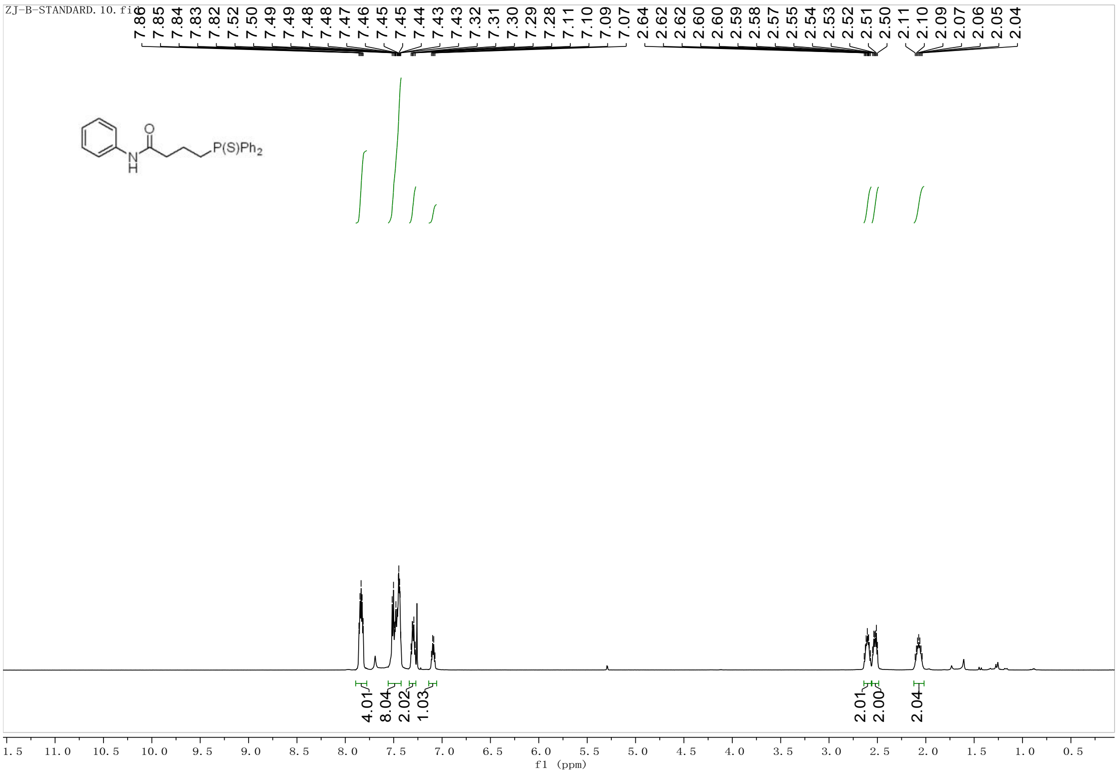

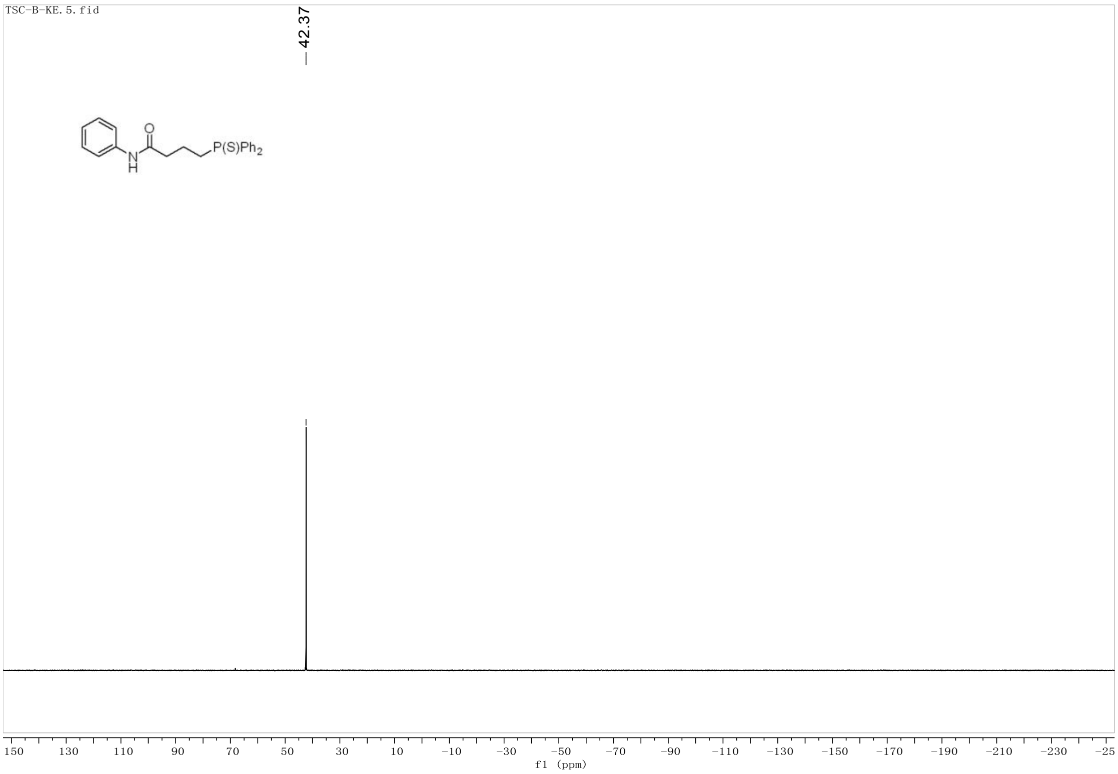

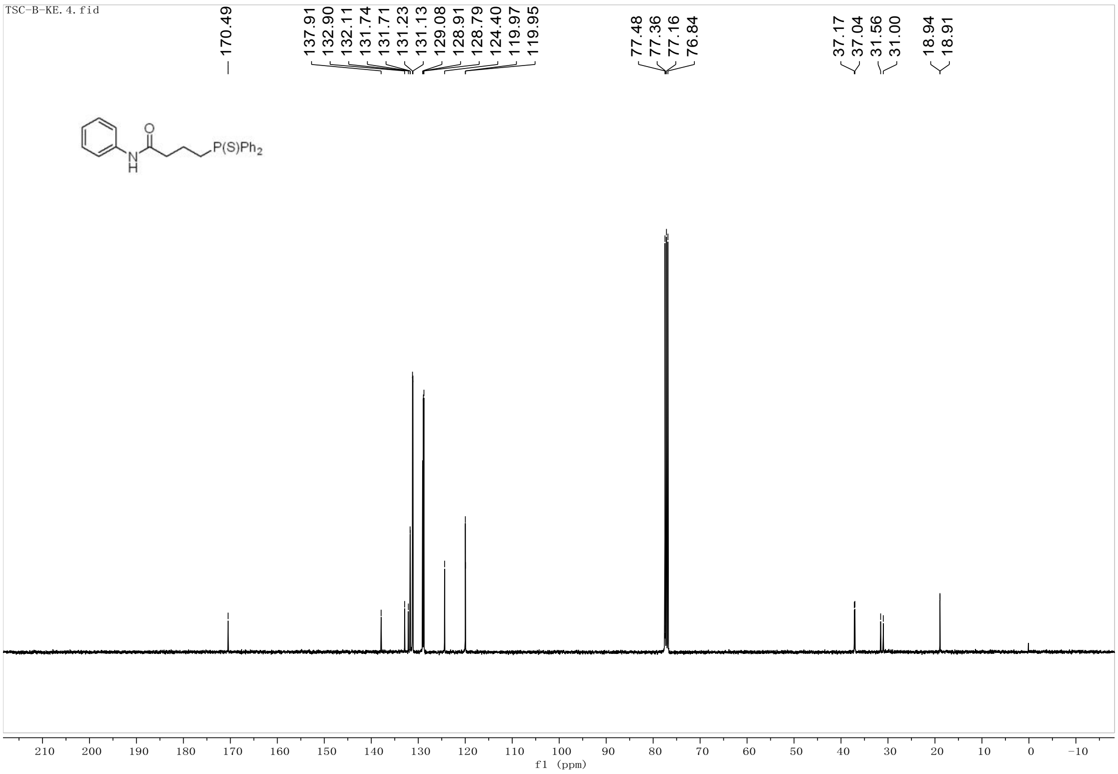

4-(diphenylphosphorothioyl)-N-(o-tolyl)butanamide (4b)

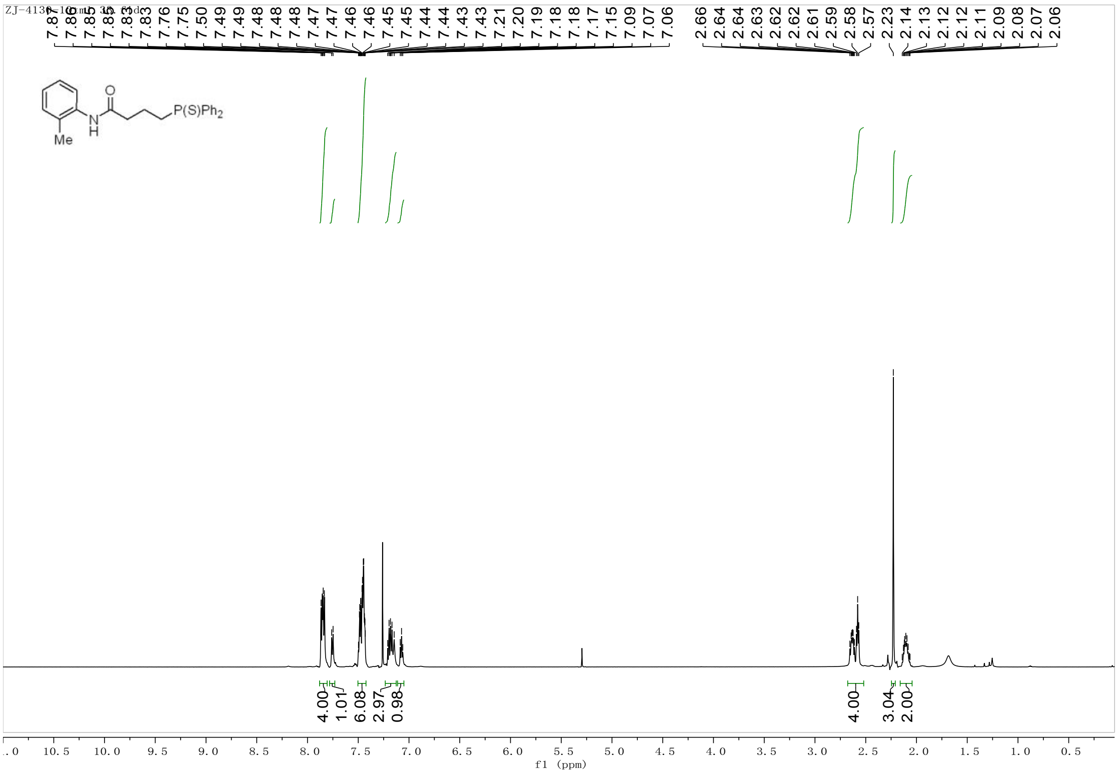

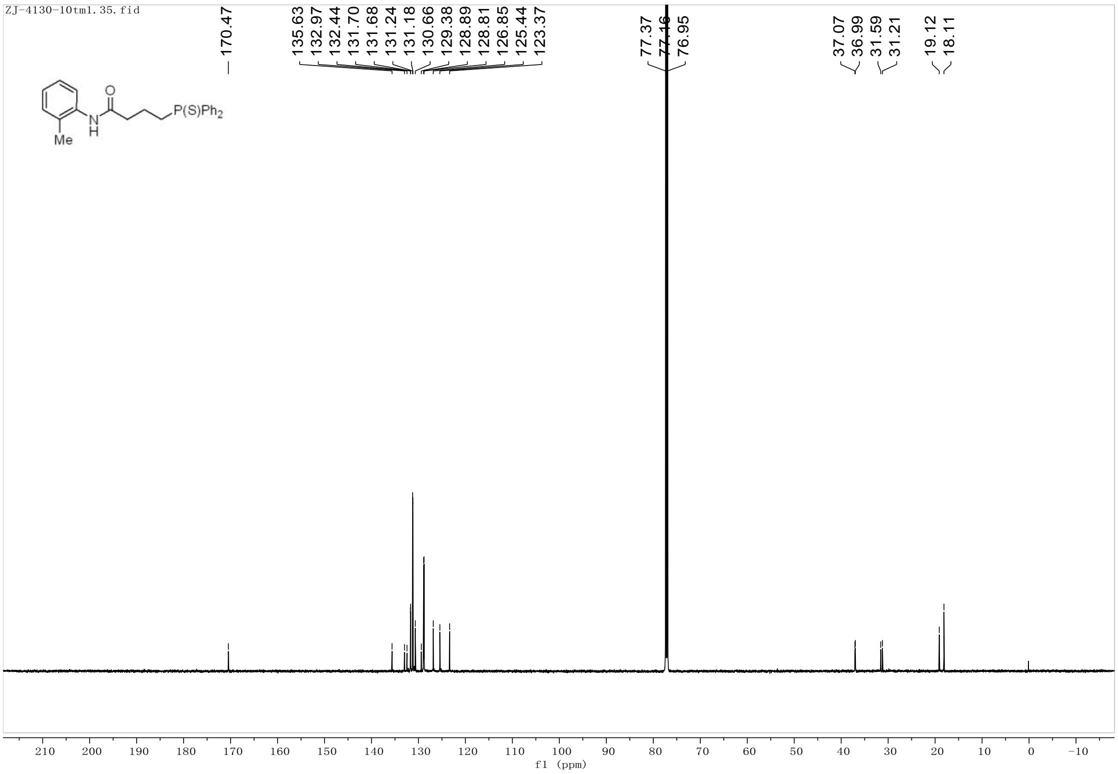
ZJ-4130-10tm1.35.fid
170.47
135.63
132.97
132.44
131.70
131.68
131.24
131.18
130.66
129.38
128.89
128.81
126.85
125.44
123.37
77.37
77.16
76.95
37.07
36.99
31.59
31.21
19.12
18.11
Me
N
H
O
P(S)Ph2
f1 (ppm)

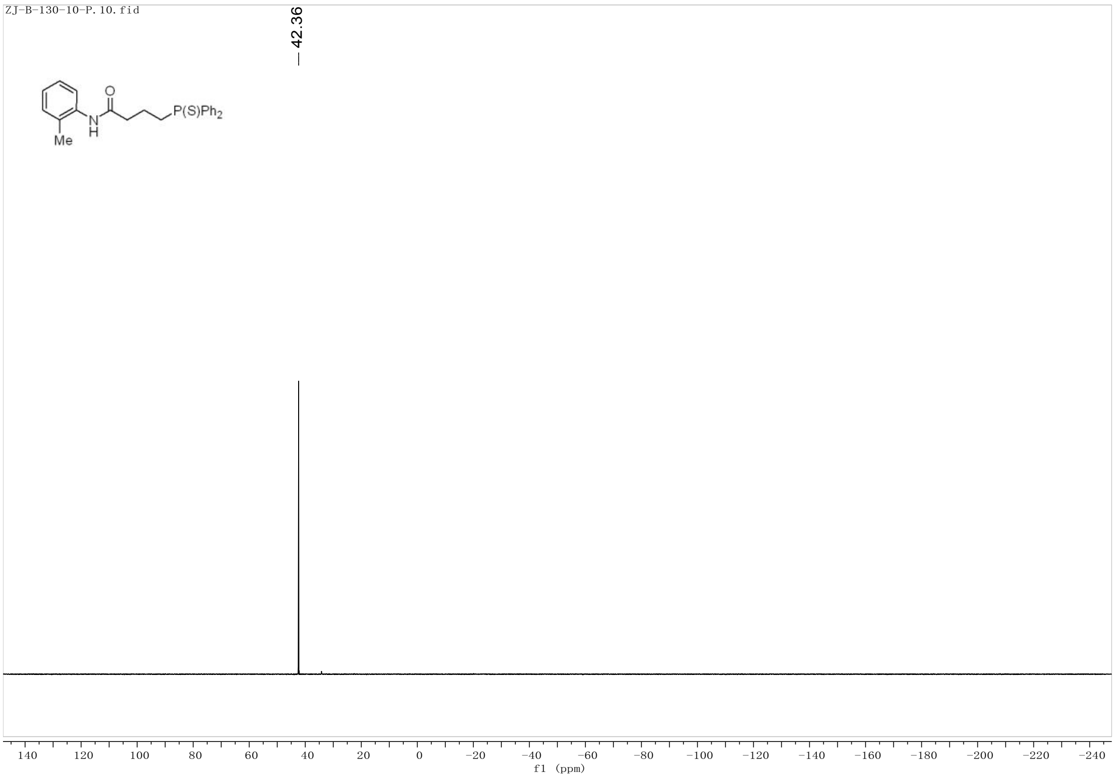
ZJ-B-130-10-P.10.fid
42.36
Me
N
H
O
P(S)Ph2
f1 (ppm)

4-(diphenylphosphorothioyl)-N-(m-tolyl)butanamide (4c)

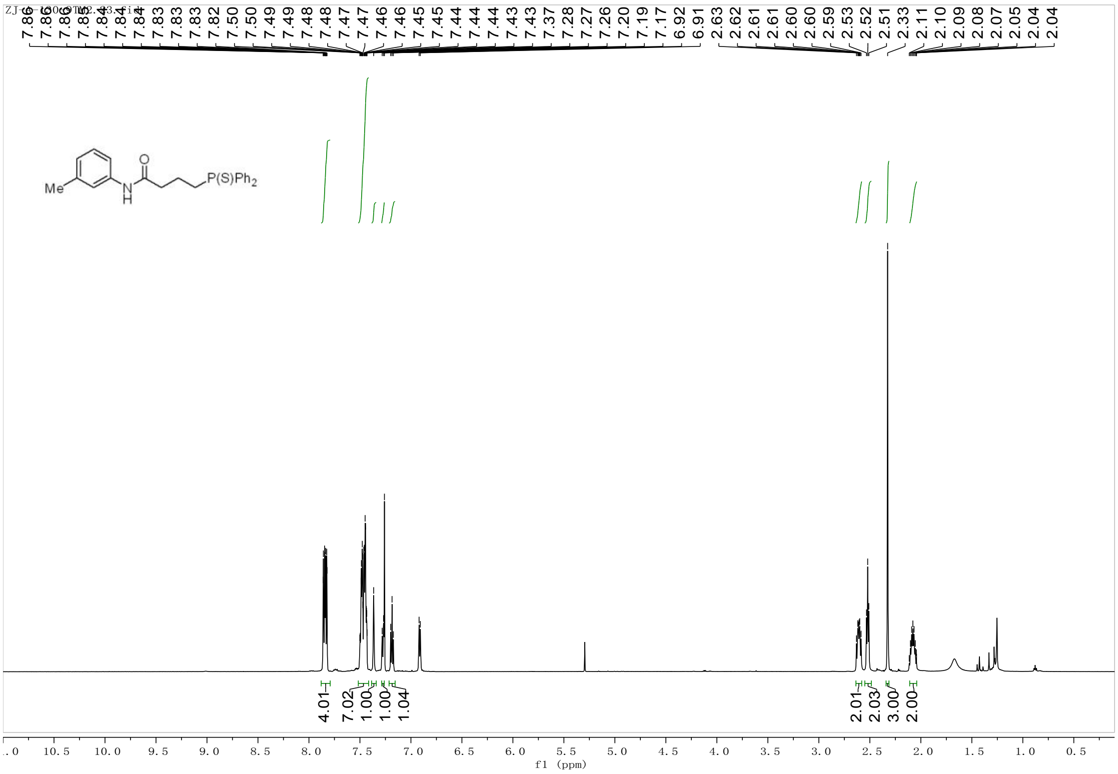

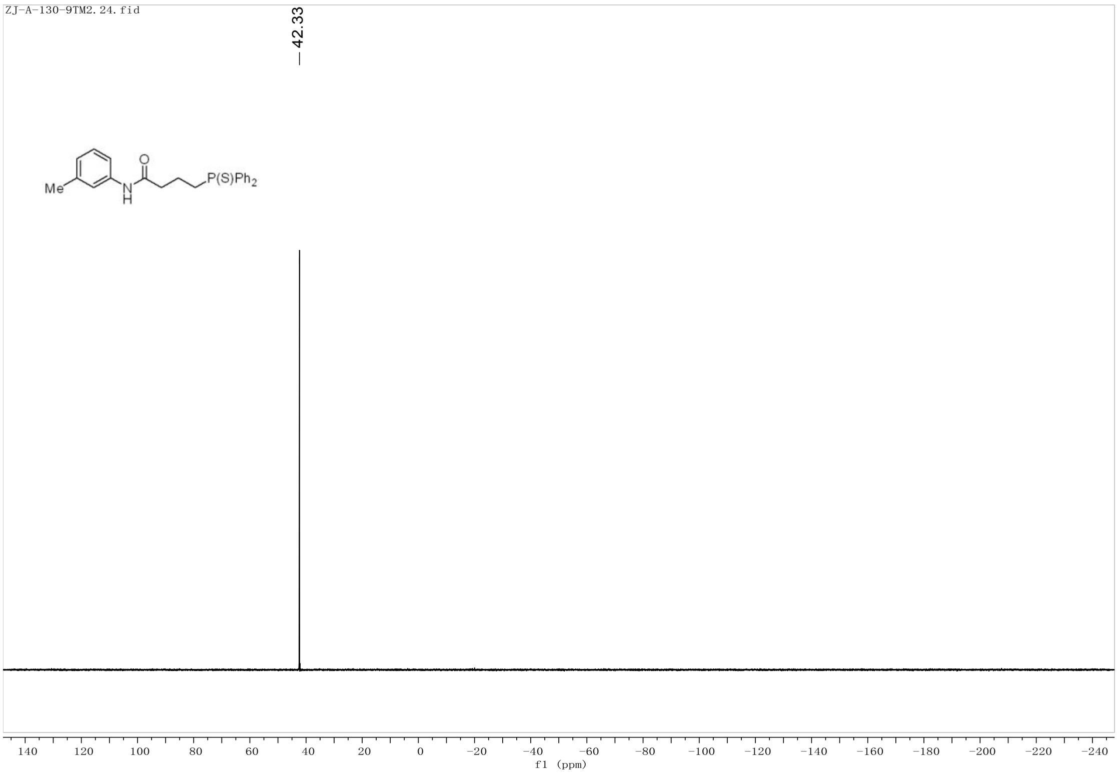

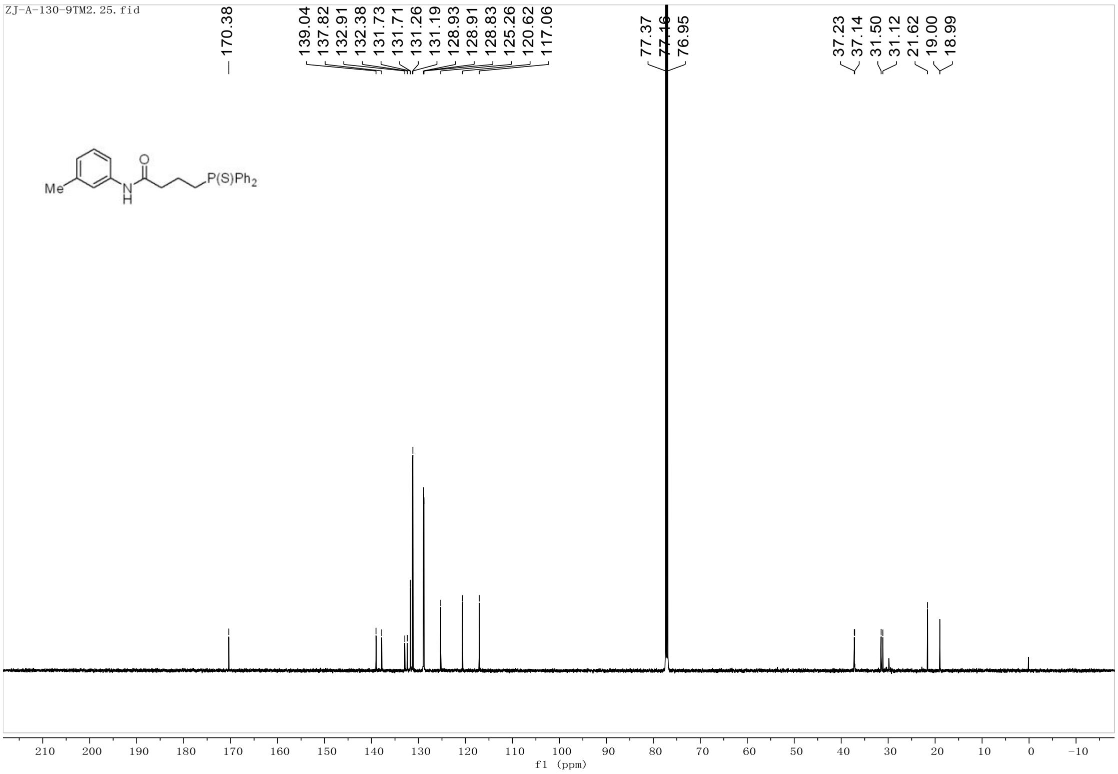

4-(diphenylphosphorothioyl)-N-(p-tolyl)butanamide (4d)

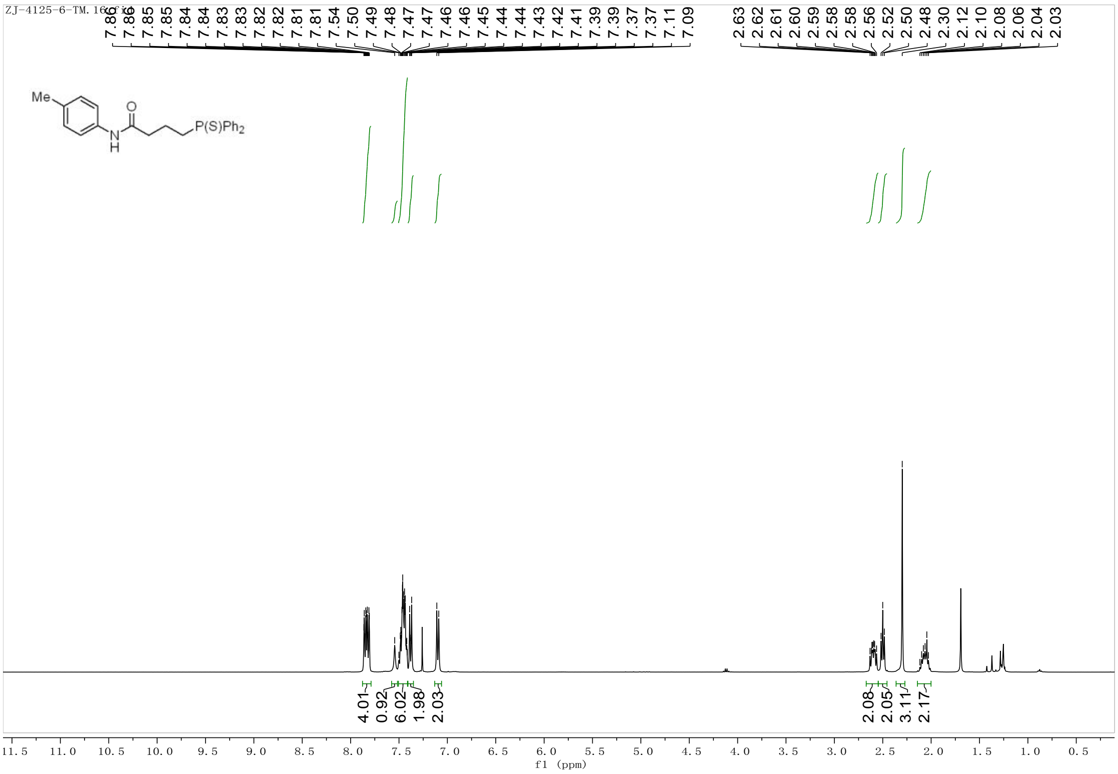

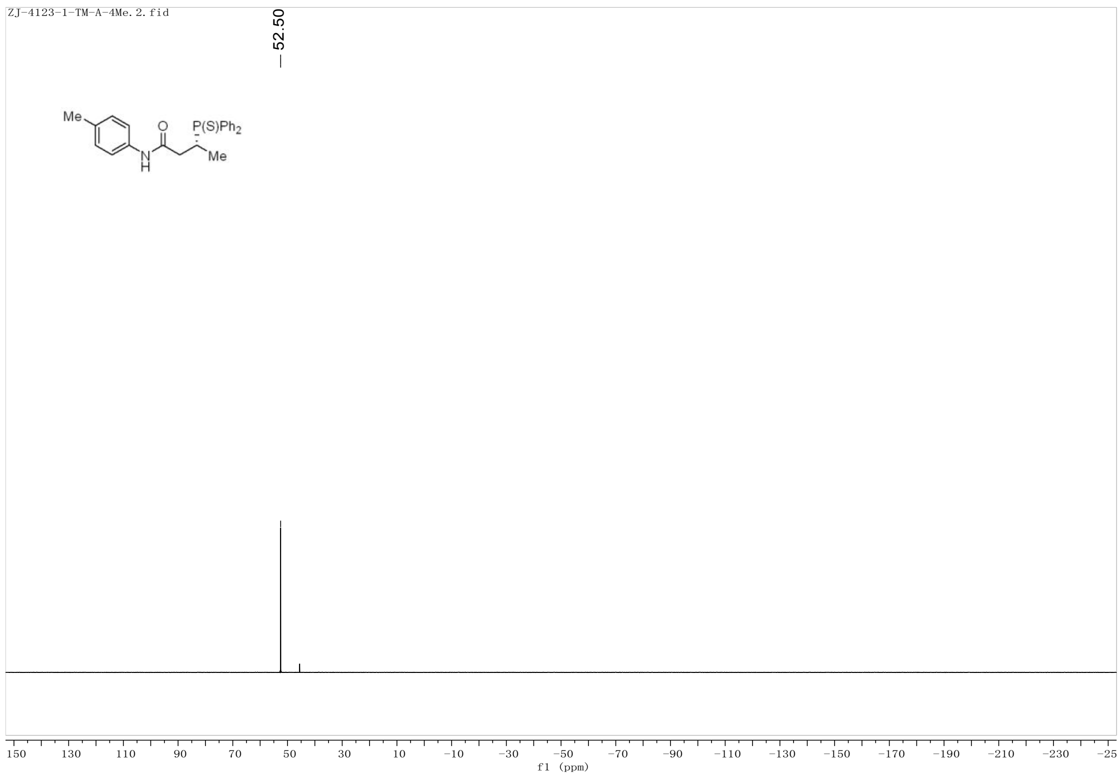
ZJ-4123-1-TM-A-4Me.2.fid
52.50
Me
O
P(S)Ph2
N
H
Me
150 130 110 90 70 50 30 10 −10 −30 −50 −70 −90 −110 −130 −150 −170 −190 −210 −230 −25
f1 (ppm)

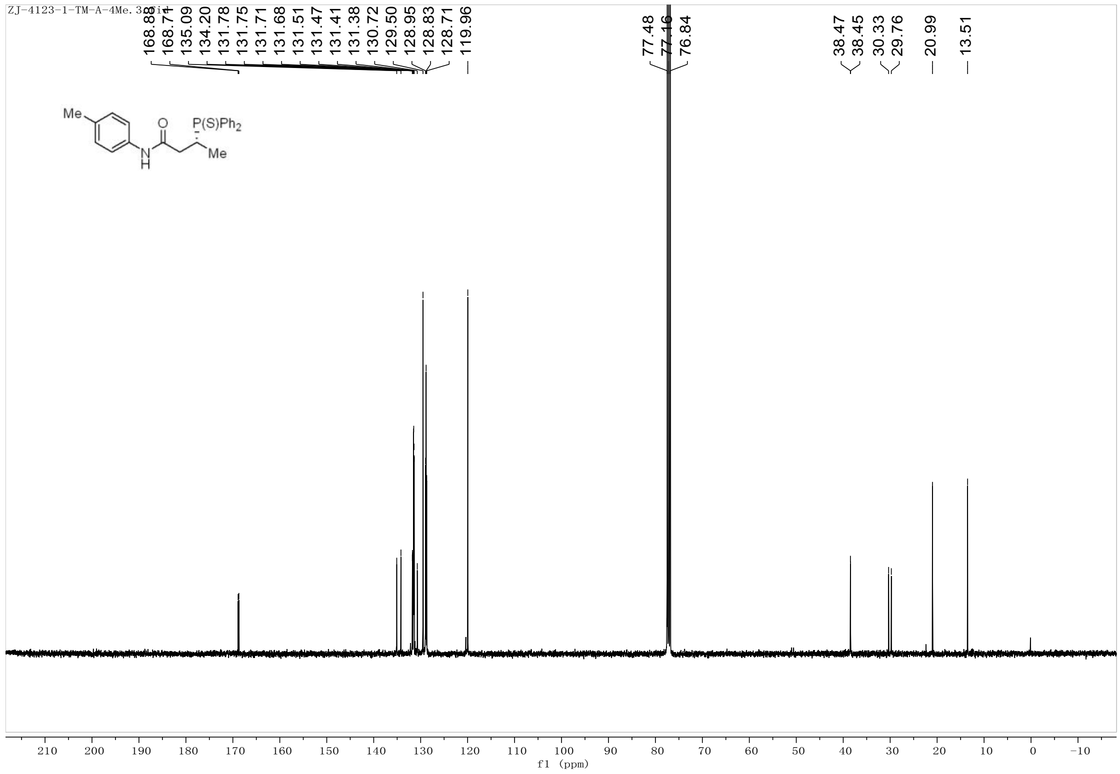
ZJ-4123-1-TM-A-4Me.3
168.88
168.71
135.09
134.20
131.78
131.75
131.71
131.68
131.51
131.47
131.41
131.38
130.72
129.50
128.95
128.83
128.71
119.96
77.48
77.16
76.84
38.47
38.45
30.33
29.76
20.99
13.51
Me
O
P(S)Ph2
N
H
Me
210 200 190 180 170 160 150 140 130 120 110 100 90 80 70 60 50 40 30 20 10 0 −10
f1 (ppm)

N-(4-(tert-butyl)phenyl)-4-(diphenylphosphorothioyl)butanamide (4e)

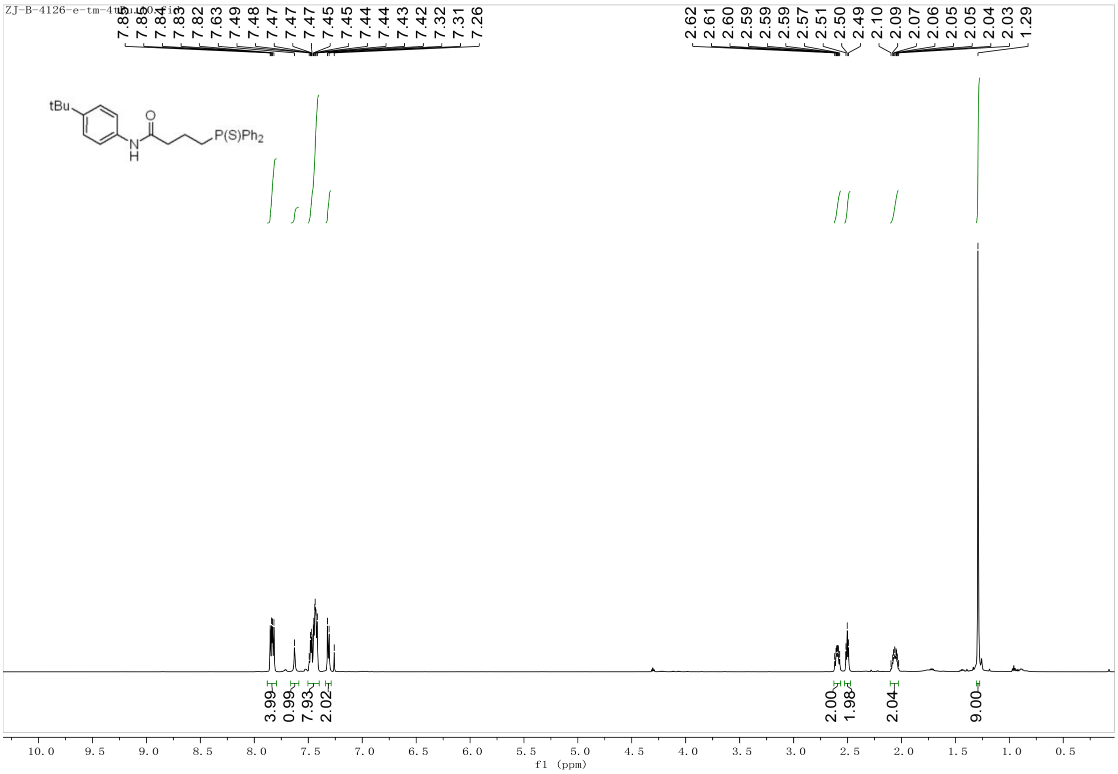

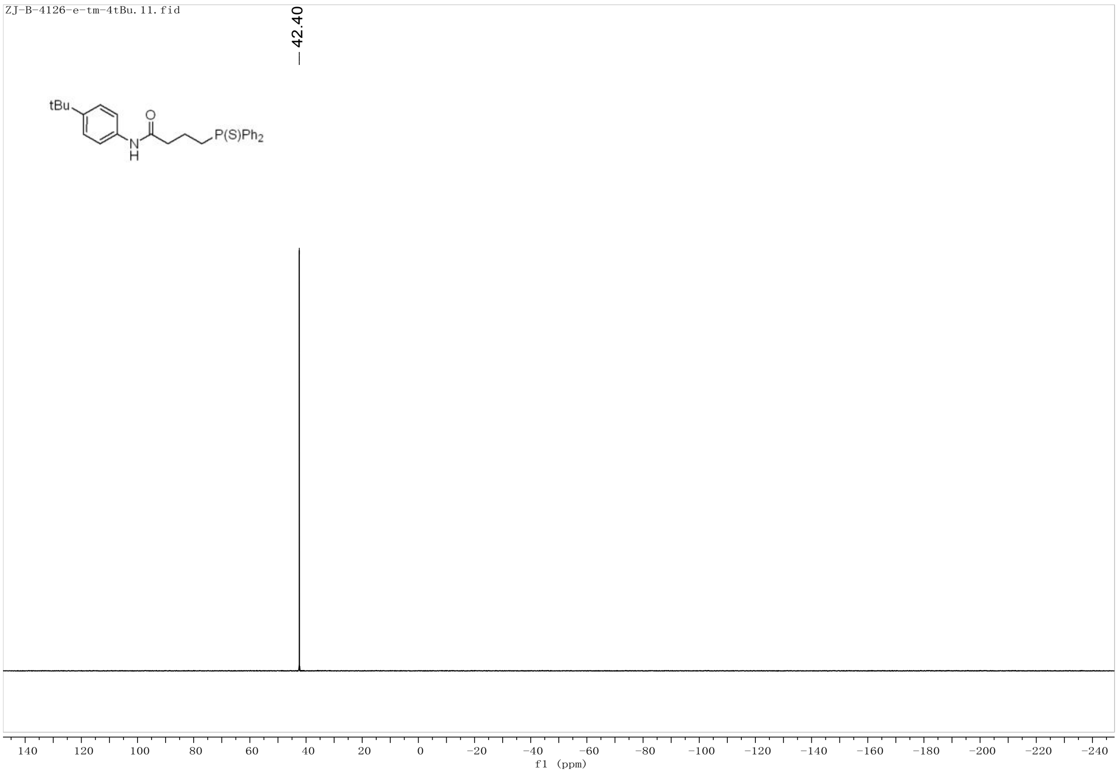

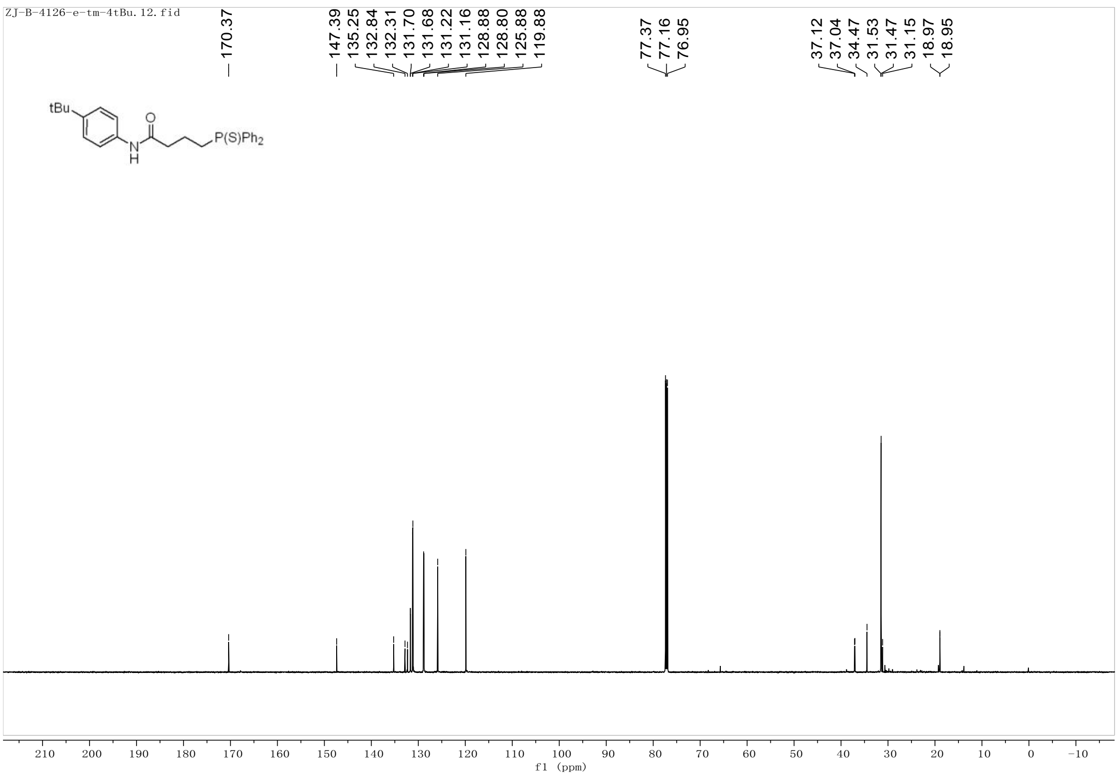

N-(4-benzylphenyl)-4-(diphenylphosphorothioyl)butanamide (4f)

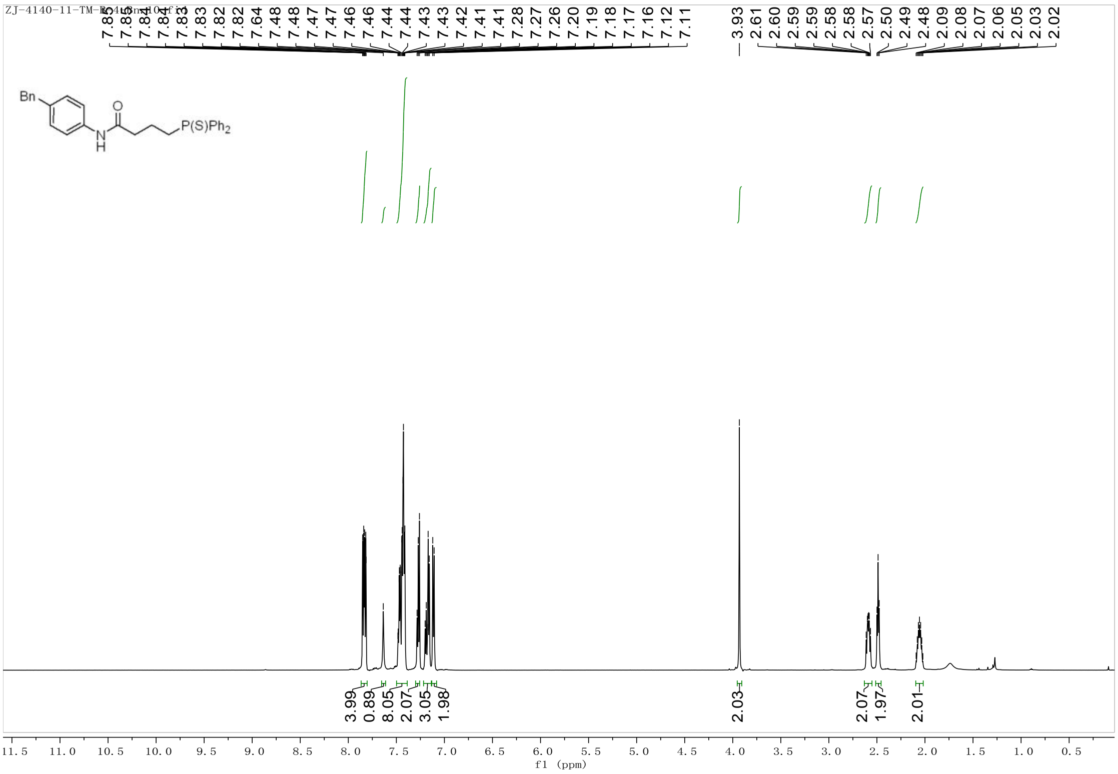

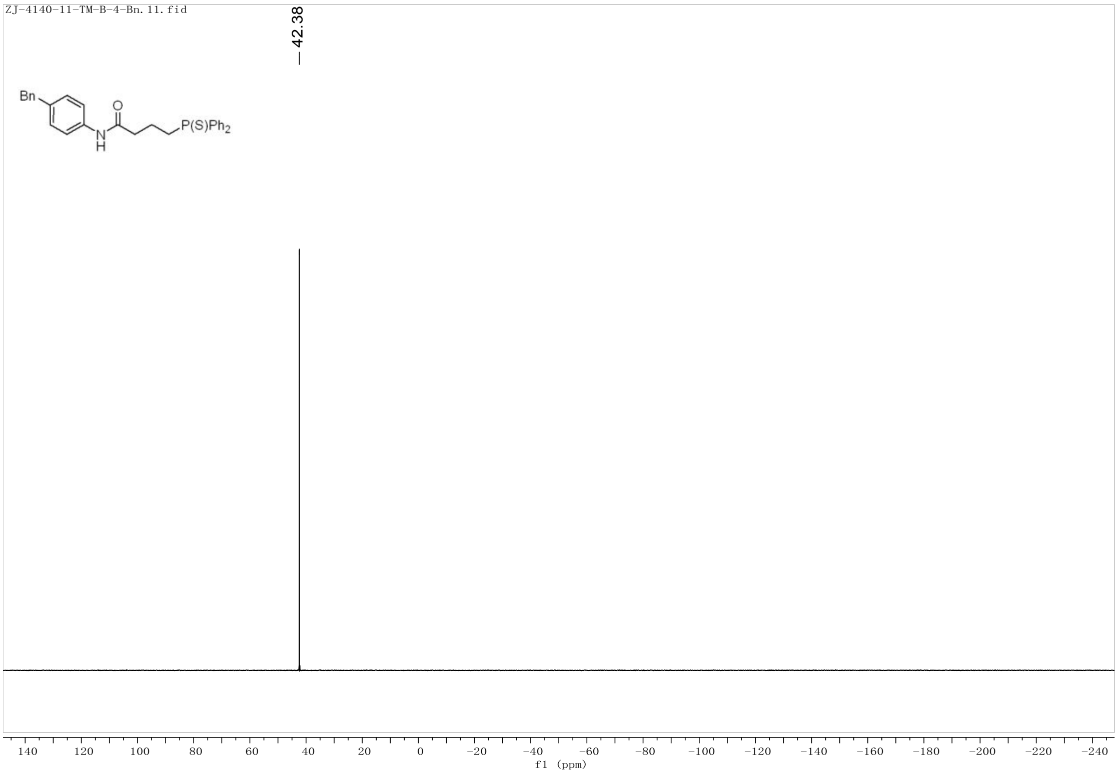
ZJ-4140-11-TM-B-4-Bn.11.fid
42.38
Bn
O
N
H
P(S)Ph2
f1 (ppm)

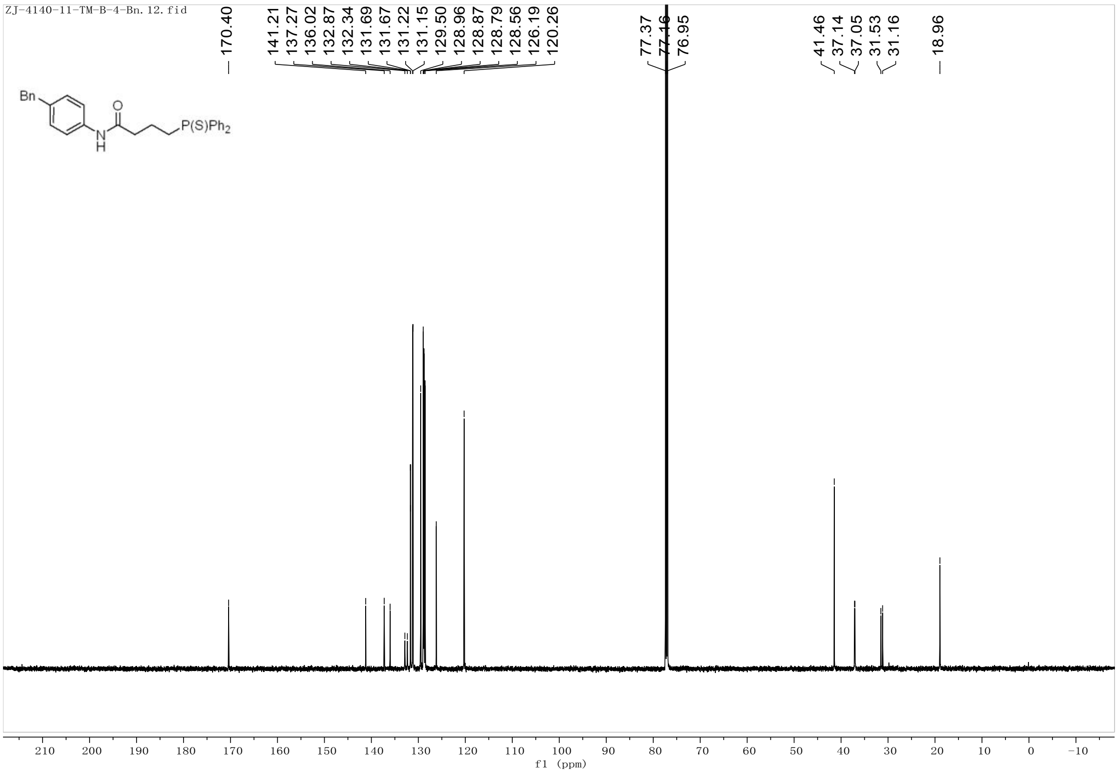
ZJ-4140-11-TM-B-4-Bn.12.fid
170.40
141.21
137.27
136.02
132.87
132.34
131.69
131.67
131.22
131.15
129.50
128.96
128.87
128.79
128.56
126.19
120.26
77.37
77.16
76.95
41.46
37.14
37.05
31.53
31.16
18.96
Bn
O
N
H
P(S)Ph2
f1 (ppm)

4-(diphenylphosphorothioyl)-N-(4-(trifluoromethyl)phenyl)butanamide (4g)

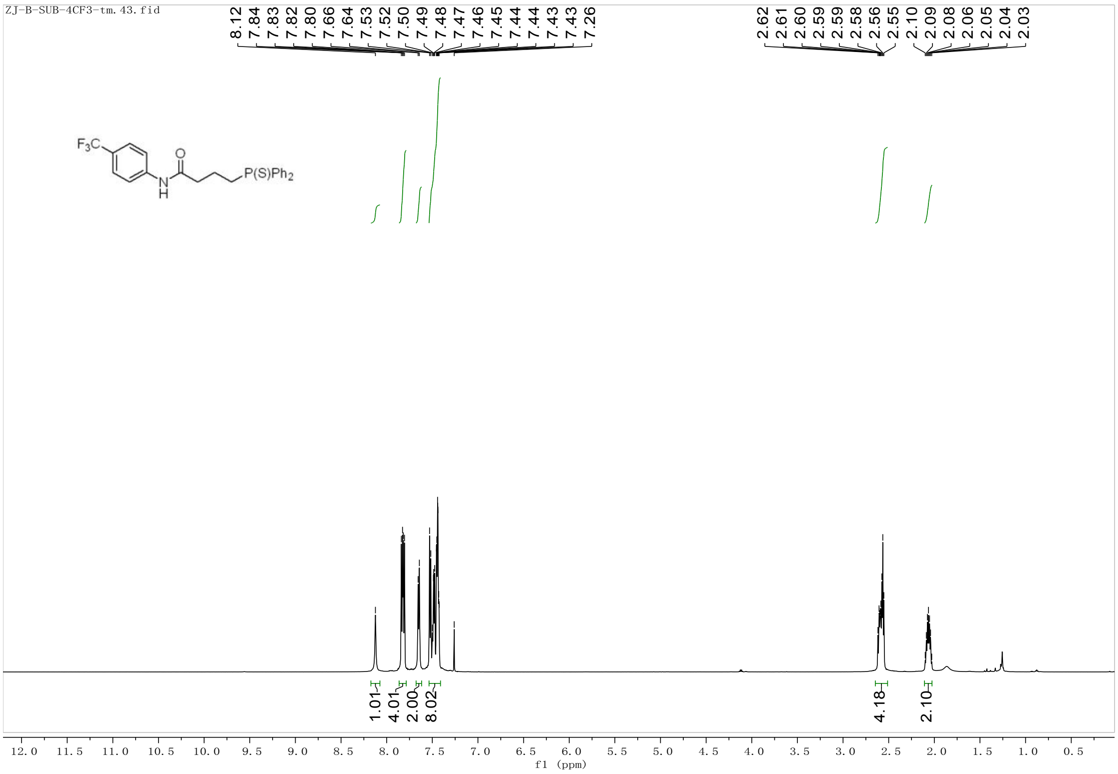

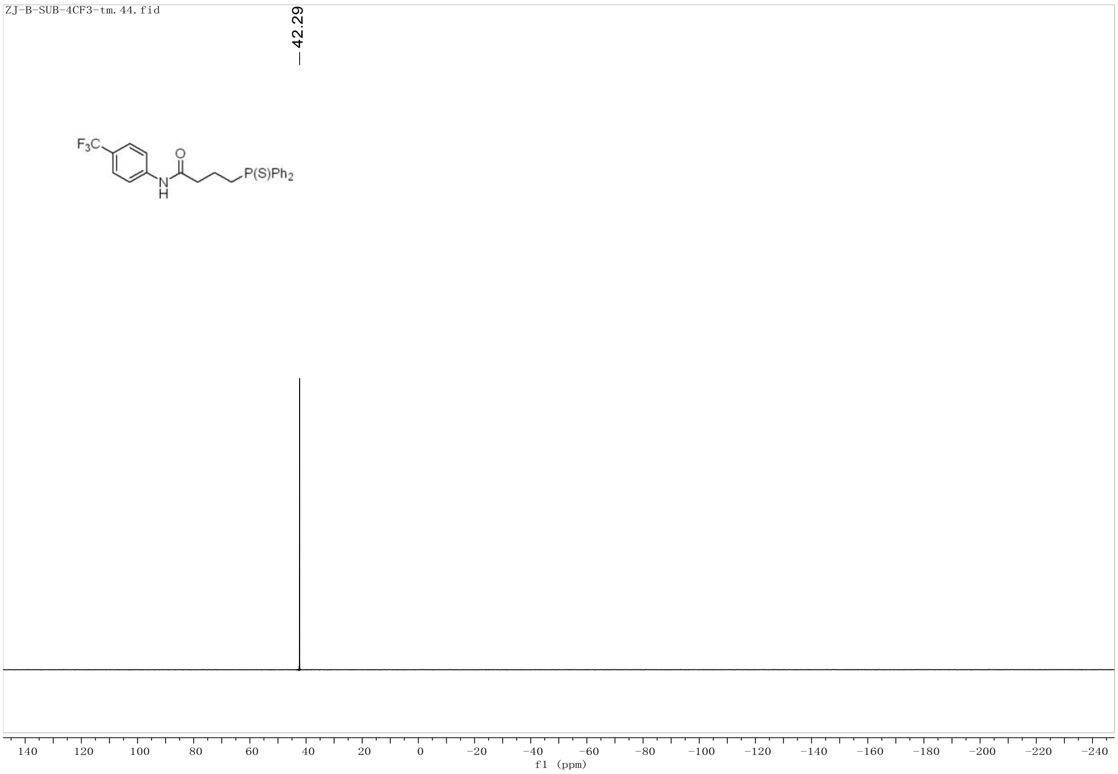

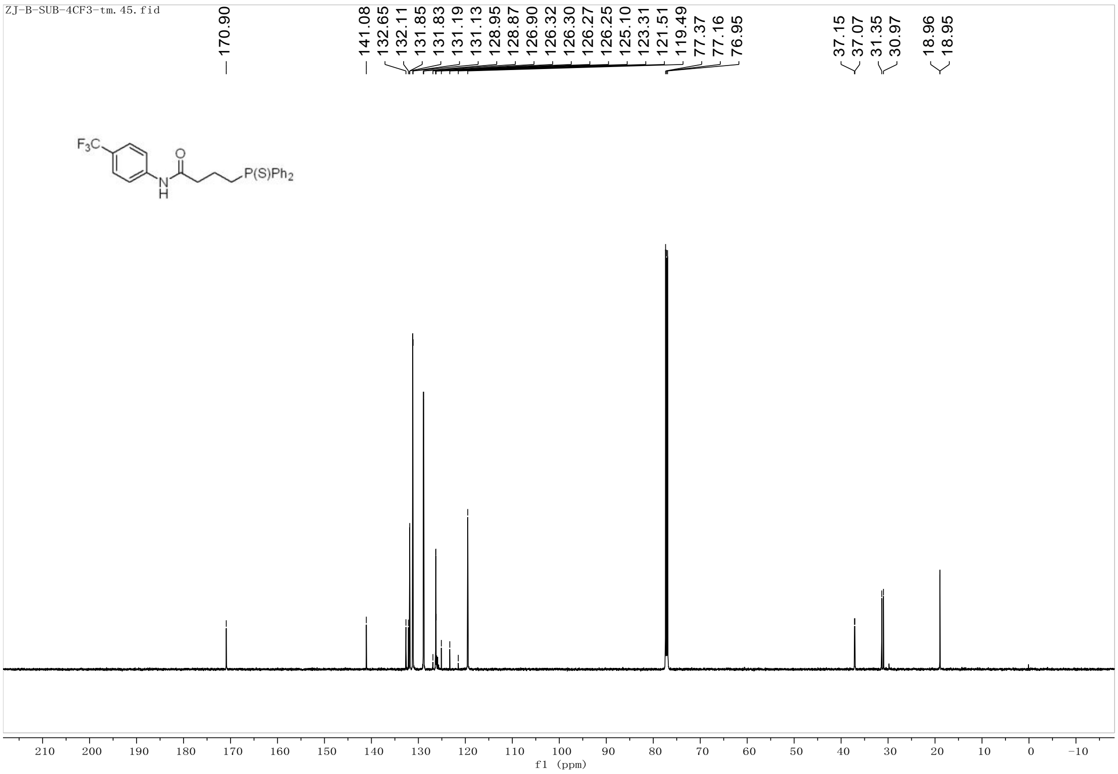
ZJ-B-SUB-4CF3-tm.45.fid
170.90
141.08
132.65
132.11
131.85
131.83
131.19
131.13
128.95
128.87
126.90
126.32
126.30
126.27
126.25
125.10
123.31
121.51
119.49
77.37
77.16
76.95
37.15
37.07
31.35
30.97
18.96
18.95
F_3C
O
N
H
P(S)Ph_2
f1 (ppm)

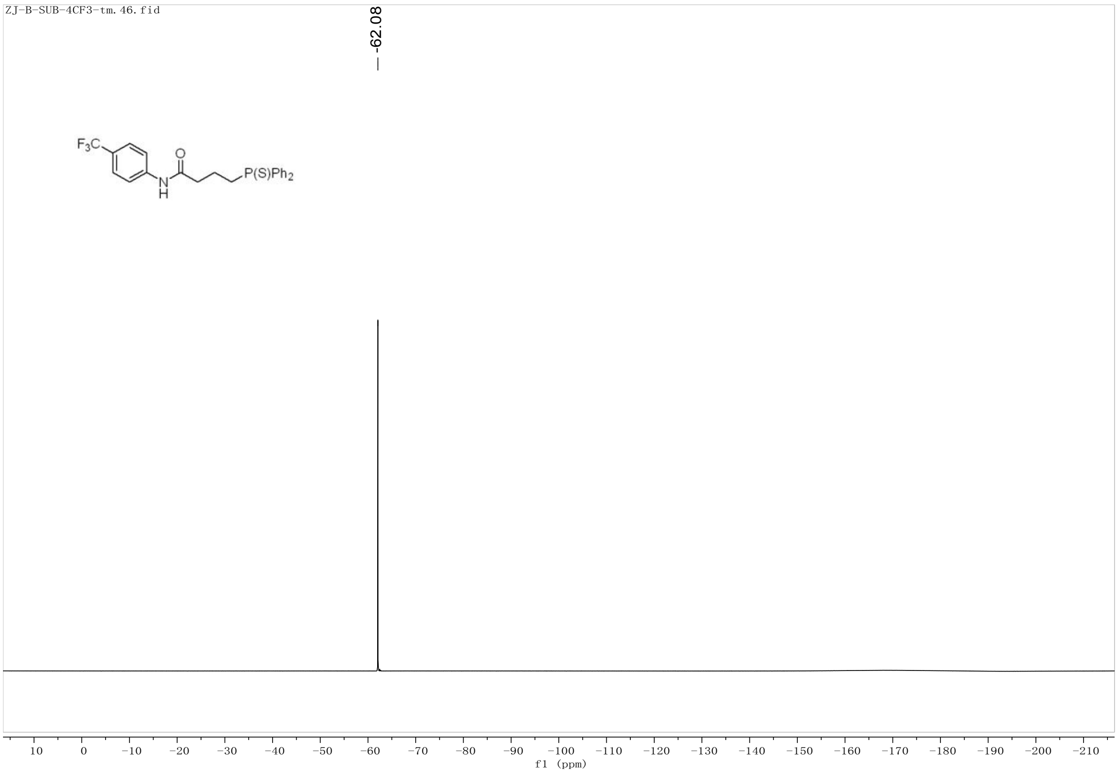
ZJ-B-SUB-4CF3-tm.46.fid
-62.08
F_3C
O
N
H
P(S)Ph_2
f1 (ppm)

4-(diphenylphosphorothioyl)-N-(4-fluorophenyl)butanamide (4h)

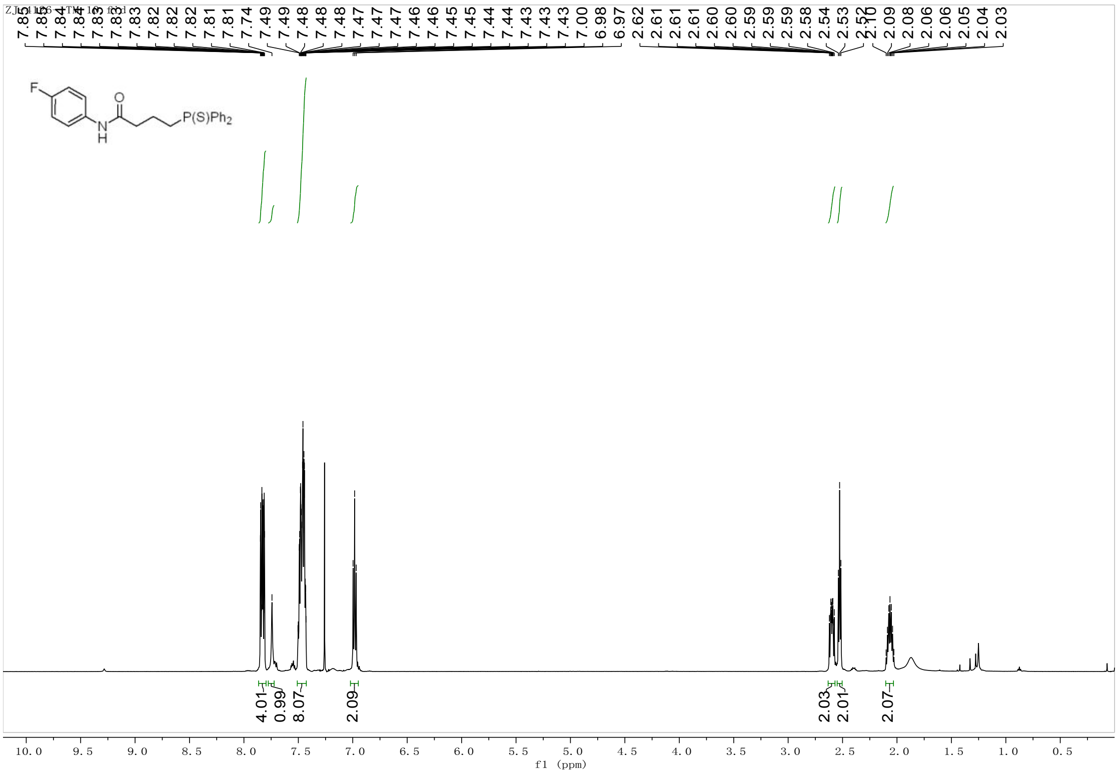

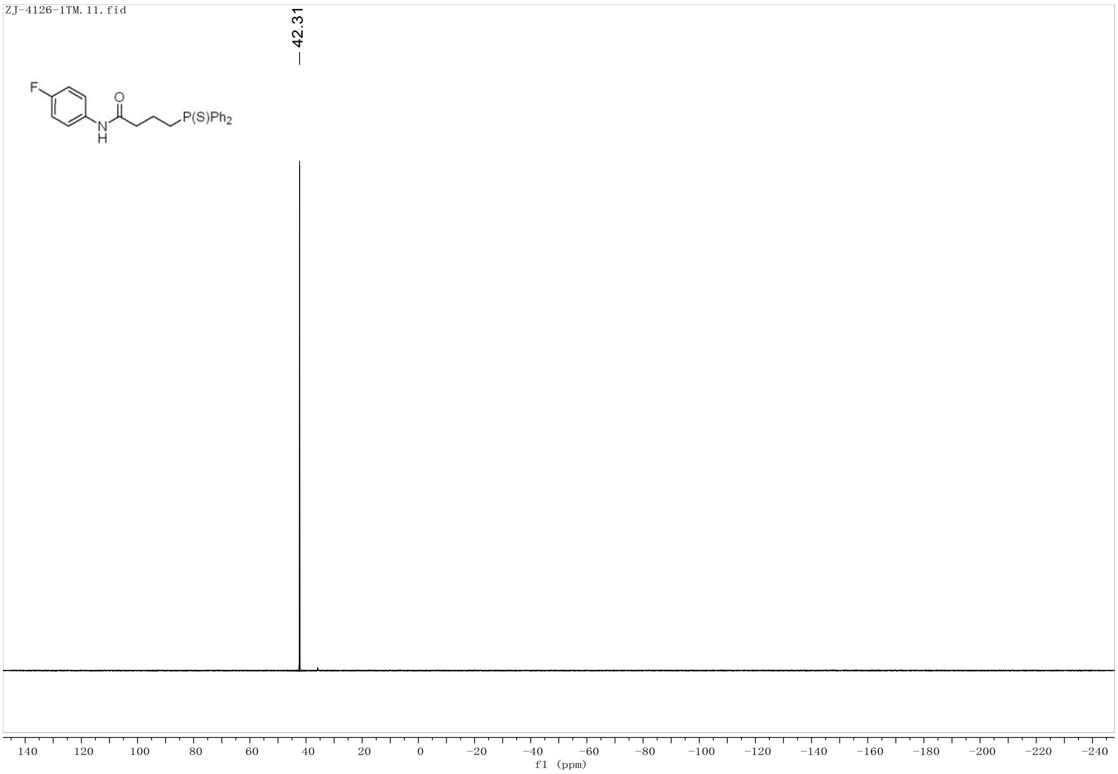

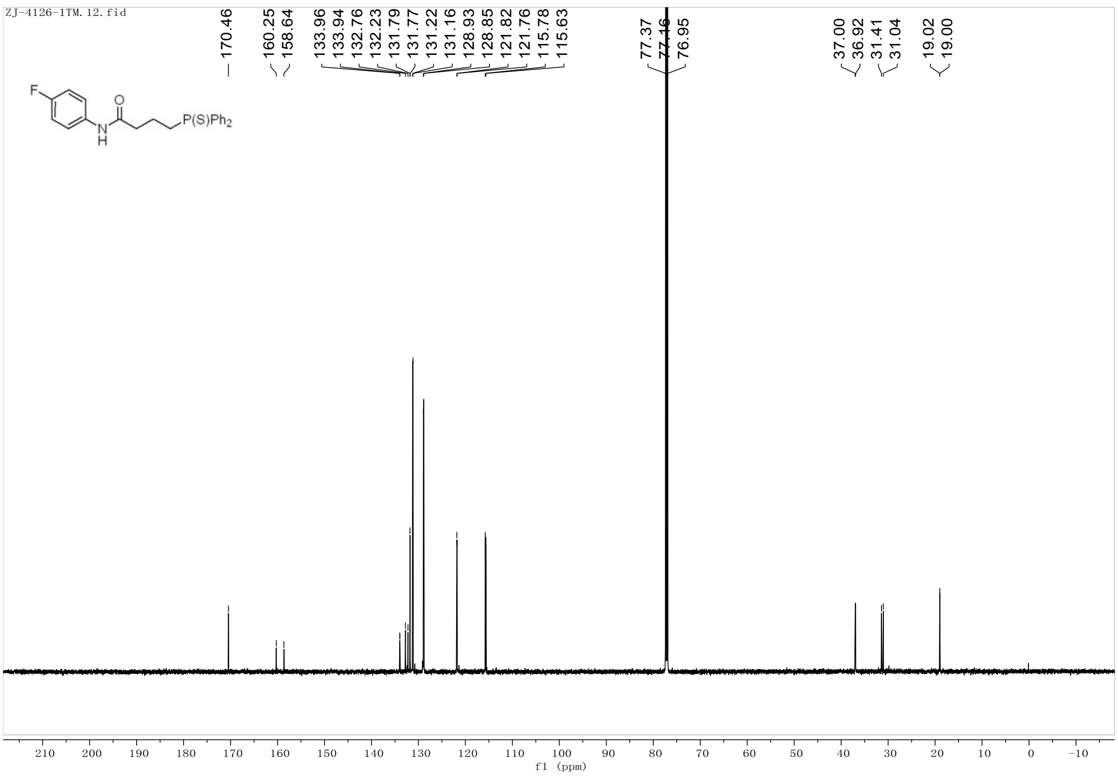
ZJ-4126-1TM.12.fid
170.46
160.25
158.64
133.96
133.94
132.76
132.23
131.79
131.77
131.22
131.16
128.93
128.85
121.82
121.76
115.78
115.63
77.37
77.16
76.95
37.00
36.92
31.41
31.04
19.02
19.00
F
O
N
H
P(S)Ph2
f1 (ppm)

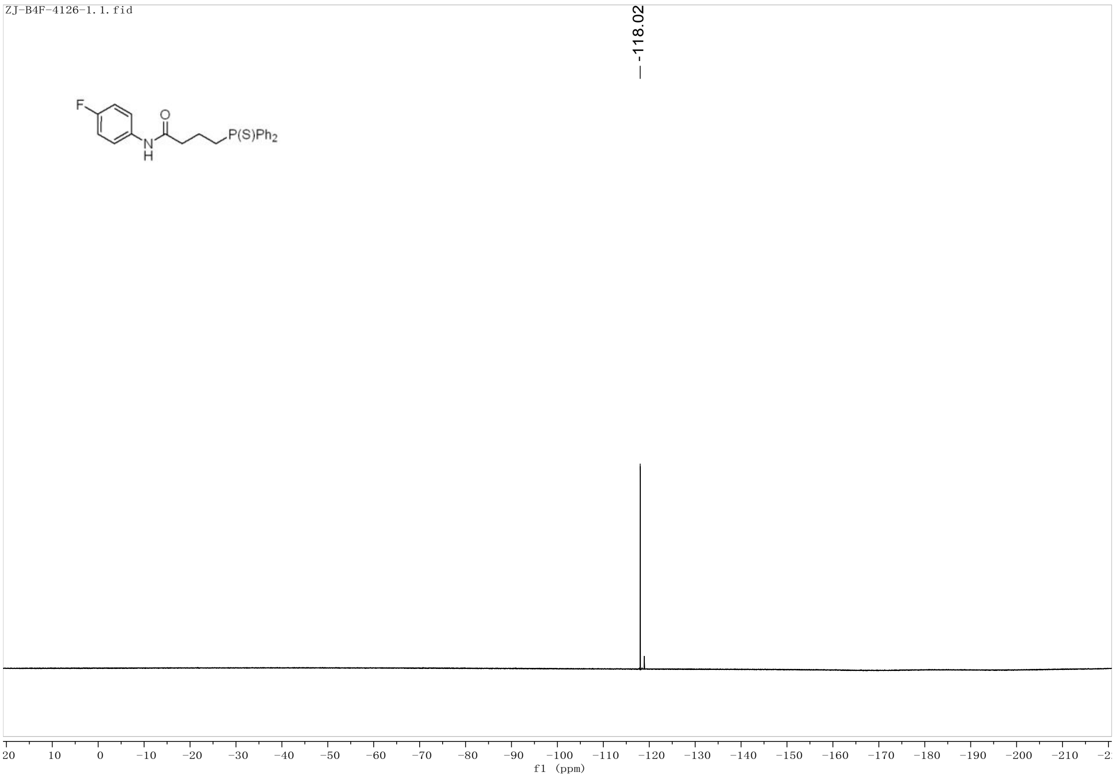
ZJ-B4F-4126-1.1.fid
-118.02
F
O
N
H
P(S)Ph2
f1 (ppm)

N-(4-chlorophenyl)-4-(diphenylphosphorothioyl)butanamide (4i)

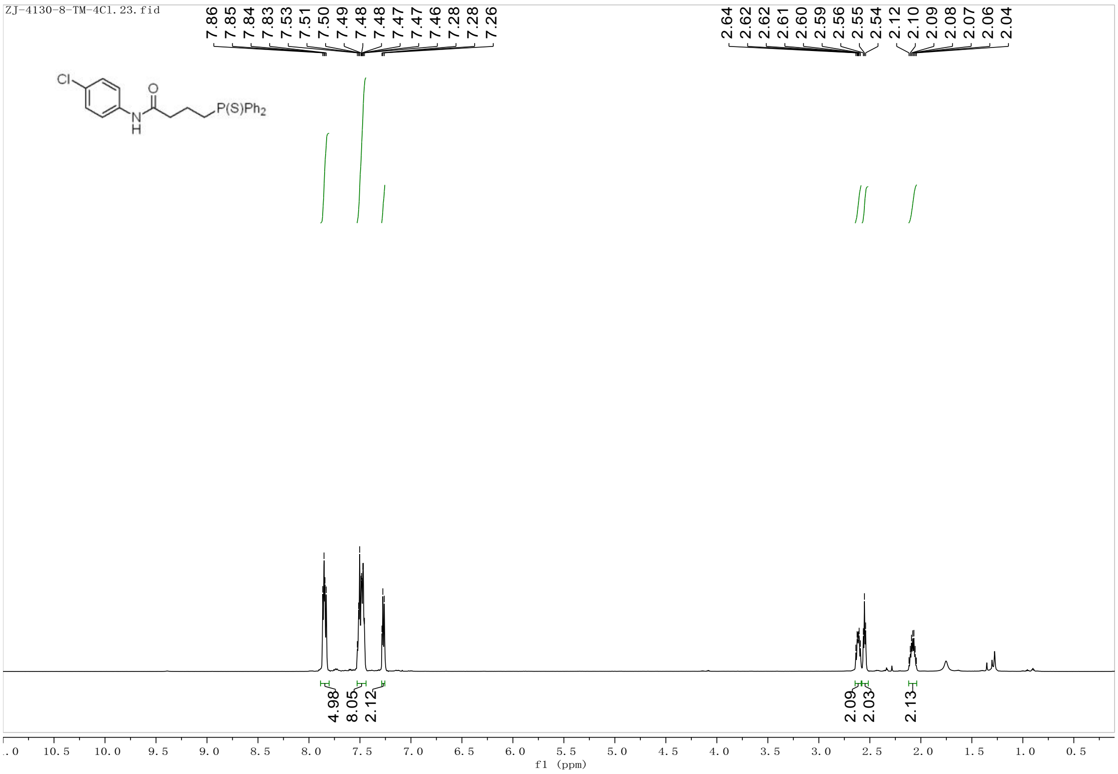

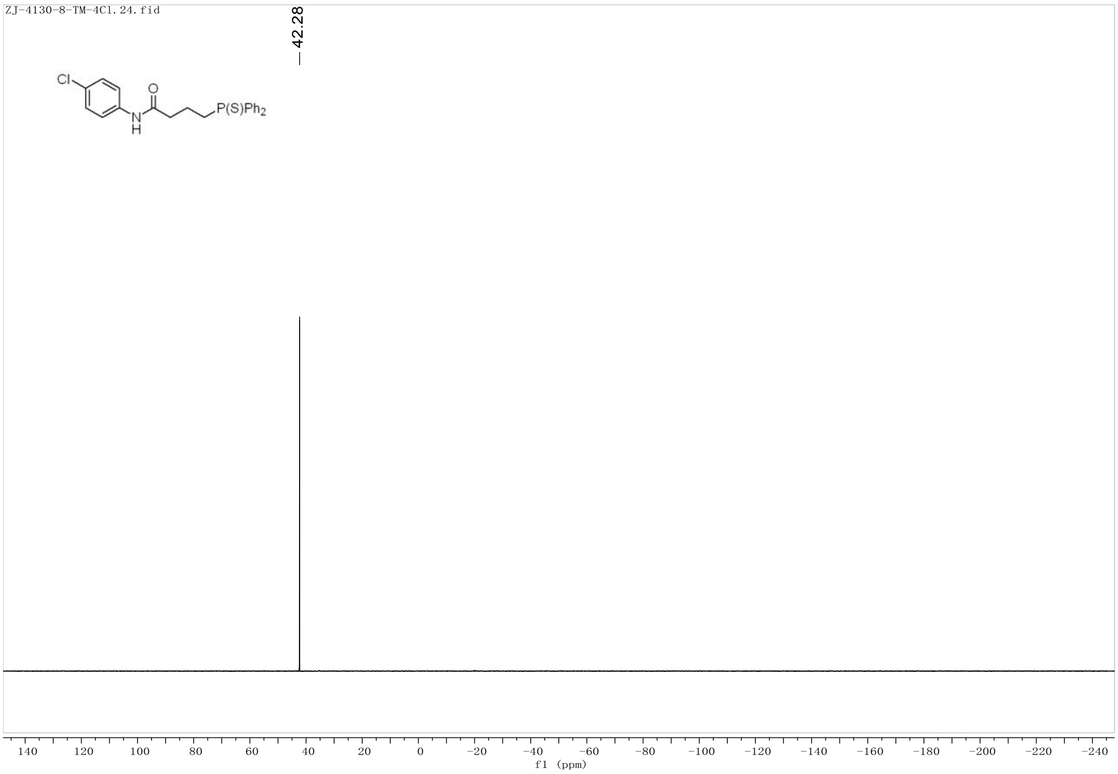

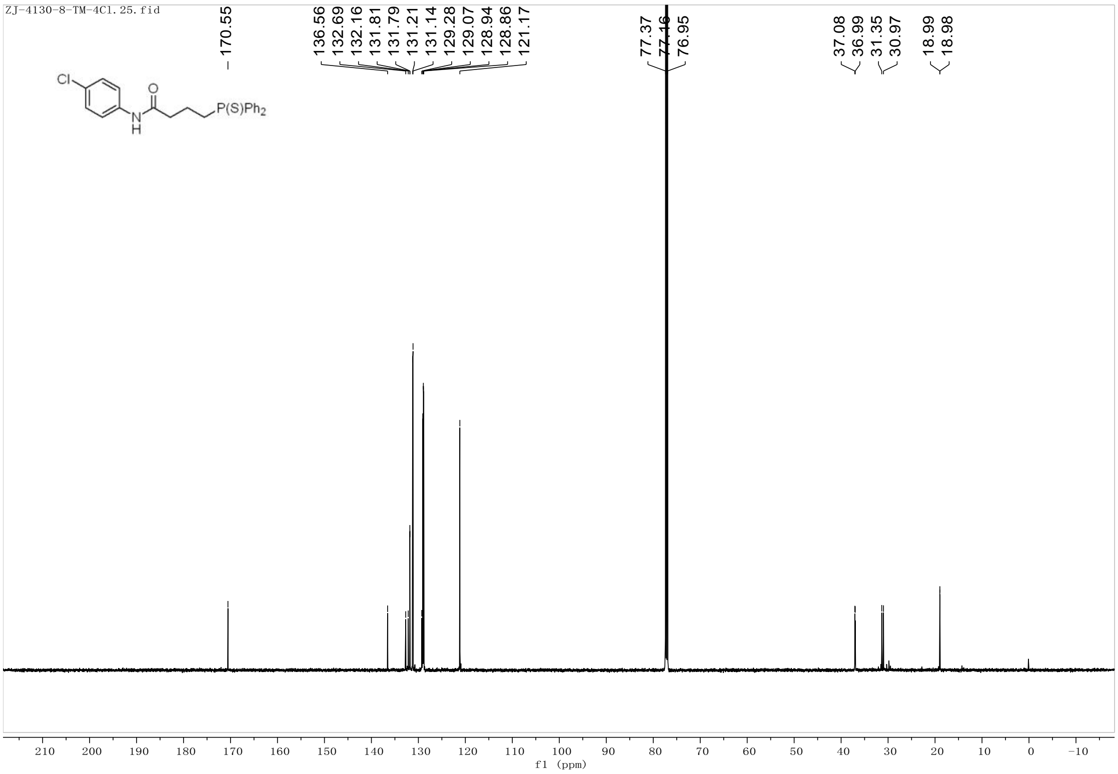

N-(4-bromophenyl)-4-(diphenylphosphorothioyl)butanamide (4j)

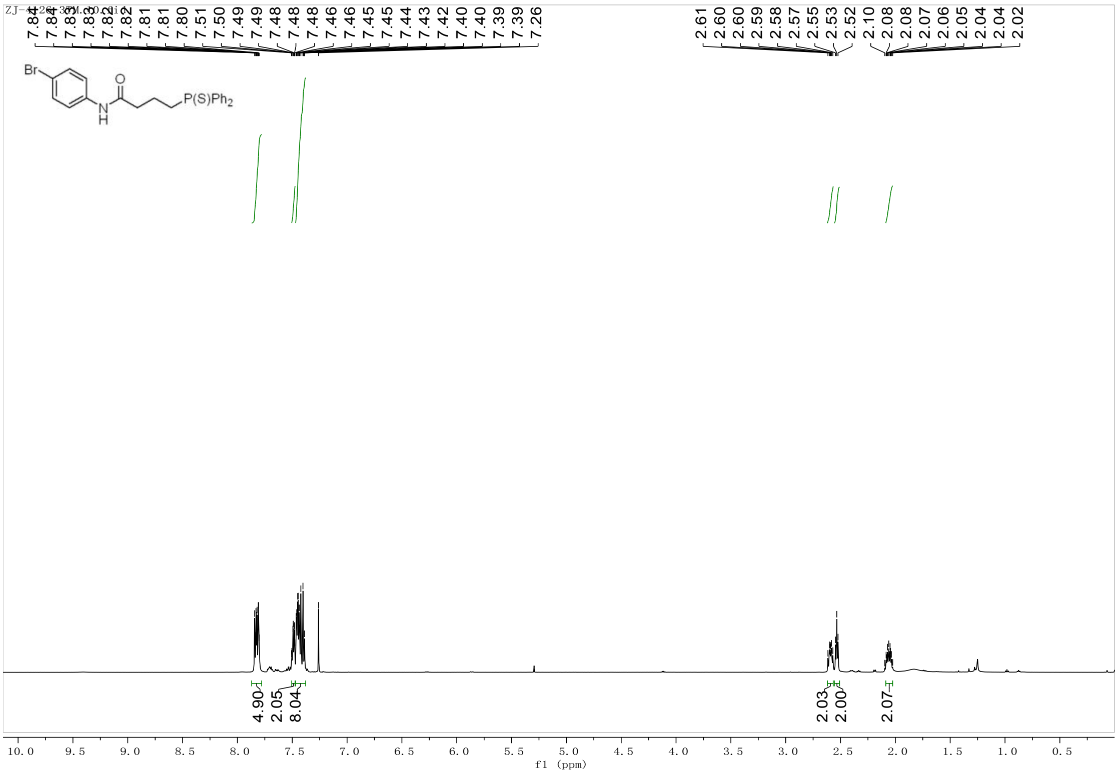

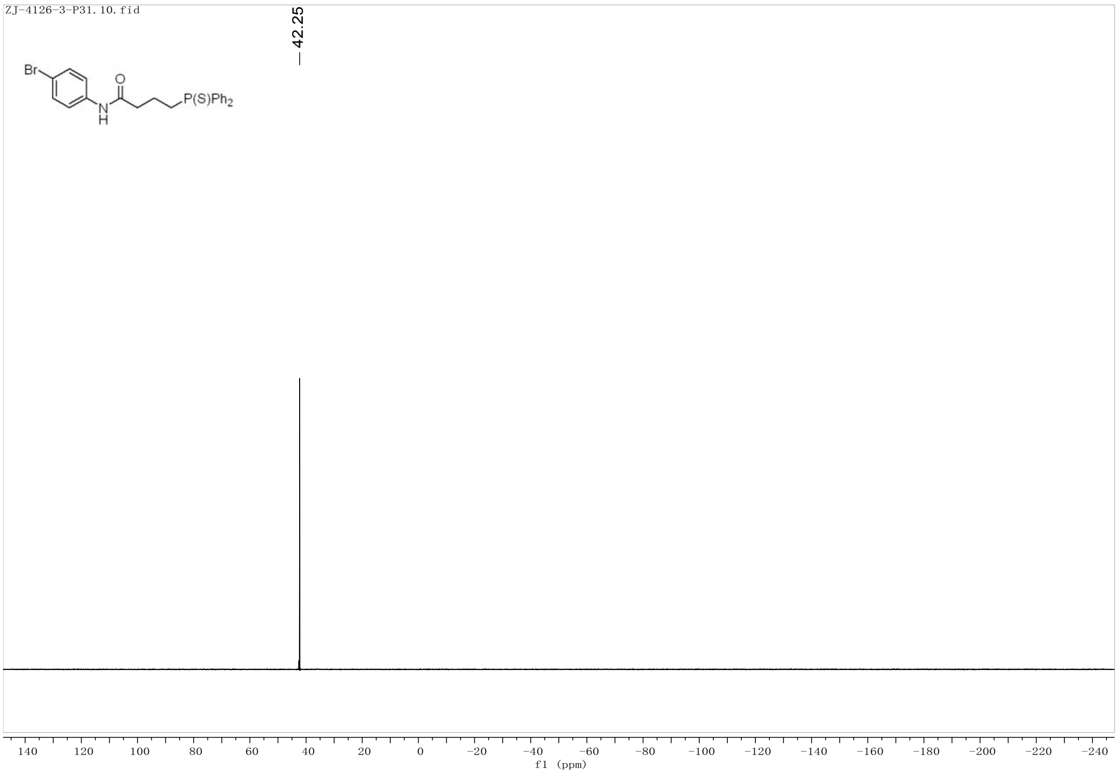
ZJ-4126-3-P31.10.fid
42.25
Br
O
N
H
P(S)Ph2
140
120
100
80
60
40
20
0
−20
−40
−60
−80
−100
−120
−140
−160
−180
−200
−220
−240
f1 (ppm)

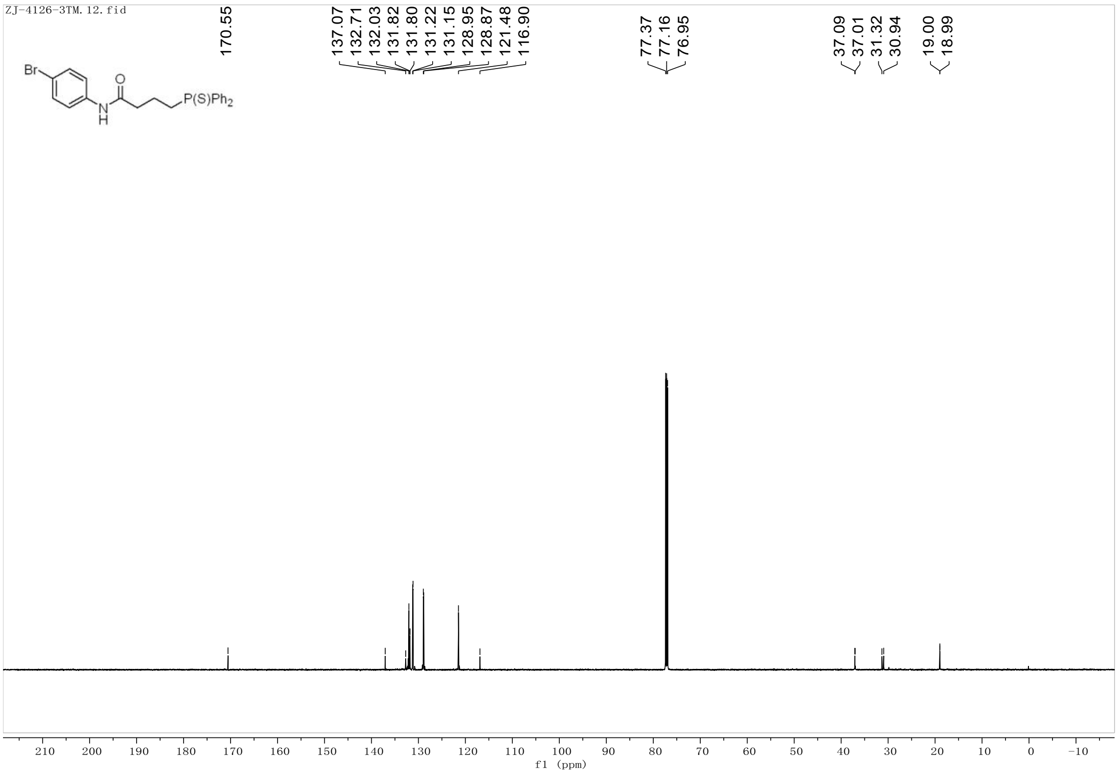
ZJ-4126-3TM.12.fid
170.55
137.07
132.71
132.03
131.82
131.80
131.22
131.15
128.95
128.87
121.48
116.90
77.37
77.16
76.95
37.09
37.01
31.32
30.94
19.00
18.99
Br
O
N
H
P(S)Ph2
210
200
190
180
170
160
150
140
130
120
110
100
90
80
70
60
50
40
30
20
10
0
−10
f1 (ppm)

4-(diphenylphosphorothioyl)-N-(4-methoxyphenyl)butanamide (4k)

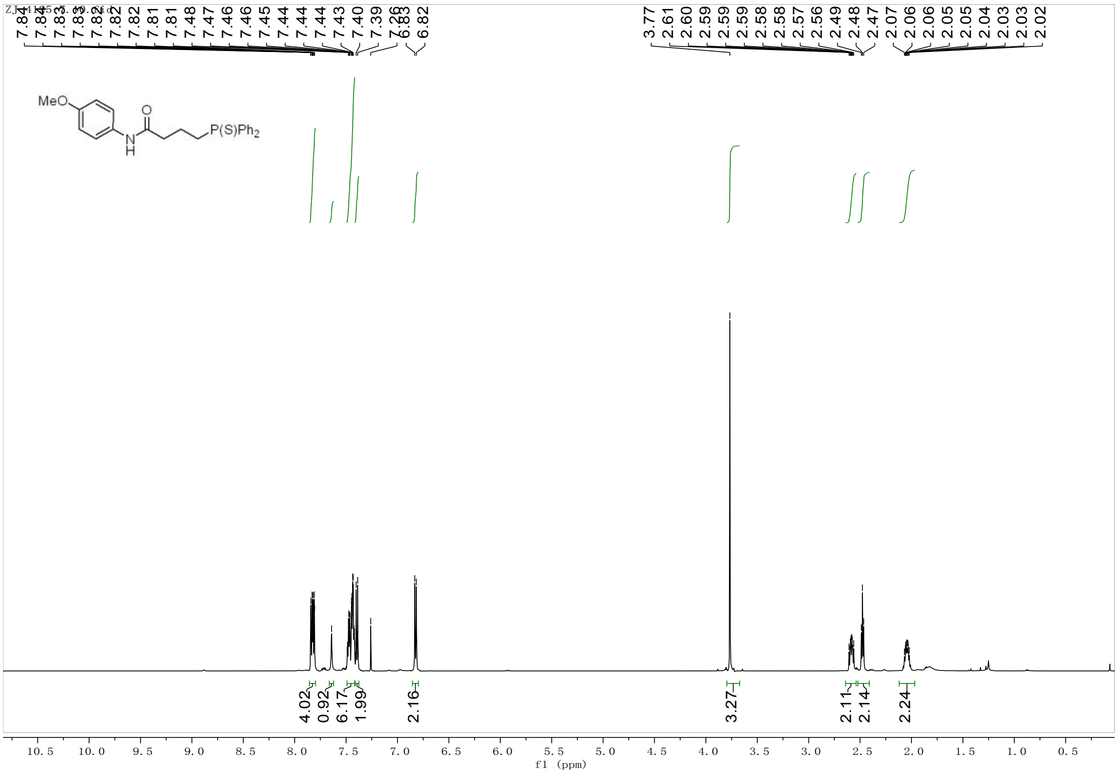

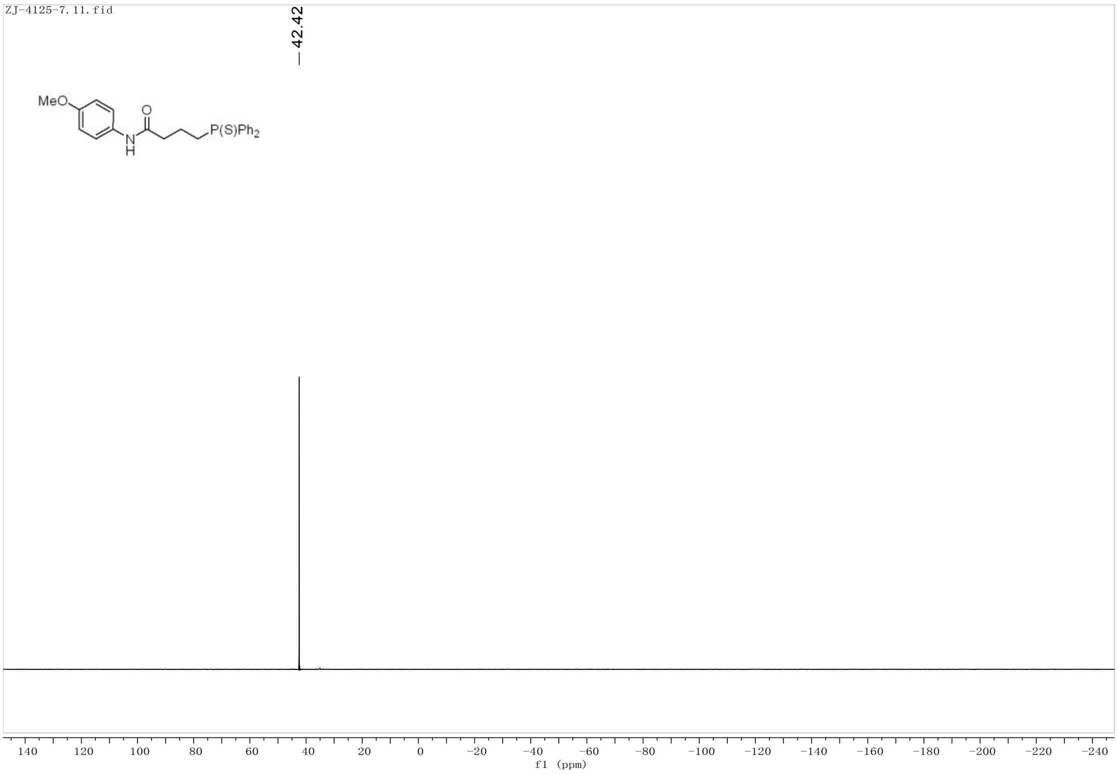

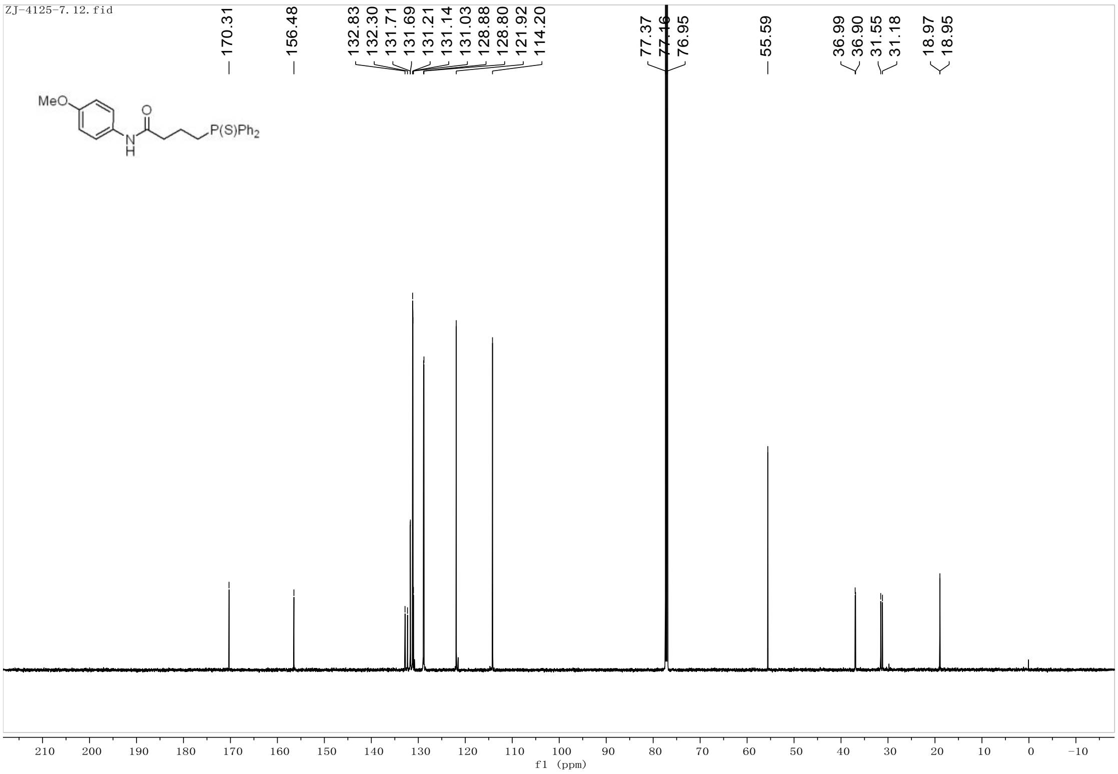

4-(diphenylphosphorothioyl)-N-(4-phenoxyphenyl)butanamide (4l)

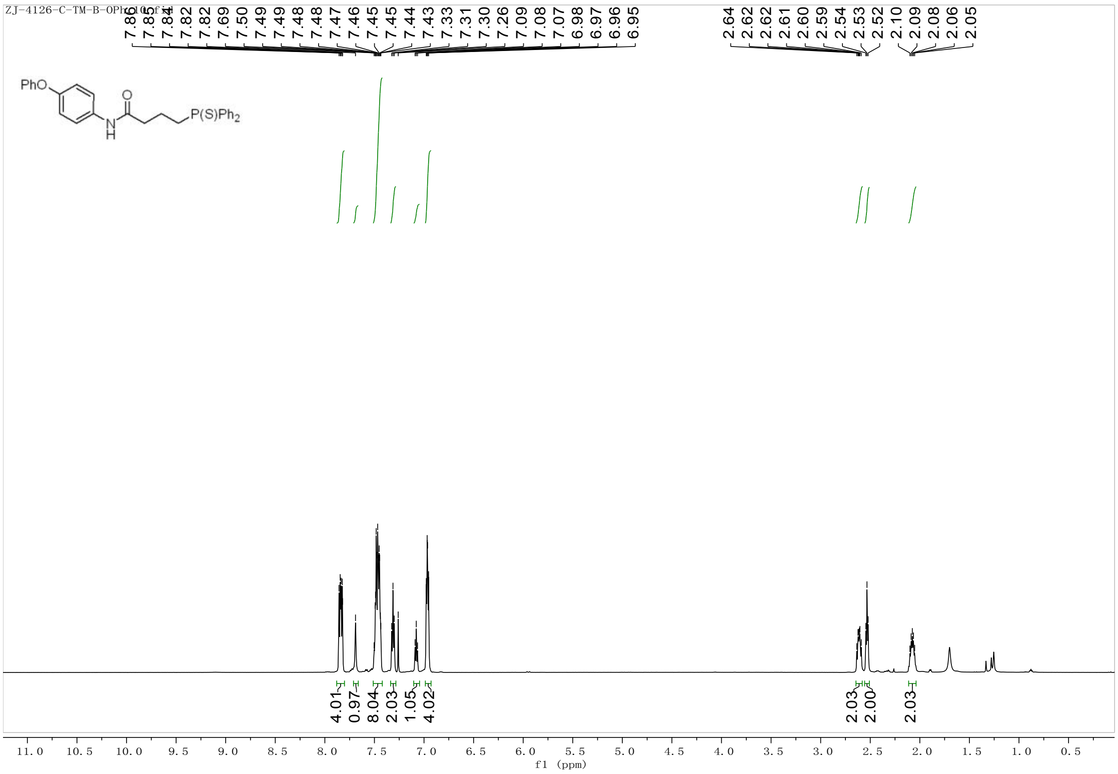

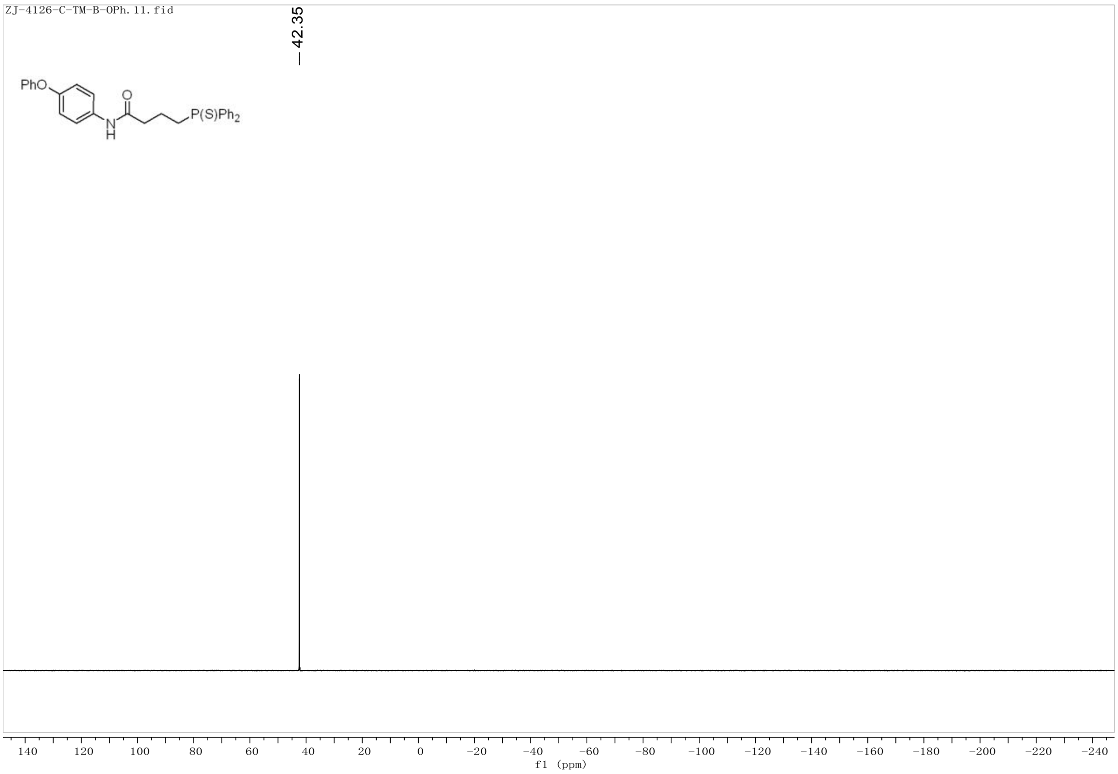
ZJ-4126-C-TM-B-OPh.11.fid
42.35
PhO
O
N
H
P(S)Ph2
140
120
100
80
60
40
20
0
−20
−40
−60
−80
−100
−120
−140
−160
−180
−200
−220
−240
f1 (ppm)

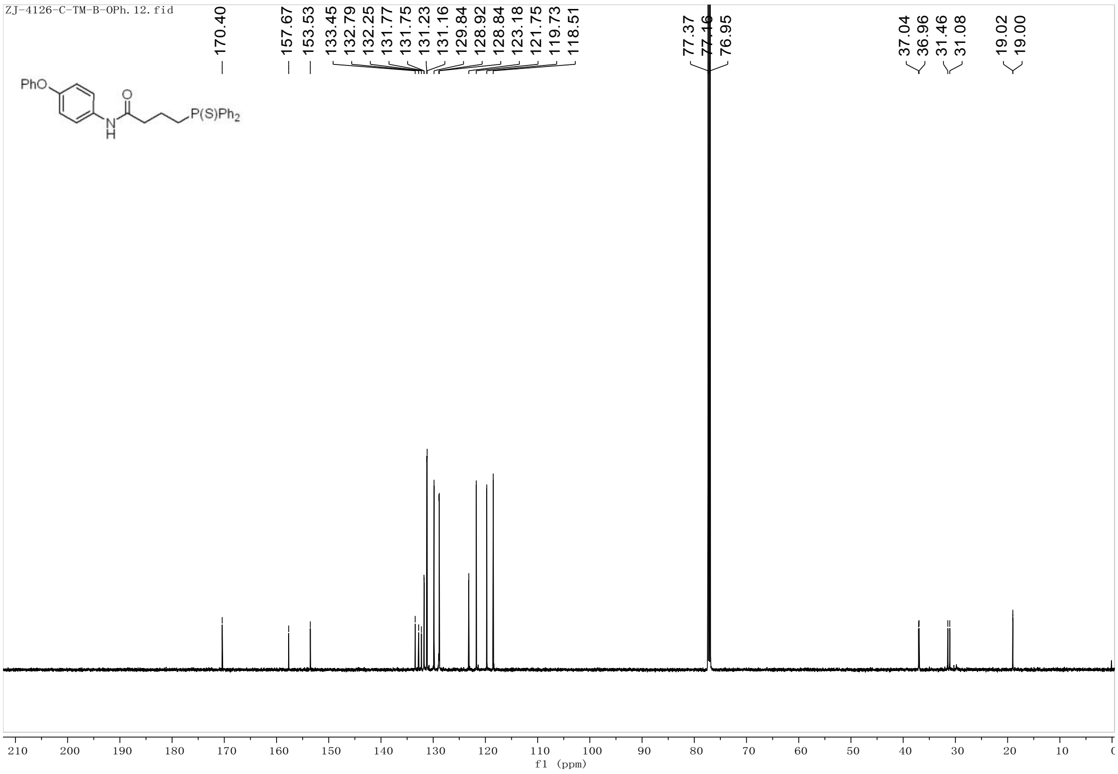
ZJ-4126-C-TM-B-OPh.12.fid
170.40
157.67
153.53
133.45
132.79
132.25
131.77
131.75
131.23
131.16
129.84
128.92
128.84
123.18
121.75
119.73
118.51
77.37
77.16
76.95
37.04
36.96
31.46
31.08
19.02
19.00
PhO
O
N
H
P(S)Ph2
210
200
190
180
170
160
150
140
130
120
110
100
90
80
70
60
50
40
30
20
10
f1 (ppm)

4-(diphenylphosphorothioyl)-N-(4-(trifluoromethoxy)phenyl)butanamide (4m)

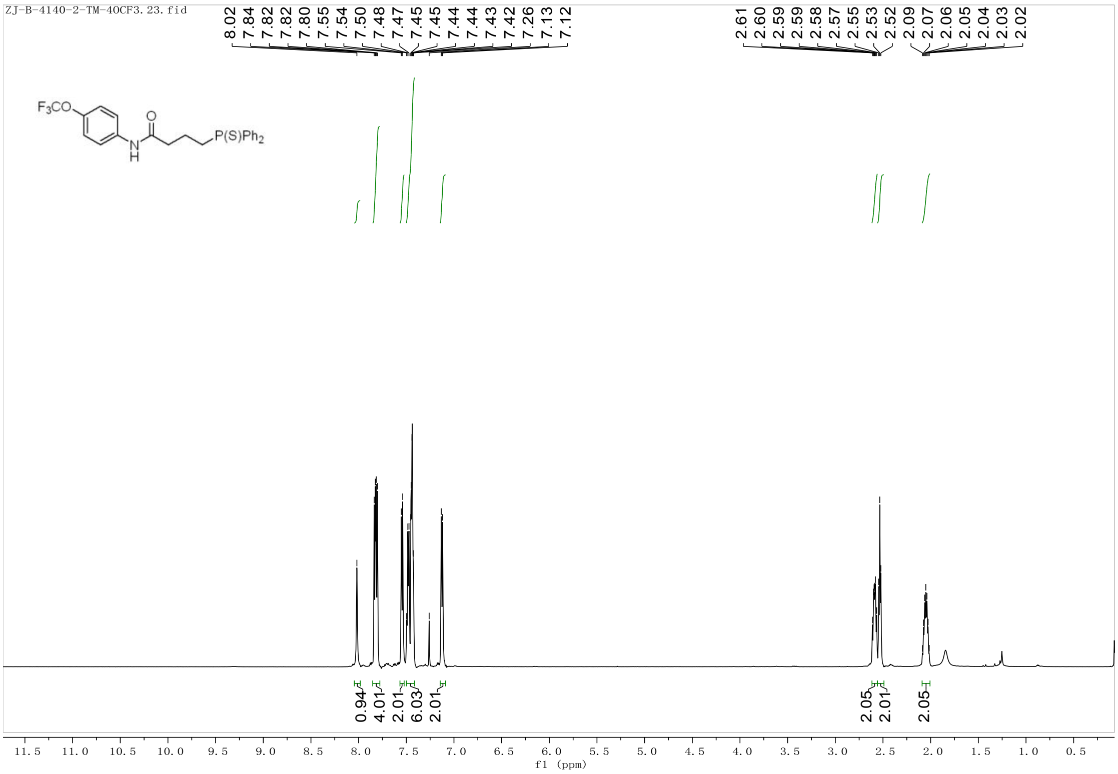

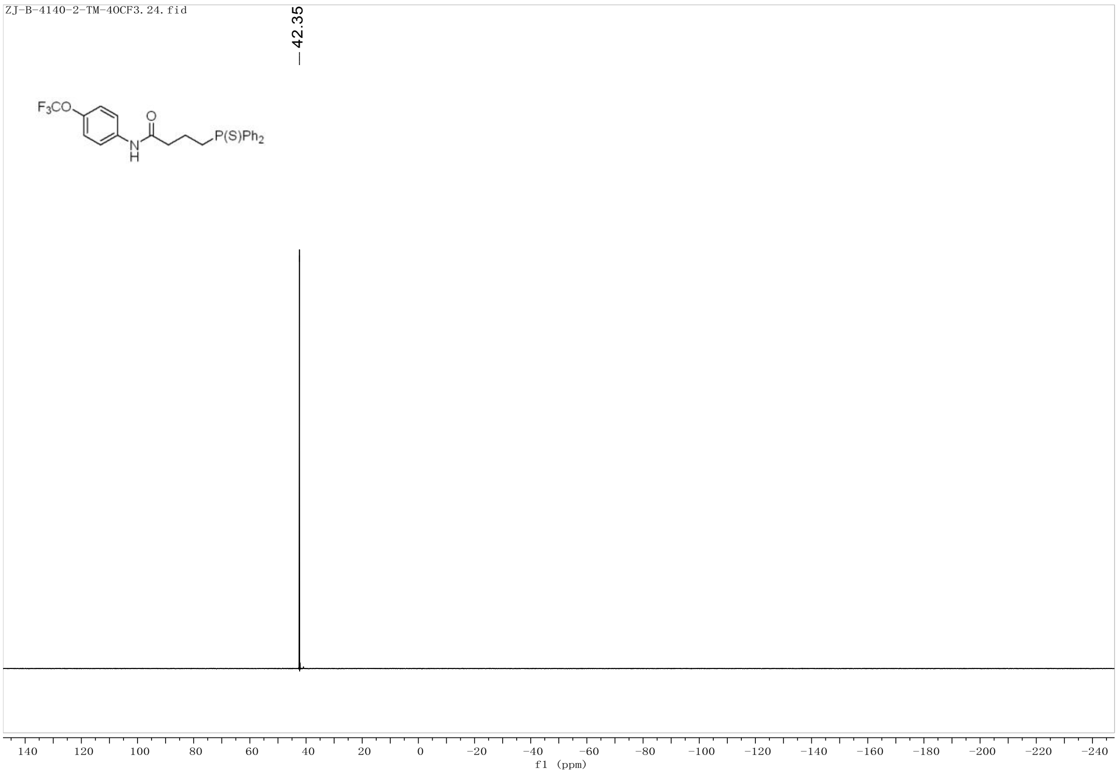

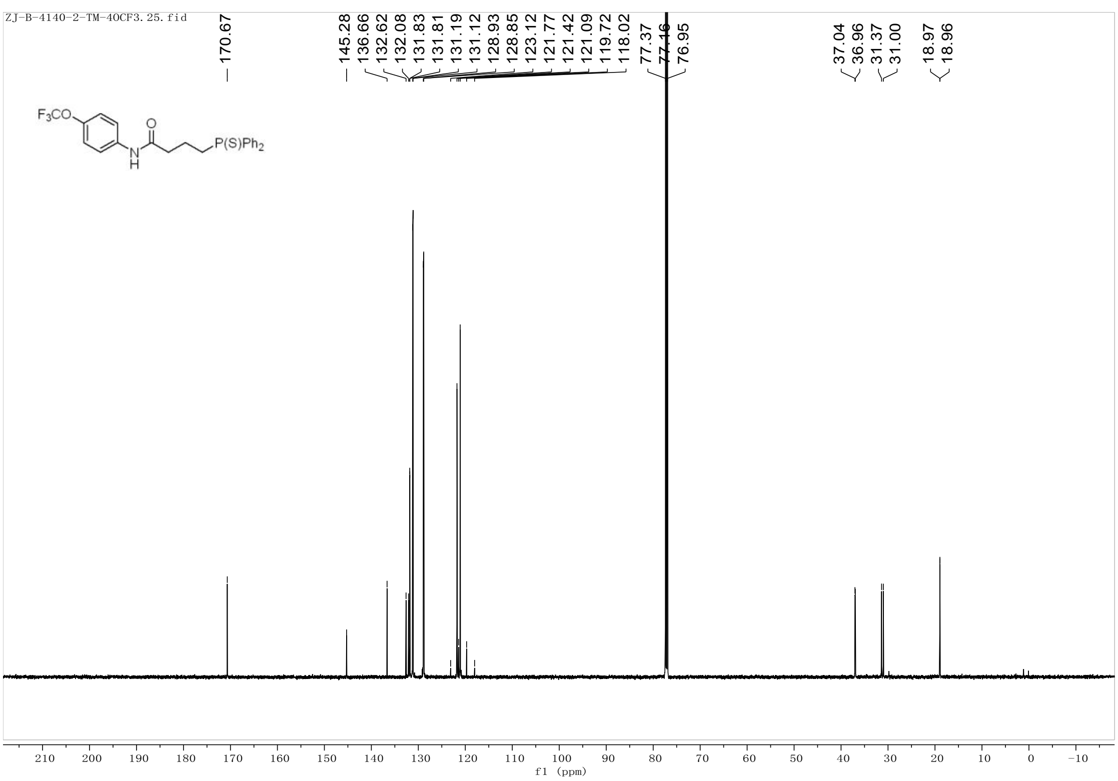
ZJ-B-4140-2-TM-4OCF3.25.fid
170.67
145.28
136.66
132.62
132.08
131.83
131.81
131.19
131.12
128.93
128.85
123.12
121.77
121.42
121.09
119.72
118.02
77.37
77.16
76.95
37.04
36.96
31.37
31.00
18.97
18.96
F3CO
O
N
H
P(S)Ph2
f1 (ppm)

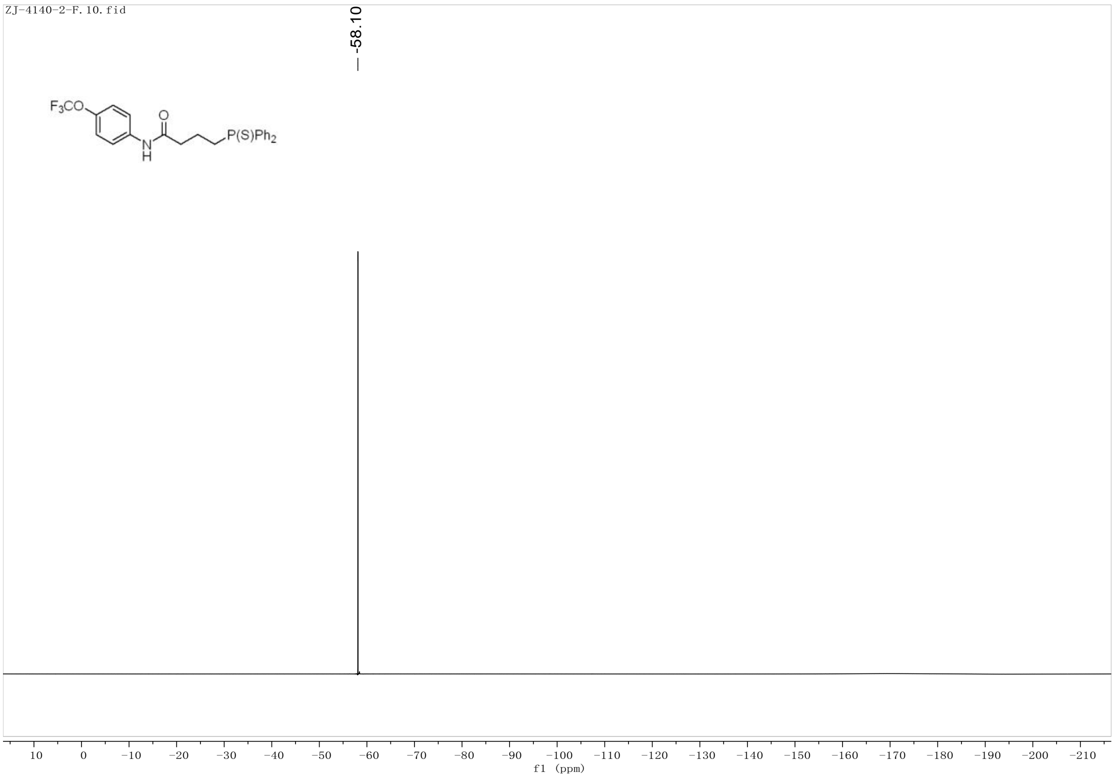
ZJ-4140-2-F.10.fid
-58.10
F3CO
O
N
H
P(S)Ph2
f1 (ppm)

4-(diphenylphosphorothioyl)-N-(4-vinylphenyl)butanamide (4n)

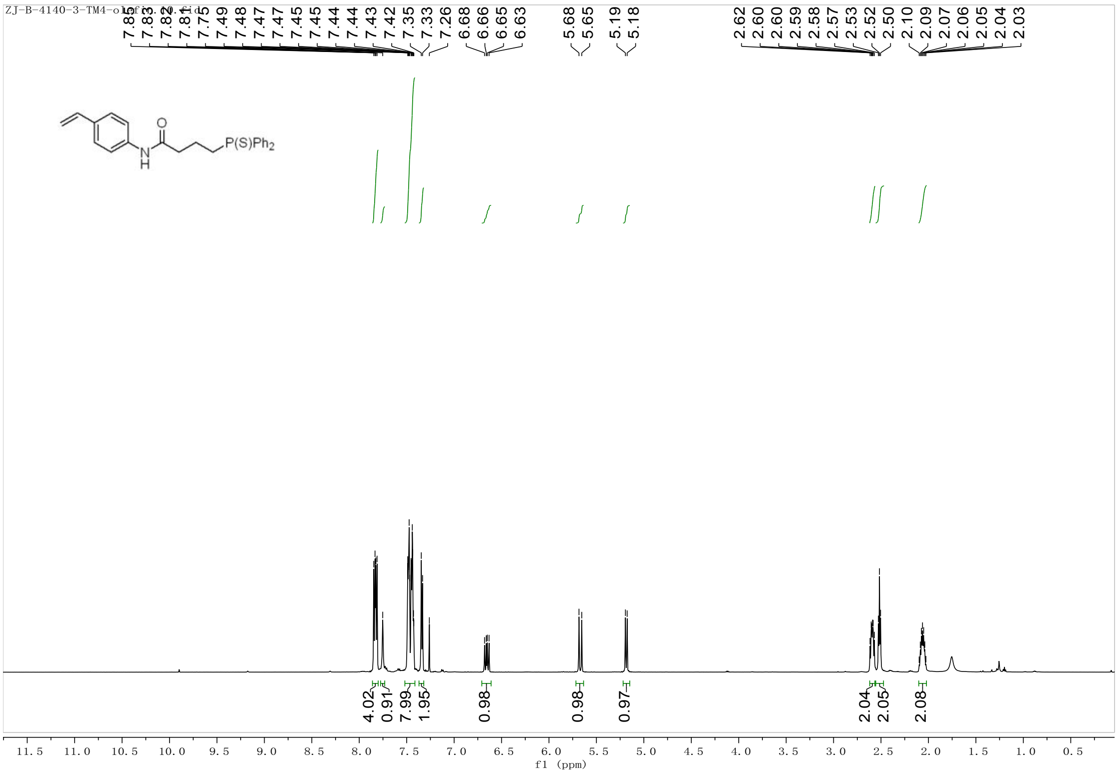

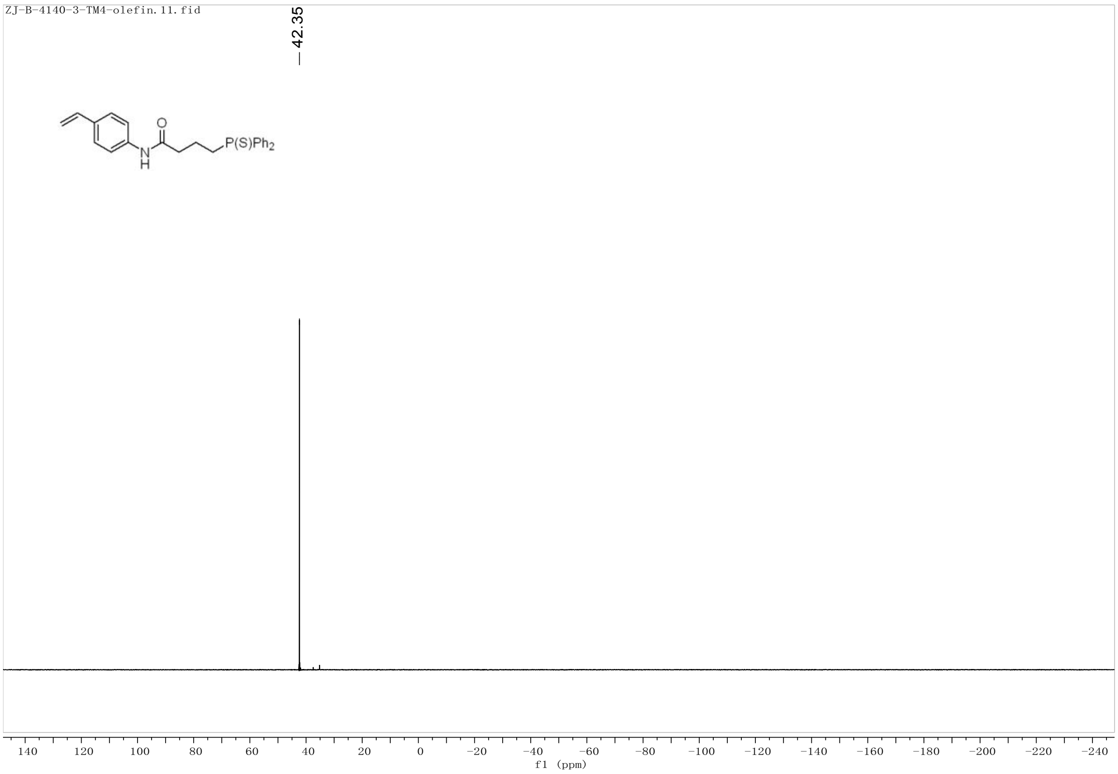

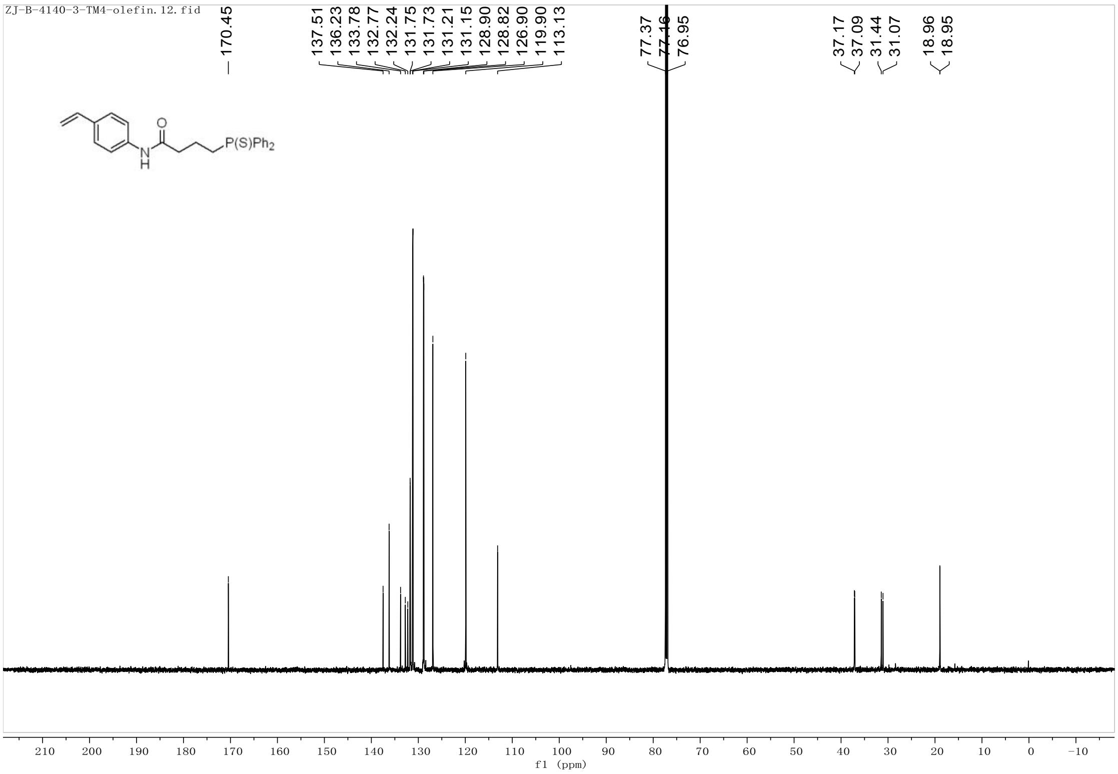

N-(3,5-difluorophenyl)-4-(diphenylphosphorothioyl)butanamide (4o)

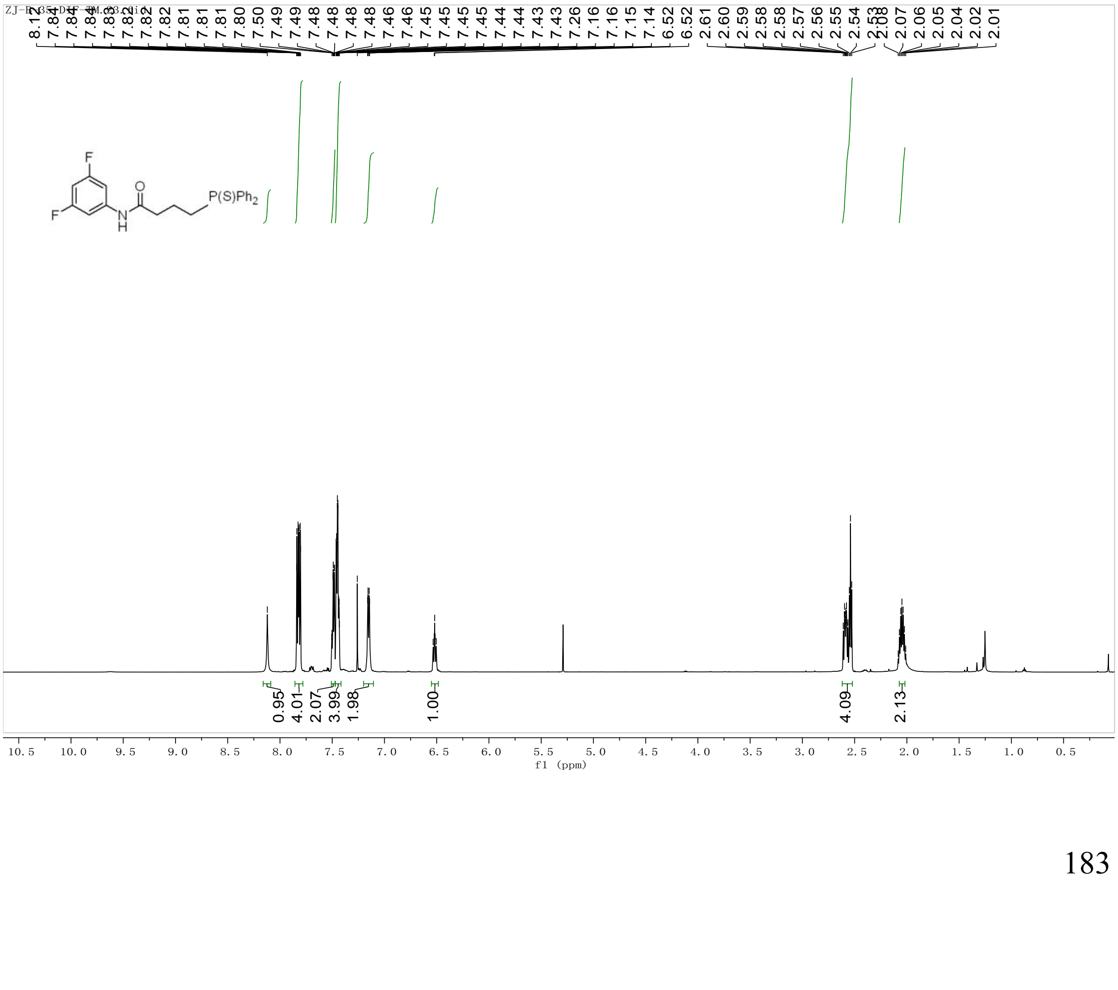

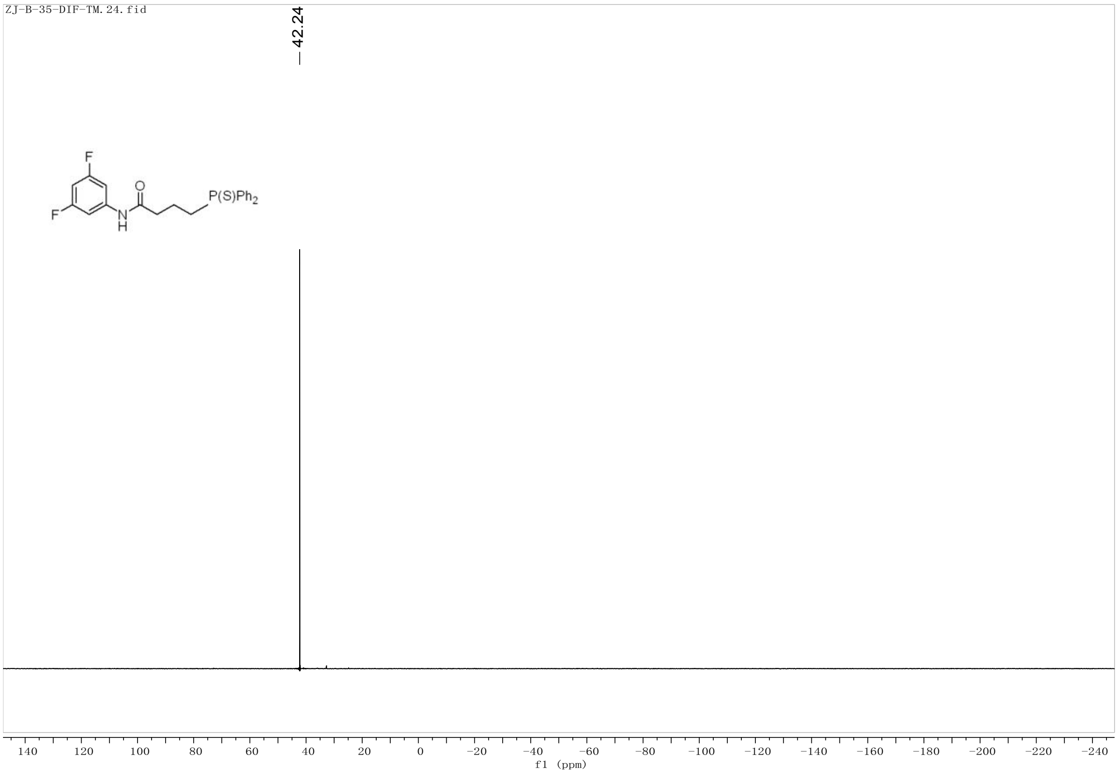
ZJ-B-35-DIF-TM.24.fid
42.24
F
F
O
N
H
P(S)Ph2
140 120 100 80 60 40 20 0 -20 -40 -60 -80 -100 -120 -140 -160 -180 -200 -220 -240
f1 (ppm)

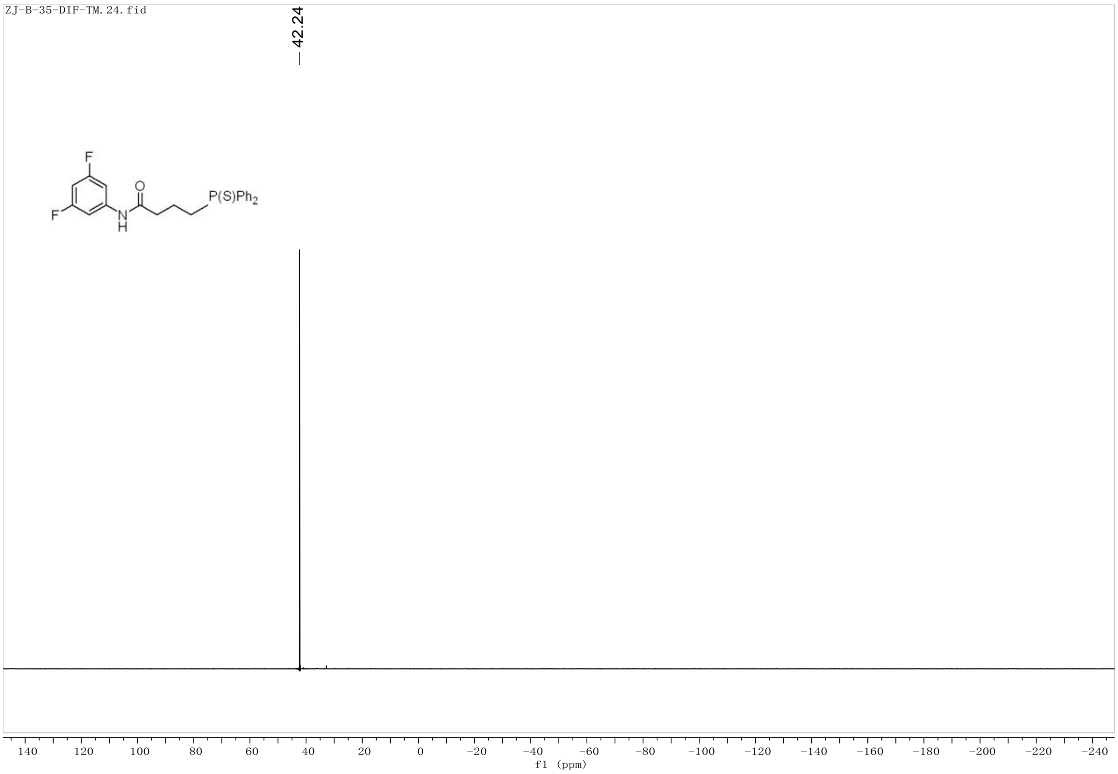
ZJ-B-35-DIF-TM.24.fid
42.24
F
F
O
N
H
P(S)Ph2
140 120 100 80 60 40 20 0 -20 -40 -60 -80 -100 -120 -140 -160 -180 -200 -220 -240
f1 (ppm)

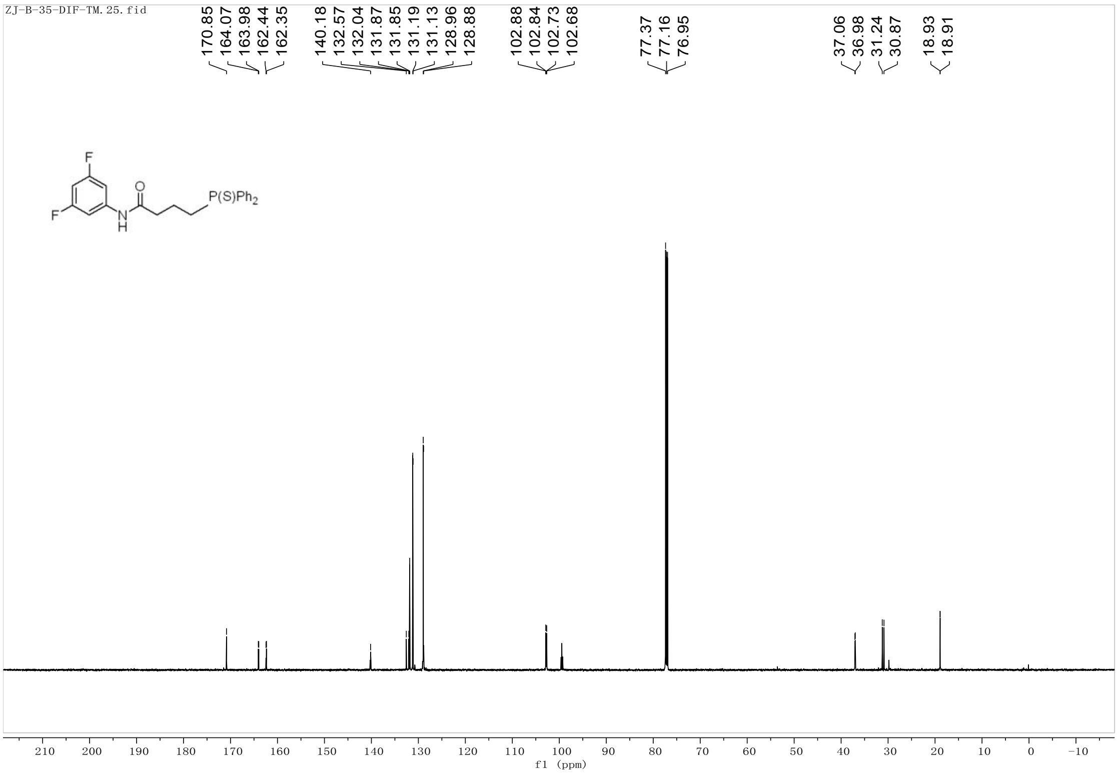

4-(diphenylphosphorothioyl)-N-(4-morpholinophenyl)butanamide (4p)

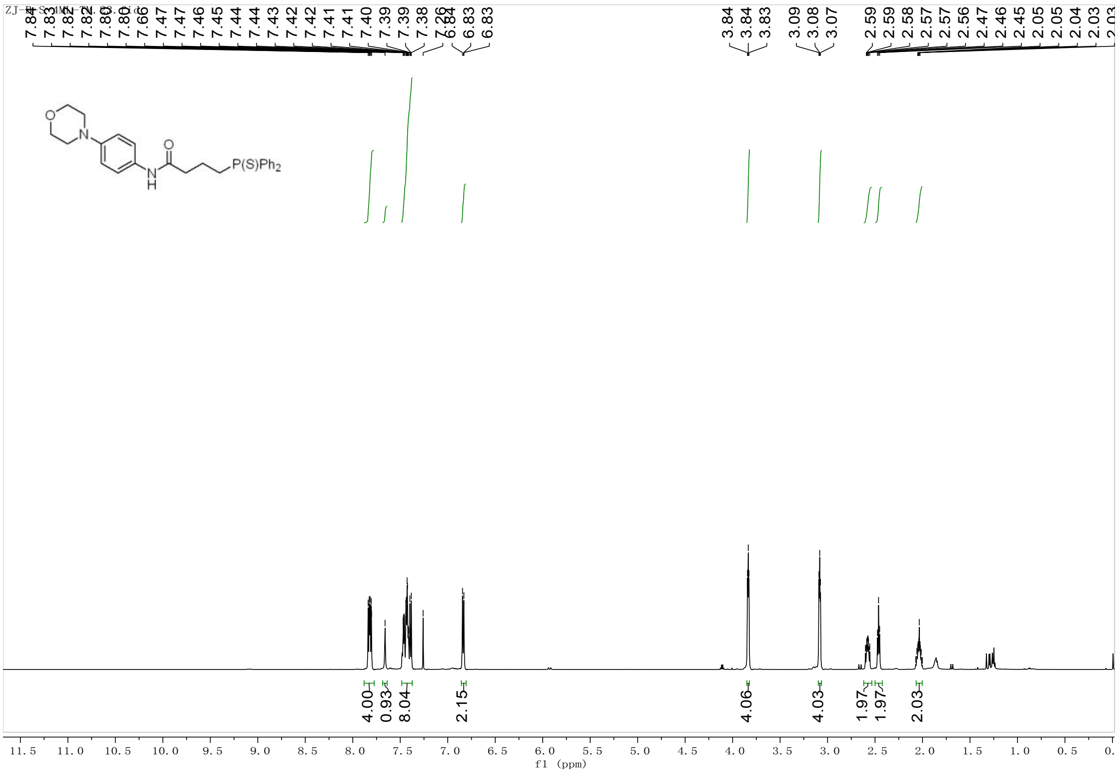

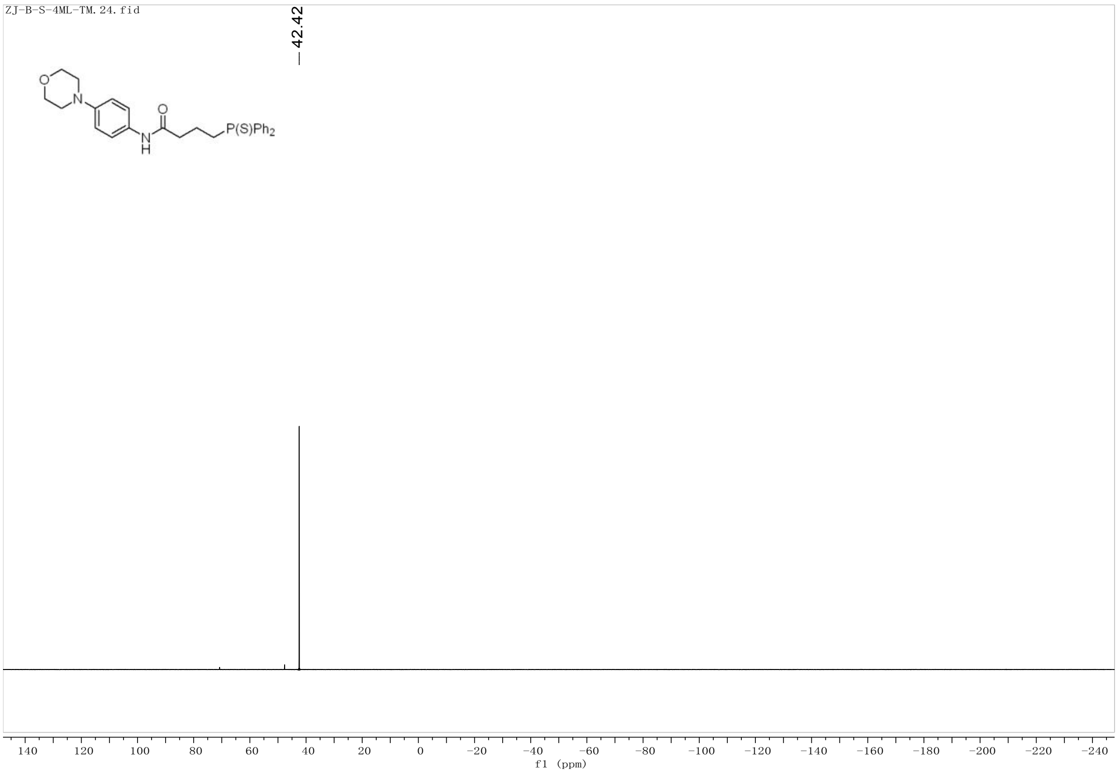

ZJ-B-S-4ML-TM.24.fid
42.42
P(S)Ph2
f1 (ppm)

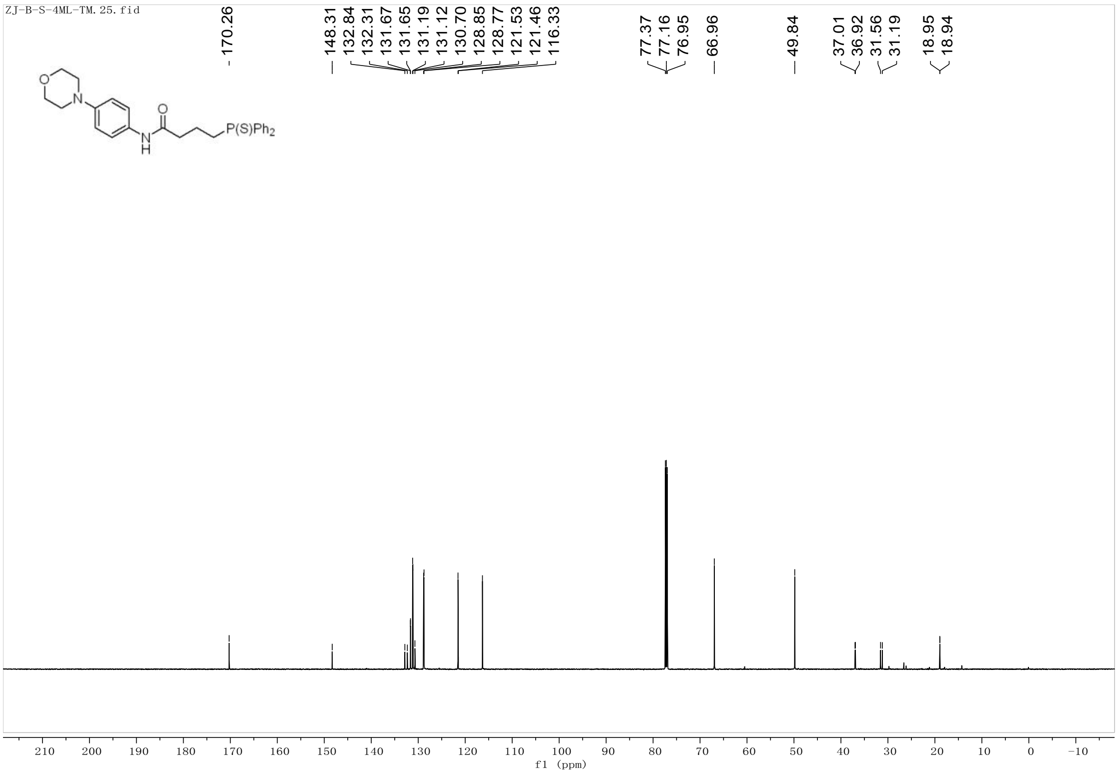

ZJ-B-S-4ML-TM.25.fid
170.26
148.31
132.84
132.31
131.67
131.65
131.19
131.12
130.70
128.85
128.77
121.53
121.46
116.33
77.37
77.16
76.95
66.96
49.84
37.01
36.92
31.56
31.19
18.95
18.94
P(S)Ph2
f1 (ppm)

N-(4-acetylphenyl)-4-(diphenylphosphorothioyl)butanamide (4q)

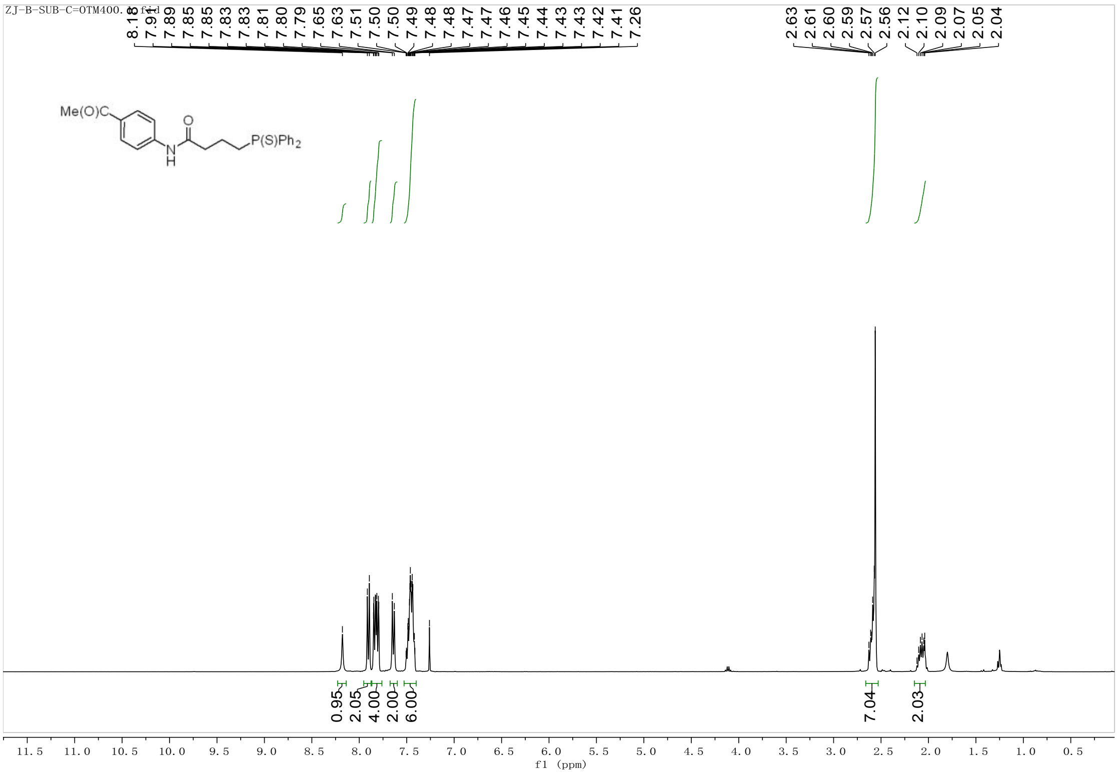

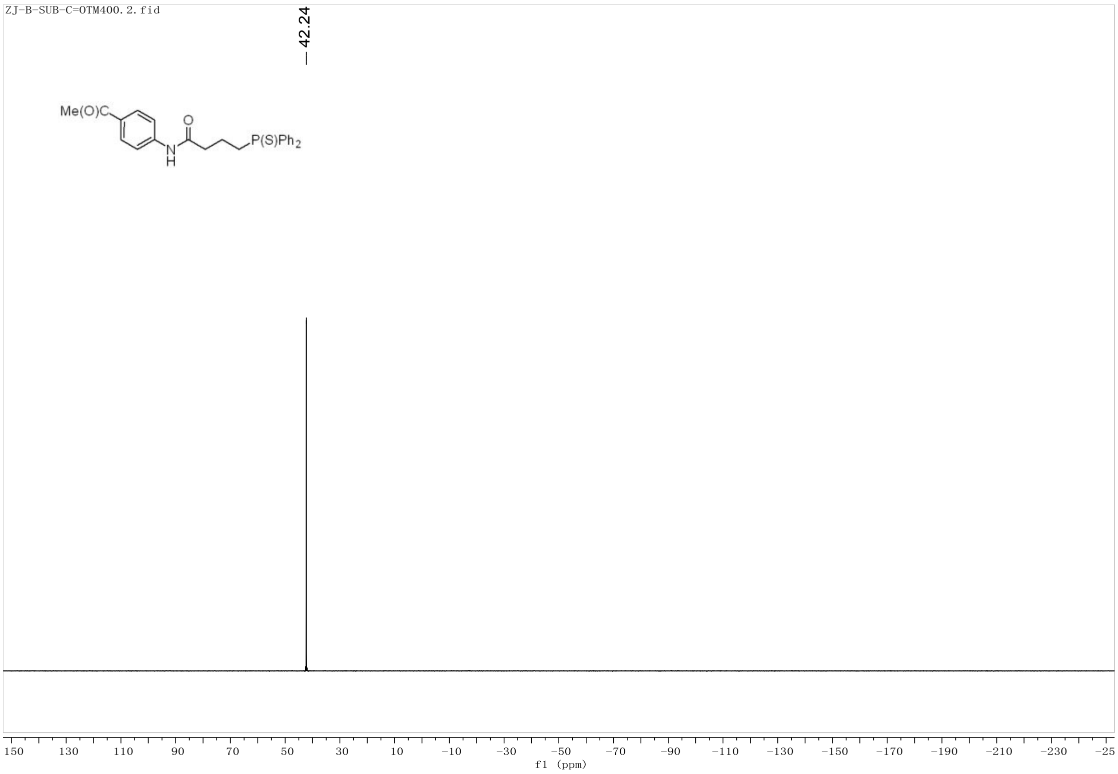

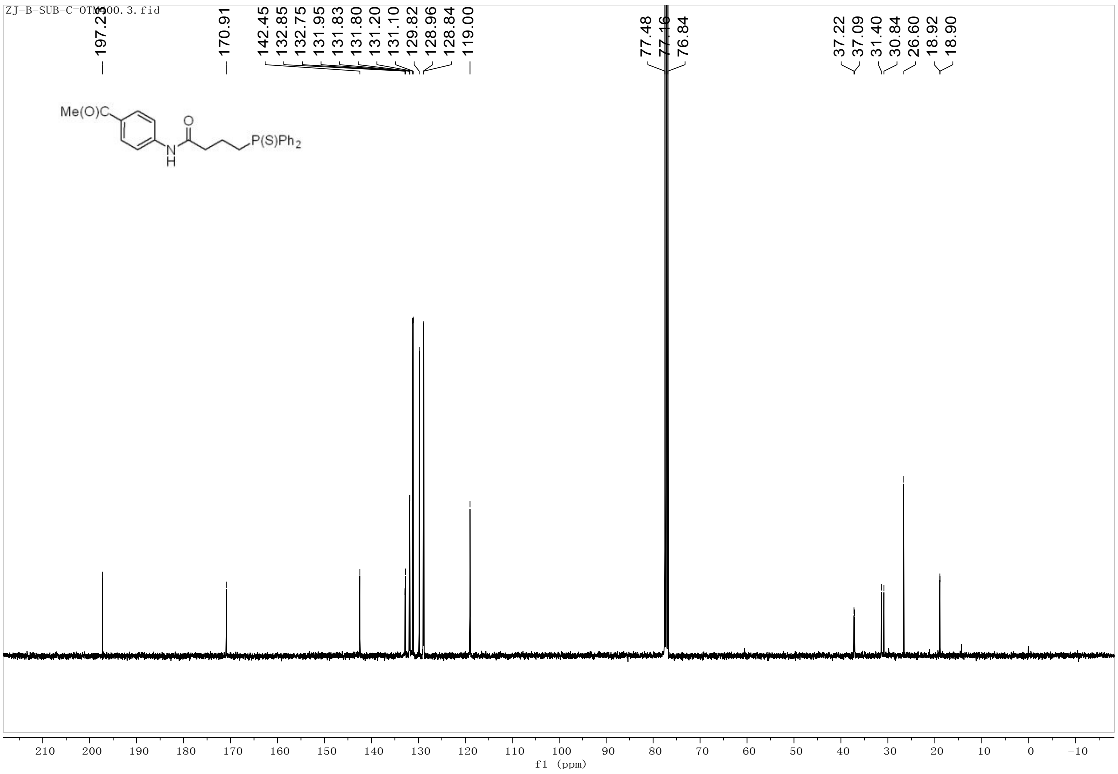

methyl 4-(4-(diphenylphosphorothioyl)butanamido)benzoate (4r)

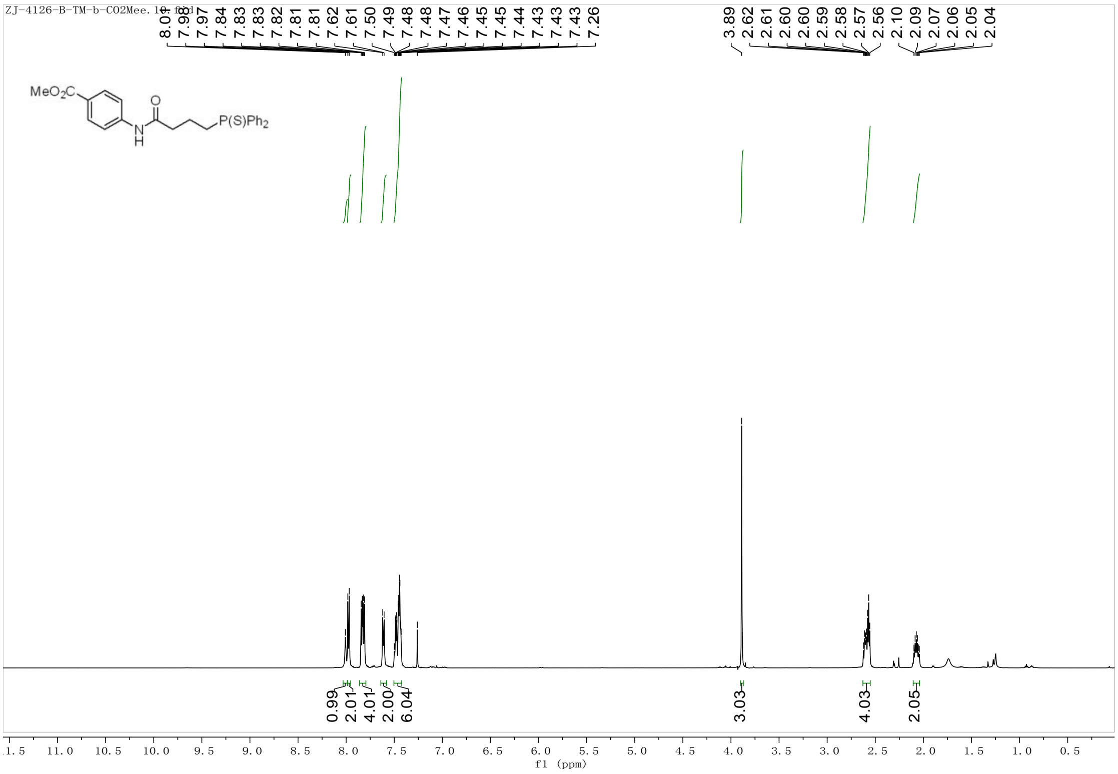

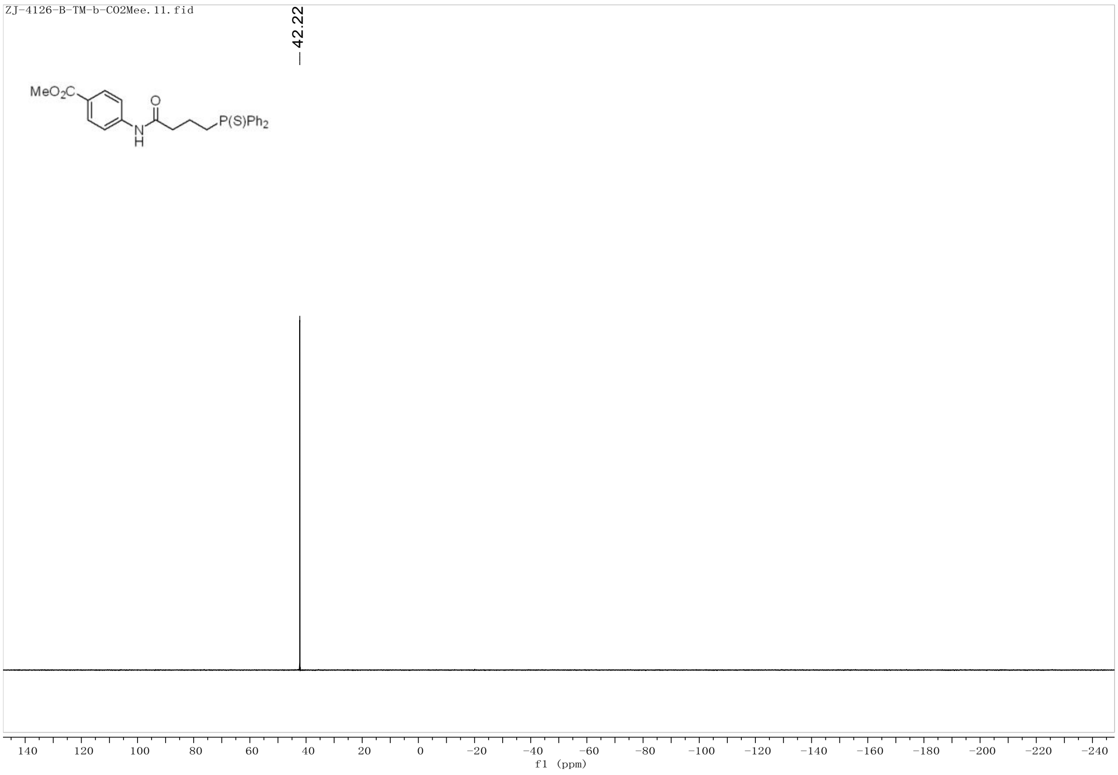
ZJ-4126-B-TM-b-CO2Mee.11.fid
42.22
MeO2C
N
H
O
P(S)Ph2
140 120 100 80 60 40 20 0 −20 −40 −60 −80 −100 −120 −140 −160 −180 −200 −220 −240
f1 (ppm)

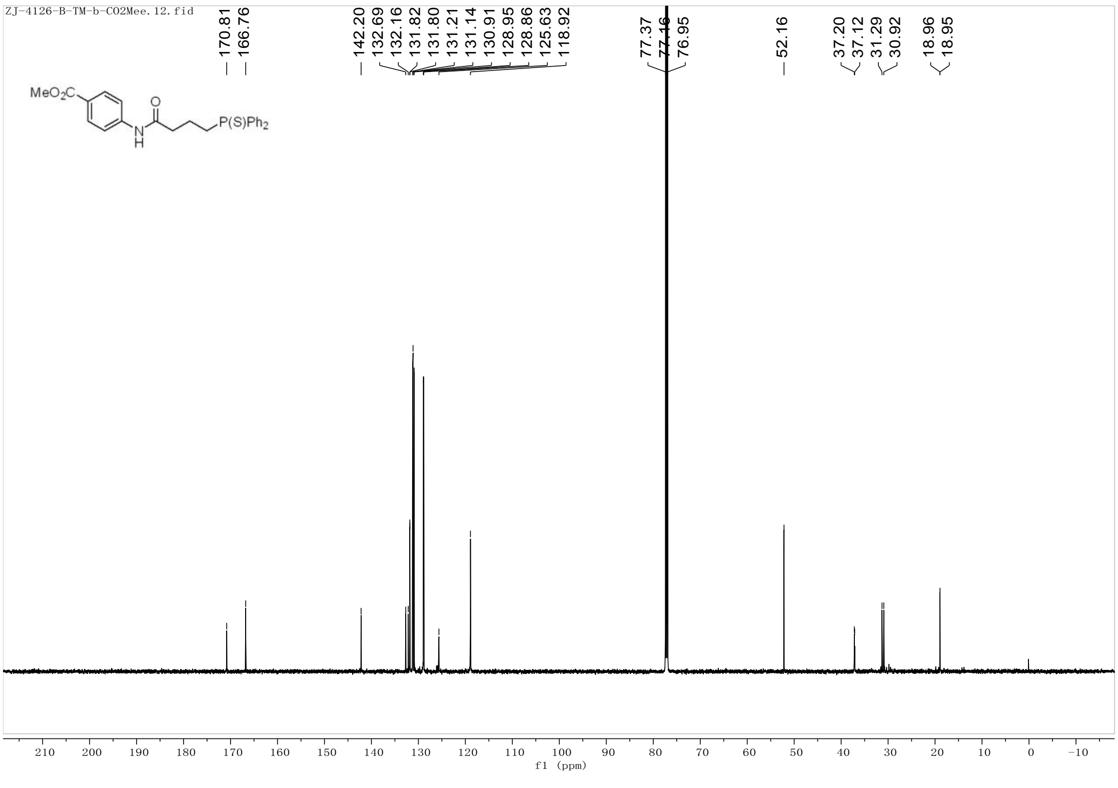
ZJ-4126-B-TM-b-CO2Mee.12.fid
170.81
166.76
142.20
132.69
132.16
131.82
131.80
131.21
131.14
130.91
128.95
128.86
125.63
118.92
77.37
77.16
76.95
52.16
37.20
37.12
31.29
30.92
18.96
18.95
MeO2C
N
H
O
P(S)Ph2
210 200 190 180 170 160 150 140 130 120 110 100 90 80 70 60 50 40 30 20 10 0 −10
f1 (ppm)

4-(diphenylphosphorothioyl)-N-(naphthalen-2-yl)butanamide (4s)

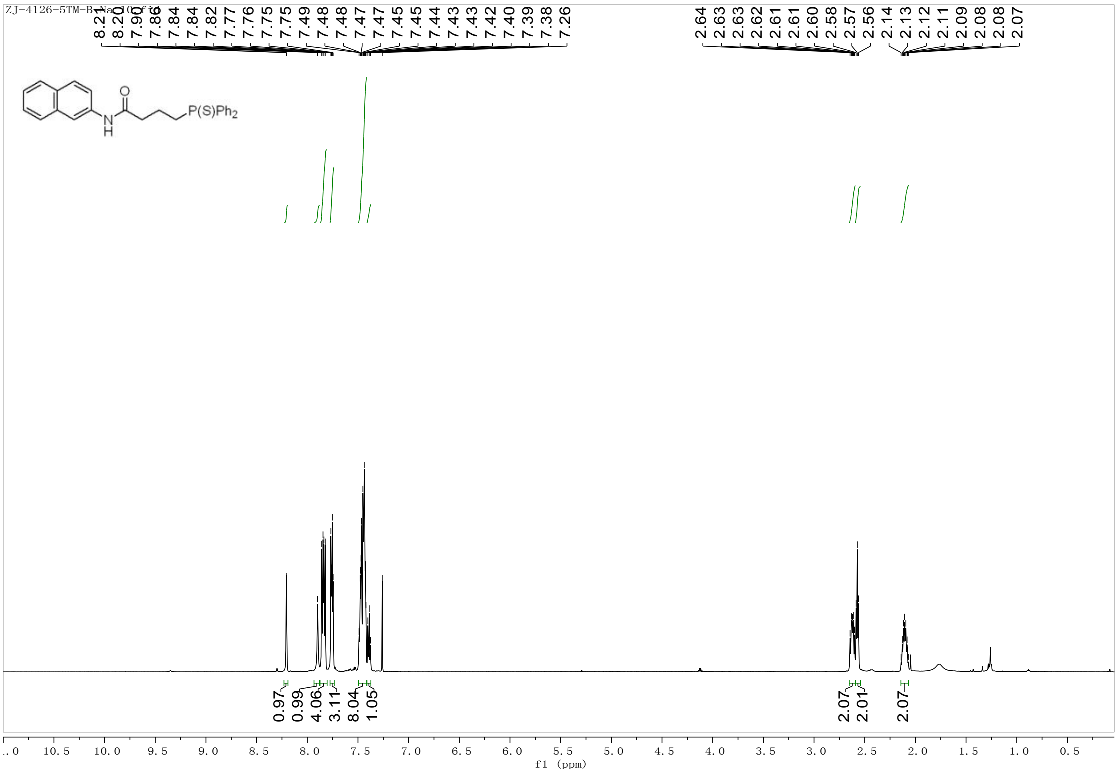

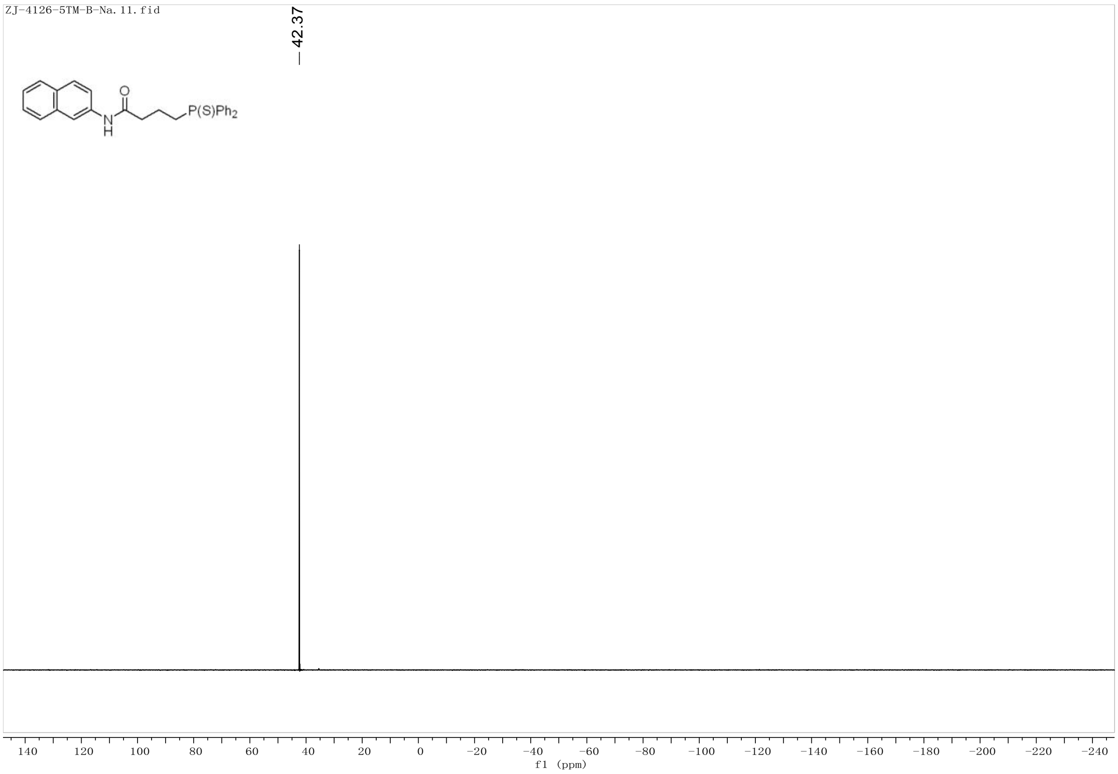

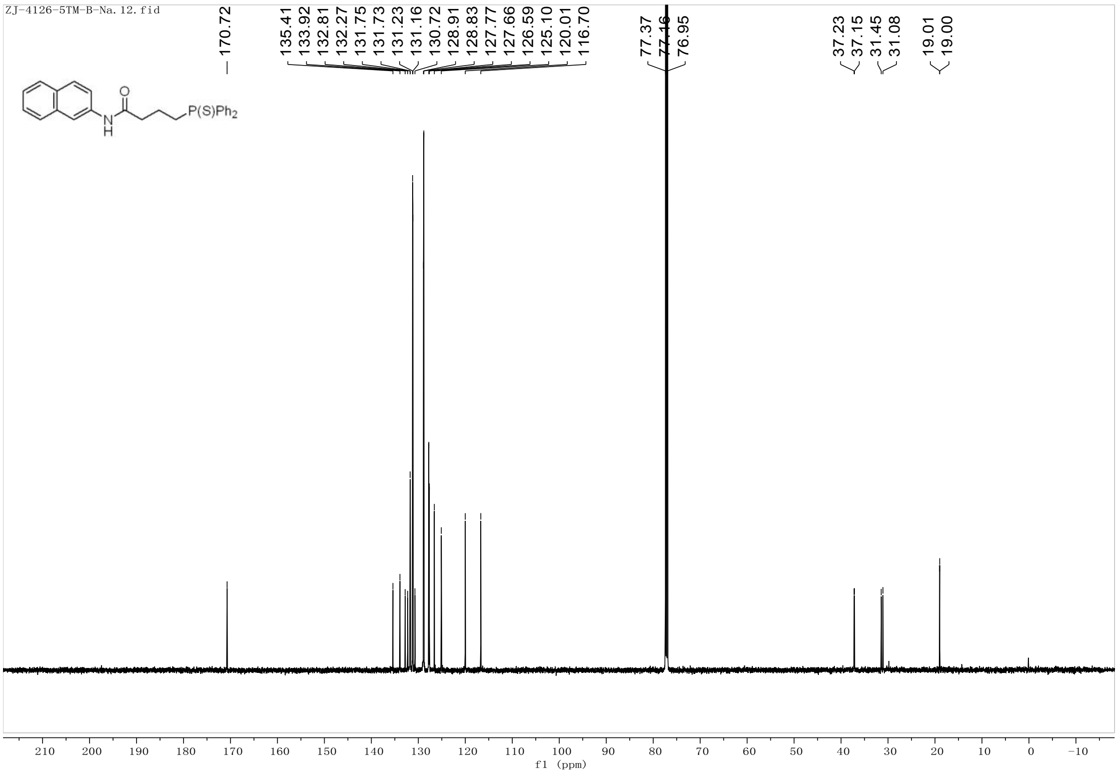

4-(diphenylphosphorothioyl)-N-(isoquinolin-4-yl)butanamide (4t)

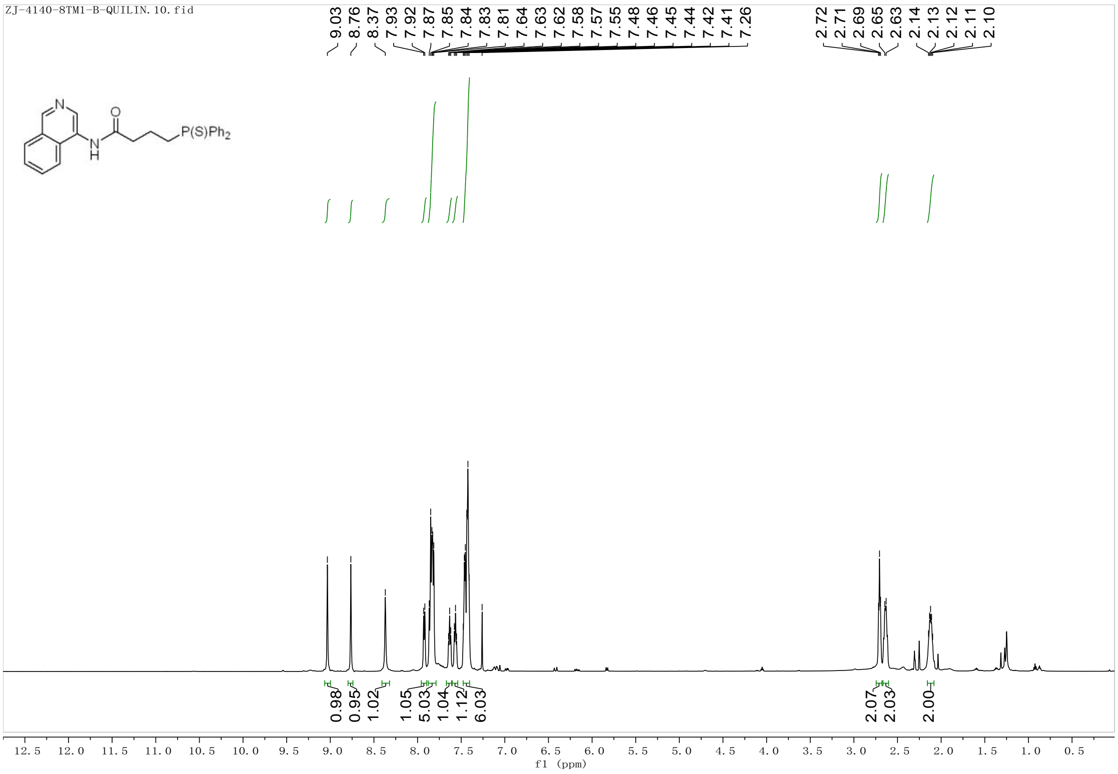

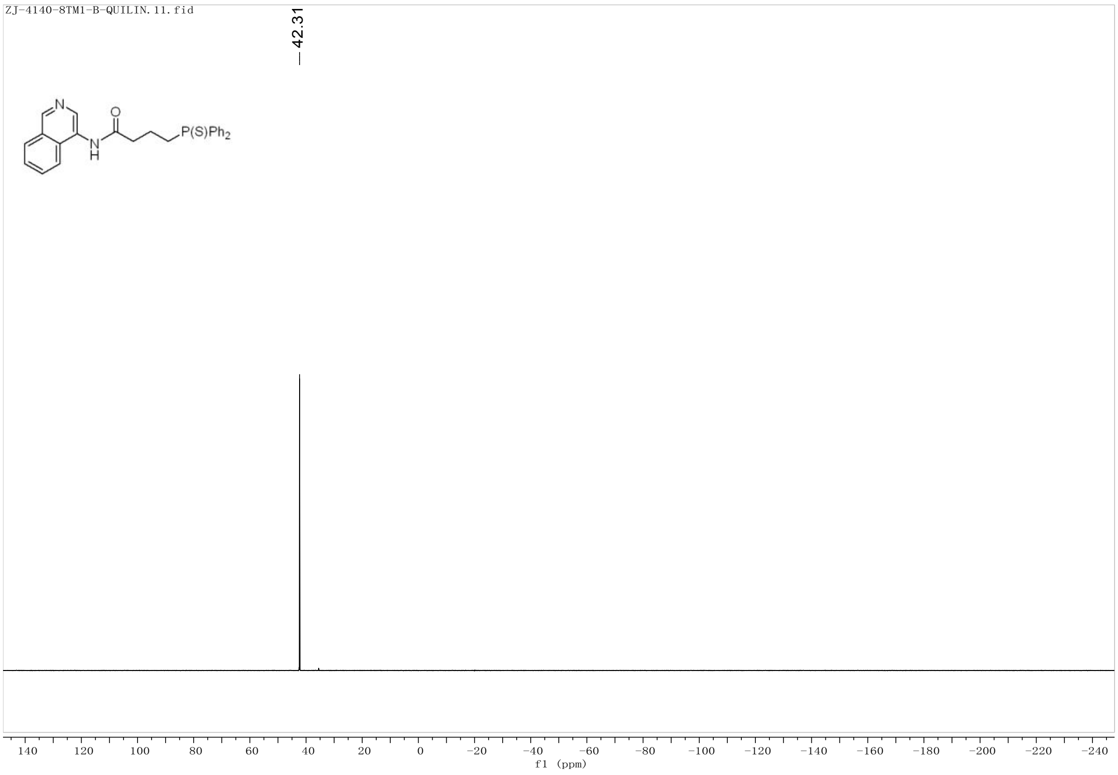
ZJ-4140-8TM1-B-QUILIN.11.fid
42.31
P(S)Ph2
N
O
H
f1 (ppm)

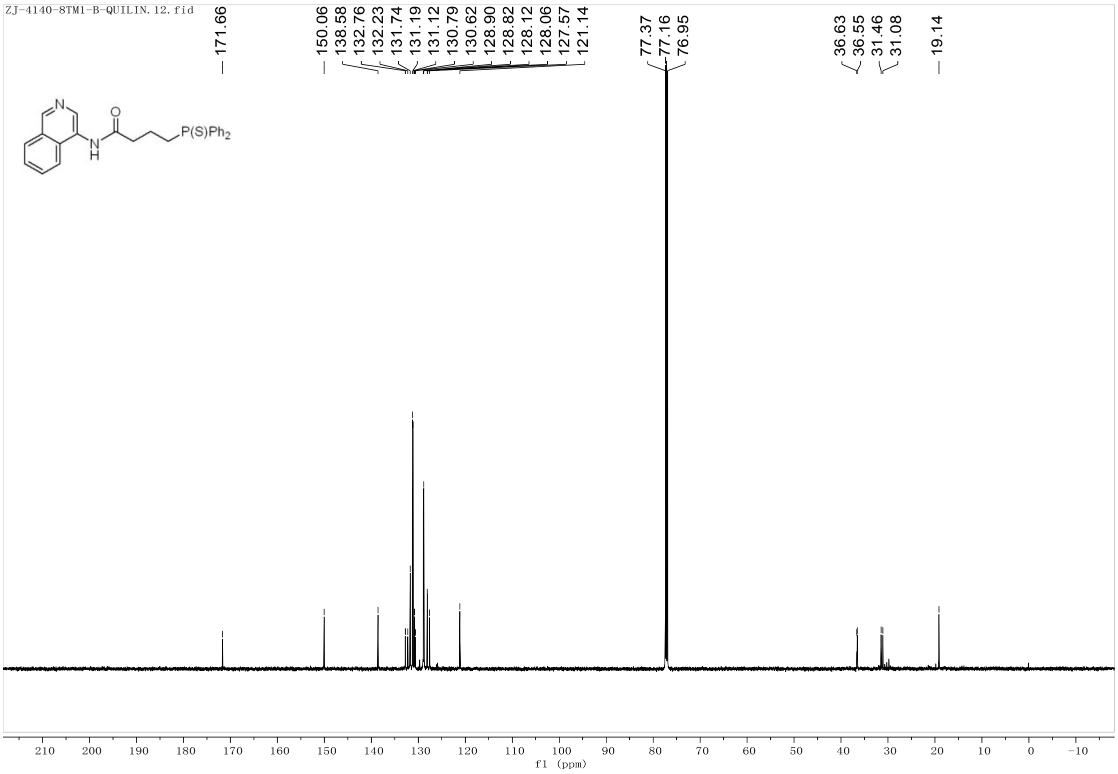
ZJ-4140-8TM1-B-QUILIN.12.fid
171.66
150.06
138.58
132.76
132.23
131.74
131.19
131.12
130.79
130.62
128.90
128.82
128.12
128.06
127.57
121.14
77.37
77.16
76.95
36.63
36.55
31.46
31.08
19.14
P(S)Ph2
f1 (ppm)

4-(diphenylphosphorothioyl)-N-(thiophen-3-yl)butanamide (4u)

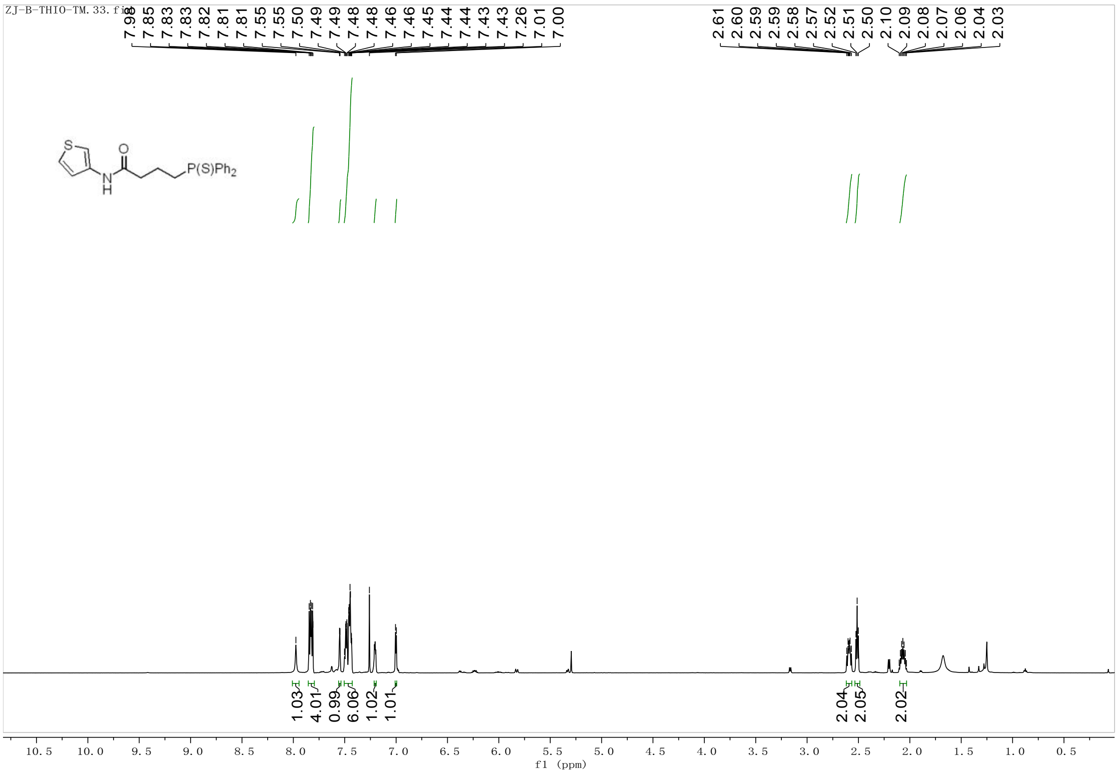

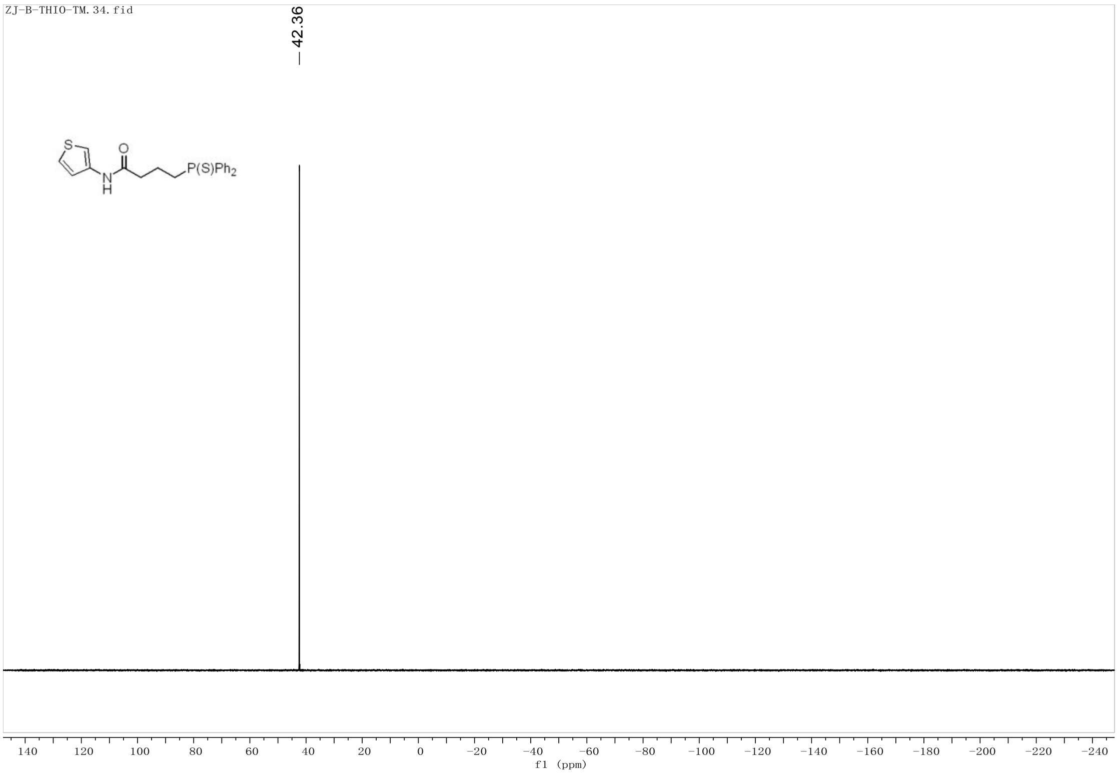

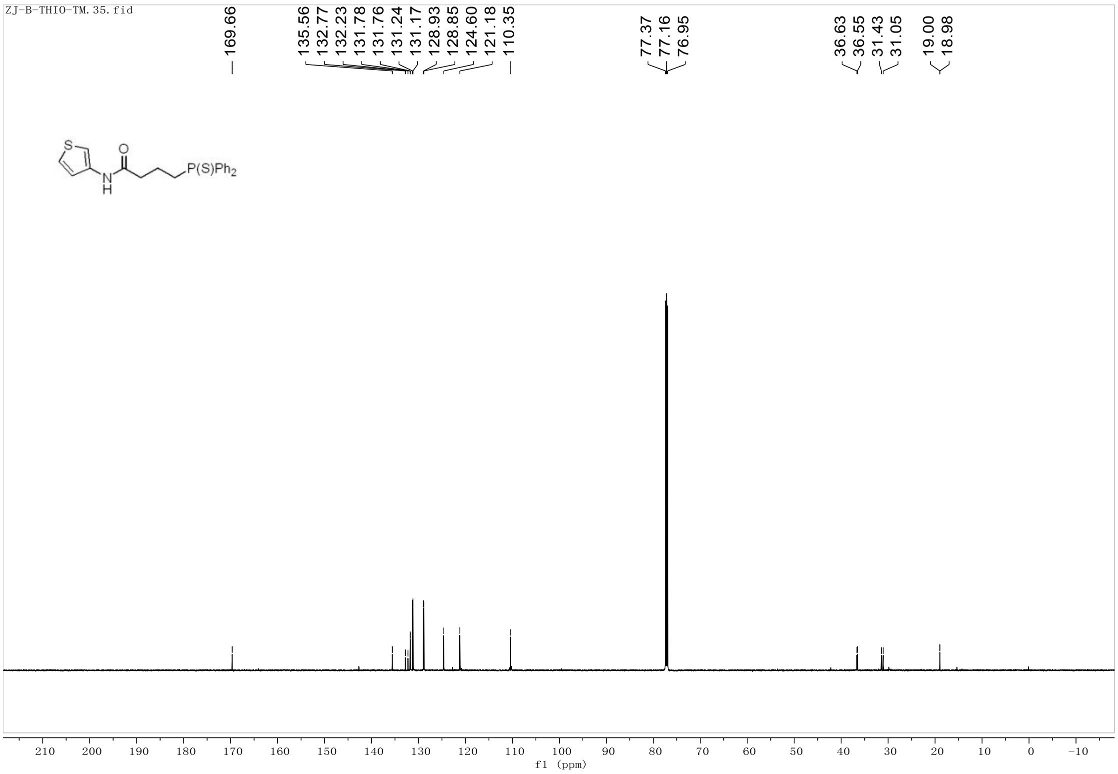

N-benzyl-4-(diphenylphosphorothioyl)butanamide (4v)

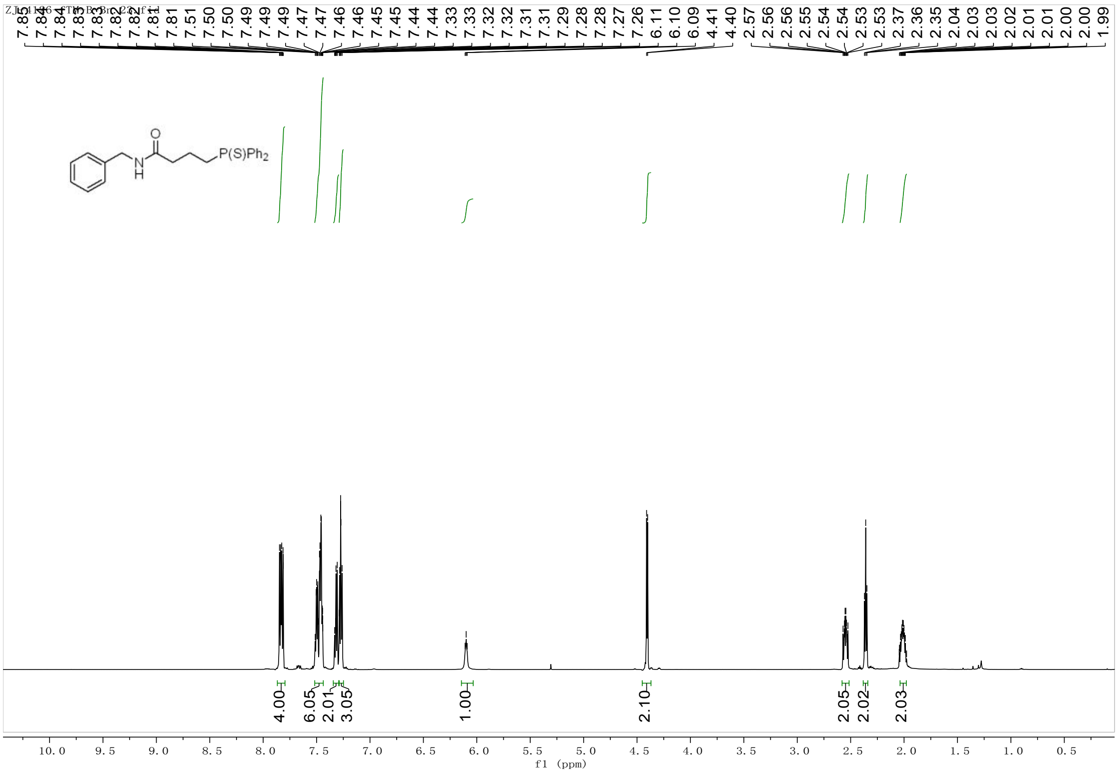

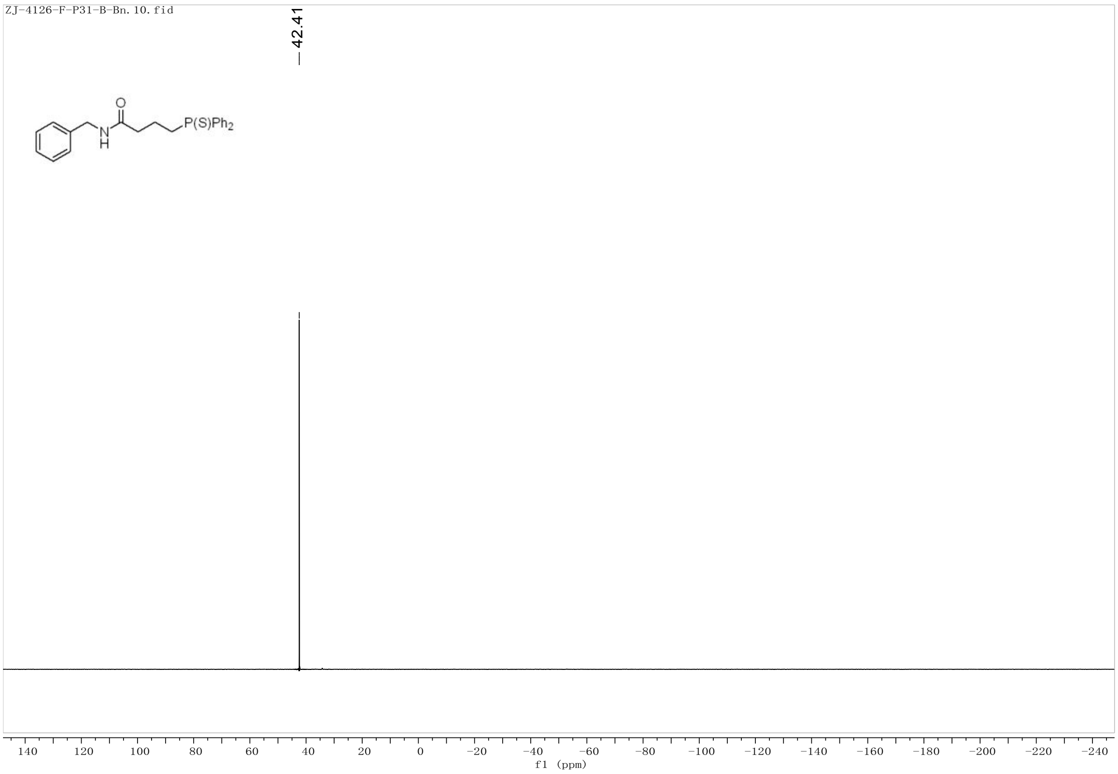

ZJ-4126-F-P31-B-Bn.10.fid
42.41
P(S)Ph2
N
H
O
f1 (ppm)

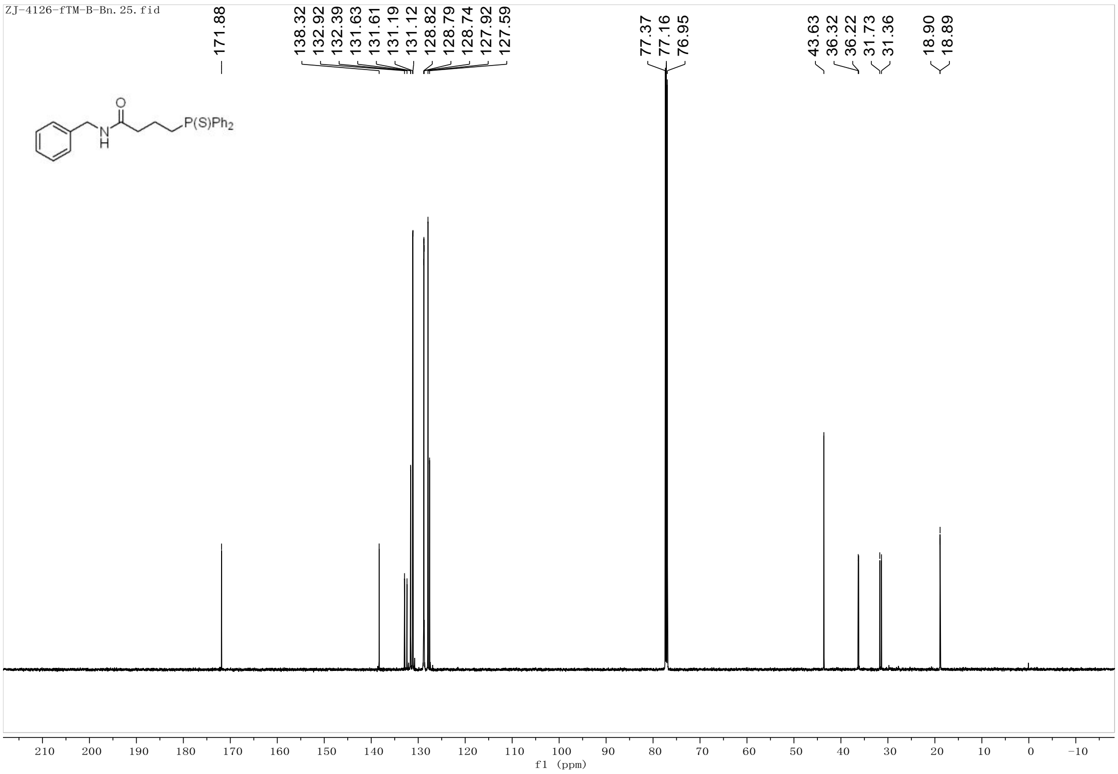

ZJ-4126-fTM-B-Bn.25.fid
171.88
138.32
132.92
132.39
131.63
131.61
131.19
131.12
128.82
128.79
128.74
127.92
127.59
77.37
77.16
76.95
43.63
36.32
36.22
31.73
31.36
18.90
18.89
P(S)Ph2
N
H
O
f1 (ppm)

methyl (S)-2-(4-(diphenylphosphorothioyl)butanamido)-2-phenylacetate (4w)

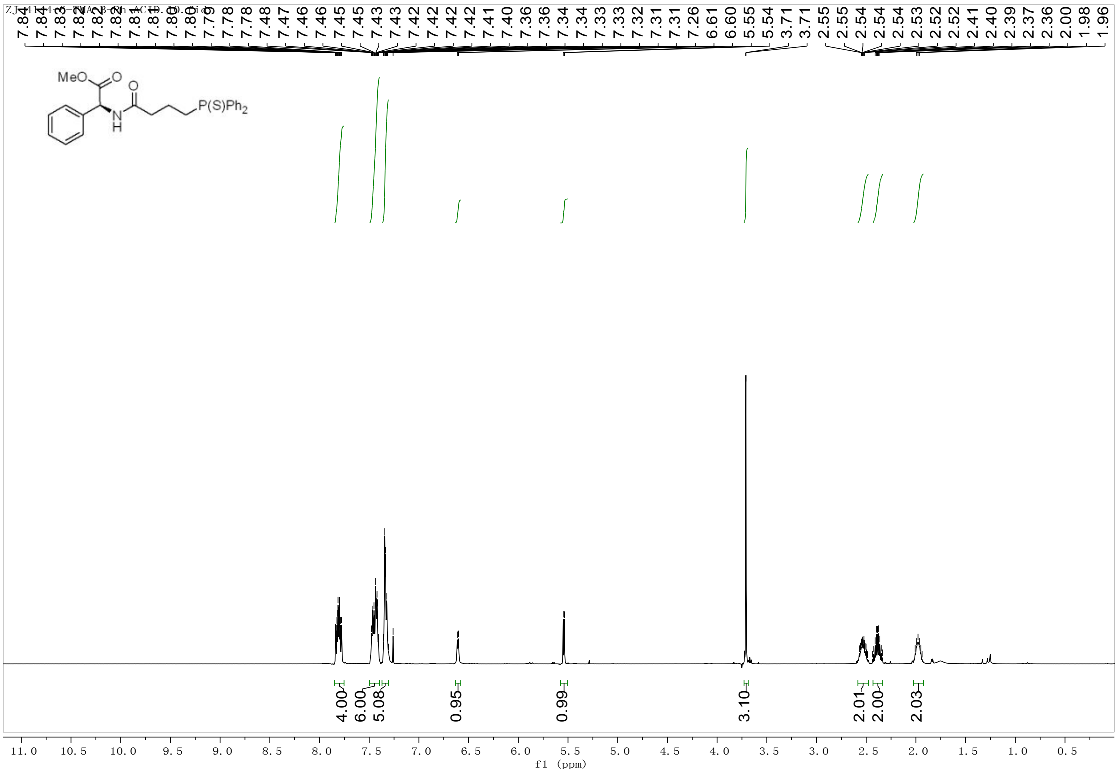

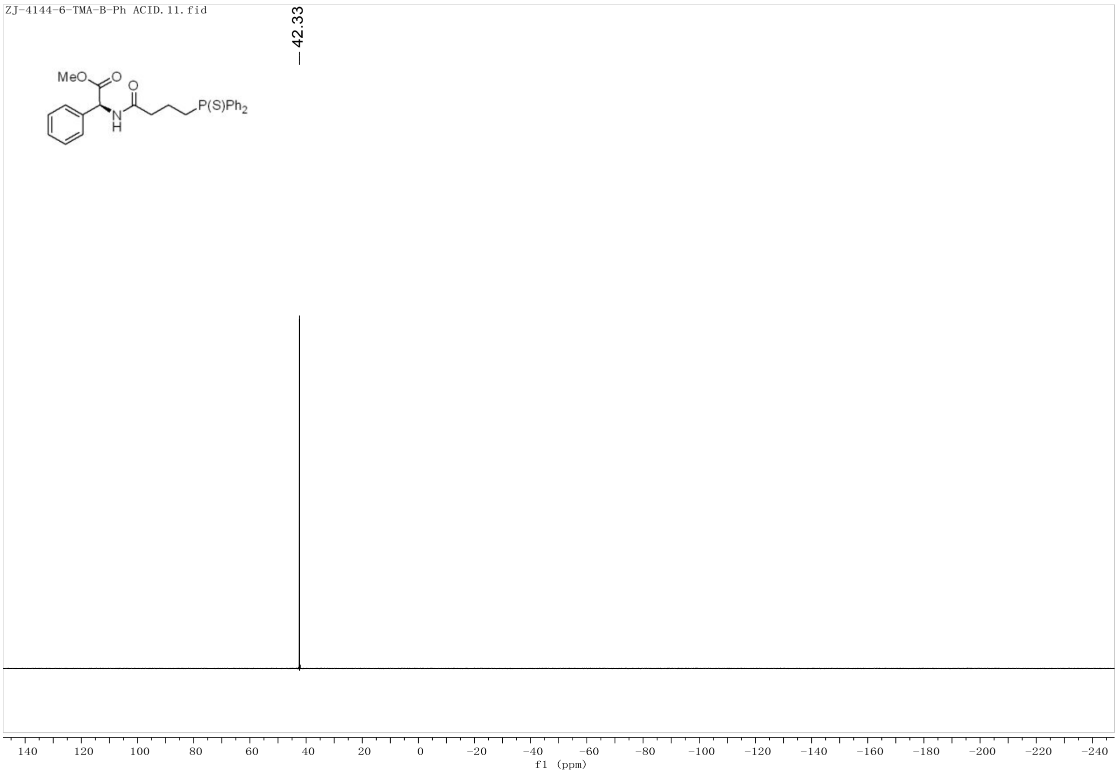

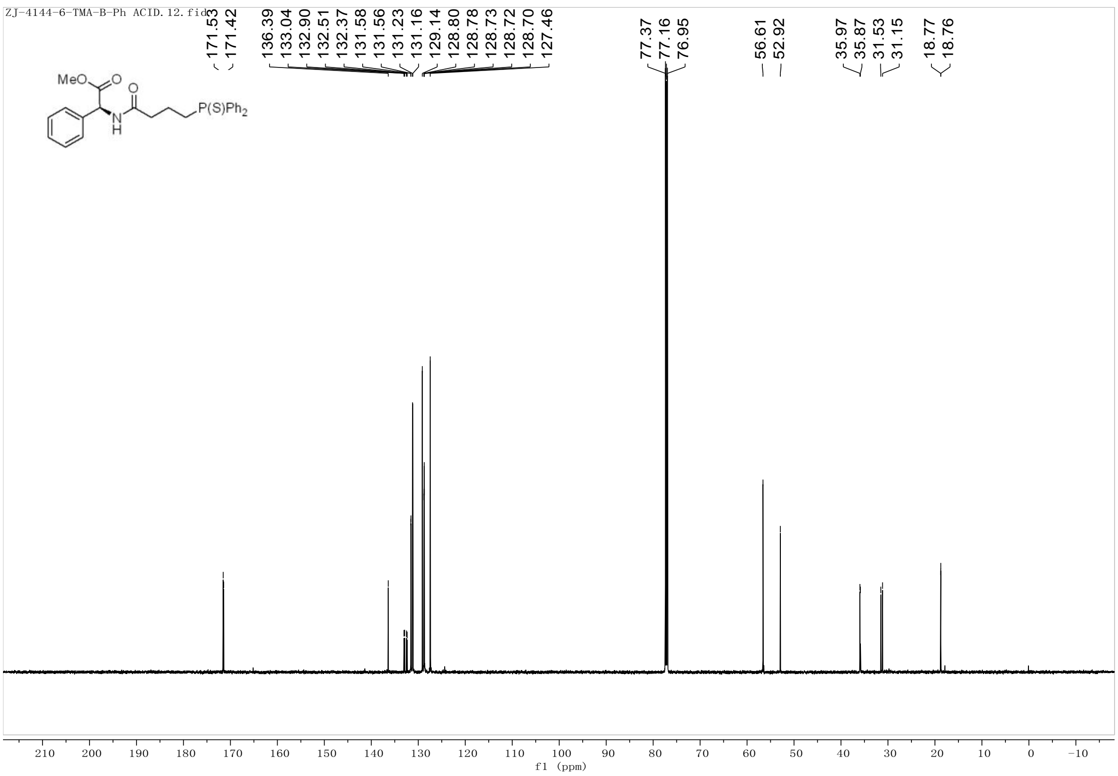

methyl (4-(diphenylphosphorothioyl)butanoyl)-L-alaninate (4x)

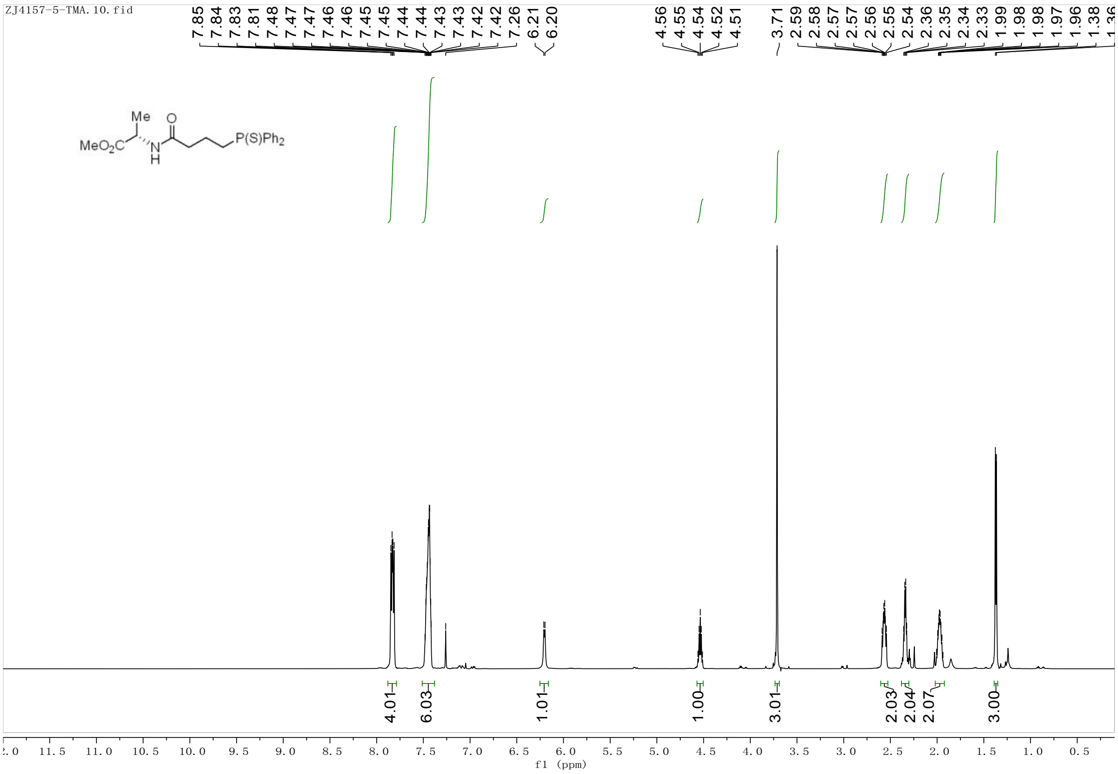

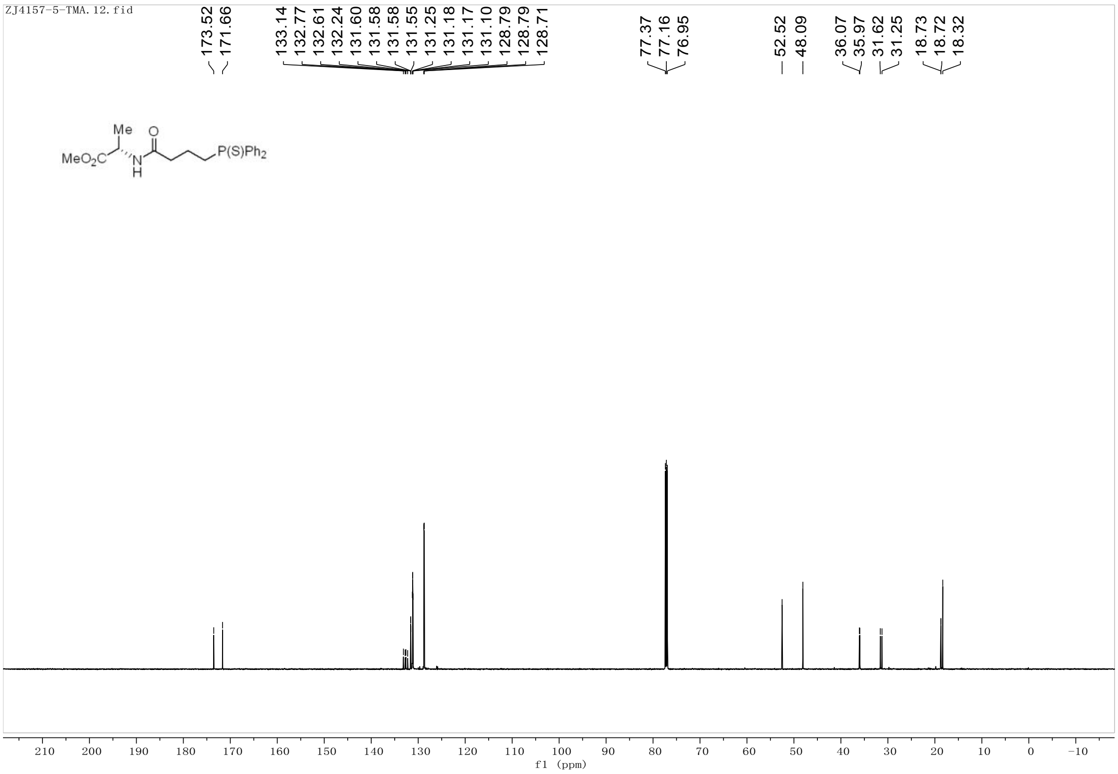
ZJ4157-5-TMA.12.fid
173.52
171.66
133.14
132.77
132.61
132.24
131.60
131.58
131.58
131.55
131.25
131.18
131.17
131.10
128.79
128.79
128.71
77.37
77.16
76.95
52.52
48.09
36.07
35.97
31.62
31.25
18.73
18.72
18.32
Me
MeO2C
N
H
O
P(S)Ph2
f1 (ppm)

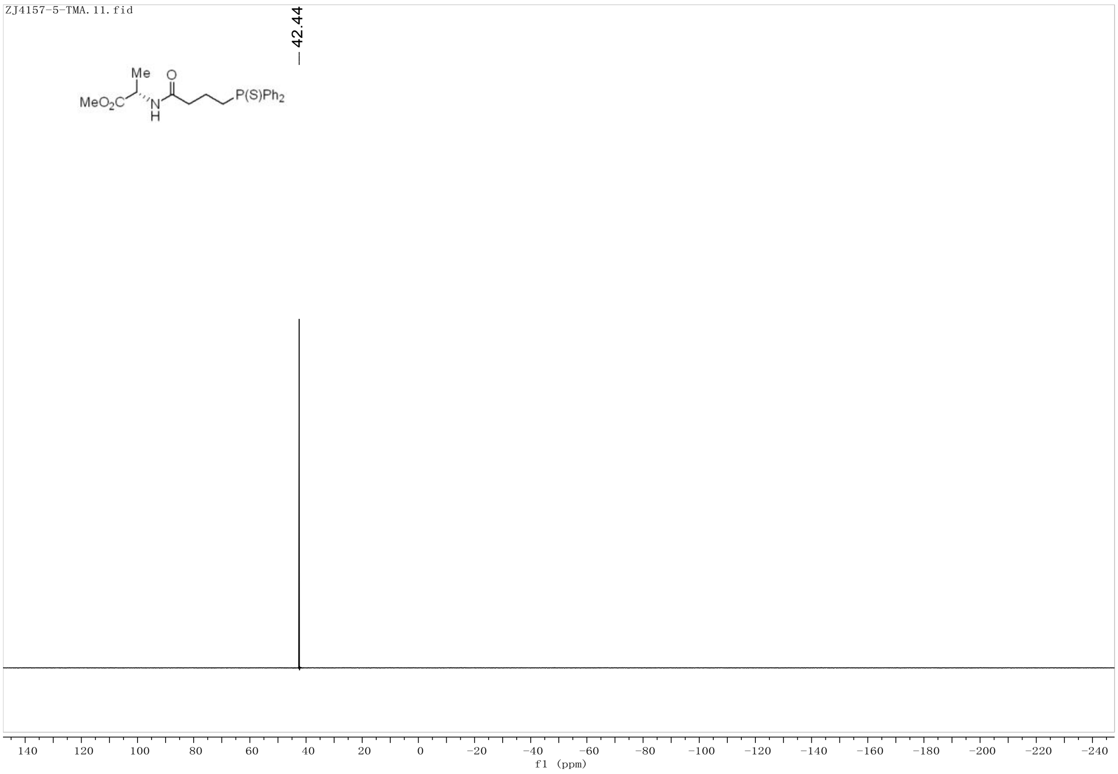
ZJ4157-5-TMA.11.fid
42.44
Me
MeO2C
N
H
O
P(S)Ph2
f1 (ppm)

5-(diphenylphosphorothioyl)-N-phenylpentanamide (4y)

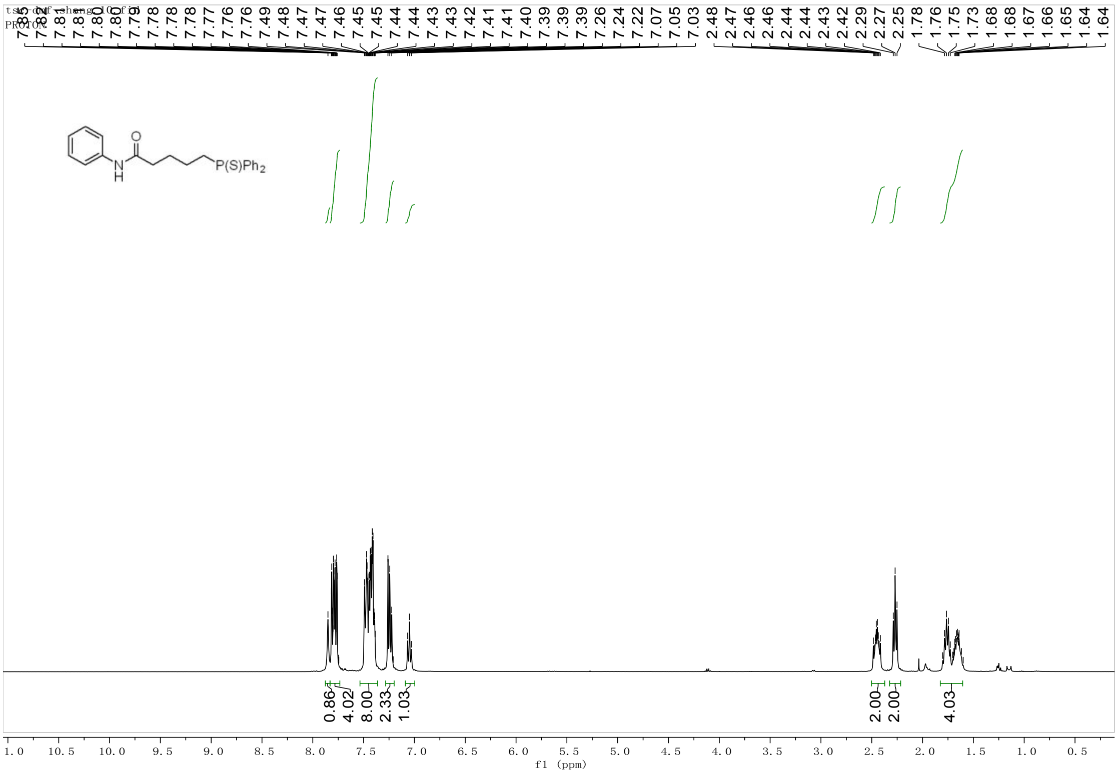

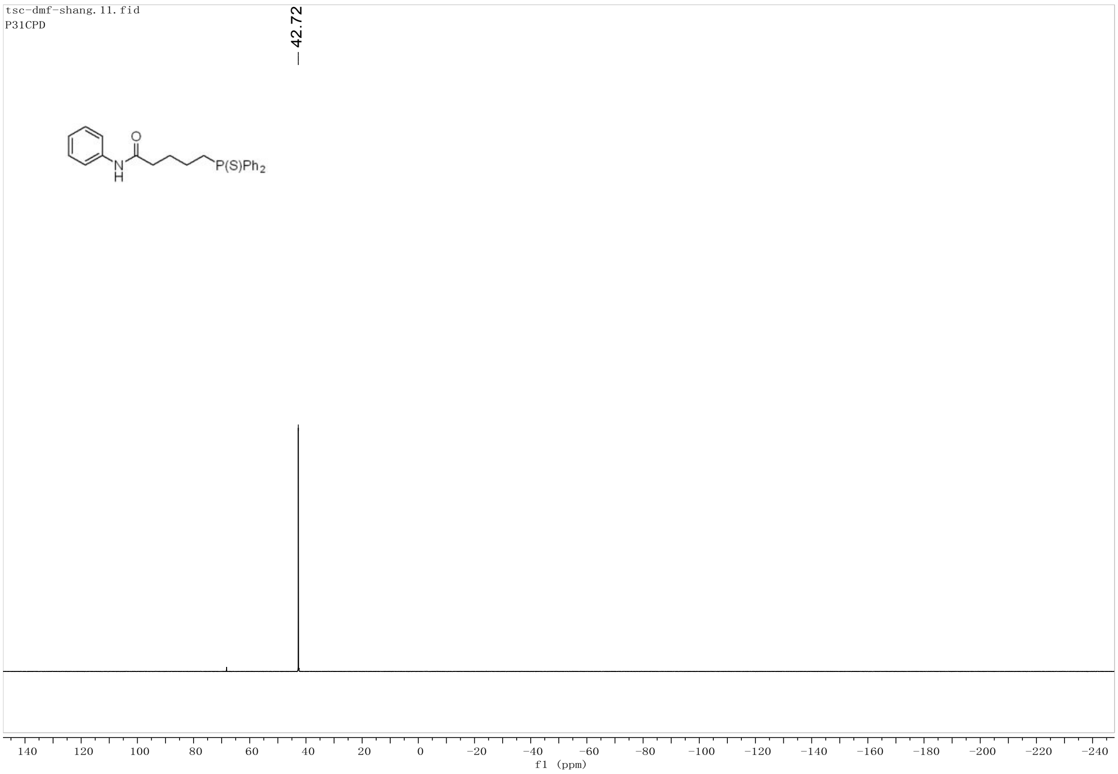

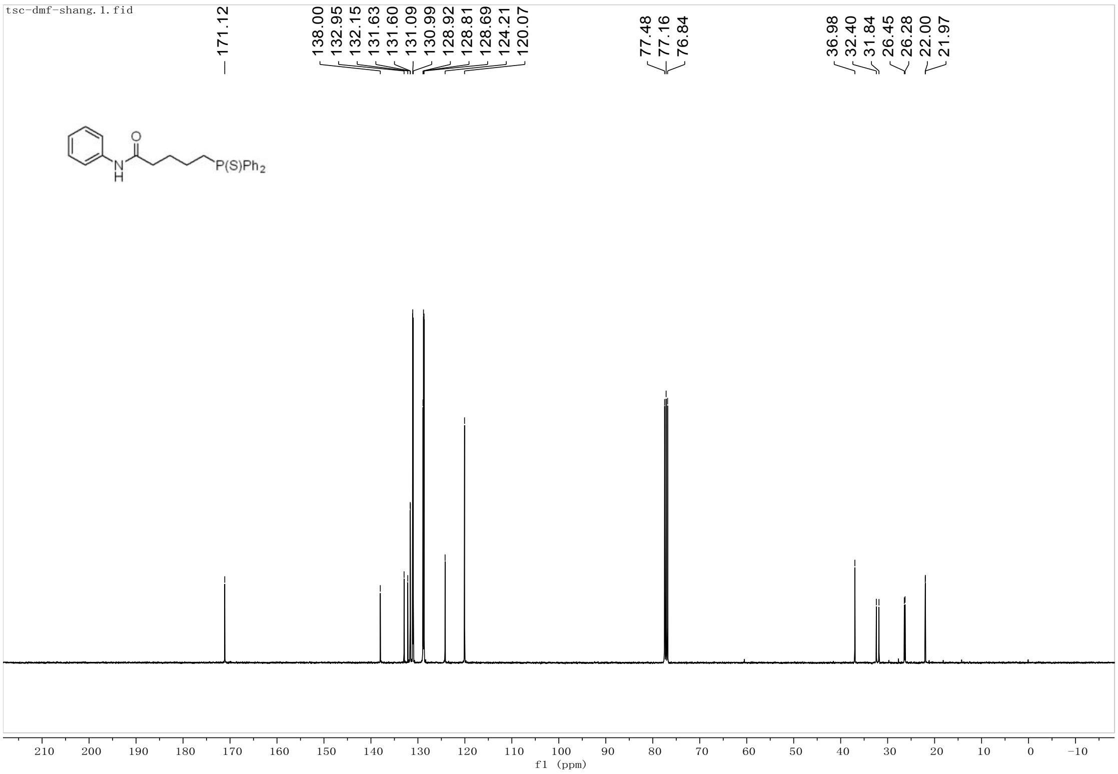

4-(di-p-tolylphosphorothioyl)-N-phenylbutanamide (4z)

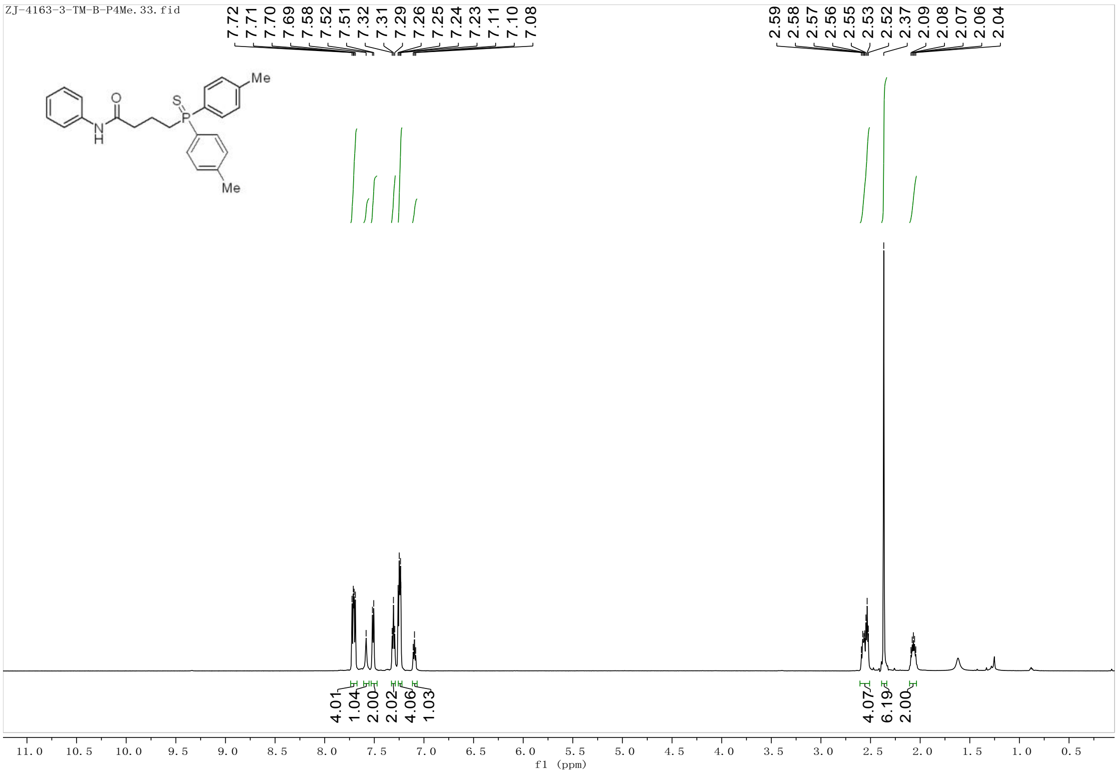

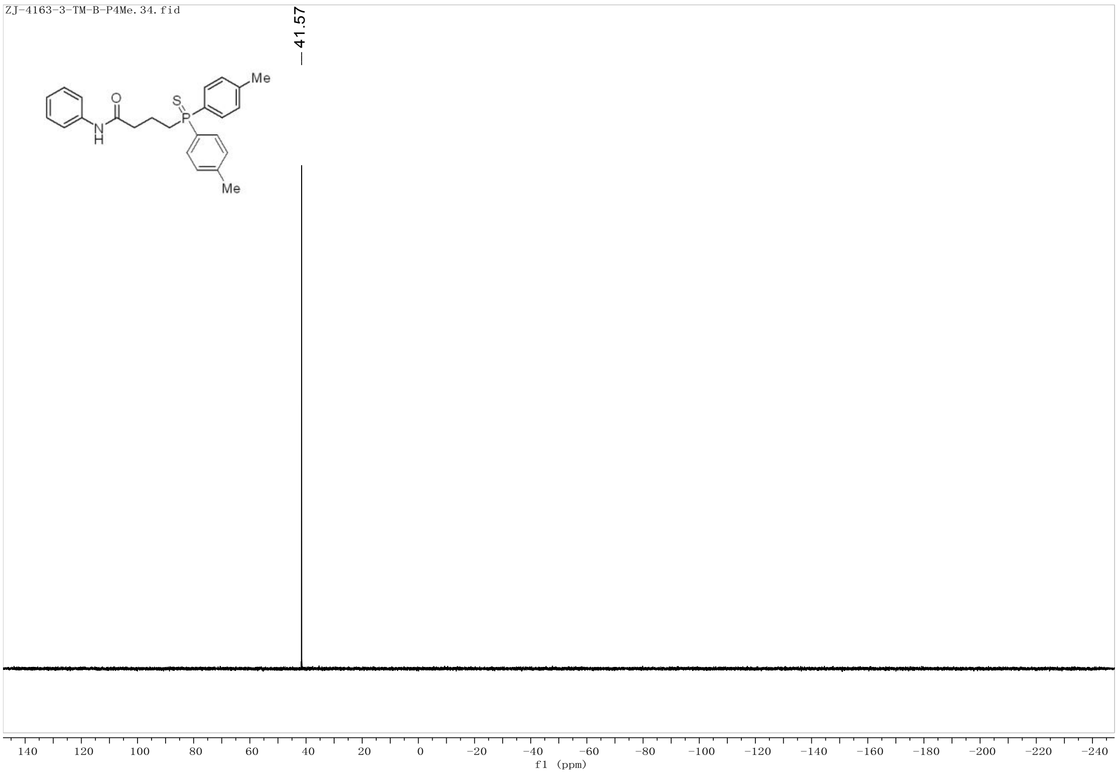
ZJ-4163-3-TM-B-P4Me.34.fid
41.57
f1 (ppm)

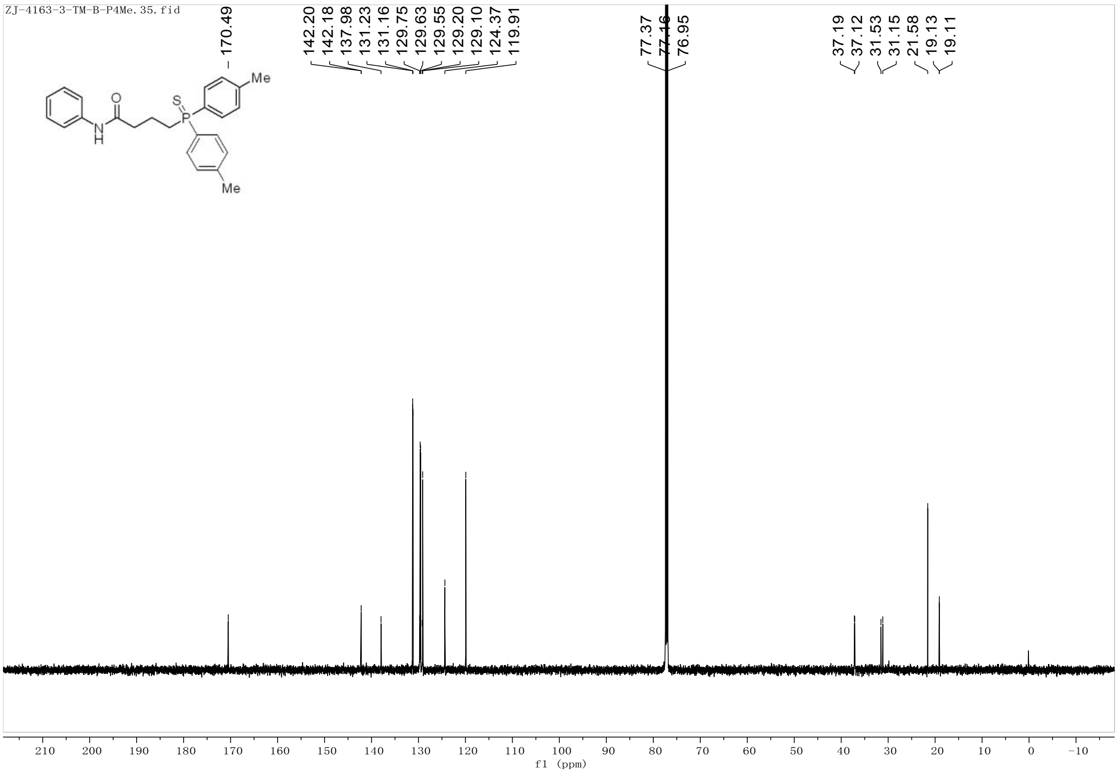
ZJ-4163-3-TM-B-P4Me.35.fid
170.49
142.20
142.18
137.98
131.23
131.16
129.75
129.63
129.55
129.20
129.10
124.37
119.91
77.37
77.16
76.95
37.19
37.12
31.53
31.15
21.58
19.13
19.11
f1 (ppm)

4-(di-m-tolylphosphorothioyl)-N-phenylbutanamide (4aa)

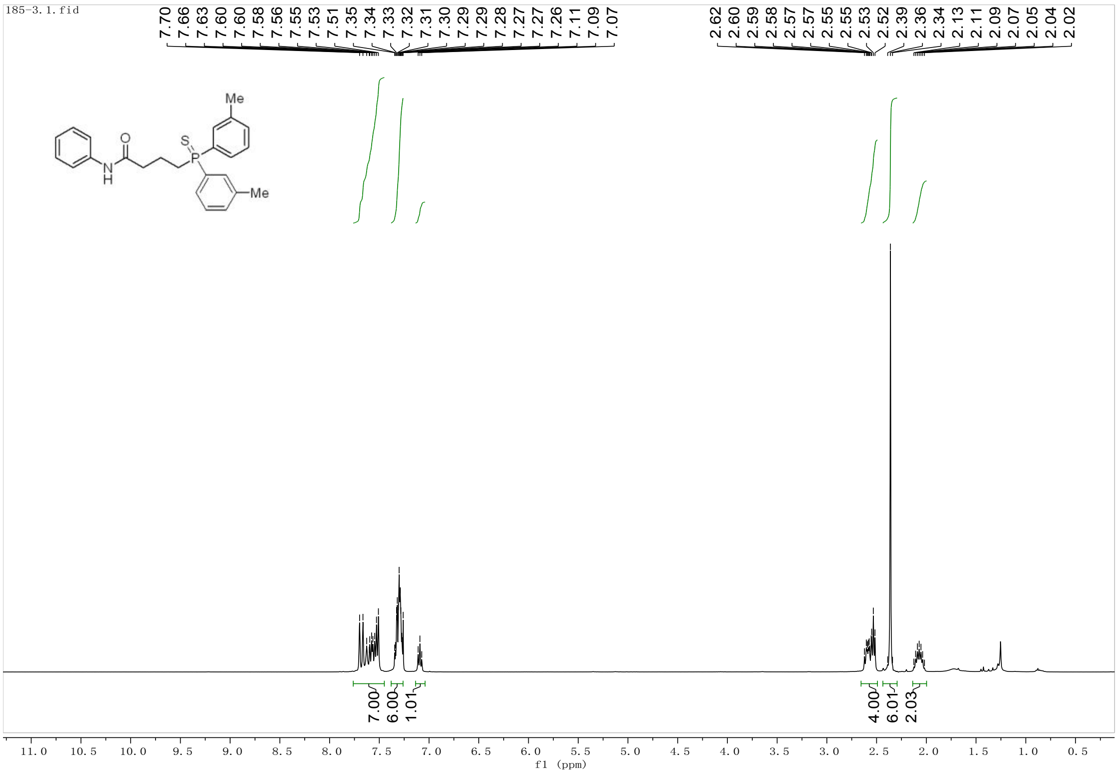

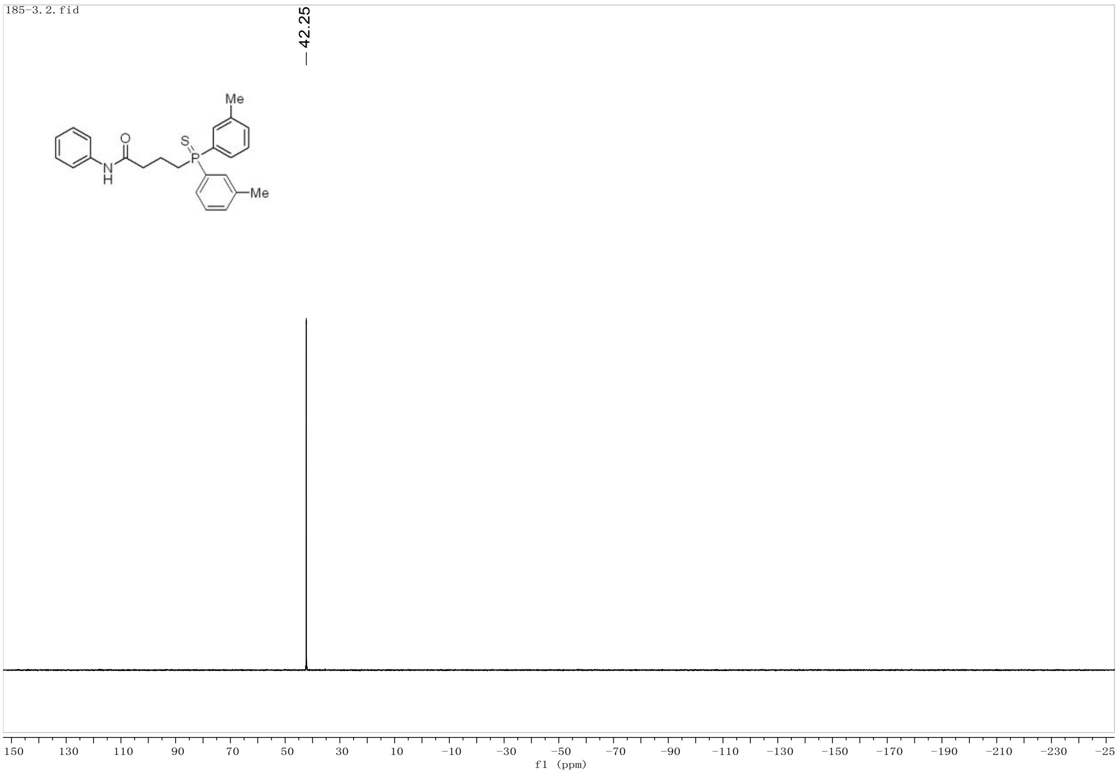

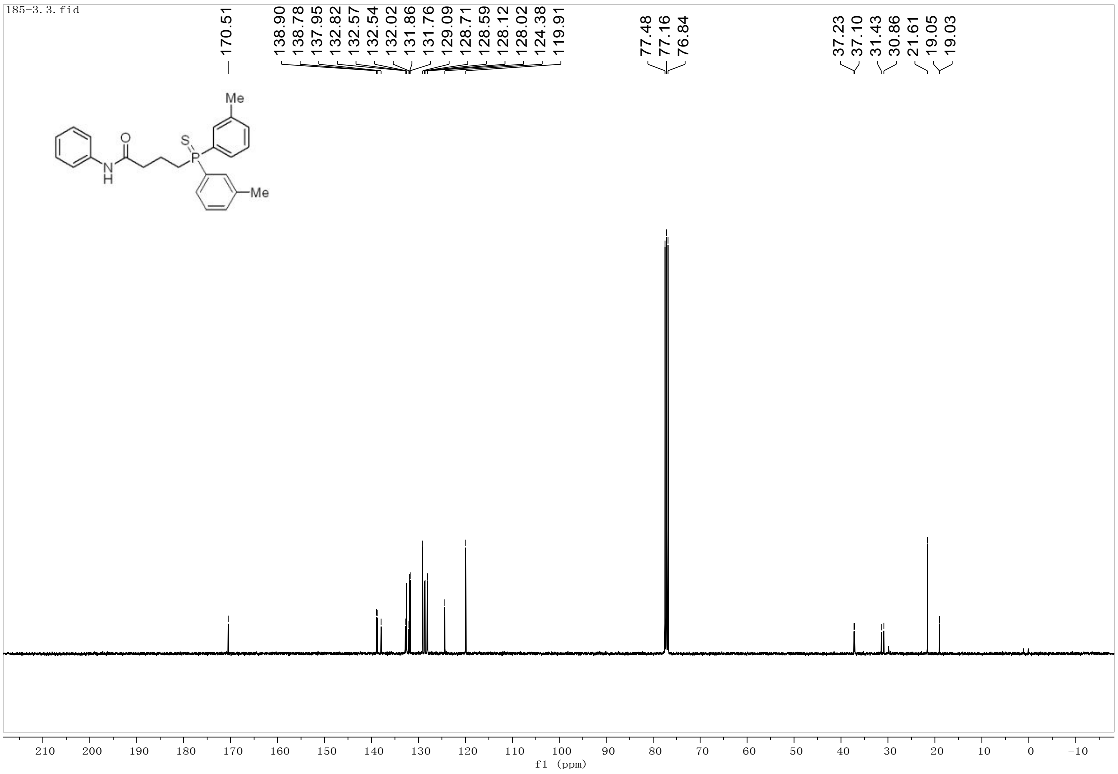

4-(bis(4-(tert-butyl)phenyl)phosphorothioyl)-N-phenylbutanamide (4ab)

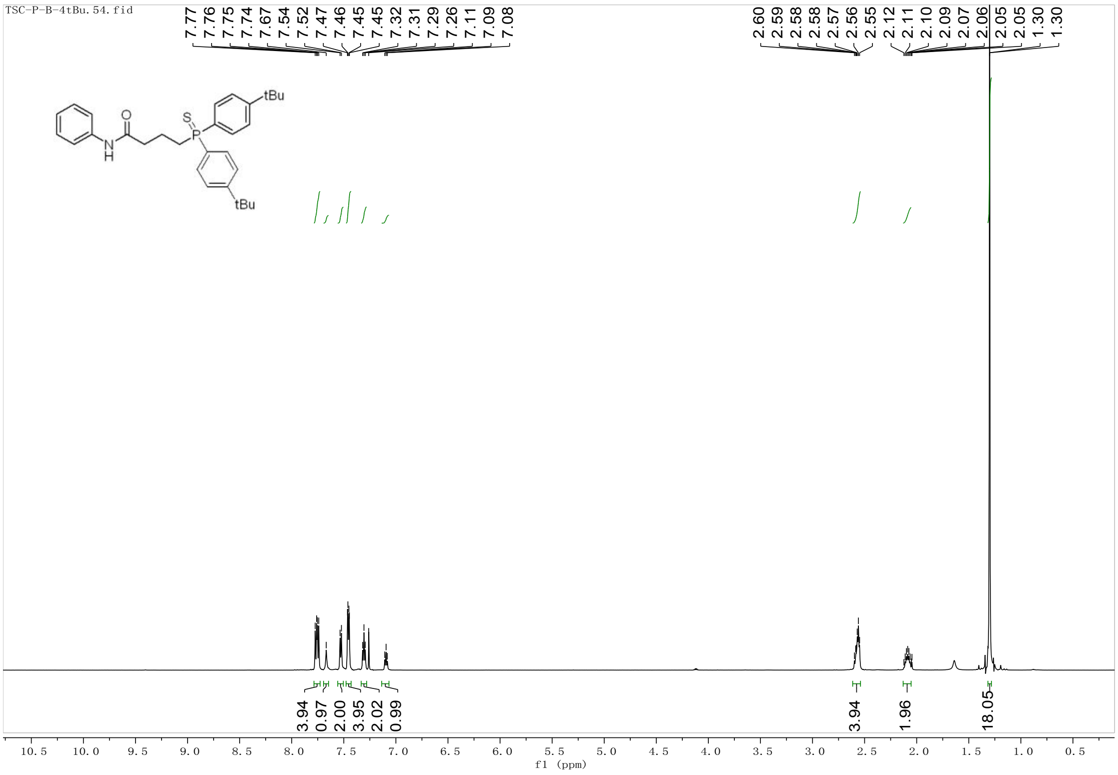

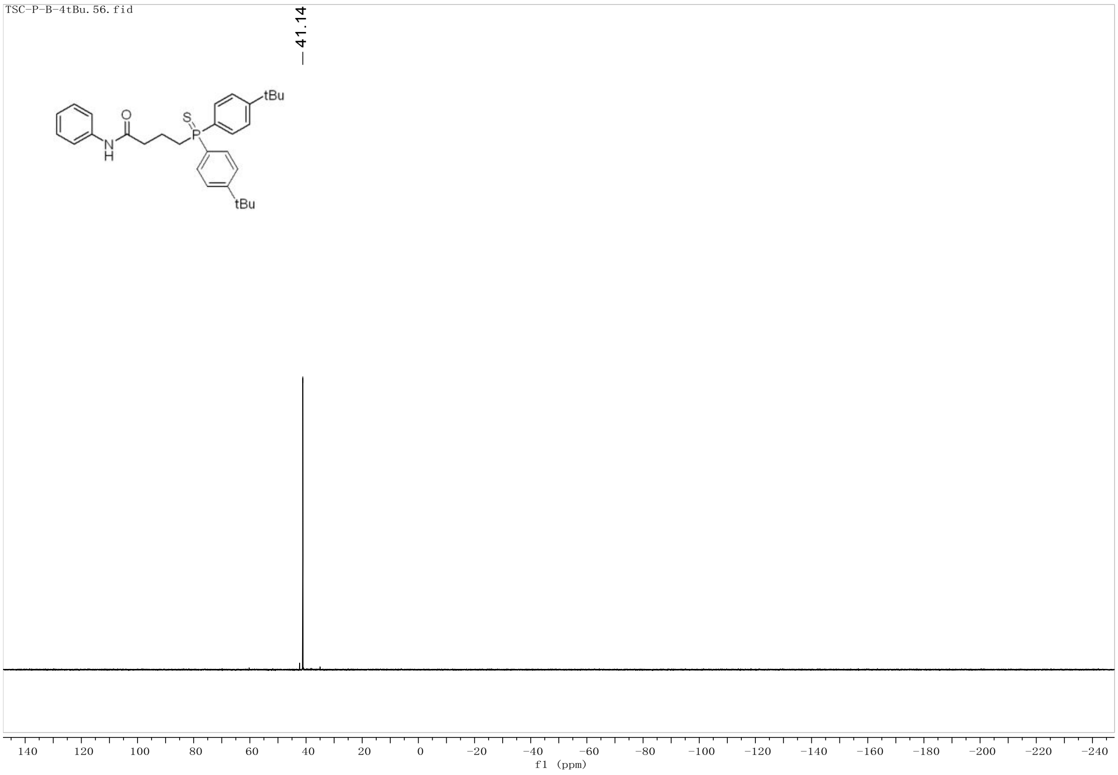
TSC-P-B-4tBu.56.fid
41.14
tBu
S
O
P
N
H
tBu
140 120 100 80 60 40 20 0 −20 −40 −60 −80 −100 −120 −140 −160 −180 −200 −220 −240
f1 (ppm)

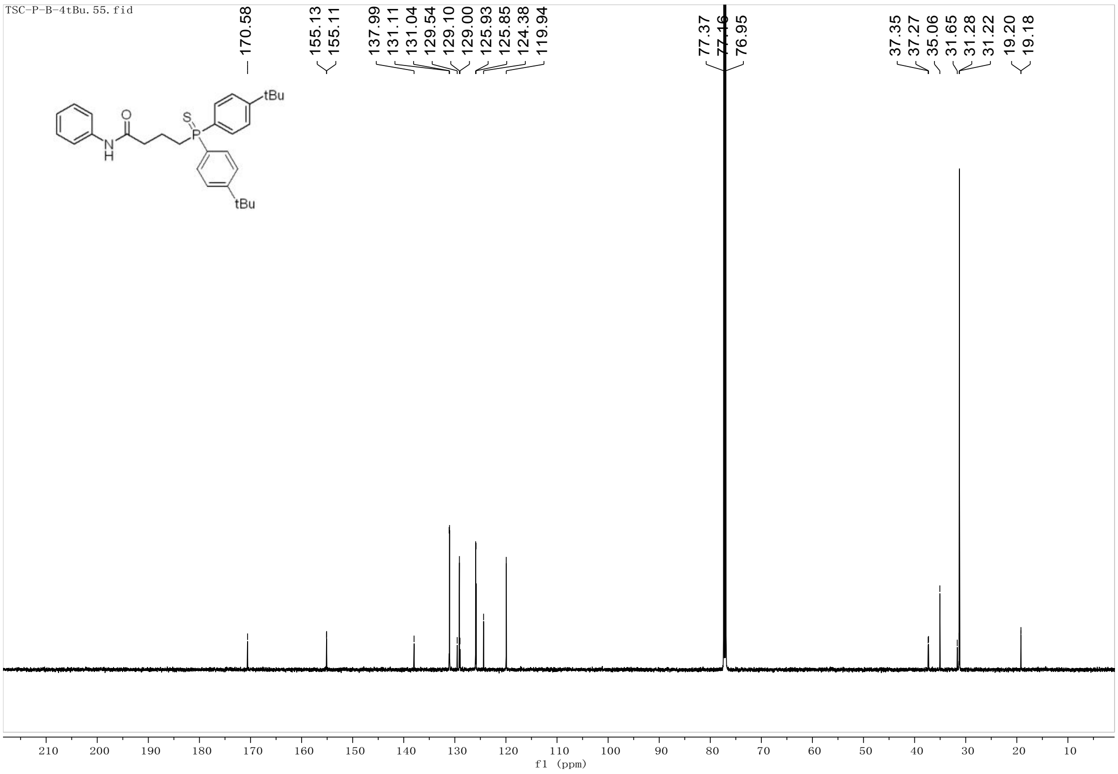
TSC-P-B-4tBu.55.fid
170.58
155.13
155.11
137.99
131.11
131.04
129.54
129.10
129.00
125.93
125.85
124.38
119.94
77.37
77.16
76.95
37.35
37.27
35.06
31.65
31.28
31.22
19.20
19.18
tBu
S
O
P
N
H
tBu
210 200 190 180 170 160 150 140 130 120 110 100 90 80 70 60 50 40 30 20 10
f1 (ppm)

4-(bis(3-methoxyphenyl)phosphorothioyl)-N-phenylbutanamide (4ac)

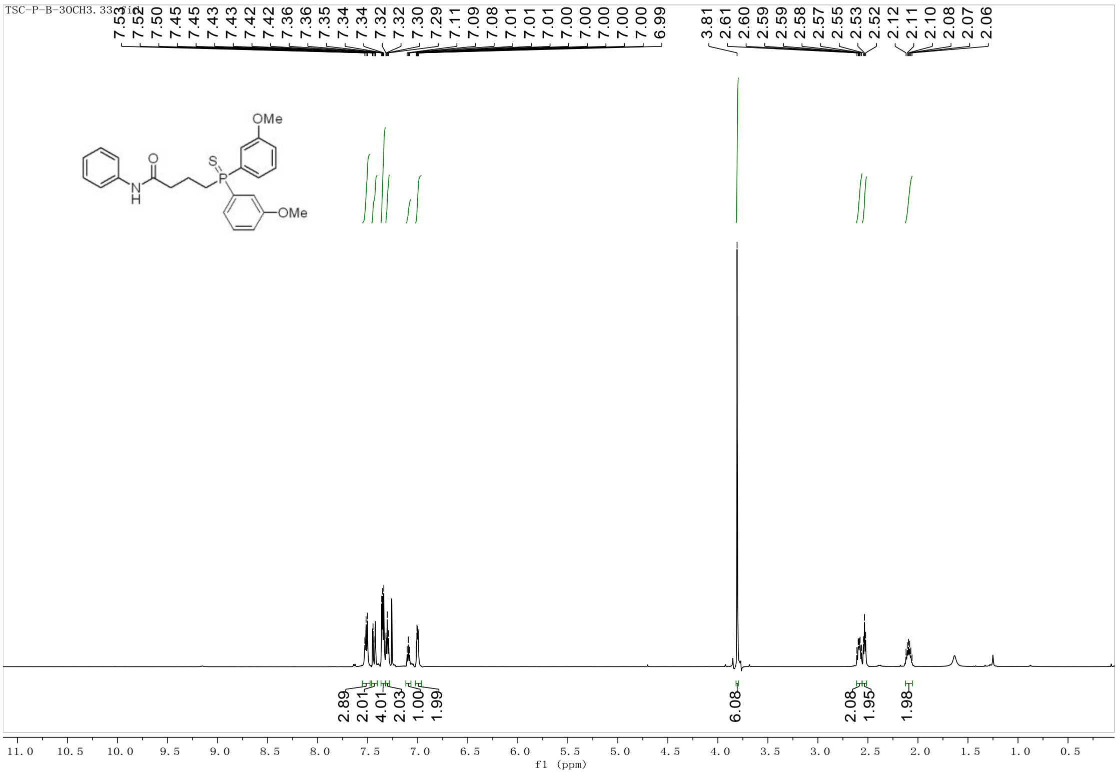

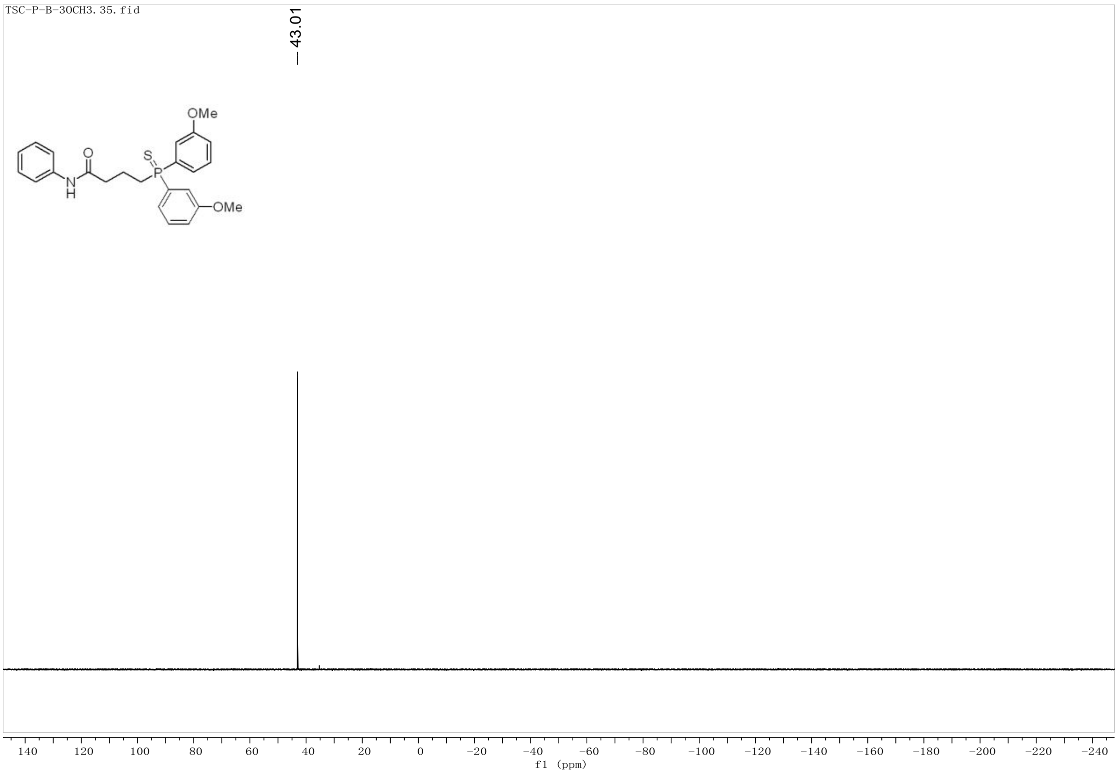

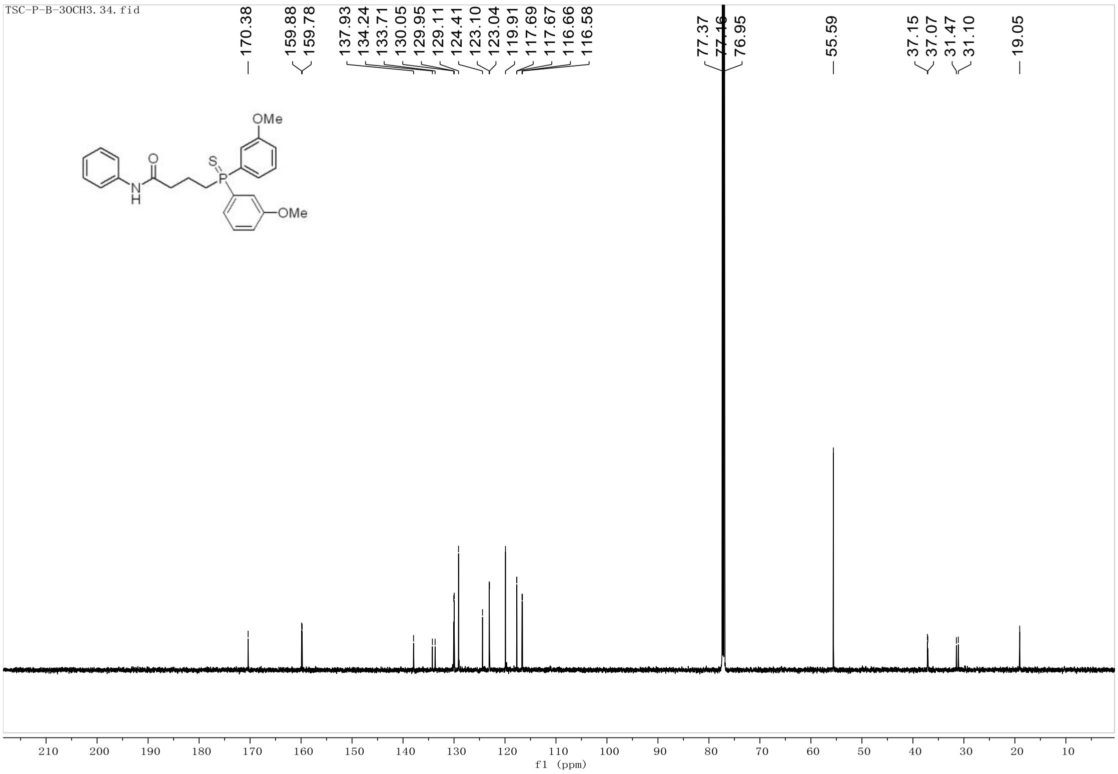

4-(bis(4-fluorophenyl)phosphorothioyl)-N-phenylbutanamide (4ad)

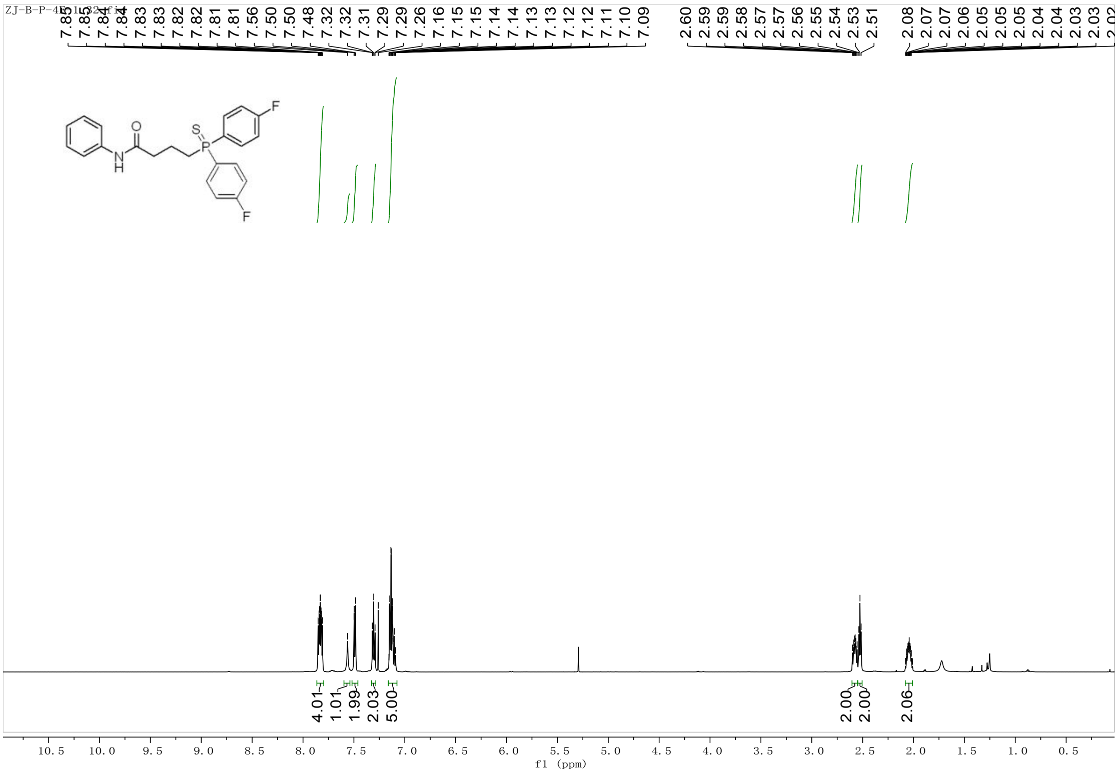

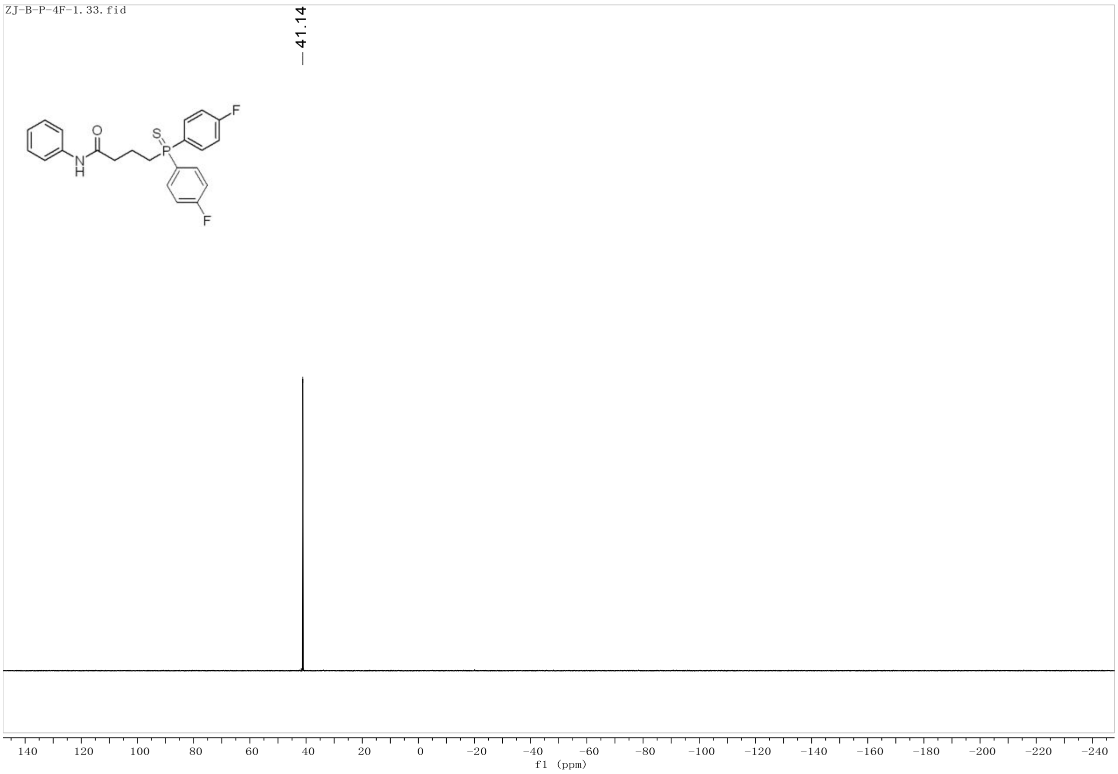
ZJ-B-P-4F-1.33.fid
41.14
140 120 100 80 60 40 20 0 −20 −40 −60 −80 −100 −120 −140 −160 −180 −200 −220 −240
f1 (ppm)

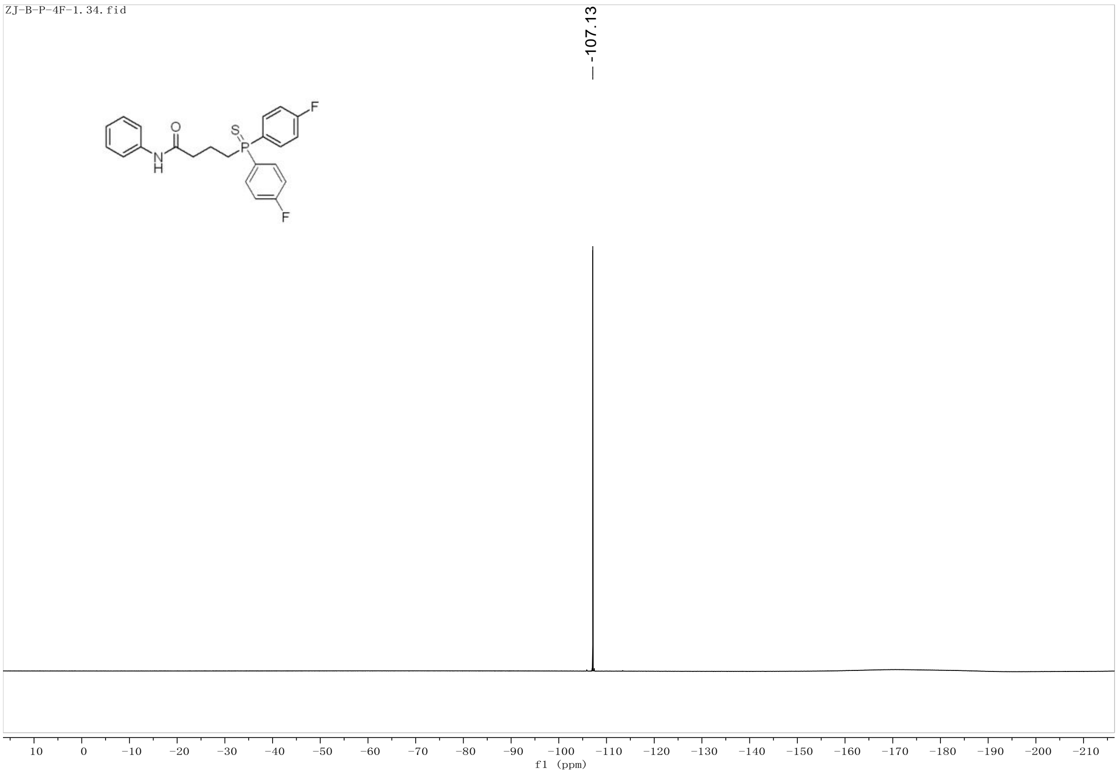
ZJ-B-P-4F-1.34.fid
-107.13
10 0 −10 −20 −30 −40 −50 −60 −70 −80 −90 −100 −110 −120 −130 −140 −150 −160 −170 −180 −190 −200 −210
f1 (ppm)

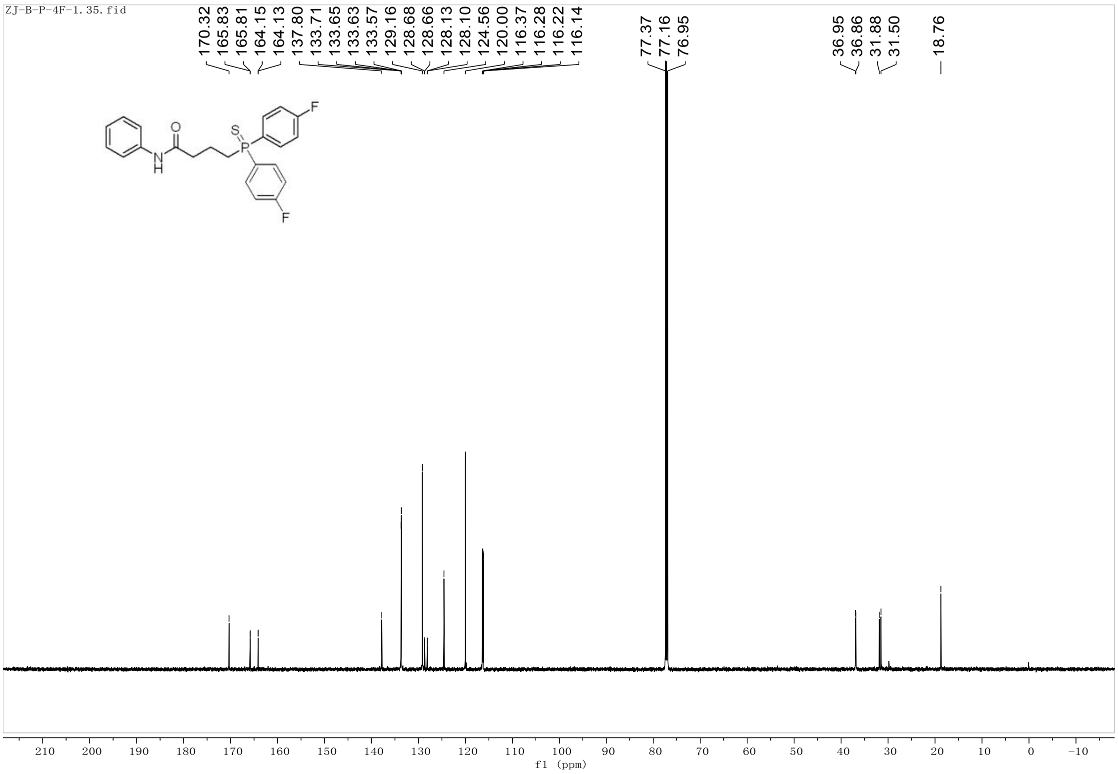

4-(bis(4-chlorophenyl)phosphorothioyl)-N-phenylbutanamide (4ae)

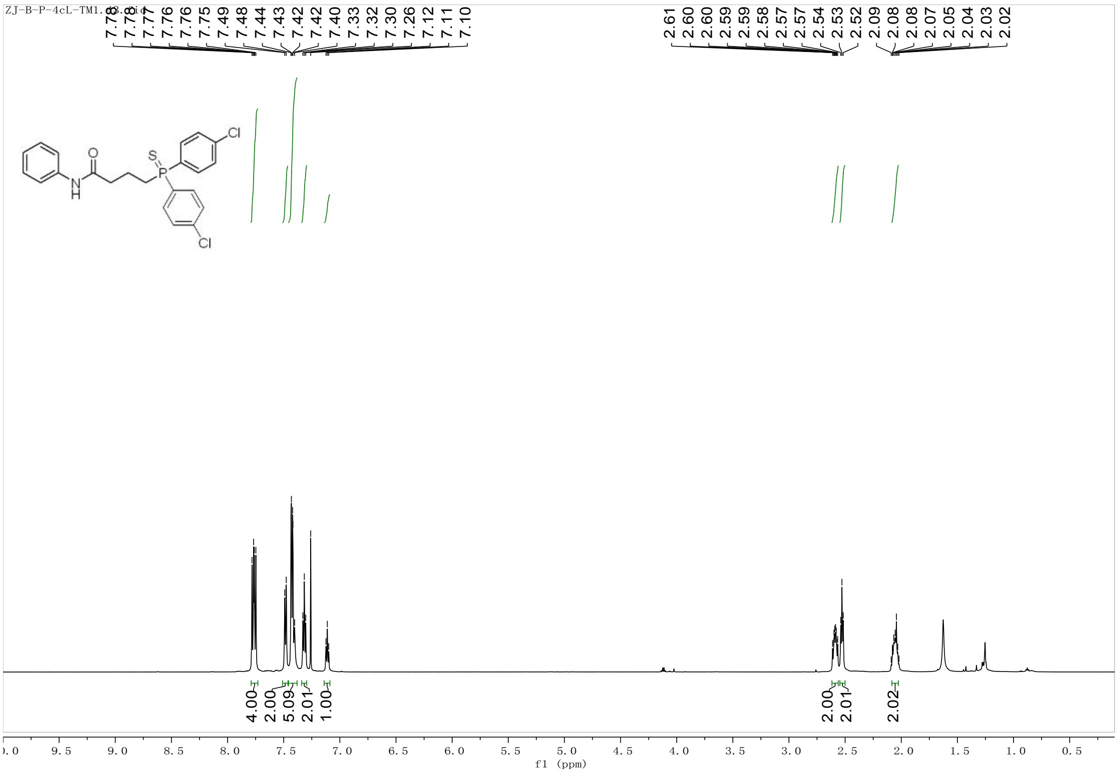

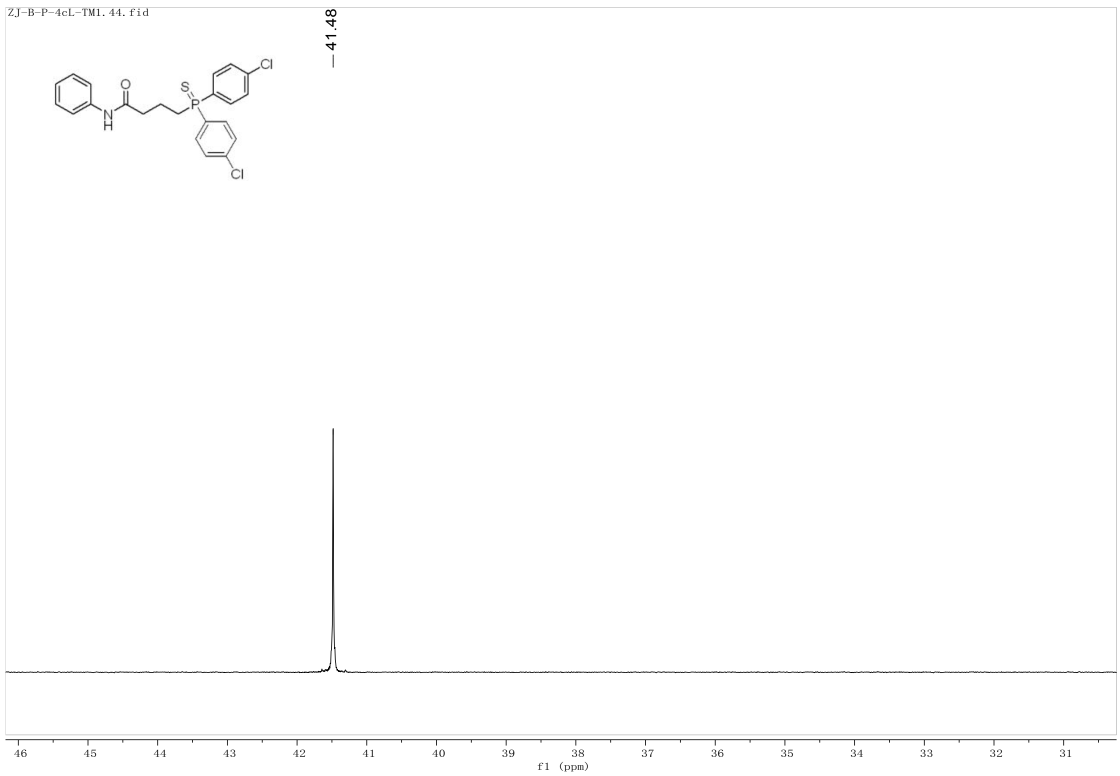
ZJ-B-P-4cL-TM1.44.fid
41.48
S
P
N
H
O
Cl
Cl
46 45 44 43 42 41 40 39 38 37 36 35 34 33 32 31
f1 (ppm)

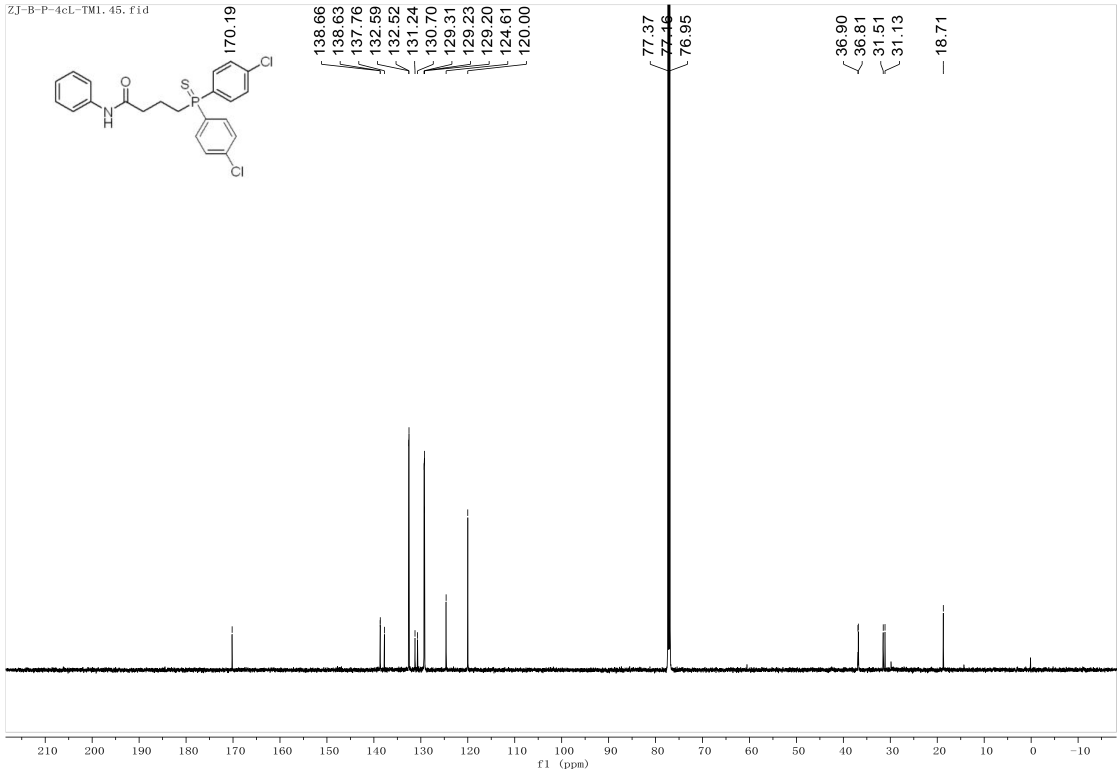
ZJ-B-P-4cL-TM1.45.fid
170.19
138.66
138.63
137.76
132.59
132.52
131.24
130.70
129.31
129.23
129.20
124.61
120.00
77.37
77.16
76.95
36.90
36.81
31.51
31.13
18.71
S
P
N
H
O
Cl
Cl
210 200 190 180 170 160 150 140 130 120 110 100 90 80 70 60 50 40 30 20 10 0 -10
f1 (ppm)

4-(bis(3-chlorophenyl)phosphorothioyl)-N-phenylbutanamide (4af)

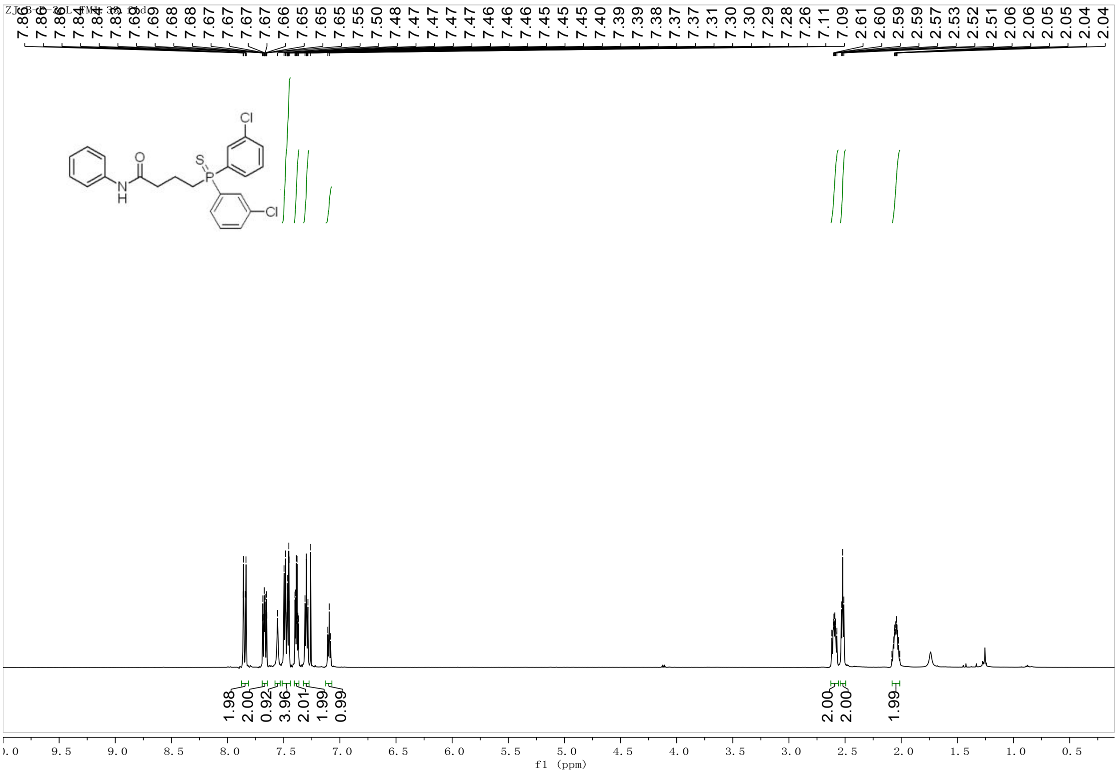

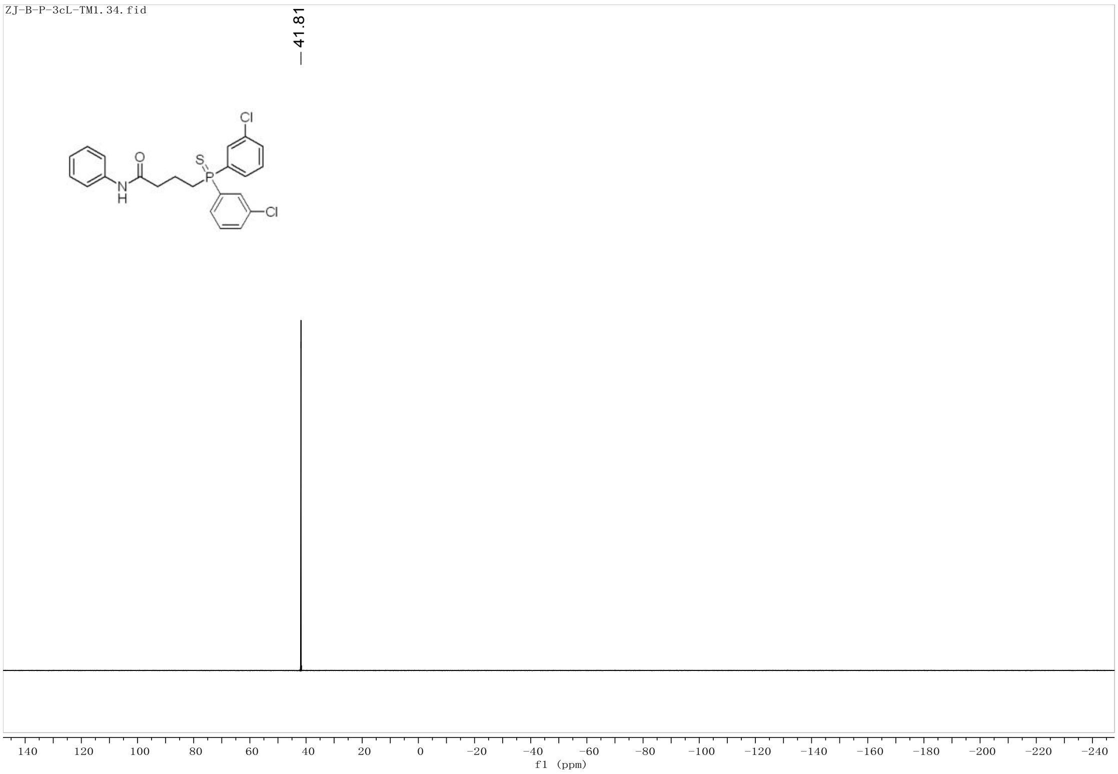

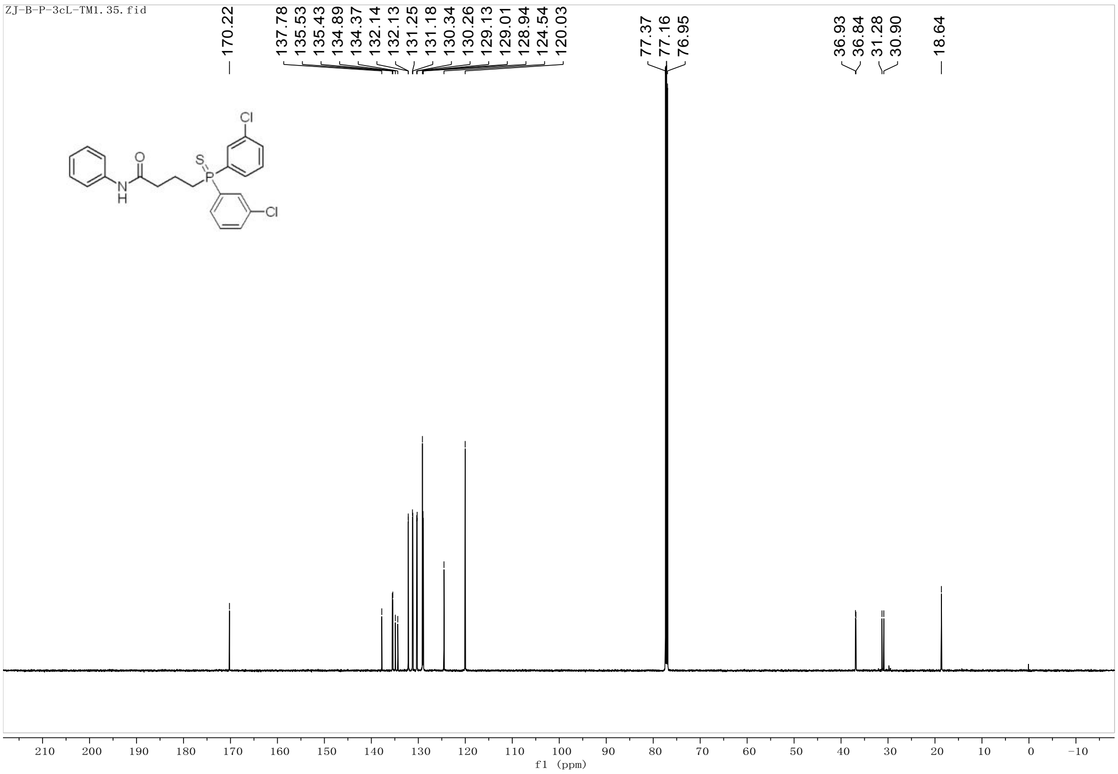

4-(bis(4-(trifluoromethoxy)phenyl)phosphorothioyl)-N-phenylbutanamide (4ag)

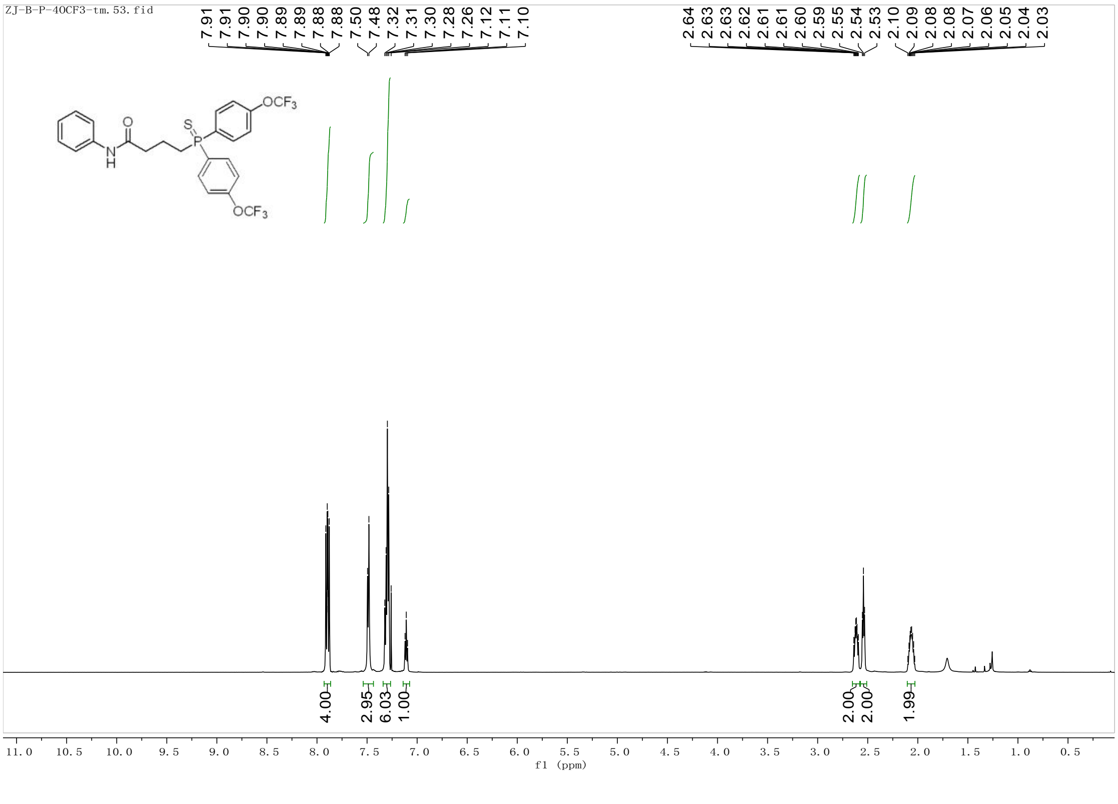

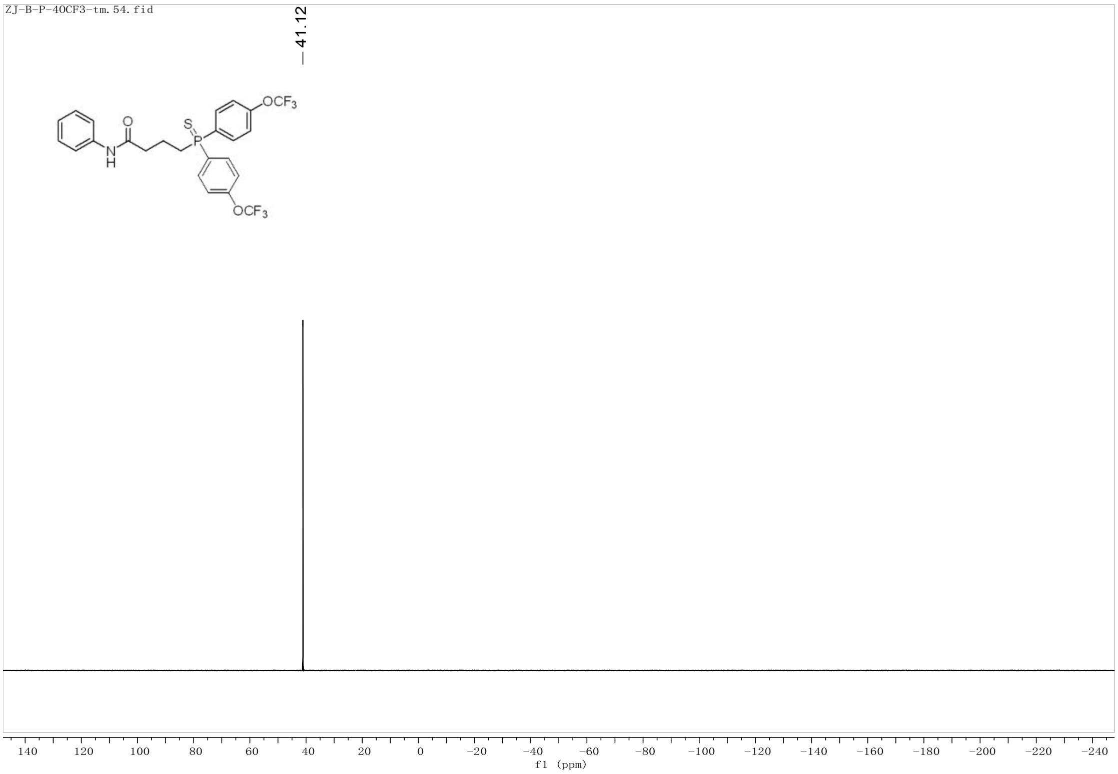
ZJ-B-P-4OCF3-tm.54.fid
41.12
OCF3
S
P
O
N
H
OCF3
f1 (ppm)

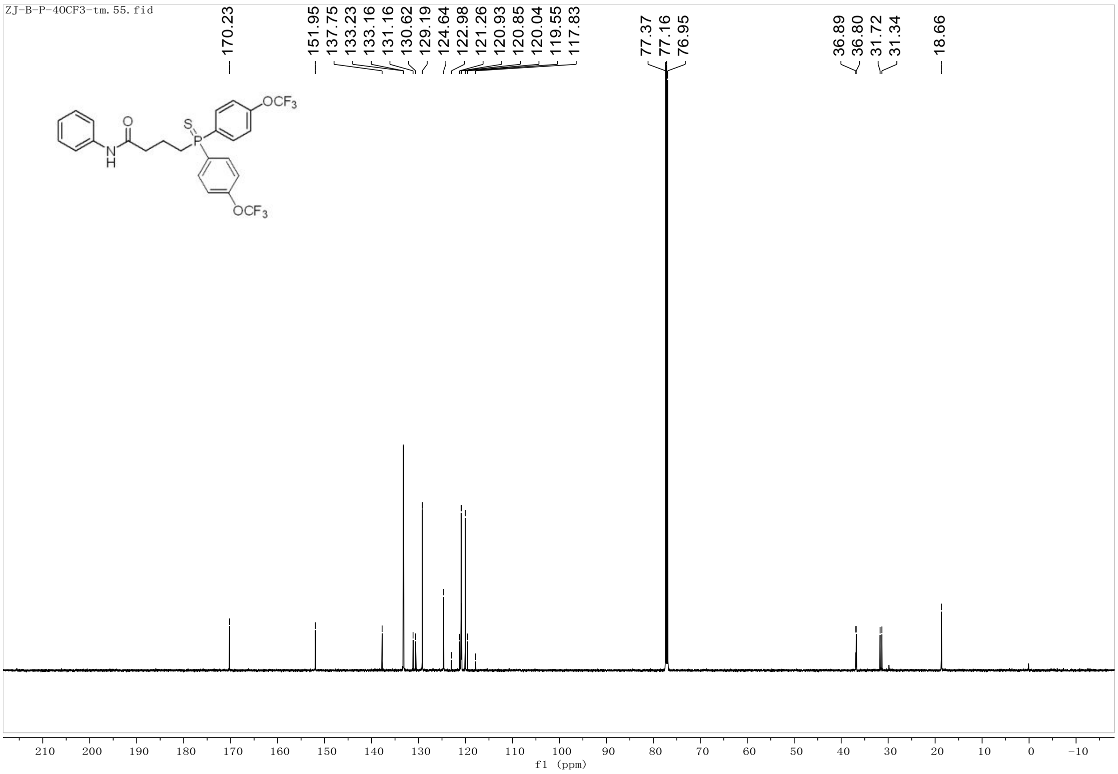
ZJ-B-P-4OCF3-tm.55.fid
170.23
151.95
137.75
133.23
133.16
131.16
130.62
129.19
124.64
122.98
121.26
120.93
120.85
120.04
119.55
117.83
77.37
77.16
76.95
36.89
36.80
31.72
31.34
18.66
OCF3
S
P
O
N
H
OCF3
f1 (ppm)

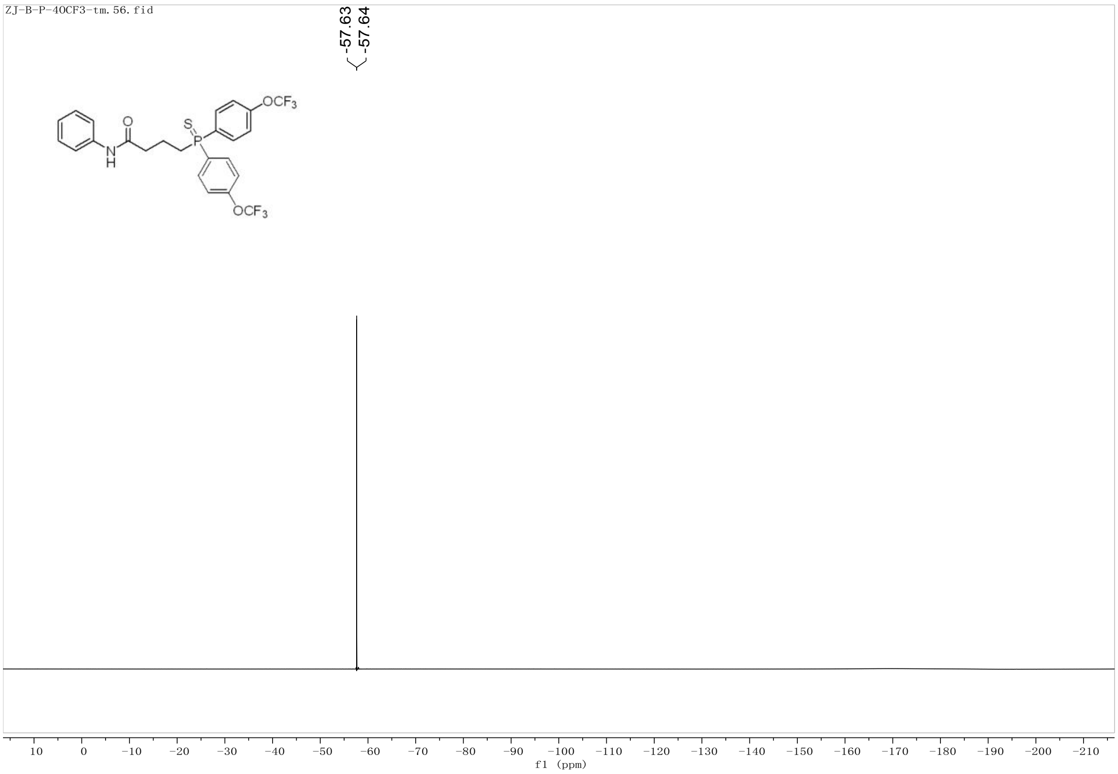

4-(di(naphthalen-2-yl)phosphorothioyl)-N-phenylbutanamide (4ah)

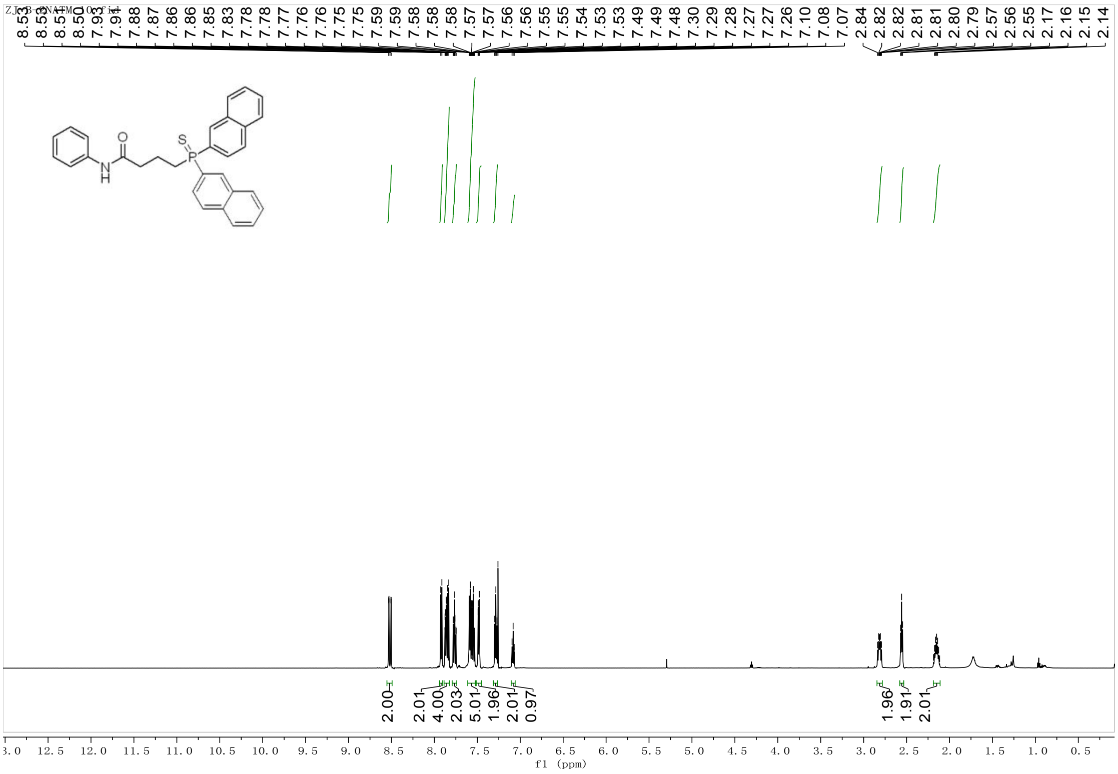

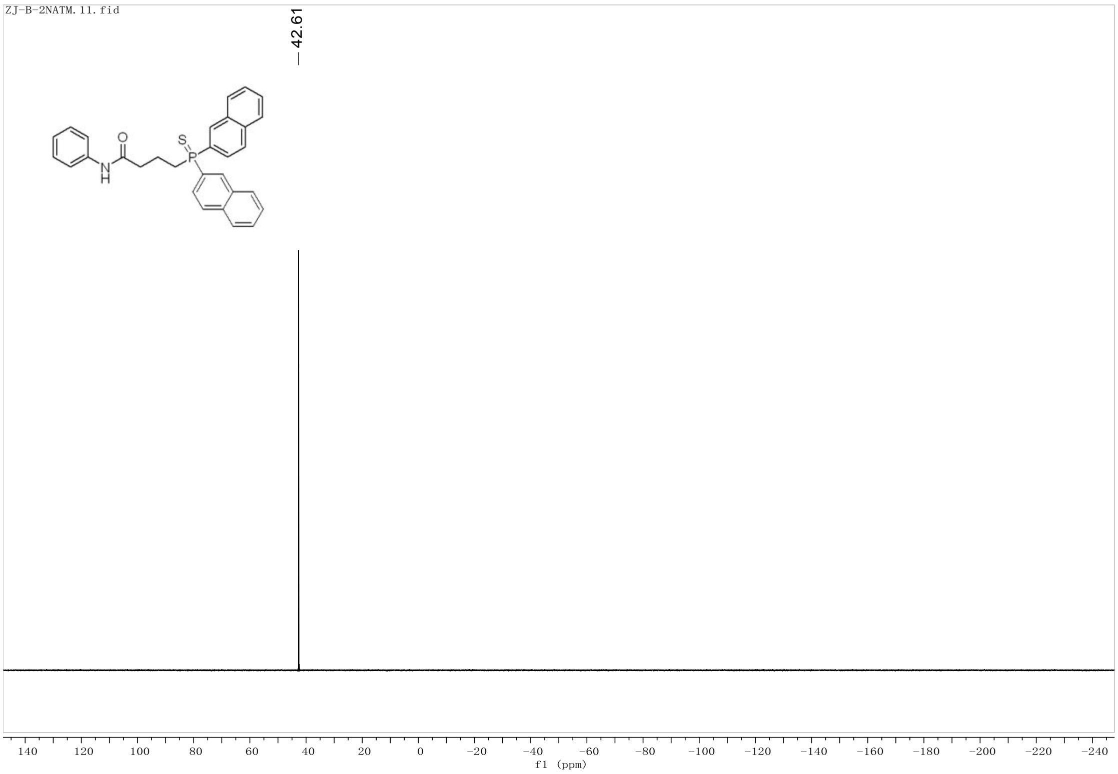
ZJ-B-2NATM.11.fid
42.61
f1 (ppm)

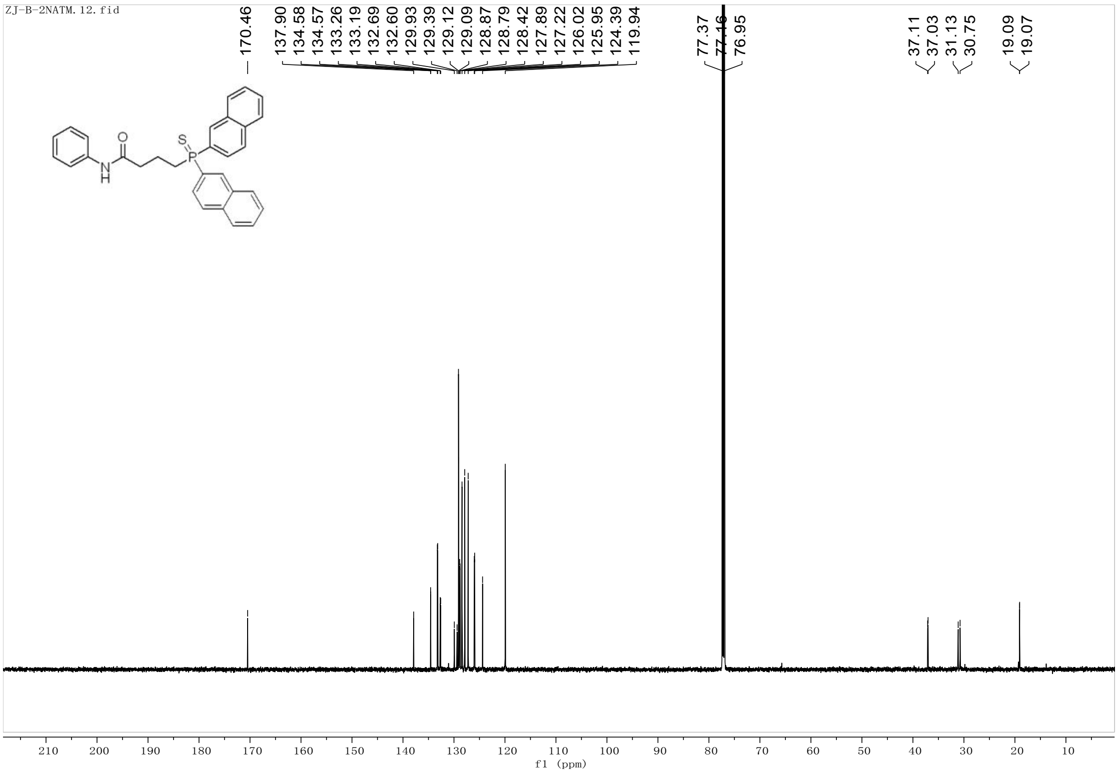
ZJ-B-2NATM.12.fid
170.46
137.90
134.58
134.57
133.26
133.19
132.69
132.60
129.93
129.39
129.12
129.09
128.87
128.79
128.42
127.89
127.22
126.02
125.95
124.39
119.94
77.37
77.16
76.95
37.11
37.03
31.13
30.75
19.09
19.07
f1 (ppm)

(*R*)-diphenyl(4-(phenylamino)butan-2-yl)phosphine sulfide (5)

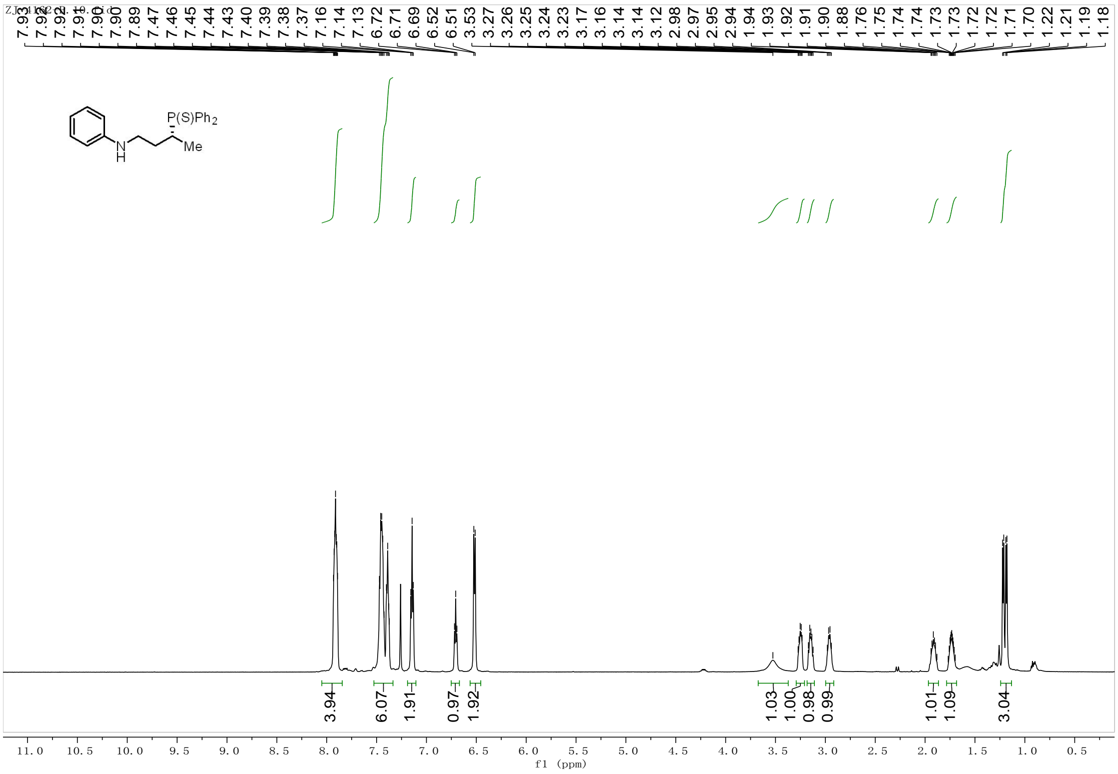

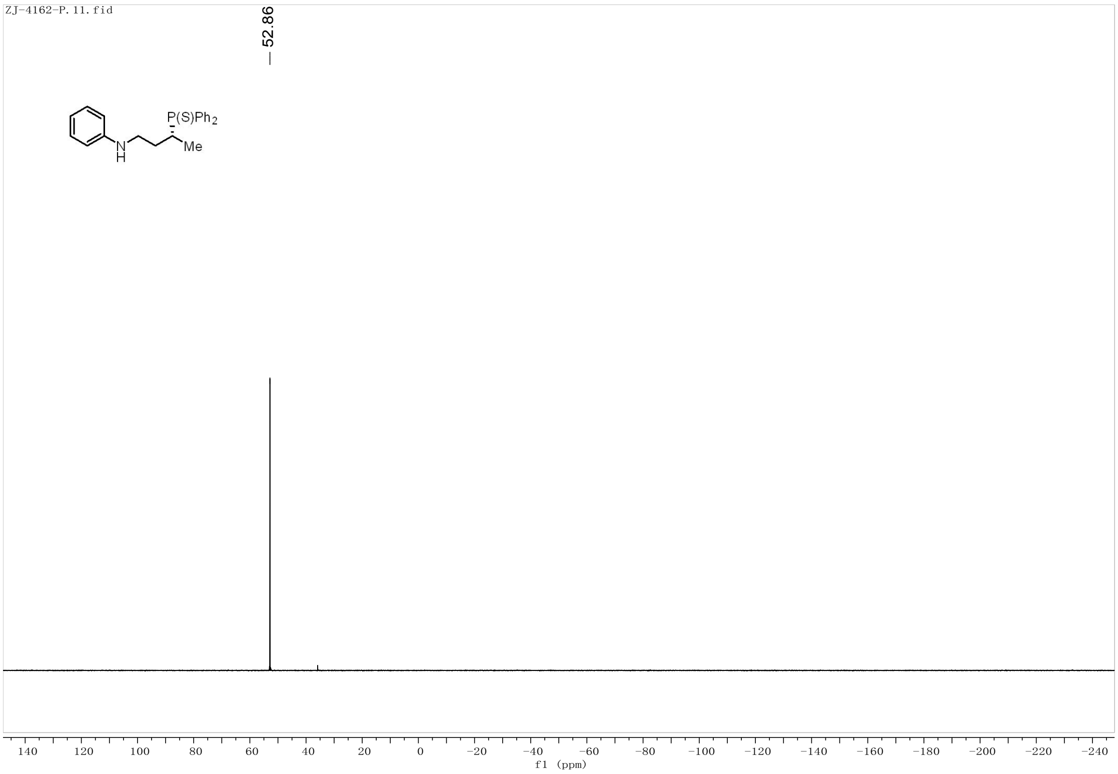

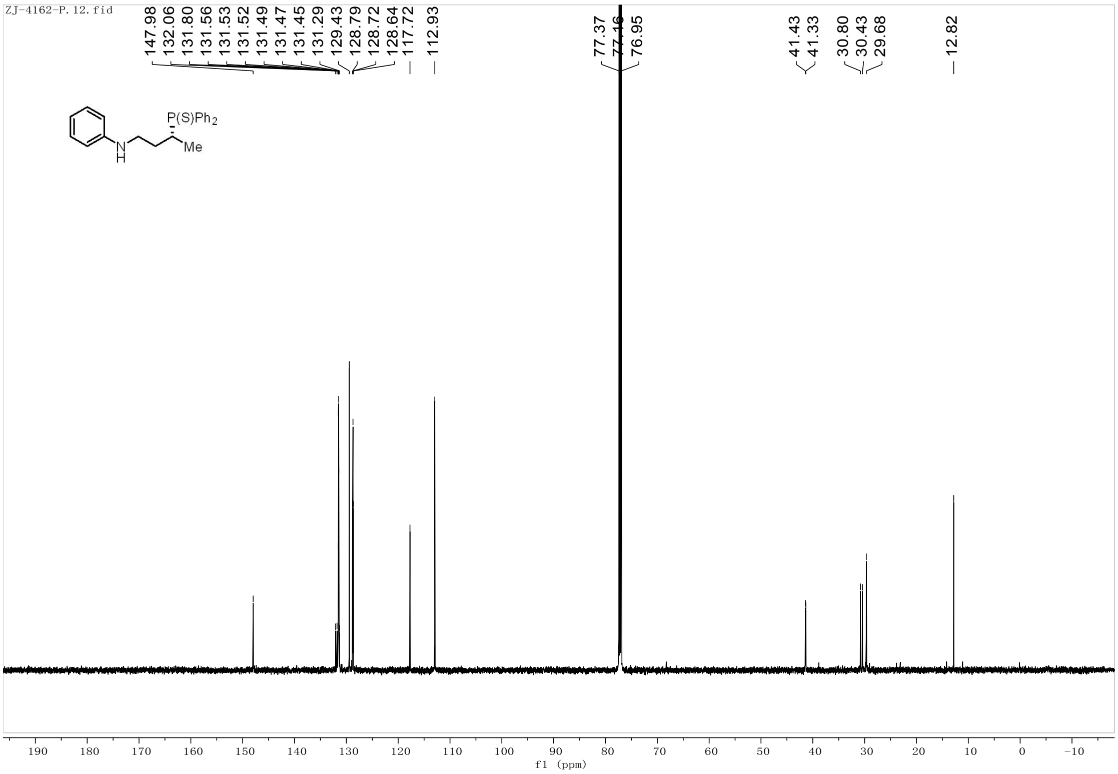

(*R*)-diphenyl(4-(phenyl(pyridin-4-yl)amino)butan-2-yl)phosphine sulfide (6)

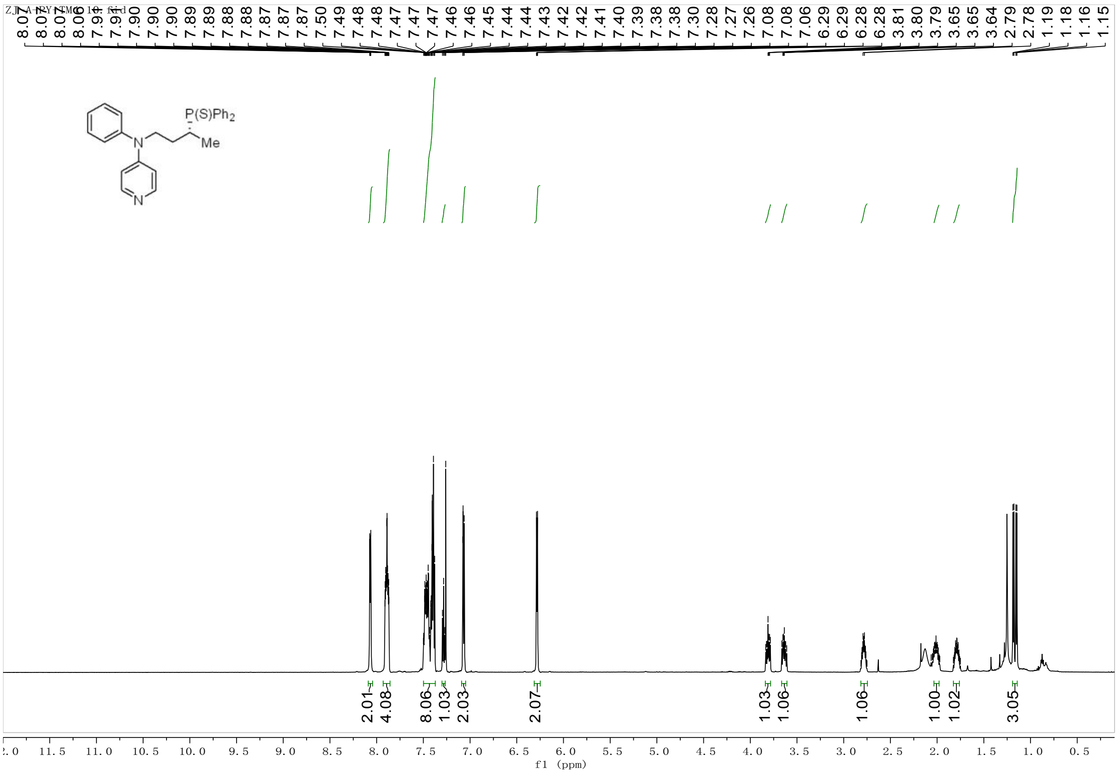

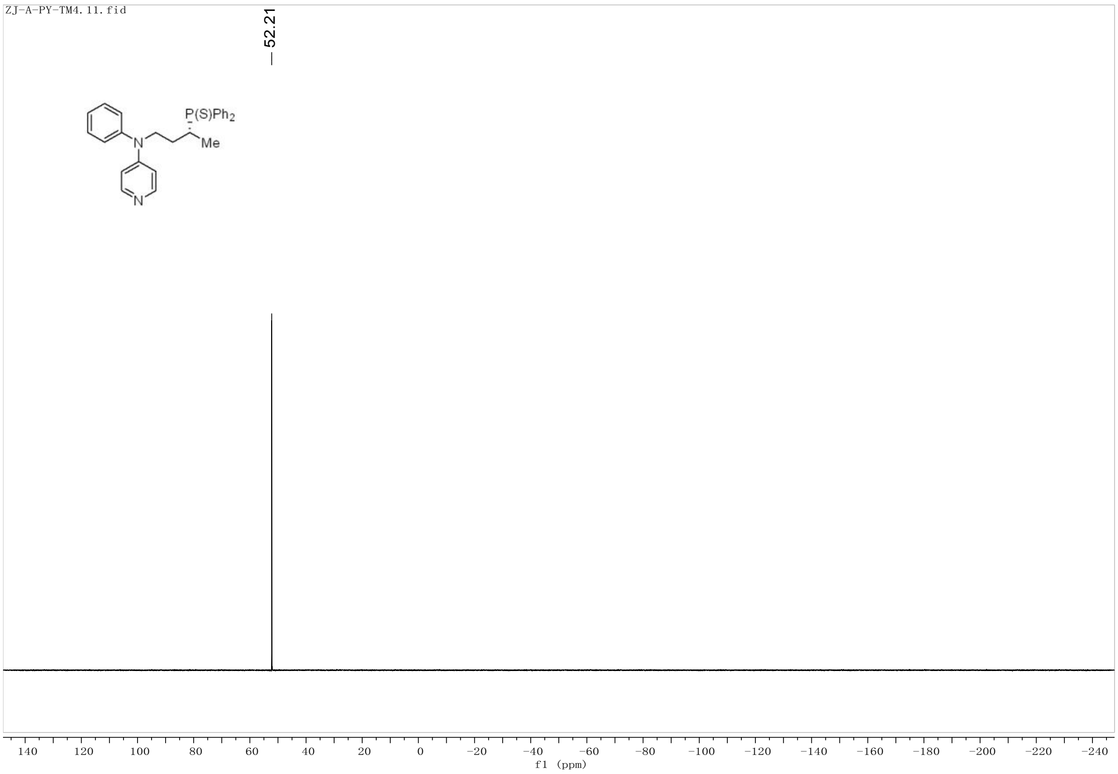
ZJ-A-PY-TM4.11.fid
52.21
P(S)Ph2
N
Me
N
f1 (ppm)

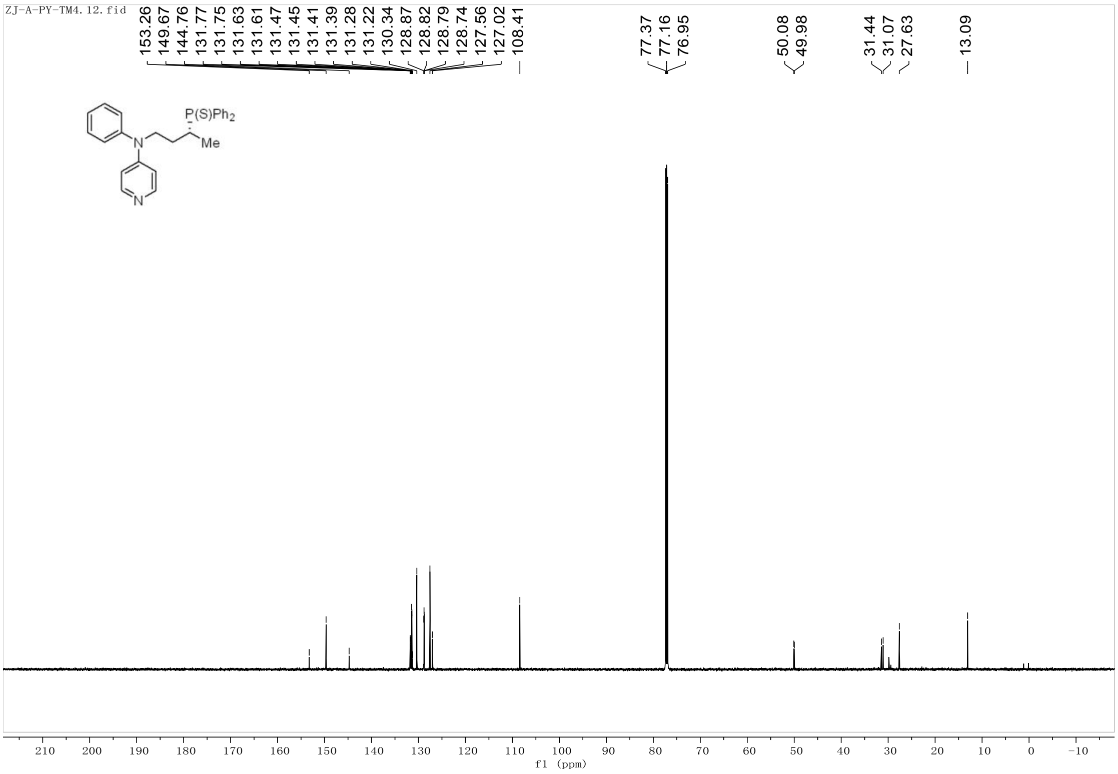
ZJ-A-PY-TM4.12.fid
153.26
149.67
144.76
131.77
131.75
131.63
131.61
131.47
131.45
131.41
131.39
131.28
131.22
130.34
128.87
128.82
128.79
128.74
127.56
127.02
108.41
77.37
77.16
76.95
50.08
49.98
31.44
31.07
27.63
13.09
P(S)Ph2
N
Me
N
f1 (ppm)

(*R*)-1-(3-(diphenylphosphorothioyl)butyl)-1-phenyl-3-(4-(trifluoromethyl)phenyl) thiourea (7)

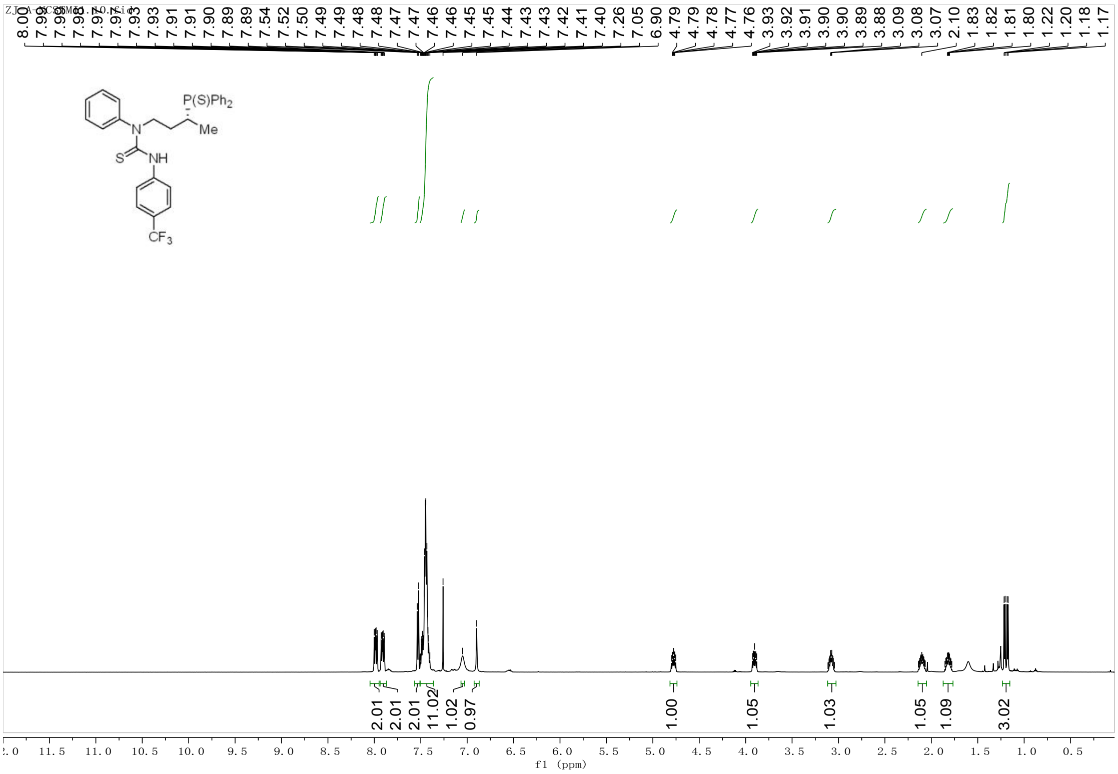

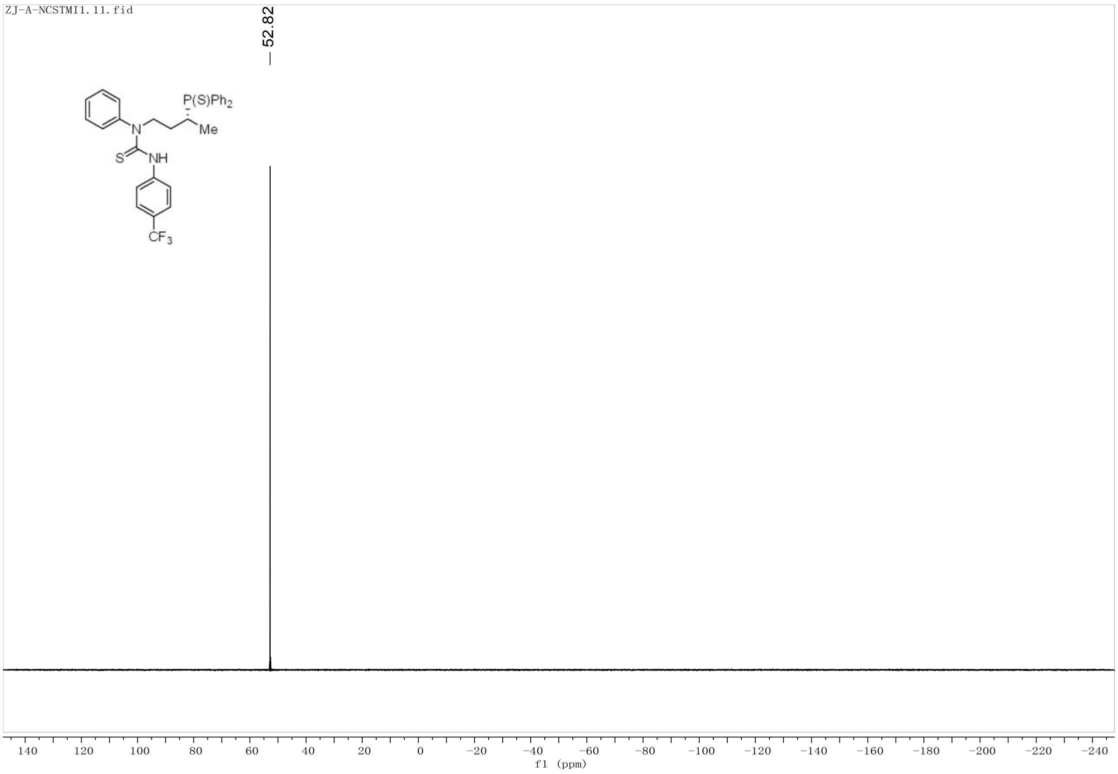

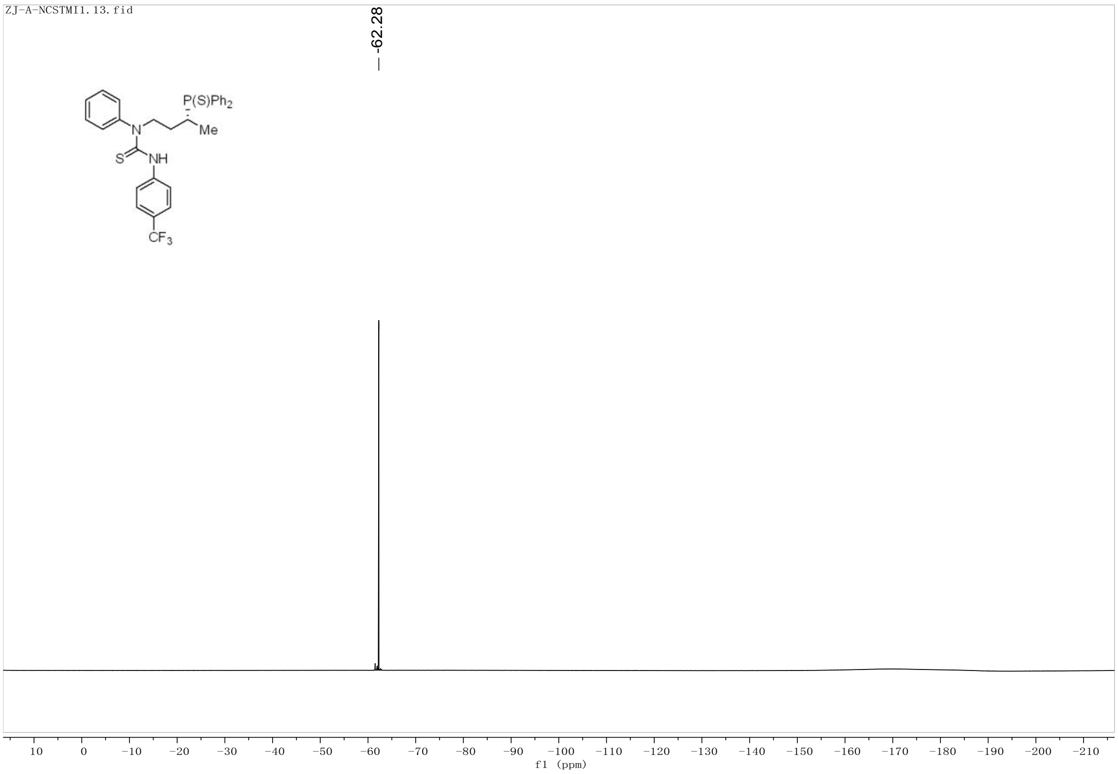
ZJ-A-NCSTMI1.13.fid
-62.28
P(S)Ph2
Me
N
S
NH
CF3
f1 (ppm)

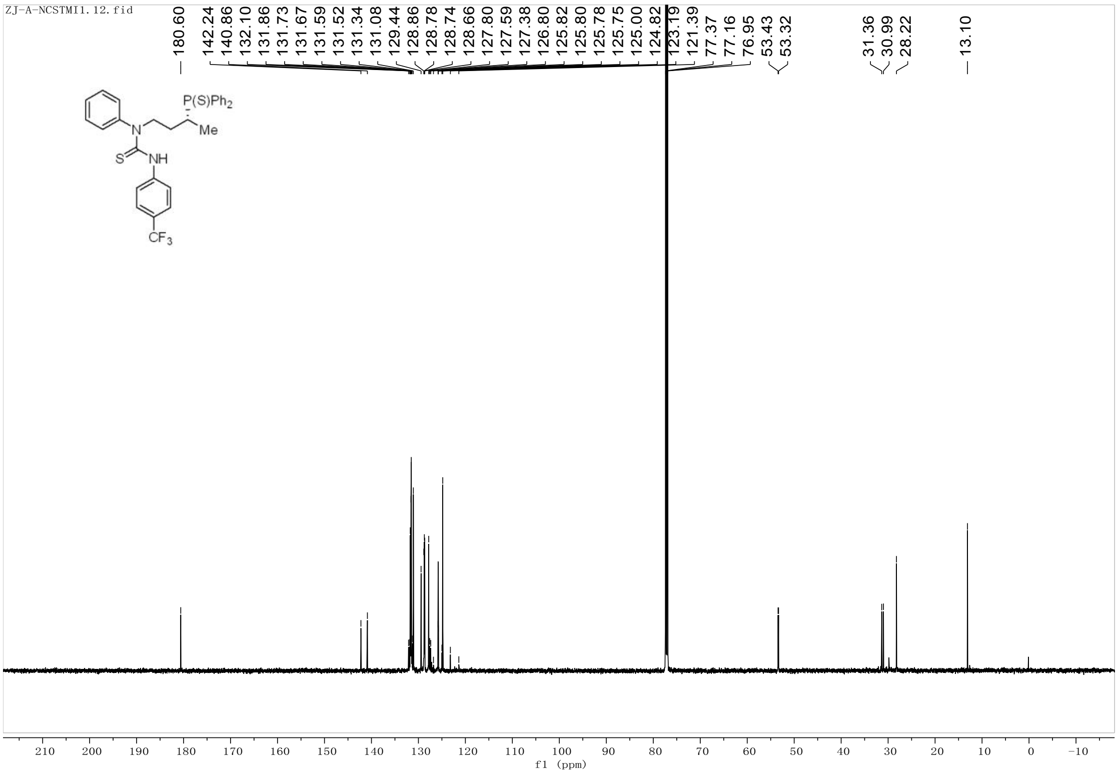
ZJ-A-NCSTMI1.12.fid
180.60
142.24
140.86
132.10
131.86
131.73
131.67
131.59
131.52
131.34
131.08
129.44
128.86
128.78
128.74
128.66
127.80
127.59
127.38
126.80
125.82
125.80
125.78
125.75
125.00
124.82
123.19
121.39
77.37
77.16
76.95
53.43
53.32
31.36
30.99
28.22
13.10
P(S)Ph2
Me
N
S
NH
CF3
f1 (ppm)

diphenyl(4-(phenylamino)butyl)phosphine sulfide (8)

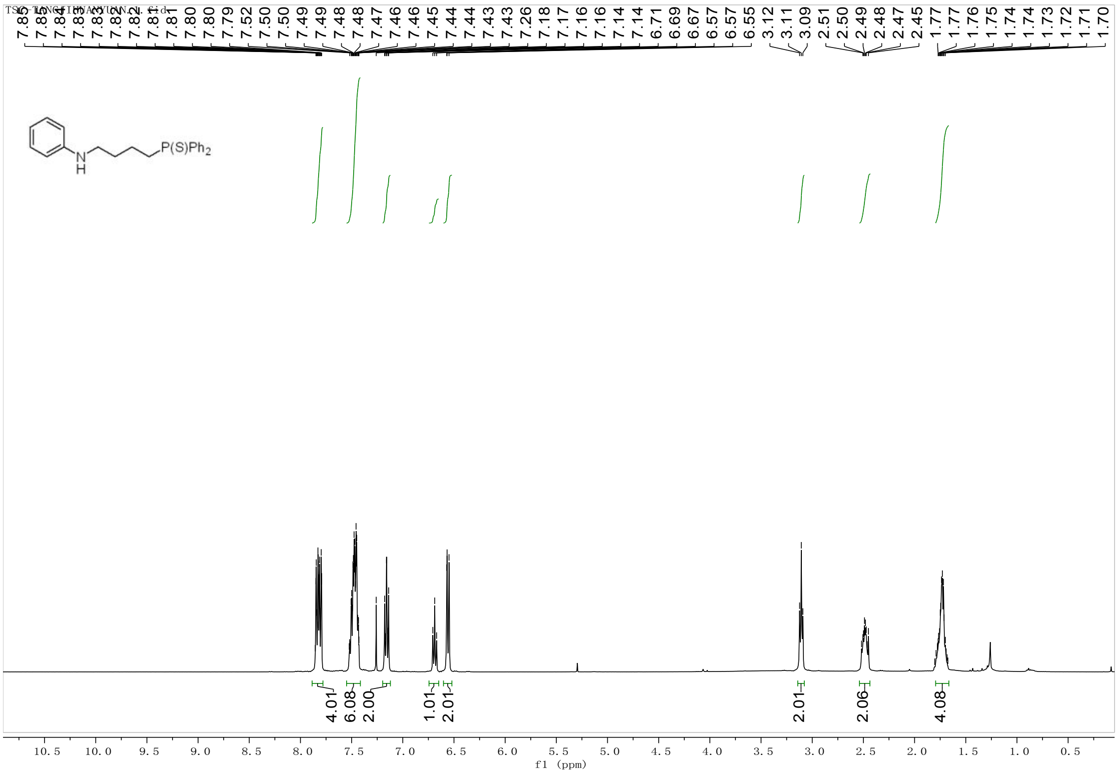

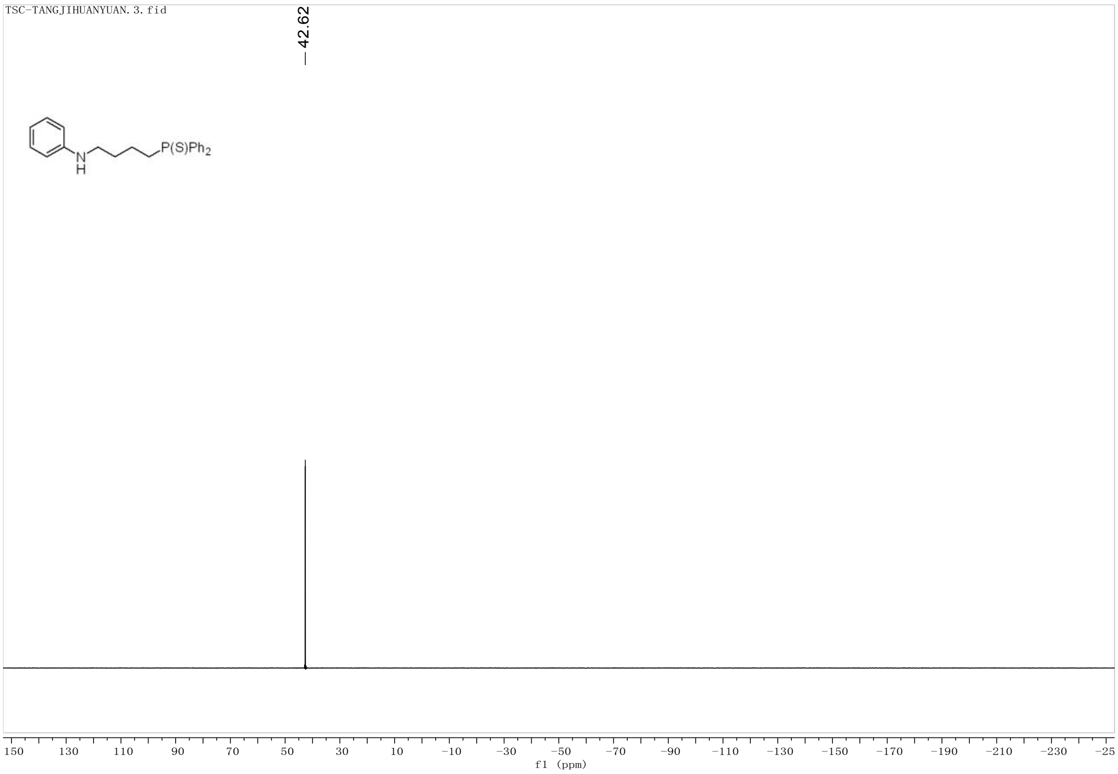

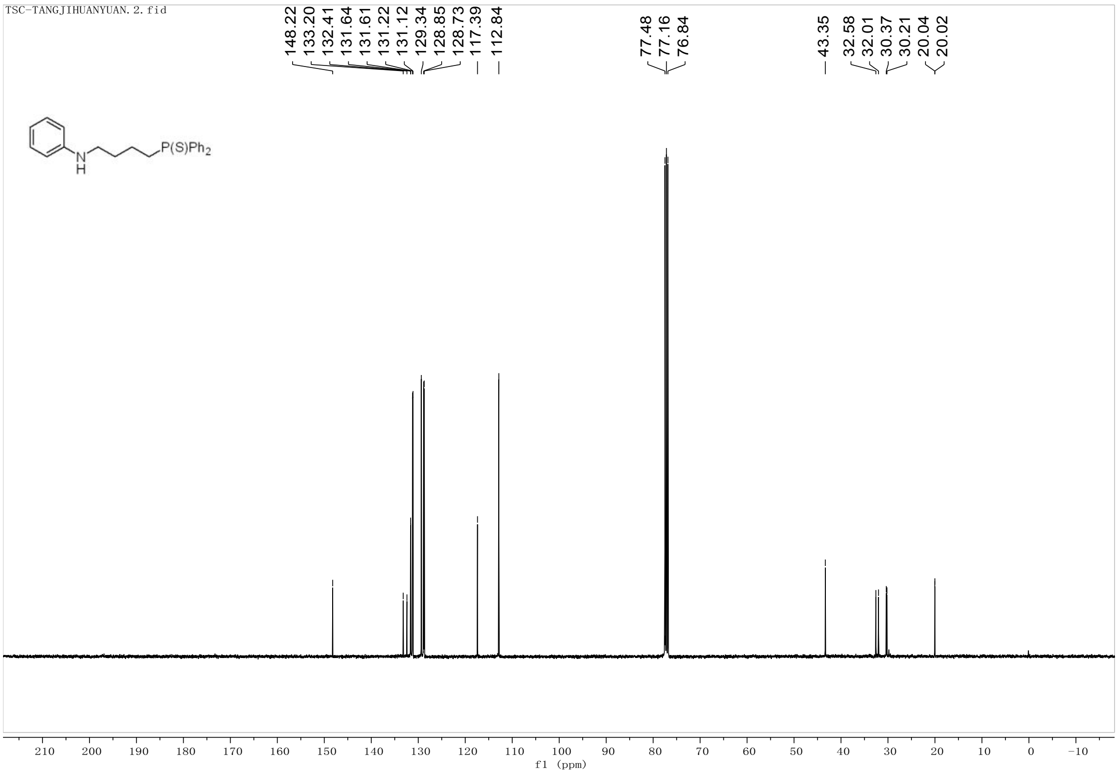

1-(4-(diphenylphosphorothioyl)butyl)-1-phenyl-3-(4-(trifluoromethyl) phenyl) thiourea (9)

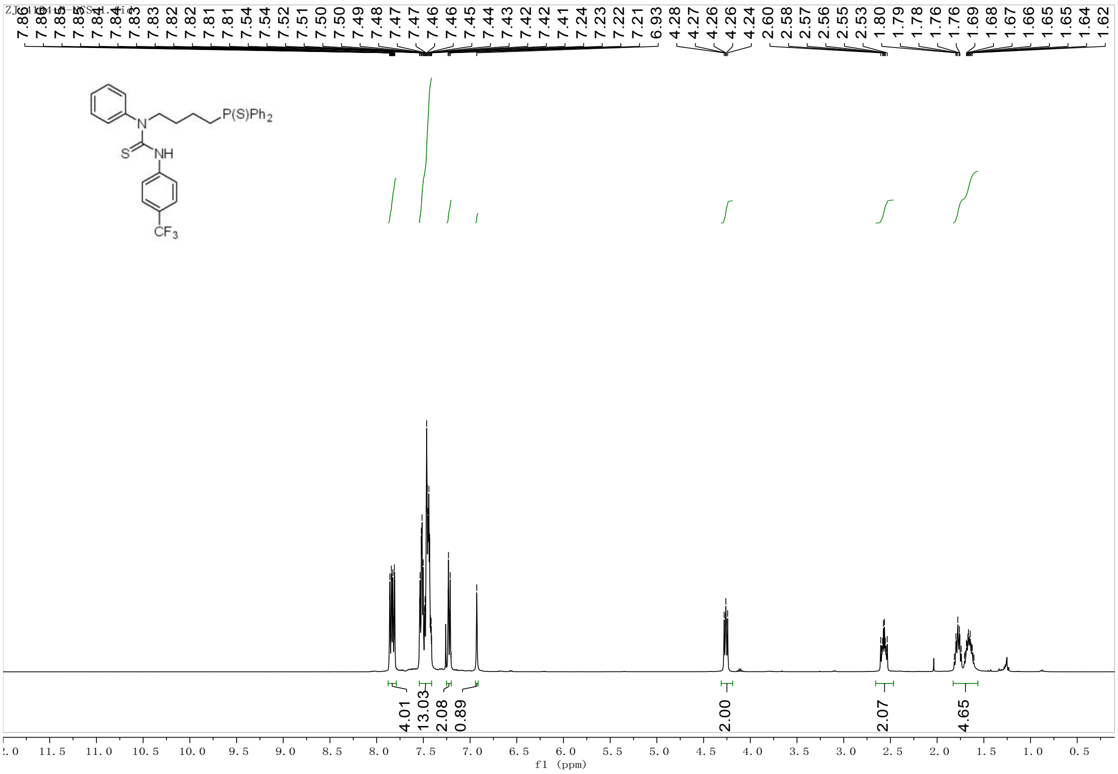

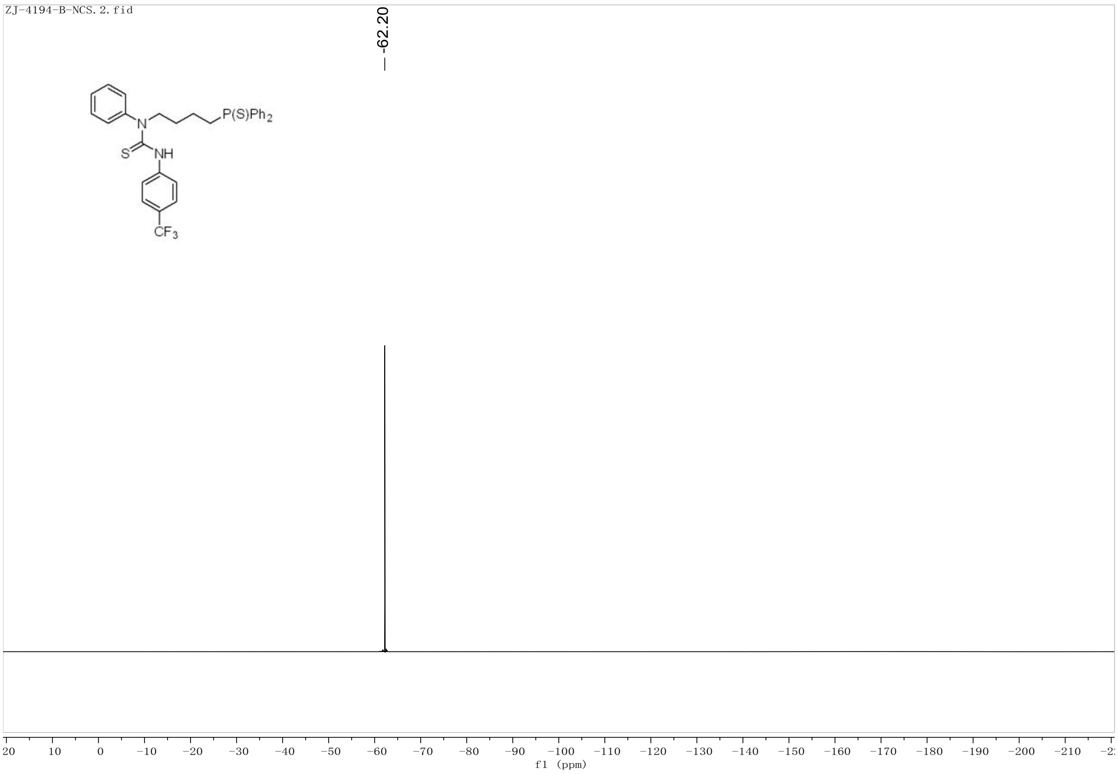
ZJ-4194-B-NCS.2.fid
-62.20
P(S)Ph2
N
S
NH
CF3
f1 (ppm)

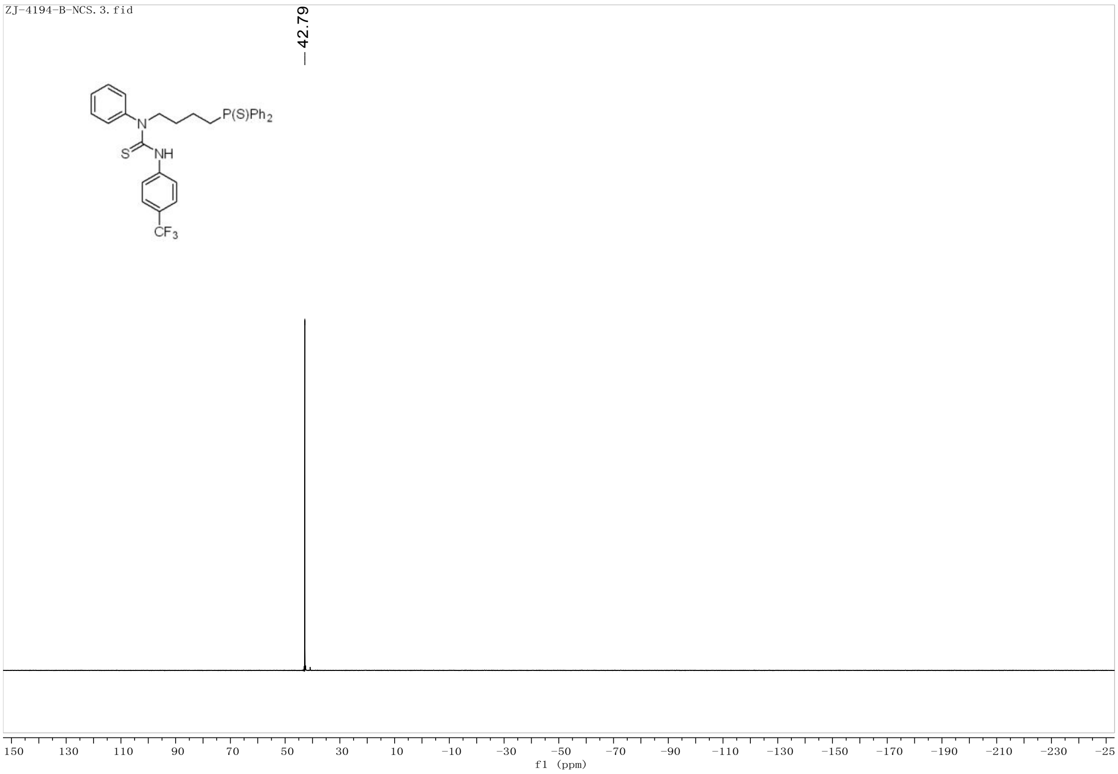
ZJ-4194-B-NCS.3.fid
42.79
P(S)Ph2
N
S
NH
CF3
f1 (ppm)

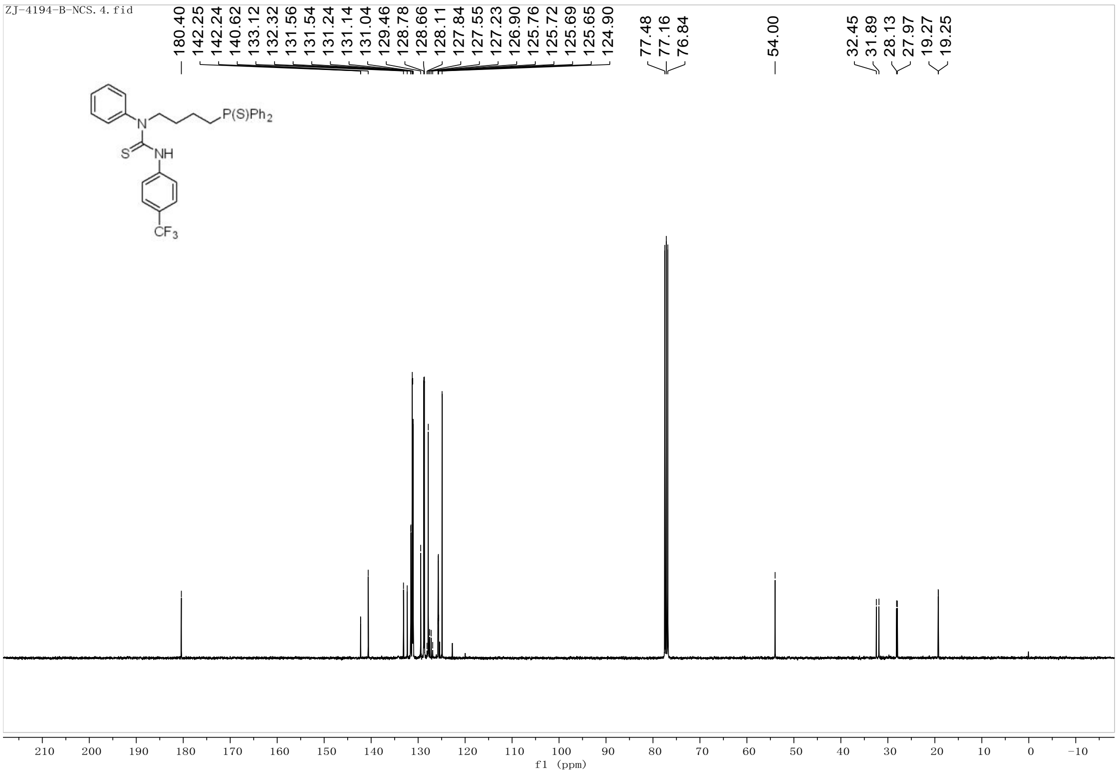

diphenyl(4-(phenyl(pyridin-4-yl)amino)butyl)phosphine sulfide (10)

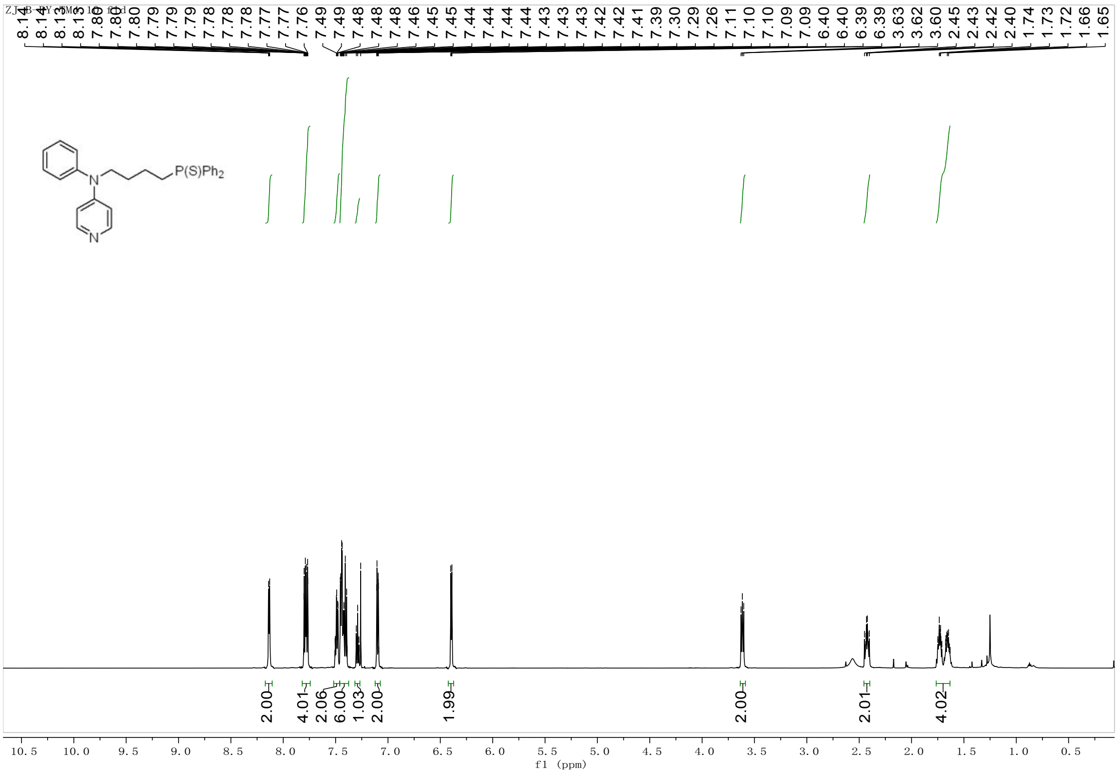

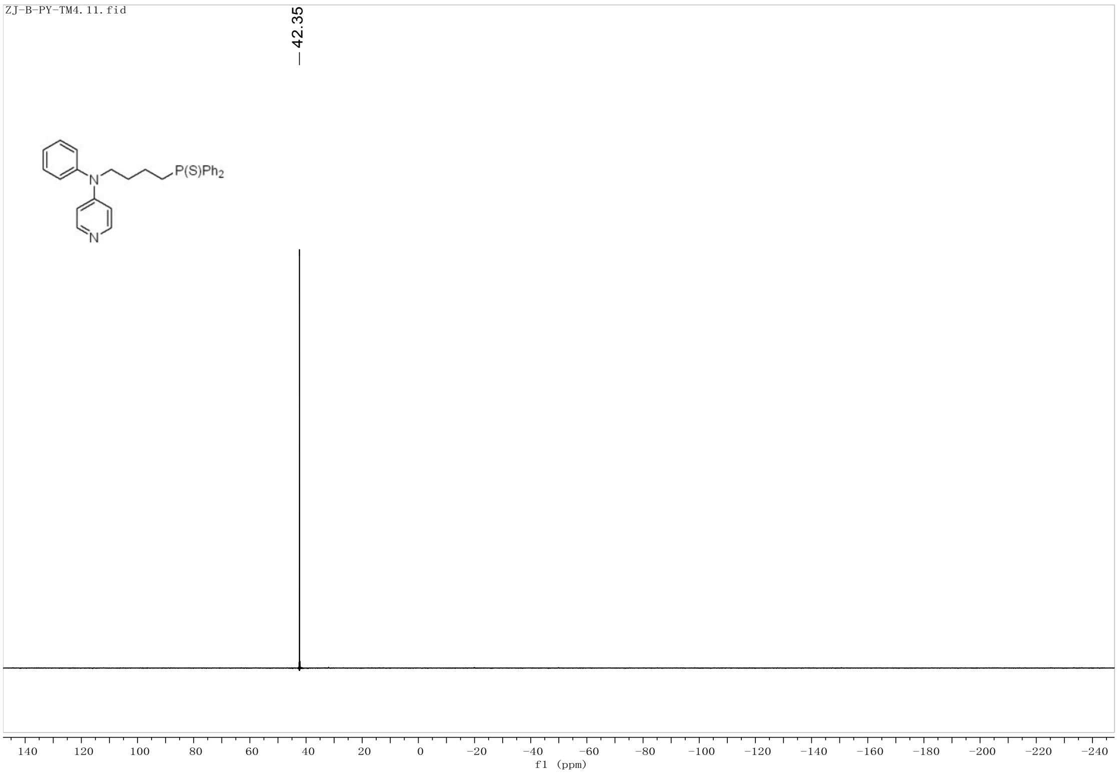
ZJ-B-PY-TM4.11.fid
42.35
P(S)Ph2
N
N
140 120 100 80 60 40 20 0 −20 −40 −60 −80 −100 −120 −140 −160 −180 −200 −220 −240
f1 (ppm)

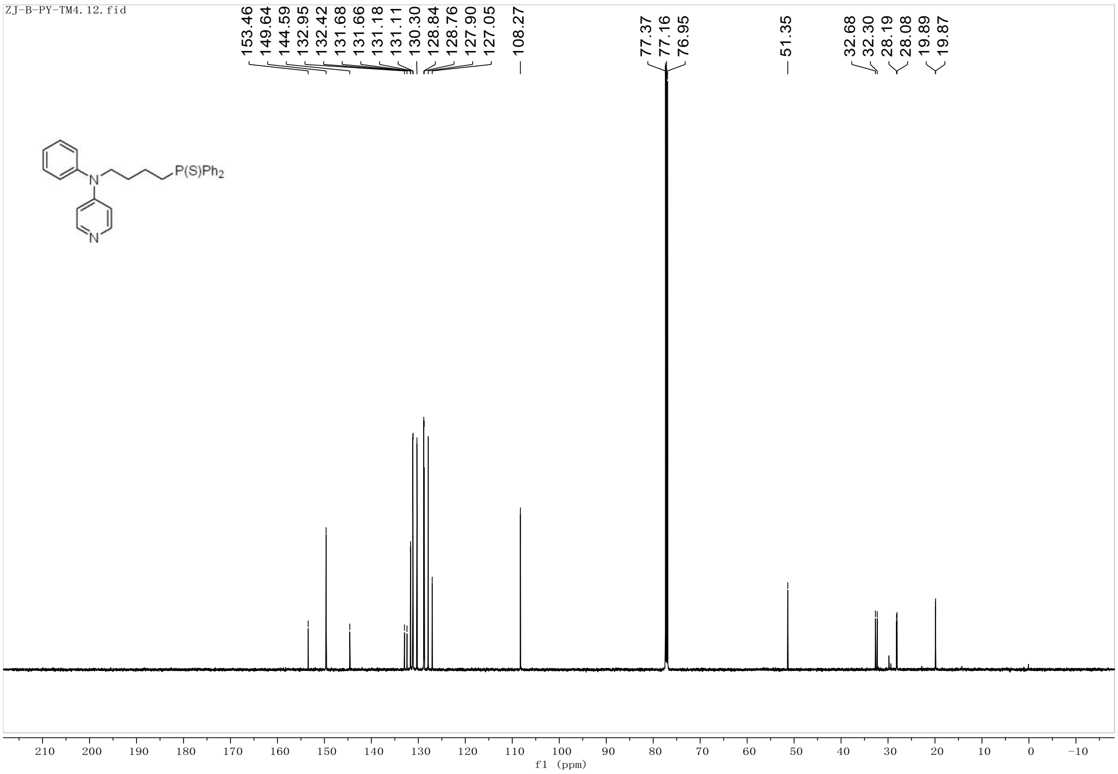
ZJ-B-PY-TM4.12.fid
153.46
149.64
144.59
132.95
132.42
131.68
131.66
131.18
131.11
130.30
128.84
128.76
127.90
127.05
108.27
77.37
77.16
76.95
51.35
32.68
32.30
28.19
28.08
19.89
19.87
P(S)Ph2
N
N
210 200 190 180 170 160 150 140 130 120 110 100 90 80 70 60 50 40 30 20 10 0 −10
f1 (ppm)

((*R*)-3-(diphenylphosphorothioyl)butanoyl)-L-alanine (11)

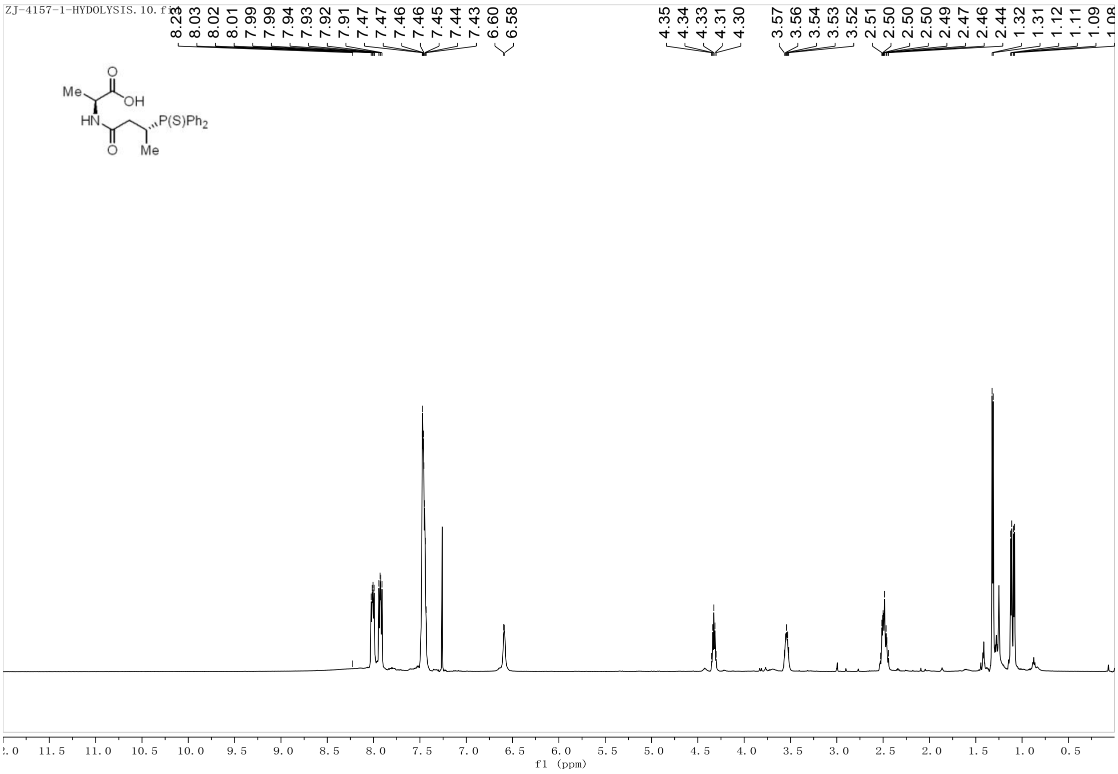

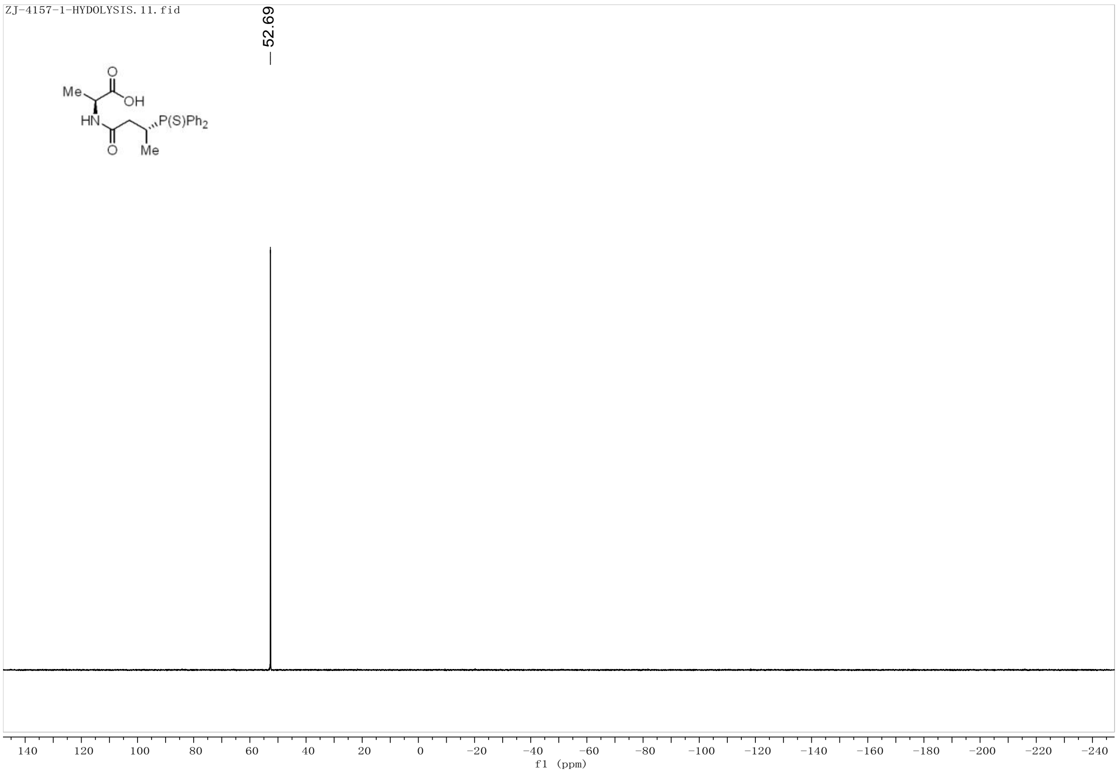

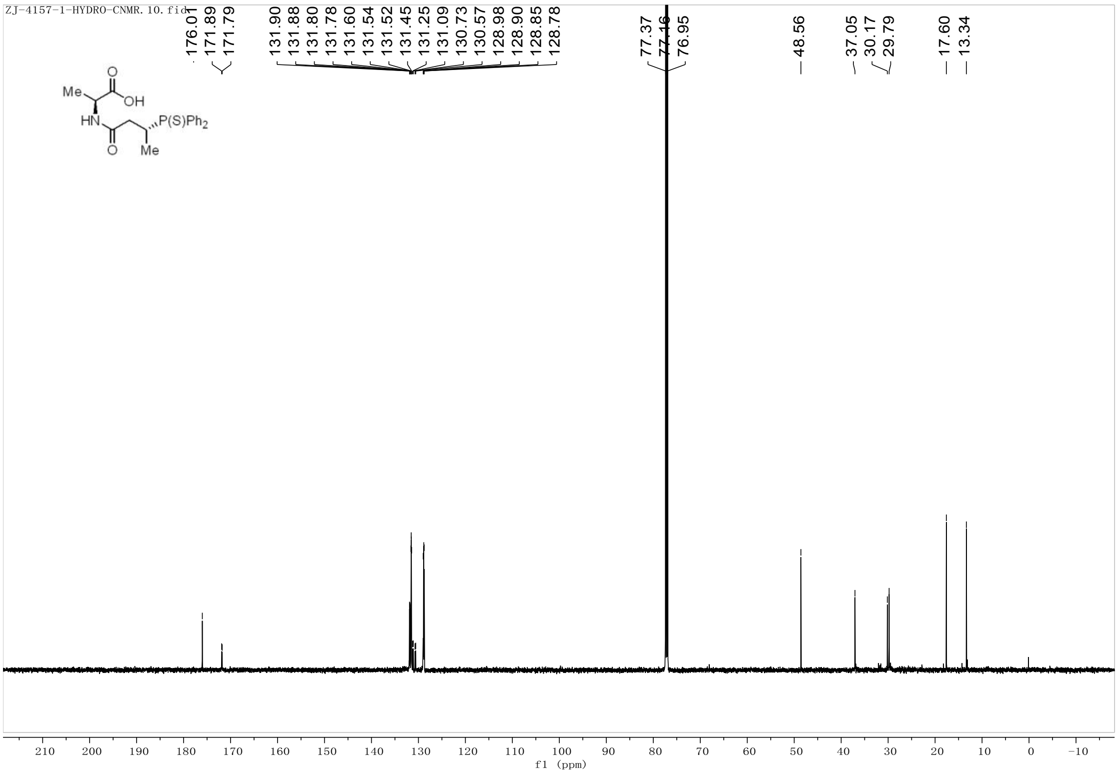

((*R*)-3-(diphenylphosphorothioyl)butanoyl)-D-tryptophan (12)

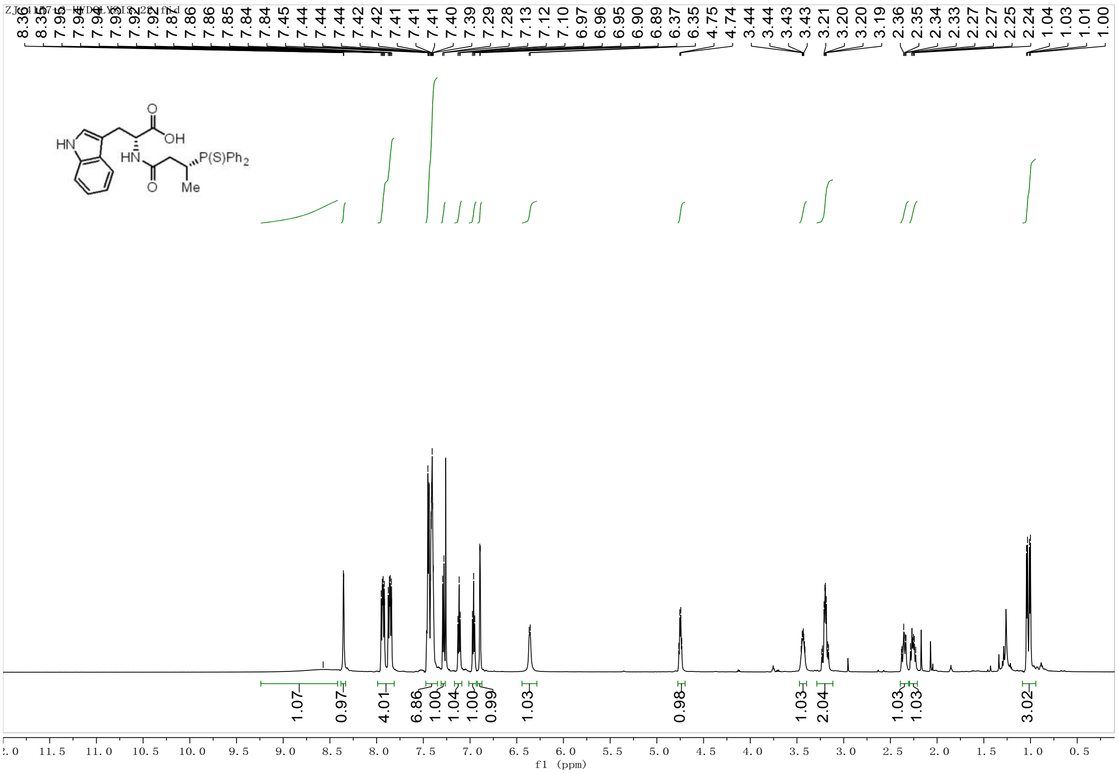

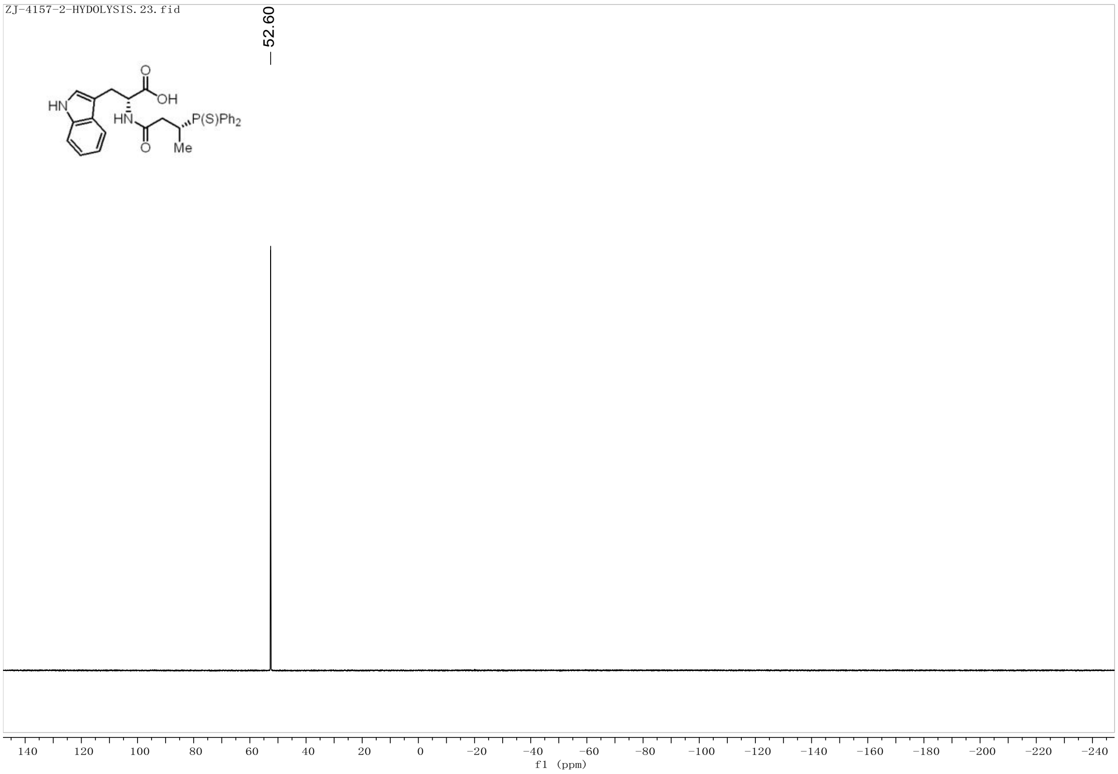
ZJ-4157-2-HYDOLYSIS.23.fid
52.60
HN
O
OH
HN
P(S)Ph2
O
Me
f1 (ppm)

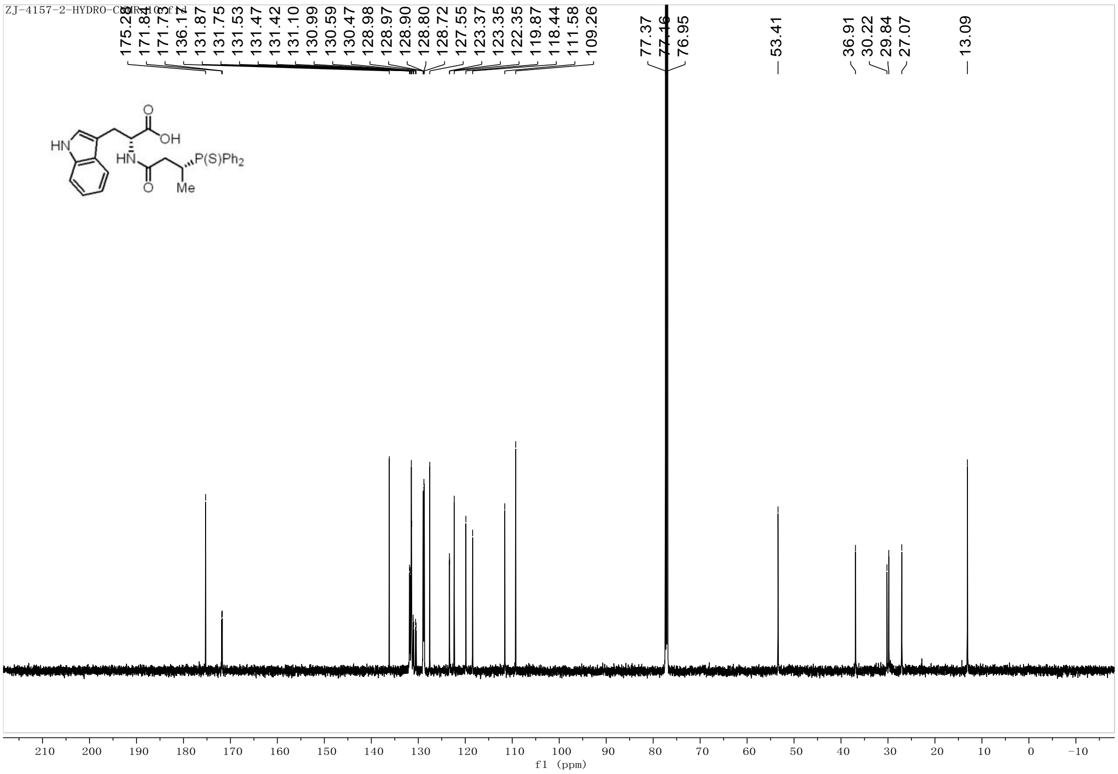
175.28
171.84
171.73
136.17
131.87
131.75
131.53
131.47
131.42
131.10
130.99
130.59
130.47
128.98
128.97
128.90
128.80
128.72
127.55
123.37
123.35
122.35
119.87
118.44
111.58
109.26
77.37
77.16
76.95
53.41
36.91
30.22
29.84
27.07
13.09
HN
O
OH
HN
P(S)Ph2
O
Me
f1 (ppm)

(4-(diphenylphosphorothioyl)butanoyl)-L-alanine (13)

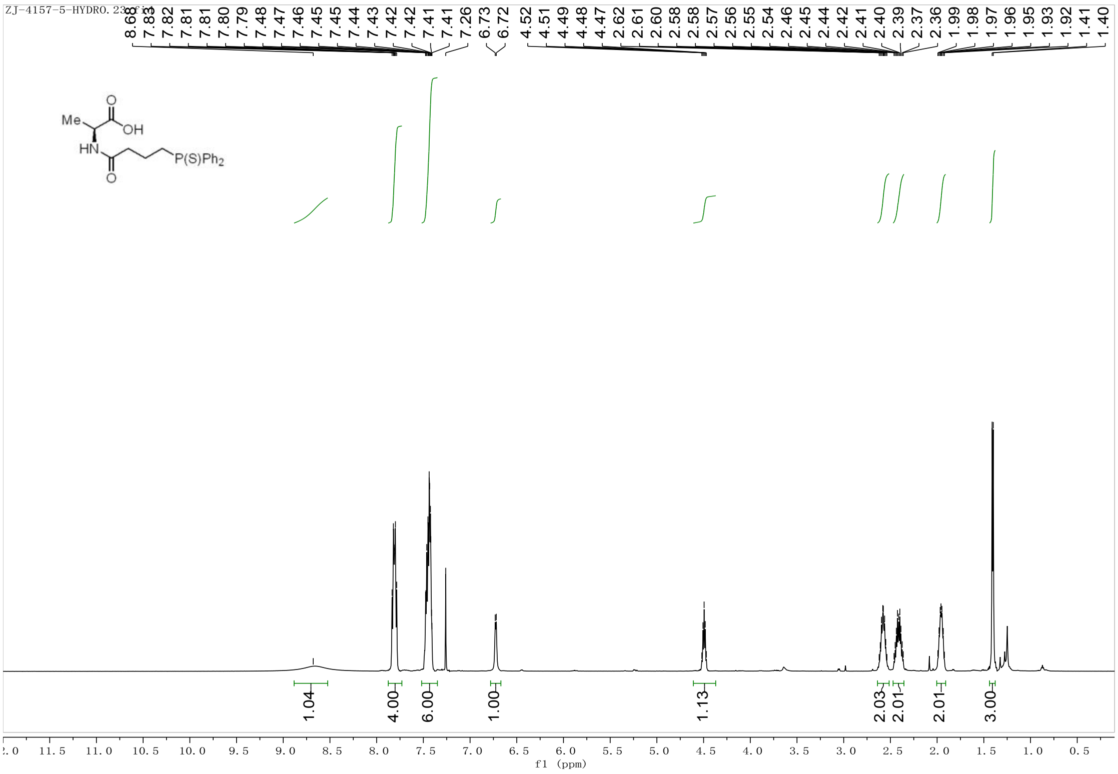

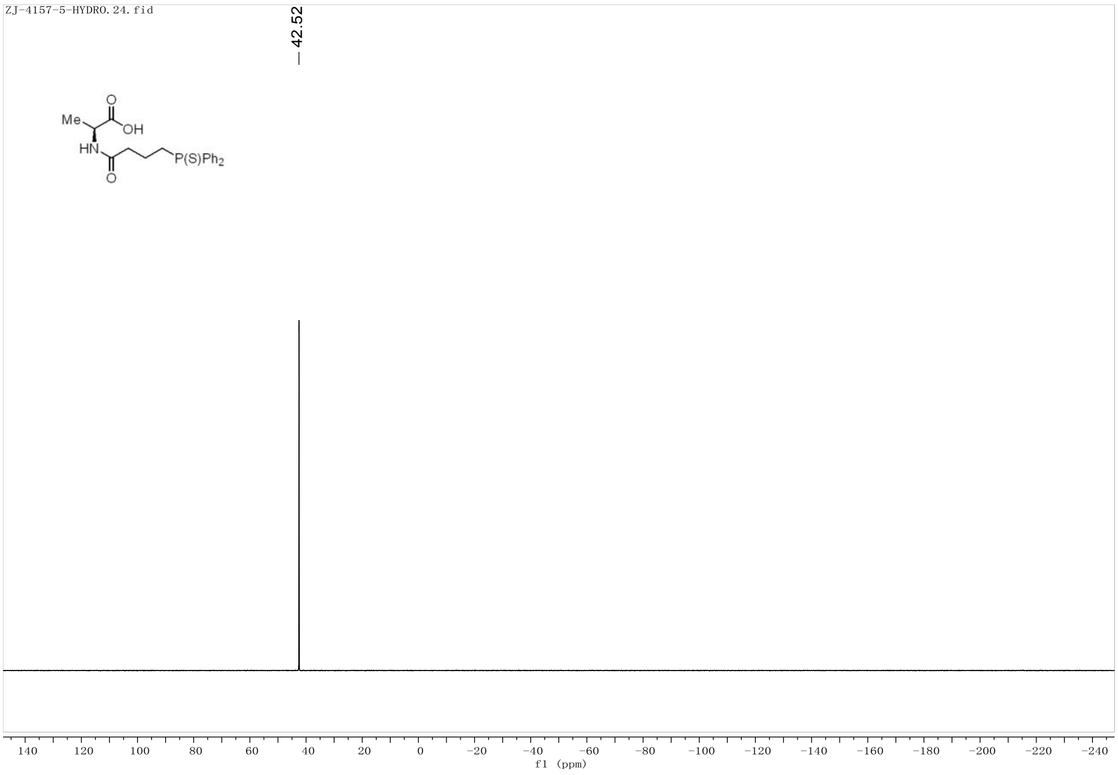

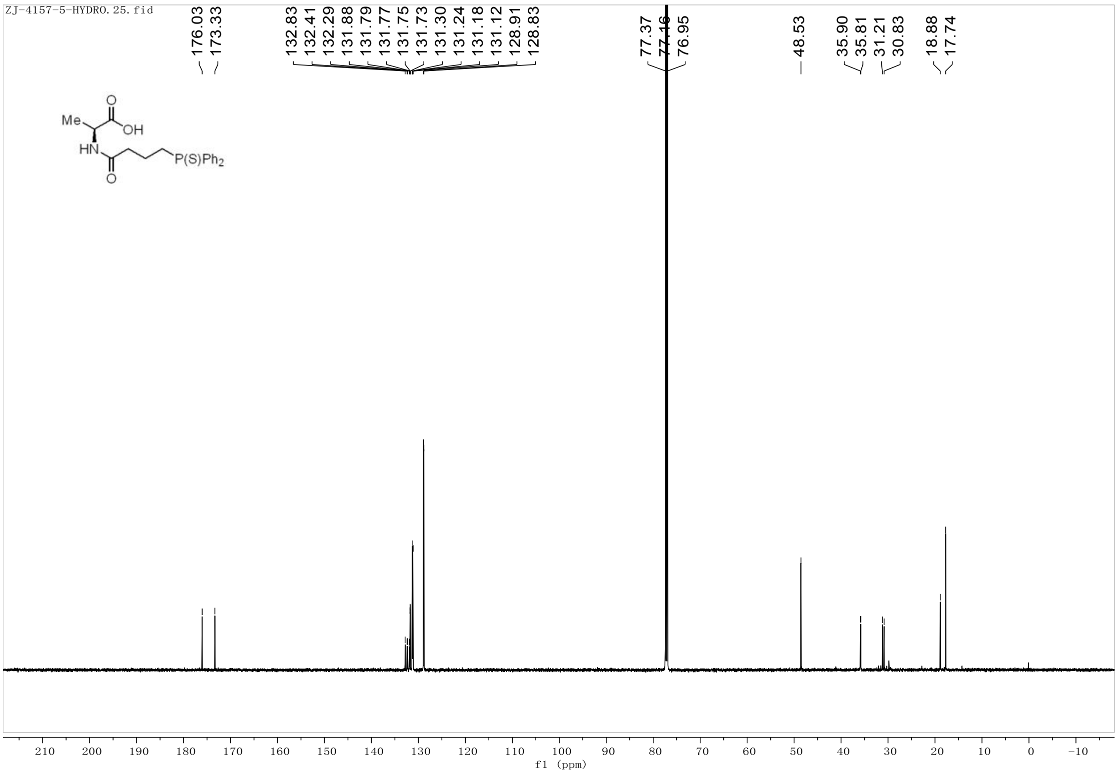

(*S*)-2-(4-(diphenylphosphorothioyl)butanamido)-2-phenylacetic acid (14)

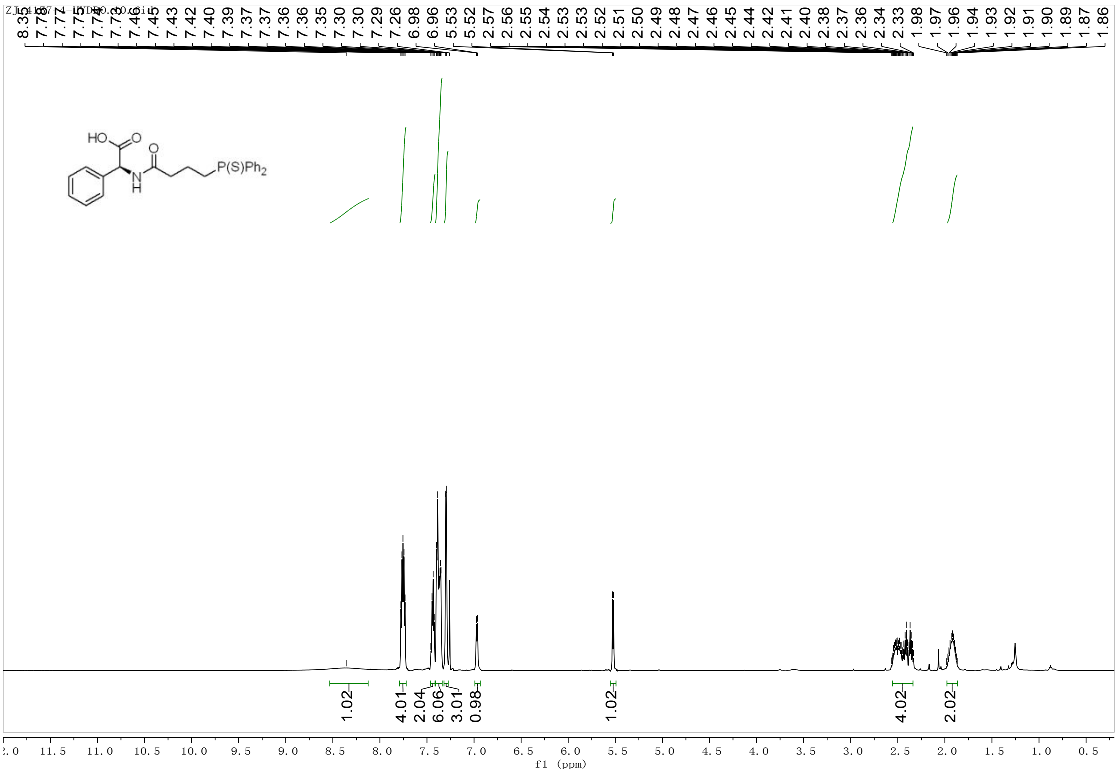

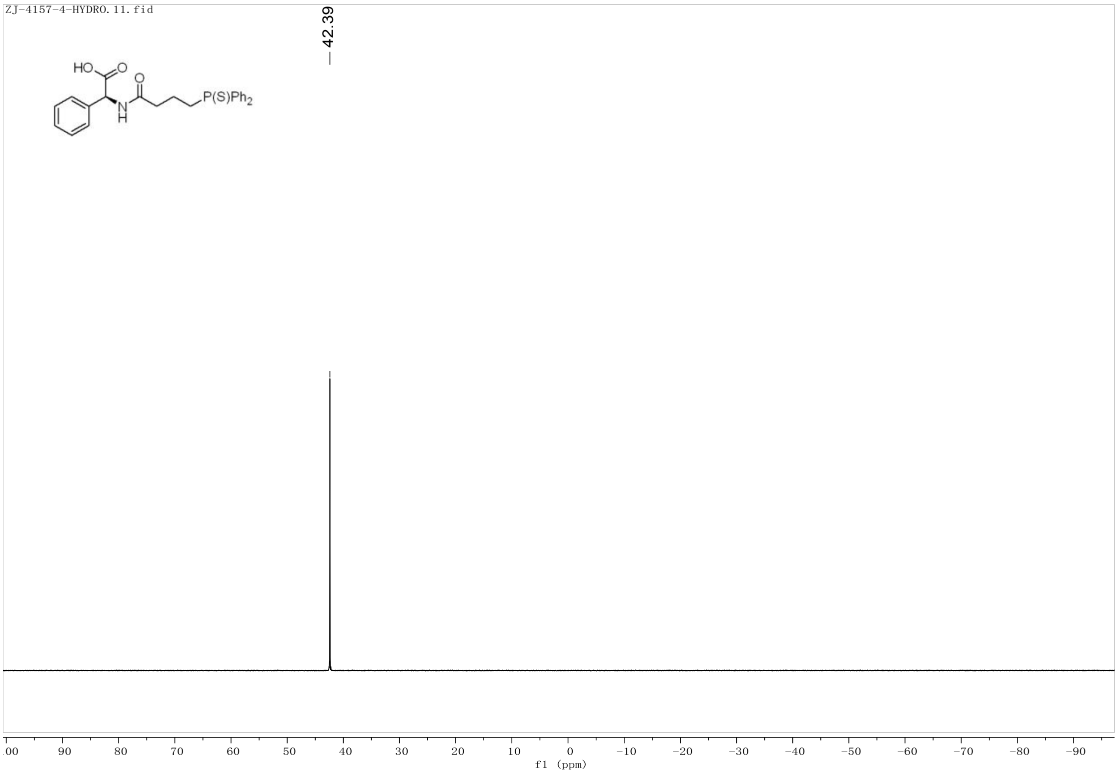
ZJ-4157-4-HYDRO.11.fid
42.39
HO
O
O
N
H
P(S)Ph2
f1 (ppm)

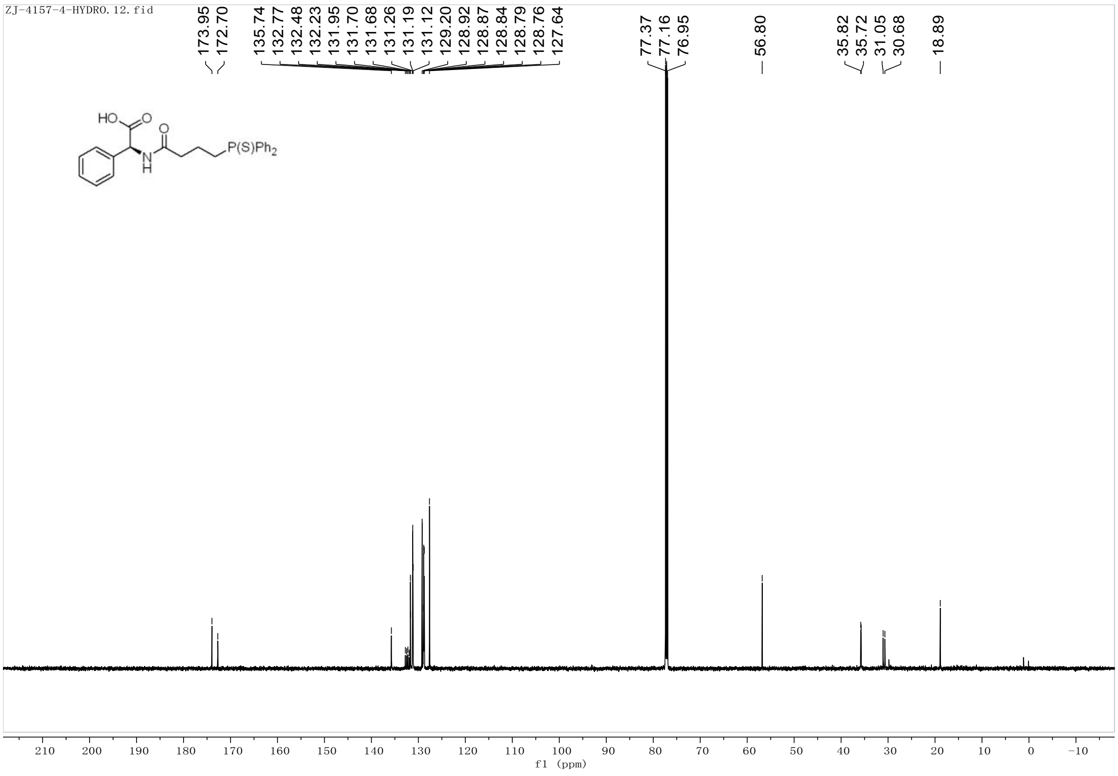
ZJ-4157-4-HYDRO.12.fid
173.95
172.70
135.74
132.77
132.48
132.23
131.95
131.70
131.68
131.26
131.19
131.12
129.20
128.92
128.87
128.84
128.79
128.76
127.64
77.37
77.16
76.95
56.80
35.82
35.72
31.05
30.68
18.89
HO
O
O
N
H
P(S)Ph2
f1 (ppm)

11. HPLC Spectrum of target compounds

(*R*)-3-(diphenylphosphorothioyl)-N-phenylbutanamide (3a)

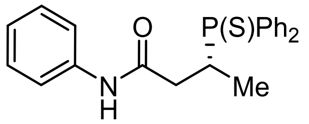

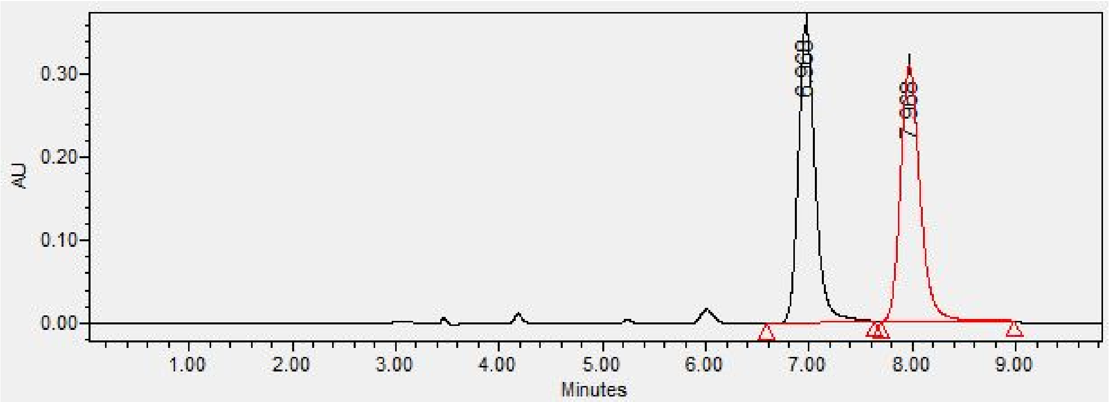

	Name	Retention Time (min)	Area (µV*sec)	% Area	Height (µV)	Int Type	Amount	Units	Peak Type	Peak Codes
1		6.968	4116204	50.07	360625	bb			Unknown	
2		7.968	4104349	49.93	309691	bb			Unknown	

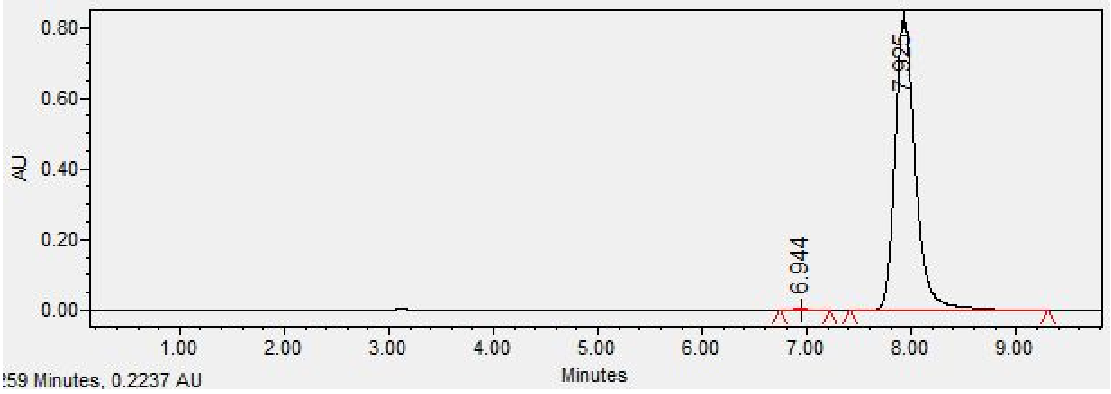

	Name	Retention Time (min)	Area (µV*sec)	% Area	Height (µV)	Int Type	Amount	Units	Peak Type	Peak Codes
1		6.944	48229	0.42	4370	bb			Unknown	
2		7.925	11304435	99.58	824954	bb			Unknown	

(*R*)-3-(diphenylphosphorothioyl)-N-(o-tolyl)butanamide (3b)

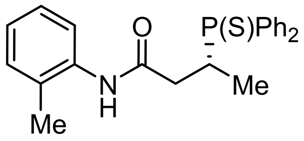

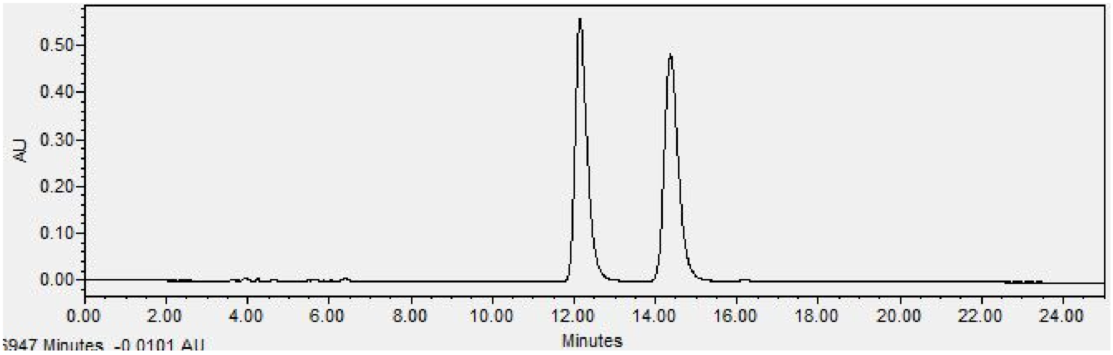

	Name	Retention Time (min)	Area (µV*sec)	% Area	Height (µV)	Int Type	Amount	Units	Peak Type	Peak Codes
1		12.141	11496475	49.82	559709	bb			Unknown	
2		14.367	11581555	50.18	483662	bb			Unknown	

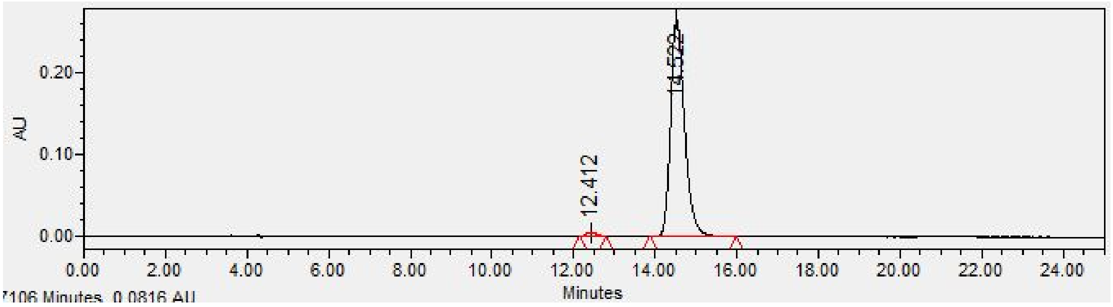

	Name	Retention Time (min)	Area (µV*sec)	% Area	Height (µV)	Int Type	Amount	Units	Peak Type	Peak Codes
1		12.412	92656	1.45	5150	bb			Unknown	
2		14.522	6298279	98.55	264947	bb			Unknown	

(*R*)-3-(diphenylphosphorothioyl)-N-(m-tolyl)butanamide (3c)

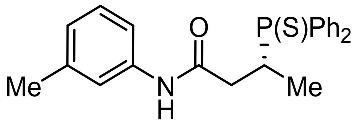

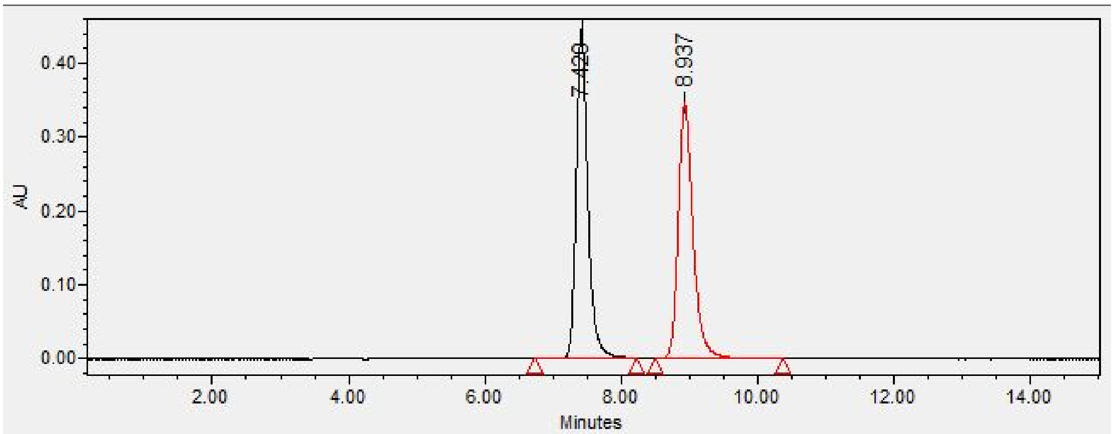

	Name	Retention Time (min)	Area (µV*sec)	% Area	Height (µV)	Int Type	Amount	Units	Peak Type	Peak Codes
1		7.420	5325135	50.65	445811	bb			Unknown	
2		8.937	5188321	49.35	347290	bb			Unknown	

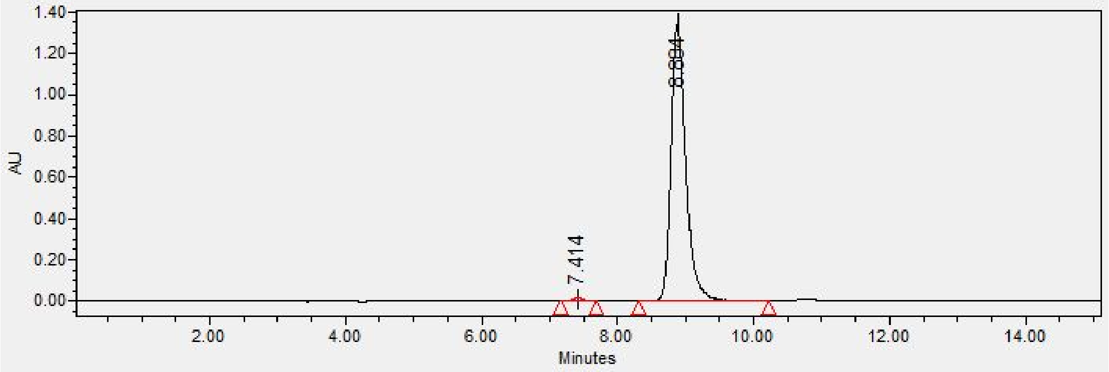

	Name	Retention Time (min)	Area (µV*sec)	% Area	Height (µV)	Int Type	Amount	Units	Peak Type	Peak Codes
1		7.414	201616	0.99	17658	bb			Unknown	
2		8.884	20136172	99.01	1339394	bb			Unknown	

(*R*)-3-(diphenylphosphorothioyl)-N-(p-tolyl)butanamide (3d)

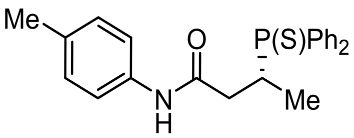

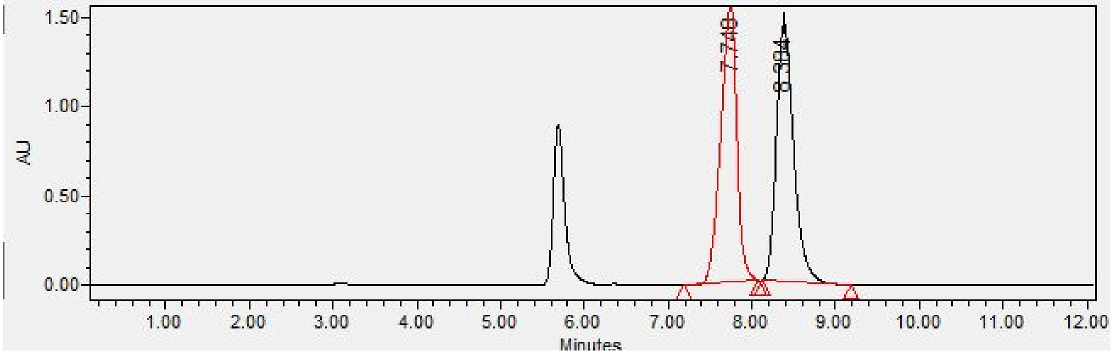

	Name	Retention Time (min)	Area (μV*sec)	% Area	Height (μV)	Int Type	Amount	Units	Peak Type	Peak Codes
1		7.748	20761956	49.98	1562493	bb			Unknown	
2		8.384	20779402	50.02	1444774	bb			Unknown	

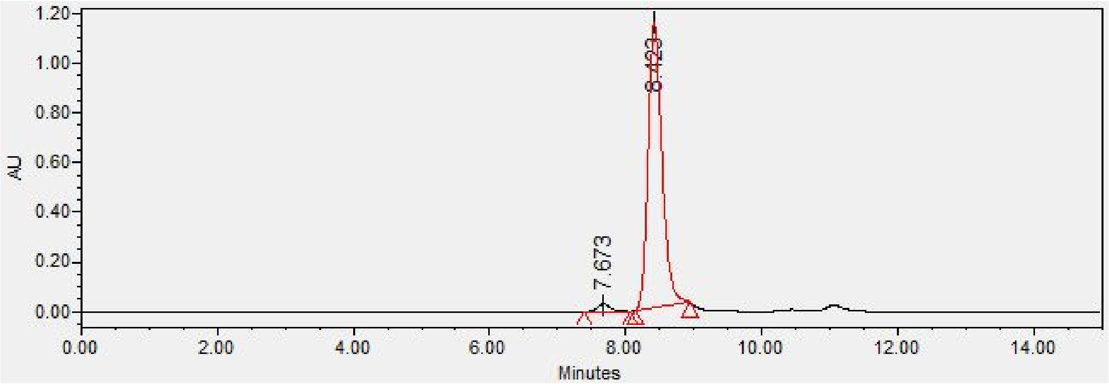

	Name	Retention Time (min)	Area (μV*sec)	% Area	Height (μV)	Int Type	Amount	Units	Peak Type	Peak Codes
1		7.673	380039	2.27	29661	bb			Unknown	
2		8.423	16382579	97.73	1145425	bb			Unknown	

(*R*)-N-(4-(tert-butyl)phenyl)-3-(diphenylphosphorothioyl)butanamide (3e)

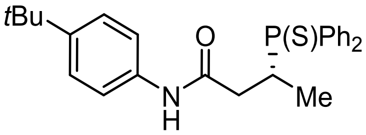

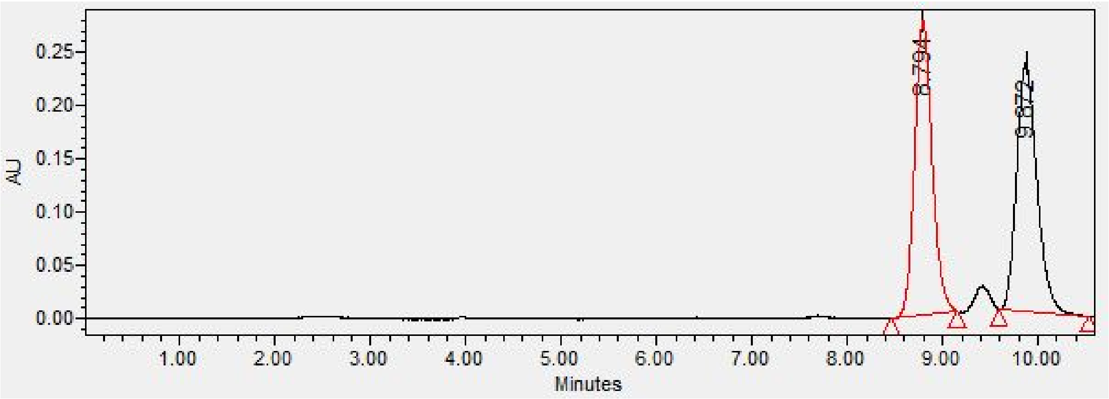

	Name	Retention Time (min)	Area (µV*sec)	% Area	Height (µV)	Int Type	Amount	Units	Peak Type	Peak Codes
1		8.794	3509865	50.39	275396	bb			Unknown	
2		9.872	3455252	49.61	232690	bb			Unknown	

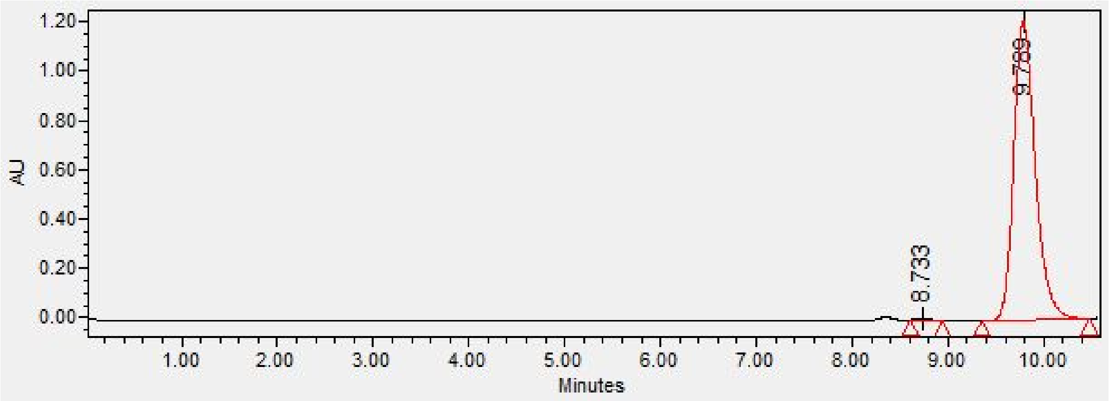

	Name	Retention Time (min)	Area (µV*sec)	% Area	Height (µV)	Int Type	Amount	Units	Peak Type	Peak Codes
1		8.733	80344	0.42	8211	bb			Unknown	
2		9.789	18826887	99.58	1208142	bb			Unknown	

(*R*)-N-(4-benzylphenyl)-3-(diphenylphosphorothioyl)butanamide (3f)

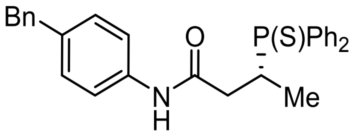

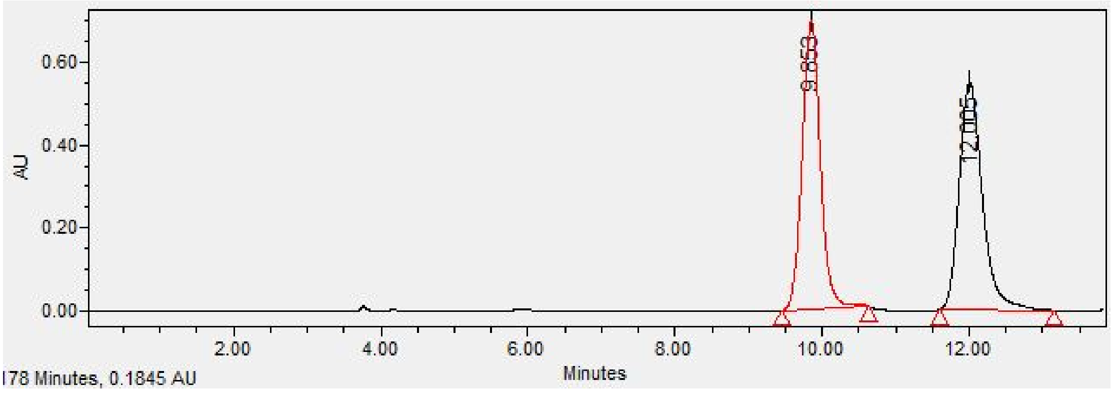

	Name	Retention Time (min)	Area (µV*sec)	% Area	Height (µV)	Int Type	Amount	Units	Peak Type	Peak Codes
1		9.853	12044168	49.83	695527	bb			Unknown	
2		12.005	12126124	50.17	550069	bb			Unknown	

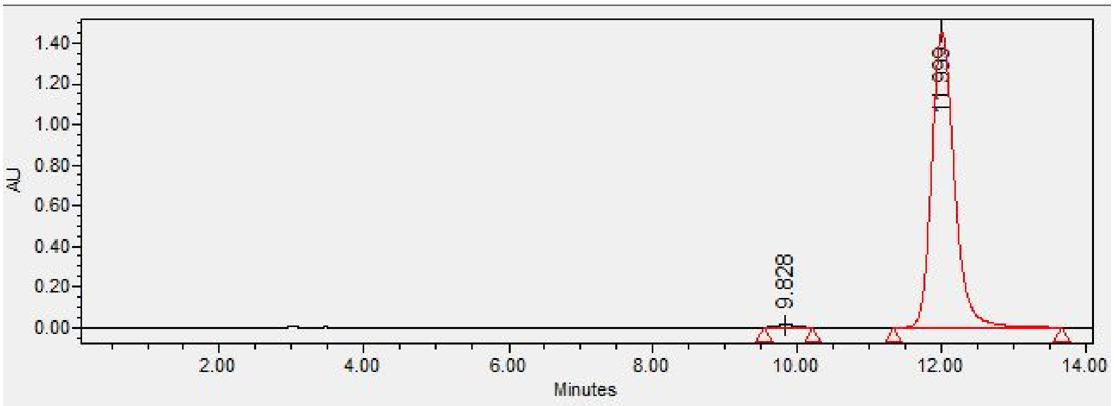

	Name	Retention Time (min)	Area (µV*sec)	% Area	Height (µV)	Int Type	Amount	Units	Peak Type	Peak Codes
1		9.828	217864	0.68	13250	bb			Unknown	
2		11.999	31667460	99.32	1454853	bb			Unknown	

(*R*)-3-(diphenylphosphorothioyl)-N-(4-(trifluoromethyl)phenyl)butanamide (3g)

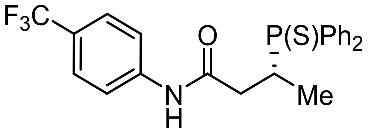

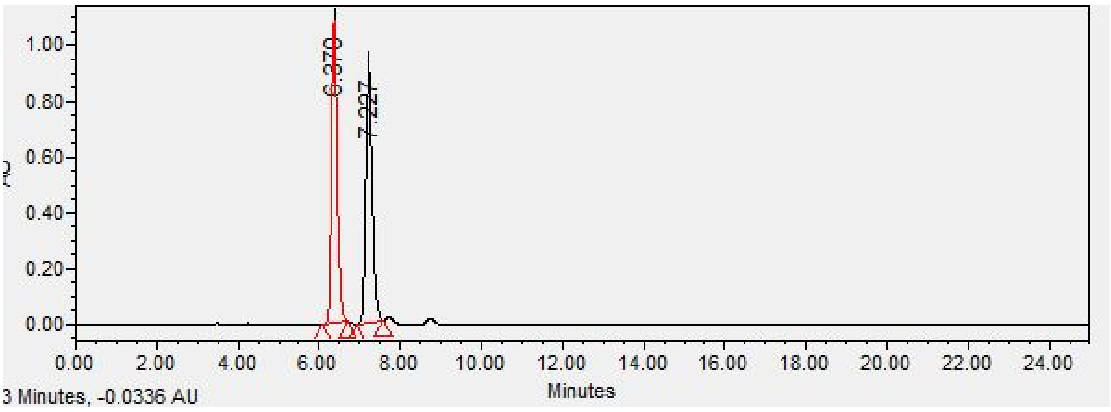

	Name	Retention Time (min)	Area (µV*sec)	% Area	Height (µV)	Int Type	Amount	Units	Peak Type	Peak Codes
1		6.370	10151337	50.37	1070014	bb			Unknown	
2		7.227	10003112	49.63	910352	bb			Unknown	

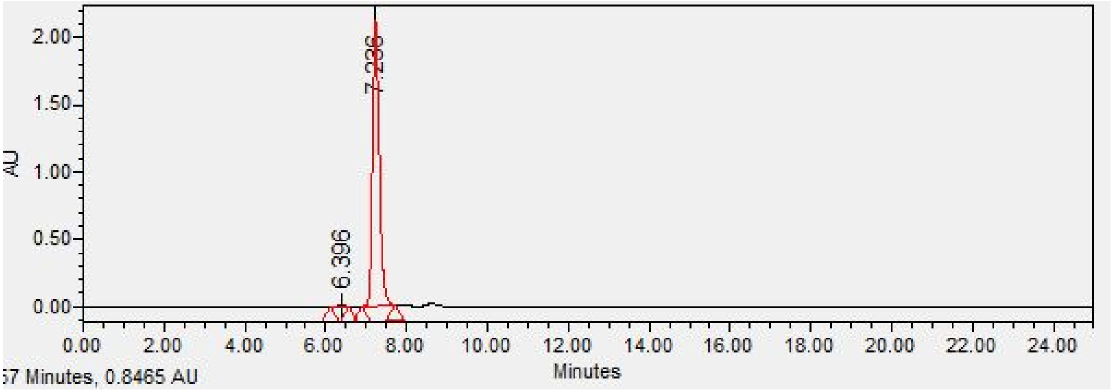

	Name	Retention Time (min)	Area (µV*sec)	% Area	Height (µV)	Int Type	Amount	Units	Peak Type	Peak Codes
1		6.396	110920	0.45	12407	bb			Unknown	
2		7.236	24510193	99.55	2122872	bb			Unknown	

(*R*)-3-(diphenylphosphorothioyl)-N-(4-fluorophenyl)butanamide (3h)

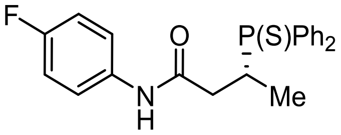

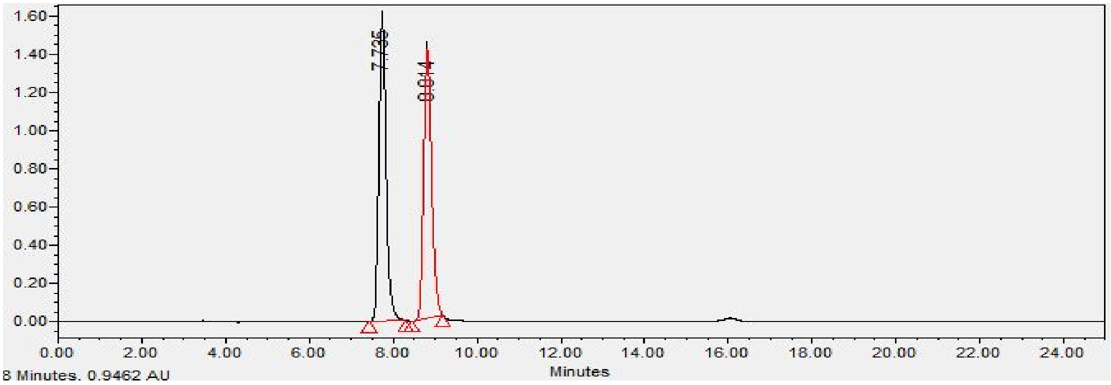

	Name	Retention Time (min)	Area (µV*sec)	% Area	Height (µV)	Int Type	Amount	Units	Peak Type	Peak Codes
1		7.735	19049305	50.31	1577913	bb			Unknown	
2		8.814	18818304	49.69	1407384	bb			Unknown	

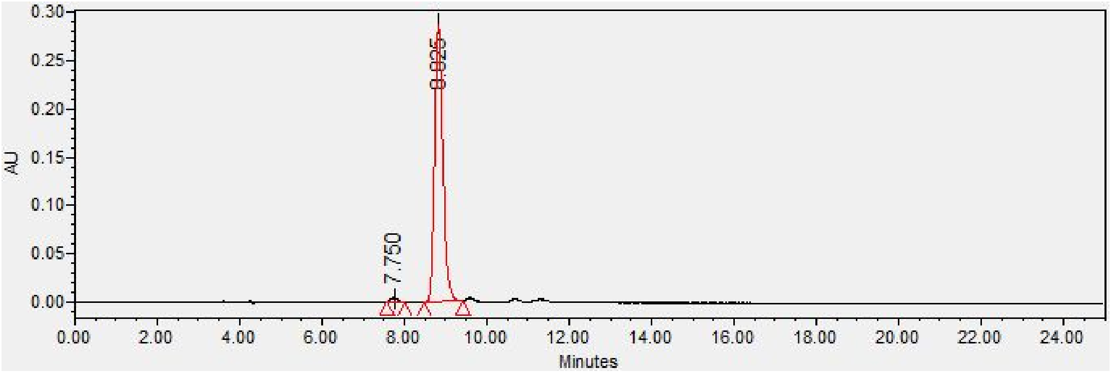

	Name	Retention Time (min)	Area (µV*sec)	% Area	Height (µV)	Int Type	Amount	Units	Peak Type	Peak Codes
1		7.750	48890	1.22	4592	bb			Unknown	
2		8.825	3964051	98.78	287569	bb			Unknown	

(*R*)-N-(4-chlorophenyl)-3-(diphenylphosphorothioyl)butanamide (3i)

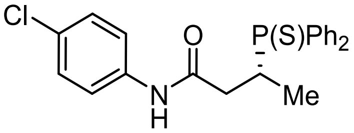

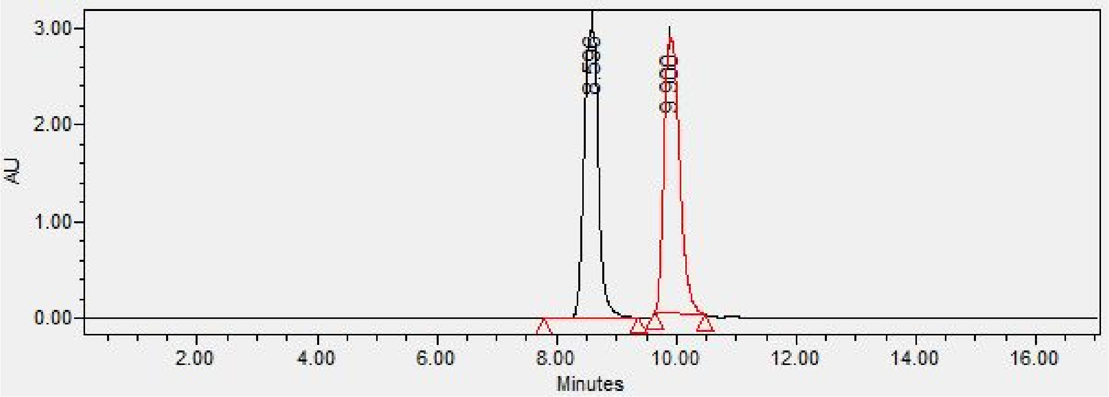

	Name	Retention Time (min)	Area (µV*sec)	% Area	Height (µV)	Int Type	Amount	Units	Peak Type	Peak Codes
1		8.596	50333503	49.25	3075665	bb			Unknown	
2		9.900	51863725	50.75	2847091	bb			Unknown	

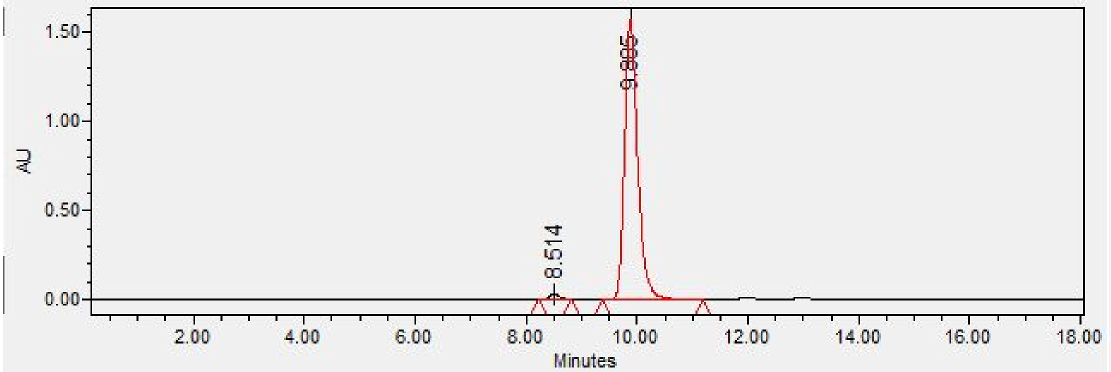

	Name	Retention Time (min)	Area (µV*sec)	% Area	Height (µV)	Int Type	Amount	Units	Peak Type	Peak Codes
1		8.514	452362	1.72	35054	bb			Unknown	
2		9.885	25832921	98.28	1565794	bb			Unknown	

(*R*)-N-(4-bromophenyl)-3-(diphenylphosphorothioyl)butanamide (3j)

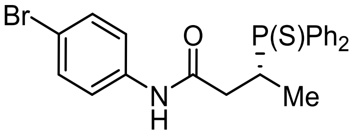

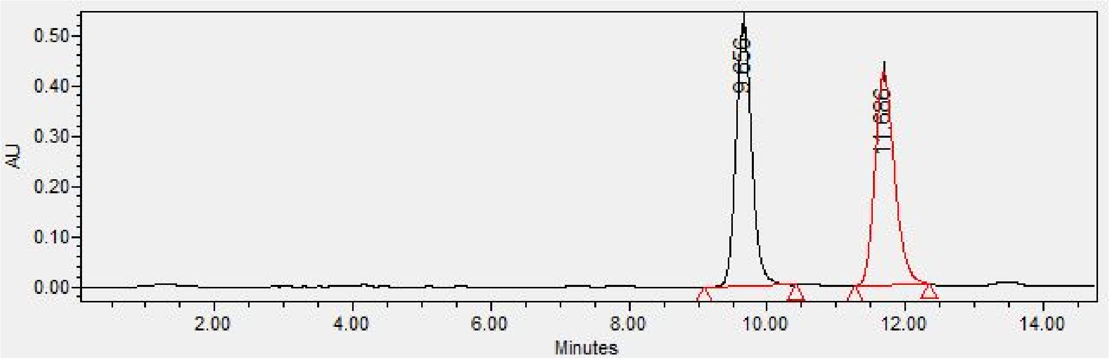

	Name	Retention Time (min)	Area (µV*sec)	% Area	Height (µV)	Int Type	Amount	Units	Peak Type	Peak Codes
1		9.656	8349975	49.77	522552	bb			Unknown	
2		11.686	8426546	50.23	423218	bb			Unknown	

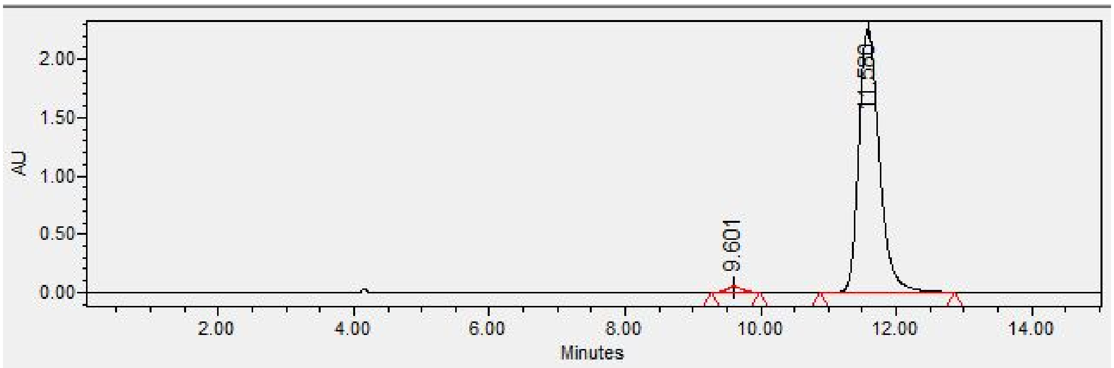

	Name	Retention Time (min)	Area (µV*sec)	% Area	Height (µV)	Int Type	Amount	Units	Peak Type	Peak Codes
1		9.601	784600	1.62	50996	bb			Unknown	
2		11.580	47529591	98.38	2259935	bb			Unknown	

(*R*)-3-(diphenylphosphorothioyl)-N-(4-methoxyphenyl)butanamide (3k)

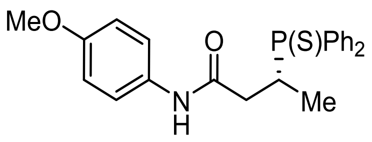

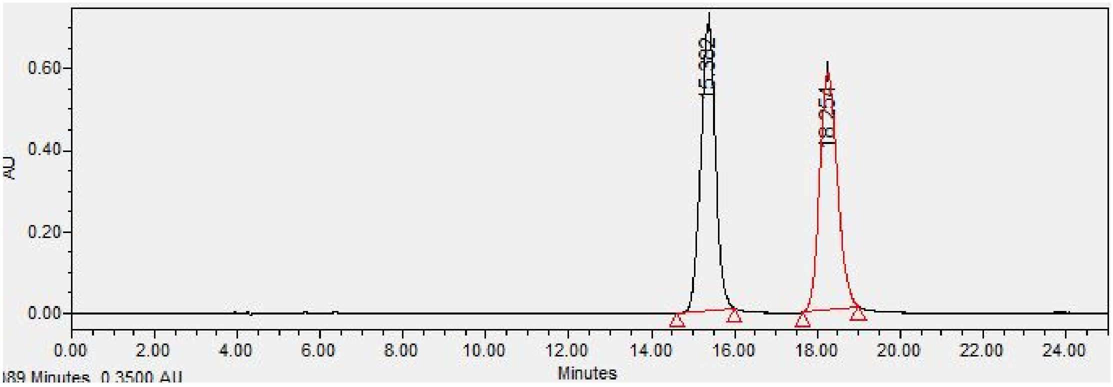

	Name	Retention Time (min)	Area (µV*sec)	% Area	Height (µV)	Int Type	Amount	Units	Peak Type	Peak Codes
1		15.382	17488088	50.31	705618	bb			Unknown	
2		18.254	17273362	49.69	581721	bb			Unknown	

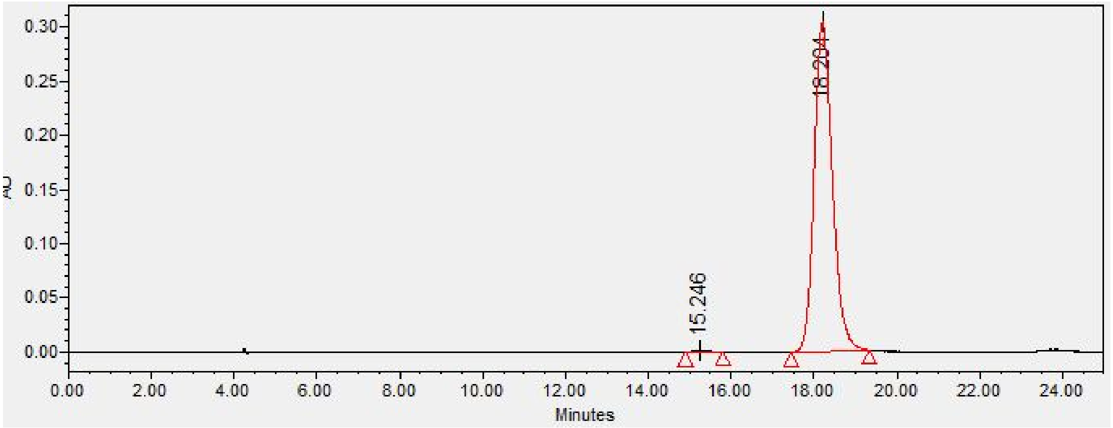

	Name	Retention Time (min)	Area (µV*sec)	% Area	Height (µV)	Int Type	Amount	Units	Peak Type	Peak Codes
1		15.246	43858	0.47	2049	bb			Unknown	
2		18.204	9228232	99.53	303666	bb			Unknown	

(*R*)-3-(diphenylphosphorothioyl)-N-(4-phenoxyphenyl)butanamide (3l)

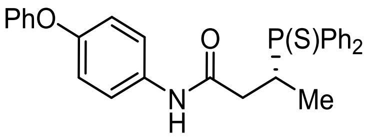

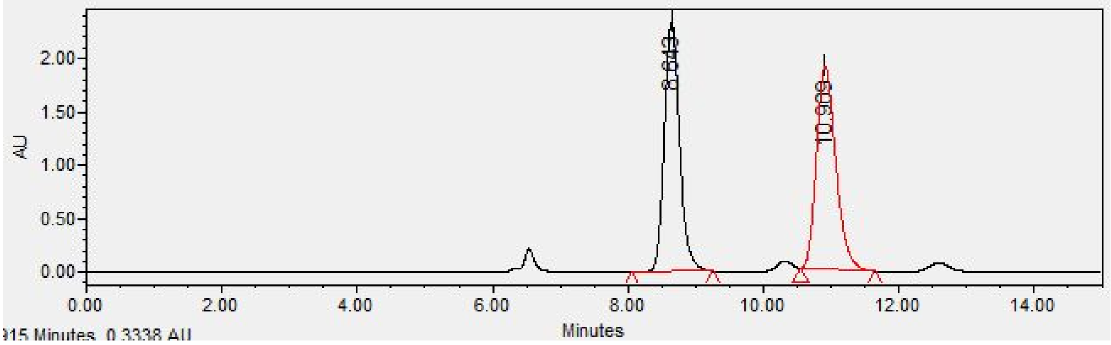

	Name	Retention Time (min)	Area (μV*sec)	% Area	Height (μV)	Int Type	Amount	Units	Peak Type	Peak Codes
1		8.643	36692488	49.30	2337699	bb			Unknown	
2		10.909	37739379	50.70	1896958	bb			Unknown	

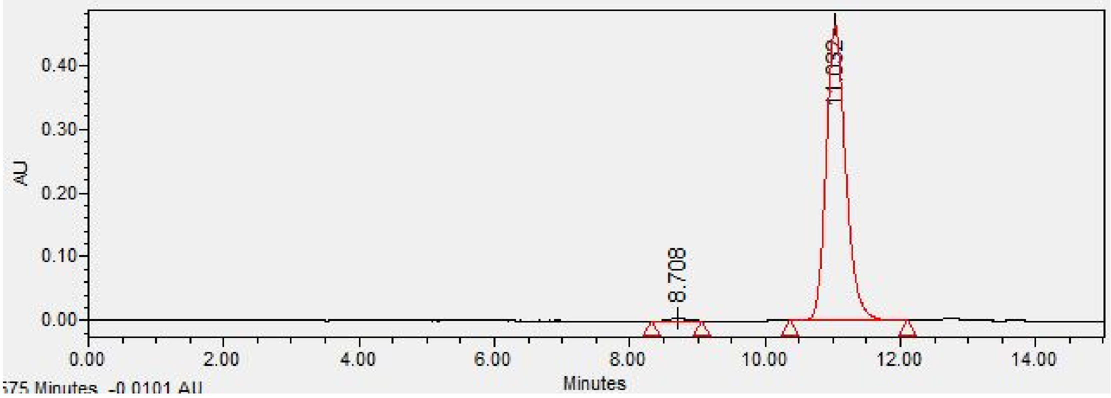

	Name	Retention Time (min)	Area (μV*sec)	% Area	Height (μV)	Int Type	Amount	Units	Peak Type	Peak Codes
1		8.708	73703	0.79	4919	bb			Unknown	
2		11.032	9297711	99.21	463549	bb			Unknown	

(*R*)-3-(diphenylphosphorothioyl)-N-(4-(trifluoromethoxy)phenyl)butanamide (3m)

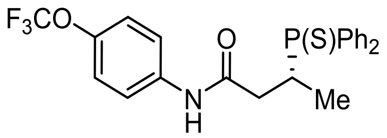

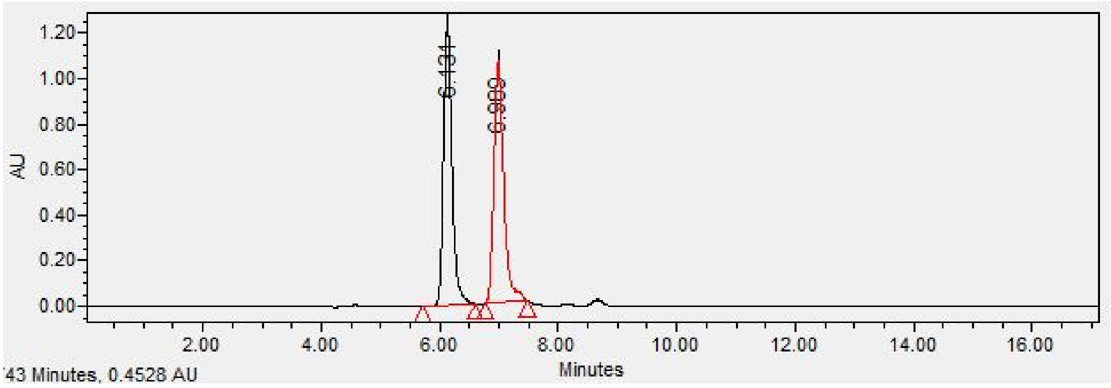

	Name	Retention Time (min)	Area (µV*sec)	% Area	Height (µV)	Int Type	Amount	Units	Peak Type	Peak Codes
1		6.131	12181596	49.55	1221329	bb			Unknown	
2		6.989	12403779	50.45	1062026	bb			Unknown	

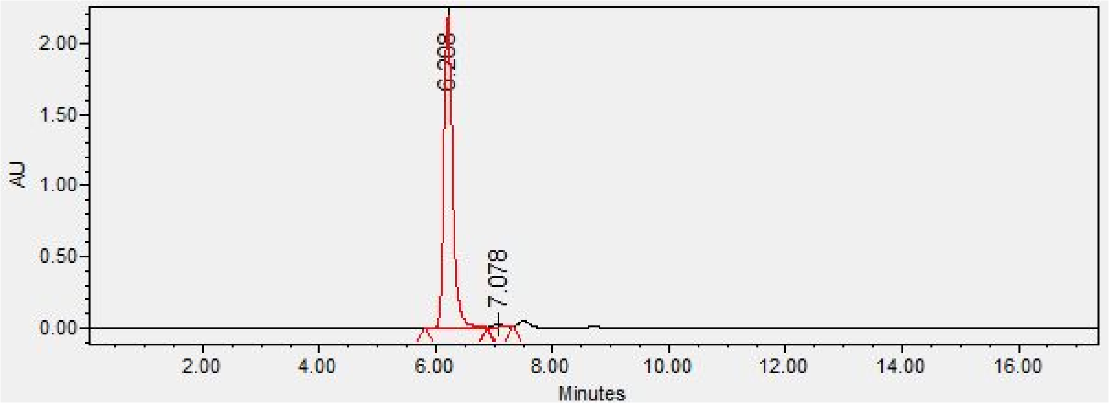

	Name	Retention Time (min)	Area (µV*sec)	% Area	Height (µV)	Int Type	Amount	Units	Peak Type	Peak Codes
1		6.208	22428717	99.03	2184414	bb			Unknown	
2		7.078	218703	0.97	21547	bb			Unknown	

(*R*)-3-(diphenylphosphorothioyl)-N-(4-vinylphenyl)butanamide (3n)

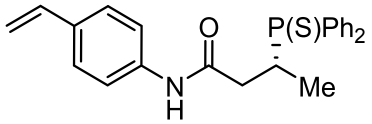

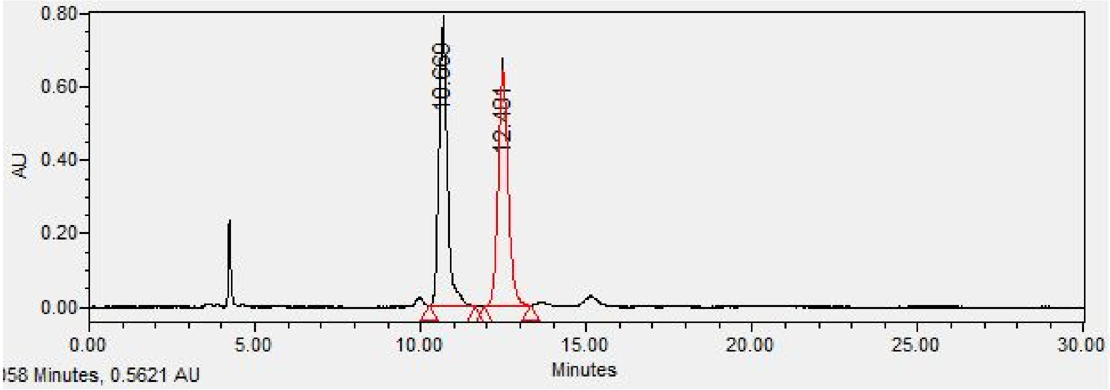

	Name	Retention Time (min)	Area (µV*sec)	% Area	Height (µV)	Int Type	Amount	Units	Peak Type	Peak Codes
1		10.669	13698242	49.86	764258	bb			Unknown	
2		12.481	13772652	50.14	644822	bb			Unknown	

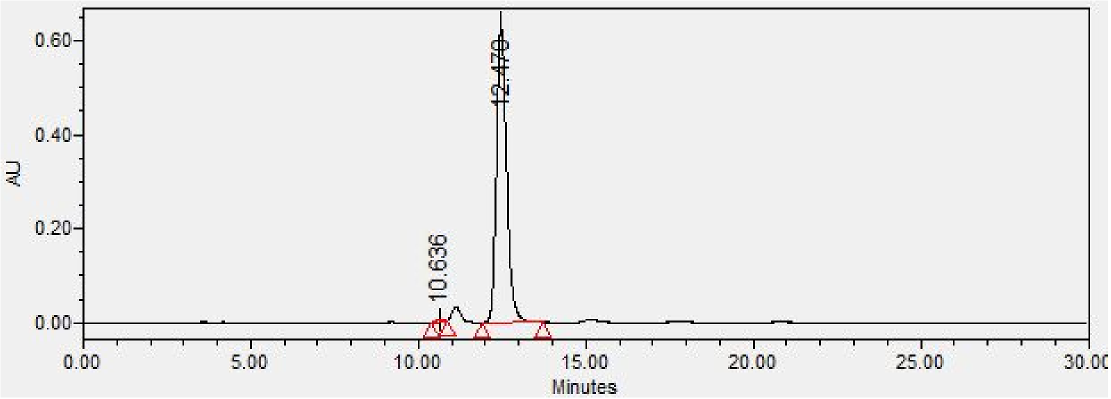

	Name	Retention Time (min)	Area (µV*sec)	% Area	Height (µV)	Int Type	Amount	Units	Peak Type	Peak Codes
1		10.636	66447	0.50	5500	bb			Unknown	
2		12.470	13276073	99.50	636820	bb			Unknown	

(*R*)-N-(3,5-difluorophenyl)-3-(diphenylphosphorothioyl)butanamide (3o)

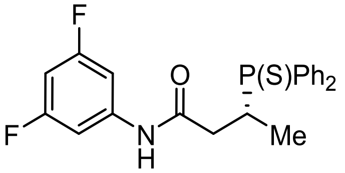

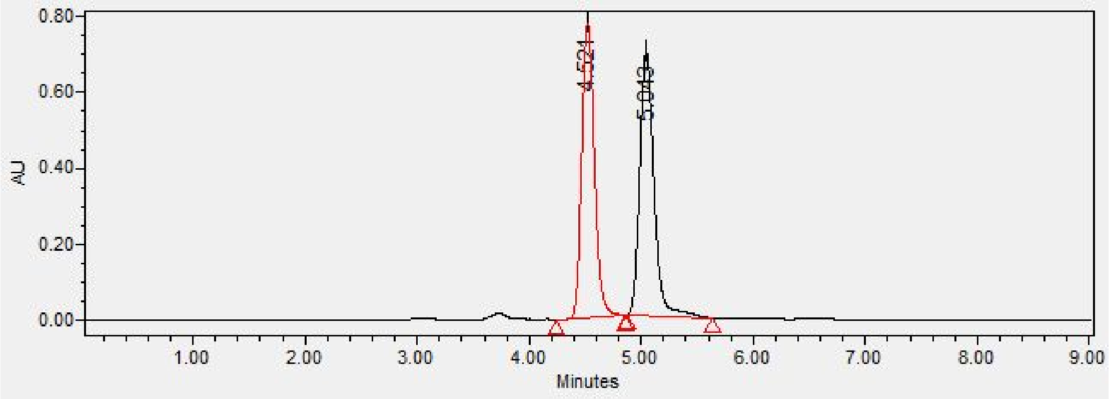

	Name	Retention Time (min)	Area (μV*sec)	% Area	Height (μV)	Int Type	Amount	Units	Peak Type	Peak Codes
1		4.521	6086774	49.80	779124	bb			Unknown	
2		5.043	6136143	50.20	696822	bb			Unknown	

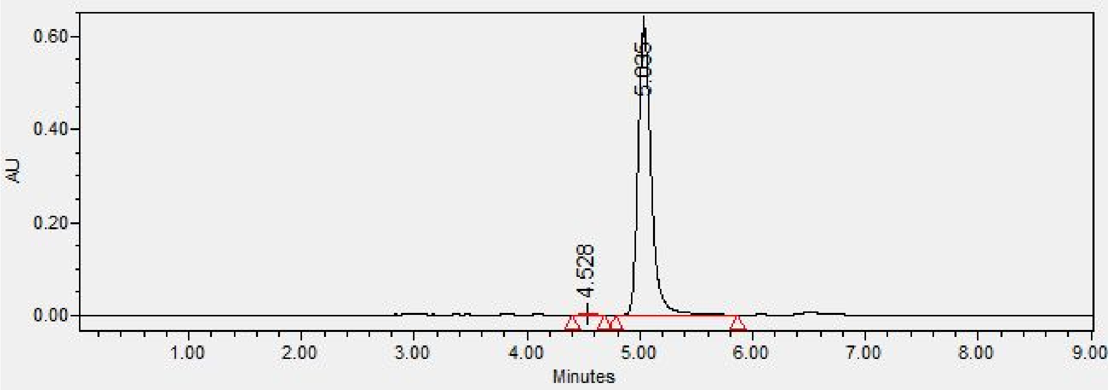

	Name	Retention Time (min)	Area (μV*sec)	% Area	Height (μV)	Int Type	Amount	Units	Peak Type	Peak Codes
1		4.528	28181	0.56	4166	bb			Unknown	
2		5.035	5001036	99.44	620248	bb			Unknown	

(*R*)-3-(diphenylphosphorothioyl)-N-(4-morpholinophenyl)butanamide (3p)

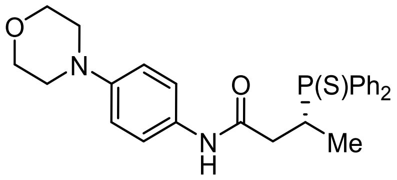

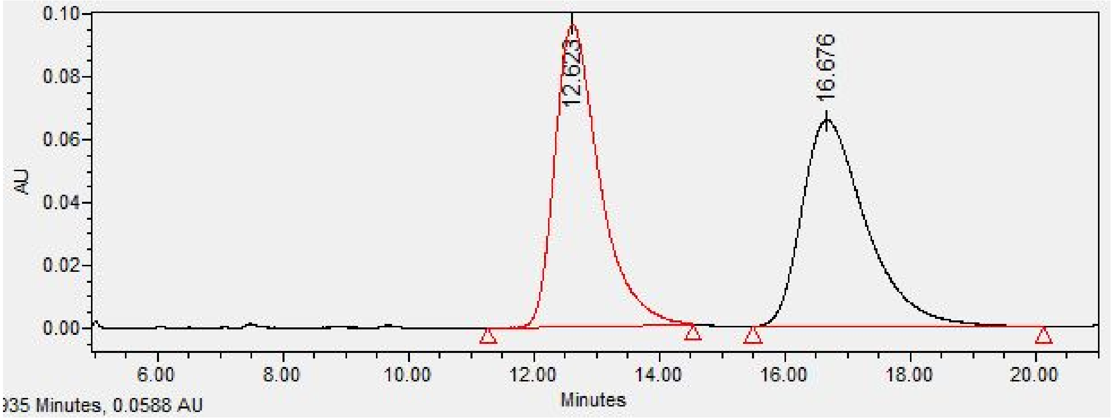

	Name	Retention Time (min)	Area (µV*sec)	% Area	Height (µV)	Int Type	Amount	Units	Peak Type	Peak Codes
1		12.623	4755032	50.27	96322	bb			Unknown	
2		16.676	4704139	49.73	65866	bb			Unknown	

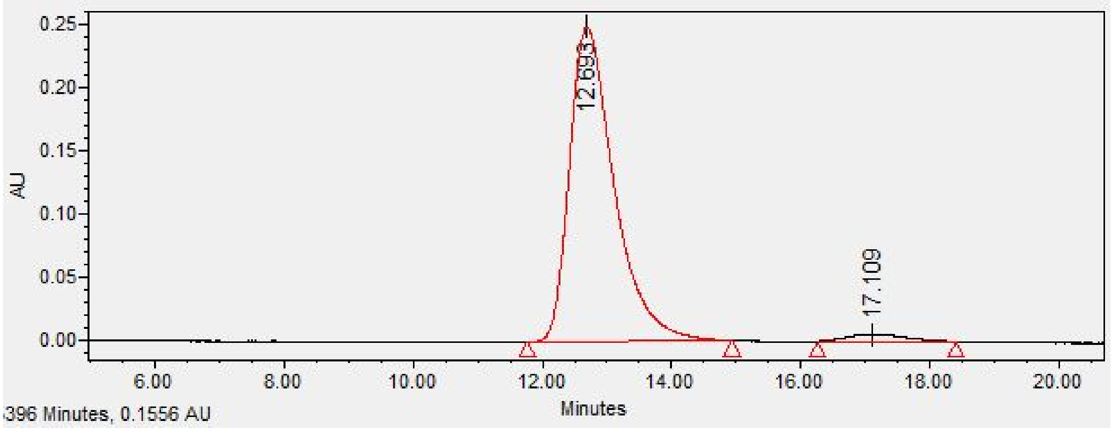

	Name	Retention Time (min)	Area (µV*sec)	% Area	Height (µV)	Int Type	Amount	Units	Peak Type	Peak Codes
1		12.693	11960553	97.01	249138	BB			Unknown	
2		17.109	368806	2.99	5903	BB			Unknown	

(*R*)-N-(4-acetylphenyl)-3-(diphenylphosphorothioyl)butanamide (3q)

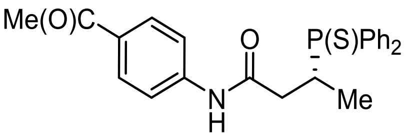

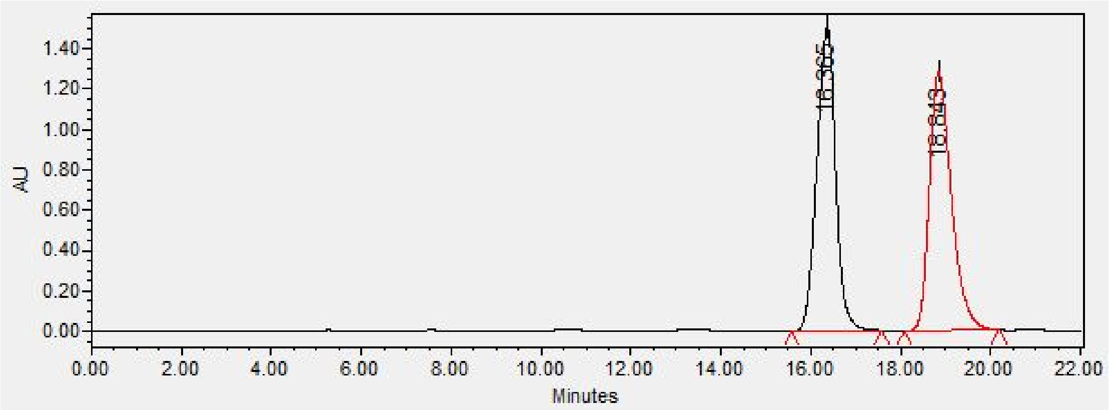

	Name	Retention Time (min)	Area (μV*sec)	% Area	Height (μV)	Int Type	Amount	Units	Peak Type	Peak Codes
1		16.365	43612434	50.00	1503228	bb			Unknown	
2		18.843	43621043	50.00	1279954	bb			Unknown	

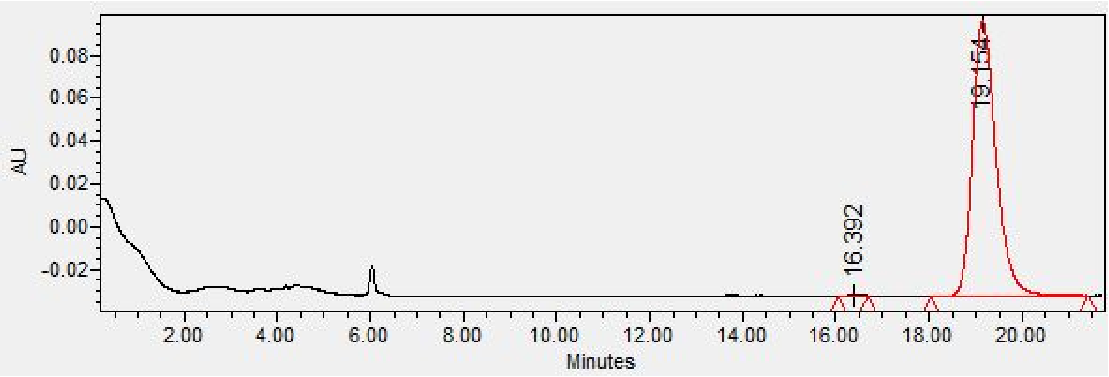

	Name	Retention Time (min)	Area (μV*sec)	% Area	Height (μV)	Int Type	Amount	Units	Peak Type	Peak Codes
1		16.392	13343	0.29	701	bb			Unknown	
2		19.154	4513179	99.71	128756	bb			Unknown	

methyl (*R*)-4-(3-(diphenylphosphorothioyl)butanamido)benzoate (3r)

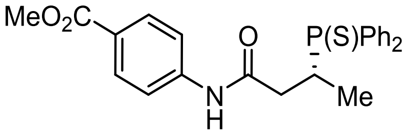

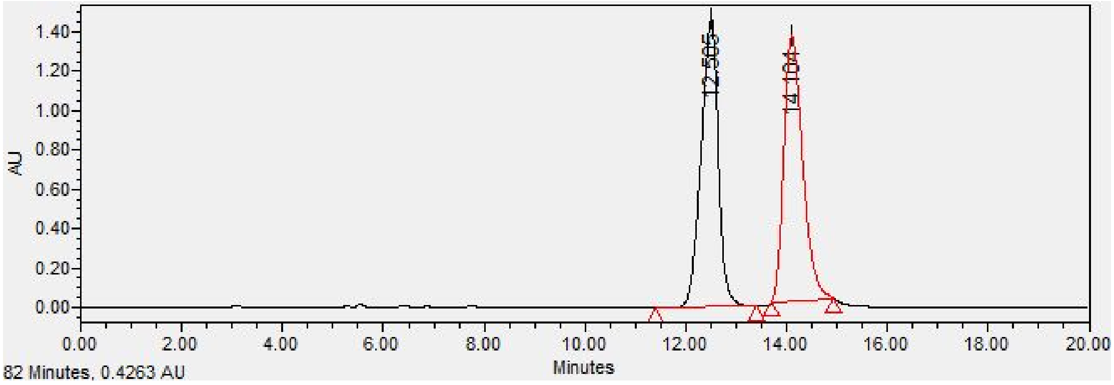

	Name	Retention Time (min)	Area (µV*sec)	% Area	Height (µV)	Int Type	Amount	Units	Peak Type	Peak Codes
1		12.505	34600357	49.70	1459901	bb			Unknown	
2		14.104	35020498	50.30	1346100	bb			Unknown	

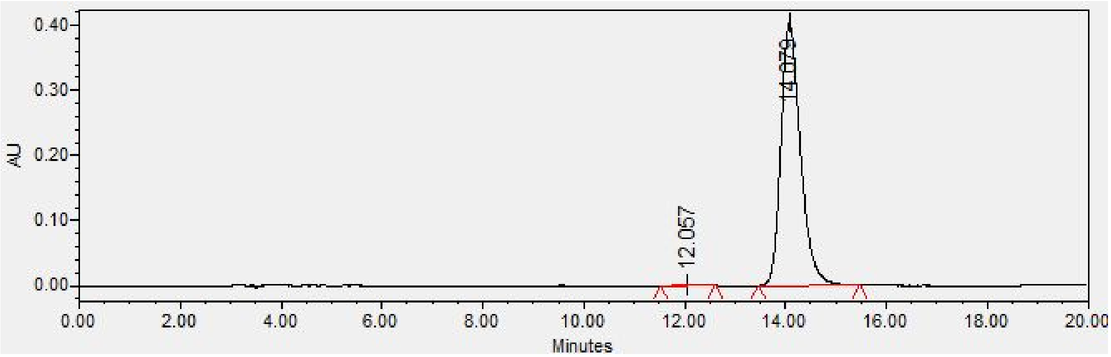

	Name	Retention Time (min)	Area (µV*sec)	% Area	Height (µV)	Int Type	Amount	Units	Peak Type	Peak Codes
1		12.057	68921	0.63	2110	bb			Unknown	
2		14.079	10880987	99.37	403037	BB			Unknown	

(*R*)-3-(diphenylphosphorothioyl)-N-(naphthalen-2-yl)butanamide (3s)

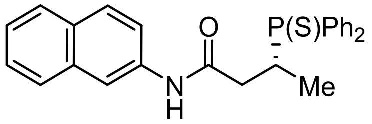

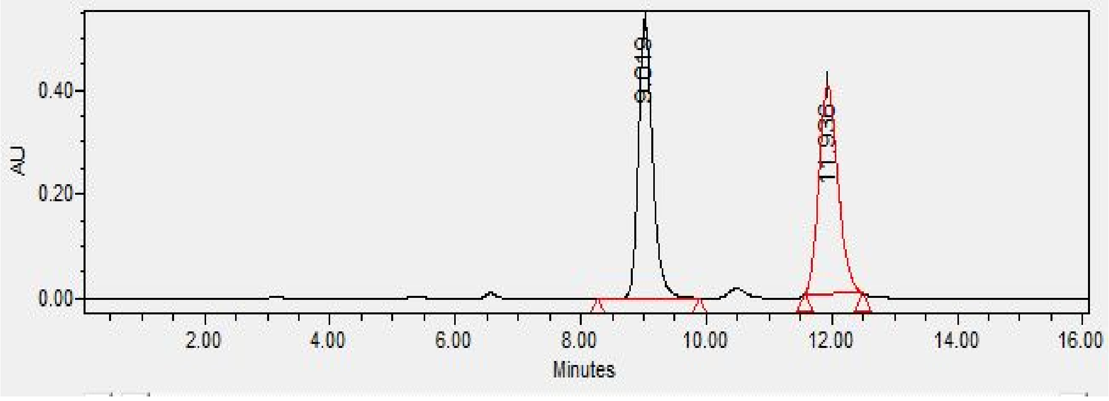

	Name	Retention Time (min)	Area (µV*sec)	% Area	Height (µV)	Int Type	Amount	Units	Peak Type	Peak Codes
1		9.019	8438022	49.87	537759	bb			Unknown	
2		11.936	8480644	50.13	400048	bb			Unknown	

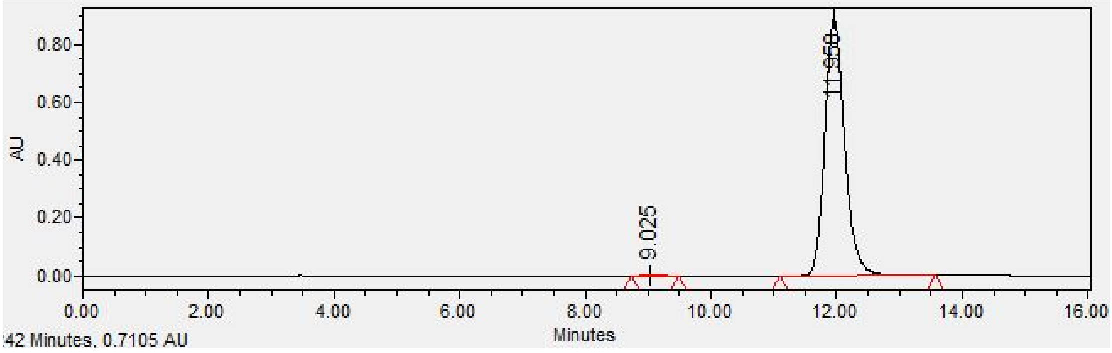

	Name	Retention Time (min)	Area (µV*sec)	% Area	Height (µV)	Int Type	Amount	Units	Peak Type	Peak Codes
1		9.025	62841	0.32	4005	bb			Unknown	
2		11.958	19567355	99.68	888104	bb			Unknown	

(*R*)-N-(dibenzo[b,d]furan-2-yl)-3-(diphenylphosphorothioyl)butanamide (3t)

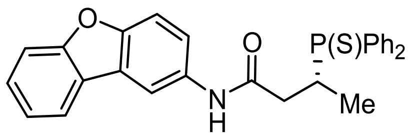

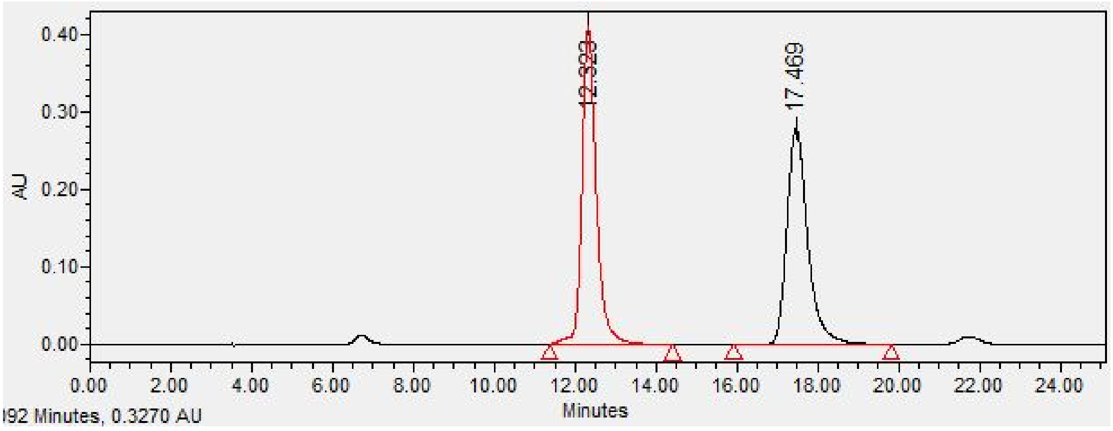

	Name	Retention Time (min)	Area (µV*sec)	% Area	Height (µV)	Int Type	Amount	Units	Peak Type	Peak Codes
1		12.323	9997242	50.20	411090	bb			Unknown	
2		17.469	9917828	49.80	279888	bb			Unknown	

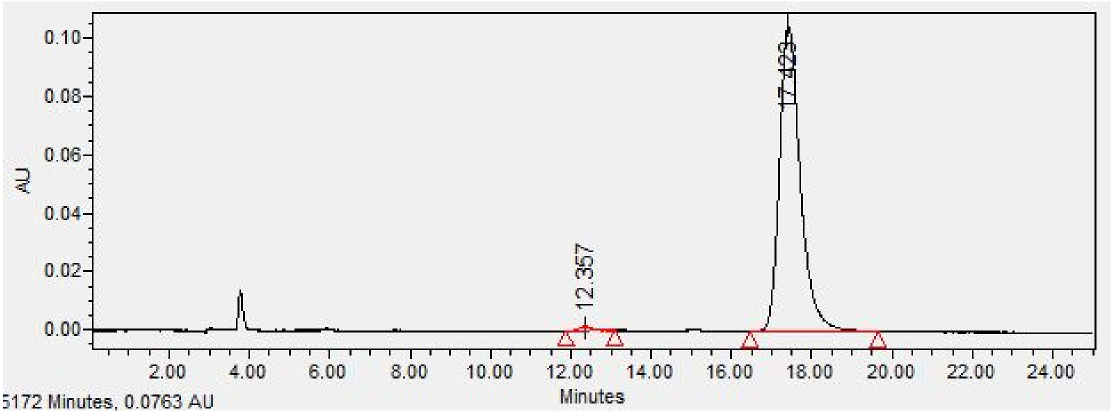

	Name	Retention Time (min)	Area (µV*sec)	% Area	Height (µV)	Int Type	Amount	Units	Peak Type	Peak Codes
1		12.357	31536	0.83	1390	bb			Unknown	
2		17.423	3788303	99.17	104568	bb			Unknown	

(*R*)-3-(diphenylphosphorothioyl)-N-(isoquinolin-4-yl)butanamide (3u)

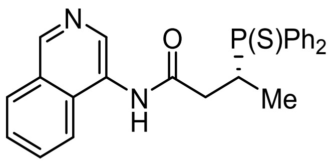

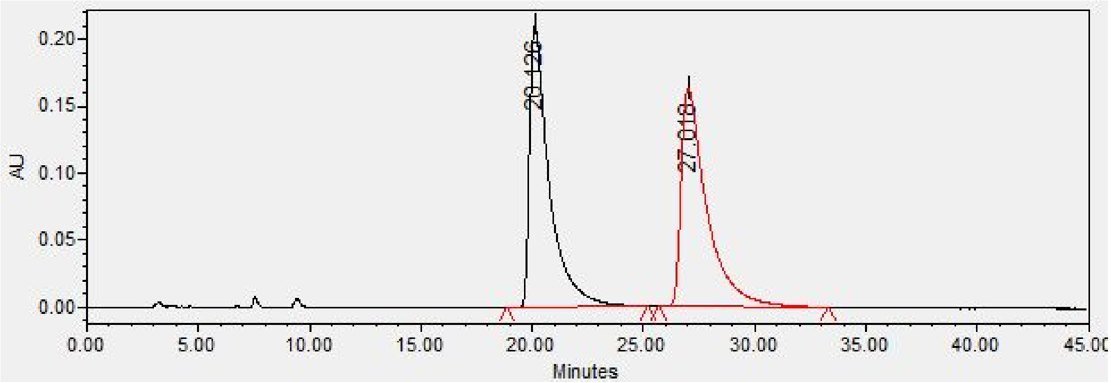

	Name	Retention Time (min)	Area (µV*sec)	% Area	Height (µV)	Int Type	Amount	Units	Peak Type	Peak Codes
1		20.126	12991370	49.81	211562	bb			Unknown	
2		27.018	13090487	50.19	162767	bb			Unknown	

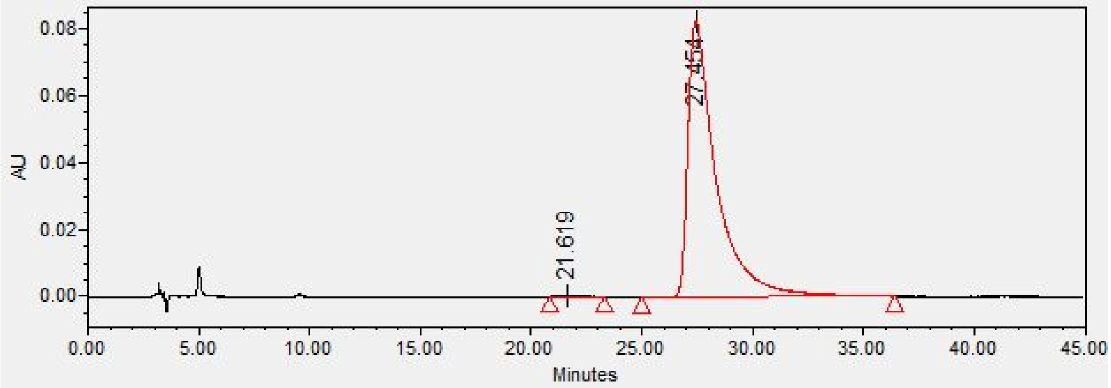

	Name	Retention Time (min)	Area (µV*sec)	% Area	Height (µV)	Int Type	Amount	Units	Peak Type	Peak Codes
1		21.619	26696	0.36	445	bb			Unknown	
2		27.454	7319666	99.64	82190	bb			Unknown	

(*R*)-3-(diphenylphosphorothioyl)-N-(thiophen-3-yl)butanamide (3v)

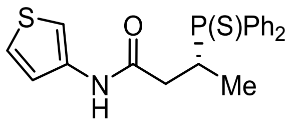

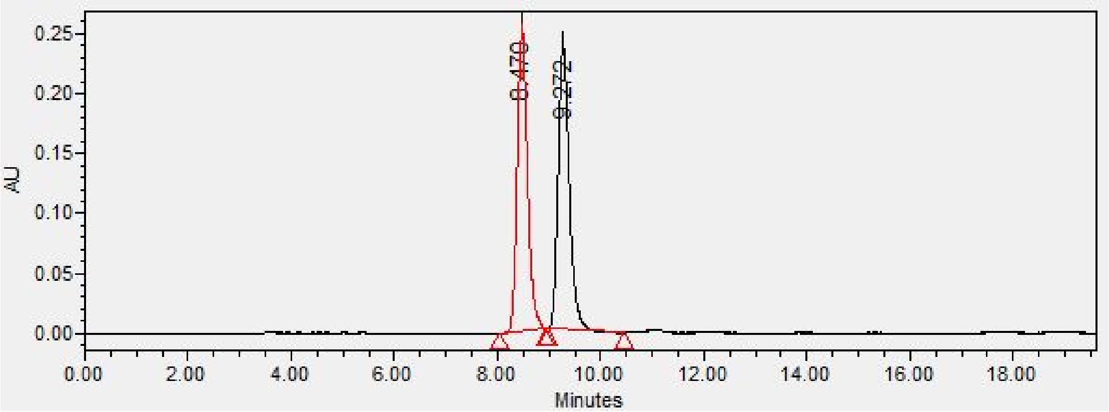

Name	Retention Time (min)	Area (μV*sec)	% Area	Height (μV)	Int Type	Amount	Units	Peak Type	Peak Codes
1	8.470	3487608	49.46	254984	bb			Unknown	
2	9.272	3563370	50.54	237979	bb			Unknown	

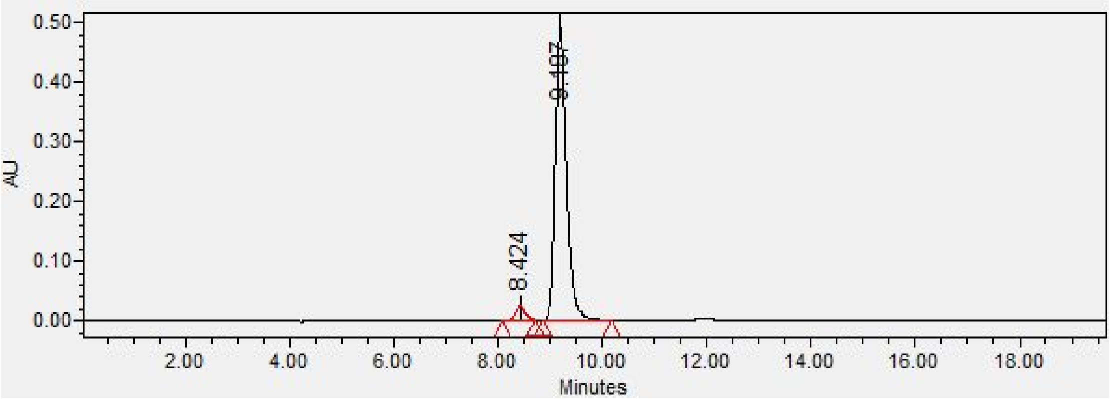

Name	Retention Time (min)	Area (μV*sec)	% Area	Height (μV)	Int Type	Amount	Units	Peak Type	Peak Codes
1	8.424	313873	4.08	24334	bb			Unknown	
2	9.187	7378351	95.92	497531	bb			Unknown	

(*R*)-N-benzhydryl-3-(diphenylphosphorothioyl)butanamide (3w)

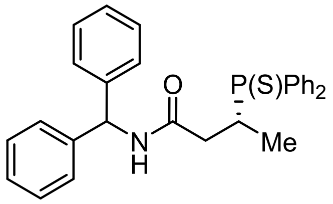

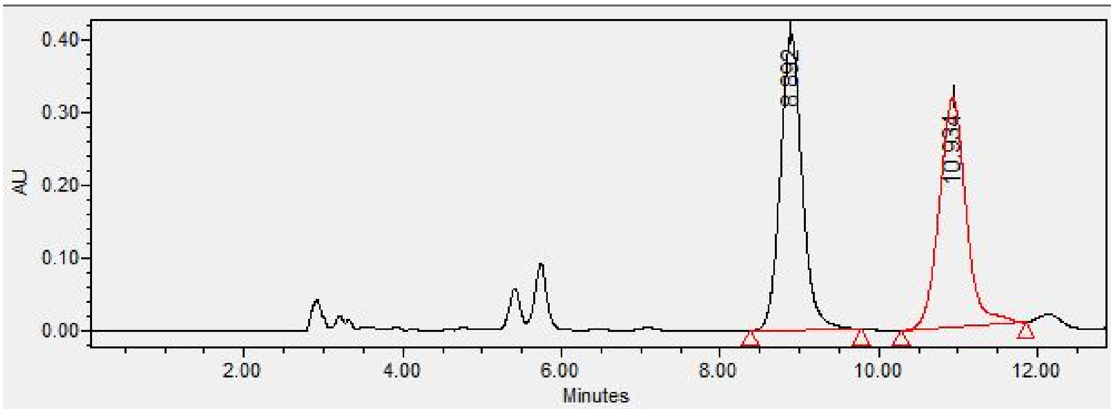

	Name	Retention Time (min)	Area (μV*sec)	% Area	Height (μV)	Int Type	Amount	Units	Peak Type	Peak Codes
1		8.892	7738729	51.22	407509	bb			Unknown	
2		10.934	7370568	48.78	314290	bb			Unknown	

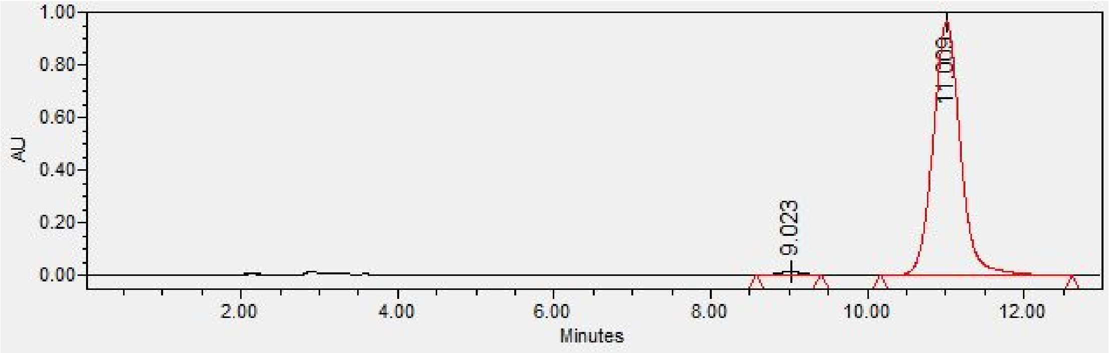

	Name	Retention Time (min)	Area (μV*sec)	% Area	Height (μV)	Int Type	Amount	Units	Peak Type	Peak Codes
1		9.023	270690	1.15	14357	bb			Unknown	
2		11.009	23252773	98.85	964768	bb			Unknown	

(*R*)-3-(diphenylphosphorothioyl)-N-(thiophen-3-ylmethyl)butanamide (3x)

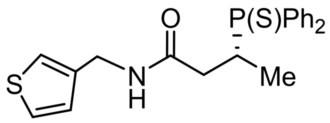

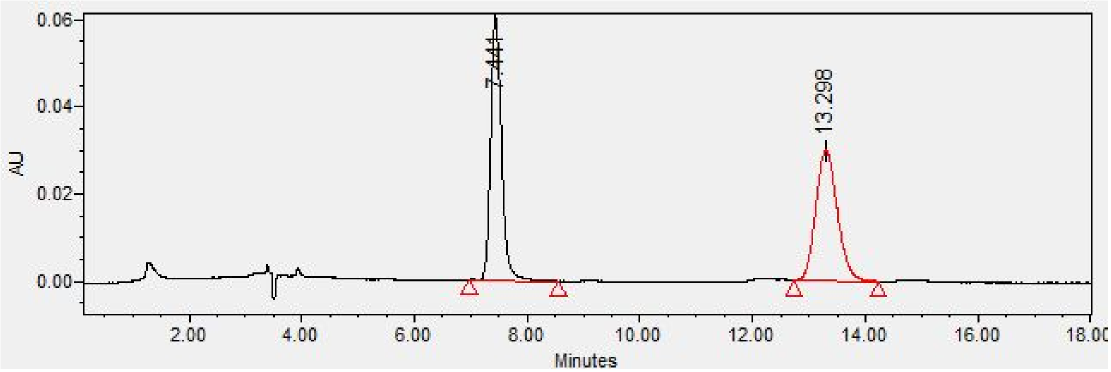

	Name	Retention Time (min)	Area (µV*sec)	% Area	Height (µV)	Int Type	Amount	Units	Peak Type	Peak Codes
1		7.441	805493	50.36	59971	bb			Unknown	
2		13.298	793949	49.64	29990	bb			Unknown	

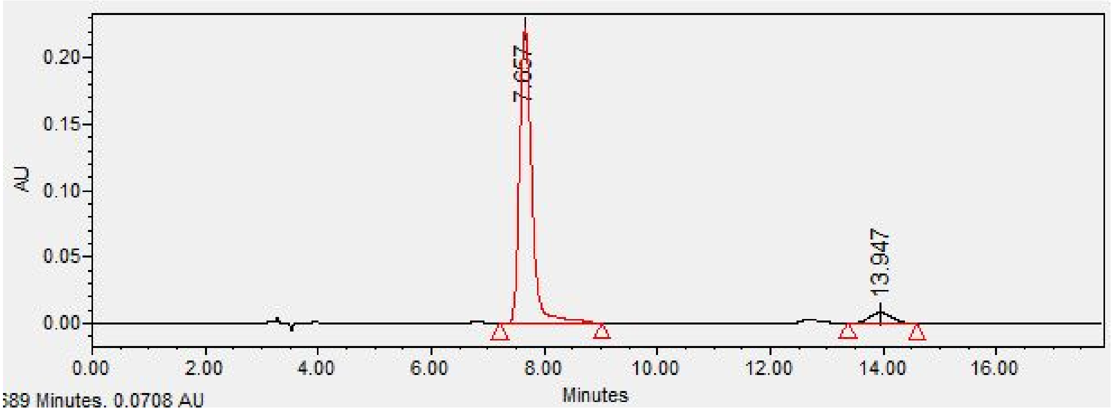

	Name	Retention Time (min)	Area (µV*sec)	% Area	Height (µV)	Int Type	Amount	Units	Peak Type	Peak Codes
1		7.657	3343263	93.77	221881	bb			Unknown	
2		13.947	221992	6.23	8015	bb			Unknown	

(*R*)-N-benzyl-3-(diphenylphosphorothioyl)butanamide (3y)

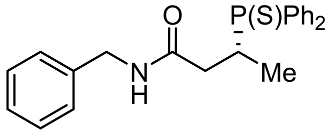

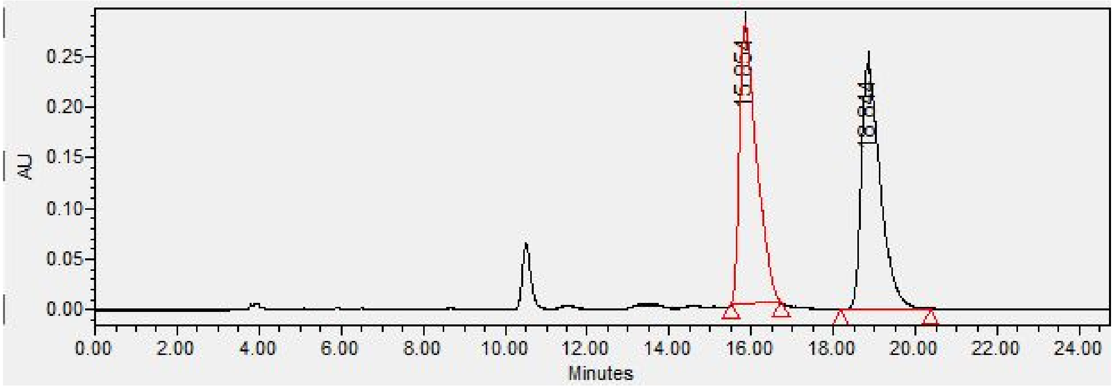

	Name	Retention Time (min)	Area (µV*sec)	% Area	Height (µV)	Int Type	Amount	Units	Peak Type	Peak Codes
1		15.854	7988408	50.49	276313	bb			Unknown	
2		18.844	7832388	49.51	242037	bb			Unknown	

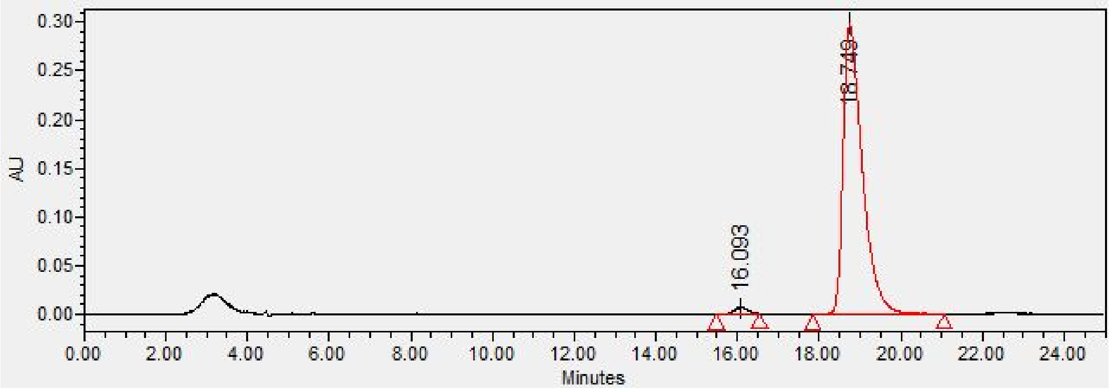

	Name	Retention Time (min)	Area (µV*sec)	% Area	Height (µV)	Int Type	Amount	Units	Peak Type	Peak Codes
1		16.093	163303	1.65	6915	bb			Unknown	
2		18.749	9737660	98.35	297959	bb			Unknown	

(*R*)-N-(2-bromobenzyl)-3-(diphenylphosphorothioyl)butanamide (3z)

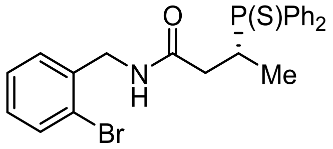

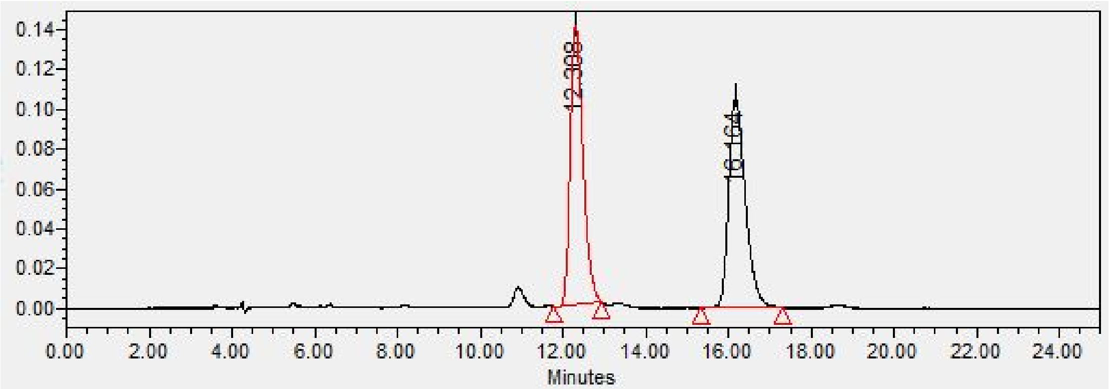

	Name	Retention Time (min)	Area (µV*sec)	% Area	Height (µV)	Int Type	Amount	Units	Peak Type	Peak Codes
1		12.308	2968706	50.12	139867	bb			Unknown	
2		16.164	2954724	49.88	106272	bb			Unknown	

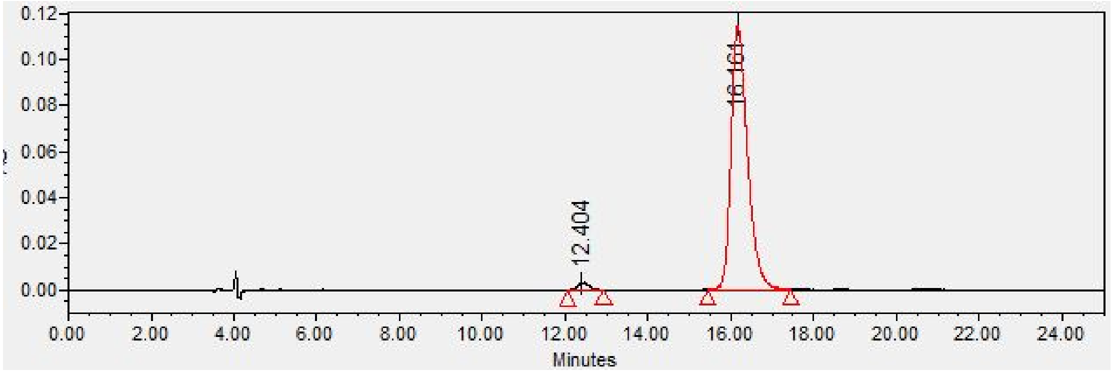

	Name	Retention Time (min)	Area (µV*sec)	% Area	Height (µV)	Int Type	Amount	Units	Peak Type	Peak Codes
1		12.404	66694	2.04	3417	bb			Unknown	
2		16.161	3199544	97.96	114554	bb			Unknown	

(*R*)-3-(diphenylphosphorothioyl)-N-methyl-N-phenylbutanamide (3ac)

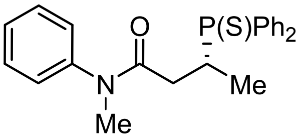

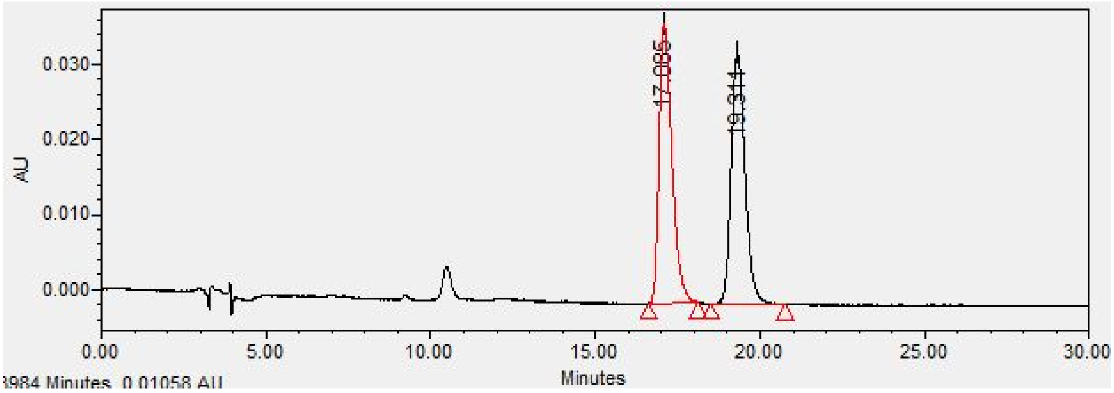

	Name	Retention Time (min)	Area (µV*sec)	% Area	Height (µV)	Int Type	Amount	Units	Peak Type	Peak Codes
1		17.085	995678	50.66	37227	bb			Unknown	
2		19.311	969881	49.34	33504	bb			Unknown	

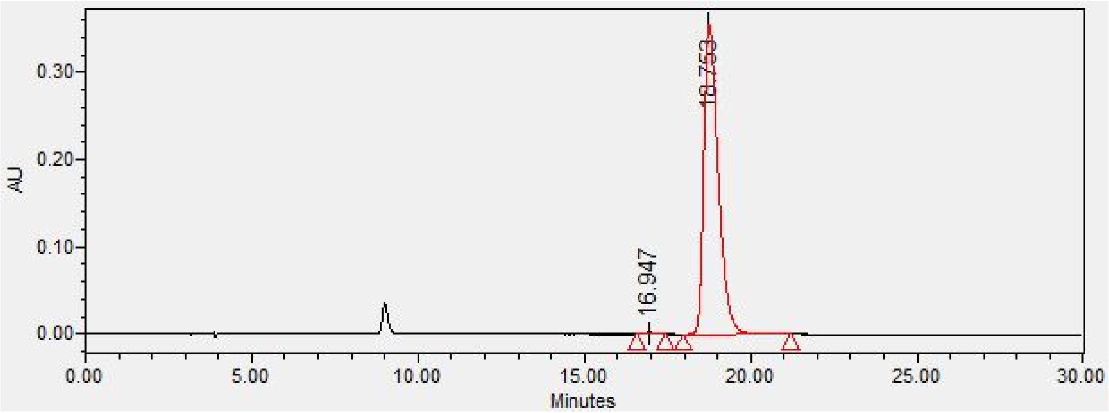

	Name	Retention Time (min)	Area (µV*sec)	% Area	Height (µV)	Int Type	Amount	Units	Peak Type	Peak Codes
1		16.947	53781	0.50	2131	bb			Unknown	
2		18.753	10784114	99.50	355443	bb			Unknown	

(*R*)-3-(diphenylphosphorothioyl)-N-phenylpentanamide (3ad)

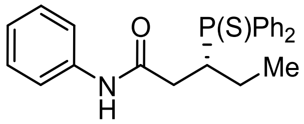

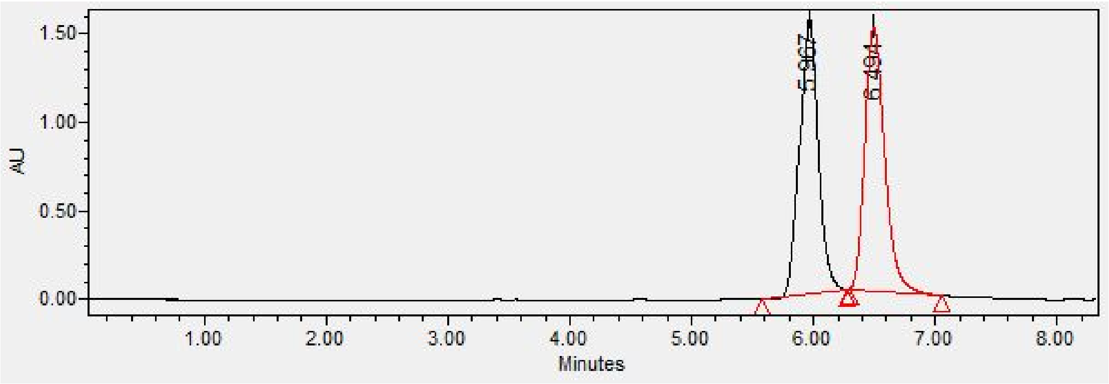

	Name	Retention Time (min)	Area (μV*sec)	% Area	Height (μV)	Int Type	Amount	Units	Peak Type	Peak Codes
1		5.967	16658364	49.64	1559953	bb			Unknown	
2		6.494	16902959	50.36	1488019	bb			Unknown	

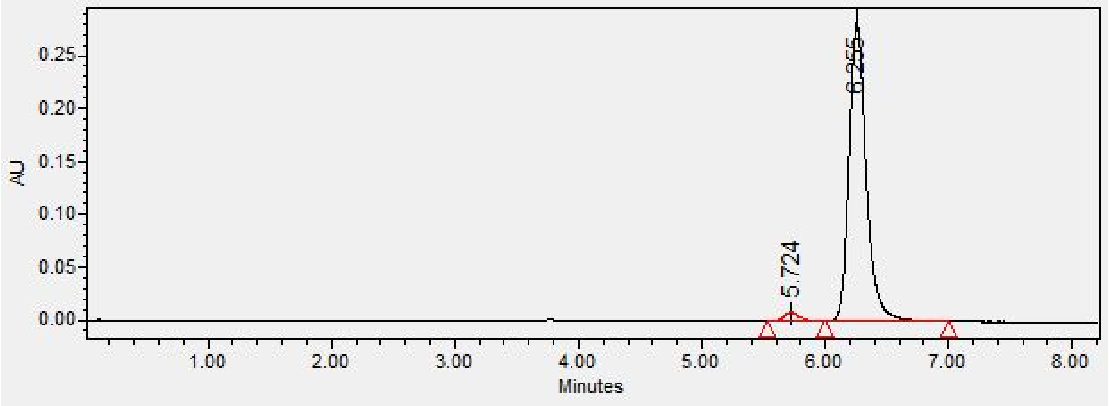

	Name	Retention Time (min)	Area (μV*sec)	% Area	Height (μV)	Int Type	Amount	Units	Peak Type	Peak Codes
1		5.724	70672	2.51	7962	BB			Unknown	
2		6.255	2748743	97.49	283560	BB			Unknown	

(*R*)-3-(di-*p*-tolylphosphorothioyl)-N-phenylbutanamide (3ae)

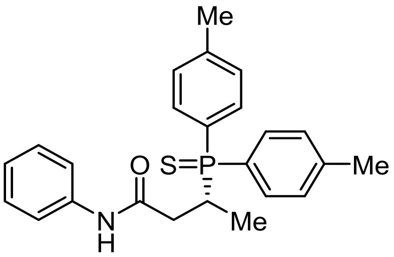

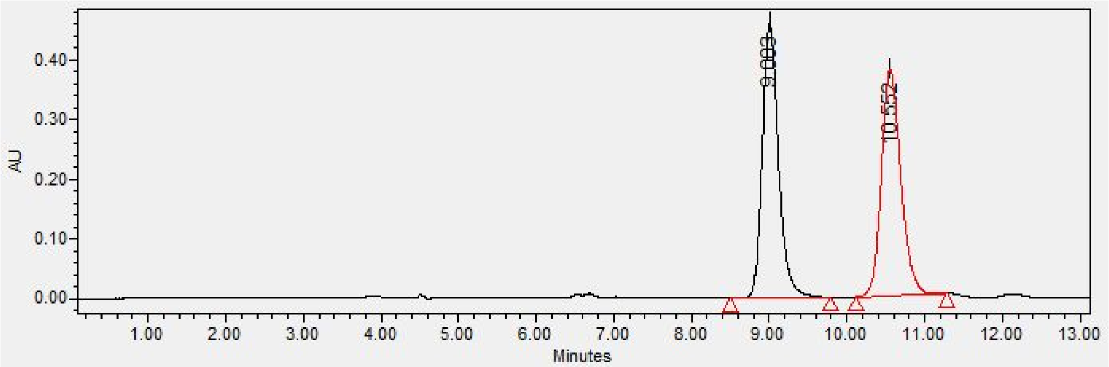

	Name	Retention Time (min)	Area (µV*sec)	% Area	Height (µV)	Int Type	Amount	Units	Peak Type	Peak Codes
1		9.003	6666097	49.76	460187	bb			Unknown	
2		10.552	6729130	50.24	380080	bb			Unknown	

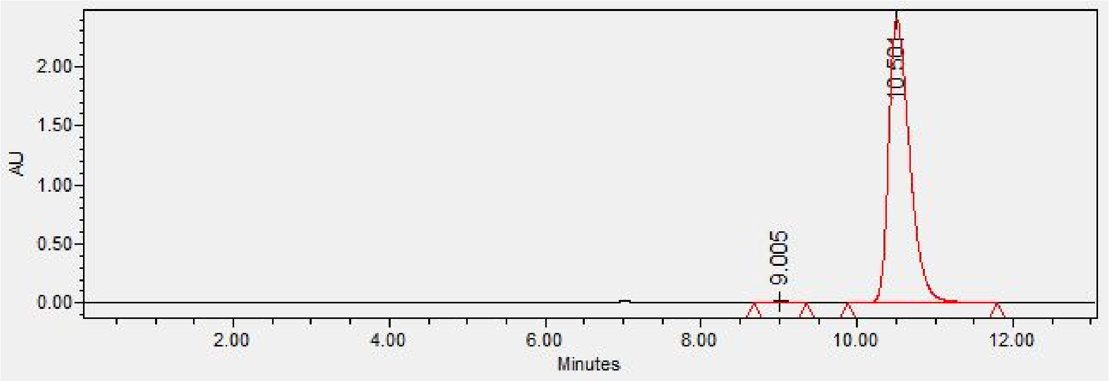

	Name	Retention Time (min)	Area (µV*sec)	% Area	Height (µV)	Int Type	Amount	Units	Peak Type	Peak Codes
1		9.005	220616	0.49	16101	bb			Unknown	
2		10.501	44851707	99.51	2404243	bb			Unknown	

(*R*)-3-(bis(3,5-dimethylphenyl)phosphorothioyl)-N-phenylbutanamide (3af)

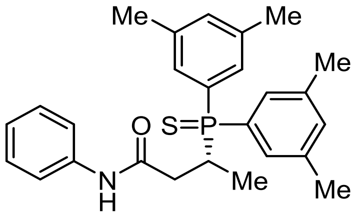

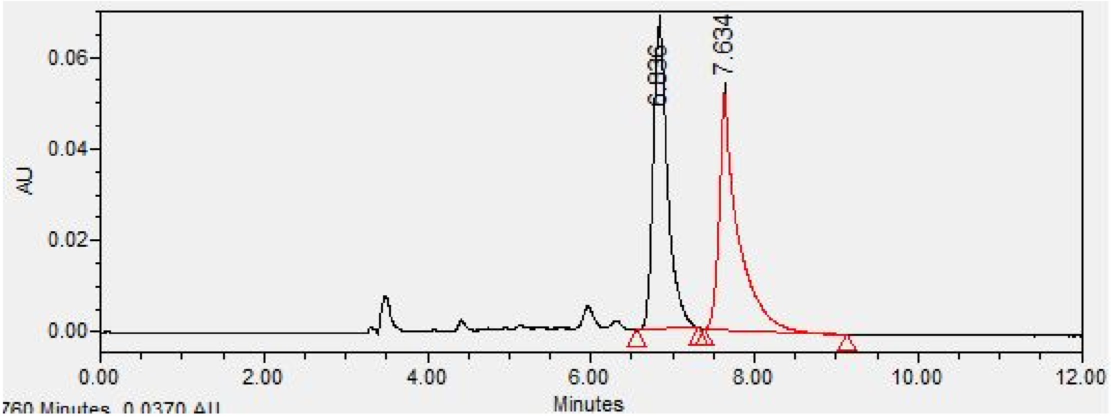

E	Name	Retention Time (min)	Area (µV*sec)	% Area	Height (µV)	Int Type	Amount	Units	Peak Type	Peak Codes
1		6.836	829776	50.62	66045	bb			Unknown	
2		7.634	809588	49.38	51705	bb			Unknown	

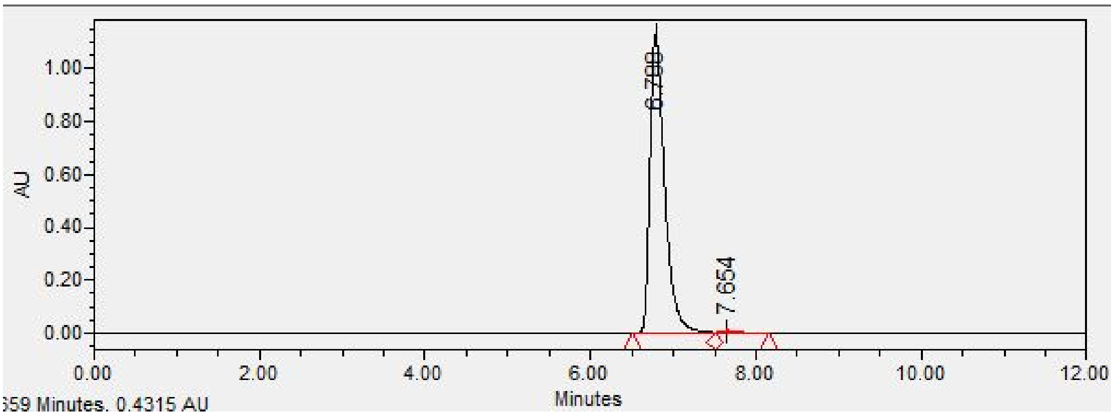

E	Name	Retention Time (min)	Area (µV*sec)	% Area	Height (µV)	Int Type	Amount	Units	Peak Type	Peak Codes
1		6.788	14105884	98.60	1130294	BV			Unknown	
2		7.654	200825	1.40	12307	Vb			Unknown	

(*R*)-3-(di-m-tolylphosphorothioyl)-N-phenylbutanamide (3ag)

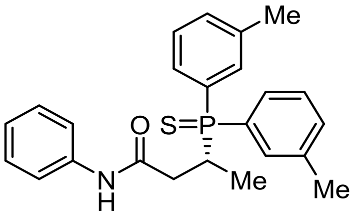

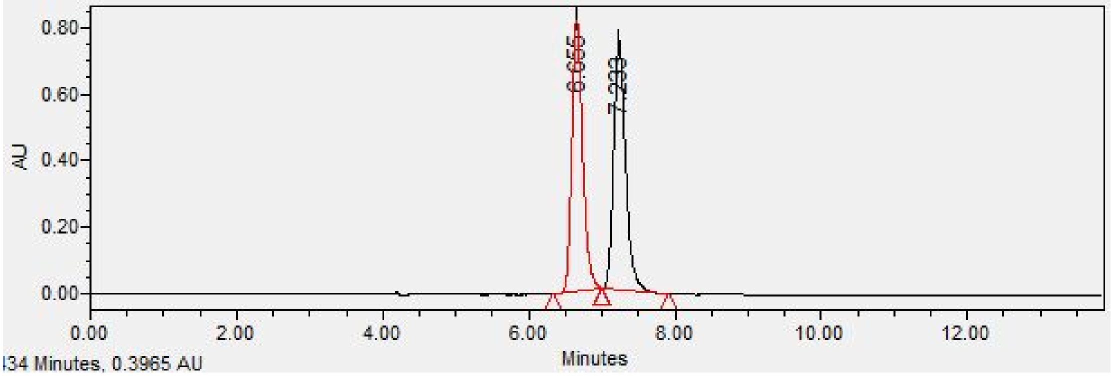

	Name	Retention Time (min)	Area (µV*sec)	% Area	Height (µV)	Int Type	Amount	Units	Peak Type	Peak Codes
1		6.655	8412687	49.69	815422	bb			Unknown	
2		7.233	8517641	50.31	749278	bb			Unknown	

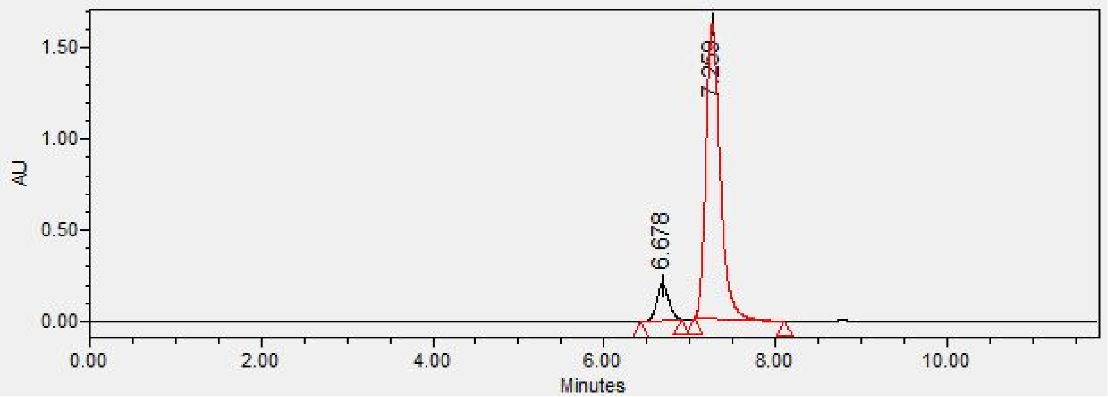

	Name	Retention Time (min)	Area (µV*sec)	% Area	Height (µV)	Int Type	Amount	Units	Peak Type	Peak Codes
1		6.678	1941797	9.39	197466	bb			Unknown	
2		7.259	18747395	90.61	1614951	bb			Unknown	

(*R*)-3-(bis(4-(tert-butyl)phenyl)phosphorothioyl)-N-phenylbutanamide (3ah)

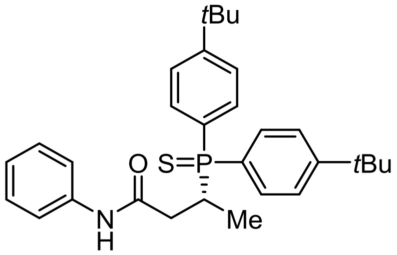

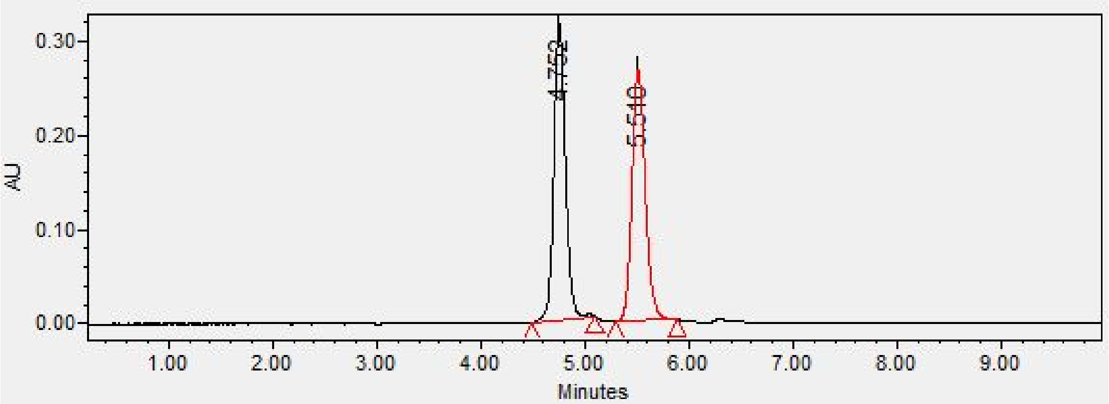

	Name	Retention Time (min)	Area (µV*sec)	% Area	Height (µV)	Int Type	Amount	Units	Peak Type	Peak Codes
1		4.752	2424955	50.27	315158	bb			Unknown	
2		5.510	2399155	49.73	266996	bb			Unknown	

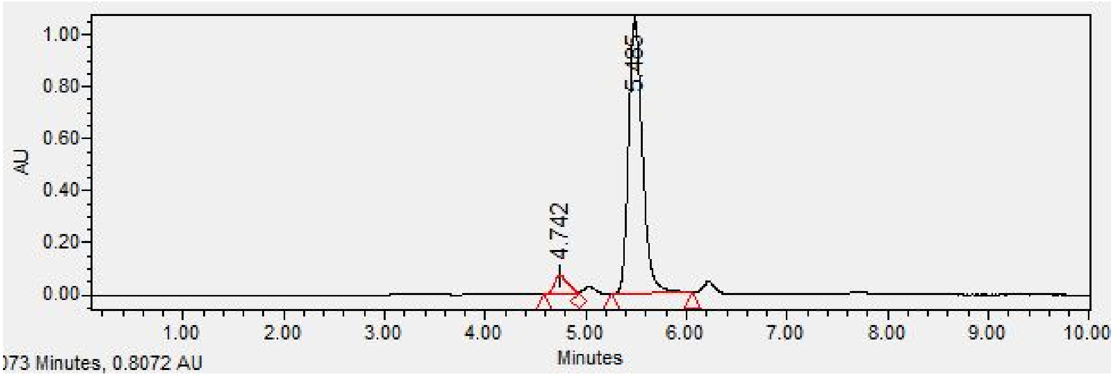

	Name	Retention Time (min)	Area (µV*sec)	% Area	Height (µV)	Int Type	Amount	Units	Peak Type	Peak Codes
1		4.742	644854	5.96	70632	bV			Unknown	
2		5.485	10168539	94.04	1056637	bb			Unknown	

(*R*)-3-(bis(4-chlorophenyl)phosphorothioyl)-N-phenylbutanamide (3ai)

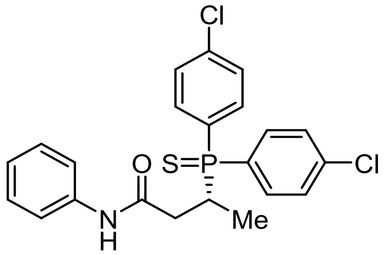

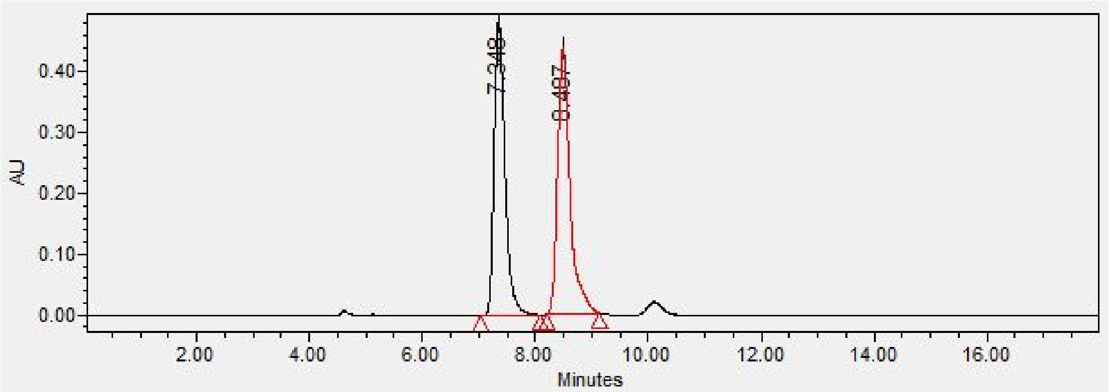

	Name	Retention Time (min)	Area (μV*sec)	% Area	Height (μV)	Int Type	Amount	Units	Peak Type	Peak Codes
1		7.348	6442058	49.09	483919	bb			Unknown	
2		8.487	6680751	50.91	436720	bb			Unknown	

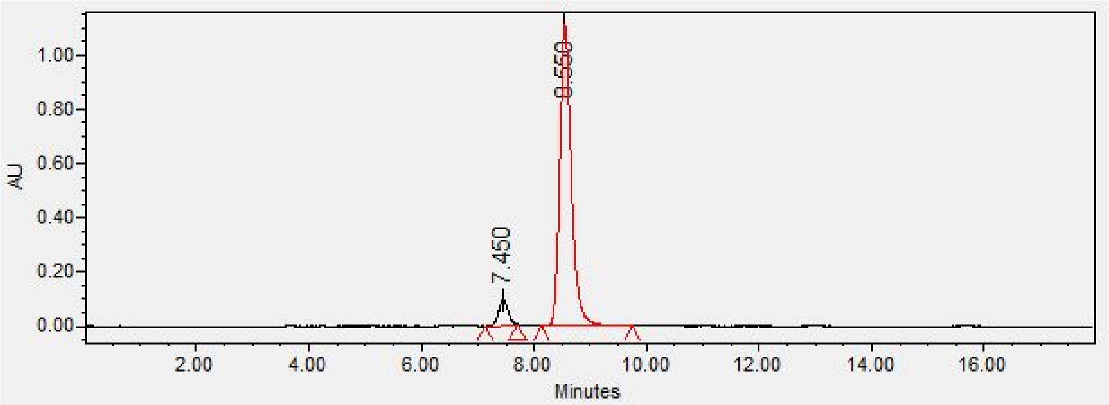

	Name	Retention Time (min)	Area (μV*sec)	% Area	Height (μV)	Int Type	Amount	Units	Peak Type	Peak Codes
1		7.450	1111171	6.53	96445	bb			Unknown	
2		8.550	15892712	93.47	1107574	bb			Unknown	

(*R*)-3-(bis(3-chlorophenyl)phosphorothioyl)-N-phenylbutanamide (3aj)

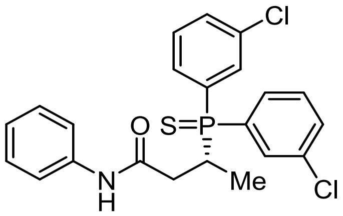

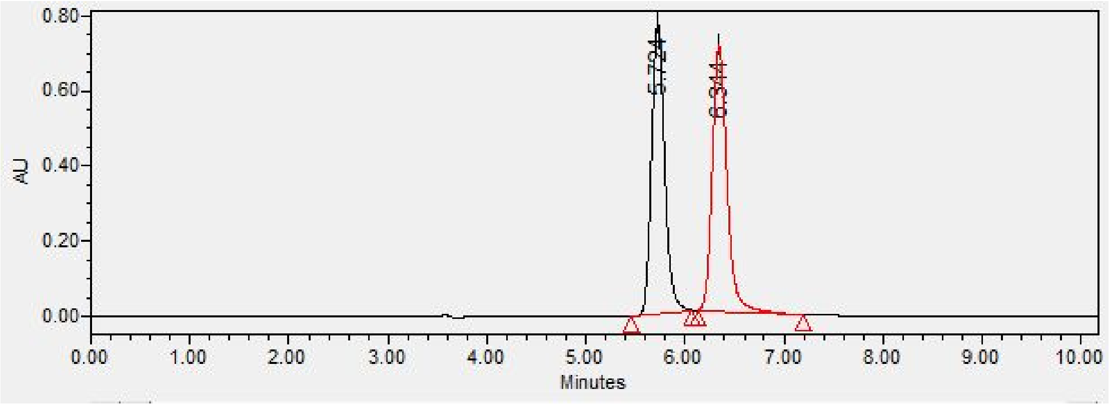

	Name	Retention Time (min)	Area (μV*sec)	% Area	Height (μV)	Int Type	Amount	Units	Peak Type	Peak Codes
1		5.724	7058106	49.20	775128	bb			Unknown	
2		6.344	7288606	50.80	707530	bb			Unknown	

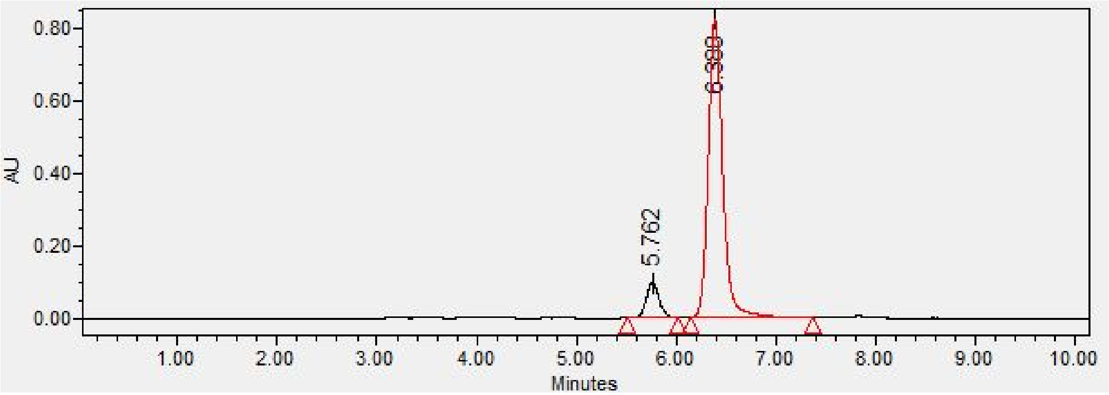

	Name	Retention Time (min)	Area (μV*sec)	% Area	Height (μV)	Int Type	Amount	Units	Peak Type	Peak Codes
1		5.762	845691	9.30	96096	bb			Unknown	
2		6.388	8244313	90.70	820581	bb			Unknown	

(*R*)-3-(bis(4-(trifluoromethoxy)phenyl)phosphorothioyl)-N-phenylbutanamide (3ak)

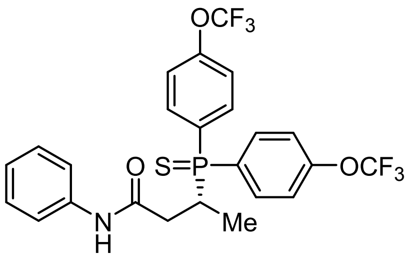

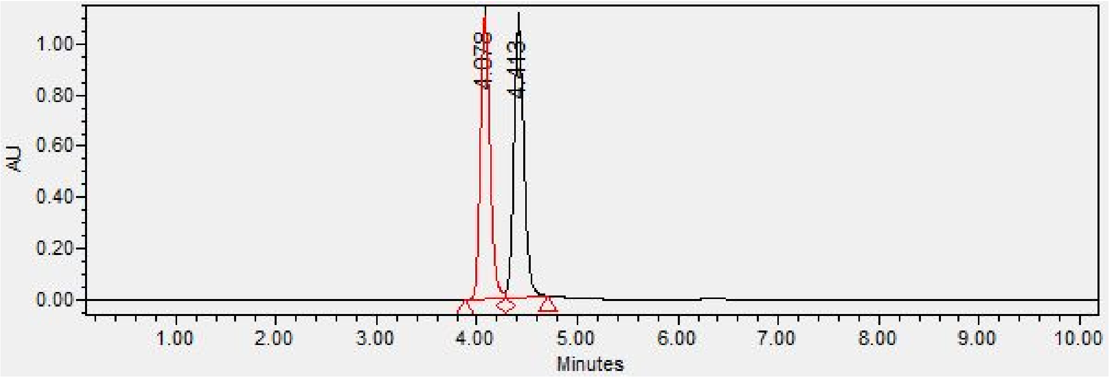

	Name	Retention Time (min)	Area (µV*sec)	% Area	Height (µV)	Int Type	Amount	Units	Peak Type	Peak Codes
1		4.078	7360874	49.66	1105319	Bv			Unknown	
2		4.413	7461717	50.34	1062450	vb			Unknown	

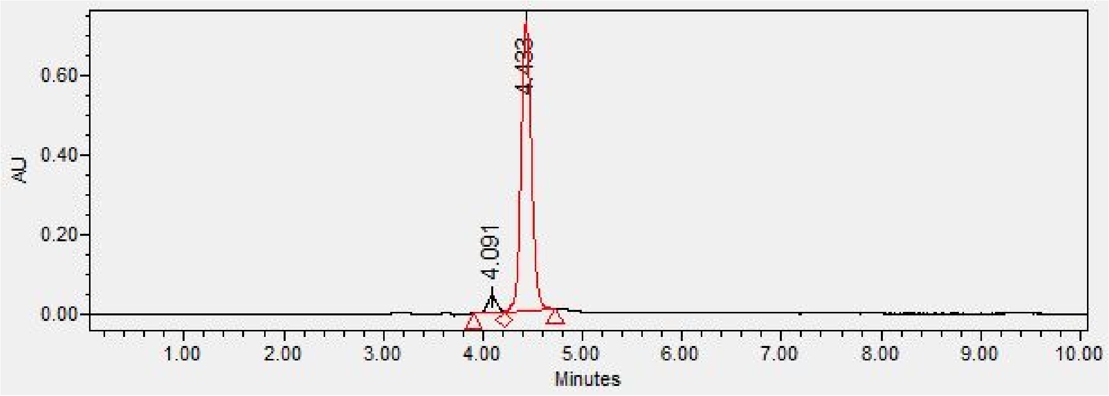

	Name	Retention Time (min)	Area (µV*sec)	% Area	Height (µV)	Int Type	Amount	Units	Peak Type	Peak Codes
1		4.091	261119	4.73	40280	bV			Unknown	
2		4.433	5264963	95.27	729760	Vb			Unknown	

(*R*)-3-(di([1,1'-biphenyl]-4-yl)phosphorothioyl)-N-phenylbutanamide (3al)

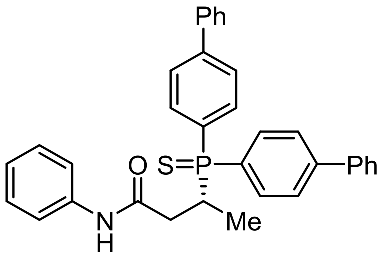

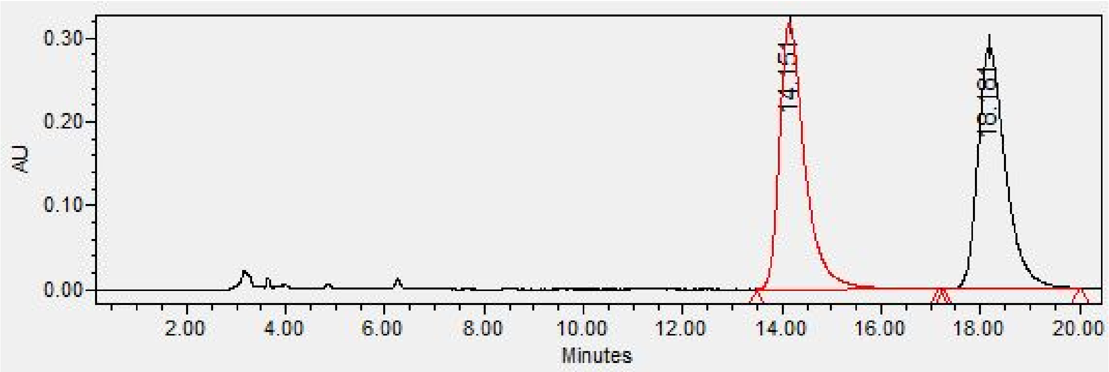

	Name	Retention Time (min)	Area (µV*sec)	% Area	Height (µV)	Int Type	Amount	Units	Peak Type	Peak Codes
1		14.151	11233098	50.23	317944	BB			Unknown	
2		18.181	11129331	49.77	287834	BB			Unknown	

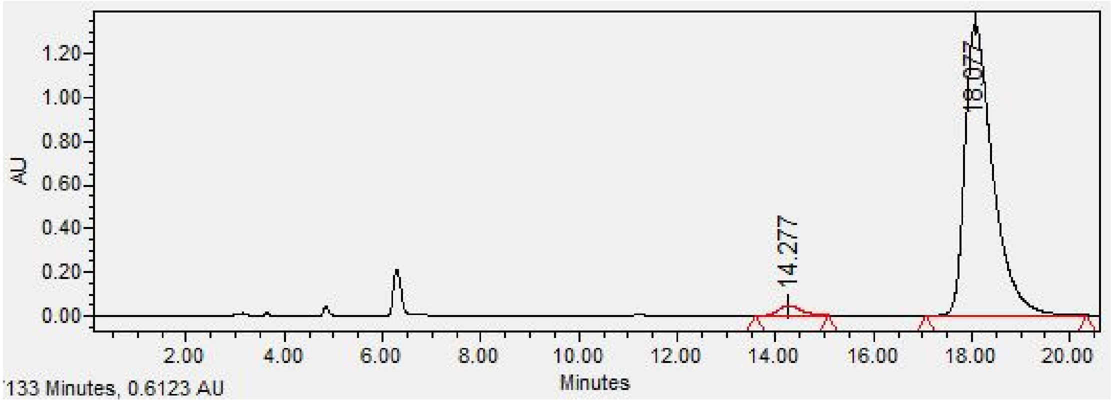

	Name	Retention Time (min)	Area (µV*sec)	% Area	Height (µV)	Int Type	Amount	Units	Peak Type	Peak Codes
1		14.277	1519710	2.79	45402	Bb			Unknown	
2		18.077	53003341	97.21	1334266	Bb			Unknown	

(*R*)-3-(di(naphthalen-2-yl)phosphorothioyl)-N-phenylbutanamide (3am)

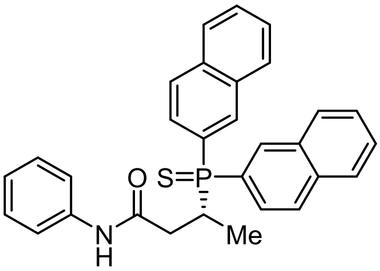

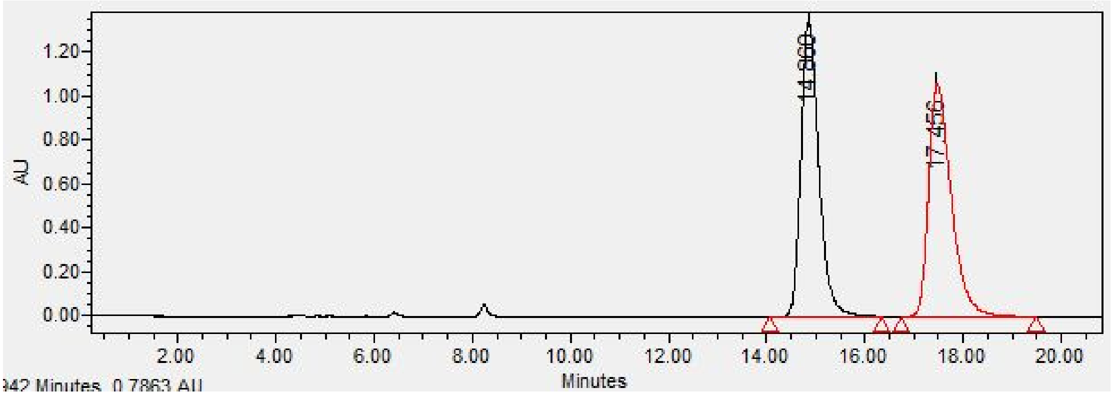

	Name	Retention Time (min)	Area (μV*sec)	% Area	Height (μV)	Int Type	Amount	Units	Peak Type	Peak Codes
1		14.860	34058084	49.98	1363424	bb			Unknown	
2		17.456	34079135	50.02	1061504	bb			Unknown	

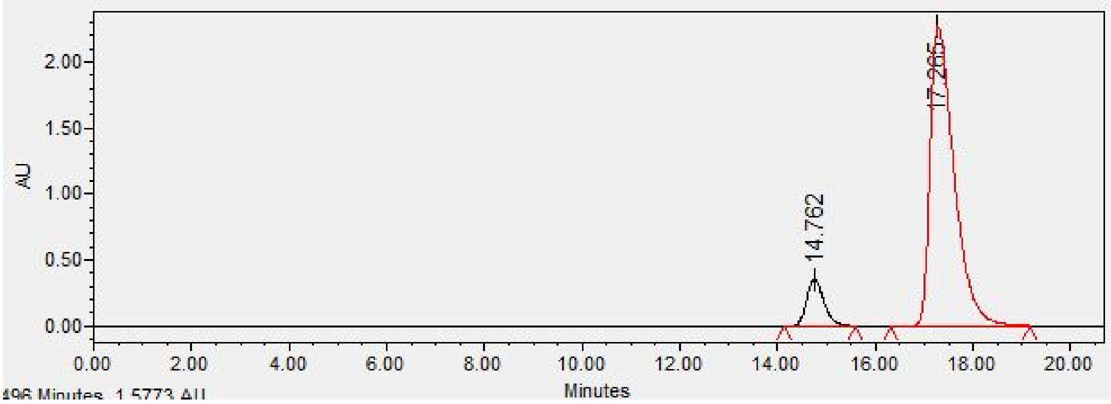

	Name	Retention Time (min)	Area (μV*sec)	% Area	Height (μV)	Int Type	Amount	Units	Peak Type	Peak Codes
1		14.762	8828917	10.19	360644	bb			Unknown	
2		17.265	77779696	89.81	2269435	bb			Unknown	

(*R*)-diphenyl(4-(phenylamino)butan-2-yl)phosphine sulfide (5)

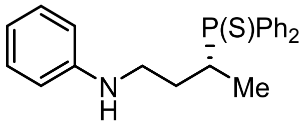

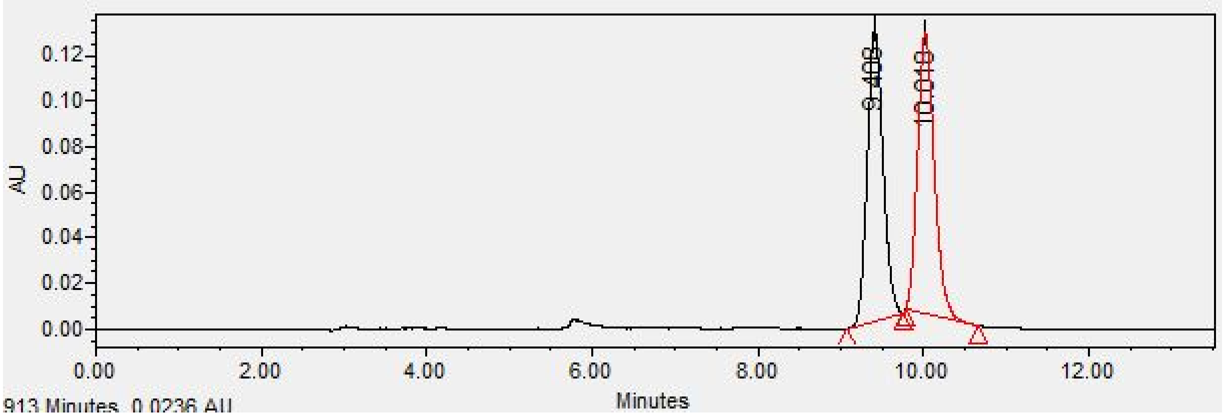

	Name	Retention Time (min)	Area (µV*sec)	% Area	Height (µV)	Int Type	Amount	Units	Peak Type	Peak Codes
1		9.408	1697265	49.45	127770	bb			Unknown	
2		10.018	1735100	50.55	123009	bb			Unknown	

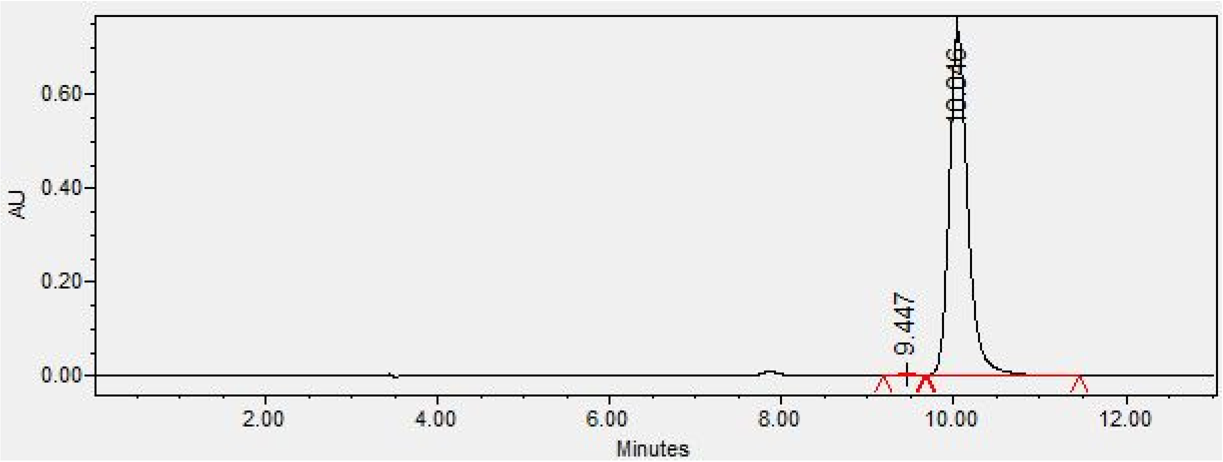

	Name	Retention Time (min)	Area (µV*sec)	% Area	Height (µV)	Int Type	Amount	Units	Peak Type	Peak Codes
1		9.447	48684	0.44	4175	bb			Unknown	
2		10.046	11008572	99.56	734257	bb			Unknown	

(*R*)-diphenyl(4-(phenyl(pyridin-4-yl)amino)butan-2-yl)phosphine sulfide (6)

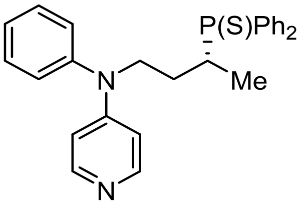

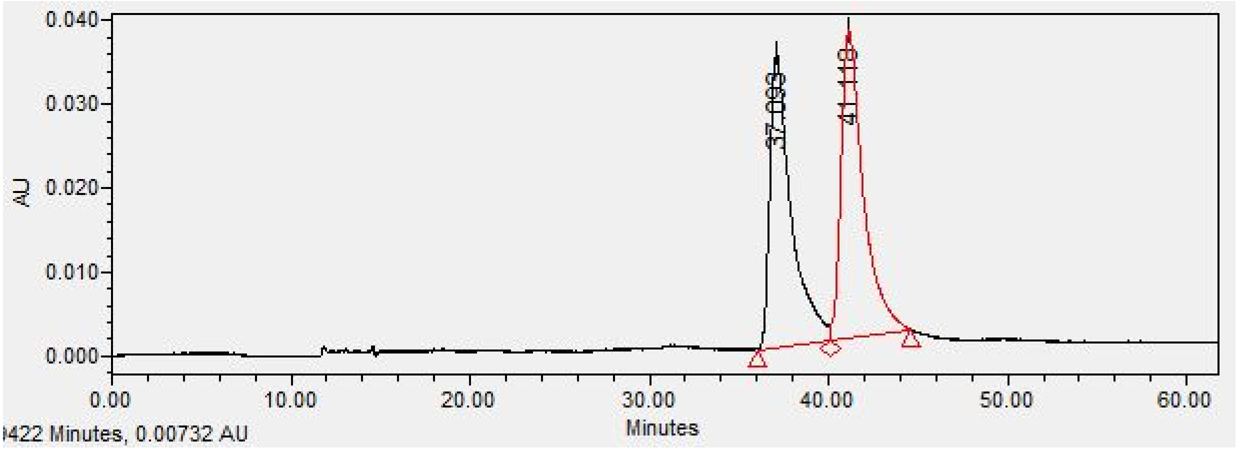

	Name	Retention Time (min)	Area (μV*sec)	% Area	Height (μV)	Int Type	Amount	Units	Peak Type	Peak Codes
1		37.093	2928317	48.04	35033	Bv			Unknown	
2		41.118	3167043	51.96	36724	vb			Unknown	

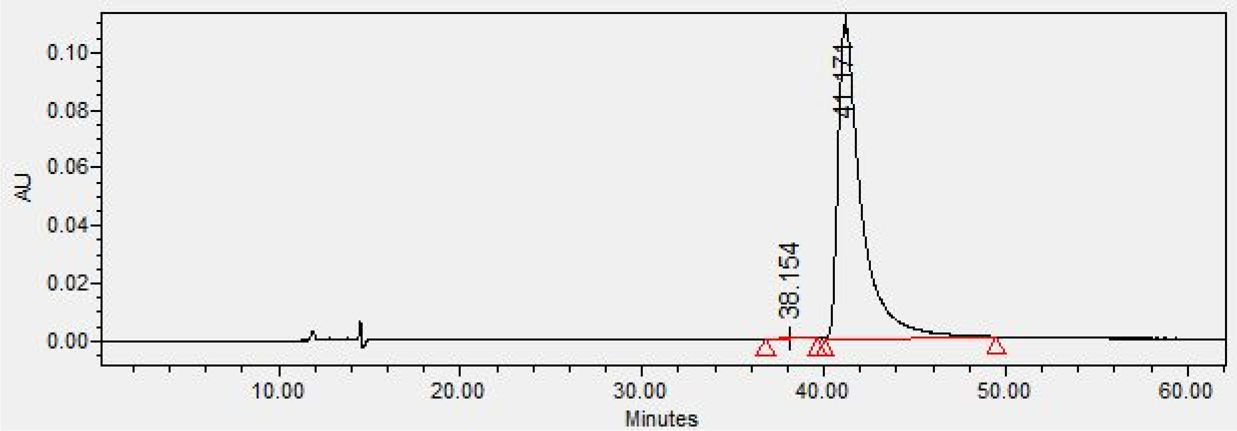

	Name	Retention Time (min)	Area (μV*sec)	% Area	Height (μV)	Int Type	Amount	Units	Peak Type	Peak Codes
1		38.154	33729	0.34	408	bb			Unknown	
2		41.171	9747928	99.66	109442	Bb			Unknown	

(*R*)-1-(3-(diphenylphosphorothioyl)butyl)-1-phenyl-3-(4-(trifluoromethyl)phenyl) thiourea (7)

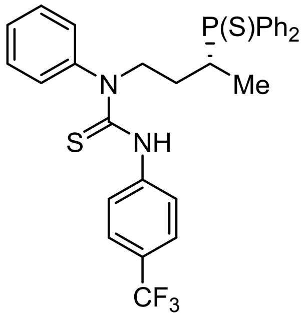

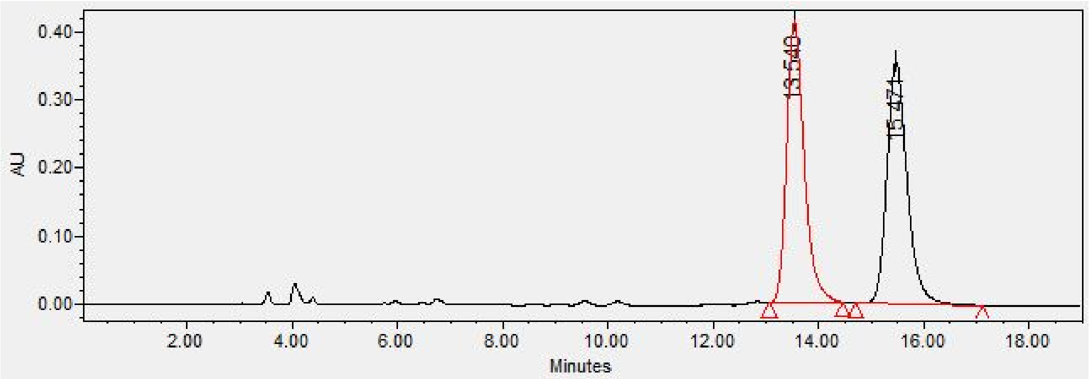

	Name	Retention Time (min)	Area (μV*sec)	% Area	Height (μV)	Int Type	Amount	Units	Peak Type	Peak Codes
1		13.540	9616538	50.44	413277	bb			Unknown	
2		15.471	9447158	49.56	355544	bb			Unknown	

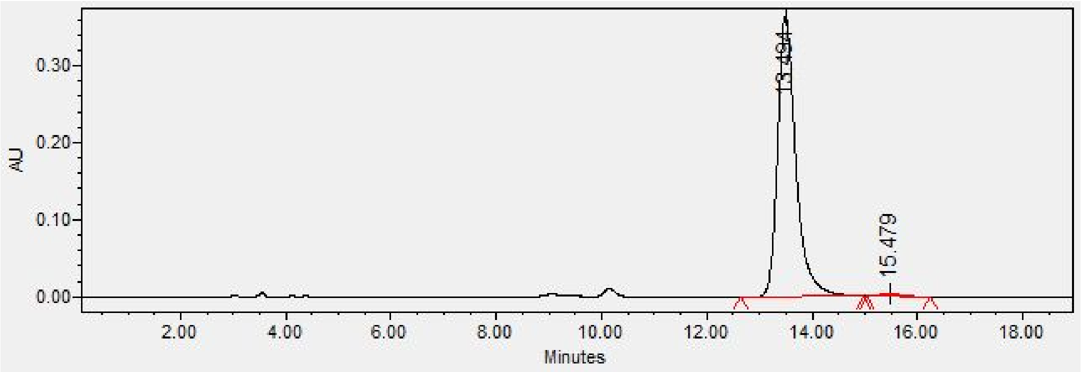

	Name	Retention Time (min)	Area (μV*sec)	% Area	Height (μV)	Int Type	Amount	Units	Peak Type	Peak Codes
1		13.494	8766947	99.04	364825	bb			Unknown	
2		15.479	84614	0.96	3347	bb			Unknown	

12. X-ray crystal structure of 3j

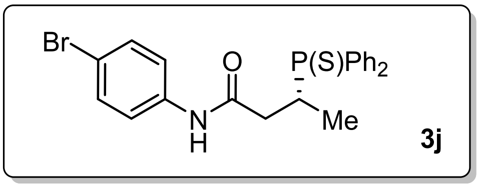

Crystal data and structure refinement for ZJ_4_Br_0m.

Identification code	ZJ_4_Br_0m
Empirical formula	C22H21BrNOPS
Formula weight	458.34
Temperature/K	100.0(2)
Crystal system	monoclinic
Space group	P21
a/Å	9.2719(7)
b/Å	9.2834(7)
c/Å	11.9905(9)
α/°	90
β/°	93.150(3)
γ/°	90
Volume/Å3	1030.52(13)

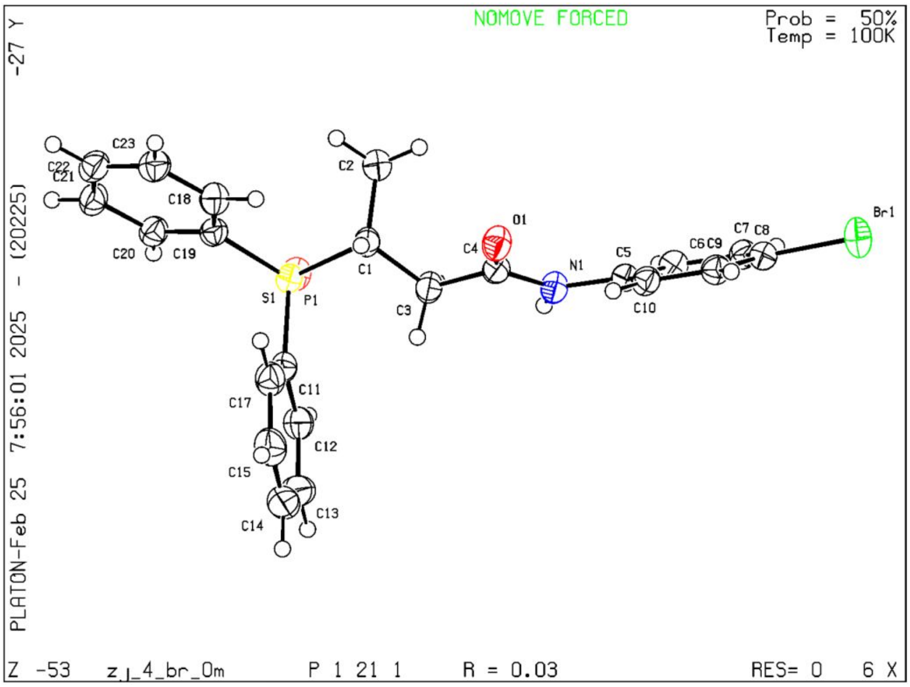

13. Rerference

1. Airiau, E.; Spangenberg, T.; Girard, N.; Schoenfelder, A.; Salvadori, J.; Taddei, M.; Mann, A., A general approach to aza-heterocycles by means of domino sequences driven by hydroformylation. *Chem. Eur. J.* **2008,** *14*, 10938-10948.
2. Derosa, J.; Kleinmans, R.; Tran, V. T.; Karunananda, M. K.; Wisniewski, S. R.; Eastgate, M. D.; Engle, K. M., Nickel-Catalyzed 1,2-Diarylation of Simple Alkenyl Amides. *J Am Chem Soc* **2018,** *140*, 17878-17883.
3. Nie, S. Z.; Davison, R. T.; Dong, V. M., Enantioselective Coupling of Dienes and Phosphine Oxides. *J. Am. Chem. Soc.* **2018,** *140*, 16450-16454.
4. Lee, C.; Kang, H. J.; Seo, H.; Hong, S., Nickel-Catalyzed Regio- and Enantioselective Hydroamination of Unactivated Alkenes Using Carbonyl Directing Groups. *J. Am. Chem. Soc.* **2022,** *144*, 9091-9100.
5. Yang, P.-F.; Liang, J.-X.; Zhao, H.-T.; Shu, W., Access to Enantioenriched 1,n-Diamines via Ni-Catalyzed Hydroamination of Unactivated Alkenes with Weakly Coordinating Groups. *ACS Catal.* **2022,** *12*, 9638-9645.
6. Yang, P.-F.; Zhu, L.; Liang, J.-X.; Zhao, H.-T.; Zhang, J.-X.; Zeng, X.-W.; Ouyang, Q.; Shu, W., Regio- and Enantioselective Hydroalkylations of Unactivated Olefins Enabled by Nickel Catalysis: Reaction Development and Mechanistic Insights. *ACS Catal.* **2022,** *12*, 5795-5805.
7. Wang, D. M.; She, L. Q.; Yuan, H.; Wu, Y.; Tang, Y.; Wang, P., Ligand-Enabled Ni(II) -Catalyzed Hydroxylarylation of Alkenes with Molecular Oxygen. *Angew. Chem. Int. Ed.* **2023,** *62*, e202304573.
8. Jankins, T. C.; Rubel, C. Z.; Ho, H. C.; Martin-Montero, R.; Engle, K. M., Tungsten-catalyzed stereodivergent isomerization of terminal olefins. *Chem. Sci.* **2025,** *16*, 2307-2315.
9. Nurseiit, A.; Janabel, J.; Gudun, K. A.; Kassymbek, A.; Segizbayev, M.; Seilkhanov, T. M.; Khalimon, A. Y., Bench-Stable Cobalt Pre-Catalysts for Mild Hydrosilative Reduction of Tertiary Amides to Amines and Beyond. *ChemCatChem* **2019,** *11*, 790-798.
10. Zhou, C.; Lei, T.; Wei, X.-Z.; Ye, C.; Liu, Z.; Chen, B.; Tung, C.-H.; Wu, L.-Z., Metal-Free, Redox-Neutral, Site-Selective Access to Heteroarylamine via Direct Radical–Radical Cross-Coupling Powered by Visible Light Photocatalysis. *J. Am. Chem. Soc.* **2020,** *142*, 16805-16813.
11. Grimme, S., Exploration of Chemical Compound, Conformer, and Reaction Space with Meta-Dynamics Simulations Based on Tight-Binding Quantum Chemical Calculations. *J. Chem. Theory Comput.* **2019,** *15*, 2847-2862.
12. Pracht, P.; Bohle, F.; Grimme, S., Automated exploration of the low-energy chemical space with fast quantum chemical methods. *Phys. Chem. Chem. Phys.* **2020,** *22*, 7169-7192.
13. Grimme, S.; Bannwarth, C.; Shushkov, P., A Robust and Accurate Tight-Binding Quantum Chemical Method for Structures, Vibrational Frequencies, and Noncovalent Interactions of Large Molecular Systems Parametrized for All spd-Block Elements (Z = 1-86). *J. Chem. Theory Comput.* **2017,** *13*, 1989-2009.

14. Bannwarth, C.; Ehlert, S.; Grimme, S., GFN2-xTB-An Accurate and Broadly Parametrized Self-Consistent Tight-Binding Quantum Chemical Method with Multipole Electrostatics and Density-Dependent Dispersion Contributions. *J. Chem. Theory Comput.* **2019,** *15*, 1652-1671.
15. Bannwarth, C.; Caldeweyher, E.; Ehlert, S.; Hansen, A.; Pracht, P.; Seibert, J.; Spicher, S.; Grimme, S., Extended tight-binding quantum chemistry methods. *Wiley Interdiscip. Rev. Comput. Mol. Sci.* **2021**, *11*, E1493.
16. Frisch, M. J et al., Gaussian 16, Revision B.01. 2016.
17. Yu, H. S.; He, X.; Li, S. L.; Truhlar, D. G., MN15: A Kohn–Sham global-hybrid exchange–correlation density functional with broad accuracy for multi-reference and single-reference systems and noncovalent interactions. *Chem. Sci.* **2016,** *7*, 5032-5051.
18. Weigend, F.; Ahlrichs, R., Balanced basis sets of split valence, triple zeta valence and quadruple zeta valence quality for H to Rn: Design and assessment of accuracy. *Phys. Chem. Chem. Phys.* **2005,** *7*, 3297-305.
19. Weigend, F., Accurate Coulomb-fitting basis sets for H to Rn. *Phys. Chem. Chem. Phys.* **2006,** *8*, 1057-65.
20. Achar, T. K.; Zhang, X.; Mondal, R.; Shanavas, M. S.; Maiti, S.; Maity, S.; Pal, N.; Paton, R. S.; Maiti, D., Palladium-Catalyzed Directed meta-Selective C-H Allylation of Arenes: Unactivated Internal Olefins as Allyl Surrogates. *Angew. Chem. Int. Ed.* **2019,** *58*, 10353-10360.
21. Guin, S.; Dolui, P.; Zhang, X.; Paul, S.; Singh, V. K.; Pradhan, S.; Chandrashekar, H. B.; Anjana, S. S.; Paton, R. S.; Maiti, D., Iterative Arylation of Amino Acids and Aliphatic Amines via delta-C(sp(3))-H Activation: Experimental and Computational Exploration. *Angew. Chem. Int. Ed.* **2019,** *58*, 5633-5638.
22. Porey, S.; Zhang, X.; Bhowmick, S.; Kumar Singh, V.; Guin, S.; Paton, R. S.; Maiti, D., Alkyne Linchpin Strategy for Drug:Pharmacophore Conjugation: Experimental and Computational Realization of a Meta-Selective Inverse Sonogashira Coupling. *J. Am. Chem. Soc.* **2020,** *142*, 3762-3774.
23. Sinha, S. K.; Panja, S.; Grover, J.; Hazra, P. S.; Pandit, S.; Bairagi, Y.; Zhang, X.; Maiti, D., Dual Ligand Enabled Nondirected C-H Chalcogenation of Arenes and Heteroarenes. *J. Am. Chem. Soc.* **2022,** *144*, 12032-12042.
24. Das, J.; Ali, W.; Ghosh, A.; Pal, T.; Mandal, A.; Teja, C.; Dutta, S.; Pothikumar, R.; Ge, H.; Zhang, X.; Maiti, D., Access to unsaturated bicyclic lactones by overriding conventional C(sp(3))-H site selectivity. *Nat. Chem.* **2023,** *15*, 1626-1635.
25. Dutta, U.; Prakash, G.; Devi, K.; Borah, K.; Zhang, X.; Maiti, D., Directing group assisted para-selective C-H alkynylation of unbiased arenes enabled by rhodium catalysis. *Chem. Sci.* **2023,** *14*, 11381-11388.
26. Porey, S.; Bairagi, Y.; Guin, S.; Zhang, X.; Maiti, D., Nondirected C–H/C–F Coupling for the Synthesis of α-Fluoro Olefinated Arenes. *ACS Catal.* **2023,** *13*, 14000-14011.
27. Bairagi, Y.; Porey, S.; Vummaleti, S. V. C.; Zhang, X.; Lahiri, G. K.; Maiti, D., Synthesis of β-(Hetero)aryl Ketones via Ligand-Enabled Nondirected C–H Alkylation. *ACS Catal.* **2024,** *14*, 15654-15664.

28. Barone, V.; Cossi, M., cossi-barone-1998-quantum-calculation-of-molecular-energies-and-energy-gradients-in-solution-by-a-conductor-solvent. *J. Phys. Chem. A* **1998,** *102*, 1995-2001.
29. Cossi, M.; Rega, N.; Scalmani, G.; Barone, V., Energies, structures, and electronic properties of molecules in solution with the C-PCM solvation model. *J. Comput. Chem.* **2003,** *24*, 669-681.
30. Boruah, A.; Boro, B.; Wang, J.; Paul, R.; Ghosh, R.; Mohapatra, D.; Li, P. Z.; Zhang, X.; Mondal, J., Influence of Keto-Enol Tautomerism in Regulating CO(2) Photoreduction Activity in Porous Organic Porphyrinic Photopolymers. *ACS Appl. Mater. Interfaces* **2025,** *17*, 1259-1272.
31. Grimme, S., Supramolecular binding thermodynamics by dispersion-corrected density functional theory. *Chem. Eur. J.* **2012,** *18*, 9955-9964.
32. Luchini, G.; Alegre-Requena, J. V.; Funes-Ardoiz, I.; Paton, R. S., GoodVibes: automated thermochemistry for heterogeneous computational chemistry data. *F1000Research* **2020,** *9*.
33. Bryantsev, V. S.; Diallo, M. S.; Goddard III, W. A., Calculation of Solvation Free Energies of Charged Solutes Using Mixed Cluster/Continuum Models. *J. Phys. Chem. B* **2008,** *112*, 9709-9719.
34. Schrödinger, L. The PyMOL Molecular Graphics Development Component, Version 1.8; 2015.
35. Ess, D. H.; Houk, K. N., Distortion-interaction-energy-control-of-1-3-dipolar-cycloaddition-reactivity. *J. Am. Chem. Soc.* **2007**, 10646-10647.
36. Bickelhaupt, F. M.; Houk, K. N., Analyzing Reaction Rates with the Distortion/Interaction-Activation Strain Model. *Angew. Chem. Int. Ed.* **2017,** *56*, 10070-10086.

Full reference Gaussian 16:

Gaussian 16, Revision B.01, Frisch, M. J.; Trucks, G. W.; Schlegel, H. B.; Scuseria, G. E.; Robb, M. A.; Cheeseman, J. R.; Scalmani, G.; Barone, V.; Mennucci, B.; Petersson, G. A.; Nakatsuji, H.; Caricato, M.; Li, X.; Hratchian, H. P.; Izmaylov, A. F.; Bloino, J.; Zheng, G.; Sonnenberg, J. L.; Hada, M.; Ehara, M.; Toyota, K.; Fukuda, R.; Hasegawa, J.; Ishida, M.; Nakajima, T.; Honda, Y.; Kitao, O.; Nakai, H.; Vreven, T.; Montgomery Jr., J. A.; Peralta, J. E.; Ogliaro, F.; Bearpark, M.; Heyd, J. J.; Brothers, E.; Kudin, K. N.; Staroverov, V. N.; Kobayashi, R.; Normand, J.; Raghavachari, K.; Rendell, A.; Burant, J. C.; Iyengar, S. S.; Tomasi, J.; Cossi, M.; Rega, N.; Millam, J. M.; Klene, M.; Knox, J. E.; Cross, J. B.; Bakken, V.; Adamo, C.; Jaramillo, J.; Gomperts, R.; Stratmann, R. E.; Yazyev, O.; Austin, A. J.; Cammi, R.; Pomelli, C.; Ochterski, J. W.; Martin, R. L.; Morokuma, K.; Zakrzewski, V. G.; Voth, G. A.; Salvador, P.; Dannenberg, J. J.; Dapprich, S.; Daniels, A. D.; Farkas, Ö.; Foresman, J.

B.; Ortiz, J. V; Cioslowski, J.; Fox, D. J. Gaussian, Inc., Wallingford CT, 2016.
